# Diversity-oriented synthesis of stereodefined tetrasubstituted alkenes via a modular alkyne *gem*-addition strategy

**DOI:** 10.1038/s41467-025-56184-3

**Published:** 2025-01-25

**Authors:** Xuan Di, Sitian Zhou, Yali Qin, Wenjun Li, Yue Zhang, Jie Zhang, Xu Shen, Jie Han, Jin Xie, Hongming Jin

**Affiliations:** 1 School of Pharmacy, Nanjing University of Chinese Medicine, Nanjing, China; 2 State Key Laboratory of Coordination Chemistry, Jiangsu Key Laboratory of Advanced Organic Materials, Chemistry and Biomedicine Innovation Center (ChemBIC), School of Chemistry and Chemical Engineering, Nanjing University, Nanjing, China; 3 Jiangsu Key Laboratory of Drug Target Research and Drug Discovery of Neurodegenerative Disease, School of Medicine, Nanjing University of Chinese Medicine, Nanjing, China; 4 State Key Laboratory of Natural Medicines, China Pharmaceutical University, Nanjing, China

**Keywords:** Synthetic chemistry methodology, Reaction mechanisms

## Abstract

Stereocontrolled construction of tetrasubstituted olefins has been an attractive issue yet remains challenging for synthetic chemists. In this manuscript, alkynyl selenides, when treated with ArBCl_2_, are subject to an exclusive 1,1-carboboration, affording tetrasubstituted alkenes with excellent levels of *E*-selectivity. Detailed mechanistic studies, supported by DFT calculations, elucidates the role of selenium in this 1,1-addition process. Coupled with subsequent C-B and C-Se bond transformations, this 1,1-addition protocol constitutes a modular access to stereodefined all-carbon tetrasubstituted alkenes. The merit of this approach is demonstrated by programmed assembly of diverse functionalized multi-arylated alkenes, especially enabling the stereospecific synthesis of all six possible stereoisomers of tetraarylethene (TAE) derived from the random permutation of four distinct aryl substituents around the double bond. The diversity-oriented synthesis is further utilized to explore different TAE luminogenic properties and potential Se-containing antitumor lead compounds.

## Abstract (web summary)

Tetrasubstituted alkenes are found in bioactive compounds and materials, but modular stereospecific synthesis of these compounds remains challenging due to reactivity and selectivity issues of the intermediate compounds. Here, the authors disclose a carboboration of alkynyl selenides, which provides an intermediate with two readily distinguishable sites of functionalization, providing rapid access to stereodefined tetrasubstituted alkenes.

## Introduction

**Fig. 1:**
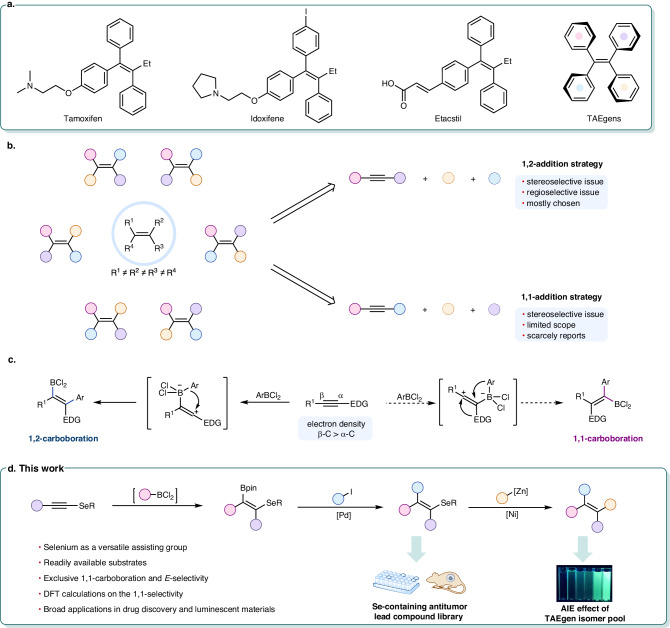
Diversity-oriented synthesis of stereodefined olefins with up to four different substituents. **a** Functionalized multi-arylated all-carbon tetrasubstituted alkenes. **b** Stereocontrolled synthesis of all-carbon tetrasubstituted alkenes from alkynes. **c** 1,1- vs. 1,2-selective carboboration of electron-rich alkynes. **d** This work: Modular alkyne *gem*-addition to access functionalized tetrasubstituted alkenes.

Multi-arylated all-carbon tetrasubstituted alkenes have been recognized as privileged motifs featured in drug molecules, aggregation-induced emission luminogens (AIEgens), and supramolecular materials (Fig. 1a). Specifically, triphenylethylene derivatives such as Tamoxifen and Idoxifene, which serve as nonsteroidal selective estrogen receptor modulators, have been approved for the therapy of breast cancer and myeloproliferative neoplasm^1–4^. Tetraarylethenes (TAE) also draw numerous attentions as prototypical AIEgens are widely used in the assembly of fluorescent probes^5–9^. Given the functional relevance of aryl substituents, olefins endowed with diverse—preferably all-different—aromatic rings are highly appealing for further exploring potential pharmaceutical activities and optoelectronic peculiarities. Of note, permuting four different substituents about the double bond can indeed boost the structural diversity of the scaffold, resulting in no less than six stereoisomers (Fig. 1b, left). Although extensive research on structure-property relationships has unveiled that the biochemical and physical properties of the isomers may be critically dependent on the order of substituents^10–13^, systematic comparative investigations of each isomer’s characteristics are limited due to the formidable synthetic challenges in the stereocontrolled construction of the *entire* isomer pool. Thus, a modular synthesis strategy to access the collection of stereodefined tetrasubstituted alkenes with maximal appendages and stereochemical diversity is in great demand^14–18^.

Currently, stereoselective approaches toward tetrasubstituted acyclic unsymmetrical alkenes generally begin with alkynes through a 1,2-difunctionalization^19–36^, followed by downstream unmasking reactions. In contrast to the dual rigorous control of stereo- and regiospecificity required in 1,2-addition protocols, only *E/Z*-selectivity is a concern in the 1,1-addition of alkynes^37–43^ (Fig. 1b, right). A few pioneering studies on the 1,1-carboboration of alkynes have shed light on the conceptual 1,1-addition strategy for achieving tetrasubstituted alkenes. Wrackmeyer reaction is the first 1,1-carboboration of internal alkynes involving trialkylboranes^44^, which has earned considerable importance due to its well-defined *gem*-addition paradigm with high stereoselectivity. Although Kehr and Erker et al. further expanded the scope and facilitated Wrackmeyer-type *gem*-additions under milder conditions with strongly Lewis-acidic B(C_6_F_5_)_3_^45–48^, the uneconomic cost, inconvenient derivatization, and low functional group compatibility of pentafluorophenyl boranes limit the synthetic value. In continuation of our interest in boron chemistry^49–51^, we envision that less aggressive ArBCl_2_^52^, readily obtainable from BCl_3_, could render the 1,1-carboboration of electron-rich alkynes viable. However, as exemplified by the 1,2-carboboration of ynamides via a keteniminium intermediate^53^, the polarized C≡C triple bond in electron-rich alkynes, possessing an electronic bias on the β carbon, is amenable to 1,2-addition by electrophiles^54–57^ (Fig. 1c, left).

Selenium is well-known for its unique biological functionality in modulating physiological processes^58^ and pharmacological activities^59^, whereas the selenium effect on chemical reactivity is rarely addressed. Herein, we develop an ArBCl_2_-promoted 1,1-carboboration reaction of alkynyl selenides (Fig. 1d). We thoroughly investigate the role of the selenium atom in the 1,1-selectivity, particularly in comparison with its sulfur analog, with the aid of density functional theory (DFT) calculations. By exploiting selenides as cross-coupling partners, the alkyne *gem*-addition strategy is successfully programmed into a diversity-oriented synthesis of all-carbon tetrasubstituted alkenes. Up to six tetra-arylated alkene isomers can be individually produced. Moreover, benefiting from this methodology, a set of Se-substituted triphenylethylene derivatives thus formed exhibits potent antitumor activity in vitro as well as in vivo, which demonstrates the relevance of this methodology in medicinal chemistry^60,61^.

## Results and discussion

### Reaction design with DFT calculations

**Fig. 2:**
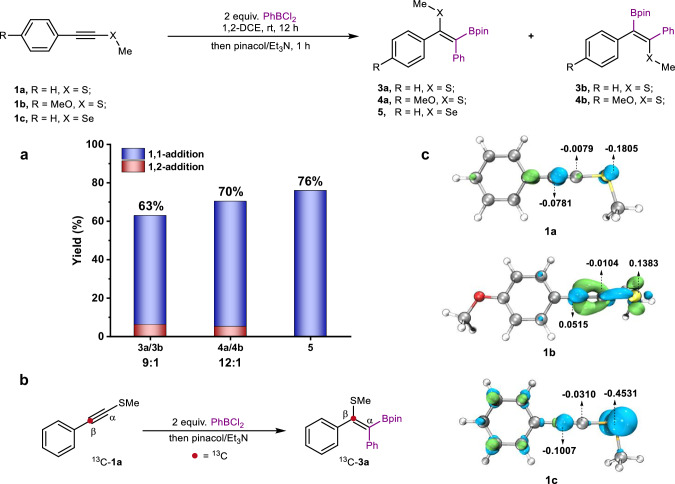
Examinations on electron-rich alkynes. **a** Reaction yields and selectivity. **b** ^13^C-labeled experiment. **c** Isosurface of condensed dual descriptors (CDD) for **1a**, **1b**, and **1c**. Blue indicates nucleophilicity, while green represents electrophilicity. The negative and positive values (in a.u.) correspond to the overall nucleophilicity and electrophilicity condensed on each atom, respectively.

At the outset, we commenced with the treatment of alkynyl sulfide **1a** with 2 equivalent PhBCl_2_ in 1,2-dichloroethane (1,2-DCE) solvent. After quenching the reaction with pinacol/Et_3_N, a mixture of 1,1-addition and 1,2-addition products (**3a**/**3b** = 9:1) was obtained with a total yield of 63% (Fig. 2a). Using *p*-methoxy substituted alkynyl sulfide **1b** improved the ratio of 1,1-addition (**4a**/**4b** = 12:1), delivering a total yield of 70%. An experiment with a ^13^C-labeled substrate indicated that the 1,1-carboboration takes place on the α-carbon of C≡C triple bond, undergoing an intramolecular sulfur migration (Fig. 2b). Combining these results with DFT calculations, the effect of the methoxy group on the addition selectivity was elucidated by condensed dual descriptors (CDD) of **1a** and **1b** (Fig. 2c). It is noticed that an electronic bias induced by the electron-donating methoxy group not only enhances the nucleophilicity of the α-carbon atom in **1b** compared to **1a** but also endows the β-carbon atom with some electrophilicity. For comparison with alkynyl sulfides, CDD was also performed on alkynyl selenide **1c**. The selenium atom proves to be much more nucleophilic than sulfur, increasing the electron density of the α-carbon atom through a p-π conjugation effect. On the basis of these preliminary computational results, we focused on the reaction of PhBCl_2_ with **1c**. To our delight, only the 1,1-carboboration *E*-product **5** was produced with a 76% yield. Various solvents were tested, and 1,2-DCE proved the most effective (Table S1). Adjustments in reaction time and the amount of PhBCl_2_ equivalents did not significantly raise the yield.

**Fig. 3:**
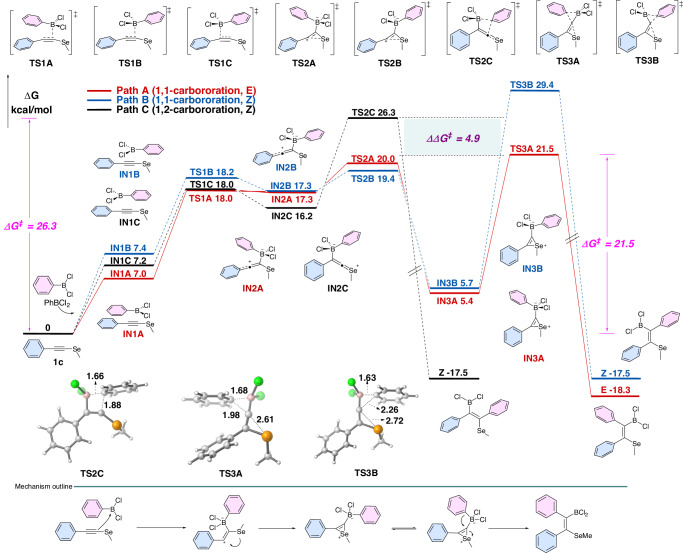
Computational mechanistic study of carboboration of 1c. Free-energy profiles of reaction pathways for carboboration of alkynyl selenide (**1c**) computed at the B3LYP(D3BJ)/def2-TZVP/PCM(DCE)//M06-2X/def2-TZVPP/SMD (1,2-DCE) level of theory. The geometries of the key transition states are illustrated below, with the bond lengths indicated in Angstrom (Å).

To gain deeper insight into the reaction mechanism and clarify the selenium effect on the specificity of 1,1-carboboration, DFT calculations on reaction pathways of alkynyl selenide **1c** were carried out (Fig. 3). We investigated the pathways for 1,1-carboboration leading to the *E*- (Path A) or *Z*-configured (Path B) product, as well as 1,2-carboboration producing a *Z*-configured product (Path C). In Path A, the 1,1-addition reaction of **1c** is initiated by the coordination of PhBCl_2_ to the alkyne. Subsequently, the α-carbon of **1c** is subjected to the electrophilic addition of PhBCl_2_, forming a zwitterionic intermediate **IN2A**. Owing to the strong nucleophilicity and large diameter of selenium, a three-membered selenonium ion **IN3A** is formed with a free-energy change of only 5.4 kcal/mol. The *E*-product **3c** is then generated through an intramolecular 1,2-migration of the boronate complex, crossing the rate-determining transition state **TS3A** (21.5 kcal/mol). Alternatively, the formation of *Z-*product via Path B is kinetically unfavorable, requiring a higher free-energy barrier of 29.4 kcal/mol (**TS3B**). The coordination effect of Se to boron also facilitates the formation of a thermodynamically stable *E*-product. Distortion-interaction analysis and the highest occupied molecular orbital (HOMO) of **TS3A** and **TS3B** suggest that selenonium plays a critical role in favoring the *E*-selectivity of the 1,1-caboboration process (Table S5). Furthermore, the divergent route (Path C) to the 1,2-carboboration of **1c** was examined. The electrophilic addition of PhBCl_2_ to the β-carbon could produce another zwitterionic species **IN2C**. Then, an intramolecular 1,3-phenyl migration occurs via **TS2C** with a free activation energy of 26.34 kcal/mol, yielding the 1,2-addition product. Therefore, Path A and Path C are the most favorable pathways for generating *E*- and *Z*-products. In line with the Curtin-Hammett principle, the ΔΔ*G*^*‡*^ value of 4.9 kcal/mol (**TS2C** relative to **TS3A**) ensures the reaction preference for the *E*-product of 1,1-carboboration, which aligns well with the experimental results.

**Fig. 4:**
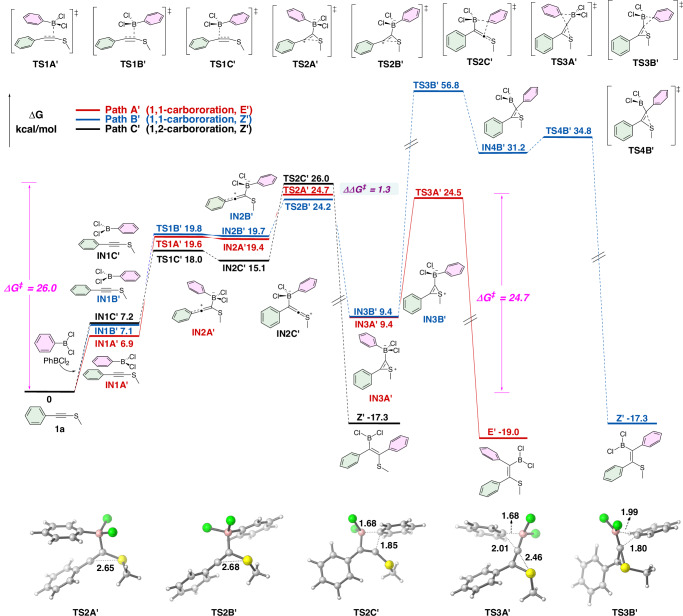
Computational mechanistic study of carboboration of 1a. Free-energy profiles of reaction pathways for carboboration of alkynyl sulfide (**1a**) computed at the B3LYP(D3BJ)/def2-TZVP/PCM(DCE)//M06-2X/def2-TZVPP/SMD (1,2-DCE) level of theory. The geometries of the key transition states are illustrated below, with the bond lengths indicated in Angstrom (Å).

The reaction progress of alkynyl sulfide **1a** was also calculated to compare it with the above-mentioned mechanism of the alkynyl selenide **1c** (Fig. 4 and Fig.  S5). Again, Path B’ can be ruled out due to its excessively high overall energy barrier (Δ*G*^‡^ = 56.8 kcal/mol). Path A’ and Path C’ are the preferred routes to the *E*-product (**E’**) and *Z*-product (**Z’**), respectively. It is worth to note that in the case of **1a**, the highest energy barrier for accessing the 1,1-carboboration *E*-product involves the generation of sulfonium ion via **TS2A’** (24.7 kcal/mol), rather than the 1,2-migration of boronate complex via **TS3A’** (24.5 kcal/mol). Additionally, the 1,2-carboboration intermediate **IN2C’** is more stable than **IN2A’** by 4.3 kcal/mol. The longer C-Se bond length in **TS3A** (2.61 Å) compared to the C-S bond length in **TS3A’** (2.46 Å) further accounts for that the concerted ring opening process may be easier for the selenonium ion than the sulfonium ion. The ΔΔ*G*^‡^ value of 1.3 kcal/mol (**TS2C’** relative to **TS2A’**) is lower than that observed for **1c**, suggesting the reduced selectivity.

### Reaction scope

**Fig. 5:**
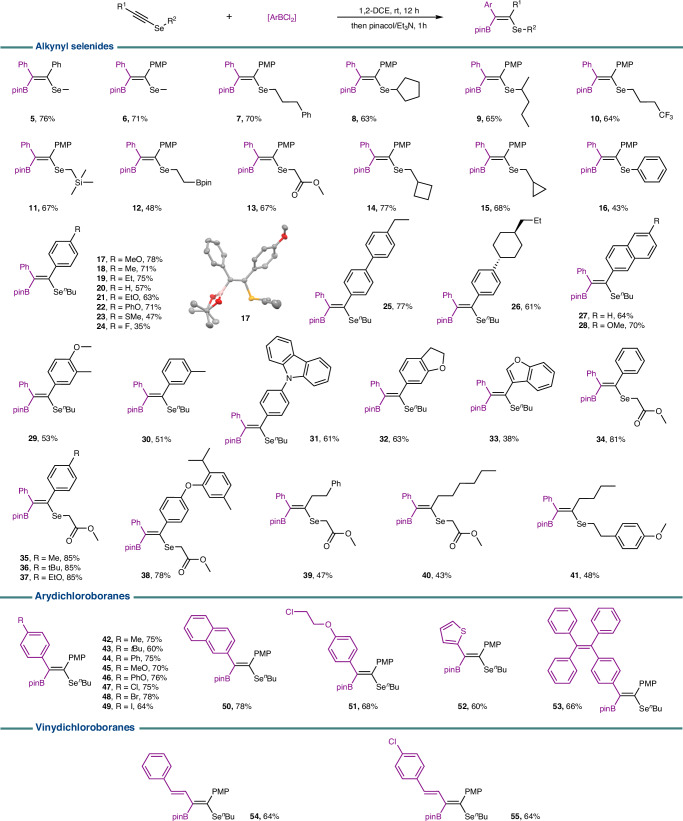
Substrate scope. General reaction conditions: alkynes (0.2 mmol), ArBCl_2_ (0.4 mmol), 1,2-DCE (1 mL), room temperature, 12 h, then quenching with pinacol/Et_3_N. Isolated yield. Except for commercially available PhBCl_2_, another ArBCl_2_ was generated in situ through the reaction of ArTMS with BCl_3_. PMP *p*-methoxyphenyl.

Under the optimal reaction conditions, the scope of alkynyl selenides was explored (Fig. 5). An assortment of primary and secondary alkyl-substituted selenides proved effective, giving moderate to good yields. Various functional groups, including CF_3_, TMS, Bpin, and esters, were well tolerated (**10**–**13**), and strained small rings remained intact (**14,** **15**). The phenyl substituent on the selenium atom was found to attenuate the nucleophilicity of selenium, decreasing the yield of **16**. Then, variants of the alkynyl moiety were examined. Generally, aromatic rings with electron-donating groups provided higher yields (**17**–**23**). The Lewis acid-sensitive substituents containing N, O, and S atoms turned out to be endurable as well (**23,** **31**–**33**, **38**). Besides aryl alkynes, an array of alkyl-substituted alkynes reacted smoothly, although they led to more moderate yields (**39**–**41**). The configuration of product **17** (CCDC 2370343) was confirmed by a single-crystal X-ray diffraction. Next, a wide range of aryldichloroboranes was surveyed, which can be readily generated in situ by reacting corresponding trimethyl(aryl)silanes with BCl_3_. Unexpectedly, the methoxy group on benzene proved stable in the presence of BCl_3_, furnishing a 70% yield of **45**. Other compatible functional groups, such as *t*-Bu (**43**), halogens (**47**–**49**), thiophene (**52**), naphthalene (**50**), and TPEgen (**53**), further augmented the peripheral diversity. In addition, polysubstituted conjugated dienes were accessible by utilization of alkenyl dichloroboranes (**54,** **55**).

**Fig. 6:**
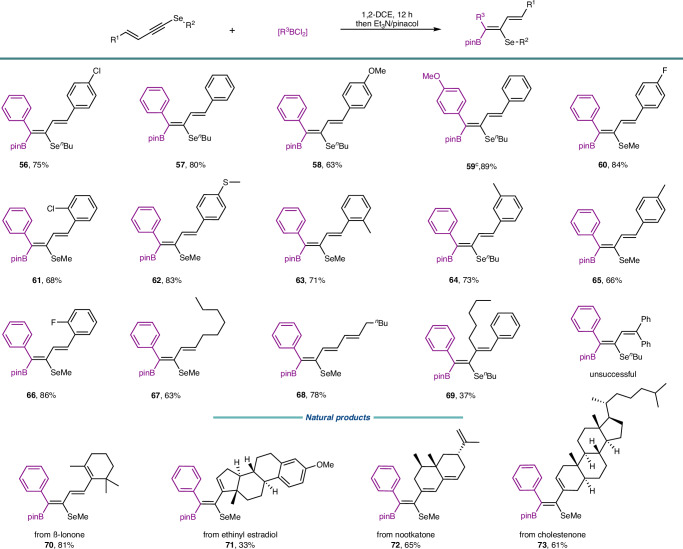
Synthesis of polysubstituted 1,3-dienes. General reaction conditions: alkynes (0.2 mmol), R^3^BCl_2_ (0.4 mmol), 1,2-DCE (1 mL), room temperature, 12 h, then quenching with pinacol/Et_3_N. Isolated yield. In situ generation of *p*-MeO-PhBCl_2_ through the reaction of *p*-MeO-PhTMS with BCl_3_.

Considering that stereodefined conjugated dienes are advanced building blocks in synthetic chemistry, an alternative approach for the expedient preparation of polysubstituted 1,3-dienes from 1,3-enynes was devised (Fig. 6). A variety of 1,3-enynyl selenides bearing either electron-withdrawing or electron-donating groups exhibited excellent performance, leading to high yields (**56**–**66**). The configuration of the double bond in substrates was retained. Due to steric hindrance, trisubstituted 1,3-enyne substrates typically afforded products in moderate yields (**69**). Moreover, the linear conjugated triene **68** was procured in a 78% yield. The 1,1-diphenyl 1,3-enyne is not successful. The method is also suitable for the late-stage modification of complex natural product derivatives, resembling the frameworks of β-lonone, nootkatone, ethinyl estradiol, and cholestenone (**70**–**73**).

### Derivations and applications

**Fig. 7:**
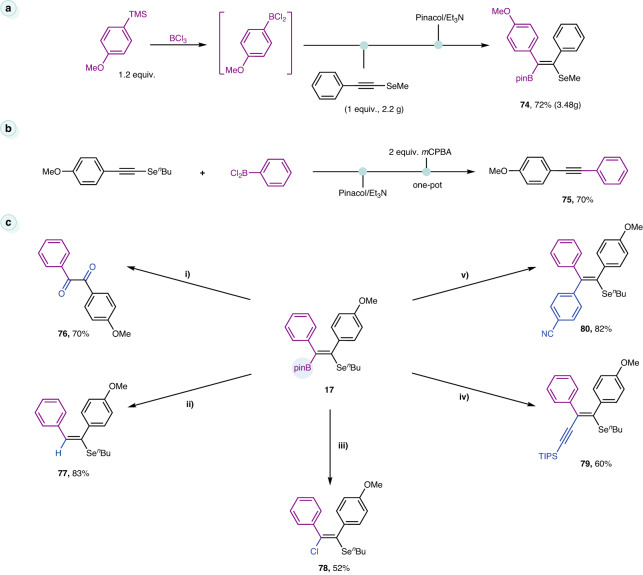
Derivations. **a** Scale-up synthesis. **b** Transition metal-free Suzuki-type cross-coupling. **c** Downstream transformations of alkenyl boronate: (i) KOAc (3 equiv.), 30% H_2_O_2_ (3 equiv.), 0 °C to rt, 20 h; (ii) TBAF•3H_2_O, 45 °C, 16 h; (iii) CuCl_2_ (5 equiv.), MeOH/H_2_O, 24 h; (iv) 5 mol% Pd_2_(dba)_3_, 20 mol% P(furan)_3_, NaO^*t*^Bu, (bromoethynyl)triisopropylsilane (3 equiv.), THF/PhMe, 75 °C, 20 h; (v) 10 mol% PdCl_2_(dppf), Cs_2_CO_3_ (2 equiv.), 4-iodobenzonitrile (3 equiv.), 50 °C, 1,4-dioxane, 36 h.

The scalability and robustness of this protocol have been reinforced by a gram-scale reaction of **1c** (2.2 g), offering the product in 72% yield, even when the amount of ArBCl_2_ was decreased to 1.2 equivalents (Fig. 7a). A net aryl-alkyne coupling product was obtained in 70% yield through the cascade reaction of **17** with *m*-CPBA (**75**). The two-step, one-pot reaction features a transition metal-free formal Suzuki cross-coupling (Fig. 7b). A series of product derivatizations took advantage of the rich downstream chemistry of alkenyl boronates (Fig. 7c). Firstly, treatment of **17** with H_2_O_2_ produced a 1,2-diketone (**76**). In the presence of TBAF•3H_2_O, trisubstituted alkene was formed at the expense of the Bpin (**77**). Likewise, tetrasubstituted alkenyl monochloride was available from the alkenyl boronate using CuCl as a benign chlorinating agent (**78**). Finally, an aryl and an alkynyl group were efficiently attached to the double bond via Pd-catalyzed Suzuki-Miyaura cross-coupling with yields of 82% and 60%, respectively (**79**, **80**).

**Fig. 8:**
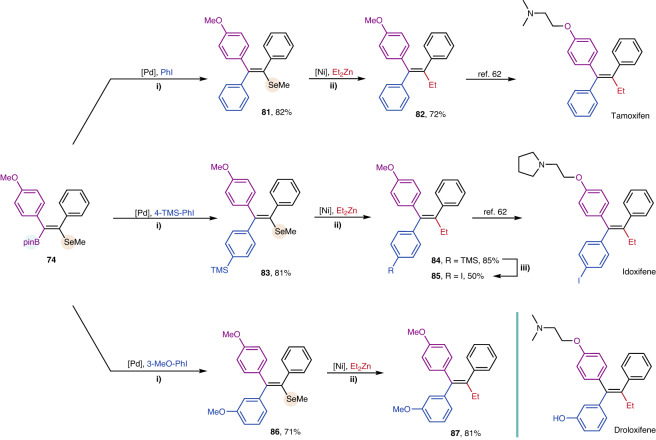
Modular synthesis of drug molecules based on the C-Se bond transformation: (i) 10 mol% PdCl_2_(dppf), 9 M aqueous NaOH (4 equiv.), aryl iodide (2 equiv.), in THF, rt, 1 h; (ii) 15 mol% NiCl_2_(dppe), 1 M Et_2_Zn in ether, 50 °C, 15 h; (iii) ICl (2 equiv.), in DCM, −50 °C to 0 °C, 36 h.

To further illustrate the versatility and synthetic potential of the methodology, the following C-Se bond transformations employing selenide as a removable assisting group were conducted (Fig. 8). Despite scarce literature on C-Se bond activation, we developed direct C-C coupling reactions of alkenyl selenides-based on known Ni-catalyzed C-S bond activation with a few alterations to the reaction conditions (Table S2). Compound **81** was subject to a Ni-catalyzed cross-coupling with Et_2_Zn, enabling a stereospecific synthesis of Tamoxifen without the formation of other stereoisomeric byproducts^62^. The analogous frameworks of Idoxifene and Droloxifene were also assembled modularly to validate the applicability of this approach.

### AIEgens synthesis and fluorescence characterizations

**Fig. 9:**
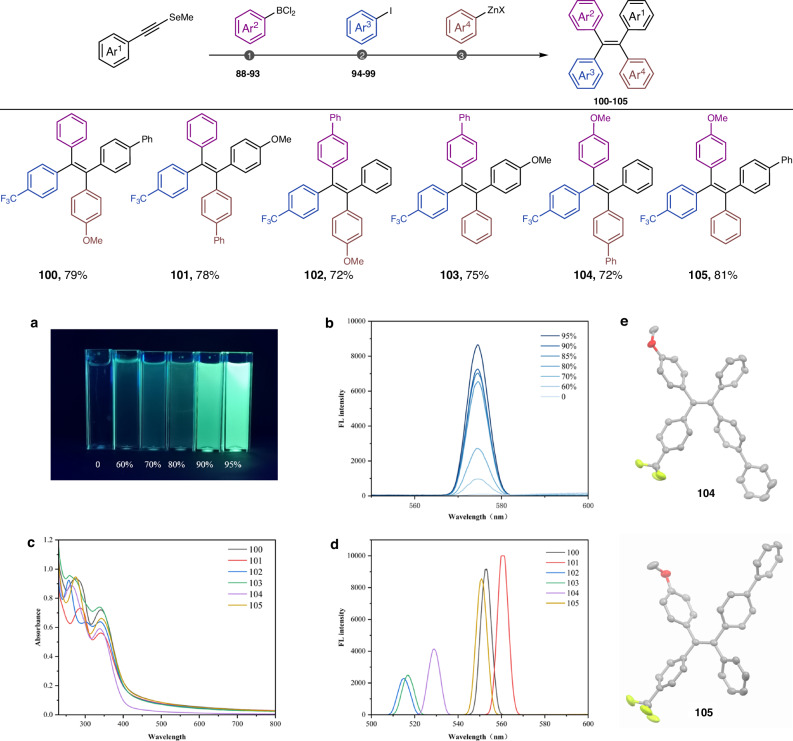
Programmed synthesis of up to six TAE isomers and fluorescence determinations. **a** Fluorescence photographs of **100** in THF/H_2_O mixtures with different H_2_O fractions, [c] = 2 µM. **b** FL intensity of **100** with different H_2_O fractions, [c] = 2 µM. **c** UV–Vis spectra of **100**–**105**, [c] = 20 µM, 95% H_2_O. **d** Fluorescence spectra of **100**–**105**, [c] = 2 µM, 95% H_2_O. **e** Solid-state molecular structure of **104** and **105**.

The alkyne *gem*-addition strategy was then devoted to the programmed construction of stereodefined alkenes with up to four different aryl substituents. Aryl zinc reagents were coupled with the selenides under nickel catalysis (Table S3). Diverse aromatic rings with distinct electrostatic properties could be sequentially installed in just 3 steps, giving rise to a library of all six possible alkene isomers with a donor-π-acceptor structure. The 3 steps procedure is the shortest linear synthesis protocol for stereodefined olefins with four distinct aryl groups. Although previous methodologies also afforded efficient approaches to achieve tetraarylethylenes with excellent stereoselectivity, a small amount of regioselective isomers is still inevitable. Moreover, compared with the prior synthesis from moisture-sensitive alkynyl boron substrates^20^, the alkynyl selenides are easily prepared, isolated, and handled. As manifested in Fig. 9, the luminescence of compound **100** in THF/H_2_O mixtures was gradually enhanced with increasing water content, revealing an obvious AIE effect (Fig. 9a, b). The UV/Vis and fluorescence spectra of all six isomers were comprehensively assessed (Fig. 9c, d). Apparently, fluorescence intensity, emission wavelength, and Stokes shift were prominently affected by the simple permutation of the aryl positions. A fluorescence red shift was observed in the compounds **100** and **101** compared to other isomers. The solid-state molecular structures of **104** and **105** (CCDC 2369379, 2369378) were identified by single-crystal X-ray diffraction (Fig. 9e).

### Evaluation of antitumor activity

**Fig. 10:**
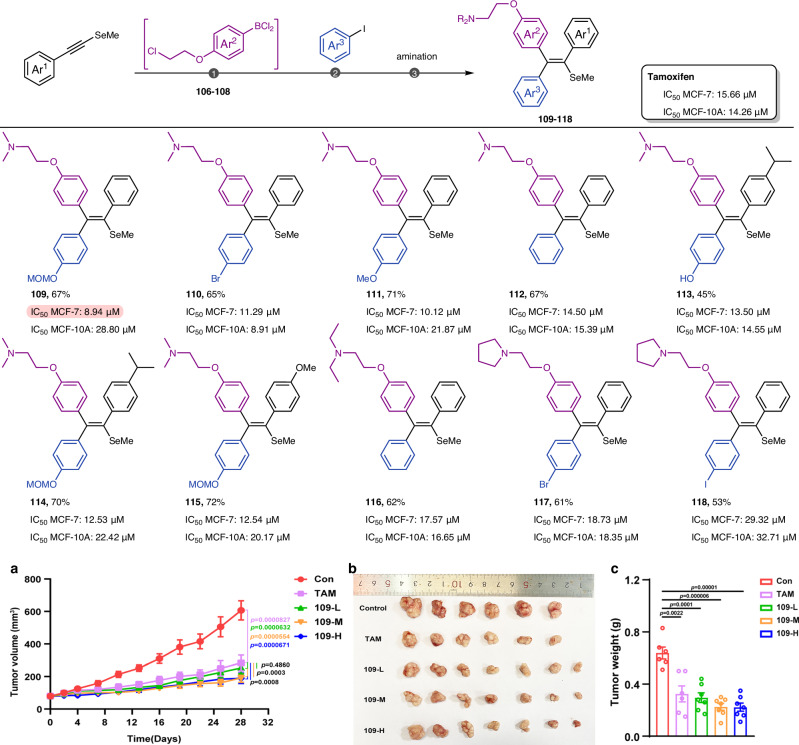
Diversified construction of Se-containing antitumor compound library and evaluation of antitumor activities. **a** Tumor volume changes by treatment with different dosages of compound **109** in MCF-7 xenograft model mice compared to Tamoxifen. All values were presented as mean ± SEM. Significance was determined using Two-way ANOVA followed by Tukey’s multiple comparison test with two-tailed adjusted *p*-values displayed in the figure (con vs. **TAM**, *p* = 0.0000827, 95% confidence interval (CI): 92.91 to 147.4; con vs. **109-L**, *p* = 0.0000632, 95% CI: 109.1 to 163.1; con vs. **109-M**, *p* = 0.0000554, 95% CI: 135.1 to 189.2; con vs. **109-H**, *p* = 0.0000671, 95% CI: 132.5 to 187.6; **TAM** vs. **109-L**, *p* = 0.4860, 95% CI: −11.08 to 42.99; **TAM** vs. **109-M**, *p* = 0.0003, 95% CI: 15.00 to 69.06; **TAM** vs. **109-H**, *p* = 0.0008, 95% CI: 12.32 to 67.47). Data are *n* = 6 (con group and **TAM** group) or *n* = 7 (**109-L**, **109-M**, and **109-H** group) biological replicates, one independent experiment. **b** Photographs of all MCF-7 xenograft tumors resected on day 28. **c** Measured weight of the resected MCF-7 xenograft tumors on day 28. All values were presented as mean ± SEM. Significance was determined using a two-tailed unpaired *t*-test with *p*-values displayed in the figure (con vs. **TAM**, *p* = 0.0022; con vs. **109-L**, *p* = 0.0001; con *vs*. **109-M**, *p* = 0.000006; con *vs*. **109-H**, *p* = 0.00001). Data are *n* = 6 (con group and **TAM** group) or *n* = 7 (**109-L**, **109-M**, and **109-H** group) biological replicates, one independent experiment. **TAM** = 13.4 µmol/kg; **109-L** = 6.6 µmol/kg; **109-M** = 13.3 µmol/kg; **109-H** = 26.6 µmol/kg.

As a chalcogen on the periodic table, selenium is commonly chosen as an alternative to sulfur and oxygen atoms in drug design^59^. However, the incorporation of selenium as a substitute for carbon in drug molecules is rarely reported. To enrich the Se-based compound library, a suite of Tamoxifen analogs featuring selenium instead of the allylic CH_2_ group was prepared, and their anti-breast cancer activities were tentatively evaluated in MCF-7 cells (Fig. 10). Compared to tamoxifen as the positive control, seven compounds proved to have superior antitumor activity. Among these, compound **109** exhibited the most potent tumor inhibitory effect on MCF-7 cell growth, with an IC_50_ value of 8.94 μM. The cytotoxicity of the normal human mammary epithelial cell (MCF-10A) was examined. Notably, the compound **109** has lower cytotoxicity to the normal cell than Tamoxifen.

To evaluate the potential of compound **109** in suppressing tumor growth in vivo, a subcutaneous MCF-7 xenograft tumor model in nude mice was utilized (Approval No. 202406A093). The findings demonstrated that treatments with compound **109** markedly decreased both the volume (Fig. 10a, b) and weight (Fig. 10c) of transplanted tumors. **109** showed a greater inhibitory effect than the equal molar concentration of tamoxifen (**TAM** vs. **109-M**), and the lower concentration of **109** performed closely to tamoxifen (**TAM** vs. **109-L**). Notably, the treatment by **109** did not result in significant changes in the body weight of the nude mice. Additionally, histopathological analysis using HE staining revealed no severe side effects on normal tissues, including the liver and kidneys (Figs. S1–S4).

In summary, a practicable electrophilic *gem*-addition reaction of alkynyl selenides with aryldichloroboranes has been developed for the stereospecific construction of tetrasubstituted alkenes. The role of selenium in the carboboration mechanism and selectivity was elaborated through a combination of experimental results with DFT calculations. This method features a broad substrate scope, impressive functionality tolerance, and high scalability, making it suitable for a wide range of synthetic applications. The versatility of the resulting alkenyl boronates as building blocks enabled the assembly of various functionalized alkynes, ketones, and alkenes. By taking advantage of following explored C-Se bond transformations, robust modular access to stereodefined all-carbon tetrasubstituted alkenes was established, facilitating the diversity-oriented synthesis of Tamoxifen frameworks and TAE fluorogens with up to four different aryl groups. The utility of this approach was further extended to the exploration of Se-containing antineoplastic lead compounds, highlighting the favorable properties of selenium again.

## Methods

### Representative procedure for the scale-up synthesis

An oven-dried Schlenk tube equipped with a magnetic stir bar was charged with (4-methoxyphenyl)trimethylsilane (13.6 mmol, 2.45 g) and BCl_3_ (27.2 mmol, 1 M in DCM, 27.2 mL) at 0 °C under N_2_ atmosphere. The mixture was stirred at room temperature for 24 h. Subsequently, the excess BCl_3_ and solvent were removed under reduced pressure conditions. After refilling the tube with N_2_ atmosphere, the methyl(phenylethynyl)selane (11.3 mmol, 2.2 g) in anhydrous 1,2-dichloroethane (6 mL) was added. The mixture was stirred for 12 h, and then the pinacol (56 mmol, 6.6 g) in 6 mL Et_3_N was introduced. After continuously stirring for 1 h, the reaction was quenched with saturated NH_4_Cl solution (150 mL), followed by extraction with EtOAc (3 × 50 mL) in a separatory funnel. The combined organic layer was dried over anhydrous Na_2_SO_4_, filtered, and concentrated in vacuo. The residue was purified by flash column chromatography on silica gel to afford the desired product **74** in 72% yield (3.48 g).

## Supplementary information

NCOMMS-24-50497-T-s01

NCOMMS-24-50497-T-s02

NCOMMS-24-50497-T-s03

NCOMMS-24-50497-T-s04

NCOMMS-24-50497-T-s05

NCOMMS-24-50497-T-s06

NCOMMS-24-50497-T-s07

## Data Availability

CCDC 2370343 (**17**), 2369379 (**104**), and 2369378 (**105**) contain supplementary crystallographic data for this paper. The data can be obtained free of charge from the Cambridge Crystallographic Data Centre via www.ccdc.cam.ac.uk/data_request/cif. All other data that support the findings of this paper, including detailed experimental procedures, biological data, compound characterizations, and DFT calculations, are available within the paper and its Supplementary Information.
