# Supplementary material for: Diversity-oriented synthesis of stereodefined tetrasubstituted alkenes via a modular alkyne gem-addition strategy
Source: Nat Commun. 2025 Jan 25;16:1025. doi: 10.1038/s41467-025-56184-3 (PMC11763084; doi:10.1038/s41467-025-56184-3)
Supplement: Supplementary file 1 — NCOMMS-24-50497-T-s01 [file 41467_2025_56184_MOESM1_ESM.pdf]

Supplementary Information

Diversity-Oriented Synthesis of Stereodefined Tetrasubstituted Alkenes via a Modular Alkyne *gem*-Addition Strategy

Xuan Di[1‡], Sitian Zhou[2‡], Yali Qin[1‡], Wenjun Li[3‡], Yue Zhang[3], Jie Zhang[1], Xu Shen[3], Jie Han[2*], Jin Xie[2,4*], Hongming Jin[1,3*]

[1]School of Pharmacy, Nanjing University of Chinese Medicine, Nanjing, China
[2]State Key Laboratory of Coordination Chemistry, Jiangsu Key Laboratory of Advanced Organic Materials, Chemistry and Biomedicine Innovation Center (ChemBIC), School of Chemistry and Chemical Engineering, Nanjing University, Nanjing, China
[3]School of Medicine, Jiangsu Key Laboratory of Drug Target Research and Drug Discovery of Neurodegenerative Disease, Nanjing University of Chinese Medicine, Nanjing, China
[4]State Key Laboratory of Natural Medicines, China Pharmaceutical University, Nanjing, China
[‡]These authors contribute equally to this work.

Contents

1. General Methods S1
2. Reaction Optimization S2
3. Experimental Procedures S4
4. Computational Studies S13
5. Characterization S16
6. NMR Spectra S74
7. Supporting Crystallographic Data S214
8. References S217

1. General Methods

Unless stated otherwise, all reactions were carried out under Argon atmosphere in flame-dried glassware. Terminal alkynes, *n*-BuLi (2.5 M in hexane), $PhBCl_2$, Se powder and various metal catalysts are bought from Adamas, TCI and Sigma-Aldrich. Deuterated solvents were bought from Adamas. NMR spectra were, if not mentioned otherwise, recorded at room temperature on the following spectrometers: Bruker Avance-III-500. Chemical shifts are given in ppm and coupling constants in Hz. The following abbreviations were used for ^{1}H NMR spectra to indicate the signal multiplicity: s (singlet), brs (broad singlet), d (doublet), t (triplet), q (quartet), quint (quintet), sext (sextet), sept (septet) and m (multiplet) as well as combinations of them. When combinations of multiplicities are given the first character noted refers to the biggest coupling constant. All ^{13}C NMR spectra were measured with ^{1}H-decoupling. Mass spectra (MS and HRMS) were measured on an Agilent 6546 TOF LC-MS spectrometer. Infrared X-ray crystal structure analyses were measured on a Bruker D8 Quest instrument using Mo-K_{α}-radiation. Diffraction intensities were corrected for Lorentz and polarization effects. An empirical absorption correction was applied using SADABS based on the Laue symmetry of reciprocal space. Heavy atom diffractions were solved by direct methods and refined against F2 with full matrix least square algorithm. Hydrogen atoms were either isotropically refined or calculated. The structures were solved and refined using the SHELXTL software package. Melting Points were measured in open glass capillaries in a Büchi melting point apparatus. Flash Column Chromatography was accomplished using Silica gel 60 (0.04 - 0.063 mm / 230 - 400 mesh ASTM) purchased from Santai Science Inc. or Aluminium oxide (neutral or basic) purchased from Santai Science Inc.. As eluents, mixtures of petroleum ether (PE), ethyl acetate (EA), dichloromethane (DCM) and methanol (MeOH) were used. Analytical Thin Layer Chromatography (TLC) was carried out on precoated Yantai POLYGRAM® SIL G/UV254 or POLYGRAM® ALOX N/UV254 plastic sheets. Detection was accomplished using UV-light (254 nm), $KMnO_4$ (in 1.5M Na_2CO_3 (aq.)), molybdatophosphoric acid (5 % in ethanol), vanillin/H_2SO_4 (in ethanol) or anisaldehyde/HOAc (in ethanol). IUPAC names of the compounds described in the experimental section were determined with the program ACDLabs 12.0®.

2. Reaction Optimization

2.1 Table S1[a, b].

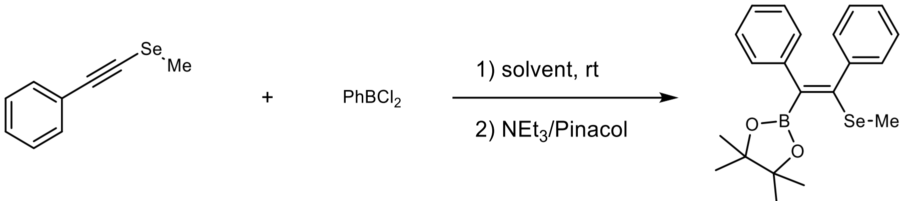

1c 2a 5

Entry[a]	Solvent	Time	X equiv. 2a	Yield%
1	1,2-DCE	12 h	2.0 equiv.	76%
2	DCM	12 h	2.0 equiv.	45%
3	PhF_3	12 h	2.0 equiv.	46%
4	THF	12 h	2,0 equiv.	NR[g]
5	CH_3CN	12 h	2.0 equiv.	NR[g]
6	1,2-DCE	12 h	1.2 equiv.	65%
7	1,2-DCE	12 h	1.5 equiv.	73%
8	1,2-DCE	12 h	3.0 equiv.	77%
9	1,2-DCE	1 h	2.0 equiv.	42%
10	1,2-DCE	3 h	2.0 equiv.	59%
11	1,2-DCE	6 h	2.0 equiv.	73%
12[c]	1,2-DCE	12 h	2.0 equiv.	61%
13[d]	1,2-DCE	12 h	2.0 equiv.	44%
14[e]	1,2-DCE	12 h	2.0 equiv.	40%
15[f]	1,2-DCE	12 h	2.0 equiv.	23%

[a]Reaction conditions: **1c** (0.20 mmol), **2a** (0.40 mmol) in 1,2-DCE (1 mL), rt, 12 h, N_2 atmosphere, then pinacol (1.0 mmol), NEt_3 (0.5 mL), rt, 1 h. [b]Isolated yields. [c]iPr_2NEt instead of Et_3N. [d]Pyridine instead of Et_3N. [e]DBU instead of Et_3N. [f]Piperidine instead of Et_3N. [g]NR = no reaction.

2.2 Table S2[a, b].

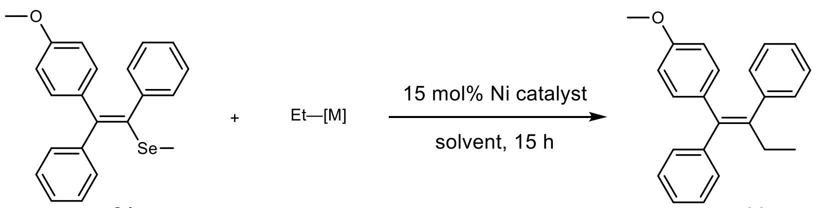

81 82

Entry	Et-[M]	Ni catalyst	Ligand	Solvent	T (℃)	Yield%

1	2 equiv. EtMgBr	$Ni(COD)_2$[c]	Dcype[c]	Toluene	90	20
2	10 equiv. EtMgBr	$NiCl_2$(dppe)	/	Et_2O	25	0
3	10 equiv. EtMgBr	$NiCl_2$(dppe)	/	Et_2O	50	31
4	5 equiv. $Zn(Et)_2$	$NiCl_2$(dppe)	/	Et_2O	50	50
5	3 equiv. $Zn(Et)_2$	$NiCl_2$(dppe)	/	Et_2O	50	72
6	3 equiv. $Zn(Et)_2$	$NiCl_2$(dppe)	/	Et_2O	25	0
7	3 equiv. $Zn(Et)_2$	$NiCl_2$(dppe)	/	Et_2O	45	50
8	3 equiv. $Zn(Et)_2$	$NiCl_2$(dppe)	/	THF	60	63

[a]Reaction conditions[1, 2]: **81** (0.2 mmol), [Ni] (15 mol%), solvent (2 mL), N_2 atmosphere, 15 h, in sealed tube. [b]Isolated yields. [c]5 mol% $Ni(COD)_2$ & Dcype. Dcype = 1,2-Bis(dicyclohexylphosphino)ethane.

2.3 Table S3[a, b].

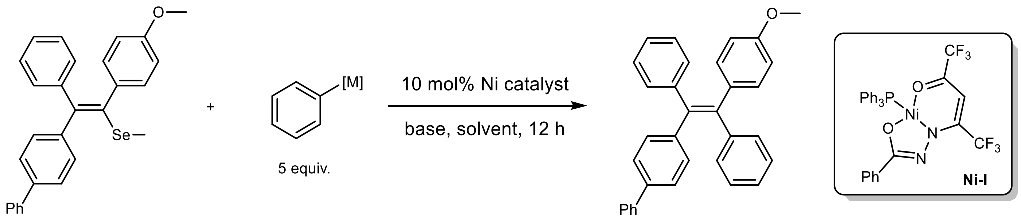

Entry	5 equiv. Ph-[M]	Ni catalyst	Ligand	Base	Solvent	T (℃)	Yield%
1	PhMgCl	$Ni(COD)_2$	/	/	Et_2O	50	29
2	PhMgCl	$NiCl_2$(dppe)	/	/	Et_2O	50	14
3	PhMgCl	NiF_2	/	/	Et_2O	50	30
4	PhMgCl	NiF_2	PPh_3	/	Et_2O	25	trace
5	PhMgCl	NiF_2	dppe	/	Et_2O	25	trace
6	PhMgCl	NiF_2	Xphos	/	Et_2O	25	trace
7	PhMgCl	NiF_2	Sphos	/	Et_2O	25	trace
8	PhZnCl	NiF_2	/	/	Et_2O	50	82
9	PhZnCl	Ni-I	/	LiO*t*Bu	THF	70	91
10	PhMgCl	Ni-I	/	LiO*t*Bu	THF	70	81

[a]Reaction conditions[3-5]: alkenyl selenide (0.2 mmol), [Ni] (10 mol%), ligand (10 mol%), base (0.3 mmol), solvent (2 mL), N_2 atmosphere, 12 h in sealed tube. [b]Isolated yields.

3. Experimental Procedures

3.1 General procedures for the synthesis of alkynyl selenides[6-10]:

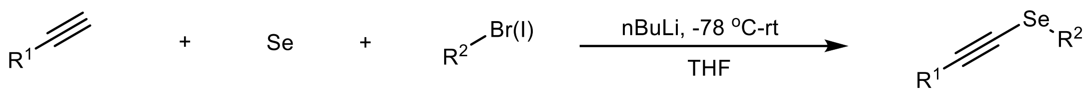

General procedure A: Under an argon atmosphere, an oven-dried Schlenk tube with a magnetic stir bar was charged with alkyne (2.0 mmol) and 10 mL anhydrous THF. n-BuLi (2.50 M in THF, 2.2 mmol) was then added at –78 °C, and the mixture was stirred for 30 minutes at the same temperature. Subsequently, Se powder (2.0 mmol, 158 mg) was added slowly at –78 °C. After continuous stirring for 1 hour at –78 °C, alkyl bromide (2.4 mmol) was introduced, and the mixture was allowed to warm to room temperature. The reaction was monitored by thin-layer chromatography and quenched with saturated NH_4Cl solution, followed by extraction with EtOAc (3×30 mL) in a separatory funnel. The combined organic layer was dried over anhydrous Na_2SO_4, filtered and concentrated in vacuo. The residue was purified by flash column chromatography on silica gel to afford the desired product.

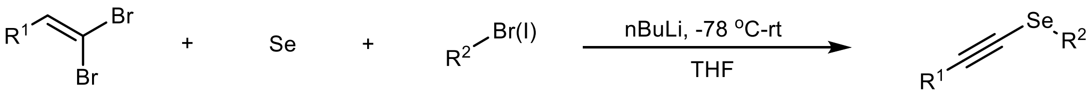

General procedure B: Under an argon atmosphere, an oven-dried Schlenk tube with a magnetic stir bar was charged with *gem*-dibromo alkene (2.0 mmol) and 10 mL anhydrous THF. n-BuLi (2.50 M in THF, 4.4 mmol) was then added at –78 °C, and the mixture was stirred for 30 minutes at – 40 °C. Subsequently, Se powder (2.0 mmol, 158 mg) was added slowly at –78 °C. After continuous stirring for 1 hour at –78 °C, alkyl bromide (2.4 mmol) was introduced, and the mixture was allowed to warm to room temperature. The reaction was monitored by thin-layer chromatography and quenched with saturated NH_4Cl solution, followed by extraction with EtOAc (3×30 mL) in a separatory funnel. The combined organic layer was dried over anhydrous Na_2SO_4, filtered and concentrated in vacuo. The residue was purified by flash column chromatography on silica gel to afford the desired product.

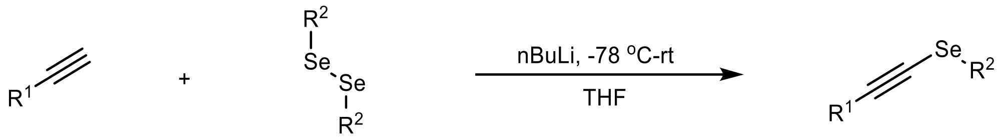

General procedure C: Under an argon atmosphere, an oven-dried Schlenk tube with a magnetic stir bar was charged with alkyne (2.0 mmol) and 10 mL anhydrous THF. n-BuLi (2.50 M in THF, 2.2 mmol) was then added at –78 °C, and the mixture was stirred for 30 minutes at the same temperature. Subsequently, R^2SeSeR^2 (2.2 mmol) was added slowly at –78 °C, and the mixture was allowed to warm to room temperature. The reaction was monitored by thin-layer chromatography and quenched with saturated NH_4Cl solution, followed by extraction with EtOAc (3×30 mL) in a separatory funnel. The combined organic layer was dried over anhydrous Na_2SO_4, filtered and concentrated in vacuo. The residue was purified by flash column chromatography on silica gel to afford the desired product.

3.2 General procedures for 1,1-carboboration of $ArBCl_2$ with alkynyl selenides

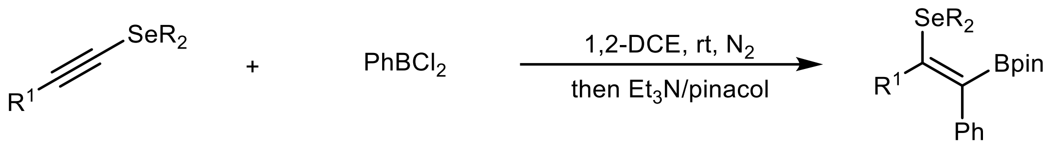

General procedure D: An oven-dried Schlenk tube equipped with a magnetic stir bar was charged with alkynyl selenide (0.2 mmol), anhydrous 1,2-dichloroethane (1 mL) and $PhBCl_2$ (0.4 mmol, 52 μL) under N_2 atmosphere. The mixture was stirred for 12 h at room temperature. Then, the pinacol (1.0 mmol, 118.2 mg) in 1 mL Et_3N was introduced. After continuously stirring for 1 hour, the reaction was quenched with saturated saturated NH_4Cl solution (10 mL), followed by extraction with EtOAc (3×10 mL) in a separatory funnel. The combined organic layer was dried over anhydrous Na_2SO_4, filtered, and concentrated in vacuo. The residue was purified by flash column chromatography on silica gel to afford the desired product.

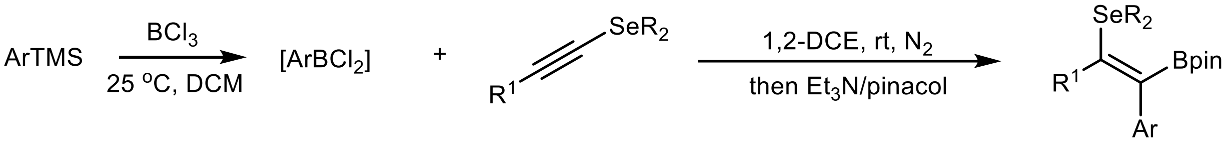

General procedure E: An oven-dried Schlenk tube equipped with a magnetic stir bar was charged with ArTMS (0.4 mmol), anhydrous dichloromethane (1 mL) and BCl_3 (0.8 mmol, 1 M in DCM, 0.8 mL) at 0 °C under N_2 atmosphere. The mixture was stirred at room temperature for 24 h. Subsequently, the excess BCl_3 and solvent was removed under reduced pressure conditions. After refilling the tube with N_2 atmosphere, alkynyl selenide (0.2 mmol) in anhydrous 1,2-dichloroethane (1 mL) was added. The mixture was stirred for 12 h, and then the pinacol (1.0 mmol, 118.2 mg) in 1 mL Et_3N was introduced. After continuously stirring for 1 hour, the reaction was quenched with saturated NH_4Cl solution (10 mL), followed by extraction with EtOAc (3×10 mL) in a separatory funnel. The combined organic layer was dried over anhydrous Na_2SO_4, filtered, and concentrated in vacuo. The residue was purified by flash column chromatography on silica gel to afford the desired product.

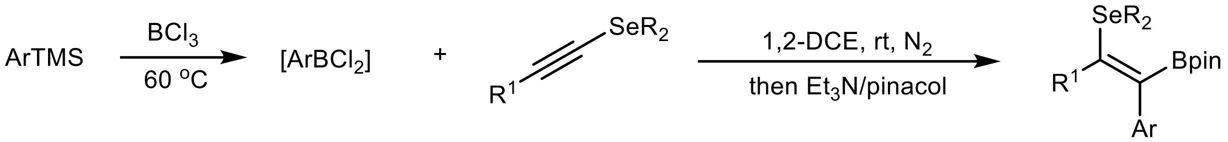

General procedure F: An oven-dried sealed tube equipped with a magnetic stir bar was charged with ArTMS (0.6 mmol), anhydrous dichloromethane (1 mL) and BCl_3 (1.2 mmol, 1 M in DCM, 1.2 mL) at 0 °C under N_2 atmosphere. The reaction vessel was sealed with a Teflon-lined screw cap and stirred at 60 °C for 24 h. Subsequently, the excess BCl_3 and solvent was removed under reduced pressure conditions. After refilling the tube with N_2 atmosphere, alkynyl selenide (0.2 mmol) in anhydrous 1,2-dichloroethane (1 mL) was added. The mixture was stirred for 12 h, and then the pinacol (1.0 mmol, 118.2 mg) in 1 mL Et_3N was introduced. After continuously stirring for 1 hour, the reaction was quenched with saturated saturated NH_4Cl solution (10 mL), followed by extraction with EtOAc (3×10 mL) in a separatory funnel. The combined organic layer was dried over anhydrous Na_2SO_4, filtered,

and concentrated in vacuo. The residue was purified by flash column chromatography on silica gel to afford the desired product.

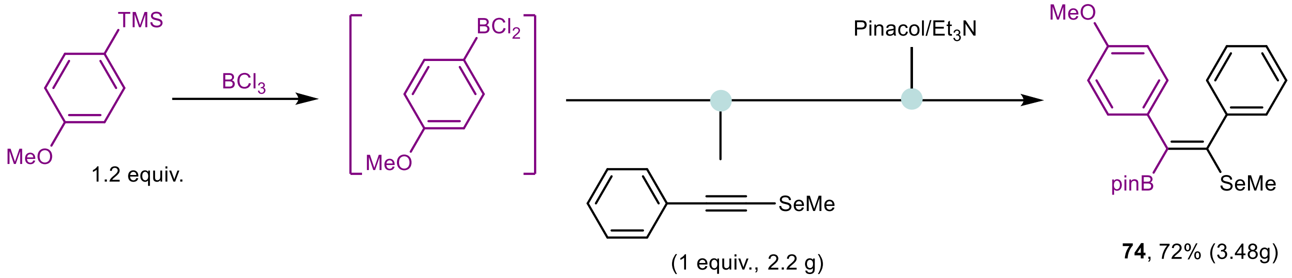

74, 72% (3.48g)

Representative procedure for the scale-up synthesis: An oven-dried Schlenk tube equipped with a magnetic stir bar was charged with (4-methoxyphenyl)trimethylsilane (13.6 mmol, 2.45 g) and BCl_3 (27.2 mmol, 1 M in DCM, 27.2 mL) at 0 °C under N_2 atmosphere. The mixture was stirred at room temperature for 24 h. Subsequently, the excess BCl_3 and solvent was removed under reduced pressure conditions. After refilling the tube with N_2 atmosphere, the methyl(phenylethynyl)selane (11.3 mmol, 2.2 g) in anhydrous 1,2-dichloroethane (6 mL) was added. The mixture was stirred for 12 h, and then the pinacol (56 mmol, 6.6 g) in 6 mL Et_3N was introduced. After continuously stirring for 1 hour, the reaction was quenched with saturated NH_4Cl solution (150 mL), followed by extraction with EtOAc (3×50 mL) in a separatory funnel. The combined organic layer was dried over anhydrous Na_2SO_4, filtered and concentrated in vacuo. The residue was purified by flash column chromatography on silica gel to afford the desired product **74** in 72% yield (3.48 g).

3.3 Representative procedure for downstream derivatizations:

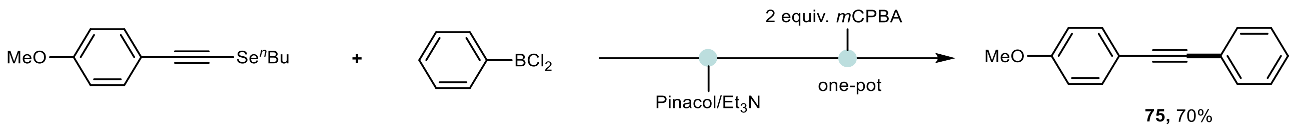

75, 70%

An oven-dried round-bottom flask equipped with a magnetic stir bar was charged with alkynyl selenide (0.2 mmol, 53.4 mg), anhydrous 1,2-dichloroethane (1 mL) and $PhBCl_2$ (0.4 mmol, 52 μL) at room temperature under N_2 atmosphere. The reaction was stirred for 12 h, and then pinacol (1.0 mmol, 118.2 mg) in 1 mL Et_3N was introduced. After continuously stirring for 1 hour, the solvent and Et_3N were removed on a rotary evaporator. To the flask was added *m*-CPBA (0.4 mmol, 69 mg) in 2 mL THF. The mixture was stirred overnight and quenched with saturated NH_4Cl solution (10 mL), followed by extraction with EtOAc (3×10 mL) in a separatory funnel. The combined organic layer was dried over anhydrous Na_2SO_4, filtered and concentrated in vacuo. The residue was purified by flash column chromatography on silica gel to afford the desired product **75** in 70 % yield (29.2 mg).

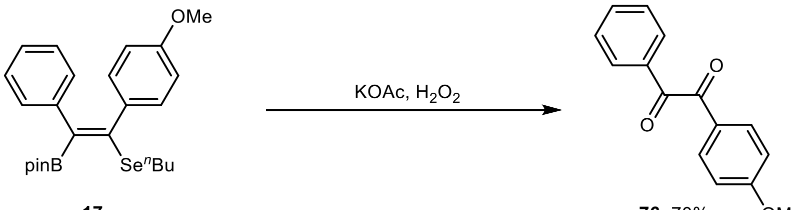

17

76, 70%

A round-bottom flask equipped with a magnetic stir bar was charged with **17** (0.2 mmol, 94.2 mg), THF (2 mL) and 30% H_2O_2 (30 μL) at 0 °C. The reaction was stirred for 20 hours at room temperature, and then quenched with saturated NH_4Cl solution (10 mL), followed by extraction with EtOAc (3×10 mL) in a separatory funnel. The combined organic layer was dried over anhydrous Na_2SO_4, filtered and concentrated in vacuo. The residue was purified by flash column chromatography on silica gel to afford the desired product **76** in 70 % yield (33.6 mg).

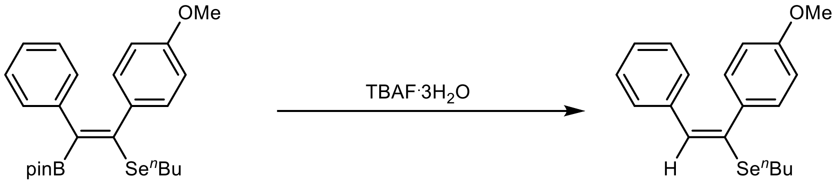

17 **77**, 83%

A round-bottom flask equipped with a magnetic stir bar was charged with **17** (0.1 mmol, 47 mg), THF (1.5 mL) and TBAF·3H_2O (0.3 mmol, 1M, 0.3 mL) at room temperature. The reaction was stirred for 16 hours at 45 °C, and then quenched with saturated aqueous NH_4Cl (10 mL), followed by extraction with EtOAc (3×10 mL). The combined organic layer was dried over anhydrous Na_2SO_4, filtered and concentrated in vacuo. The residue was purified by flash column chromatography on silica gel to afford the desired product **77** in 70 % yield (28.5 mg).

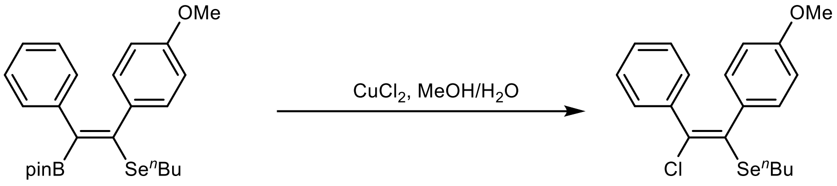

17 **78**, 52%

A round-bottom flask equipped with a magnetic stir bar was charged with **17** (0.1 mmol, 47 mg), $CuCl_2$ (0.5 mmol, 65 mg) and MeOH/H_2O (1:1, 1.3 mL) at room temperature. The reaction was stirred for 24 hours and quenched with saturated aqueous NH_4Cl (10 mL), followed by extraction with EtOAc (3×10 mL). The combined organic layer was dried over anhydrous Na_2SO_4, filtered and concentrated in vacuo. The residue was purified by flash column chromatography on silica gel to afford the desired product **78** in 52 % yield (19.8 mg).

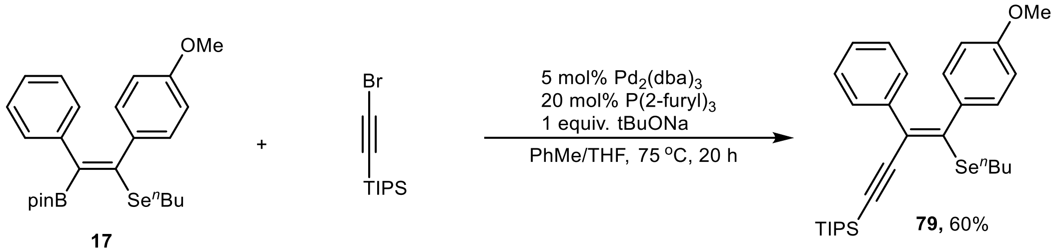

17 **79**, 60%

An oven-dried Schlenk tube equipped with a magnetic stir bar was charged under N_2 atmosphere with **17** (47 mg, 0.1 mmol), $Pd_2(dba)_3$ (4.6 mg, 5 mol%), P(2-furyl)$_3$ (4.6 mg, 20 mol%) and PhMe/THF = 10:1 (1.1 mL). To the mixture was added (bromoethynyl)triisopropylsilane (78 mg, 0.3 mmol) and tBuONa (9.6 mg, 0.1 mmol) in sequence. The resultant suspension was stirred at 75 °C for 20 hours. The reaction was

cooled to room temperature and quenched with brine and extracted with EtOAc for three times in a separatory funnel. The combined organic layer was dried over anhydrous Na_2SO_4, filtered and concentrated on a rotary evaporator. The crude product was purified by flash column chromatography on silica gel to afford the desired product **79** in 60% yield (31.5 mg).

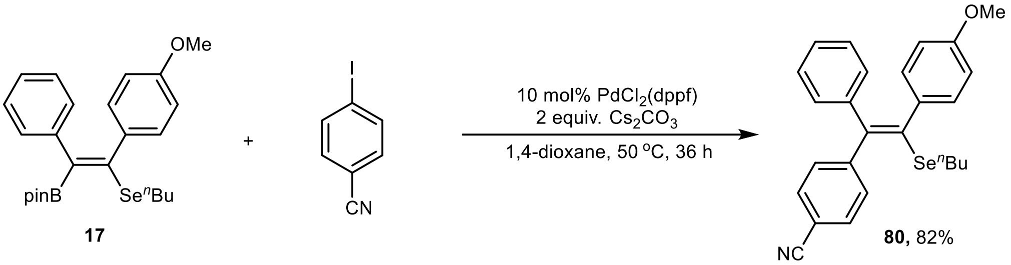

An oven-dried Schlenk tube equipped with a magnetic stir bar was charged under N_2 atmosphere with **17** (47 mg, 0.1 mmol), 4-iodobenzonitrile (69 mg, 0.3 mmol), $PdCl_2$(dppf) (7.3 mg, 10 mol%), Cs_2CO_3 (65 mg,0.2 mmol) and 1,4-dioxane (1 mL). The resultant suspension was stirred at 50 ºC for 36 hours. The reaction was cooled to room temperature and quenched with brine and extracted with EtOAc for three times in a separatory funnel. The combined organic layer was dried over anhydrous Na_2SO_4, filtered and concentrated on a rotary evaporator. The crude product was purified by flash column chromatography on silica gel to afford the desired product **80** in 82% yield (36.6 mg).

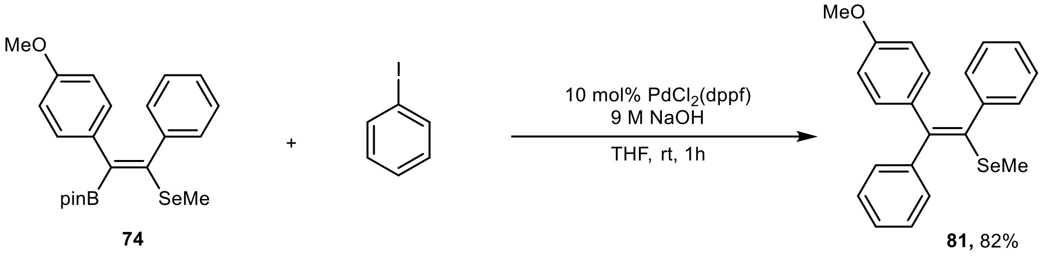

A Schlenk tube equipped with a magnetic stir bar was charged under N_2 atmosphere with the **74** (430 mg, 1 mmol), Pd(dppf)Cl_2 (73 mg, 0.1 mmol) and THF (10 mL). To the mixture was added iodobenzene (225 µL, 2 mmol) and 9 M aqueous KOH solution (0.44 mL) in sequence. The mixture was stirred at room temperature for 1 hour. The reaction was quenched with brine and extracted with EtOAc for three times in a separatory funnel. The combined organic layer was dried over anhydrous Na_2SO_4, filtered and concentrated on a rotary evaporator. The crude product was purified by flash column chromatography on silica gel to afford the desired product **81** in 82% yield (311 mg).

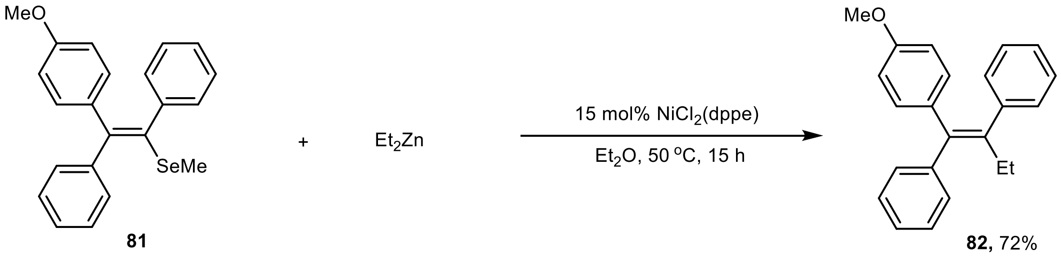

An oven-dried sealed tube equipped with a magnetic stir bar was charged under N_2 atmosphere with the **81** (75.8 mg, 0.2 mmol), Ni(dppe)Cl_2 (16 mg, 15 mol%) and anhydrous Et_2O (2 mL). To the mixture was added Et_2Zn (0.6 mmol, 1 M in ether, 0.6 mL). The reaction

vessel was sealed with a Teflon-lined screw cap and heated at 50 °C. After continuously stirring for 15 hours, the mixture was quenched with H_2O and extracted with EtOAc for three times. The combined organic layer was dried over anhydrous Na_2SO_4, filtered, and concentrated on a rotary evaporator. The crude product was purified by flash column chromatography on silica gel to afford the desired product **82** in 82% yield (45.2 mg).

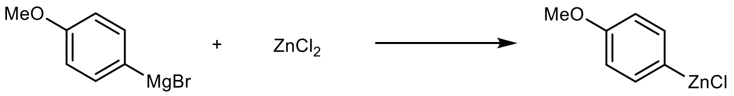

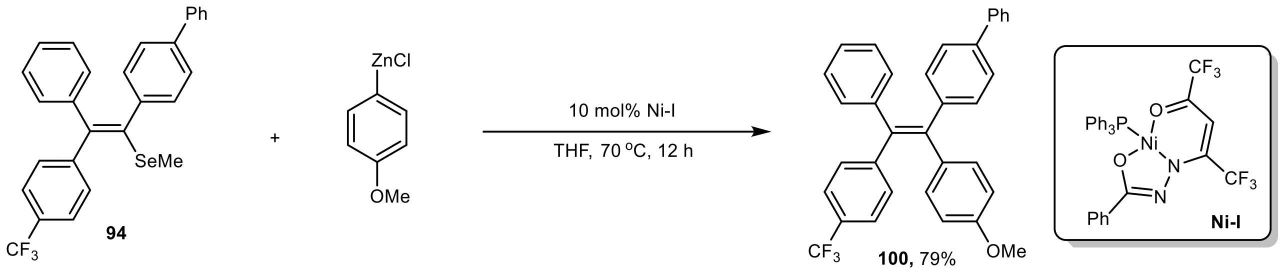

An oven-dried sealed tube equipped with a magnetic stir bar was charged under N_2 atmosphere with the anhydrous $ZnCl_2$ (136.3 mg, 1.0 mmol). The reaction vessel was sealed with a Teflon-lined screw cap and heated at 150 °C for 8 hours. After cooling to room temperature, to the tube was added THF (2 mL). A clear THF solution of $ZnCl_2$ was obtained. To the solution was added (4-methoxyphenyl)magnesium bromide (1.0 mmol), leading to the freshly prepared (4-methoxyphenyl)zinc(II) chloride.

An oven-dried Schlenk tube equipped with a magnetic stir bar was charged under N_2 atmosphere with the **94** (98.6 mg, 0.2 mmol), **Ni-I** (13 mg, 10 mol%) and above freshly prepared zinc reagent. The reaction was stirred at 70 °C for 12 h and quenched with saturated NH_4Cl solution (10 mL), followed by extraction with EtOAc (3×10 mL). The combined organic layer was dried over anhydrous Na_2SO_4, filtered and concentrated on a rotary evaporator. The crude product was purified by flash column chromatography on silica gel to afford the desired product **100** in 79% yield (80 mg).

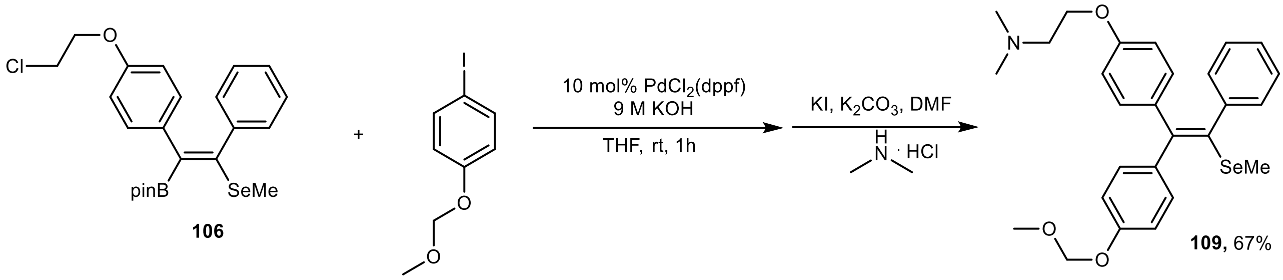

A Schlenk tube equipped with a magnetic stir bar was charged under N_2 atmosphere with the **106** (350 mg, 1 mmol), Pd(dppf)Cl_2 (73 mg, 0.1 mmol) and THF (10 mL). To the mixture was added 1-iodo-4-(methoxymethoxy)benzene (530 mg, 2 mmol) and 9 M aqueous KOH solution (0.44 mL) in sequence. The mixture was stirred at room temperature for 1 hour. The reaction was quenched with brine and extracted with EtOAc for three times in a separatory funnel. The combined organic layer was dried over anhydrous Na_2SO_4, filtered and concentrated on a rotary evaporator. Subsequently, to the residue was added KI (166 mg, 1 mmol), K_2CO_3 (690 mg, 5 mmol), dimethylamine hydrochloride (815 mg, 10 mmol) and DMF

(5 mL). The mixture was stirred at 100 °C for 12 hours. Then, the reaction was quenched with brine and extracted with EtOAc (3×30 mL). The combined organic layer was dried over anhydrous Na_2SO_4, filtered and concentrated on a rotary evaporator. The crude product was purified by flash column chromatography on silica gel to afford the desired product **109** in 67% yield (332 mg).

Cell cultures

The human breast cancer MCF-7 cells were provided by Cell Bank, Chinese Academy of Sciences (SCSP-531). MCF-7 cells were cultured in Dulbecco's modified Eagle medium (DMEM; Gibco, Grand Island, NY, USA) supplemented with 10% fetal bovine serum (FBS; Gibco, Grand Island, NY, USA), 1% penicillin/streptomycin in a humidified atmosphere containing 5% CO_2 at 37℃.

MTT assays

MCF-7 cells were plated at a density of 7×10^3 cells per well in 96-well plates. After 24h, cells were treated with tamoxifen (TAM) or Compound 109-119 at various concentrations for 48h. After treatment, 20 μL 0.5% MTT (Sigma-Aldrich, St. Louis, MO) solution was added into the medium for another 4 h. The supernatant was discarded, and formazan was resolved in 100 μL DMSO (Solarbio, China). The absorbance was measured at 570 nm using a Spectra Max i3x reader (Molecular Devices, Sunnyvale, CA, USA).

In vivo experiments

Female NU/NU nude mice (4 weeks old) were obtained from Vital River Laboratory Animal Technology Co., Ltd. (Beijing, China). All animal experiments were performed in accordance with the institutional ethical guidelines on animal care of Nanjing University of Chinese Medicine (Approval No. 202406A093). The mice were housed under standard conditions at room temperature (22 °C) with a 12-hour light/dark cycle and acclimated to the environment for one week prior to the experiments. All experiments were performed in a blinded manner.

MCF-7 cells (3.5×10^7 cells) were subcutaneously implanted in the armpits of female NU/NU nude mice. Tumor volume (TV) was measured by a vernier caliper and calculated using the following formula: TV (mm^3) = $D/2 \times d^2$ (D: longest diameter; d: shortest diameters). Once tumor size reached 80-100 mm^3, the mice were randomized into five groups (n = 7/group): Control group (mice treated with vehicle), TAM group (mice treated with 13.4 μmol/kg TAM), 109-L group (mice treated with 6.6 μmol/kg compound 109), 109-M group (mice treated with 13.3 μmol/kg compound 109), and 109-H group (mice treated with 26.6 μmol/kg compound 109). TAM and 109 were administered intragastrically every other day for 28 days. General tissue morphology was visualized using hematoxylin and eosin (H&E) staining, and captured using Leica DM1000 Upright microscope.

Statistical analysis

All data were replicated three times and expressed as mean ± SEM and analyzed by GraphPad Prism version 7.0 (RRID: SCR_002798). Two-way ANOVA followed by Tukey's multiple comparison test were used for at least three group's comparisons. Two-tailed unpaired t test was used for two-group comparison. p values were displayed in each Figure legend.

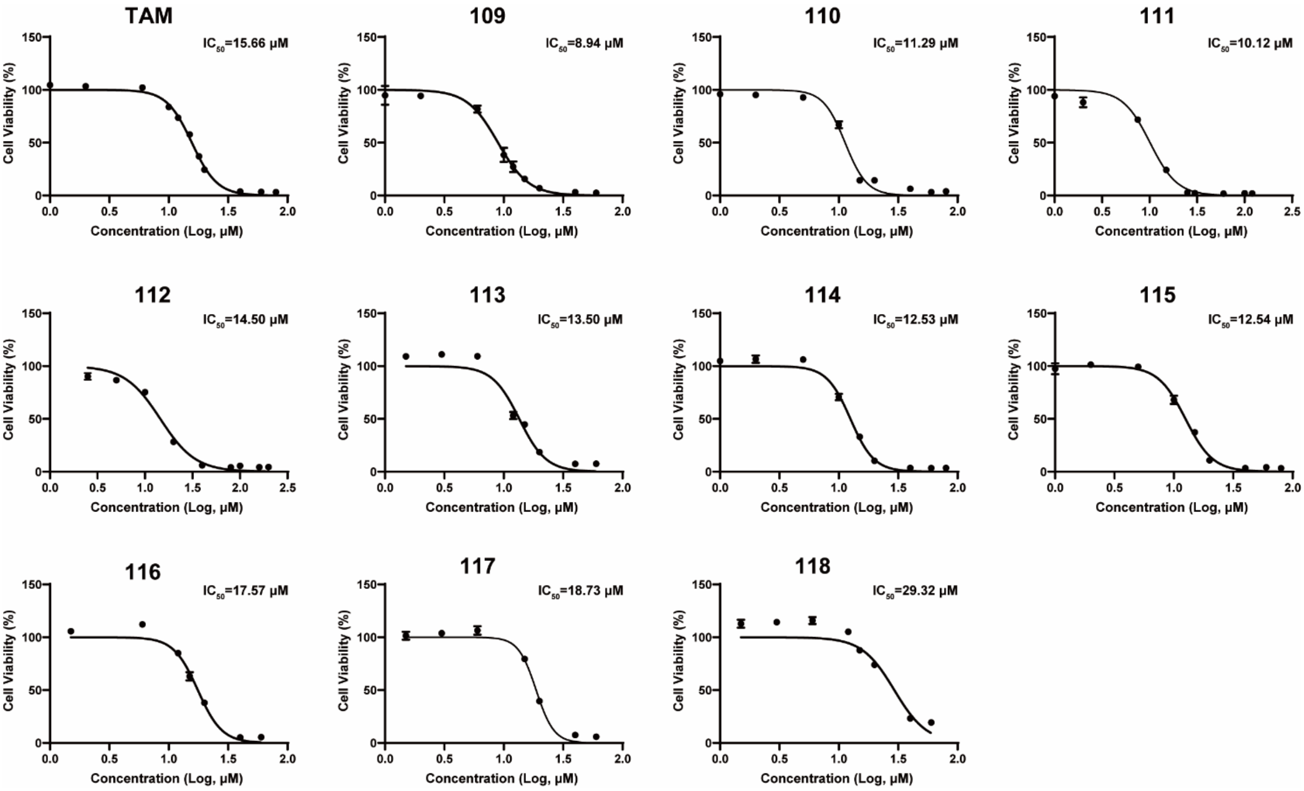

Figure S1. The cytotoxicity of compounds to MCF-7 cells. The cell viabilities of cells were detected by MTT assay. Data are n = 5 (TAM and 109) or n = 3 (other compounds) technical replicates, three independent experiments. All values were presented as mean ± SEM.

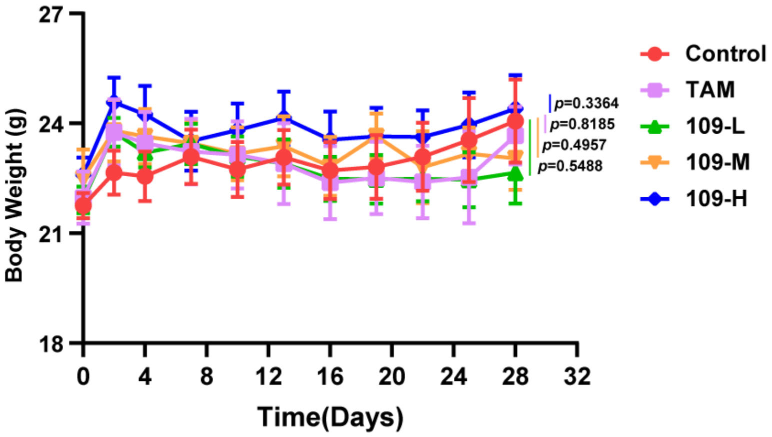

Figure S2. Changes in body weight of the nude mice. All values were presented as mean ± SEM. Significance was determined using two-tailed unpaired t test. There was no significant difference between the groups (con *vs*. TAM, p = 0.8185; con *vs*. 109-L, p = 0.5488; con *vs*. 109-M, p = 0.4957; con *vs*. 109-H, p = 0.3364). Data are n = 6 (con group and TAM group) or n = 7 (109-L, 109-M and 109-H group) biological replicates, one independent experiment. **TAM** = 13.4 μmol/kg; **109-L** = 6.6 μmol/kg; **109-M** = 13.3 μmol/kg; **109-H** = 26.6 μmol/kg.

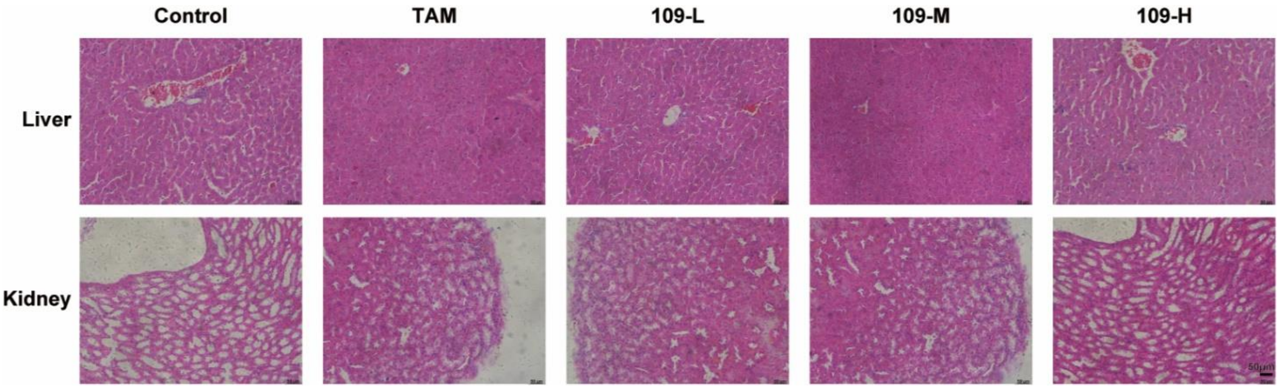

Figure S3. Histopathological analysis using HE staining. Scale bar：50 μm. Data are *n* = 6 (con group and TAM group) or *n* = 7 (109-L, 109-M and 109-H group) biological replicates, one independent experiment.

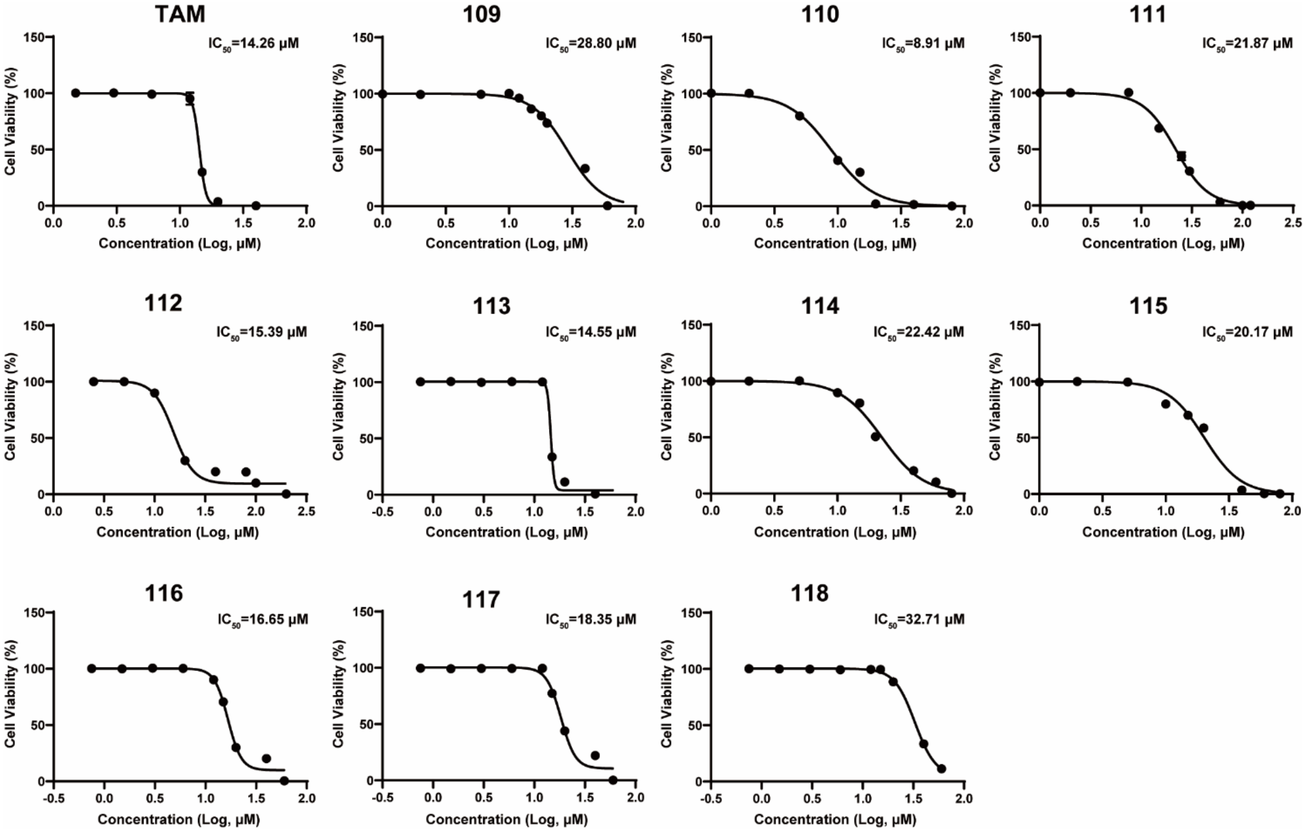

Figure S4. The cytotoxicity of compounds to normal cell (MCF-10A). The cell viabilities of cells were detected by MTT assay. Data are *n* = 5 technical replicates, three independent experiments. All values were presented as mean ± SEM.

Optical and Photophysical Properties

All properties were examined in a mixture solution of THF/H_2O and in quartz cuvettes providing a layer thickness of 1 cm. UV-vis absorption spectra were recorded on an Agilent Cary 60 spectrophotometer. Fluorescence spectra were measured on a HITACHI F-4700 device. Photographs were taken under UV light irradiation (λ = 365 nm) using a Sony A7M4 digital camera.

Table S4.

	λ_{abs} [nm](log ε)	λ_{em} [nm](intensity)	Stokes shift [nm]
**100**	276 (0.93)	553 (9175)	277
**101**	288 (0.73)	560 (9999)	272
**102**	257 (0.92)	515 (2292)	258
**103**	258 (0.96)	516 (2497)	258
**104**	264 (0.88)	529 (4140)	265
**105**	275 (0.95)	550 (8555)	275

4. Computational Studies

All the DFT calculations were performed with Gaussian 09B program[11]. Geometry optimizations were conducted by using the B3LYP functional[12, 13] and Grimme's dispersion correction with Becke-Johnson damping[14]. The def2-TZVP basis set[15] were applied to all atoms. To account for the solvation effects of dichloroethane, the polarizable-continuum model (PCM)[16] was utilized. To ensure all stationary points as local minima (zero imaginary frequencies) or transition states (one imaginary frequency), vibrational frequency analyses were performed at the same level of theory, and the thermodynamic corrections were derived accordingly. Validation of the transition states with the corresponding intermediates was confirmed by intrinsic reaction coordinate (IRC)[17, 18] calculations. To get more accurate energies, single point energies were performed at the M06-2X/def2-TZVPP level[19-22] with the solvent model density (SMD) method[23] of 1,2-dichloroethane. All reported Gibbs free energy values are concentration corrected to correspond to the concentrations of compounds and intermediates in the reactions. All geometric figures were plotted using CYLview[24]. All the condensed dual descriptors (CDD) and the corresponding ISOsufaces were calculated and plotted using Multiwfn[25] and VMD[26].

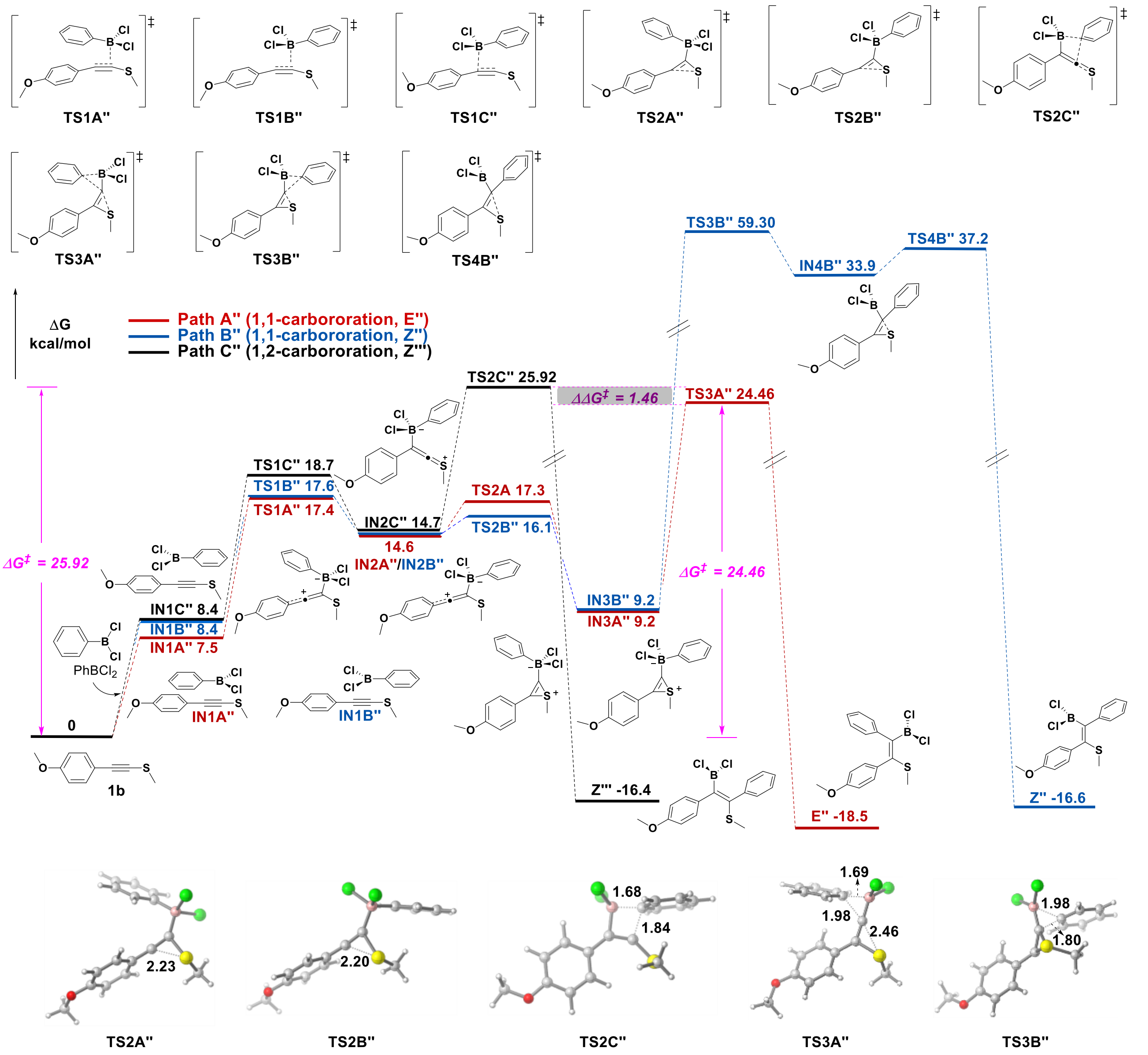

Figure S5. Free-energy profiles of reaction pathways for carboboration of acetylenic thioethers (**1b**) computed at the B3-LYP(D3BJ)/def2-TZVP/PCM (1,2-DCE) //M06-2X/def2-TZVPP/SMD (1,2-DCE) level of theory. Reaction pathways of the electrophilic 1,1-carboboration of alkynyl sulfide to produce **E”** (Path A”, in red) and **Z”** (Path B”, in blue) products and 1,2-carboronation to produce **Z’”** (Path C’’, in black) products.

Since the reaction is thermodynamically controlled, we have conducted distortion-interaction analysis for transition states **TS3A** and **TS3B,** which lead to the formation of the final *E*- and *Z*- products, respectively. Our analysis reveals that the primary factor contributing to the difference in the electronic energy barriers between **TS3A** and **TS3B** is the variation in electronic interactions (Supplementary **Table S5**). Additionally, the highest occupied molecular orbital (HOMO) of **TS3A** shows an extended electronic delocalization from the Se atom in fragment 1 to the C atom bonded to the B atom in fragment 2. In contrast, HOMO of **TS3B** displays a notable repulsion interaction between these atoms (Supplementary **Figure S6**). Based on these results, it is concluded that the Se atom plays an important role in favoring the *E*-selectivity of the 1,1-caboboration process.

Table S5. Distortion-interaction analysis of **TS3A** and **TS3B**. Energies are in kcal mol^{-1}.

Transition state	Fragment	$\Delta E^{\ddagger}$	$\Delta E^{\ddagger}_{dis}$	$\Delta E^{\ddagger}_{int}$
**TS3A**	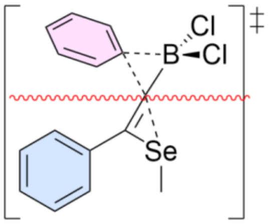<br>**Fragment 1**	7.7	199.2	-191.5
**TS3B**	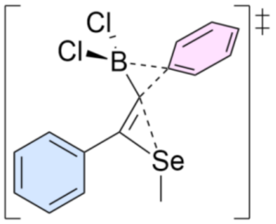<br>**Fragment 2**	15.4	196.0	-180.6

Distortion-interaction analysis[27, 28] ($\Delta E^{\ddagger} = \Delta E^{\ddagger}_{dis} + \Delta E^{\ddagger}_{int}$) of **TS3A** and **TS3B** shows comparable distortion energies ($\Delta E^{\ddagger}_{dis}$) for both transition states (199.2 vs. 196.0 kcal mol^{-1}). However, the interaction energy term ($\Delta E^{\ddagger}_{int}$) strongly favors the generation of *E*-product (-191.5 vs. -180.6 kcal mol^{-1}).

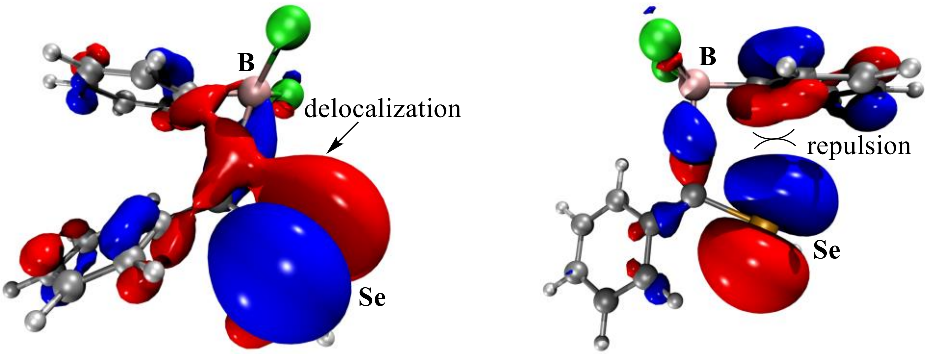

TS3A **TS3B**

Figure S6. HOMOs of **TS3A** and **TS3B** (isosurface = 0.03 a.u.).

5. Characterization

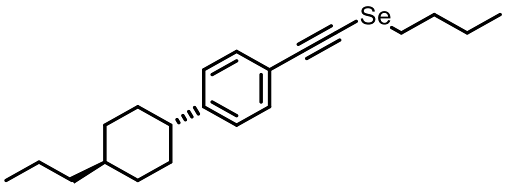

butyl((4-((1s,4r)-4-propylcyclohexyl)phenyl)ethynyl)selane
Prepared according to general procedure A.
Yield 84%, yellow oil;
^{1}H NMR (500 MHz, $CDCl_3$) δ = 7.35 (d, *J* = 8.3 Hz, 2 H), 7.14 (d, *J* = 8.3 Hz, 2 H), 2.87 (t, *J* = 7.5 Hz, 2 H), 2.48-2.42 (m, 1 H), 1.89-1.83 (m, 6 H), 1.53-1.30 (m, 7 H), 1.24-1.18 (m, 2 H), 1.08-1.00 (m, 2 H), 0.96 (t, *J* = 7.5 Hz, 3 H), 0.90 (t, *J* = 7.5 Hz, 3 H) ppm;
^{13}C NMR (126 MHz, $CDCl_3$) δ = 148.2, 131.6, 126.8, 121.0, 99.5, 69.4, 44.6, 39.7, 37.0, 34.2, 33.5, 32.2, 29.4, 22.5, 20.1, 14.4, 13.6 ppm;
HRMS (ESI): Exact mass calculated for $[C_{21}H_{30}Se+H]^+$: 363.1585, mass found: 363.1578.

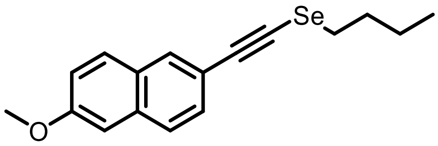

butyl((6-methoxynaphthalen-2-yl)ethynyl)selane
Prepared according to general procedure A.
Yield 74%, yellow semi-solid;
^{1}H NMR (500 MHz, $CDCl_3$) δ = 7.90-7.83 (m, 1 H), 7.69-7.62 (m, 2 H), 7.46-7.41 (m, 1 H), 7.17-7.12 (m, 1 H), 7.10-7.06 (m, 1 H), 3.92 (s, 3 H), 2.91 (t, *J* = 7.4 Hz, 2 H), 1.92-1.84 (m, 2 H), 1.53-1.46 (m, 2 H), 0.97 (t, *J* = 7.4 Hz, 3 H) ppm;
^{13}C NMR (126 MHz, $CDCl_3$) δ = 158.3, 134.0, 131.2, 129.3, 129.1, 128.4, 126.7, 119.4, 118.7, 105.8, 99.9, 69.9, 55.4, 32.3, 29.4, 22.5, 13.6 ppm;
HRMS (ESI): Exact mass calculated for $[C_{17}H_{18}OSe+H]^+$: 319.0596, mass found: 319.0596.

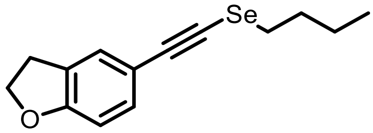

5-((butylselanyl)ethynyl)-2,3-dihydrobenzofuran
Prepared according to general procedure B.
Yield 79%, yellow oil;
^{1}H NMR (500 MHz, $CDCl_3$) δ = 7.26 (s, 1 H), 7.21 (d, *J* = 8.3 Hz, 1 H), 6.70 (d, *J* = 8.3 Hz, 1 H), 4.57 (t, *J* = 8.5 Hz, 2 H), 3.17 (t, *J* = 8.5 Hz, 2 H), 2.85 (t, *J* = 7.5 Hz, 2 H), 1.87-1.80 (m, 2 H), 1.50-1.44 (m, 2 H), 0.95 (t, *J* = 7.5 Hz, 3 H) ppm;
^{13}C NMR (126 MHz, $CDCl_3$) δ = 160.3, 132.4, 128.5, 127.2, 115.6, 109.3, 99.5, 71.5, 67.6, 32.2, 29.4, 29.3, 22.5, 13.6 ppm;
HRMS (ESI): Exact mass calculated for $[C_{14}H_{16}OSe+H]^+$: 281.0439, mass found: 281.0439.

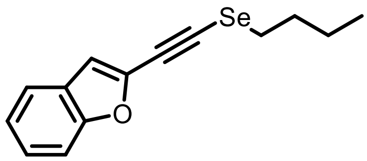

2-((butylselanyl)ethynyl)benzofuran
Prepared according to general procedure B.
Yield 81%, yellow oil;
^{1}H NMR (500 MHz, $CDCl_3$) δ = 7.59-7.55 (m, 1 H), 7.48-7.44 (m, 1 H), 7.37-7.33 (m, 1 H), 7.29-7.23 (m, 1 H), 6.95 (s, 1 H), 2.96 (t, *J* = 7.5 Hz, 2 H), 1.92-1.86 (m, 2 H), 1.54-1.48 (m, 2 H), 0.99 (t, *J* = 7.5 Hz, 3 H) ppm;
^{13}C NMR (126 MHz, $CDCl_3$) δ = 154.7, 138.8, 127.6, 125.7, 123.2, 121.2, 112.0, 111.2, 89.6, 79.5, 32.3, 29.9, 22.5, 13.5 ppm;
HRMS (EI): Exact mass calculated for $[C_{14}H_{14}OSe]^+$: 278.0204, mass found: 278.0204.

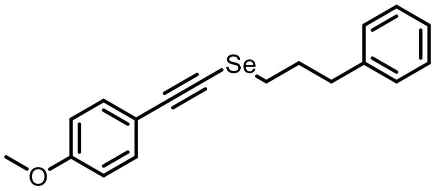

((4-methoxyphenyl)ethynyl)(3-phenylpropyl)selane
Prepared according to general procedure A.
Yield 87%, yellow oil;
^{1}H NMR (500 MHz, $CDCl_3$) δ = 7.40-7.33 (m, 2 H), 7.32-7.23 (m, 2 H), 7.22-7.15 (m, 3 H), 6.85-6.74 (m, 2 H), 3.77 (s, 3 H), 2.82 (t, *J* = 7.5 Hz, 2 H), 2.77 (t, *J* = 7.5 Hz, 2 H), 2.18 (quint, *J* = 7.5 Hz, 2 H) ppm;
^{13}C NMR (126 MHz, $CDCl_3$) δ = 159.6, 141.2, 133.4, 128.6, 128.5, 126.1, 115.8, 114.0, 99.5, 68.2, 55.3, 35.3, 31.6, 28.7 ppm;
HRMS (ESI): Exact mass calculated for $[C_{18}H_{18}OSe+H]^+$: 331.0596, mass found: 331.0590.

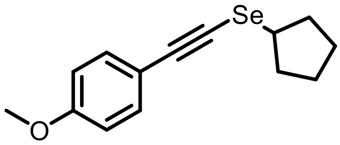

cyclopentyl((4-methoxyphenyl)ethynyl)selane
Prepared according to general procedure A.
Yield 70%, yellow oil;
^{1}H NMR (500 MHz, $CDCl_3$) δ = 7.38 (d, *J* = 8.5 Hz, 2 H), 6.82 (d, *J* = 8.5 Hz, 2 H), 3.80 (s, 3 H), 3.66-3.60 (m, 1 H), 2.12-2.05 (m, 2 H), 1.91-1.78 (m, 4 H), 1.66-1.59 (m, 2 H) ppm;
^{13}C NMR (126 MHz, $CDCl_3$) δ = 159.5, 133.3, 116.0, 113.9, 99.6, 69.2, 55.3, 43.5, 33.8, 25.0 ppm;
HRMS (ESI): Exact mass calculated for $[C_{14}H_{16}OSe+H]^+$: 281.0439, mass found: 281.0438.

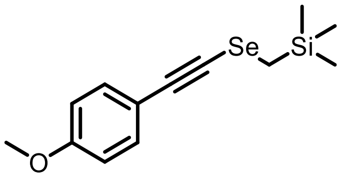

((((4-methoxyphenyl)ethynyl)selanyl)methyl)trimethylsilane
Prepared according to general procedure A.
Yield 90%, yellow oil;
^{1}H NMR (500 MHz, $CDCl_3$) δ = 7.35 (d, *J* = 9.0 Hz, 2 H), 6.82 (d, *J* = 9.0 Hz, 2 H), 3.80 (s, 3 H), 2.10 (s, 2 H), 0.17 (s, 9 H) ppm;
^{13}C NMR (126 MHz, $CDCl_3$) δ = 159.5, 133.2, 115.9, 113.9, 97.9, 70.5, 55.3, 14.1, -1.3 ppm;
HRMS (ESI): Exact mass calculated for $[C_{13}H_{18}OSeSi+H]^+$: 299.0365, mass found: 299.0362.

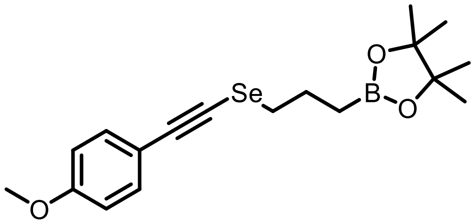

2-(3-(((4-methoxyphenyl)ethynyl)selanyl)propyl)-4,4,5,5-tetramethyl-1,3,2-dioxaborolane
Prepared according to general procedure A.
Yield 85%, yellow oil;
^{1}H NMR (500 MHz, $CDCl_3$) δ = 7.37 (d, *J* = 8.3 Hz, 2 H), 6.81 (d, *J* = 8.3 Hz, 2 H), 3.80 (s, 3 H), 2.87 (t, *J* = 7.5 Hz, 2 H), 1.97 (quint, *J* = 7.5 Hz, 2 H), 1.24 (s, 12 H), 0.94 (t, *J* = 7.5 Hz, 2 H) ppm;
^{13}C NMR (126 MHz, $CDCl_3$) δ = 159.5, 133.3, 116.0, 113.8, 98.9, 83.1, 68.6, 55.3, 32.2, 24.9, 24.8 ppm;
HRMS (ESI): Exact mass calculated for $[C_{18}H_{25}BO_3Se+H]^+$: 381.1135, mass found: 381.1129.

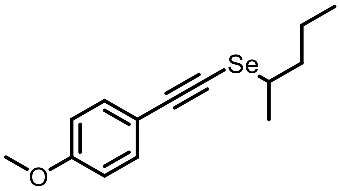

((4-methoxyphenyl)ethynyl)(pentan-2-yl)selane
Prepared according to general procedure A.
Yield 81%, yellow oil;
^{1}H NMR (500 MHz, $CDCl_3$) δ = 7.38 (d, *J* = 9.0 Hz, 2 H), 6.83 (d, *J* = 9.0 Hz, 2 H), 3.81 (s, 3 H), 3.34-3.24 (m, 1 H), 1.83-1.77 (m, 1 H), 1.67-1.62 (m, 1 H), 1.60-1.56 (m, 3 H), 1.53-1.44 (m, 2 H), 0.95 (t, *J* = 7.3 Hz, 3 H) ppm;
^{13}C NMR (126 MHz, $CDCl_3$) δ = 159.5, 133.2, 116.1, 113.9, 100.8, 68.1, 55.3, 41.3, 39.6, 22.4, 21.2, 13.8 ppm;
HRMS (ESI): Exact mass calculated for $[C_{14}H_{18}OSe+H]^+$: 283.0596, mass found: 283.0594.

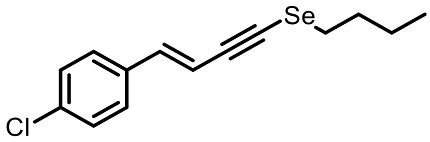

(*E*)-butyl(4-(4-chlorophenyl)but-3-en-1-yn-1-yl)selane

Prepared according to general procedure A.
Yield 80%, yellow oil;
^{1}H NMR (500 MHz, $CDCl_3$) δ = 7.28 (s, 4 H), 6.83 (d, *J* = 16.0 Hz, 1 H), 6.23 (d, *J* = 16.0 Hz, 1 H), 2.85 (t, *J* = 7.5 Hz, 2 H), 1.85-1.79 (m, 2 H), 1.50-1.43 (m, 2 H), 0.96 (t, *J* = 7.5 Hz, 3 H) ppm;
^{13}C NMR (126 MHz, $CDCl_3$) δ = 139.0, 134.8, 134.1, 128.9, 127.3, 109.1, 98.7, 74.0, 32.3, 29.6, 22.5, 13.6 ppm;
HRMS (ESI): Exact mass calculated for $[C_{14}H_{15}ClSe+H]^+$: 299.0100, mass found: 299.0101.

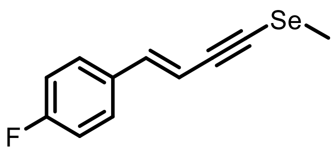

(*E*)-(4-(4-fluorophenyl)but-3-en-1-yn-1-yl)(methyl)selane
Prepared according to general procedure C.
Yield 82%, yellow oil;
^{1}H NMR (500 MHz, $CDCl_3$) δ = 7.35-7.32 (m, 2 H), 7.03-6.99 (m, 2 H), 6.86 (d, *J* = 16.0 Hz, 1 H), 6.16 (d, *J* = 16.0 Hz, 1 H), 2.35 (s, 3 H) ppm;
^{13}C NMR (126 MHz, $CDCl_3$) δ = 162.9 (d, *J* = 249.2 Hz), 139.5, 132.4 (d, *J* = 3.4 Hz), 127.8 (d, *J* = 8.1 Hz), 115.8 (d, *J* = 20.5 Hz), 108.0 (d, *J* = 2.4 Hz), 97.70, 73.76, 9.92 ppm;
HRMS (ESI): Exact mass calculated for $[C_{11}H_9FSe+H]^+$: 240.9926, mass found: 240.9925.

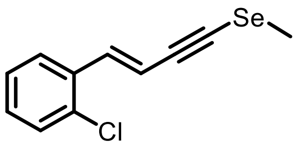

(*E*)-(4-(2-chlorophenyl)but-3-en-1-yn-1-yl)(methyl)selane
Prepared according to general procedure C.
Yield 81%, yellow oil;
^{1}H NMR (500 MHz, $CDCl_3$) δ = 7.52-7.48 (m, 1 H), 7.38-7.34 (m, 1 H), 7.29 (d, *J* = 16.0 Hz, 1 H), 7.24-7.18 (m, 2 H), 6.24 (d, *J* = 16.0 Hz, 1 H), 2.37 (s, 3 H) ppm;
^{13}C NMR (126 MHz, $CDCl_3$) δ = 136.3, 134.3, 133.1, 129.9, 129.4, 126.9, 125.9, 110.8, 97.9, 75.2, 9.9 ppm;
HRMS (ESI): Exact mass calculated for $[C_{11}H_9ClSe+H]^+$: 256.9631, mass found: 256.9627.

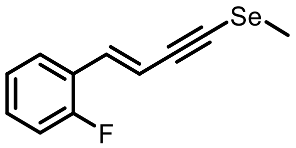

(*E*)-(4-(2-fluorophenyl)but-3-en-1-yn-1-yl)(methyl)selane
Prepared according to general procedure C.
Yield 82%, yellow oil;
^{1}H NMR (500 MHz, $CDCl_3$) δ = 7.43-7.39 (m, 1 H), 7.27-7.19 (m, 1 H), 7.11-7.00 (m, 3 H), 6.34 (d, *J* = 16.5 Hz, 1 H), 2.36 (s, 3 H) ppm;

^{13}**C NMR** (126 MHz, $CDCl_3$) δ = 160.2 (d, *J* = 252.0 Hz), 133.1 (d, *J* = 2.7 Hz), 129.7 (d, *J* = 8.6 Hz), 127.0 (d, *J* = 3.5 Hz), 124.2 (d, *J* = 3.5 Hz), 124.1, 115.9(d, *J* = 22.7 Hz) 110.8 (d, *J*= 6.7 Hz), 98.1, 74.8, 9.9 ppm;
HRMS (ESI): Exact mass calculated for $[C_{11}H_9FSe+H]^+$: 240.9926, mass found: 240.9927.

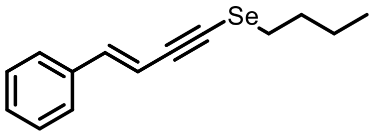

(*E*)-butyl(4-phenylbut-3-en-1-yn-1-yl)selane
Prepared according to general procedure A.
Yield 70%, yellow oil;
^{1}**H NMR** (500 MHz, $CDCl_3$) δ = 7.39-7.35 (m, 2 H), 7.34-7.29 (m, 2 H), 7.29-7.25 (m, 1 H), 6.94-6.86 (m, 1 H), 6.29-6.24 (m, 1 H), 2.85 (t, *J* = 7.5 Hz, 2 H), 1.85-1.80 (m, 2 H), 1.49-1.43 (m, 2 H), 0.99-0.92 (m, 3 H) ppm;
^{13}**C NMR** (126 MHz, $CDCl_3$) δ = 140.5, 136.3, 128.7, 128.5, 126.2, 108.4, 99.0, 73.1, 32.3, 29.5, 22.5, 13.6 ppm;
HRMS (ESI): Exact mass calculated for $[C_{14}H_{16}Se+H]^+$: 265.0490, mass found: 265.0492.

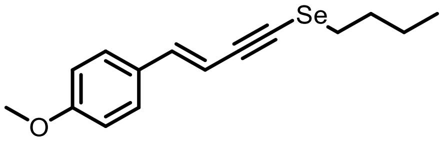

(*E*)-butyl(4-(4-methoxyphenyl)but-3-en-1-yn-1-yl)selane
Prepared according to general procedure A.
Yield 73%, yellow oil;
^{1}**H NMR** (500 MHz, $CDCl_3$) δ = 7.31 (d, *J* = 8.5 Hz, 2 H), 6.88-6.84 (m, 3 H), 6.12 (d, *J* = 16.0 Hz, 1 H), 3.81 (s, 3 H), 2.84 (t, *J* = 7.5 Hz, 2 H), 1.85-1.77 (m, 2 H), 1.49-1.44 (m, 2 H), 0.96 (t, *J* = 7.5 Hz, 3 H) ppm;
^{13}**C NMR** (126 MHz, $CDCl_3$) δ = 160.0, 140.4, 129.1, 127.5, 114.2, 106.1, 99.2, 71.8, 55.3, 32.3, 29.5, 22.5, 13.5 ppm;
HRMS (ESI): Exact mass calculated for $[C_{15}H_{18}OSe+H]^+$: 295.0596, mass found: 295.0595.

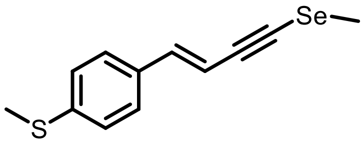

(*E*)-methyl(4-(4-(methylselanyl)but-1-en-3-yn-1-yl)phenyl)sulfane
Prepared according to general procedure C.
Yield 57%, yellow solid, mp: 60-61 ^{o}C;
^{1}**H NMR** (500 MHz, $CDCl_3$) δ = 7.28 (d, *J* = 8.0 Hz, 2 H), 7.18 (d, *J* = 8.0 Hz, 2 H), 6.85 (d, *J* = 16.0 Hz, 1 H), 6.19 (d, *J* = 16.0 Hz, 1 H), 2.48 (s, 3H), 2.35 (s, 3 H) ppm;
^{13}**C NMR** (126 MHz, $CDCl_3$) δ = 140.2, 139.3, 133.0, 126.5, 126.4, 107.4, 98.0, 73.7, 15.6, 9.9 ppm;

HRMS (ESI): Exact mass calculated for $[C_{12}H_{12}SSe+H]^+$: 268.9898, mass found: 268.9897.

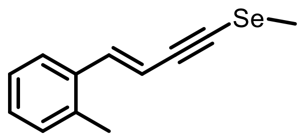

(*E*)-methyl(4-(o-tolyl)but-3-en-1-yn-1-yl)selane
Prepared according to general procedure C.
Yield 63%, yellow oil;
^{1}H NMR (500 MHz, $CDCl_3$) δ = 7.45-7.42 (m, 1 H), 7.19-7.14 (m, 4 H), 6.16 (d, *J* = 16.0 Hz, 1 H), 2.36 (s, 3 H), 2.36 (s, 3 H) ppm;
^{13}C NMR (126 MHz, $CDCl_3$) δ = 138.6, 135.8, 135.1, 130.5, 128.5, 126.2, 124.8, 109.2, 98.3, 73.3, 19.8, 9.9 ppm;
HRMS (ESI): Exact mass calculated for $[C_{12}H_{12}Se+H]^+$: 237.0177, mass found: 237.0175.

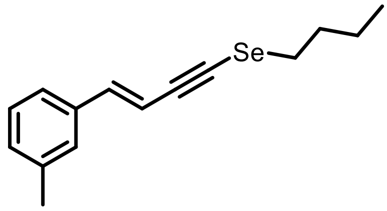

(*E*)-butyl(4-(m-tolyl)but-3-en-1-yn-1-yl)selane
Prepared according to general procedure A.
Yield 73%, yellow oil;
^{1}H NMR (500 MHz, $CDCl_3$) δ = 7.22-7.16 (m, 3 H), 7.11-7.05 (m, 1 H), 6.87 (d, *J* = 16.0 Hz, 1 H), 6.25 (d, *J* = 16.0 Hz, 1 H), 2.85 (t, *J* = 7.5 Hz, 2 H), 2.34 (s, 3 H), 1.86-1.79 (m, 2 H), 1.50-1.43 (m, 2 H), 0.96 (t, *J* = 7.5 Hz, 3 H) ppm;
^{13}C NMR (126 MHz, $CDCl_3$) δ = 140.7, 138.3, 136.2, 129.3, 128.6, 126.9, 123.3, 108.2, 99.1, 72.9, 32.3, 29.5, 22.5, 21.4, 13.6 ppm;
HRMS (ESI): Exact mass calculated for $[C_{15}H_{18}Se+H]^+$: 279.0646, mass found: 279.0644.

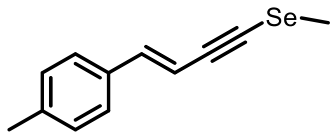

(*E*)-methyl(4-(p-tolyl)but-3-en-1-yn-1-yl)selane
Prepared according to general procedure C.
Yield 82%, yellow oil;
^{1}H NMR (500 MHz, $CDCl_3$) δ = 7.26 (d, *J* = 7.5 Hz, 2 H), 7.12 (d, *J* = 8.0 Hz, 2 H), 6.89 (d, *J* = 16.0 Hz, 1 H), 6.19 (d, *J* = 16.0 Hz, 1 H), 2.35 (s, 3 H), 2.34 (s, 3 H) ppm;
^{13}C NMR (126 MHz, $CDCl_3$) δ = 140.9, 138.7, 133.5, 129.4, 126.1, 107.1, 98.1, 73.1, 21.3, 9.9 ppm;
HRMS (ESI): Exact mass calculated for $[C_{12}H_{12}Se+H]^+$: 237.0177, mass found: 237.0176.

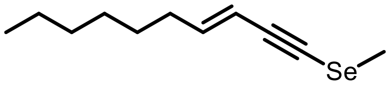

(*E*)-dec-3-en-1-yn-1-yl(methyl)selane
Prepared according to general procedure C.
Yield 65%, yellow oil;
^{1}H NMR (500 MHz, $CDCl_3$) δ = 6.22-6.04 (m, 1 H), 5.65-5.45 (m, 1 H), 2.30 (s, 3 H), 2.14-2.06 (m, 2 H), 1.40-1.34 (m, 2 H), 1.31-1.23 (m, 6 H), 0.88 (t, *J* = 7.5 Hz, 3 H) ppm;
^{13}C NMR (126 MHz, $CDCl_3$) δ = 145.2, 109.7, 97.3, 68.8, 33.0, 31.7, 28.8, 28.7, 22.6, 14.1, 9.7 ppm;
HRMS (ESI): Exact mass calculated for $[C_{11}H_{18}Se+H]^+$: 231.0646, mass found: 231.0648.

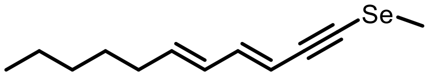

Methyl((3*E*,5*E*)-undeca-3,5-dien-1-yn-1-yl)selane
Prepared according to general procedure C.
Yield 67%, yellow oil;
^{1}H NMR (500 MHz, $CDCl_3$) δ = 6.53 (dd, *J* = 15.5, 11.0 Hz, 1 H), 6.06 (dd, *J* = 15.0, 11.0 Hz, 1 H), 5.83-5.75 (m, 1H), 5.57 (d, *J* = 15.5 Hz, 1 H), 2.31 (s, 3 H), 2.09 (q, *J* = 7.5 Hz, 2 H), 1.42-1.37 (m, 2 H), 1.31-1.25 (m, 4 H), 0.88 (t, *J* = 6.5 Hz, 3 H) ppm;
^{13}C NMR (126 MHz, $CDCl_3$) δ = 142.0, 138.3, 129.5, 108.8, 98.1, 72.5, 32.8, 31.4, 28.7, 22.5, 14.0, 9.8 ppm;
HRMS (ESI): Exact mass calculated for $[C_{12}H_{18}Se+H]^+$: 243.0646, mass found: 243.0649.

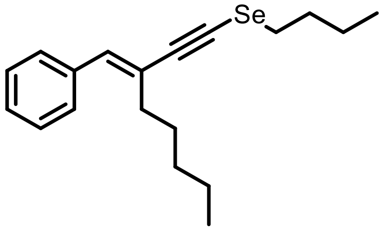

(*E*)-(3-benzylideneoct-1-yn-1-yl)(butyl)selane
Prepared according to general procedure B.
Yield 63%, yellow oil;
^{1}H NMR (500 MHz, $CDCl_3$) δ = 7.33 (t, *J* = 7.5 Hz, 2 H), 7.25-7.21 (m, 3 H), 6.79 (s, 1 H), 2.85 (t, *J* = 7.5 Hz, 2 H), 2.37 (t, *J* = 7.5 Hz, 2 H), 1.87-1.81 (m, 2 H), 1.65-1.61 (m, 2 H), 1.50-1.46 (m, 2 H), 1.33-1.29 (m, 4 H), 0.96 (t, *J* = 7.5 Hz, 3 H), 0.88 (t, *J* = 7.0 Hz, 3 H) ppm;
^{13}C NMR (126 MHz, $CDCl_3$) δ = 136.8, 135.0, 128.8, 128.2, 127.0, 126.1, 102.2, 70.0, 32.3, 31.5, 31.4, 29.4, 28.3, 22.5, 14.0, 13.5 ppm;
HRMS (ESI): Exact mass calculated for $[C_{19}H_{26}Se+H]^+$: 335.1272, mass found: 335.1270.

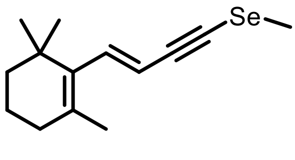

(*E*)-methyl(4-(2,6,6-trimethylcyclohex-1-en-1-yl)but-3-en-1-yn-1-yl)selane

Prepared according to general procedure B.

Yield 62%, yellow oil;

^{1}H NMR (500 MHz, $CDCl_3$) δ = 6.55 (d, *J* = 16.0 Hz, 1 H), 5.54 (d, *J* = 16.0 Hz, 1 H), 2.32 (s, 3 H), 2.00 (t, *J* = 6.3 Hz, 2 H), 1.71 (s, 3 H), 1.61-1.56 (m, 2 H), 1.45-1.42 (m, 2 H), 1.01 (s, 6 H) ppm;

^{13}C NMR (126 MHz, $CDCl_3$) δ = 140.8, 136.9, 131.6, 112.1, 98.1, 70.5, 39.6, 34.0, 33.1, 28.8, 21.6, 19.1, 9.8 ppm;

HRMS (ESI): Exact mass calculated for $[C_{14}H_{20}Se+H]^+$: 269.0803, mass found: 269.0800.

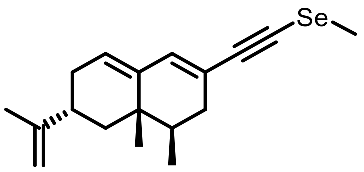

(((4*R*,4a*S*,6*R*)-4,4a-dimethyl-6-(prop-1-en-2-yl)-3,4,4a,5,6,7-hexahydronaphthalen-2-yl)ethynyl)(methyl)selane

Prepared according to general procedure C.

Yield 58%, yellow oil;

^{1}H NMR (500 MHz, $CDCl_3$) δ = 5.73 (s, 1 H), 5.58 (s, 1 H), 4.71 (s, 2 H), 2.42-2.23 (m, 6 H), 2.18-2.10 (m, 1 H), 1.92-1.86 (m, 1 H), 1.82-1.77 (m, 1 H), 1.73 (s, 3 H), 1.31-1.22 (m, 1 H), 1.15-1.07 (m, 1 H), 1.01 (d, *J* = 7.5 Hz, 3 H), 0.81 (s, 3 H) ppm;

^{13}C NMR (126 MHz, $CDCl_3$) δ = 150.1, 146.4, 137.0, 119.2, 118.7, 108.7, 98.4, 68.4, 45.6, 42.4, 41.2, 38.1, 31.29, 31.27, 20.9, 14.5, 13.3, 9.8 ppm;

HRMS (ESI): Exact mass calculated for $[C_{18}H_{24}Se+H]^+$: 321.1116, mass found: 321.1119.

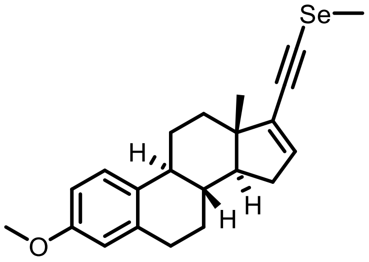

(((8*S*,9*S*,13*S*,14*S*)-3-methoxy-13-methyl-7,8,9,11,12,13,14,15-octahydro-6H-cyclopenta[a]phenanthren-17-yl)ethynyl)(methyl)selane

Prepared according to general procedure C.

Yield 65%, yellow solid, mp: 82-83 °C;

^{1}H NMR (500 MHz, $CDCl_3$) δ = 7.20 (d, *J* = 8.5 Hz, 1 H), 6.72 (dd, *J* = 8.5, 2.5 Hz, 1 H), 6.64 (d, *J* = 2.5 Hz, 1 H), 6.17-5.88 (m, 1 H), 3.78 (s, 3 H), 2.93-2.84 (m, 2 H), 2.40-1.89 (m, 10 H), 1.63-1.57 (m, 3 H), 1.48-1.42 (m, 1 H), 0.88 (s, 3 H) ppm;

^{13}C NMR (126 MHz, $CDCl_3$) δ = 157.5, 137.9, 137.6, 135.1, 132.8, 126.1, 113.9, 111.4, 94.3, 74.2, 55.4, 55.2, 48.5, 44.3, 37.6, 34.6, 31.8, 29.7, 27.8, 26.5, 16.3, 10.2 ppm;

HRMS (ESI): Exact mass calculated for $[C_{22}H_{26}OSe+H]^+$: 387.1222, mass found: 387.1220.

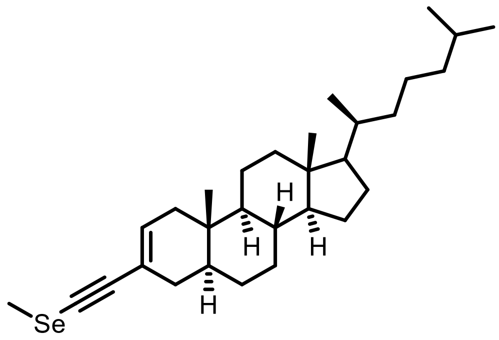

(((10*S*,13*R*)-10,13-dimethyl-17-((S)-6-methylheptan-2-yl)-4,5,6,7,8,9,10,11,12,13,14,15,16,17-tetradecahydro-1H-cyclopenta[a]phenanthren-3-yl)ethynyl)(methyl)selane

Prepared according to general procedure C.

Yield 54%, colorless solid, mp: 108-109 °C;

^{1}H NMR (500 MHz, $CDCl_3$) δ = 6.22-5.88 (m, 1 H), 2.29 (s, 3 H), 2.10-1.92 (m, 3 H), 1.90-1.73 (m, 3 H), 1.69-1.61 (m, 1 H), 1.60-1.47 (m, 2 H), 1.45-0.93 (m, 19 H), 0.92-0.81 (m, 10 H), 0.73 (s, 3 H), 0.65 (s, 3 H) ppm;

^{13}C NMR (126 MHz, $CDCl_3$) δ = 134.1, 119.8, 99.8, 67.6, 56.4, 56.3, 53.7, 42.5, 41.3, 40.3, 40.0, 39.5, 36.2, 35.8, 35.6, 34.1, 34.0, 31.7, 28.4, 28.2, 28.0, 24.2, 23.9, 22.8, 22.6, 21.0, 18.7, 12.0, 11.9, 9.8 ppm;

HRMS (ESI): Exact mass calculated for $[C_{30}H_{48}Se+H]^+$: 489.2994, mass found: 489.2993.

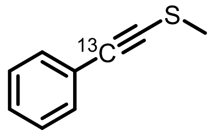

C^{13}-1a

Prepared according to general procedure C.

Yield 85%, colorless oil;

^{1}H NMR (500 MHz, $CDCl_3$) δ = 7.45-7.40 (m, 2 H), 7.31-7.28 (m, 3 H), 2.48 (s, 3 H) ppm;

^{13}C NMR (126 MHz, $CDCl_3$) δ = 131.4 (d, *J* = 1.9 Hz), 128.3 (d, *J* = 5.5 Hz), 128.0 (d, *J* = 1.8 Hz), 124.7, 91.9, 63.9, 46.1 ppm.

HRMS (ESI): Exact mass calculated for $[C_8{}^{13}CH_8S+H]^+$: 150.0453, mass found: 150.0454.

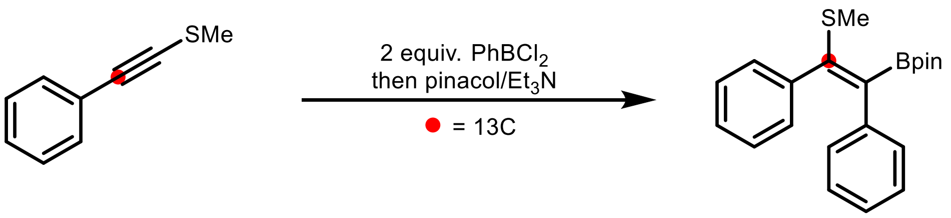

^{1}H NMR (500 MHz, $CDCl_3$) δ = 7.23-7.13 (m, 5 H), 7.08-6.97 (m, 5 H), 1.94 (d, *J* = 4.0 Hz, 3 H), 1.38 (s, 12 H) ppm.

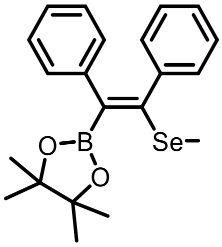

(*E*)-4,4,5,5-tetramethyl-2-(2-(methylselanyl)-1,2-diphenylvinyl)-1,3,2-dioxaborolane (5), prepared according to general procedure D.
Yield 76%, yellow oil;
^{1}H NMR (500 MHz, $CDCl_3$) δ = 7.18-7.13 (m, 4 H), 7.10-6.99 (m, 6 H), 1.77 (s, 3 H), 1.37 (s, 12 H) ppm;
^{13}C NMR (126 MHz, $CDCl_3$) δ = 142.3, 140.6, 139.3, 130.1, 129.1, 127.9, 127.8, 127.0, 125.9, 84.2, 24.8, 6.9 ppm;
^{11}B NMR (160 MHz, $CDCl_3$) δ = 30.7 ppm;
HRMS (ESI): Exact mass calculated for $[C_{21}H_{25}BO_2Se+H]^+$: 401.1186, mass found: 401.1187

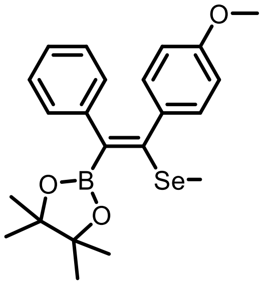

(*E*)-2-(2-(4-methoxyphenyl)-2-(methylselanyl)-1-phenylvinyl)-4,4,5,5-tetramethyl-1,3,2-dioxaborolane (6), prepared according to general procedure D.
Yield 71%, yellow oil;
^{1}H NMR (500 MHz, $CDCl_3$) δ = 7.12-7.05 (m, 4 H), 7.04-6.99 (m, 3 H), 6.70-6.65 (m, 2 H), 3.73 (s, 3 H), 1.79 (s, 3 H), 1.36 (s, 12 H) ppm;
^{13}C NMR (126 MHz, $CDCl_3$) δ = 158.5, 142.0, 140.9, 131.6, 131.4, 129.1, 127.9, 125.7, 113.3, 84.1, 55.1, 24.8, 6.9 ppm;
^{11}B NMR (160 MHz, $CDCl_3$) δ = 31.5 ppm;
HRMS (ESI): Exact mass calculated for $[C_{22}H_{27}BO_3Se+H]^+$: 431.1291, mass found: 431.1293.

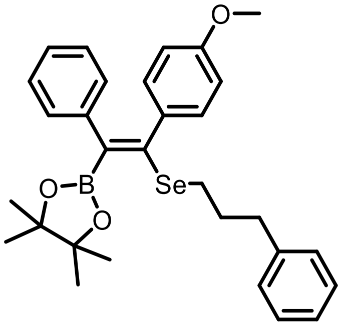

(*E*)-2-(2-(4-methoxyphenyl)-1-phenyl-2-((3-phenylpropyl)selanyl)vinyl)-4,4,5,5-tetramethyl-1,3,2-dioxaborolane (7), prepared according to general procedure D.
Yield 70%, yellow oil;
^{1}H NMR (500 MHz, $CDCl_3$) δ = 6.45-6.40 (m, 2 H), 6.37-6.22 (m, 10 H), 5.87-5.84 (m, 2 H),

2.93 (s, 3 H), 1.82 (t, *J* = 7.5 Hz, 2 H), 1.56 (t, *J* = 7.5 Hz, 2 H), 1.06-1.00 (m, 2 H), 0.57 (s, 12 H) ppm;
^{13}C NMR (126 MHz, $CDCl_3$) δ = 158.5, 141.8, 140.8, 140.0, 131.9, 131.6, 129.1, 128.6, 128.3, 127.9, 125.9, 125.7, 113.3, 84.1, 55.1, 35.5, 31.8, 25.8, 24.9 ppm;
^{11}B NMR (160 MHz, $CDCl_3$) δ = 29.9 ppm;
HRMS (ESI): Exact mass calculated for $[C_{30}H_{35}BO_3Se+H]^+$: 535.1917, mass found: 535.1916.

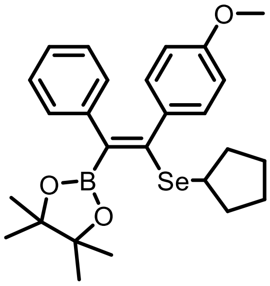

(*E*)-2-(2-(cyclopentylselanyl)-2-(4-methoxyphenyl)-1-phenylvinyl)-4,4,5,5-tetramethyl-1,3,2-dioxaborolane (8), prepared according to general procedure D.
Yield 63%, colorless solid, mp: 106-107 °C;
^{1}H NMR (500 MHz, $CDCl_3$) δ = 7.17 (d, *J* = 9.0 Hz, 2 H), 7.11-7.02 (m, 5 H), 6.67 (d, *J* = 9.0 Hz, 2 H), 3.74 (s, 3 H), 3.07-3.01 (m, 1 H), 1.79-1.64 (m, 6 H), 1.50-1.43 (m, 2 H), 1.36 (s, 12 H) ppm;
^{13}C NMR (126 MHz, $CDCl_3$) δ = 158.4, 140.7, 140.6, 133.0, 131.5, 129.1, 127.9, 125.9, 113.1, 84.0, 55.1, 40.5, 33.5, 24.9, 24.8 ppm;
^{11}B NMR (160 MHz, $CDCl_3$) δ = 31.1 ppm;
HRMS (ESI): Exact mass calculated for $[C_{26}H_{33}BO_3Se+H]^+$: 485.1761, mass found: 485.1760.

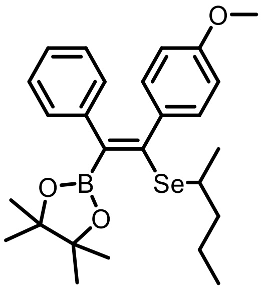

(*E*)-2-(2-(4-methoxyphenyl)-2-(pentan-2-ylselanyl)-1-phenylvinyl)-4,4,5,5-tetramethyl-1,3,2-dioxaborolane (9), prepared according to general procedure D.
Yield 65%, colorless solid, mp: 86-87 °C;
^{1}H NMR (500 MHz, $CDCl_3$) δ = 7.18 (d, *J* = 9.0 Hz, 2 H), 7.10-7.02 (m, 5 H), 6.66 (d, *J* = 9.0 Hz, 2 H), 3.74 (s, 3 H), 2.75-2.67 (m, 1 H), 1.58-1.41 (m, 3 H), 1.37 (s, 6 H), 1.36 (s, 6 H), 1.32-1.28 (m, 4 H), 0.79 (t, *J* = 7.5 Hz, 3 H) ppm;
^{13}C NMR (126 MHz, $CDCl_3$) δ = 158.5, 140.8, 139.9, 132.9, 131.6, 129.1, 127.9, 125.9, 113.1, 83.9, 55.1, 39.6, 37.6, 24.9, 24.8, 22.1, 20.7, 13.9 ppm;
^{11}B NMR (160 MHz, $CDCl_3$) δ = 32.8 ppm;
HRMS (ESI): Exact mass calculated for $[C_{26}H_{35}BO_3Se+H]^+$: 487.1917, mass found: 487.1916.

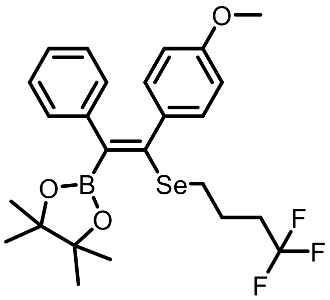

(*E*)-2-(2-(4-methoxyphenyl)-1-phenyl-2-((4,4,4-trifluorobutyl)selanyl)vinyl)-4,4,5,5-tetramethyl-1,3,2-dioxaborolane (10), prepared according to general procedure D.

Yield 64%, yellow solid, mp: 71-72 °C;

^{1}H NMR (500 MHz, $CDCl_3$) δ = 7.16 (d, *J* = 8.5 Hz, 2 H), 7.13-7.03 (m, 5 H), 6.70 (d, *J* = 8.5 Hz, 2 H), 3.77 (s, 3 H), 2.40 (t, *J* = 7.5 Hz, 2 H), 2.27-2.18 (m, 2 H), 1.81-1.75 (m, 2 H), 1.38 (s, 12 H) ppm;

^{13}C NMR (126 MHz, $CDCl_3$) δ = 158.7, 140.6, 138.9, 131.54, 131.51, 129.0, 128.0, 126.0, 113.4, 84.1, 55.1, 33.2 (q, *J* = 29.0 Hz), 24.8, 24.6, 22.42, 22.39 ppm;

^{11}B NMR (160 MHz, $CDCl_3$) δ = 32.2 ppm;

HRMS (ESI): Exact mass calculated for $[C_{25}H_{30}BF_3O_3Se+H]^+$: 527.1478, mass found: 527.1476.

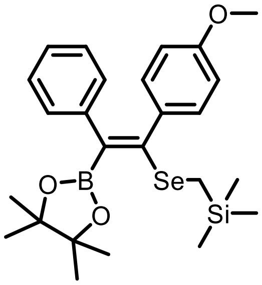

(*E*)-(((1-(4-methoxyphenyl)-2-phenyl-2-(4,4,5,5-tetramethyl-1,3,2-dioxaborolan-2-yl)vinyl)selanyl)methyl)trimethylsilane (11), prepared according to general procedure D.

Yield 67%, yellow solid, mp: 110-111 °C;

^{1}H NMR (500 MHz, $CDCl_3$) δ = 7.12 (d, *J* = 8.5 Hz, 2 H), 7.09-7.06 (m, 2 H), 7.04-7.01 (m, 3 H), 6.68 (d, *J* = 8.5 Hz, 2 H), 3.75 (s, 3 H), 1.53 (s, 2 H), 1.37 (s, 12 H), 0.04 (s, 9 H) ppm;

^{13}C NMR (126 MHz, $CDCl_3$) δ = 158.4, 142.5, 140.9, 131.7, 131.5, 129.1, 127.9, 125.7, 113.2, 84.0, 55.1, 24.8, 11.3, -1.2 ppm;

^{11}B NMR (160 MHz, $CDCl_3$) δ = 31.4 ppm;

HRMS (ESI): Exact mass calculated for $[C_{25}H_{35}BO_3SeSi+H]^+$: 503.1687, mass found: 503.1685.

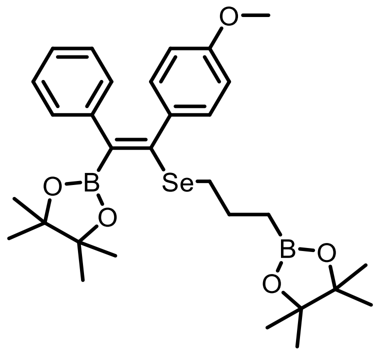

(*E*)-2-(2-(4-methoxyphenyl)-1-phenyl-2-((3-(4,4,5,5-tetramethyl-1,3,2-dioxaborolan-2-yl)propyl)selanyl)vinyl)-4,4,5,5-tetramethyl-1,3,2-dioxaborolane (12), prepared according to general procedure D.

Yield 48%, yellow oil;

^{1}H NMR (500 MHz, $CDCl_3$) δ = 7.13 (d, *J* = 8.5 Hz, 2 H), 7.09-7.05 (m, 2 H), 7.03-7.00 (m, 3 H), 6.65 (d, *J* = 8.5 Hz, 2 H), 3.72 (s, 3 H), 2.36 (t, *J* = 7.5 Hz, 2 H), 1.65 (quint, *J* = 7.5 Hz, 2 H), 1.35 (s, 12 H), 1.19 (s, 12 H), 0.77 (t, *J* = 7.5 Hz, 2 H) ppm;

^{13}C NMR (126 MHz, $CDCl_3$) δ = 158.4, 140.9, 140.5, 132.1, 131.5, 129.1, 127.9, 125.8, 113.2, 84.0, 82.9, 55.1, 29.0, 24.84, 24.79, 24.7 ppm;

^{11}B NMR (160 MHz, $CDCl_3$) δ = 34.3, 22.6 ppm;

HRMS (ESI): Exact mass calculated for $[C_{30}H_{42}B_2O_5Se+H]^+$: 585.2456, mass found: 585.2504.

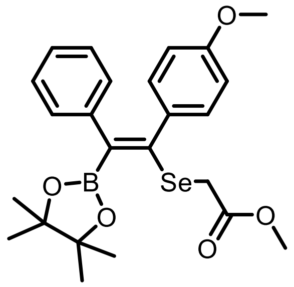

Methyl (*E*)-2-((1-(4-methoxyphenyl)-2-phenyl-2-(4,4,5,5-tetramethyl-1,3,2-dioxaborolan-2-yl)vinyl)selanyl)acetate (13), prepared according to general procedure D.

Yield 67%, yellow solid, mp: 88-90 ^oC;

^{1}H NMR (500 MHz, $CDCl_3$) δ = 7.14 (d, *J* = 8.5 Hz, 2 H), 7.10-7.01 (m, 5 H), 6.68 (d, *J* = 8.5 Hz, 2 H), 3.73 (s, 3 H), 3.66 (s, 3 H), 3.00 (s, 2 H), 1.34 (s, 12 H) ppm;

^{13}C NMR (126 MHz, $CDCl_3$) δ = 171.2, 158.8, 140.4, 139.0, 131.7, 131.3, 128.9, 128.0, 126.2, 113.3, 84.2, 55.1, 52.2, 25.5, 24.8 ppm;

^{11}B NMR (160 MHz, $CDCl_3$) δ = 31.5 ppm;

HRMS (ESI): Exact mass calculated for $[C_{24}H_{29}BO_5Se+H]^+$: 489.1346, mass found: 489.1345.

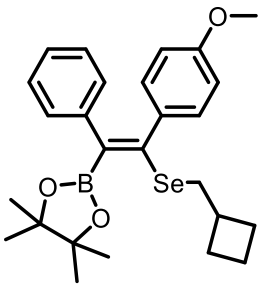

(*E*)-2-(2-((cyclobutylmethyl)selanyl)-2-(4-methoxyphenyl)-1-phenylvinyl)-4,4,5,5-tetramethyl-1,3,2-dioxaborolane (14), prepared according to general procedure D.
Yield 77%, colorless solid, mp: 85-85 ^{o}C;
^{1}H NMR (500 MHz, $CDCl_3$) δ = 7.14 (d, *J* = 7.5 Hz, 2 H), 7.10-7.05 (m, 2 H), 7.05-6.99 (m, 3 H), 6.67 (d, *J* = 7.5 Hz, 2 H), 3.74 (s, 3 H), 2.42 (d, *J* = 7.5 Hz, 2 H), 2.08-1.98 (m, 2 H), 1.82-1.69 (m, 2 H), 1.58-1.55 (m, 1 H), 1.36 (s, 12 H), 0.89-0.77 (m, 2 H) ppm;
^{13}C NMR (126 MHz, $CDCl_3$) δ = 158.5, 140.8, 140.0, 132.1, 131.6, 129.1, 127.9, 125.8, 113.2, 84.0, 55.1, 35.9, 33.4, 28.7, 24.8, 17.5 ppm;
^{11}B NMR (160 MHz, $CDCl_3$) δ = 32.6 ppm;
HRMS (ESI): Exact mass calculated for $[C_{26}H_{33}BO_3Se+H]^+$: 485.1761, mass found: 485.1760.

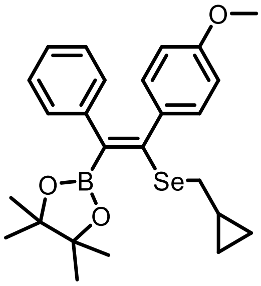

(*E*)-2-(2-((cyclopropylmethyl)selanyl)-2-(4-methoxyphenyl)-1-phenylvinyl)-4,4,5,5-tetramethyl-1,3,2-dioxaborolane (15), prepared according to general procedure D.
Yield 68%, yellow solid, mp: 93-94 ^{o}C;
^{1}H NMR (500 MHz, $CDCl_3$) δ = 7.15 (d, *J* = 8.5 Hz, 2 H), 7.10-7.06 (m, 2 H), 7.06-6.99 (m, 3 H), 6.67 (d, *J* = 8.5 Hz, 2 H), 3.74 (s, 3 H), 2.33 (d, *J* = 7.5 Hz, 2 H), 1.37 (s, 12 H), 0.94-0.88 (m, 1 H), 0.55-0.47 (m, 2 H), 0.12-0.05 (m, 2 H) ppm;
^{13}C NMR (126 MHz, $CDCl_3$) δ = 158.5, 140.8, 140.4, 132.4, 131.5, 129.0, 127.9, 125.8, 113.2, 84.0, 55.1, 32.5, 24.8, 11.5, 6.9 ppm;
^{11}B NMR (160 MHz, $CDCl_3$) δ = 32.3 ppm;
HRMS (ESI): Exact mass calculated for $[C_{25}H_{31}BO_3Se+H]^+$: 471.1604, mass found: 471.1603.

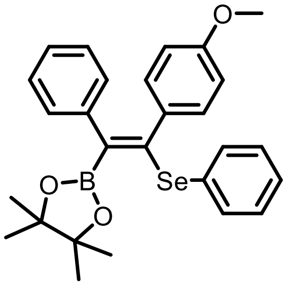

(*E*)-2-(2-(4-methoxyphenyl)-1-phenyl-2-(phenylselanyl)vinyl)-4,4,5,5-tetramethyl-1,3,2-dioxaborolane (16), prepared according to general procedure D.
Yield 43%, yellow solid, mp: 157-158 ^{o}C;

^{1}H NMR (500 MHz, CD_2Cl_2) δ = 7.33 (d, *J* = 8.5 Hz, 2 H), 7.16-7.04 (m, 10 H), 6.54 (d, *J* = 8.5 Hz, 2 H), 3.65 (s, 3 H), 1.35 (s, 12 H) ppm;
^{13}C NMR (126 MHz, $CDCl_3$) δ = 158.7, 141.5, 140.0, 134.9, 131.7, 130.6, 130.4, 129.1, 128.5, 128.1, 126.43, 126.38, 113.0, 84.2, 55,0, 24.7 ppm;
^{11}B NMR (160 MHz, $CDCl_3$) δ = 32.7 ppm;
HRMS (ESI): Exact mass calculated for $[C_{27}H_{29}BO_3Se+H]^+$: 493.1448, mass found: 493.1461.

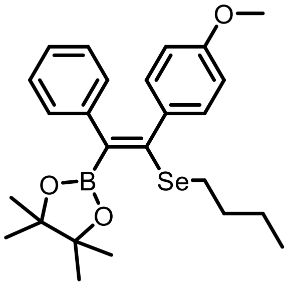

(*E*)-2-(2-(butylselanyl)-2-(4-methoxyphenyl)-1-phenylvinyl)-4,4,5,5-tetramethyl-1,3,2-dioxaborolane (17), prepared according to general procedure D.
Yield 78%, yellow solid, mp: 69-70 °C;
^{1}H NMR (500 MHz, $CDCl_3$) δ = 7.13 (d, *J* = 8.5 Hz, 2 H), 7.10-7.06 (m, 2 H), 7.05-7.01 (m, 3 H), 6.67 (d, *J* = 8.5 Hz, 2 H), 3.74 (s, 3 H), 2.34 (t, *J* = 7.5 Hz, 2 H), 1.53-1.49 (m, 2 H), 1.36 (s, 12 H), 1.31-1.27 (m, 2 H), 0.80 (t, *J* = 7.5 Hz, 3 H) ppm;
^{13}C NMR (126 MHz, $CDCl_3$) δ = 158.5, 140.8, 140.2, 132.1, 131.5, 129.1, 127.9, 125.8, 113.2, 84.0, 55.1, 32.2, 26.2, 24.8, 22.8, 13.5 ppm;
^{11}B NMR (160 MHz, $CDCl_3$) δ = 31.2 ppm;
HRMS (ESI): Exact mass calculated for $C_{25}H_{33}BO_3Se+H^+$: 473.1761, mass found: 473.1759.

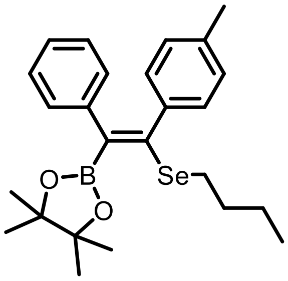

(*E*)-2-(2-(butylselanyl)-1-phenyl-2-(p-tolyl)vinyl)-4,4,5,5-tetramethyl-1,3,2-dioxaborolane (18), prepared according to general procedure D.
Yield 71%, colorless solid, mp: 73-74 °C;
^{1}H NMR (500 MHz, $CDCl_3$) δ = 7.10-7.05 (m, 4 H), 7.04-7.00 (m, 3 H), 6.94 - 6.91(m, 2 H), 2.34 (t, *J* = 7.5 Hz, 2 H), 2.25 (s, 3 H), 1.53-1.50 (m, 2 H), 1.36 (s, 12 H), 1.29-1.27 (m, 2 H), 0.80 (t, *J* = 7.5 Hz, 3 H) ppm;
^{13}C NMR (126 MHz, $CDCl_3$) δ = 140.8, 140.6, 136.8, 136.7, 130.1, 129.1, 128.5, 127.8, 125.8, 84.0, 32.2, 26.2, 24.8, 22.7, 21.2, 13.5 ppm;
^{11}B NMR (160 MHz, $CDCl_3$) δ = 29.8 ppm;
HRMS (ESI): Exact mass calculated for $[C_{25}H_{33}BO_2Se+H]^+$: 457.1812, mass found: 457.1811.

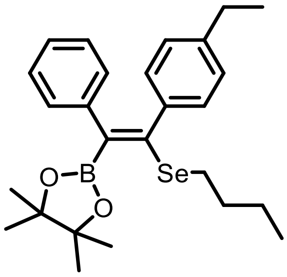

(*E*)-2-(2-(butylselanyl)-2-(4-ethylphenyl)-1-phenylvinyl)-4,4,5,5-tetramethyl-1,3,2-dioxaborolane (19), prepared according to general procedure D.

Yield 75%, colorless solid, mp: 76-77 °C;

^{1}H NMR (500 MHz, $CDCl_3$) δ = 7.13-7.01 (m, 7 H), 6.99-6.95 (m, 2 H), 2.55 (q, *J* = 7.5 Hz, 2 H), 2.34 (t, *J* = 7.5 Hz, 2 H), 1.56-1.48 (m, 2 H), 1.37 (s, 12 H), 1.28 (q, *J* = 7.5 Hz, 2 H), 1.17 (t, *J* = 7.5 Hz, 3 H), 0.79 (t, *J* = 7.5 Hz, 3 H) ppm;

^{13}C NMR (126 MHz, $CDCl_3$) δ = 143.0, 140.74, 140.70, 137.1, 130.1, 129.1, 127.8, 127.2, 125.8, 84.0, 32.2, 28.5, 26.2, 24.8, 22.7, 15.2, 13.5 ppm;

^{11}B NMR (160 MHz, $CDCl_3$) δ = 31.9 ppm;

HRMS (ESI): Exact mass calculated for $[C_{26}H_{35}BO_2Se+H]^+$: 471.1968, mass found: 471.1967.

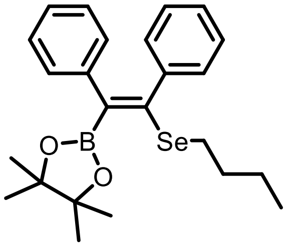

(*E*)-2-(2-(butylselanyl)-1,2-diphenylvinyl)-4,4,5,5-tetramethyl-1,3,2-dioxaborolane (20), prepared according to general procedure D.

Yield 57%, yellow oil;

^{1}H NMR (500 MHz, $CDCl_3$) δ = 7.21-7.17 (m, 2 H), 7.16-7.00 (m, 8 H), 2.33 (t, *J* = 7.5 Hz, 2 H), 1.54-1.47 (m, 2 H), 1.37 (s, 12 H), 1.30-1.24 (m, 2 H), 0.79 (t, *J* = 7.5 Hz, 3 H) ppm;

^{13}C NMR (126 MHz, $CDCl_3$) δ = 140.55, 140.53, 139.9, 130.1, 129.1, 127.8, 127.78, 127.0, 126.0, 84.1, 32.2, 26.2, 24.8, 22.3, 13.5 ppm;

^{11}B NMR (160 MHz, $CDCl_3$) δ = 31.8 ppm;

HRMS (ESI): Exact mass calculated for $[C_{24}H_{31}BO_2Se+H]^+$: 443.1655, mass found: 443.1659.

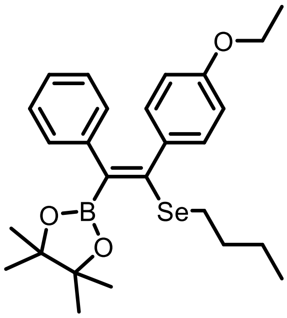

(*E*)-2-(2-(butylselanyl)-2-(4-ethoxyphenyl)-1-phenylvinyl)-4,4,5,5-tetramethyl-1,3,2-dioxaborolane (21), prepared according to general procedure D.

Yield 63%, yellow solid, mp: 75-76 °C;

^{1}H NMR (500 MHz, $CDCl_3$) δ = 7.14-6.99 (m, 7 H), 6.72-6.61 (m, 2 H), 3.95 (q, *J* = 7.0 Hz, 2

H), 2.34 (t, J = 7.5 Hz, 2 H), 1.55-1.49 (m, 2 H), 1.35-1.38 (m, 15 H), 1.31-1.26 (m, 2 H), 0.80 (t, J = 7.5 Hz, 3 H) ppm;
^{13}C NMR (126 MHz, $CDCl_3$) δ = 157.9, 140.8, 140.4, 131.9, 131.5, 129.1, 127.9, 125.8, 113.7, 84.0, 63.3, 32.2, 26.2, 24.8, 22.7, 14.8, 13.5 ppm;
^{11}B NMR (160 MHz, $CDCl_3$) δ = 31.7 ppm;
HRMS (ESI): Exact mass calculated for $[C_{26}H_{35}BO_3Se+H]^+$: 487.1917, mass found: 487.1915.

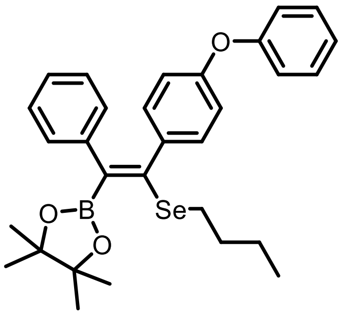

(*E*)-2-(2-(butylselanyl)-2-(4-phenoxyphenyl)-1-phenylvinyl)-4,4,5,5-tetramethyl-1,3,2-dioxaborolane (22), prepared according to general procedure D.
Yield 71%, yellow oil;
^{1}H NMR (500 MHz, $CDCl_3$) δ = 7.32-7.28 (m, 2 H), 7.18-7.14 (m, 2 H), 7.11-7.03 (m, 6 H), 6.96-6.93 (m, 2 H), 6.80-6.75 (m, 2 H), 2.39 (t, J = 7.5 Hz, 2 H), 1.55-1.52 (m, 2 H), 1.37 (s, 12 H), 1.34-1.30 (m, 2 H), 0.82 (t, J = 7.5 Hz, 3 H) ppm;
^{13}C NMR (126 MHz, $CDCl_3$) δ = 157.0, 156.1, 140.7, 139.9, 134.9, 131.7, 129.7, 129.1, 127.9, 126.0, 123.3, 118.9, 118.1, 84.1, 32.2, 26.3, 24.8, 22.8, 13.5 ppm;
^{11}B NMR (160 MHz, $CDCl_3$) δ = 32.0 ppm;
HRMS (ESI): Exact mass calculated for $[C_{30}H_{35}BO_3Se+H]^+$: 535.1917, mass found: 535.1961.

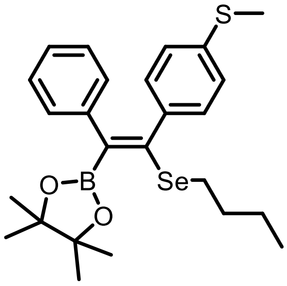

(*E*)-2-(2-(butylselanyl)-2-(4-(methylthio)phenyl)-1-phenylvinyl)-4,4,5,5-tetramethyl-1,3,2-dioxaborolane (23), prepared according to general procedure D.
Yield 47%, yellow solid, mp: 92-93 ^{o}C;
^{1}H NMR (500 MHz, $CDCl_3$) δ = 7.15-6.98 (m, 9 H), 2.42 (s, 3 H), 2.35 (t, J = 7.5 Hz, 2 H), 1.53-1.48 (m, 2 H), 1.36 (s, 12 H), 1.30-1.27 (m, 2 H), 0.80 (t, J = 7.5 Hz, 3 H) ppm;
^{13}C NMR (126 MHz, $CDCl_3$) δ = 140.5, 139.7, 137.2, 136.6, 130.7, 129.0, 128.0, 126.0, 125.5, 84.1, 32.2, 26.3, 24.8, 22.7, 15.4, 13.5 ppm;
^{11}B NMR (160 MHz, $CDCl_3$) δ = 31.6 ppm;
HRMS (ESI): Exact mass calculated for $[C_{25}H_{33}BO_2SSe+H]^+$: 489.1532, mass found: 489.1531.

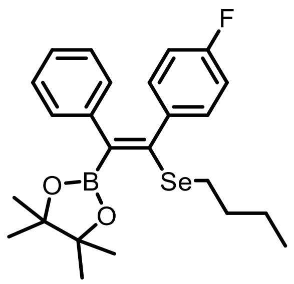

(*E*)-2-(2-(butylselanyl)-2-(4-fluorophenyl)-1-phenylvinyl)-4,4,5,5-tetramethyl-1,3,2-dioxaborolane (24), prepared according to general procedure D.
Yield 35%, colorless solid, mp: 100-101 °C;
^{1}H NMR (500 MHz, $CDCl_3$) δ = 7.20-7.15 (m, 2 H), 7.10-6.99 (m, 5 H), 6.85-6.80 (m, 2 H), 2.34 (t, *J* = 7.5 Hz, 2 H), 1.54 – 1.48 (m, 2 H), 1.36 (s, 12 H), 1.31-1.27 (m, 2 H), 0.80 (t, *J* = 7.5 Hz, 3 H) ppm;
^{13}C NMR (126 MHz, $CDCl_3$) δ = 161.6 (d, *J* = 246.9 Hz), 140.4, 139.2, 135.9 (d, *J* = 2.5 Hz), 131.8 (d, *J* = 7.5 Hz), 129.0, 127.9, 126.1, 114.7 (d, *J* = 21.4 Hz), 84.1, 32.1, 26.2, 24.8, 22.7, 13.5 ppm;
^{11}B NMR (160 MHz, $CDCl_3$) δ = 31.7 ppm;
HRMS (ESI): Exact mass calculated for $[C_{24}H_{30}BFO_2Se+H]^+$: 461.1561, mass found: 461.1561.

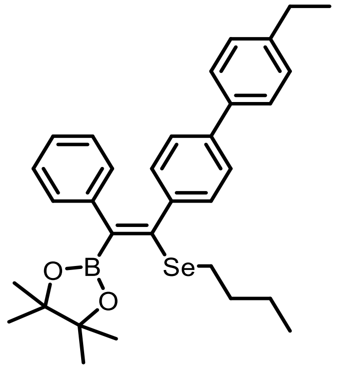

(*E*)-2-(2-(butylselanyl)-2-(4'-ethyl-[1,1'-biphenyl]-4-yl)-1-phenylvinyl)-4,4,5,5-tetramethyl-1,3,2-dioxaborolane (25), prepared according to general procedure D.
Yield 77%, yellow solid, mp: 99-100 °C;
^{1}H NMR (500 MHz, $CDCl_3$) δ = 7.40 (d, *J* = 8 Hz, 2 H), 7.30 (d, *J* = 8.5 Hz, 2 H), 7.18 (d, *J* = 8.5 Hz, 2 H), 7.15 (d, *J* = 8 Hz, 2 H), 7.01-6.97 (m, 4 H), 6.97-6.93 (m, 1 H), 2.59 (q, *J* = 7.5 Hz, 2 H), 2.31 (t, *J* = 7.5 Hz, 2 H), 1.48-1.43 (m, 2 H), 1.30 (s, 12 H), 1.20-1.15 (m, 5 H), 0.72 (t, *J* = 7.5 Hz, 3 H) ppm;
^{13}C NMR (126 MHz, $CDCl_3$) δ = 143.5, 140.6, 140.1, 139.4, 138.6, 137.9, 130.7, 129.1, 128.3, 127.9, 126.8, 126.2, 126.0, 84.1, 32.2, 28.5, 26.3, 24.9, 22.8, 15.6, 13.5 ppm;
^{11}B NMR (160 MHz, $CDCl_3$) δ = 31.1 ppm;
HRMS (ESI): Exact mass calculated for $[C_{32}H_{39}BO_2Se+H]^+$: 547.2281, mass found: 547.2280.

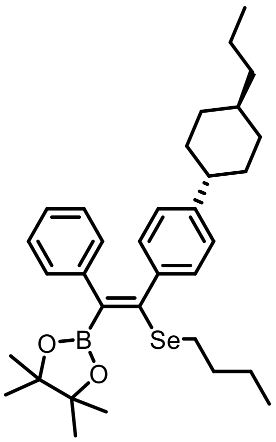

2-((*E*)-2-(butylselanyl)-1-phenyl-2-(4-((1s,4r)-4-propylcyclohexyl)phenyl)vinyl)-4,4,5,5-tetramethyl-1,3,2-dioxaborolane (26), prepared according to general procedure D.
Yield 61%, yellow solid, mp: 76-77 °C;
^{1}H NMR (500 MHz, $CDCl_3$) δ = 7.09 (d, *J* = 8.0 Hz, 2 H), 7.07-6.99 (m, 5 H), 6.96 (d, *J* = 8.0 Hz, 2 H), 2.35-2.32 (m, 2 H), 1.85-1.79 (m, 4 H), 1.53-1.47 (m, 2 H), 1.37 (s, 12 H), 1.35-1.16 (m, 10 H), 1.04-0.96 (m, 2 H), 0.89 (t, *J* = 7.5 Hz, 3 H), 0.77 (t, *J* = 7.5 Hz, 3 H) ppm;
^{13}C NMR (126 MHz, $CDCl_3$) δ = 146.7, 140.8, 140.7, 137.1, 130.0, 129.1, 127.8, 126.2, 125.8, 84.0, 44.2, 39.7, 37.0, 34.2, 33.5, 32.2, 26.2, 24.8, 22.7, 20.0, 14.4, 13.5 ppm;
^{11}B NMR (160 MHz, $CDCl_3$) δ = 32.0 ppm;
HRMS (ESI): Exact mass calculated for $[C_{33}H_{47}BO_2Se+H]^+$: 567.2907, mass found: 567.2904.

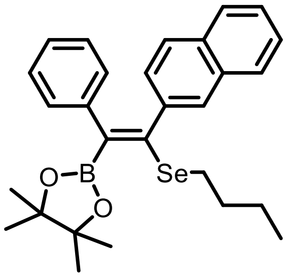

(*E*)-2-(2-(butylselanyl)-2-(naphthalen-2-yl)-1-phenylvinyl)-4,4,5,5-tetramethyl-1,3,2-dioxaborolane(27), prepared according to general procedure D.
Yield 64%, yellow solid, mp: 89-90 °C;
^{1}H NMR (500 MHz, $CDCl_3$) δ = 7.76-7.70 (m, 2 H), 7.70-7.65 (m, 1 H), 7.59 (d, *J* = 8.5 Hz, 1 H), 7.42-7.38 (m, 2 H), 7.30 (d, *J* = 8.5 Hz, 1 H), 7.09-6.97 (m, 5 H), 2.34 (t, *J* = 7.5 Hz, 2 H), 1.54-1.49 (m, 2 H), 1.40 (s, 12 H), 1.28-1.24 (m, 2 H), 0.76 (t, *J* = 7.5 Hz, 3 H) ppm;
^{13}C NMR (126 MHz, $CDCl_3$) δ = 140.5, 140.1, 137.5, 133.1, 132.4, 129.3, 129.1, 128.4, 128.2, 128.0, 127.6, 127.2, 126.1, 125.9, 125.8, 84.1, 32.2, 26.3, 24.9, 22.7, 13.5 ppm;
^{11}B NMR (160 MHz, $CDCl_3$) δ = 31.9 ppm;
HRMS (ESI): Exact mass calculated for $[C_{28}H_{33}BO_2Se+H]^+$: 493.1812, mass found: 493.1818.

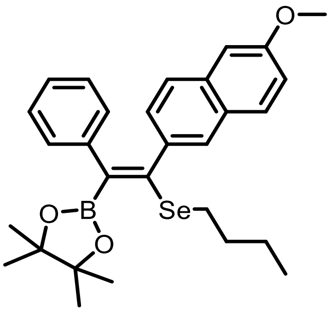

(*E*)-2-(2-(butylselanyl)-2-(6-methoxynaphthalen-2-yl)-1-phenylvinyl)-4,4,5,5-tetramethyl-1,3,2-dioxaborolane (28), prepared according to general procedure D.
Yield 70%, yellow oil;
^{1}H NMR (500 MHz, $CDCl_3$) δ = 7.60-7.56 (m, 1 H), 7.49 (d, *J* = 8.5 Hz, 1 H), 7.40 (d, *J* = 8.5 Hz, 1 H), 7.20-7.15 (m, 1 H), 7.02-6.87 (m, 7 H), 3.80 (s, 3 H), 2.25 (t, *J* = 7.5 Hz, 2 H), 1.43 (t, *J* = 7.5 Hz, 2 H), 1.31 (s, 12 H), 1.20-1.15 (m, 2 H), 0.68 (t, *J* = 7.5 Hz, 3 H) ppm;
^{13}C NMR (126 MHz, $CDCl_3$) δ = 157.8, 140.6, 140.4, 135.1, 133.6, 129.7, 129.2, 129.1, 129.0, 128.5, 127.9, 126.1, 125.9, 118.6, 105.6, 84.1, 55.3, 32.2, 26.3, 24.9, 22.7, 13.5 ppm;
^{11}B NMR (160 MHz, $CDCl_3$) δ = 32.7 ppm;
HRMS (ESI): Exact mass calculated for $[C_{29}H_{35}BO_3Se+H]^+$: 523.1917, mass found: 523.1920.

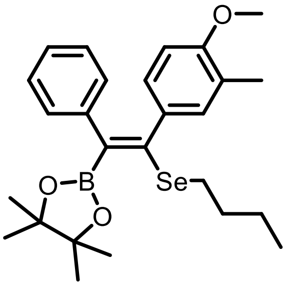

(*E*)-2-(2-(butylselanyl)-2-(4-methoxy-3-methylphenyl)-1-phenylvinyl)-4,4,5,5-tetramethyl-1,3,2-dioxaborolane (29), prepared according to general procedure D.
Yield 53%, yellow oil;
^{1}H NMR (500 MHz, $CDCl_3$) δ = 7.10-7.00 (m, 6 H), 6.94-6.92 (m, 1 H), 6.59-6.50 (m, 1 H), 3.75 (s, 3 H), 2.35 (t, *J* = 7.5 Hz, 2 H), 2.08 (s, 3 H), 1.55-1.49 (m, 2 H), 1.37 (s, 12 H), 1.31-1.27 (m, 2 H), 0.81 (t, *J* = 7.5 Hz, 3 H) ppm;
^{13}C NMR (126 MHz, $CDCl_3$) δ = 156.7, 140.9, 140.6, 132.5, 131.5, 129.1, 128.9, 127.8, 125.7, 109.0, 83.9, 55.2, 32.2, 26.2, 24.8, 22.7, 16.2, 13.5 ppm;
^{11}B NMR (160 MHz, $CDCl_3$) δ = 32.1 ppm;
HRMS (ESI): Exact mass calculated for $[C_{26}H_{35}BO_3Se+H]^+$: 487.1917, mass found: 487.1932.

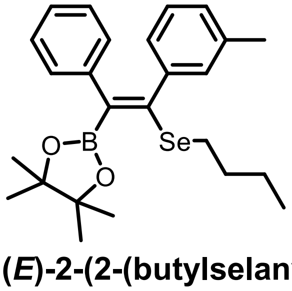

(*E*)-2-(2-(butylselanyl)-1-phenyl-2-(m-tolyl)vinyl)-4,4,5,5-tetramethyl-1,3,2-dioxaborolane (30), prepared according to general procedure D.
Yield 51%, colorless solid, mp: 58-59 ^oC;

^{1}**H NMR** (500 MHz, $CDCl_3$) δ = 7.09-6.97 (m, 7 H), 6.95-6.88 (m, 2 H), 2.34 (t, *J* = 7.5 Hz, 2 H), 2.20 (s, 3 H), 1.52-1.49 (m, 2 H), 1.37 (s, 12 H), 1.29-1.30 (m, 2 H), 0.79 (t, *J* = 7.5 Hz, 3 H) ppm;
^{13}**C NMR** (126 MHz, $CDCl_3$) δ = 140.69, 140.66, 139.8, 137.2, 130.7, 129.0, 127.8, 127.7, 127.5, 127.3, 125.9, 84.0, 32.2, 26.2, 24.8, 22.7, 21.3, 13.5 ppm;
^{11}**B NMR** (160 MHz, $CDCl_3$) δ = 31.9 ppm;
HRMS (ESI): Exact mass calculated for $[C_{25}H_{33}BO_2Se+H]^+$: 457.1812, mass found: 457.1818.

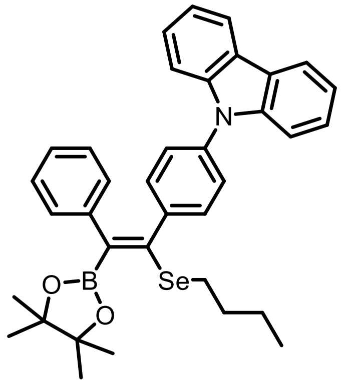

(*E*)-9-(4-(1-(butylselanyl)-2-phenyl-2-(4,4,5,5-tetramethyl-1,3,2-dioxaborolan-2-yl)vinyl)phenyl)-9H-carbazole (31), prepared according to general procedure D.
Yield 61%, brown semi-solid;
^{1}**H NMR** (500 MHz, $CDCl_3$) δ = 8.15-8.10 (m, 2 H), 7.44-7.38 (m, 4 H), 7.36-7.26 (m, 6 H), 7.18-7.09 (m, 5 H), 2.50 (t, *J* = 7.5 Hz, 2 H), 1.64-1.59 (m, 2 H), 1.40 (s, 12 H), 1.37-1.32 (m, 2 H), 0.85 (t, *J* = 7.5 Hz, 3 H) ppm;
^{13}**C NMR** (126 MHz, $CDCl_3$) δ = 140.7, 140.4, 139.4, 139.3, 136.2, 131.6, 129.1, 128.0, 126.3, 125.9, 123.4, 120.3, 119.9, 109.7, 84.2, 32.3, 26.4, 24.9, 22.8, 13.5 ppm;
^{11}**B NMR** (160 MHz, $CDCl_3$) δ = 31.9 ppm;
HRMS (ESI): Exact mass calculated for $[C_{36}H_{38}BNO_2Se+H]^+$: 608.2234, mass found: 608.2240.

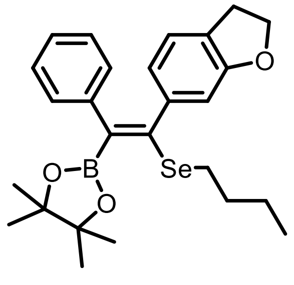

(*E*)-2-(2-(butylselanyl)-2-(2,3-dihydrobenzofuran-6-yl)-1-phenylvinyl)-4,4,5,5-tetramethyl-1,3,2-dioxaborolane (32), prepared according to general procedure D.
Yield 63%, yellow oil;
^{1}**H NMR** (500 MHz, $CDCl_3$) δ = 7.10-7.00 (m, 6 H), 6.91 (d, *J* = 8.5 Hz, 1 H), 6.51 (d, *J* = 8.5 Hz, 1 H), 4.51 (t, *J* = 8.5 Hz, 2 H), 3.07 (t, *J* = 8.5 Hz, 2 H), 2.36 (t, *J* = 7.5 Hz, 2 H), 1.56-1.50 (m, 2 H), 1.36 (s, 12 H), 1.32-1.27 (m, 2 H), 0.81 (t, *J* = 7.5 Hz, 3 H) ppm;
^{13}**C NMR** (126 MHz, $CDCl_3$) δ = 159.2, 141.0, 140.8, 132.2, 130.6, 129.2, 128.0, 126.9, 126.6, 125.9, 108.5, 84.1, 71.4, 32.3, 29.6, 26.3, 24.9, 22.8, 13.6 ppm;
^{11}**B NMR** (160 MHz, $CDCl_3$) δ = 31.8 ppm;

HRMS (ESI): Exact mass calculated for $[C_{26}H_{33}BO_3Se+H]^+$: 485.1761, mass found: 485.1767.

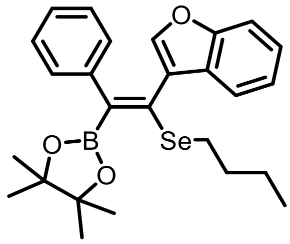

(*E*)-2-(2-(benzofuran-3-yl)-2-(butylselanyl)-1-phenylvinyl)-4,4,5,5-tetramethyl-1,3,2-dioxaborolane (33), prepared according to general procedure D.
Yield 38%, yellow oil;
^{1}H NMR (500 MHz, $CDCl_3$) δ = 7.60-7.53 (m, 1 H), 7.50-7.46 (m, 1 H), 7.18-7.06 (m, 7 H), 7.02 (s, 1 H), 2.54 (t, *J* = 7.5 Hz, 2 H), 1.59-1.54 (m, 2 H), 1.28 (s, 12 H), 1.28-1.22 (m, 2 H), 0.77 (t, *J* = 7.5 Hz, 3 H) ppm;
^{13}C NMR (126 MHz, $CDCl_3$) δ = 143.6, 140.7, 140.2, 139.4, 130.1, 128.6, 128.3, 126.9, 125.6, 124.3, 124.0, 123.7, 122.1, 84.2, 32.2, 27.7, 24.8, 22.8, 13.6 ppm;
^{11}B NMR (160 MHz, $CDCl_3$) δ = 30.7 ppm;
HRMS (ESI): Exact mass calculated for $[C_{26}H_{31}BO_3Se+H]^+$: 483.1604, mass found: 483.1601.

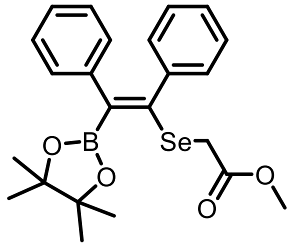

Methyl (*E*)-2-((1,2-diphenyl-2-(4,4,5,5-tetramethyl-1,3,2-dioxaborolan-2-yl)vinyl)selanyl)acetate (34), prepared according to general procedure D.
Yield 81%, Yellow solid, mp: 68-70 ^oC;
^{1}H NMR (500 MHz, $CDCl_3$) δ = 7.22-7.19 (m, 2 H), 7.17-7.12 (m, 3 H), 7.08-7.04 (m, 3 H), 7.04-7.01 (m, 2 H), 3.66 (s, 3 H), 3.00 (s, 2 H), 1.35 (s, 12 H) ppm;
^{13}C NMR (126 MHz, $CDCl_3$) δ = 171.2, 140.1, 139.2, 139.1, 130.3, 128.9, 127.93, 127.89, 127.4, 126.3, 84.3, 52.2, 25.4, 24.8 ppm;
^{11}B NMR (160 MHz, $CDCl_3$) δ = 31.4 ppm;
HRMS (ESI): Exact mass calculated for $[C_{23}H_{27}BO_4Se+Na]^+$: 481.1060, mass found: 481.1059.

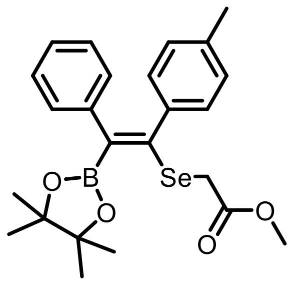

Methyl(*E*)-2-((2-phenyl-2-(4,4,5,5-tetramethyl-1,3,2-dioxaborolan-2-yl)-1-(p-tolyl)vinyl)selanyl)acetate (35), prepared according to general procedure D.
Yield 85%, yellow solid, mp: 97-98 ^oC;
^{1}H NMR (500 MHz, $CDCl_3$) δ = 7.12-7.02 (m, 7 H), 6.97-6.93 (m, 2 H), 3.66 (s, 3 H), 3.00 (s,

2 H), 2.25 (s, 3 H), 1.34 (s, 12 H) ppm;
^{13}C NMR (126 MHz, $CDCl_3$) δ = 171.2, 140.4, 139.3, 137.2, 136.1, 130.2, 128.9, 128.6, 127.9, 126.2, 84.2, 52.2, 25.4, 24.8, 21.2 ppm;
^{11}B NMR (160 MHz, $CDCl_3$) δ = 31.8 ppm;
HRMS (ESI): Exact mass calculated for $[C_{24}H_{29}BO_4Se+H]^+$: 473.1397, mass found: 473.1398.

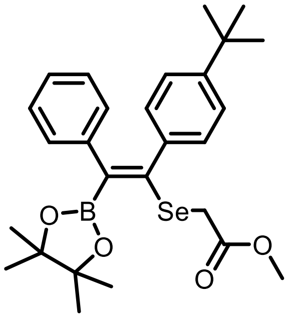

Methyl (*E*)-2-((1-(4-(tert-butyl)phenyl)-2-phenyl-2-(4,4,5,5-tetramethyl-1,3,2-dioxaborolan-2-yl)vinyl)selanyl)acetate (36), prepared according to general procedure D.
Yield 85%, yellow solid, mp: 90-92 ^oC;
^{1}H NMR (500 MHz, $CDCl_3$) δ = 7.16-7.11 (m, 4 H), 7.09-7.01 (m, 5 H), 3.65 (s, 3 H), 3.02 (s, 2 H), 1.35 (s, 12 H), 1.24 (s, 9 H) ppm;
^{13}C NMR (126 MHz, $CDCl_3$) δ = 171.3, 150.4, 140.3, 139.1, 136.1, 129.9, 128.9, 127.9, 126.2, 124.7, 84.2, 52.2, 34.5, 31.2, 25.6, 24.8 ppm;
^{11}B NMR (160 MHz, $CDCl_3$) δ = 31.7 ppm;
HRMS (ESI): Exact mass calculated for $[C_{27}H_{35}BO_4Se+Na]^+$: 537.1686, mass found: 537.1684.

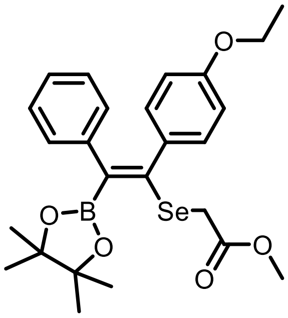

Methyl (*E*)-2-((1-(4-ethoxyphenyl)-2-phenyl-2-(4,4,5,5-tetramethyl-1,3,2-dioxaborolan-2-yl)vinyl)selanyl)acetate (37), prepared according to general procedure D.
Yield 85%, yellow solid, mp: 78-80 ^oC;
^{1}H NMR (500 MHz, $CDCl_3$) δ = 7.17-6.99 (m, 7 H), 6.69-6.62 (m, 2 H), 3.95 (q, *J* = 7.5 Hz, 2 H), 3.66 (s, 3 H), 3.01 (s, 2 H), 1.37 (t, *J* = 7.5 Hz, 3 H), 1.34 (s, 12 H) ppm;
^{13}C NMR (126 MHz, $CDCl_3$) δ = 171.2, 158.2, 140.4, 139.1, 131.7, 131.2, 129.0, 128.0, 126.2, 113.8, 84.2, 63.3, 52.2, 25.5, 24.8, 14.8 ppm;
^{11}B NMR (160 MHz, $CDCl_3$) δ = 32.1 ppm;
HRMS (ESI): Exact mass calculated for $[C_{25}H_{31}BO_5Se+H]^+$: 503.1503, mass found: 503.1502.

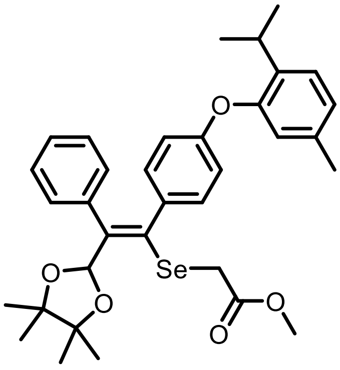

methyl(Z)-2-((1-(4-(2-isopropyl-5-methylphenoxy)phenyl)-2-phenyl-2-(4,4,5,5-tetramethyl-1,3-dioxolan-2-yl)vinyl)selanyl)acetate(38), prepared according to general procedure D.

Yield 78%, yellow solid, mp: 71-72 °C;

^{1}H NMR (500 MHz, $CDCl_3$) δ = 7.19 (d, *J* = 8.0 Hz, 1 H), 7.14-7.03 (m, 6 H), 6.93 (d, *J* = 9.0 Hz, 1 H), 6.85-6.40 (m, 3 H), 3.68 (s, 3 H), 3.14 (hept, *J* = 7.0 Hz, 1 H), 3.06 (s, 2 H), 2.27 (s, 3 H), 1.35 (s, 12 H), 1.14 (d, *J* = 7.0 Hz, 6 H) ppm;

^{13}C NMR (126 MHz, $CDCl_3$) δ = 171.2, 157.8, 153.0, 140.3, 138.8, 137.1, 136.7, 133.0, 131.8, 129.0, 127.9, 126.8, 126.3, 125.2, 120.8, 116.7, 84.3, 52.2, 26.7, 25.5, 24.8, 23.1, 20.9ppm;

^{11}B NMR (160 MHz, $CDCl_3$) δ = 31.7ppm;

HRMS (ESI): Exact mass calculated for $[C_{34}H_{40}O_5Se+H]^+$: 607.2114, mass found: 607.2124.

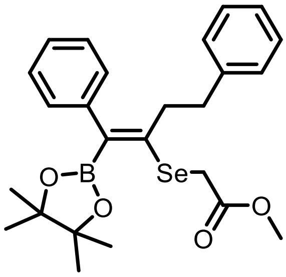

Methyl (*E*)-2-((1,4-diphenyl-1-(4,4,5,5-tetramethyl-1,3,2-dioxaborolan-2-yl)but-1-en-2-yl)selanyl)acetate (39), prepared according to general procedure D.

Yield 47%, yellow semi-solid;

^{1}H NMR (500 MHz, $CDCl_3$) δ = 7.32-7.28 (m, 2 H), 7.25-7.19 (m, 3 H), 7.16-7.10 (m, 3 H), 7.07-7.03 (m, 2 H), 3.75 (s, 3 H), 3.44 (s, 2 H), 2.87-2.80 (m, 2 H), 2.70-2.58 (m, 2 H), 1.28 (s, 12 H) ppm;

^{13}C NMR (126 MHz, $CDCl_3$) δ = 171.4, 141.3, 139.9, 139.6, 131.5, 128.4, 128.3, 127.7, 126.7, 125.9, 84.1, 52.4, 36.4, 35.6, 25.7, 24.7 ppm;

^{11}B NMR (160 MHz, $CDCl_3$) δ = 31.2 ppm;

HRMS (ESI): Exact mass calculated for $[C_{25}H_{31}BO_4Se+H]^+$: 487.1553, mass found: 487.1554.

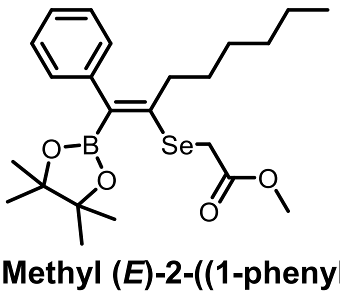

Methyl (*E*)-2-((1-phenyl-1-(4,4,5,5-tetramethyl-1,3,2-dioxaborolan-2-yl)oct-1-en-2-

yl)selanyl)acetate (40), prepared according to general procedure D.
Yield 43%, yellow solid, mp: 95-96 °C;
^{1}H NMR (500 MHz, $CDCl_3$) δ = 7.33-7.28 (m, 2 H), 7.24-7.19 (m, 1 H), 7.17-7.12 (m, 2 H), 3.74 (s, 3 H), 3.39 (s, 2 H), 2.34 (t, *J* = 7.5 Hz, 2 H), 1.54-1.48 (m, 2 H), 1.26 (s, 12 H), 1.23-1.12 (m, 6 H), 0.82 (t, *J* = 7.5 Hz, 3 H) ppm;
^{13}C NMR (126 MHz, $CDCl_3$) δ = 171.4, 140.9, 140.1, 128.2, 127.8, 126.5, 84.0, 52.3, 34.0, 31.5, 29.2, 28.8, 25.6, 24.6, 22.5, 14.1 ppm;
^{11}B NMR (160 MHz, $CDCl_3$) δ = 31.0 ppm;
HRMS (ESI): Exact mass calculated for $[C_{23}H_{35}BO_4Se+H]^+$: 467.1866, mass found: 467.1866.

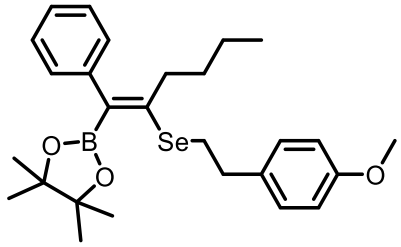

(*E*)-2-(2-((4-methoxyphenethyl)selanyl)-1-phenylhex-1-en-1-yl)-4,4,5,5-tetramethyl-1,3,2-dioxaborolane (41), prepared according to general procedure D.
Yield 48%, yellow oil;
^{1}H NMR (500 MHz, $CDCl_3$) δ = 7.32-7.29 (m, 2 H), 7.23-7.13 (m, 5 H), 6.86-6.82 (m, 2 H), 3.79 (s, 3 H), 2.95 (s, 4 H), 2.29 (t, *J* = 7.5 Hz, 2 H), 1.56-1.48 (m, 2 H), 1.27 (s, 12 H), 1.23-1.19 (m, 2 H), 0.79 (t, *J* = 7.5 Hz, 3 H) ppm;
^{13}C NMR (126 MHz, $CDCl_3$) δ = 158.1, 141.5, 140.6, 133.7, 129.4, 128.2, 128.0, 126.3, 113.9, 83.8, 55.3, 36.1, 33.9, 31.6, 27.7, 24.7, 22.3, 13.9 ppm;
^{11}B NMR (160 MHz, $CDCl_3$) δ = 32.1 ppm;
HRMS (ESI): Exact mass calculated for $[C_{27}H_{37}BO_3Se+H]^+$: 501.2074, mass found: 501.2072.

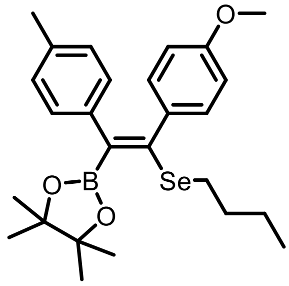

(*E*)-2-(2-(butylselanyl)-2-(4-methoxyphenyl)-1-(p-tolyl)vinyl)-4,4,5,5-tetramethyl-1,3,2-dioxaborolane (42), prepared according to general procedure E.
Yield 75%, colorless solid, mp: 99-101 °C;
^{1}H NMR (500 MHz, $CDCl_3$) δ = 7.14 (d, *J* = 8.5 Hz, 2 H), 6.94-6.85 (m, 4 H), 6.68 (d, *J* = 8.5 Hz, 2 H), 3.75 (s, 3 H), 2.34 (t, *J* = 7.5 Hz, 2 H), 2.21 (s, 3 H), 1.54-1.48 (m, 2 H), 1.36 (s, 12 H), 1.31-1.27 (m, 2 H), 0.80 (t, *J* = 7.5 Hz, 3 H) ppm;
^{13}C NMR (126 MHz, $CDCl_3$) δ = 158.4, 139.3, 137.8, 135.4, 132.4, 131.5, 128.9, 128.7, 113.2, 83.9, 55.1, 32.2, 26.1, 24.8, 22.7, 21.1, 13.5 ppm;
^{11}B NMR (160 MHz, $CDCl_3$) δ = 32.4 ppm;
HRMS (ESI): Exact mass calculated for $[C_{26}H_{35}BO_3Se+H]^+$: 487.1917, mass found: 487.1915.

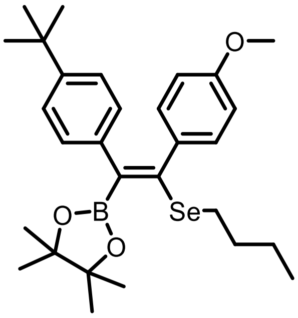

(*E*)-2-(1-(4-(tert-butyl)phenyl)-2-(butylselanyl)-2-(4-methoxyphenyl)vinyl)-4,4,5,5-tetramethyl-1,3,2-dioxaborolane (43), prepared according to general procedure E.
Yield 60%, yellow solid, mp: 51.9-52.6 °C;
^{1}H NMR (500 MHz, $CDCl_3$) δ = 7.17 (d, *J* = 8.5 Hz, 2 H), 7.09 (d, *J* = 8.5 Hz, 2 H), 6.96 (d, *J* = 8.5 Hz, 2 H), 6.70 (d, *J* = 8.5 Hz, 2 H), 3.78 (s, 3 H), 2.36 (t, *J* = 7.5 Hz, 2 H), 1.56-1.52 (m, 2 H), 1.40 (s, 12 H), 1.32-1.28 (m, 2 H), 1.24 (s, 9 H), 0.82 (t, *J* = 7.5 Hz, 3 H) ppm;
^{13}C NMR (126 MHz, $CDCl_3$) δ = 158.4, 148.6, 139.1, 137.5, 132.5, 131.5, 128.6, 124.8, 113.1, 83.9, 55.1, 34.4, 32.2, 31.3, 26.2, 24.9, 22.8, 13.5 ppm;
^{11}B NMR (160 MHz, $CDCl_3$) δ 32.5 ppm;
HRMS (ESI): Exact mass calculated for $[C_{29}H_{41}BO_3Se+H]^+$: 529.2387, mass found: 529.2385.

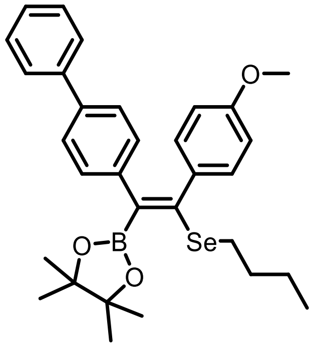

(*E*)-2-(1-([1,1'-biphenyl]-4-yl)-2-(butylselanyl)-2-(4-methoxyphenyl)vinyl)-4,4,5,5-tetramethyl-1,3,2-dioxaborolane (44), prepared according to general procedure E.
Yield 75%, yellow oil;
^{1}H NMR (500 MHz, $CDCl_3$) δ = 7.52 (d, *J* = 7.5 Hz, 2 H), 7.38-7.33 (m, 4 H), 7.29-7.24 (m, 1 H), 7.18 (d, *J* = 8.5 Hz, 2 H), 7.10 (d, *J* = 7.5 Hz, 2 H), 6.69 (d, *J* = 8.5 Hz, 2 H), 3.74 (s, 3 H), 2.36 (t, *J* = 7.5 Hz, 2 H), 1.55-1.50 (m, 2 H), 1.38 (s, 12 H), 1.32-1.27 (m, 2 H), 0.81 (t, *J* = 7.5 Hz, 3 H) ppm;
^{13}C NMR (126 MHz, $CDCl_3$) δ = 158.6, 140.8, 140.6, 139.9, 138.3, 132.2, 131.6, 129.5, 128.7, 127.0, 126.8, 126.5, 113.3, 84.1, 55.1, 32.2, 26.3, 24.9, 22.8, 13.6 ppm;
^{11}B NMR (160 MHz, $CDCl_3$) δ = 31.4 ppm;
HRMS (ESI): Exact mass calculated for $[C_{31}H_{37}BO_3Se+H]^+$: 549.2074, mass found: 549.2071.

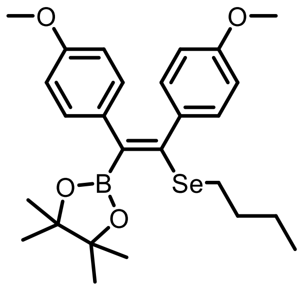

(*E*)-2-(2-(butylselanyl)-1,2-bis(4-methoxyphenyl)vinyl)-4,4,5,5-tetramethyl-1,3,2-dioxaborolane (45), prepared according to general procedure E.
Yield 70%, brown oil;
^{1}H NMR (500 MHz, $CDCl_3$) δ = 7.15 (d, *J* = 9.0 Hz, 2 H), 6.96 (d, *J* = 9.0 Hz, 2 H), 6.68 (d, *J* = 9.0 Hz, 2 H), 6.62 (d, *J* = 9.0 Hz, 2 H), 3.74 (s, 3 H), 3.70 (s, 3 H), 2.34 (t, *J* = 7.5 Hz, 2 H), 1.53-1.49 (m, 2 H), 1.36 (s, 12 H), 1.30-1.25 (m, 2 H), 0.80 (t, *J* = 7.5 Hz, 3 H) ppm;
^{13}C NMR (126 MHz, $CDCl_3$) δ = 158.4, 157.7, 138.6, 133.2, 132.4, 131.5, 130.2, 113.4, 113.2, 83.9, 55.1, 55.0, 32.2, 26.1, 24.8, 22.7, 13.5 ppm;
^{11}B NMR (160 MHz, $CDCl_3$) δ = 33.0 ppm;
HRMS (ESI): Exact mass calculated for $[C_{26}H_{35}BO_4Se+H]^+$: 503.1866, mass found: 503.1865.

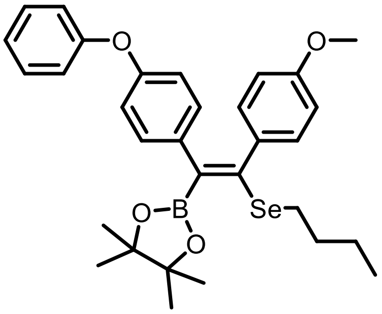

(*E*)-2-(2-(butylselanyl)-2-(4-methoxyphenyl)-1-(4-phenoxyphenyl)vinyl)-4,4,5,5-tetramethyl-1,3,2-dioxaborolane (46), prepared according to general procedure E.
Yield 76%, yellow solid, mp: 64-66 °C;
^{1}H NMR (500 MHz, $CDCl_3$) δ = 7.32-7.27 (m, 2 H), 7.16 (d, *J* = 8.5 Hz, 2 H), 7.08-7.04 (m, 1 H), 7.00 (d, *J* = 8.5 Hz, 2 H), 6.96-6.92 (m, 2 H), 6.73 – 6.69 (m, 4 H), 3.76 (s, 3 H), 2.35 (t, *J* = 7.5 Hz, 2 H), 1.54-1.49 (m, 2 H), 1.38 (s, 12 H), 1.31-1.27 (m, 2 H), 0.81 (t, *J* = 7.5 Hz, 3 H) ppm;
^{13}C NMR (126 MHz, $CDCl_3$) δ = 158.5, 157.1, 155.2, 140.0, 135.9, 132.1, 131.5, 130.5, 129.6, 123.1, 118.9, 118.2, 113.3, 84.0, 55.1, 32.2, 26.2, 24.9, 22.7, 13.5;
^{11}B NMR (160 MHz, $CDCl_3$) δ = 32.5 ppm;
HRMS (ESI): Exact mass calculated for $[C_{31}H_{37}BO_4Se+H]^+$: 565.2023, mass found: 565.2023.

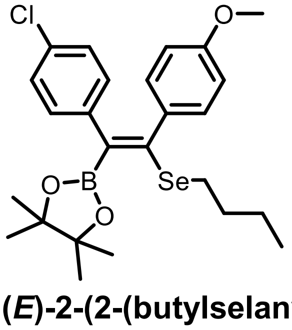

(*E*)-2-(2-(butylselanyl)-1-(4-chlorophenyl)-2-(4-methoxyphenyl)vinyl)-4,4,5,5-

tetramethyl-1,3,2-dioxaborolane (47), prepared according to general procedure F.
Yield 75%, yellow oil;
^{1}H NMR (500 MHz, $CDCl_3$) δ = 7.10 (d, *J* = 8.5 Hz, 2 H), 7.04 (d, *J* = 8.5 Hz, 2 H), 6.95 (d, *J* = 8.5 Hz, 2 H), 6.69 (d, *J* =8.5 Hz, 2 H), 3.76 (s, 3 H), 2.33 (t, *J* = 7.5 Hz, 2 H), 1.52-1.47 (m, 2 H), 1.36 (s, 12 H), 1.29-1.26 (m, 2 H), 0.79 (t, *J* = 7.5 Hz, 3 H) ppm;
^{13}C NMR (126 MHz, $CDCl_3$) δ = 158.6, 141.7, 139.4, 131.7, 131.5, 131.4, 130.4, 128.1, 113.3, 84.1, 55.1, 32.2, 26.3, 24.8, 22.7, 13.5 ppm;
^{11}B NMR (160 MHz, $CDCl_3$) δ = 31.9 ppm;
HRMS (ESI): Exact mass calculated for $[C_{25}H_{32}BClO_3Se+H]^+$: 507.1371, mass found: 507.1369.

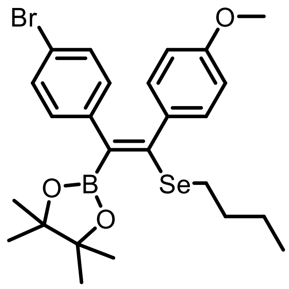

(*E*)-2-(1-(4-bromophenyl)-2-(butylselanyl)-2-(4-methoxyphenyl)vinyl)-4,4,5,5-tetramethyl-1,3,2-dioxaborolane (48) , prepared according to general procedure F.
Yield 78%, yellow oil;
^{1}H NMR (500 MHz, $CDCl_3$) δ = 7.19 (d, *J* = 8.5 Hz, 2 H), 7.10 (d, *J* = 8.5 Hz, 2 H), 6.89 (d, *J* = 8.5 Hz, 2 H), 6.69 (d, *J* = 8.5 Hz, 2 H), 3.76 (s, 3 H), 2.32 (t, *J* = 7.5 Hz, 2 H), 1.52-1.47 (m, 2 H), 1.35 (s, 12 H), 1.29-1.25 (m, 2 H), 0.79 (t, *J* = 7.5 Hz, 3 H) ppm;
^{13}C NMR (126 MHz, $CDCl_3$) δ = 158.7, 141.8, 139.9, 131.6, 131.4, 131.0, 130.8, 119.8, 113.4, 84.1, 55.2, 32.2, 26.3, 24.8, 22.7, 13.5 ppm;
^{11}B NMR (160 MHz, $CDCl_3$) δ = 31.6 ppm;
HRMS (ESI): Exact mass calculated for $[C_{25}H_{32}BBrO_3Se+H]^+$: 551.0866, mass found: 551.0882.

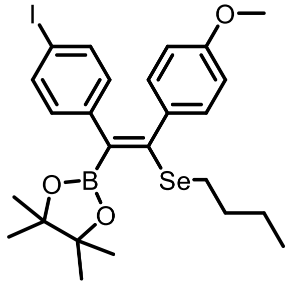

(*E*)-2-(2-(butylselanyl)-1-(4-iodophenyl)-2-(4-methoxyphenyl)vinyl)-4,4,5,5-tetramethyl-1,3,2-dioxaborolane (49) , prepared according to general procedure F.
Yield 64%, yellow solid, mp: 103-104 ^{o}C;
^{1}H NMR (500 MHz, $CDCl_3$) δ = 7.42 (d, *J* = 8.5 Hz, 2 H), 7.13 (d, *J* = 9.0 Hz, 2 H), 6.79 (d, *J* = 8.5 Hz, 2 H), 6.71 (d, *J* = 9.0 Hz, 2 H), 3.78 (s, 3 H), 2.35 (t, *J* = 7.5 Hz, 2 H), 1.54-1.49 (m, 2 H), 1.38 (s, 1 2H), 1.31-1.27 (m, 2 H), 0.81 (t, *J* = 7.5 Hz, 3 H) ppm;
^{13}C NMR (126 MHz, $CDCl_3$) δ = 158.7, 141.9, 140.5, 137.0, 131.7, 131.4, 131.0, 113.4, 91.4, 84.1, 55.2, 32.2, 26.3, 24.8, 22.7, 13.5 ppm;
^{11}B NMR (160 MHz, $CDCl_3$) δ = 33.4 ppm;

HRMS (ESI): Exact mass calculated for $[C_{25}H_{32}BIO_3Se+H]^+$: 599.0727, mass found: 599.0726.

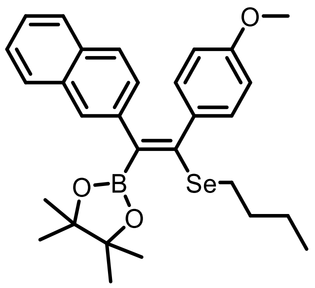

(*E*)-2-(2-(butylselanyl)-2-(4-methoxyphenyl)-1-(naphthalen-2-yl)vinyl)-4,4,5,5-tetramethyl-1,3,2-dioxaborolane (50), prepared according to general procedure E.
Yield 78%, brown oil;
^{1}H NMR (500 MHz, CD_2Cl_2) δ = 7.69-7.65 (m, 3 H), 7.49 (d, *J* = 9.0 Hz, 1 H), 7.39-7.34 (m, 2 H), 7.21-7.17 (m, 2 H), 7.06-7.04 (m, 1 H), 6.65 (d, *J* = 9.0 Hz, 2 H), 3.72 (s, 3 H), 2.38 (t, *J* = 7.5 Hz, 2 H), 1.57-1.52 (m, 2 H), 1.39 (s, 12 H), 1.34-1.29 (m, 2 H), 0.82 (t, *J* = 7.5 Hz, 3 H) ppm;
^{13}C NMR (126 MHz, $CDCl_3$) δ = 158.6, 140.8, 138.6, 133.4, 132.0, 131.8, 131.7, 127.89, 127.88, 127.6, 127.5, 127.2, 125.6, 125.4, 113.3, 84.1, 55.1, 32.2, 26.3, 24.9, 22.7, 13.5 ppm;
^{11}B NMR (160 MHz, $CDCl_3$) δ = 31.3 ppm;
HRMS (ESI): Exact mass calculated for $[C_{29}H_{35}BO_3Se+H]^+$: 523.1917, mass found: 523.1918.

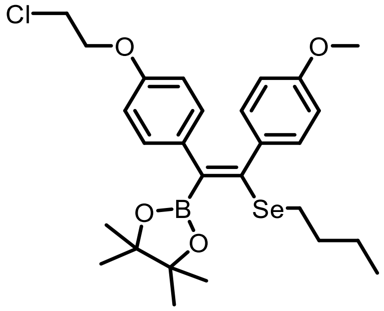

(*E*)-2-(2-(butylselanyl)-1-(4-(2-chloroethoxy)phenyl)-2-(4-methoxyphenyl)vinyl)-4,4,5,5-tetramethyl-1,3,2-dioxaborolane (51), prepared according to general procedure E.
Yield 68%, yellow oil;
^{1}H NMR (500 MHz, $CDCl_3$) δ = 7.14 (d, *J* = 8.8 Hz, 2 H), 6.96 (d, *J* = 8.8 Hz, 2 H), 6.68 (d, *J* = 8.8 Hz, 2 H), 6.63 (d, *J* = 8.8 Hz, 2 H), 4.13 (t, *J* = 6.0 Hz, 2 H), 3.76-3.73 (m, 5 H), 2.33 (t, *J* = 7.5 Hz, 2 H), 1.53-1.48 (m, 2 H), 1.36 (s, 12 H), 1.30-1.26 (m, 2 H), 0.80 (t, *J* = 7.5 Hz, 3 H) ppm;
^{13}C NMR (126 MHz, $CDCl_3$) δ = 158.4, 156.2, 139.2, 134.0, 132.2, 131.5, 130.3, 114.2, 113.3, 84.0, 67.8, 55.1, 41.9, 32.2, 26.1, 24.8, 22.7, 13.5 ppm;
^{11}B NMR (160 MHz, $CDCl_3$) δ = 32.8 ppm;
HRMS (ESI): Exact mass calculated for $[C_{27}H_{36}BClO_4Se+H]^+$: 551.1633, mass found: 551.1634.

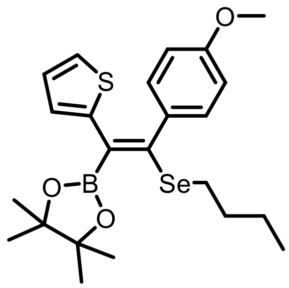

(*E*)-2-(2-(butylselanyl)-2-(4-methoxyphenyl)-1-(thiophen-2-yl)vinyl)-4,4,5,5-tetramethyl-1,3,2-dioxaborolane (52), prepared according to general procedure E.
Yield 60%, yellow oil;
^{1}H NMR (500 MHz, $CDCl_3$) δ = 7.21 (d, *J* = 8.5 Hz, 2 H), 6.99-6.95 (m, 1 H), 6.85 (d, *J* = 8.5 Hz, 2 H), 6.82-6.76 (m, 2 H), 3.82 (s, 3 H), 2.32 (t, *J* = 7.5 Hz, 2 H), 1.54-1.49 (m, 2 H), 1.43 (s, 12 H), 1.31-1.26 (m, 2 H), 0.81 (t, *J* = 7.5 Hz, 3 H) ppm;
^{13}C NMR (126 MHz, $CDCl_3$) δ = 159.3, 143.0, 138.5, 132.3, 131.1, 127.0, 125.8, 125.2, 113.9, 84.4, 55.2, 32.4, 26.4, 25.1, 22.8, 13.6 ppm;
^{11}B NMR (160 MHz, $CDCl_3$) δ = 31.1 ppm;
HRMS (ESI): Exact mass calculated for $[C_{23}H_{31}BO_3SSe+H]^+$: 479.1325, mass found: 479.1326.

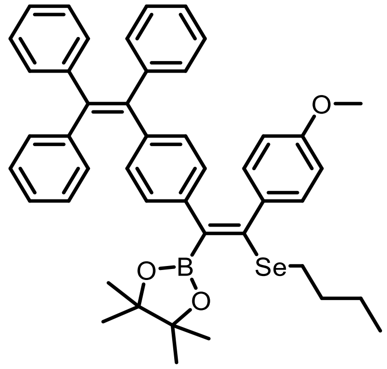

(*E*)-2-(2-(butylselanyl)-2-(4-methoxyphenyl)-1-(4-(1,2,2-triphenylvinyl)phenyl)vinyl)-4,4,5,5-tetramethyl-1,3,2-dioxaborolane (53), prepared according to general procedure E.
Yield 66%, colorless solid, mp: 156.9-157.8 ^{o}C;
^{1}H NMR (500 MHz, $CDCl_3$) δ = 7.11 (d, *J* = 8.5 Hz, 2 H), 7.07-7.06 (m, 9 H), 6.99-6.94 (m, 6 H), 6.78-6.71 (m, 4 H), 6.68 (d, *J* = 8.5 Hz, 2 H), 3.79 (s, 3 H), 2.33 (t, *J* = 7.5 Hz, 2 H), 1.53-1.47 (m, 2 H), 1.34 (s, 12 H), 1.30-1.25 (m, 2 H), 0.80 (t, *J* = 7.5 Hz, 3 H) ppm;
^{13}C NMR (126 MHz, $CDCl_3$) δ = 158.5, 143.9, 143.8, 143.5, 141.2, 140.9, 140.7, 139.9, 138.9, 132.1, 131.6, 131.4, 131.35, 131.34, 130.9, 128.4, 127.6, 127.5, 126.3, 126.28, 126.25, 113.1, 83.9, 55.1, 32.2, 26.2, 24.8, 22.7, 13.5 ppm;
^{11}B NMR (160 MHz, $CDCl_3$) δ = 32.2 ppm;
HRMS (ESI): Exact mass calculated for $[C_{45}H_{47}BO_3Se+H]^+$: 727.2856, mass found: 727.2853.

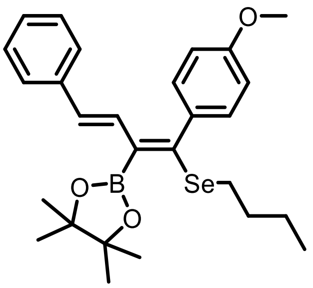

2-((1*E*,3*E*)-1-(butylselanyl)-1-(4-methoxyphenyl)-4-phenylbuta-1,3-dien-2-yl)-4,4,5,5-tetramethyl-1,3,2-dioxaborolane (54), prepared according to general procedure E.
Yield 64%, colorless oil;
^{1}H NMR (500 MHz, $CDCl_3$) δ = 7.32 (d, *J* = 8.5 Hz, 2 H), 7.24-7.20 (m, 4 H), 7.17-7.11 (m, 1 H), 6.89 (d, *J* = 8.5 Hz, 2 H), 6.81 (d, *J* = 16.0 Hz, 1 H), 6.57 (d, *J* = 16.0 Hz, 1 H), 3.85 (s, 3 H), 2.31 (t, *J* = 7.5 Hz, 2 H), 1.53-1.47 (m, 2 H), 1.47 (s, 12 H), 1.29-1.25 (m, 2 H), 0.79 (t, *J* = 7.5 Hz, 3 H) ppm;
^{13}C NMR (126 MHz, $CDCl_3$) δ = 159.0, 142.2, 137.9, 132.1, 131.4, 130.9, 130.0, 128.4, 127.1, 126.3, 113.4, 84.2, 55.3, 32.3, 26.7, 25.1, 22.7, 13.5 ppm;
^{11}B NMR (160 MHz, $CDCl_3$) δ = 32.5 ppm;
HRMS (ESI): Exact mass calculated for $[C_{27}H_{35}BO_3Se+H]^+$: 499.1917, mass found: 499.1918.

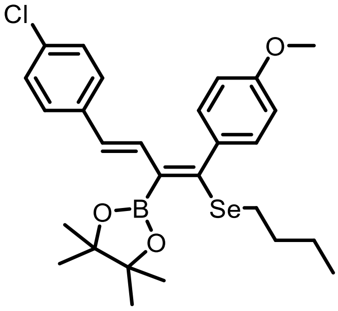

2-((1*E*,3*E*)-1-(butylselanyl)-4-(4-chlorophenyl)-1-(4-methoxyphenyl)buta-1,3-dien-2-yl)-4,4,5,5-tetramethyl-1,3,2-dioxaborolane (55), prepared according to general procedure E.
Yield 64%, yellow semi-solid;
^{1}H NMR (500 MHz, $CDCl_3$) δ = 7.28 (d, *J* = 8.5 Hz, 2 H), 7.15 (d, *J* = 8.5 Hz, 2 H), 7.09 (d, *J* = 8.5 Hz, 2 H), 6.87 (d, *J* = 8.5 Hz, 2 H), 6.74 (d, *J* = 16 Hz, 1 H), 6.48 (d, *J* = 16 Hz, 1 H), 3.82 (s, 3 H), 2.28 (t, *J* = 7.5 Hz, 2 H), 1.48-1.44 (m, 2 H), 1.43 (s, 12 H), 1.28-1.22 (m, 2 H), 0.76 (t, *J* = 7.5 Hz, 3 H) ppm;
^{13}C NMR (126 MHz, $CDCl_3$) δ = 159.1, 143.3, 136.5, 132.5, 132.0, 131.3, 130.6, 129.4, 128.5, 127.4, 113.4, , 84.3, 55.3, 32.3, 26.8, 25.1, 22.6, 13.5 ppm;
^{11}B NMR (160 MHz, $CDCl_3$) δ = 32.8 ppm;
HRMS (ESI): Exact mass calculated for $[C_{27}H_{34}BClO_3Se+H]^+$: 533.1528, mass found: 533.1528.

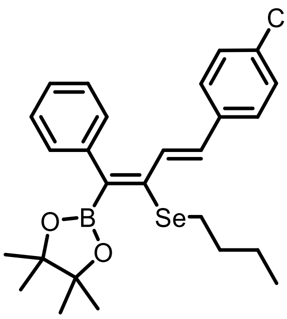

2-((1*E*,3*E*)-2-(butylselanyl)-4-(4-chlorophenyl)-1-phenylbuta-1,3-dien-1-yl)-4,4,5,5-tetramethyl-1,3,2-dioxaborolane (56), prepared according to general procedure D.
Yield 75%, yellow oil;
^{1}H NMR (500 MHz, $CDCl_3$) δ = 7.37-7.32 (m, 2 H), 7.30-7.21 (m, 7 H), 7.15 (d, *J* = 15.5 Hz, 1 H), 6.89 (d, *J* = 15.5 Hz, 1 H), 2.78 (t, *J* = 7.5 Hz, 2 H), 1.74-1.65 (m, 2 H), 1.48-1.39 (m, 2 H), 1.34 (s, 12 H), 0.90 (t, *J* = 7.5 Hz, 3 H) ppm;
^{13}C NMR (126 MHz, $CDCl_3$) δ = 139.9, 135.8, 135.5, 134.2, 133.3, 128.9, 128.8, 128.3, 128.1, 127.1, 126.9, 84.1, 32.3, 28.2, 24.8, 23.0, 13.7 ppm;
^{11}B NMR (160 MHz, $CDCl_3$) δ = 31.6 ppm
HRMS (ESI): Exact mass calculated for $[C_{26}H_{32}BClO_2Se+H]^+$: 503.1422, mass found: 503.1426.

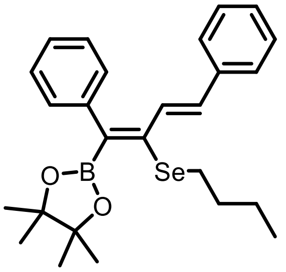

2-((1*E*,3*E*)-2-(butylselanyl)-1,4-diphenylbuta-1,3-dien-1-yl)-4,4,5,5-tetramethyl-1,3,2-dioxaborolane (57), prepared according to general procedure D.
Yield 80%, yellow oil;
^{1}H NMR (500 MHz, $CDCl_3$) δ = 7.36-7.32 (m, 4 H), 7.30-7.25 (m, 5 H), 7.22-7.18 (m, 2 H), 6.93 (d, *J* = 15.5 Hz, 1 H), 2.79 (t, *J* = 7.5 Hz, 2 H), 1.74-1.67 (m, 2 H), 1.45-1.40 (m, 2 H), 1.34 (s, 12 H), 0.90 (t, *J* = 7.5 Hz, 3 H) ppm;
^{13}C NMR (126 MHz, $CDCl_3$) δ = 140.0, 137.3, 135.9, 135.5, 129.0, 128.6, 128.2, 127.7, 127.01, 126.97, 126.3, 84.0, 32.4, 28.1, 24.8, 23.0, 13.6 ppm;
^{11}B NMR (160 MHz, $CDCl_3$) δ = 31.1 ppm;
HRMS (ESI): Exact mass calculated for $[C_{26}H_{33}BO_2Se+H]^+$: 469.1812, mass found: 469.1812.

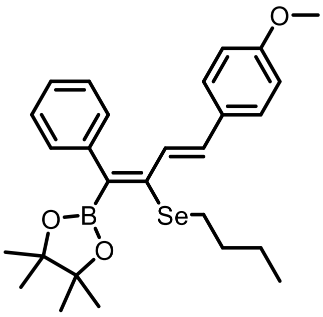

2-((1*E*,3*E*)-2-(butylselanyl)-4-(4-methoxyphenyl)-1-phenylbuta-1,3-dien-1-yl)-4,4,5,5-tetramethyl-1,3,2-dioxaborolane (58), prepared according to general procedure D.
Yield 63%, yellow oil;
^{1}H NMR (500 MHz, $CDCl_3$) δ = 7.29-7.16 (m, 7 H), 7.08 (d, *J* = 15.5 Hz, 1 H), 6.79-6.69 (m, 3 H), 3.72 (s, 3 H), 2.71 (t, *J* = 7.5 Hz, 2 H), 1.75-1.58 (m, 2 H), 1.43-1.33 (m, 2 H), 1.27 (s, 12 H), 0.83 (t, *J* = 7.5 Hz, 3 H) ppm;
^{13}C NMR (126 MHz, $CDCl_3$) δ = 159.4, 140.2, 136.2, 135.1, 130.1, 129.0, 128.3, 128.2, 126.8, 124.3, 114.1, 84.0, 55.3, 32.4, 28.1, 24.8, 23.0, 13.6 ppm;
^{11}B NMR (160 MHz, $CDCl_3$) δ = 29.3 ppm;
HRMS (ESI): Exact mass calculated for $[C_{27}H_{35}BO_3Se+H]^+$: 499.1917, mass found: 499.1918.

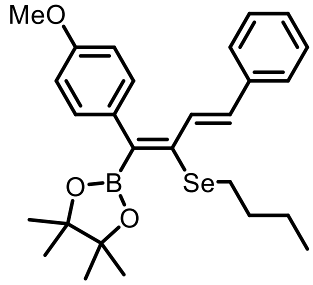

2-((1*E*,3*E*)-2-(butylselanyl)-1-(4-methoxyphenyl)-4-phenylbuta-1,3-dien-1-yl)-4,4,5,5-tetramethyl-1,3,2-dioxaborolane (59), prepared according to general procedure E.
Yield 89%, pale yellow oil;
^{1}H NMR (500 MHz, $CDCl_3$) δ = 7.35 (d, *J* = 7.5 Hz, 2 H), 7.30-7.16 (m, 6 H), 6.95 (d, *J* = 15.0 Hz, 1 H), 6.88 (d, *J* = 7.5 Hz, 2 H), 3.82 (s, 3 H), 2.77 (t, *J* = 7.5 Hz, 2 H), 1.72-1.65 (m, 2 H), 1.44-1.39 (m, 2 H), 1.34 (s, 12 H), 0.90 (t, *J* = 7.5 Hz, 3 H) ppm;
^{13}C NMR (126 MHz, $CDCl_3$) δ = 158.7, 137.4, 135.1, 134.9, 132.6, 130.3, 128.6, 127.6, 126.9, 126.5, 113.7, 84.0, 55.2, 32.4, 27.9, 24.8, 23.0, 13.6 ppm;
^{11}B NMR (160 MHz, $CDCl_3$) δ = 30.6 ppm;
HRMS (ESI): Exact mass calculated for $[C_{27}H_{35}BO_3Se+H]^+$: 499.1917, mass found: 499.1918.

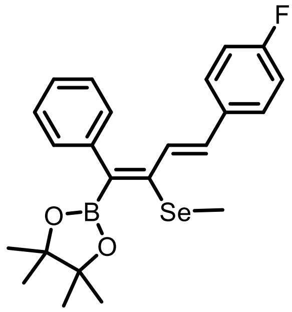

2-((1*E*,3*E*)-4-(4-fluorophenyl)-2-(methylselanyl)-1-phenylbuta-1,3-dien-1-yl)-4,4,5,5-tetramethyl-1,3,2-dioxaborolane (60), prepared according to general procedure D.
Yield 84%, colorless solid, mp: 121-122 ^{o}C;
^{1}H NMR (500 MHz, $CDCl_3$) δ = 7.37-7.25 (m, 7 H), 7.15 (d, *J* = 15.5 Hz, 1 H), 6.96 (t, *J* = 8.5 Hz, 2 H), 6.79 (d, *J* = 15.5 Hz, 1 H), 2.20 (s, 3 H), 1.35 (s, 12 H) ppm;
^{13}C NMR (126 MHz, $CDCl_3$) δ = 162.4 (d, *J* = 252 Hz), 139.8, 136.5, 134.3, 133.4 (d, *J* = 2.5 Hz), 128.9, 128.5 (d, *J* = 7.6 Hz), 128.3, 127.1, 125.3 (d, *J* = 2.0 Hz), 115.6 (d, *J* = 21.4 Hz), 84.1, 24.7, 8.6 ppm;
^{11}B NMR (160 MHz, $CDCl_3$) δ = 28.2 ppm;

HRMS (ESI): Exact mass calculated for $[C_{23}H_{26}BFO_2Se+H]^+$: 445.1248, mass found: 445.1249.

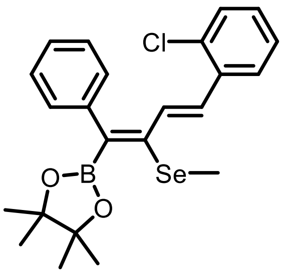

2-((1*E*,3*E*)-4-(2-chlorophenyl)-2-(methylselanyl)-1-phenylbuta-1,3-dien-1-yl)-4,4,5,5-tetramethyl-1,3,2-dioxaborolane (61), prepared according to general procedure D.
Yield 68%, pale yellow solid, mp: 107-108 °C;
^{1}H NMR (500 MHz, $CDCl_3$) δ = 7.59 (d, *J* = 15.5 Hz, 1 H), 7.38-7.32 (m, 4 H), 7.30-7.25 (m, 3 H), 7.13 (m, 2 H), 6.82 (d, *J* = 15.5 Hz, 1 H), 2.24 (s, 3 H), 1.35 (s, 12 H) ppm;
^{13}C NMR (126 MHz, $CDCl_3$) δ = 139.7, 136.7, 135.4, 133.7, 131.9, 129.8, 128.9, 128.6, 128.3, 127.9, 127.1, 126.9, 126.8, 84.2, 24.7, 8.4 ppm;
^{11}B NMR (160 MHz, $CDCl_3$) δ = 31.2 ppm;
HRMS (ESI): Exact mass calculated for $[C_{23}H_{26}BClO_2Se+H]^+$: 461.0952, mass found: 461.0953.

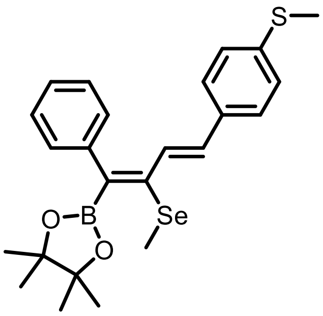

4,4,5,5-Tetramethyl-2-((1*E*,3*E*)-2-(methylselanyl)-4-(4-(methylthio)phenyl)-1-phenylbuta-1,3-dien-1-yl)-1,3,2-dioxaborolane (62), prepared according to general procedure D.
Yield 83%, pale yellow solid, mp: 118-119 °C;
^{1}H NMR (500 MHz, $CDCl_3$) δ = 7.34 (t, *J* = 7.5 Hz, 2 H), 7.29-7.24 (m, 5 H), 7.18-7.11 (m, 3 H), 6.84 (d, *J* = 15.5 Hz, 1 H), 2.46 (s, 3 H), 2.19 (s, 3 H), 1.35 (s, 12 H) ppm;
^{13}C NMR (126 MHz, $CDCl_3$) δ = 139.8, 138.2, 136.7, 134.9, 134.2, 128.9, 128.2, 127.4, 127.0, 126.6, 125.0, 84.1, 24.7, 15.8, 8.6 ppm;
^{11}B NMR (160 MHz, $CDCl_3$) δ = 29.7 ppm;
HRMS (ESI): Exact mass calculated for $[C_{24}H_{29}BO_2SSe+H]^+$: 473.1219, mass found: 473.1220.

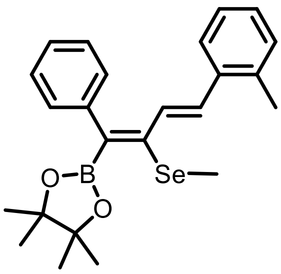

4,4,5,5-Tetramethyl-2-((1*E*,3*E*)-2-(methylselanyl)-1-phenyl-4-(o-tolyl)buta-1,3-dien-1-yl)-1,3,2-dioxaborolane (63), prepared according to general procedure D.
Yield 71%, yellow oil;
^{1}H NMR (500 MHz, $CDCl_3$) δ = 7.42 (d, *J* = 15.0 Hz, 1 H), 7.35-7.31 (m, 2 H), 7.31-7.27 (m, 3 H), 7.27-7.23 (m, 1 H), 7.16-7.06 (m, 3 H), 6.76 (d, *J* = 15.0 Hz, 1 H), 2.39 (s, 3 H), 2.21 (s, 3 H), 1.35 (s, 12 H) ppm;
^{13}C NMR (126 MHz, $CDCl_3$) δ = 139.9, 137.3, 136.2, 136.1, 133.6, 130.4, 128.9, 128.2, 127.7, 127.0, 126.5, 126.1, 125.7, 84.1, 24.7, 20.0, 8.4 ppm;
^{11}B NMR (160 MHz, $CDCl_3$) δ = 31.3 ppm;
HRMS (ESI): Exact mass calculated for $[C_{24}H_{29}BO_2Se+H]^+$: 441.1499, mass found: 441.1499.

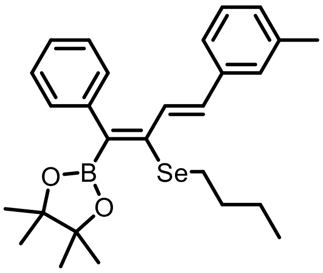

2-((1*E*,3*E*)-2-(butylselanyl)-1-phenyl-4-(m-tolyl)buta-1,3-dien-1-yl)-4,4,5,5-tetramethyl-1,3,2-dioxaborolane (64), prepared according to general procedure D.
Yield 73%, pale yellow oil;
^{1}H NMR (500 MHz, $CDCl_3$) δ = 7.36-7.32 (m, 2 H), 7.31-7.28 (m, 2 H), 7.28-7.24 (m, 1 H), 7.20-7.14 (m, 4 H), 7.03 (d, *J* = 6.5 Hz, 1 H), 6.91 (d, *J* = 15.5 Hz, 1 H), 2.78 (t, *J* = 7.5 Hz, 2 H), 2.31 (s, 3 H), 1.73-1.65 (m, 2 H), 1.46-1.38 (m, 2 H), 1.34 (s, 12 H), 0.90 (t, *J* = 7.5 Hz, 3 H) ppm;
^{13}C NMR (126 MHz, $CDCl_3$) δ = 140.0, 138.2, 137.2, 136.0, 135.8, 129.0, 128.6,128.5, 128.2, 127.9, 127.0, 126.0, 124.1, 84.0, 32.4, 28.0, 24.8, 23.0, 21.4, 13.7 ppm;
^{11}B NMR (160 MHz, $CDCl_3$) δ = 31.9 ppm;
HRMS (ESI): Exact mass calculated for $[C_{27}H_{35}BO_2Se+H]^+$: 483.1968, mass found: 483.1969.

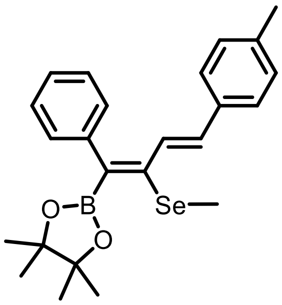

4,4,5,5-Tetramethyl-2-((1*E*,3*E*)-2-(methylselanyl)-1-phenyl-4-(p-tolyl)buta-1,3-dien-1-

yl)-1,3,2-dioxaborolane (65), prepared according to general procedure D.
Yield 66%, pale yellow solid, mp: 112-113 °C;
^{1}H NMR (500 MHz, $CDCl_3$) δ = 7.36-7.31 (m, 2 H), 7.30-7.27 (m, 2 H), 7.26-7.23 (m, 3 H), 7.15 (d, *J* = 15.5 Hz, 1 H), 7.12-7.06 (m, 2 H), 6.83 (d, *J* = 15.5 Hz, 1 H), 2.32 (s, 3 H), 2.19 (s, 3 H), 1.35 (s, 12 H) ppm;
^{13}C NMR (126 MHz, $CDCl_3$) δ = 139.9, 137.8, 136.9, 135.6, 134.4, 129.3, 129.0, 128.2, 126.9, 124.6, 84.1, 24.7, 21.2, 8.5 ppm;
^{11}B NMR (160 MHz, $CDCl_3$) δ = 30.9 ppm;
HRMS (ESI): Exact mass calculated for $[C_{24}H_{29}BO_2Se+H]^+$: 441.1499, mass found: 441.1499.

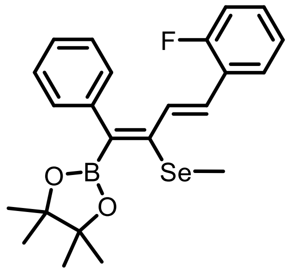

2-((1*E*,3*E*)-4-(2-fluorophenyl)-2-(methylselanyl)-1-phenylbuta-1,3-dien-1-yl)-4,4,5,5-tetramethyl-1,3,2-dioxaborolane (66), prepared according to general procedure D.
Yield 86%, colorless solid, mp: 90.9-91.7 °C;
^{1}H NMR (500 MHz, $CDCl_3$) δ = 7.38-7.31 (m, 4 H), 7.30-7.25 (m, 3 H), 7.17 (m, 1 H), 7.04-6.99 (m, 2 H), 6.94 (d, *J* = 15.5 Hz, 1 H), 2.21 (s, 3 H), 1.35 (s, 12 H) ppm;
^{13}C NMR (126 MHz, $CDCl_3$) δ = 160.4 (d, *J* = 252 Hz), 139.7, 136.8, 129.0, 128.9, 128.3, 127.8 (d, *J* = 3.8 Hz), 127.7 (d, *J* = 4.4 Hz), 127.5 (d, *J* = 3.4 Hz), 127.1, 125.2 (d, *J* = 11.3 Hz), 124.1 (d, *J* = 3.4 Hz), 115.8 (d, *J* = 22.7 Hz), 84.2, 24.7, 8.5 ppm;
^{11}B NMR (160 MHz, $CDCl_3$) δ = 29.6 ppm;
HRMS (ESI): Exact mass calculated for $[C_{23}H_{26}BFO_2Se+H]^+$: 445.1248, mass found: 445.1248.

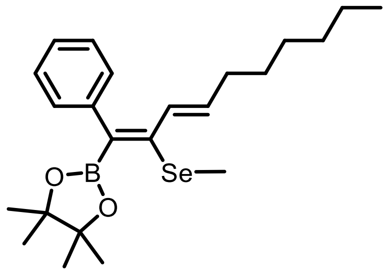

4,4,5,5-Tetramethyl-2-((1*E*,3*E*)-2-(methylselanyl)-1-phenyldeca-1,3-dien-1-yl)-1,3,2-dioxaborolane (67), prepared according to general procedure D.
Yield 63%, colorless oil;
^{1}H NMR (500 MHz, $CDCl_3$) δ = 7.33-7.26 (m, 2 H), 7.25-7.15 (m, 3 H), 6.27 (d, *J* = 15.5 Hz, 1 H), 6.08 (d, *J* = 15.5 Hz, 1 H), 2.14 (s, 3 H), 2.12-2.06 (m, 2 H), 1.38-1.34 (m, 2 H), 1.32 (s, 12 H), 1.28-1.21 (m, 6 H), 0.86 (t, *J* = 7.5 Hz, 3 H) ppm;
^{13}C NMR (126 MHz, $CDCl_3$) δ = 140.0, 139.0, 137.2, 128.8, 128.1, 126.6, 126.5, 83.9, 32.5, 31.7, 29.2, 28.8, 24.7, 22.6, 14.1, 8.2 ppm;
^{11}B NMR (160 MHz, $CDCl_3$) δ = 30.9 ppm;
HRMS (ESI): Exact mass calculated for $[C_{23}H_{35}BO_2Se+H]^+$: 435.1968, mass found: 435.1969

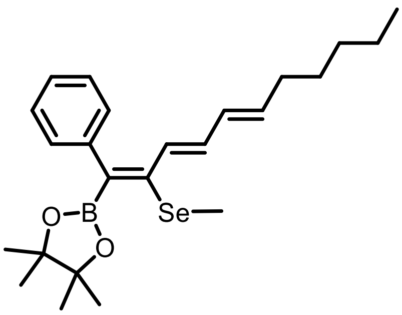

4,4,5,5-Tetramethyl-2-((1*E*,3*E*,5*E*)-2-(methylselanyl)-1-phenylundeca-1,3,5-trien-1-yl)-1,3,2-dioxaborolane (68), prepared according to general procedure D.
Yield 78%, pale yellow oil;
^{1}H NMR (500 MHz, $CDCl_3$) δ = 7.34-7.26 (m, 2 H), 7.25-7.20 (m, 3 H), 6.81 (dd, *J* = 15.0, 10.0 Hz, 1 H), 6.21 (d, *J* = 15.0 Hz, 1 H), 6.09 (dd, *J* = 15.0, 10.0 Hz, 1 H), 5.93-5.77 (m, 1 H), 2.16 (s, 3 H), 2.08 (q, *J* = 7.5 Hz, 2 H), 1.41-1.36 (m, 2 H), 1.32 (s, 12 H), 1.30-1.25 (m, 4 H), 0.88 (t, *J* = 7.5 Hz, 3 H) ppm;
^{13}C NMR (126 MHz, $CDCl_3$) δ =139.9, 137.4, 136.8, 136.7, 130.2, 128.8, 128.2, 127.0, 126.8, 84.0, 32.9, 31.5, 28.9, 24.7, 22.5, 14.0, 8.6 ppm;
^{11}B NMR (160 MHz, $CDCl_3$) δ = 31.4 ppm;
HRMS (ESI): Exact mass calculated for $[C_{24}H_{35}BO_2Se+H]^+$: 447.1968, mass found: 447.1968

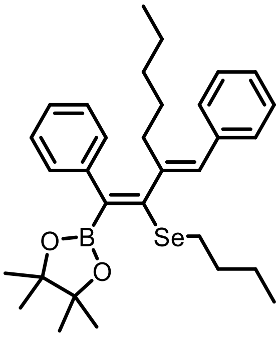

2-((*E*)-3-((*E*)-benzylidene)-2-(butylselanyl)-1-phenyloct-1-en-1-yl)-4,4,5,5-tetramethyl-1,3,2-dioxaborolane (69), prepared according to general procedure D.
Yield 37%, yellow oil;
^{1}H NMR (500 MHz, $CDCl_3$) δ = 7.33-7.26 (m, 3 H), 7.25-7.15 (m, 4 H), 7.15-7.09 (m, 1 H), 7.05-6.98 (m, 2 H), 6.33 (s, 1 H), 2.65 (t, *J* = 7.5 Hz, 2 H), 2.41-2.23 (m, 2 H), 1.73-1.65 (m, 2 H), 1.45-1.39 (m, 4 H), 1.34 (s, 12 H), 1.26-1.21 (m, 2 H), 1.20-1.14 (m, 2 H), 0.92 (t, *J* = 7.5 Hz, 3 H), 0.83 (t, *J* = 7.5 Hz, 3 H) ppm;
^{13}C NMR (126 MHz, $CDCl_3$) δ = 143.1, 140.8, 140.6, 137.6, 131.9, 128.5, 128.4, 128.0, 127.8, 126.5, 126.1, 83.9, 32.4, 32.3, 30.8, 28.0, 26.8, 24.8, 23.1, 22.4, 14.1, 13.7 ppm;
^{11}B NMR (160 MHz, $CDCl_3$) δ = 31.5 ppm;
HRMS (ESI): Exact mass calculated for $[C_{31}H_{43}BO_2Se+H]^+$: 539.2594, mass found: 539.2595.

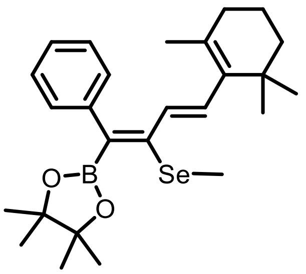

4,4,5,5-Tetramethyl-2-((1*E*,3*E*)-2-(methylselanyl)-1-phenyl-4-(2,6,6-trimethylcyclohex-1-en-1-yl)buta-1,3-dien-1-yl)-1,3,2-dioxaborolane (70), prepared according to general procedure D.

Yield 81%, colorless solid, mp: 75.3-75.8 °C;

^{1}H NMR (500 MHz, $CDCl_3$) δ = 7.32-7.27 (m, 2 H), 7.25-7.19 (m, 3 H), 6.80 (d, *J* = 15.5 Hz, 1 H), 6.02 (d, *J* = 15.5 Hz, 1 H), 2.19 (s, 3 H), 1.95 (t, *J* = 7.5 Hz, 2 H), 1.61 (s, 3 H), 1.58-1.55 (m, 2 H), 1.44-1.40 (m, 2 H), 1.34 (s, 12 H), 0.98 (s, 6 H) ppm;

^{13}C NMR (126 MHz, $CDCl_3$) δ = 140.1, 137.7, 137.6, 135.6, 129.6, 129.5, 128.9, 128.0, 126.6, 84.0, 39.5, 34.3, 33.0, 28.9, 24.7, 21.7, 19.2, 8.3 ppm;

^{11}B NMR (160 MHz, $CDCl_3$) δ = 30.7 ppm;

HRMS (ESI): Exact mass calculated for $[C_{26}H_{37}BO_2Se+H]^+$: 473.2125, mass found: 473.2125.

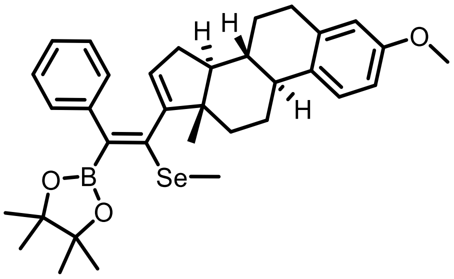

2-((*E*)-2-((8*S*,9*S*,13*S*,14*S*)-3-methoxy-13-methyl-7,8,9,11,12,13,14,15-octahydro-6H-cyclopenta[a]phenanthren-17-yl)-2-(methylselanyl)-1-phenylvinyl)-4,4,5,5-tetramethyl-1,3,2-dioxaborolane (71), prepared according to general procedure D.

Yield 33%, pale yellow solid, mp: 113-114 °C;

^{1}H NMR (500 MHz, $CDCl_3$) δ = 7.25-7.18 (m, 4 H), 7.18-7.09 (m, 2 H), 6.68 (d, *J* = 8.5, 1 H), 6.60 (s, 1 H), 5.49 (s, 1 H), 3.76 (s, 3 H), 2.88-2.76 (m, 2 H), 2.20-2.06 (m, 6 H), 1.98-1.82 (m, 3 H), 1.52-1.41 (m, 3 H), 1.33 (d, *J* = 5.5 Hz, 12 H), 1.29-1.25 (m, 2 H), 0.80 (s, 3 H) ppm;

^{13}C NMR (126 MHz, $CDCl_3$) δ = 157.3, 150.2, 140.9, 137.9, 135.4, 133.0, 131.6, 128.7, 127.5, 126.1, 126.1, 113.8, 111.3, 84.0, 56.4, 55.2, 49.2, 44.0, 37.2, 34.2, 31.6, 29.7, 27.7, 26.3, 24.7, 16.5, 8.6 ppm;

^{11}B NMR (160 MHz, $CDCl_3$) δ = 30.5 ppm;

HRMS (ESI): Exact mass calculated for $[C_{34}H_{43}BO_3Se+H]^+$: 591.2543, mass found: 591.2544.

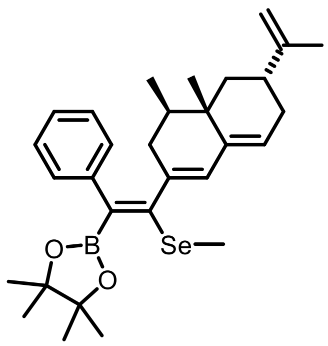

2-((*E*)-2-((4*R*,4a*S*,6*R*)-4,4a-dimethyl-6-(prop-1-en-2-yl)-3,4,4a,5,6,7-hexahydronaphthalen-2-yl)-2-(methylselanyl)-1-phenylvinyl)-4,4,5,5-tetramethyl-1,3,2-dioxaborolane (72), prepared according to general procedure D.

Yield 65%, yellow oil;

^{1}H NMR (500 MHz, $CDCl_3$) δ = 7.20 -7.13 (m, 4 H), 7.11-7.06 (m, 1 H), 5.54 (s, 1 H), 5.12 (s, 1 H), 4.68 (s, 2 H), 2.23-2.06 (m, 4 H), 2.00 (s, 3 H), 1.83 -1.72 (m, 2 H), 1.71 (s, 3 H), 1.33 (s, 12 H), 1.23-1.19 (m, 1 H), 1.09-1.02 (m, 1 H), 0.84 (d, *J* = 7.5 Hz, 3 H), 0.66 (s, 3 H) ppm;

^{13}C NMR (126 MHz, $CDCl_3$) δ = 150.4, 145.1, 142.4, 141.2, 133.4, 132.1, 128.7, 127.7, 125.9, 120.5, 108.5, 84.0, 45.8, 42.3, 41.3, 38.5, 31.5, 31.4, 24.8, 20.9, 14.4, 13.6, 7.0 ppm;

^{11}B NMR (160 MHz, $CDCl_3$) δ = 31.1 ppm;

HRMS (ESI): Exact mass calculated for $[C_{30}H_{41}BO_2Se+H]^+$: 525.2438, mass found: 525.2438.

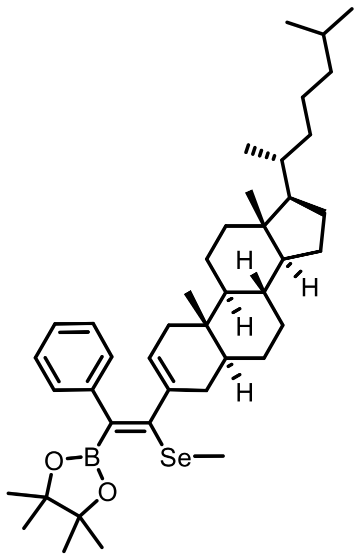

2-((*E*)-2-((10*S*,13*R*,17*R*)-10,13-dimethyl-17-((R)-6-methylheptan-2-yl)-4,5,6,7,8,9,10,11,12,13,14,15,16,17-tetradecahydro-1H-cyclopenta[a]phenanthren-3-yl)-2-(methylselanyl)-1-phenylvinyl)-4,4,5,5-tetramethyl-1,3,2-dioxaborolane (73), prepared according to general procedure D.

Yield 61%, yellow oil;

^{1}H NMR (500 MHz, $CDCl_3$) δ = 7.27 -7.04 (m, 5 H), 5.38 (t, *J* = 4.2 Hz, 1 H), 2.03 (s, 3 H), 2.01-1.92 (m, 2H), 1.91-1.77 (m, 2 H), 1.72-1.43 (m, 6 H), 1.42-1.20 (m, 22 H), 1.19-0.95 (m, 9 H), 0.92-0.87 (m, 9 H), 0.64 (s, 3 H), 0.54 (s, 3 H) ppm;

^{13}C NMR (126 MHz, $CDCl_3$) δ = 145.6, 141.6, 134.4, 128.3, 128.2, 127.7, 125.8, 83.9, 56.5, 56.3, 53.7, 42.5, 41.5, 40.1, 40.0, 39.5, 36.2, 35.8, 35.5, 34.3, 33.5, 31.7, 28.5, 28.2, 28.0,

24.78, 24.75, 24.2, 23.8, 22.8, 22.6, 21.0, 18.7, 12.04, 11.95, 6.7 ppm;
^{11}B NMR (160 MHz, $CDCl_3$) δ = 30.8 ppm;
HRMS (ESI): Exact mass calculated for $[C_{42}H_{65}BO_2Se+H]^+$: 693.4316, mass found: 693.4315.

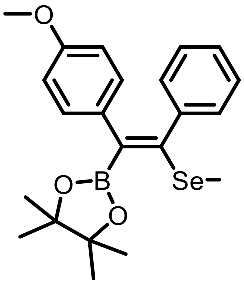

(*E*)-2-(1-(4-methoxyphenyl)-2-(methylselanyl)-2-phenylvinyl)-4,4,5,5-tetramethyl-1,3,2-dioxaborolane (74), prepared according to general procedure E.
Yield, 72%, yellow oil;
^{1}H NMR (500 MHz, $CDCl_3$) δ = 7.20-7.13 (m, 4 H), 6.93 (d, *J* = 8.8 Hz, 2 H), 6.61 (d, *J* = 8.8 Hz, 2 H), 3.69 (s, 3 H), 1.77 (s, 3 H), 1.38 (s, 12 H);
^{13}C NMR (126 MHz, $CDCl_3$) δ = 157.7, 140.3, 139.6, 133.0, 130.2, 130.1, 128.0, 126.9, 113.3, 84.1, 55.0, 24.8, 6.8 ppm;
^{11}B NMR (160 MHz, $CDCl_3$) δ = 31.7 ppm;
HRMS (ESI): Exact mass calculated for $[C_{22}H_{27}BO_3Se+H]^+$: 431.1291, mass found: 431.1299.

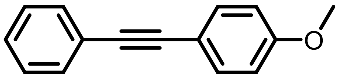

1-methoxy-4-(phenylethynyl)benzene (75).
Yield 70%, colorless solid, mp: 54-55 ^oC;
^{1}H NMR (500 MHz, $CDCl_3$) δ = 7.56-7.43 (m, 4H), 7.38-7.28 (m, 3 H), 6.91-6.85 (m, 2 H), 3.83 (s, 3 H) ppm;
^{13}C NMR (126 MHz, $CDCl_3$) δ = 159.6, 133.1, 131.5, 128.3, 127.9, 123.6, 115.4, 114.0, 89.4, 88.1, 55.3 ppm;
HRMS (ESI): Exact mass calculated for $[C_{15}H_{12}O+H]^+$: 209.0961, mass found: 209.0962.

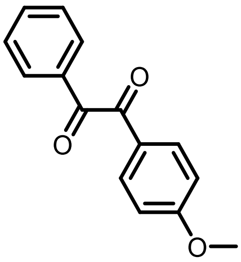

1-(4-methoxyphenyl)-2-phenylethane-1,2-dione (76).
Yield 70%, yellow solid, mp: 50-51 ^oC;
^{1}H NMR (500 MHz, $CDCl_3$) δ = 8.00-7.90 (m, 4 H), 7.66-6.62 (m, 1 H), 7.52-7.48 (m, 2 H), 6.99-6.96 (m, 2 H), 3.88 (s, 3 H) ppm;
^{13}C NMR (126 MHz, $CDCl_3$) δ = 194.9, 193.2, 165.0, 134.7, 133.2, 132.4, 129.9, 129.0, 126.1, 114.4, 55.7 ppm;
HRMS (ESI): Exact mass calculated for $[C_{15}H_{12}O_3+H]^+$: 241.0859, mass found: 241.0859.

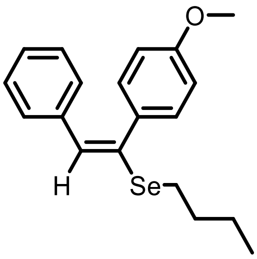

(*E*)-butyl(1-(4-methoxyphenyl)-2-phenylvinyl)selane (77).
Yield 83%, yellow oil;
^{1}H NMR (500 MHz, $CDCl_3$) δ = 7.26 (d, *J* = 8.5 Hz, 2 H), 7.13-7.05 (m, 3 H), 7.00-6.94 (m, 2 H), 6.88 (s, 1 H), 6.84 (d, *J* = 8.5 Hz, 2 H), 3.82 (s, 3 H), 2.52 (t, *J* = 7.5 Hz, 2 H), 1.64-1.58 (m, 2 H), 1.37-1.31 (m, 2 H), 0.85 (t, *J* = 7.5 Hz, 3 H) ppm;
^{13}C NMR (126 MHz, $CDCl_3$) δ = 159.1, 137.3, 134.6, 131.7, 130.7, 129.4, 128.8, 128.0, 126.4, 114.0, 55.3, 32.2, 26.0, 23.0, 13.6 ppm;
HRMS (ESI): Exact mass calculated for $[C_{19}H_{22}OSe+H]^+$: 347.0909, mass found: 347.0904.

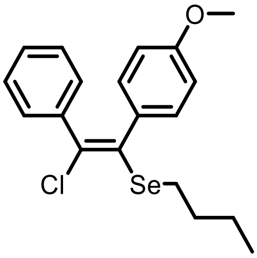

(*Z*)-butyl(2-chloro-1-(4-methoxyphenyl)-2-phenylvinyl)selane (78).
Yield 52%, yellow oil;
^{1}H NMR (500 MHz, $CDCl_3$) δ = 7.16-7.08 (m, 5 H), 7.04 (d, *J* = 8.5 Hz, 2 H), 6.73 (d, *J* = 8.5 Hz, 2 H), 3.76 (s, 3 H), 2.27 (t, *J* = 7.5 Hz, 2 H), 1.49-1.43 (m, 2 H), 1.25-1.20 (m, 2 H), 0.77 (t, *J* = 7.5 Hz, 3 H) ppm;
^{13}C NMR (126 MHz, $CDCl_3$) δ = 158.8, 138.9, 134.1, 131.4, 130.1, 129.4, 127.7, 127.5, 127.3, 113.6, 55.2, 32.4, 27.0, 22.9, 13.5 ppm;
HRMS (ESI): Exact mass calculated for $[C_{19}H_{21}ClOSe+H]^+$: 381.0519, mass found: 381.0512.

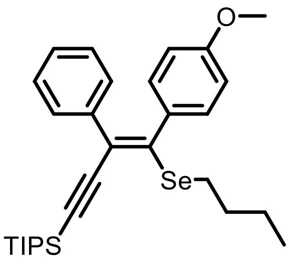

(*Z*)-(4-(butylselanyl)-4-(4-methoxyphenyl)-3-phenylbut-3-en-1-yn-1-yl)triisopropylsilane (79).
Yield 60%, yellow oil;
^{1}H NMR (500 MHz, $CDCl_3$) δ = 7.15 (d, *J* = 8.5 Hz, 2 H), 7.09-7.03 (m, 5 H), 6.75 (d, *J* = 8.5 Hz, 2 H), 3.77 (s, 3 H), 2.33 (t, *J* = 7.5 Hz, 2 H), 1.49-1.43 (m, 2 H), 1.29-1.22 (m, 5 H), 1.15 (s, 18 H), 0.78 (t, *J* = 7.5 Hz, 3 H) ppm;
^{13}C NMR (126 MHz, $CDCl_3$) δ = 158.9, 145.1, 139.0, 131.3, 130.7, 129.4, 127.5, 126.2, 121.4,

113.6, 107.8, 98.0, 55.2, 32.5, 26.7, 22.8, 18.8, 13.5, 11.5 ppm;
HRMS (ESI): Exact mass calculated for $[C_{30}H_{42}OSeSi+H]^+$: 527.2243, mass found: 527.2238.

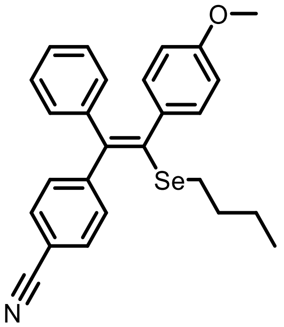

(*Z*)-4-(2-(butylselanyl)-2-(4-methoxyphenyl)-1-phenylvinyl)benzonitrile (80).
Yield 82%, yellow solid, mp: 87-88 °C;
^{1}H NMR (500 MHz, CD_2Cl_2) δ = 7.64 (d, *J* = 8.0 Hz, 2 H), 7.49 (d, *J* = 8.0 Hz, 2 H), 7.19 (d, *J* = 8.5 Hz, 2 H), 7.08-7.00 (m, 3 H), 6.90-6.83 (m, 2 H), 6.74 (d, *J* = 8.5 Hz, 2 H), 3.77 (s, 3 H), 2.27-2.20 (m, 2 H), 1.50-1.43 (m, 2 H), 1.24-1.17 (m, 2 H), 0.78 (t, *J* = 7.5 Hz, 3 H) ppm;
^{13}C NMR (126 MHz, $CDCl_3$) δ = 158.7, 149.5, 141.8, 140.4, 135.7, 132.1, 131.8, 131.7, 131.0, 130.3, 127.9, 126.4, 119.1, 113.5, 110.4, 55.2, 32.6, 26.6, 22.8, 13.5 ppm;
HRMS (ESI): Exact mass calculated for $[C_{26}H_{25}NOSe+H]^+$: 448.1174, mass found: 448.1167.

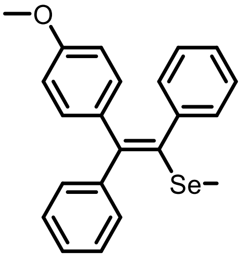

(*E*)-(2-(4-methoxyphenyl)-1,2-diphenylvinyl)(methyl)selane (81).
Yield 82%, yellow solid, mp: 94-95 °C;
^{1}H NMR (500 MHz, $CDCl_3$) δ = 7.45-7.34 (m, 4 H), 7.33-7.29 (m, 1 H), 7.27-7.22 (m, 4 H), 7.18-7.12 (m, 1 H), 6.84 (d, *J* = 8.5 Hz, 2 H), 6.54 (d, *J* = 8.5 Hz, 2 H), 3.67 (s, 3 H), 1.62 (s, 3 H);
^{13}C NMR (126 MHz, $CDCl_3$) δ = 157.7, 144.3, 140.9, 139.9, 134.6, 132.5, 131.2, 130.5, 129.7, 128.4, 128.1, 127.1, 126.8, 112.9, 55.1, 7.1 ppm;
HRMS (EI): Exact mass calculated for $[C_{22}H_{20}OSe]^+$: 380.0674, mass found: 380.0664.

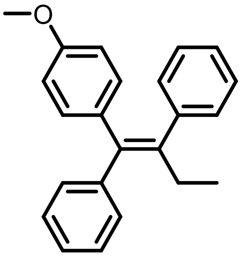

(*Z*)-(1-(4-methoxyphenyl)but-1-ene-1,2-diyl)dibenzene (82).
Yield 72%, yellow solid, mp: 114-116 °C;
^{1}H NMR (500 MHz, $CDCl_3$) δ = 7.37-7.32 (m, 2 H), 7.27-7.22 (m, 3 H), 7.05-6.96 (m, 5 H), 6.89 (d, *J* = 8.5 Hz, 2 H), 6.70 (d, *J* = 8.5 Hz, 2 H), 3.75 (s, 3 H), 2.45 (q, *J* = 7.5 Hz, 2 H),

0.93 (t, J = 7.5 Hz, 3 H) ppm;
^{13}C NMR (126 MHz, $CDCl_3$) δ = 157.9, 143.8, 143.3, 141.7, 138.4, 134.3, 130.81, 130.76, 129.5, 128.1, 127.4, 126.5, 125.6, 113.3, 55.1, 28.9, 13.7 ppm;
HRMS (ESI): Exact mass calculated for $[C_{23}H_{22}O+H]^+$: 315.1743, mass found: 315.1742.

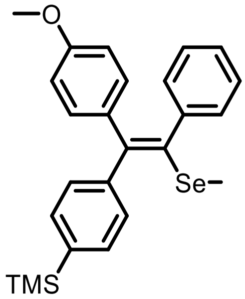

(*Z*)-(4-(1-(4-methoxyphenyl)-2-(methylselanyl)-2-phenylvinyl)phenyl)trimethylsilane (83), analogously to the preparation of 81.
Yield 81%, colorless solid, mp: 59-60 °C;
^{1}H NMR (500 MHz, $CDCl_3$) δ = 7.56 (d, J = 7.5 Hz, 2 H), 7.41 (d, J = 7.5 Hz, 2 H), 7.31-7.25 (m, 4 H), 7.20-7.15 (m, 1 H), 6.88 (d, J = 8.5 Hz, 2 H), 6.58 (d, J = 8.5 Hz, 2 H), 3.70 (s, 3 H), 1.66 (s, 3 H), 0.32 (s, 9 H) ppm;
^{13}C NMR (126 MHz, $CDCl_3$) δ = 157.7, 144.5, 141.0, 139.9, 139.1, 134.7, 133.3, 132.3, 131.2, 130.5, 128.8, 128.1, 126.8, 112.9, 55.0, 7.2, -1.0 ppm;
HRMS (ESI): Exact mass calculated for $[C_{25}H_{28}OSeSi+H]^+$: 453.1147, mass found: 453.1149.

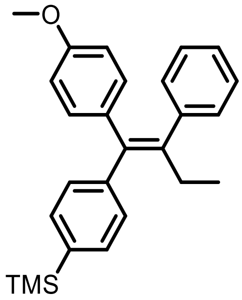

(*E*)-(4-(1-(4-methoxyphenyl)-2-phenylbut-1-en-1-yl)phenyl)trimethylsilane (84), analogously to the preparation of 82.
Yield 85%, colorless solid, mp: 78-79 °C;
^{1}H NMR (500 MHz, $CDCl_3$) δ = 7.56-7.48 (m, 2 H), 7.39-7.35 (m, 1 H), 7.29-7.24 (m, 2 H), 7.10-7.05 (m, 4 H), 6.95 (d, J = 7.5 Hz, 2 H), 6.75 (d, J = 7.5 Hz, 2 H), 3.79 (s, 3 H), 2.52 (q, J = 7.5 Hz, 2 H), 0.99 (t, J = 7.5 Hz, 3 H), 0.33 (s, 9 H) ppm;
^{13}C NMR (126 MHz, $CDCl_3$) δ = 157.5, 144.2, 142.5, 141.4, 138.3, 135.5, 133.1, 131.9, 129.7, 128.7, 127.9, 126.0, 112.8, 55.0, 29.0, 13.6, -1.0 ppm;
HRMS (ESI): Exact mass calculated for $[C_{26}H_{30}OSi+H]^+$: 387.2139, mass found: 387.2141.

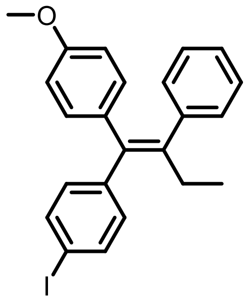

(*E*)-1-iodo-4-(1-(4-methoxyphenyl)-2-phenylbut-1-en-1-yl)benzene (85).
Yield 50%, colorless solid, mp: 93-94 °C;
^{1}H NMR (500 MHz, $CDCl_3$) δ = 7.67 (d, *J* = 7.5 Hz, 2 H), 7.20-7.15 (m, 2 H), 7.14-7.06 (m, 3 H), 6.99 (d, *J* = 7.8 Hz, 2 H), 6.75 (d, *J* = 8.0 Hz, 2 H), 6.55 (d, *J* = 8.0 Hz, 2 H), 3.68 (s, 3 H), 2.45 (q, *J* = 7.0 Hz, 2 H), 0.92 (t, *J* = 7.5 Hz, 3 H) ppm;
^{13}C NMR (126 MHz, $CDCl_3$) δ = 157.7, 143.4, 142.1, 141.9, 137.3, 137.2, 134.9, 131.9, 131.5, 129.6, 127.9, 126.2, 112.9, 92.1, 55.0, 29.0, 13.6 ppm;
HRMS (ESI): Exact mass calculated for $[C_{23}H_{21}IO+H]^+$: 441.0710, mass found: 441.0707.

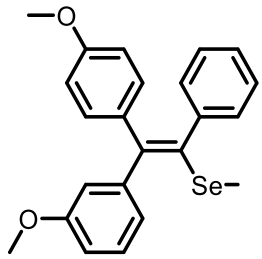

(*Z*)-(2-(3-methoxyphenyl)-2-(4-methoxyphenyl)-1-phenylvinyl)(methyl)selane (86), analogously to the preparation of 81.
Yield 71%, yellow solid, mp: 82-84 °C;
^{1}H NMR (500 MHz, CD_2Cl_2) δ = 7.33-7.28 (m, 1 H), 7.27-7.22 (m, 4 H), 7.17-7.12 (m, 1 H), 7.01-6.92 (m, 2 H), 6.87-6.83 (m, 3 H), 6.56-6.51 (m, 2 H), 3.82 (s, 3 H), 3.67 (s, 3 H), 1.61 (s, 3 H) ppm;
^{13}C NMR (126 MHz, $CDCl_3$) δ = 159.5, 157.7, 145.5, 140.6, 139.8, 134.4, 132.5, 131.1, 130.5, 129.4, 128.1, 126.8, 122.0, 115.2, 112.9, 112.6, 55.3, 55.1, 7.2 ppm;
HRMS (ESI): Exact mass calculated for $[C_{23}H_{22}O_2Se+H]^+$: 411.0858, mass found: 411.0853.

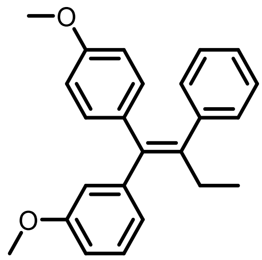

(*E*)-1-methoxy-3-(1-(4-methoxyphenyl)-2-phenylbut-1-en-1-yl)benzene (87), analogously to the preparation of 82.
Yield 81%, colorless solid, 91-92 °C;
^{1}H NMR (500 MHz, $CDCl_3$) δ = 7.27-7.24 (m, 1 H), 7.06-6.97 (m, 5 H), 6.92-6.87 (m, 2 H), 6.85-6.76 (m, 3 H), 6.73-6.67 (m, 2 H), 3.79 (s, 3 H), 3.75 (s, 3 H), 2.46 (q, *J* = 7.5 Hz, 2 H),

0.93 (t, J = 7.5 Hz, 3 H) ppm;
^{13}C NMR (126 MHz, $CDCl_3$) δ = 159.4, 157.9, 145.2, 143.0, 141.7, 138.2, 134.2, 130.8, 130.7, 129.1, 127.4, 125.6, 122.0, 115.1, 113.3, 112.0, 55.2, 55.1, 29.0, 13.7 ppm;
HRMS (ESI): Exact mass calculated for $[C_{24}H_{24}O_2+H]^+$: 345.1849, mass found: 345.1859.

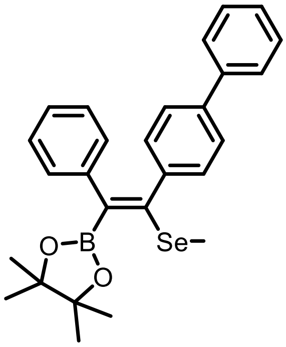

(*E*)-2-(2-([1,1'-biphenyl]-4-yl)-2-(methylselanyl)-1-phenylvinyl)-4,4,5,5-tetramethyl-1,3,2-dioxaborolane (88), prepared according to general procedure D.
Yield 75%, yellow solid, mp: 89-90 °C;
^{1}H NMR (500 MHz, $CDCl_3$) δ = 7.53 (d, J = 7.5 Hz, 2 H), 7.41-7.35 (m, 4 H), 7.30-7.26 (m, 1 H), 7.24 (d, J = 7.5 Hz, 2 H), 7.09-7.04 (m, 4 H), 7.04-6.98 (m, 1 H), 1.82 (s, 3 H), 1.37 (s, 12 H) ppm;
^{13}C NMR (126 MHz, $CDCl_3$) δ = 141.6, 140.7, 140.5, 139.5, 138.4, 130.6, 129.1, 128.8, 127.9, 127.3, 126.9, 126.5, 126.0, 84.2, 24.8, 7.1 ppm;
^{11}B NMR (160 MHz, $CDCl_3$) δ = 32.5 ppm;
HRMS (ESI): Exact mass calculated for $[C_{27}H_{29}BO_2Se+H]^+$: 477.1499, mass found: 477.1500.

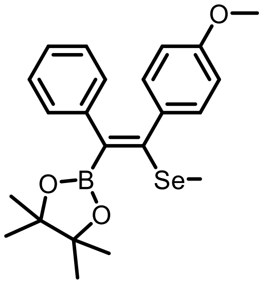

The compound 89 is identical to compound 6.

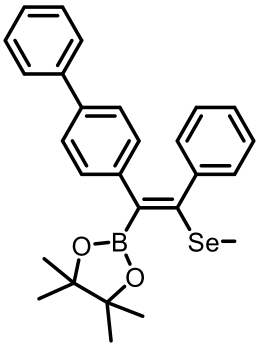

(*E*)-2-(1-([1,1'-biphenyl]-4-yl)-2-(methylselanyl)-2-phenylvinyl)-4,4,5,5-tetramethyl-1,3,2-dioxaborolane (90), prepared according to general procedure E.
Yield 70%, yellow solid, mp: 91-92 °C;
^{1}H NMR (500 MHz, $CDCl_3$) δ = 7.52-7.48 (m, 2 H), 7.38-7.34 (m, 2 H), 7.33-7.30 (m, 2 H),

7.28-7.25 (m, 1 H), 7.22-7.11 (m, 5 H), 7.09-7.05 (m, 2 H), 1.78 (s, 3 H), 1.39 (s, 12 H) ppm;
^{13}C NMR (126 MHz, $CDCl_3$) δ = 142.4, 140.7, 139.7, 139.4, 138.4, 130.1, 129.5, 128.6, 128.0, 127.1, 127.0, 126.8, 126.4, 84.3, 24.9, 7.0 ppm;
^{11}B NMR (160 MHz, $CDCl_3$) δ = 32.6 ppm;
HRMS (ESI): Exact mass calculated for $[C_{27}H_{29}BO_2Se+H]^+$: 477.1499, mass found: 477.1500.

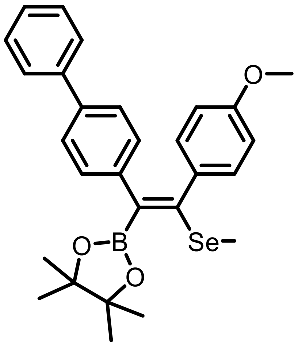

(*E*)-2-(1-([1,1'-biphenyl]-4-yl)-2-(4-methoxyphenyl)-2-(methylselanyl)vinyl)-4,4,5,5-tetramethyl-1,3,2-dioxaborolane (91), prepared according to general procedure E.
Yield 72%, yellow solid, mp: 91-92 ^{o}C;
^{1}H NMR (500 MHz, $CDCl_3$) δ = 7.52 (d, *J* = 7.6 Hz, 2 H), 7.39-7.32 (m, 4 H), 7.30-7.25 (m, 1 H), 7.14 (d, *J* = 8.3 Hz, 2 H), 7.09 (d, *J* = 8.0 Hz, 2 H), 6.70 (d, *J* = 8.4 Hz, 2 H), 3.74 (s, 3 H), 1.80 (s, 3 H), 1.39 (s, 12 H) ppm;
^{13}C NMR (126 MHz, $CDCl_3$) δ = 158.6, 142.2, 140.8, 140.0, 138.2, 131.6, 131.5, 129.5, 128.7, 127.0, 126.8, 126.5, 113.4, 84.2, 55.1, 24.9, 7.0 ppm;
^{11}B NMR (160 MHz, $CDCl_3$) δ = 32.3 ppm;
HRMS (ESI): Exact mass calculated for $[C_{28}H_{31}BO_3Se+H]^+$: 507.1604, mass found: 507.1614.

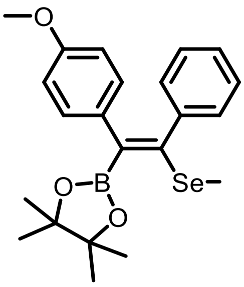

The compound 92 is identical to compound 74.

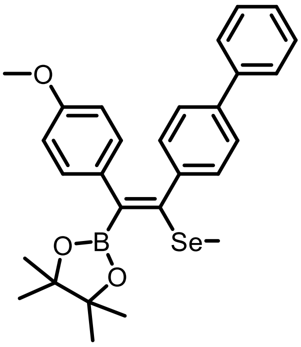

(*E*)-2-(2-([1,1'-biphenyl]-4-yl)-1-(4-methoxyphenyl)-2-(methylselanyl)vinyl)-4,4,5,5-tetramethyl-1,3,2-dioxaborolane (93), prepared according to general procedure E.
Yield 71%, yellow solid, mp: 82-84 ^{o}C;

^{1}H NMR (500 MHz, $CDCl_3$) δ = 7.58-7.55 (m, 2 H), 7.44-7.39 (m, 4 H), 7.33-7.29 (m, 1 H), 7.26-7.23 (m, 2 H), 6.98 (d, *J* = 9.0 Hz, 2 H), 6.63 (d, *J* = 9.0 Hz, 2 H), 3.70 (s, 3 H), 1.83 (s, 3 H), 1.38 (s, 12 H) ppm;
^{13}C NMR (126 MHz, $CDCl_3$) δ = 157.8, 140.5, 139.7, 139.3, 138.6, 133.0, 130.6, 130.3, 128.7, 127.3, 126.8, 126.5, 113.4, 84.2, 55.0, 24.8, 7.0 ppm;
^{11}B NMR (160 MHz, $CDCl_3$) δ = 32.7 ppm;
HRMS (ESI): Exact mass calculated for $[C_{28}H_{31}BO_3Se+H]^+$: 507.1604, mass found: 507.1605.

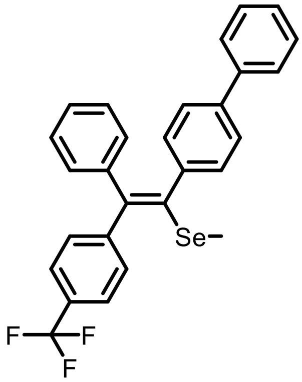

(*Z*)-(1-([1,1'-biphenyl]-4-yl)-2-phenyl-2-(4-(trifluoromethyl)phenyl)vinyl)(methyl)selane (94), analogously to the preparation of 81.
Yield 79%, yellow solid, mp: 186-187 °C;
^{1}H NMR (500 MHz, Chloroform-*d*) δ = 7.67-7.64 (m, 2 H), 7.61-7.55 (m, 4 H), 7.51-7.48 (m, 2 H), 7.45-7.41 (m, 2 H), 7.36-7.31 (m, 3 H), 7.07-7.02 (m, 3 H), 6.98-6.93 (m, 2 H), 1.73 (s, 3 H) ppm;
^{13}C NMR (126 MHz, $CDCl_3$) δ = 147.7, 141.7, 140.4, 140.3, 139.7, 138.3, 134.9, 130.9, 130.2, 130.1, 129.1 (q, *J* = 32.8 Hz), 128.8, 127.8, 127.5, 126.9, 126.7, 126.5, 125.4 (q, *J* = 3.8 Hz), 124.4 (q, *J* = 273.4 Hz), 7.4 ppm;
HRMS (ESI): Exact mass calculated for $[C_{28}H_{21}F_3Se+H]^+$: 495.0833, mass found: 495.0834.

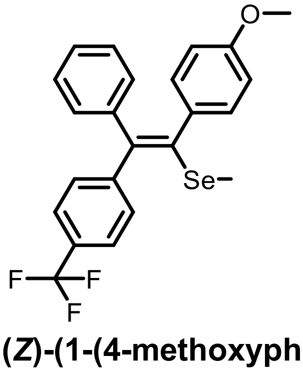

(*Z*)-(1-(4-methoxyphenyl)-2-phenyl-2-(4-(trifluoromethyl)phenyl)vinyl)(methyl)selane (95), analogously to the preparation of 81.
Yield 78%, yellow solid, mp: 85-86 °C;
^{1}H NMR (500 MHz, $CDCl_3$) δ = 7.63 (d, *J* = 8.0 Hz, 2 H), 7.53 (d, *J* = 8.0 Hz, 2 H), 7.18 (d, *J* = 8.5 Hz, 2 H), 7.04 (d, *J* = 7.0 Hz, 3 H), 6.91 (d, *J* = 7.0 Hz, 2 H), 6.77 (d, *J* = 8.5 Hz, 2 H), 3.78 (s, 3 H), 1.68 (s, 3 H) ppm;
^{13}C NMR (126 MHz, $CDCl_3$) δ = 158.6, 147.9, 141.9, 139.7, 135.3, 131.7, 131.5, 130.3, 130.1, 129.0 (q, *J* = 32.8 Hz), 127.8, 126.3, 125.4(q, *J* = 3.8 Hz), 124.3 (q, *J* = 273.4 Hz), 113.6, 55.2, 7.3 ppm;

HRMS (EI): Exact mass calculated for [$C_{23}H_{19}F_3OSe$]$^+$: 448.0548, mass found: 448.0563.

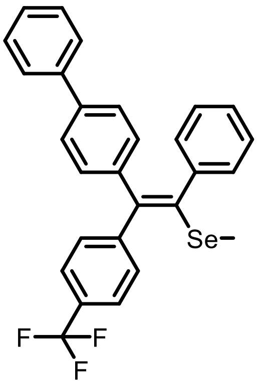

(*Z*)-(2-([1,1'-biphenyl]-4-yl)-1-phenyl-2-(4-(trifluoromethyl)phenyl)vinyl)(methyl)selane (96), analogously to the preparation of 81.
Yield 72%, yellow solid, mp: 111-112 ^{o}C;
^{1}H NMR (500 MHz, $CDCl_3$) δ = 7.68-7.64 (m, 2 H), 7.5-7.55 (m, 2 H), 7.49-7.45 (m, 2 H), 7.38-7.34 (m, 2 H), 7.30-7.25 (m, 6 H), 7.21-7.16 (m, 1 H), 6.97-6.90 (m, 2 H), 1.66 (s, 3 H) ppm;
^{13}C NMR (126 MHz, $CDCl_3$) δ = 147.6, 140.5, 140.4, 139.5, 139.2, 138.9, 135.6, 130.5, 130.4, 130.3, 129.2 (q, *J* = 32.8 Hz), 128.7, 128.3, 127.3, 127.3, 126.8, 126.3, 125.5 (q, *J* = 3.8 Hz), 124.4 (q, *J* = 273.4 Hz), 7.3 ppm;
HRMS (EI): Exact mass calculated for [$C_{28}H_{21}F_3Se$]$^+$: 494.0755, mass found: 494.0749.

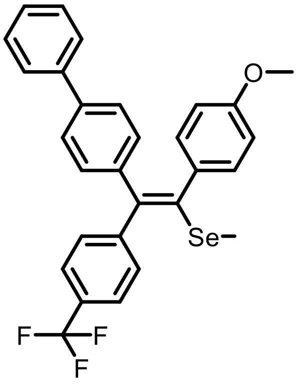

(*Z*)-(2-([1,1'-biphenyl]-4-yl)-1-(4-methoxyphenyl)-2-(4-(trifluoromethyl)phenyl)vinyl)(methyl)selane (97), analogously to the preparation of 81.
Yield 75%, yellow solid, mp: 116-117 ^{o}C;
^{1}H NMR (500 MHz, $CDCl_3$) δ = 7.70 (d, *J* = 8.0 Hz, 2 H), 7.63-7.60 (m, 2 H), 7.53 (d, *J* = 8.0 Hz, 2 H), 7.43-7.39 (m, 2 H), 7.36-7.30 (m, 3 H), 7.29-7.25 (m, 2 H), 7.00 (d, *J* = 8.5 Hz, 2 H), 6.84 (d, *J* = 8.5 Hz, 2 H), 3.82 (s, 3 H), 1.73 (s, 3 H) ppm;
^{13}C NMR (126 MHz, CD_2Cl_2) δ = 158.8, 148.1, 141.1, 140.3, 139.0, 138.6, 135.8, 131.7, 131.5, 130.6, 130.4, 128.7, 127.3, 127.0 (q, *J* = 13.6 Hz), 126.7, 126.2, 125.3 (q, *J* = 13.8 Hz), 124.4 (q, *J* = 270.5 Hz), 113.6, 55.1, 7.0ppm;
HRMS (ESI): Exact mass calculated for [$C_{29}H_{23}F_3OSe$]$^+$: 524.0861, mass found: 524.0871.

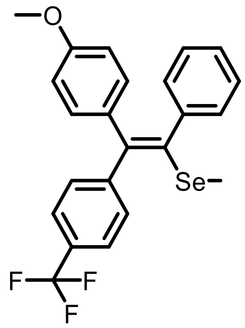

(*E*)-(2-(4-methoxyphenyl)-1-phenyl-2-(4-(trifluoromethyl)phenyl)vinyl)(methyl)selane (98), analogously to the preparation of 81.
Yield 72%, yellow solid, mp: 92-93 °C;
^{1}H NMR (500 MHz, $CDCl_3$) δ = 7.63 (d, *J* = 7.9 Hz, 2H), 7.52 (d, *J* = 7.9 Hz, 2H), 7.28-7.22 (m, 4H), 7.19-7.14 (m, 1H), 6.80 (d, *J* = 8.7 Hz, 2H), 6.55 (d, *J* = 8.7 Hz, 2H), 3.67 (s, 3H), 1.64 (s, 3H) ppm;
^{13}C NMR (126 MHz, $CDCl_3$) δ = 157.9, 147.9, 139.7, 139.5, 134.0, 133.8, 131.3, 130.4, 130.2, 129.0(q, *J* = 32.8 Hz), 128.2, 127.1, 125.4 (q, *J* = 3.8 Hz), 124.4 (q, *J* = 273.4 Hz), 113.1, 55.1, 7.2 ppm;
HRMS (ESI): Exact mass calculated for $[C_{23}H_{19}F_3OSe+H]^+$: 449.0626, mass found: 449.0628.

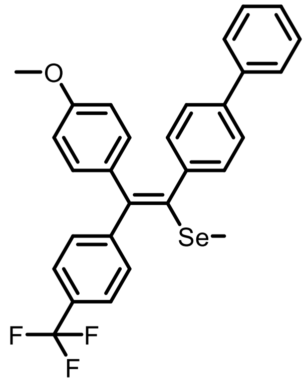

(*E*)-(1-([1,1'-biphenyl]-4-yl)-2-(4-methoxyphenyl)-2-(4-(trifluoromethyl)phenyl)vinyl)(methyl)selane (99), analogously to the preparation of 81.
Yield 81%, yellow solid, mp: 123 -124 °C;
^{1}H NMR (500 MHz, $CDCl_3$) δ = 7.63 (d, *J* = 8.0 Hz, 2 H), 7.59 (d, *J* = 7.5 Hz, 2 H), 7.53 (d, *J* = 7.5 Hz, 2 H), 7.50 (d, *J* = 8.0 Hz, 2 H), 7.43-7.39 (m, 2 H), 7.35-7.30 (m, 3 H), 6.84 (d, *J* = 8.5 Hz, 2 H), 6.57 (d, *J* = 8.5 Hz, 2 H), 3.65 (s, 3 H), 1.70 (s, 3 H) ppm;
^{13}C NMR (126 MHz, $CDCl_3$) δ = 158.0, 148.0, 140.3, 140.1, 139.5, 138.6, 134.1, 133.5, 131.4, 131.0, 130.2, 129.1(q, *J* = 32.8 Hz), 128.8, 127.4, 126.9, 126.8, 125.4 (q, *J* = 3.8 Hz), 124.4 (q, *J* = 272.2 Hz), 113.2, 55.1, 7.3 ppm;
HRMS (EI): Exact mass calculated for $[C_{29}H_{23}F_3OSe]^+$: 524.0861, mass found: 524.0860.

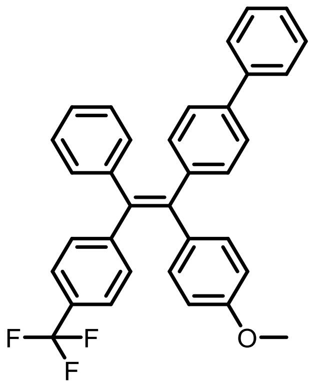

(*Z*)-4-(1-(4-methoxyphenyl)-2-phenyl-2-(4-(trifluoromethyl)phenyl)vinyl)-1,1'-biphenyl (100).

Yield 79%, yellow solid, mp: 184-185 ^{o}C;

^{1}H NMR (500 MHz, $CDCl_3$) δ = 7.58-7.54 (m, 2 H), 7.42-7.35 (m, 6 H), 7.33-7.29 (m, 1 H), 7.18-7.11 (m, 5 H), 7.11-7.07 (m, 2 H), 7.06-7.02 (m, 2 H), 6.97 (d, *J* = 8.5 Hz, 2 H), 6.69 (d, *J* = 8.5 Hz, 2 H), 3.77 (s, 3 H) ppm;

^{13}C NMR (126 MHz, $CDCl_3$) δ = 158.5, 147.9, 143.3, 142.5, 141.8, 140.6, 139.2, 138.8, 135.4, 132.7, 131.8, 131.6, 131.4, 128.7, 128.3 (q, *J* = 32.8 Hz), 128.0, 127.3, 126.9, 126.7, 126.3, 124.7(q, *J* = 3.8 Hz), 124.3(q, *J* = 273.4 Hz), 113.4, 55.2 ppm;

HRMS (ESI): Exact mass calculated for $[C_{34}H_{25}F_3O+H]^+$: 507.1930, mass found: 507.1925.

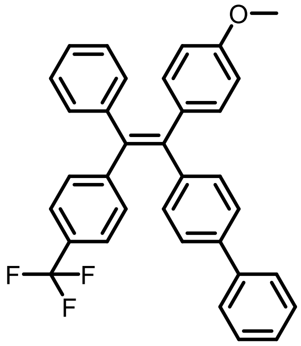

(*E*)-4-(1-(4-methoxyphenyl)-2-phenyl-2-(4-(trifluoromethyl)phenyl)vinyl)-1,1'-biphenyl (101), analogously to the preparation of 100.

Yield 78%, yellow solid, mp: 143-144 ^{o}C;

^{1}H NMR (500 MHz, $CDCl_3$) δ = 7.60-7.56 (m, 2 H), 7.44-7.36 (m, 6 H), 7.35-7.31 (m, 1 H), 7.20-7.13 (m, 5 H), 7.12-7.08 (m, 2 H), 7.07-7.02 (m, 2 H), 6.99 (d, *J* = 8.5 Hz, 2 H), 6.68 (d, *J* = 8.5 Hz, 2 H), 3.76 (s, 3 H) ppm;

^{13}C NMR (126 MHz, $CDCl_3$) δ = 158.5, 147.8, 143.5, 142.3, 141.8, 140.5, 139.4, 138.8, 135.6, 132.6, 131.9, 131.6, 131.4, 128.8, 128.2 (q, *J* = 32.8 Hz), 128.0, 127.4, 126.9, 126.7, 126.4, 124.7 (q, *J* = 3.8 Hz), 124.2 (q, *J* = 273.4 Hz), 113.2, 55.1 ppm;

HRMS (ESI): Exact mass calculated for $[C_{34}H_{25}F_3O+H]^+$: 507.1930, mass found: 506.1920

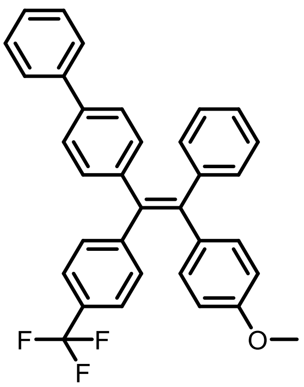

(*Z*)-4-(2-(4-methoxyphenyl)-2-phenyl-1-(4-(trifluoromethyl)phenyl)vinyl)-1,1'-biphenyl (102), analogously to the preparation of 100.
Yield 72%, colorless solid, mp: 158-159 °C;
^{1}H NMR (500 MHz, $CDCl_3$) δ = 7.55 (d, *J* = 8.0 Hz, 2 H), 7.42-7.34 (m, 6 H), 7.33-7.29 (m, 1 H), 7.21-7.18 (m, 2 H), 7.15-7.11 (m, 3 H), 7.09-7.02 (m, 4 H), 6.93 (d, *J* = 8.5 Hz, 2 H), 6.66 (d, *J* = 8.5 Hz, 2 H), 3.76 (s, 3 H) ppm;
^{13}C NMR (126 MHz, $CDCl_3$) δ = 158.5, 147.9, 143.5, 142.3, 142.3, 140.5, 139.1, 138.2, 135.4, 132.6, 131.8, 131.7, 131.3, 128.7, 128.2 (q, *J* = 32.8 Hz), 127.8, 127.3, 126.87, 126.8, 126.4, 124.7 (q, *J* = 3.8 Hz), 124.3 (q, *J* = 273.4 Hz), 113.3, 55.2 ppm;
HRMS (EI): Exact mass calculated for $[C_{34}H_{25}F_3O]^+$: 506.1852, mass found: 506.1847.

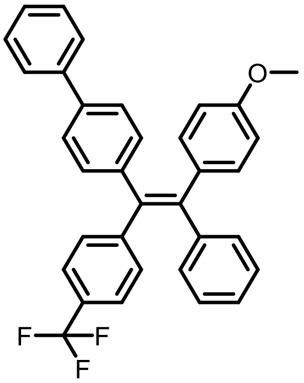

(*E*)-4-(2-(4-methoxyphenyl)-2-phenyl-1-(4-(trifluoromethyl)phenyl)vinyl)-1,1'-biphenyl (103), analogously to the preparation of 100.
Yield 75%, colorless solid, mp: 194-195 °C;
^{1}H NMR (500 MHz, $CDCl_3$) δ = 7.60-7.55 (m, 2 H), 7.44-7.31 (m, 7 H), 7.19-7.12 (m, 5 H), 7.11-7.07 (m, 2 H), 7.06-7.02 (m, 2 H), 6.98 (d, *J* = 8.5 Hz, 2 H), 6.67 (d, *J* = 8.5 Hz, 2 H), 3.75 (s, 3 H) ppm;
^{13}C NMR (126 MHz, $CDCl_3$) δ = 158.5, 147.8, 143.4, 142.4, 142.3, 140.5, 139.1, 138.2, 135.6, 132.6, 131.8, 131.7, 131.4, 128.8, 128.2 (q, *J* = 32.8 Hz), 127.9, 127.3, 126.9, 126.9, 126.5, 124.7(q, *J* = 3.8 Hz), 124.3(q, *J* = 273.4 Hz), 113.3, 55.1 ppm;
HRMS (EI): Exact mass calculated for $[C_{34}H_{25}F_3O]^+$: 506.1852, mass found: 506.1855.

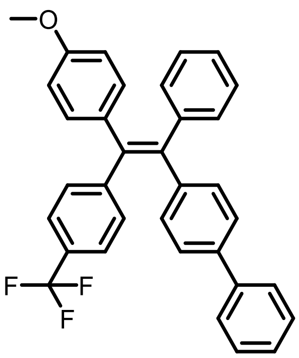

(*E*)-4-(2-(4-methoxyphenyl)-1-phenyl-2-(4-(trifluoromethyl)phenyl)vinyl)-1,1'-biphenyl (104), analogously to the preparation of 100.

Yield 72%, yellow solid, mp: 158-159 °C;

^{1}H NMR (500 MHz, $CDCl_3$) δ = 7.57-7.53 (m, 2 H), 7.42-7.35 (m, 6 H), 7.33-7.30 (m, 1 H), 7.21-7.13 (m, 5 H), 7.10-7.03 (m, 4 H), 6.92 (d, *J* = 8.5 Hz, 2 H), 6.66 (d, *J* = 8.5 Hz, 2 H), 3.75 (s, 3 H) ppm;

^{13}C NMR (126 MHz, $CDCl_3$) δ = 158.4, 147.8, 143.5, 142.3, 141.2, 140.5, 139.3, 139.2, 135.5, 132.6, 131.8, 131.7, 131.3, 128.8, 128.3 (q, *J* = 32.8 Hz), 127.9, 127.3, 126.9, 126.7, 126.4, 124.7 (q, *J* = 3.8 Hz), 124.2 (q, *J* = 273.4 Hz), 113.3, 55.1 ppm;

HRMS (ESI): Exact mass calculated for $[C_{34}H_{25}F_3O+H]^+$: 507.1930, mass found: 507.1923.

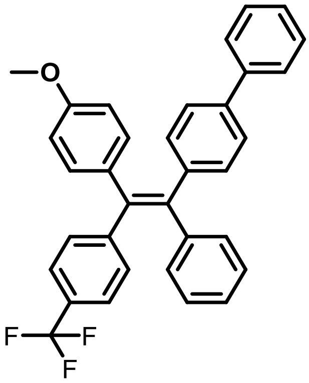

(*E*)-4-(2-(4-methoxyphenyl)-2-phenyl-1-(4-(trifluoromethyl)phenyl)vinyl)-1,1'-biphenyl (105), analogously to the preparation of 100.

Yield 81%, colorless solid, mp: 189-190 °C;

^{1}H NMR (500 MHz, $CDCl_3$) δ = 7.60-7.55 (m, 2 H), 7.44-7.35 (m, 6 H), 7.34-7.30 (m, 1 H), 7.18-7.13 (m, 5 H), 7.13-7.09 (m, 2 H), 7.08-7.03 (m, 2 H), 6.97 (d, *J* = 8.5 Hz, 2 H), 6.69 (d, *J* = 8.5 Hz, 2 H), 3.76 (s, 3 H) ppm;

^{13}C NMR (126 MHz, $CDCl_3$) δ = 158.5, 147.9, 143.3, 142.6, 141.3, 140.6, 139.3, 139.1, 135.4, 132.6, 131.7, 131.6, 131.4, 128.8, 128.2 (q, *J* = 32.8 Hz), 128.0, 127.3, 126.9, 126.9, 126.4, 124.6 (q, *J* = 3.8 Hz), 124.3 (q, *J* = 273.4 Hz), 113.4, 55.1 ppm;

HRMS (EI): Exact mass calculated for $[C_{34}H_{25}F_3O]^+$: 506.1852, mass found: 506.1849.

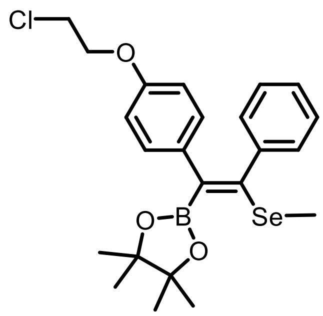

(E)-2-(1-(4-(2-chloroethoxy)phenyl)-2-(methylselanyl)-2-phenylvinyl)-4,4,5,5-tetramethyl-1,3,2-dioxaborolane (106), prepared according to general procedure E.
Yield 69 %, colorless oil;
^{1}H NMR (500 MHz, $CDCl_3$) δ = 7.18-7.14 (m, 5 H), 6.94 (d, J = 8.7 Hz, 2 H), 6.61 (d, J = 8.7 Hz, 2 H), 4.11 (t, J = 6.0 Hz, 2 H), 3.73 (t, J = 6.0 Hz, 2 H), 1.76 (s, 3 H), 1.37 (s, 12 H) ppm;
^{13}C NMR (126 MHz, $CDCl_3$) δ = 156.3, 141.0, 139.5, 133.8, 130.3, 130.0, 128.0, 126.9, 114.0, 84.2, 67.7, 41.9, 24.8, 6.8 ppm
^{11}B NMR (160 MHz, $CDCl_3$) δ = 32.0 ppm;
HRMS (ESI): Exact mass calculated for $[C_{23}H_{28}BClO_3Se+H]^+$: 479.1058, mass found: 479.1060.

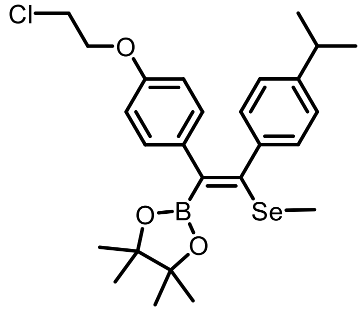

(E)-2-(1-(4-(2-chloroethoxy)phenyl)-2-(4-isopropylphenyl)-2-(methylselanyl)vinyl)-4,4,5,5-tetramethyl-1,3,2-dioxaborolane (107), prepared according to general procedure E.
Yield 71 %, colorless oil;
^{1}H NMR (500 MHz, $CDCl_3$) δ = 7.08 (d, J = 8.5 Hz, 2 H), 7.01 (d, J = 8.5 Hz, 2 H), 6.94 (d, J = 8.5 Hz, 2 H), 6.61 (d, J = 8.5 Hz, 2 H), 4.11 (t, J = 6.0 Hz, 2 H), 3.72 (t, J = 6.0 Hz, 2 H), 2.86 – 2.77 (m, 1 H), 1.77 (s, 3 H), 1.37 (s, 12 H), 1.19 (d, J = 7.0 Hz, 6 H) ppm;
^{13}C NMR (126 MHz, $CDCl_3$) δ = 156.2, 147.6, 140.9, 136.8, 134.0, 130.3, 130.0, 125.9, 114.0, 84.1, 67.8, 41.9, 33.7, 24.8, 23.9, 6.9 ppm;
^{11}B NMR (160 MHz, $CDCl_3$) δ = 31.8 ppm;
HRMS (ESI): Exact mass calculated for $[C_{26}H_{34}BClO_3Se+H]^+$: 521.1528, mass found: 521.1528.

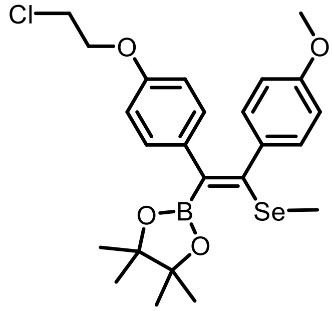

(*E*)-2-(1-(4-(2-chloroethoxy)phenyl)-2-(4-methoxyphenyl)-2-(methylselanyl)vinyl)-4,4,5,5-tetramethyl-1,3,2-dioxaborolane (108), prepared according to general procedure E.

Yield 75 %, colorless oil;

^{1}H NMR (500 MHz, $CDCl_3$) δ = 7.10 (d, *J* = 8.7 Hz, 2 H), 6.95 (d, *J* = 8.7 Hz, 2 H), 6.69 (d, *J* = 8.7 Hz, 2 H), 6.63 (d, *J* = 8.8 Hz, 2 H), 4.12 (t, *J* = 5.9 Hz, 2 H), 3.76-3.72 (m, 5 H), 1.78 (s, 3 H), 1.36 (s, 12 H) ppm;

^{13}C NMR (126 MHz, $CDCl_3$) δ = 158.4, 156.2, 140.8, 134.1, 131.7, 131.4, 130.3, 114.1, 113.4, 84.1, 67.8, 55.1, 41.9, 24.8, 6.8 ppm

^{11}B NMR (160 MHz, $CDCl_3$) δ = 31.4 ppm;

HRMS (ESI): Exact mass calculated for $[C_{24}H_{30}BClO_4Se+H]^+$: 509.1164, mass found: 509.1160.

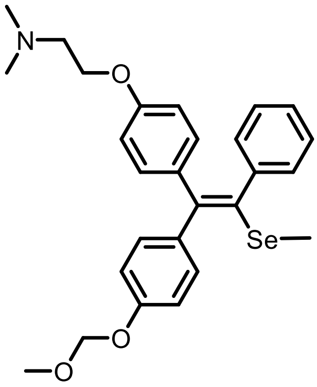

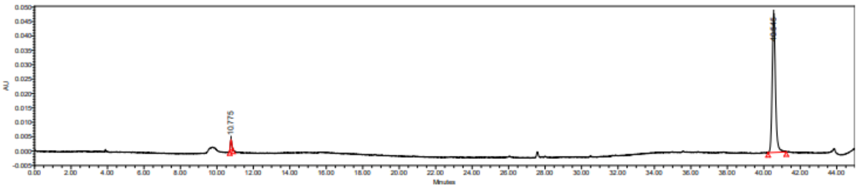

Name	Retention Time	Area	% Area	Height
1	10.775	19798	3.21	3688
2	40.545	596712	96.79	48311

(*Z*)-2-(4-(1-(4-(methoxymethoxy)phenyl)-2-(methylselanyl)-2-phenylvinyl)phenoxy)-N,N-dimethylethan-1-amine (109).

Yield 67%, yellow oil;

^{1}H NMR (500 MHz, $CDCl_3$) δ = 7.31 (d, *J* = 8.0 Hz, 2 H), 7.24 – 7.11 (m, 5 H), 7.04 (d, *J* = 8.0 Hz, 2 H), 6.82 (d, *J* = 8.0 Hz, 2 H), 6.55 (d, *J* = 8.0 Hz, 2 H), 5.20 (s, 2 H), 4.02 (t, *J* = 5.0 Hz, 2 H), 3.50 (s, 3 H), 2.83 (t, *J* = 5.0 Hz, 2 H), 2.42 (s, 6 H), 1.62 (s, 3 H) ppm;

^{13}C NMR (126 MHz, $CDCl_3$) δ = 156.5, 156.3, 140.3, 139.9, 137.7, 135.4, 132.4, 131.3, 130.9, 130.5, 128.1, 126.7, 115.9, 113.5, 94.5, 64.8, 57.9, 56.1, 45.4, 7.1 ppm;

HRMS (ESI): Exact mass calculated for $[C_{27}H_{31}NO_3Se+H]^+$: 498.1542, mass found: 498.1537.

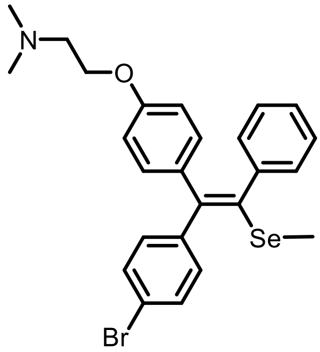

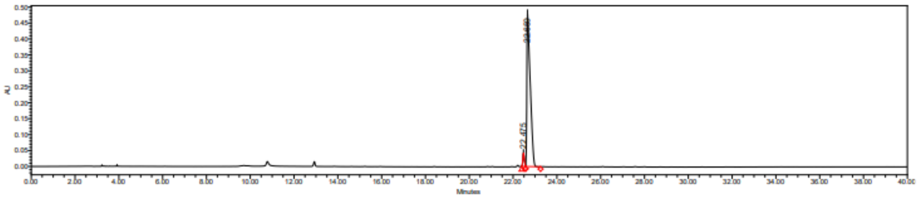

Name	Retention Time	Area	% Area	Height
1	22.475	196951	3.70	41690
2	22.659	5127062	96.30	480494

(*Z*)-2-(4-(1-(4-bromophenyl)-2-(methylselanyl)-2-phenylvinyl)phenoxy)-N,N-dimethylethan-1-amine (110), analogously to the preparation of 109.

Yield 65 %, yellow solid, mp: 112 -113 °C;

^{1}H NMR (500 MHz, $CDCl_3$) δ = 7.50 (d, *J* = 8.0 Hz, 2 H), 7.31 – 7.12 (m, 7 H), 6.78 (d, *J* = 8.0 Hz, 2 H), 6.55 (d, *J* = 8.0 Hz, 2 H), 3.91 (t, *J* = 5.0 Hz, 2 H), 2.63 (t, *J* = 5.0 Hz, 2 H), 2.28 (d, *J* = 4.5 Hz, 6 H), 1.64 (d, *J* = 4.5 Hz, 3 H) ppm;

^{13}C NMR (126 MHz, $CDCl_3$) δ = 157.2, 143.1, 139.8, 139.7, 134.3, 133.1, 131.6, 131.5, 131.2, 130.4, 128.1, 126.9, 121.1, 113.6, 65.8, 58.3, 45.9, 7.2 ppm;

HRMS (ESI): Exact mass calculated for $[C_{25}H_{26}BrNOSe+H]^+$: 516.0436, mass found: 516.0432.

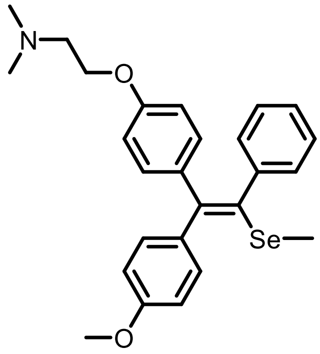

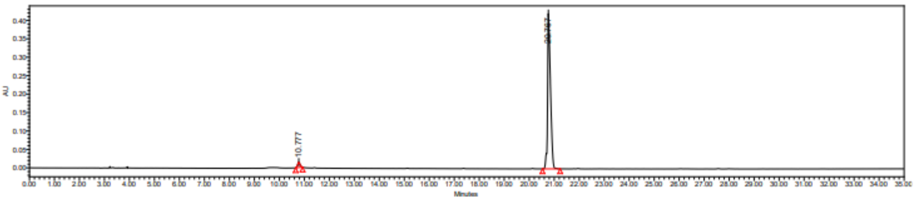

Name	Retention Time	Area	% Area	Height
1	10.777	98718	2.86	14626
2	20.767	3353948	97.14	420881

(*E*)-2-(4-(1-(4-methoxyphenyl)-2-(methylselanyl)-2-phenylvinyl)phenoxy)-N,N-dimethylethan-1-amine (111), analogously to the preparation of 109.

Yield 71%, colorless solid, mp: 97-98 °C;

^{1}H NMR (500 MHz, $CDCl_3$) δ = 7.32 (d, *J* = 8.5 Hz, 2 H), 7.27 – 7.10 (m, 5 H), 6.91 (d, *J* = 8.5 Hz, 2 H), 6.81 (d, *J* = 8.5 Hz, 2 H), 6.55 (d, *J* = 8.5 Hz, 2 H), 3.94 (t, *J* = 5.5 Hz, 2 H), 3.82 (s, 3 H), 2.68 (t, *J* = 5.5 Hz, 2 H), 2.31 (s, 6 H), 1.62 (s, 3 H) ppm;

^{13}C NMR (126 MHz, $CDCl_3$) δ = 158.6, 156.8, 140.5, 140.1, 136.6, 135.2, 132.1, 131.2, 130.9, 130.5, 128.1, 126.7, 113.6, 113.5, 65.5, 58.2, 55.2, 45.8, 7.1 ppm;

HRMS (ESI): Exact mass calculated for $[C_{26}H_{29}NO_2Se+H]^+$: 468.1437, mass found: 468.1437.

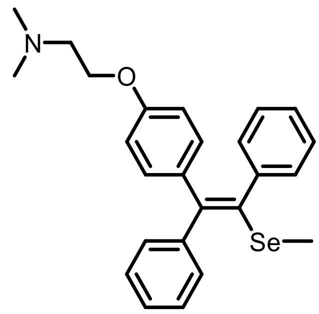

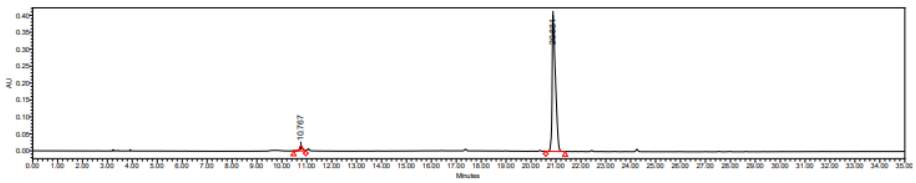

Name	Retention Time	Area	% Area	Height
1	10.767	132674	3.49	15332
2	20.881	3673003	96.51	403638

(*E*)-N,N-dimethyl-2-(4-(2-(methylselanyl)-1,2-diphenylvinyl)phenoxy)ethan-1-amine (112), analogously to the preparation of 109.

Yield 67%, yellow solid, mp: 91-92 °C;

^{1}H NMR (500 MHz, $CDCl_3$) δ = 7.41 -7.28 (m, 5 H), 7.28 -7.10 (m, 5 H), 6.82 (d, *J* = 8.5 Hz, 2 H), 6.55 (d, *J* = 8.5 Hz, 2 H), 3.93 (t, *J* = 5.5 Hz, 2 H), 2.67 (t, *J* = 5.5 Hz, 2 H), 2.30 (s, 6 H), 1.62 (s, 3 H) ppm;

^{13}C NMR (126 MHz, $CDCl_3$) δ = 156.9, 144.2, 140.9, 139.9, 134.7, 132.5, 131.1, 130.5, 129.6, 128.3, 128.1, 127.1, 126.8, 113.5, 65.5, 58.0, 45.7, 7.1 ppm;

HRMS (ESI): Exact mass calculated for $[C_{25}H_{27}NOSe+H]^+$: 438.1331, mass found: 438.1326.

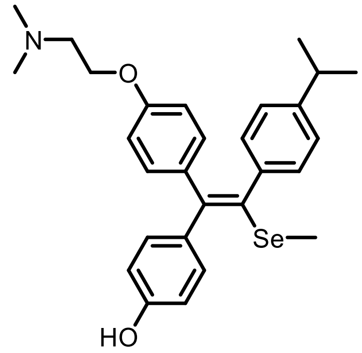

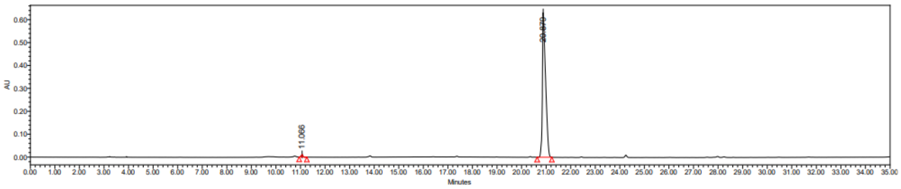

Name	Retention Time	Area	% Area	Height
1	11.066	52736	0.91	11402
2	20.879	5748056	99.09	631589

(*E*)-4-(1-(4-(2-(dimethylamino)ethoxy)phenyl)-2-(4-isopropylphenyl)-2-(methylselanyl)vinyl)phenol (113), analogously to the preparation of 109.

Yield 45%, yellow solid, mp:122-123 °C;

^{1}H NMR (500 MHz, MeOD) δ = 7.06 (m, 6 H), 6.80 (d, *J* = 7.5 Hz, 2 H), 6.71 (d, *J* = 8.0 Hz, 2 H), 6.61 (d, *J* = 8.0 Hz, 2 H), 4.15 (s, 2 H), 3.46 (s, 2 H), 3.26 (s, 1 H), 2.87 (s, 6 H), 2.78-2.73 (m, 1 H), 1.50 (s, 3 H), 1.15 (d, *J* = 6.5 Hz, 6 H) ppm;

^{13}C NMR (126 MHz, MeOD) δ = 156.2, 155.7, 147.3, 140.1, 137.6, 136.8, 135.4, 132.4, 131.1, 130.7, 130.3, 125.7, 114.6, 113.3, 61.7, 56.5, 42.8, 33.6, 23.0, 5.5 ppm;

HRMS (ESI): Exact mass calculated for $[C_{28}H_{33}NO_2Se+H]^+$: 496.1749, mass found: 496.1751.

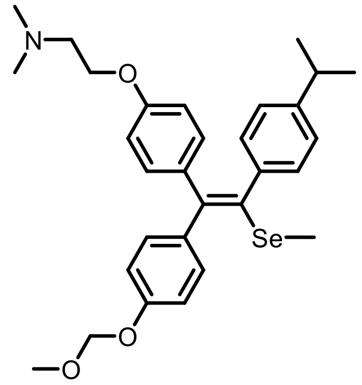

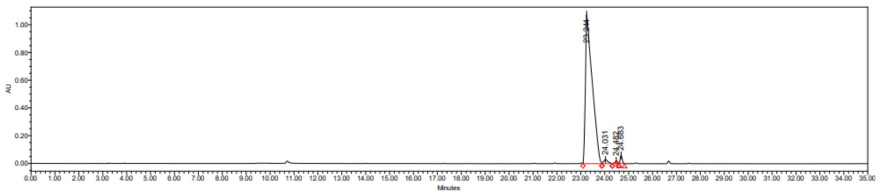

Name	Retention Time	Area	% Area	Height
1	23.244	21272601	96.24	1075014
2	24.031	372093	1.68	28897
3	24.482	141848	0.64	21464
4	24.683	317646	1.44	58045

(*Z*)-2-(4-(2-(4-isopropylphenyl)-1-(4-(methoxymethoxy)phenyl)-2-(methylselanyl)vinyl)phenoxy)-N,N-dimethylethan-1-amine (114), analogously to the preparation of 109.

Yield 70%, yellow oil;

^{1}H NMR (500 MHz, $CDCl_3$) δ = 7.31 (d, *J* = 8.5 Hz, 2 H), 7.14 (d, *J* = 8.0 Hz, 2 H), 7.07 (d, *J* = 8.0 Hz, 2 H), 7.03 (d, *J* = 8.5 Hz, 2 H), 6.81 (d, *J* = 8.5 Hz, 2 H), 6.55 (d, *J* = 8.5 Hz, 2 H), 5.20 (s, 2 H), 3.93 (t, *J* = 5.5 Hz, 2 H), 3.50 (s, 3 H), 2.86-2.82 (m, 1 H), 2.65 (t, *J* = 5.5 Hz, 2 H), 2.29 (s, 6 H), 1.62 (s, 3 H), 1.21 (d, *J* = 7.5 Hz, 6 H) ppm;

^{13}C NMR (126 MHz, $CDCl_3$) δ = 156.8, 156.3, 147.4, 140.1, 138.0, 137.3, 135.2, 132.5, 131.2, 131.0, 130.4, 126.0, 115.9, 113.4, 94.5, 65.7, 58.2, 56.1, 45.8, 33.7, 23.9, 7.1 ppm;

HRMS (ESI): Exact mass calculated for $[C_{30}H_{37}NO_3Se+H]^+$: 540.2011, mass found: 540.2013.

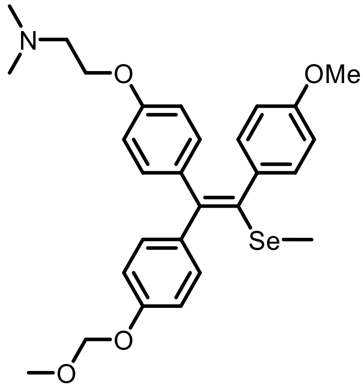

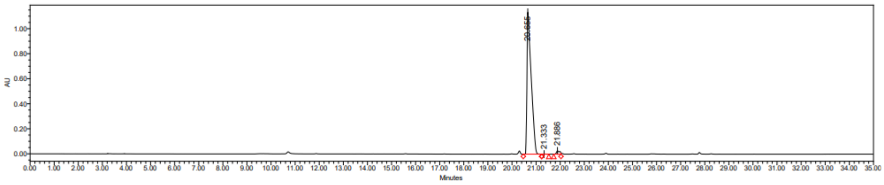

Name	Retention Time	Area	% Area	Height
2	21.333	7532	0.05	771
3	21.886	253081	1.80	26678
1	20.655	13799580	98.15	1134275

(*Z*)-2-(4-(1-(4-(methoxymethoxy)phenyl)-2-(4-methoxyphenyl)-2-(methylselanyl)vinyl)phenoxy)-N,N-dimethylethan-1-amine (115), analogously to the preparation of 109.

Yield 72%, colorless solid, mp: 80-81 °C;

^{1}H NMR (500 MHz, $CDCl_3$) δ = 7.30 (d, *J* = 8.5 Hz, 2 H), 7.15 (d, *J* = 8.5 Hz, 2 H), 7.02 (d, *J* = 8.5 Hz, 2 H), 6.81 (d, *J* = 8.5 Hz, 2 H), 6.75 (d, *J* = 8.5 Hz, 2 H), 6.56 (d, *J* = 8.5 Hz, 2 H), 5.19 (s, 2 H), 3.94 (t, *J* = 5.5 Hz, 2 H), 3.77 (s, 3 H), 3.50 (s, 3 H), 2.67 (t, *J* = 5.5 Hz, 2 H), 2.30 (s, 6 H), 1.64 (s, 3 H) ppm;

^{13}C NMR (126 MHz, $CDCl_3$) δ = 158.2, 156.8, 156.3, 140.1, 138.0, 135.3, 132.4, 132.2, 131.7, 131.2, 130.9, 115.9, 113.5, 113.4, 94.5, 65.6, 58.2, 56.1, 55.1, 45.8, 7.1 ppm;

HRMS (ESI): Exact mass calculated for $[C_{28}H_{33}NO_4Se+H]^+$: 528.1648, mass found: 528.1653.

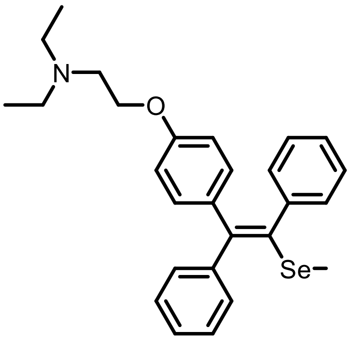

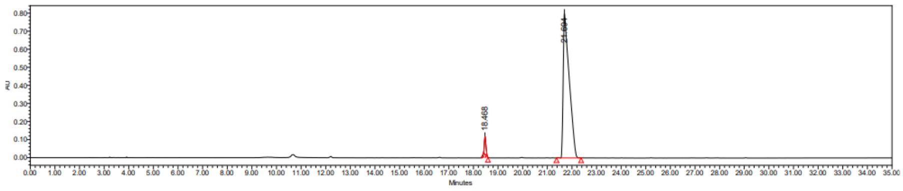

Name	Retention Time	Area	% Area	Height
1	18.468	432898	3.10	98158
2	21.694	13550296	96.90	802410

(*E*)-N,N-diethyl-2-(4-(2-(methylselanyl)-1,2-diphenylvinyl)phenoxy)ethan-1-amine (116), analogously to the preparation of 109.

Yield 62%, colorless solid, mp: 125-126 °C;

^{1}H NMR (500 MHz, $CDCl_3$) δ = 7.46-7.39 (m, 4 H), 7.36-7.24 (m, 5 H), 7.21-7.15 (m, 1 H), 6.86 (d, *J* = 8.4 Hz, 2 H), 6.58 (d, *J* = 8.4 Hz, 2 H), 3.93 (t, *J* = 6.4 Hz, 2 H), 2.81 (t, *J* = 6.4 Hz, 2 H), 2.61 (q, *J* = 7.1 Hz, 4 H), 1.66 (s, 3 H), 1.05 (t, *J* = 7.1 Hz, 6 H) ppm;
^{13}C NMR (126 MHz, $CDCl_3$) δ = 157.0, 144.3, 141.0, 140.0, 134.6, 132.4, 131.2, 130.5, 129.7, 128.3, 128.1, 127.1, 126.8, 113.5, 66.3, 51.7, 47.8, 11.9, 7.1 ppm;
HRMS (ESI): Exact mass calculated for $[C_{27}H_{31}NOSe+H]^+$: 466.1644, mass found: 466.1639.

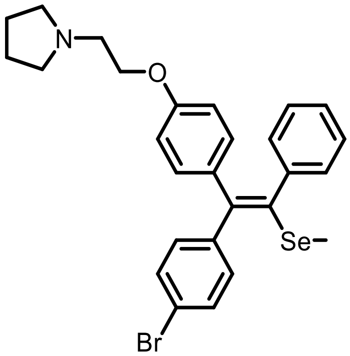

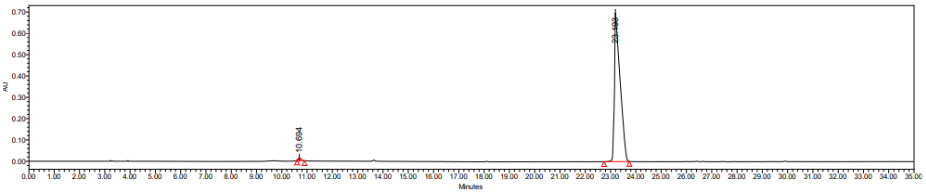

Name	Retention Time	Area	% Area	Height
1	10.694	81608	0.75	12000
2	23.193	10869002	99.25	697147

(*Z*)-1-(2-(4-(1-(4-bromophenyl)-2-(methylselanyl)-2-phenylvinyl)phenoxy)ethyl)pyrrolidine (117), analogously to the preparation of 109.
Yield 61%, colorless solid, mp: 124-125 °C;
^{1}H NMR (500 MHz, $CDCl_3$) δ = 7.50 (d, *J* = 8.0 Hz, 2 H), 7.28 (d, *J* = 8.0 Hz, 2 H), 7.25-7.20 (m, 4 H), 7.17-7.12 (m, 1 H), 6.78 (d, *J* = 8.0 Hz, 2 H), 6.55 (d, *J* = 8.0 Hz, 2 H), 3.96 (t, *J* = 6.0 Hz, 2 H), 2.80 (t, *J* = 6.0 Hz, 2 H), 2.56 (s, 4 H), 1.77 (s, 4 H), 1.63 (s, 3 H) ppm;
^{13}C NMR (126 MHz, $CDCl_3$) δ = 157.2, 143.1, 139.8, 139.7, 134.3, 133.1, 131.6, 131.5, 131.2, 130.4, 128.2, 126.9, 121.1, 113.7, 66.9, 55.1, 54.7, 23.5, 7.2 ppm;
HRMS (ESI): Exact mass calculated for $[C_{27}H_{28}BrNOSe+H]^+$: 542.0592, mass found: 542.0591.

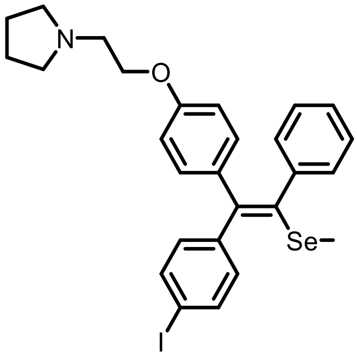

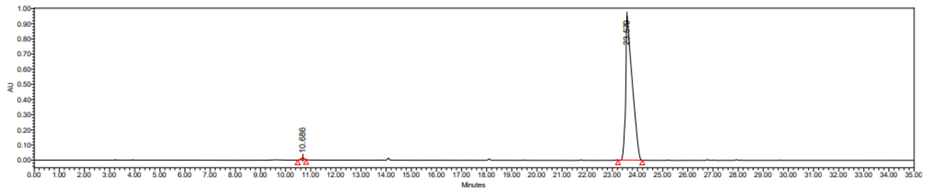

Name	Retention Time	Area	% Area	Height
1	10.686	104194	0.60	13374
2	23.579	17305979	99.40	954037

(*Z*)-1-(2-(4-(1-(4-iodophenyl)-2-(methylselanyl)-2-phenylvinyl)phenoxy)ethyl)pyrrolidine (118), analogously to the preparation of 109.
Yield 53%, colorless solid, mp: 129-131 °C;
^{1}H NMR (500 MHz, $CDCl_3$) δ = 7.70 (d, *J* = 8.0 Hz, 2 H), 7.25-7.20 (m, 4 H), 7.17-7.13 (m, 3 H), 6.78 (d, *J* = 9.0 Hz, 2 H), 6.55 (d, *J* = 9.0 Hz, 2 H), 3.96 (t, *J* = 6.0 Hz, 2 H), 2.80 (t, *J* = 6.0 Hz, 2 H), 2.56 (s, 4 H), 1.77 (s, 4 H), 1.63 (s, 3 H) ppm;
^{13}C NMR (126 MHz, $CDCl_3$) δ = 157.2, 143.8, 139.9, 139.7, 137.5, 134.2, 133.1, 131.8, 131.2, 130.4, 128.2, 126.9, 113.7, 92.8, 66.9, 55.1, 54.7, 23.5, 7.2 ppm;
HRMS (ESI): Exact mass calculated for $[C_{27}H_{28}INOSe+H]^+$: 590.0454, mass found: 590.0454.

6. NMR Spectra

500 MHz, 298 K, $CDCl_3$ as solvent

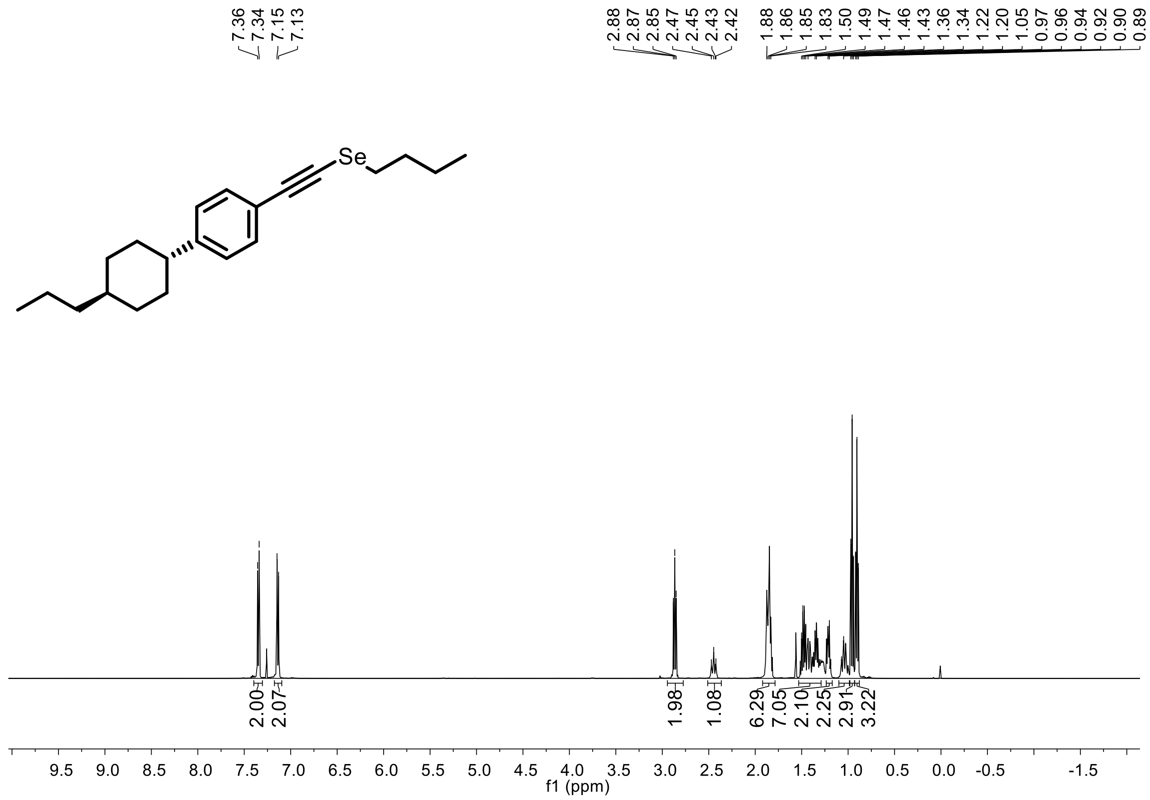

126 MHz, 298 K, $CDCl_3$ as solvent

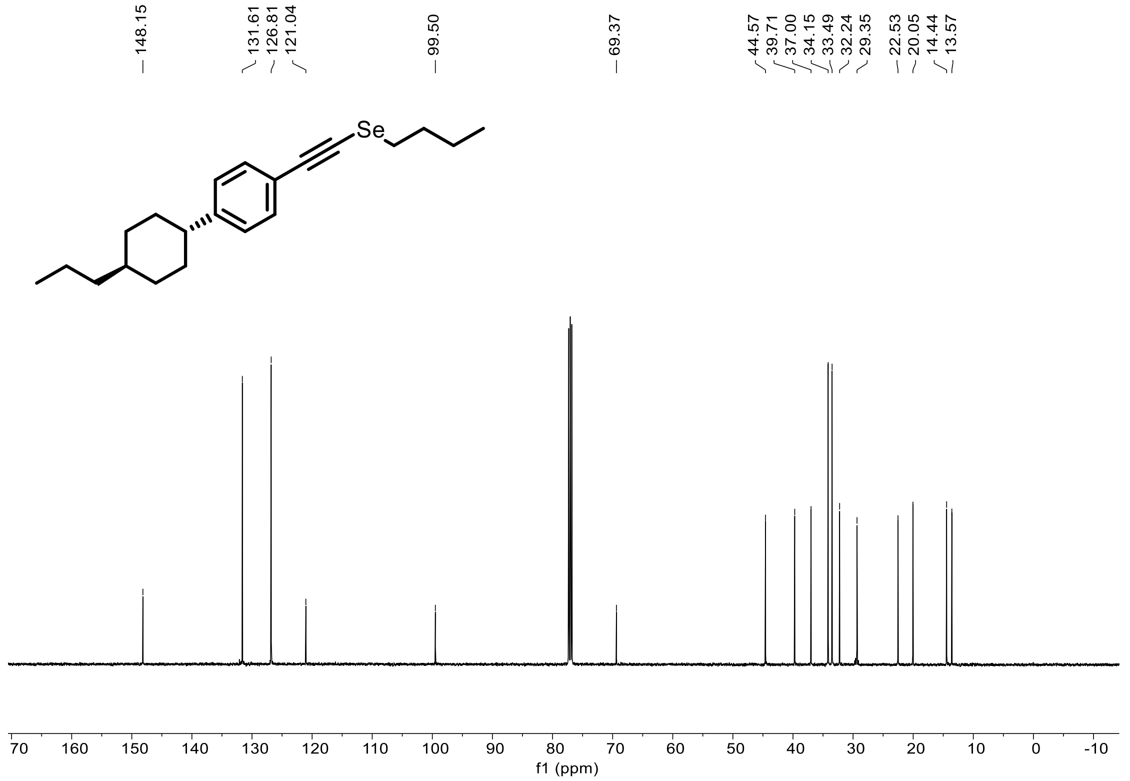

500 MHz, 298 K, $CDCl_3$ as solvent

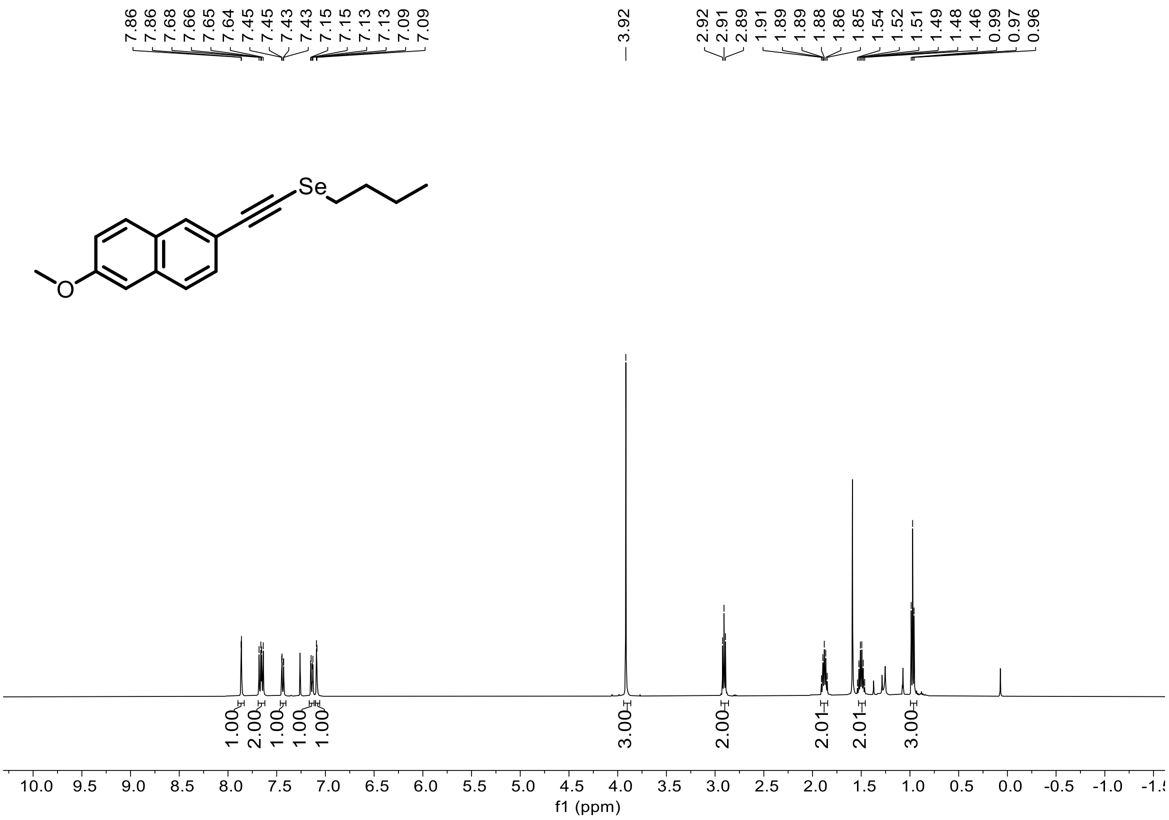

126 MHz, 298 K, $CDCl_3$ as solvent

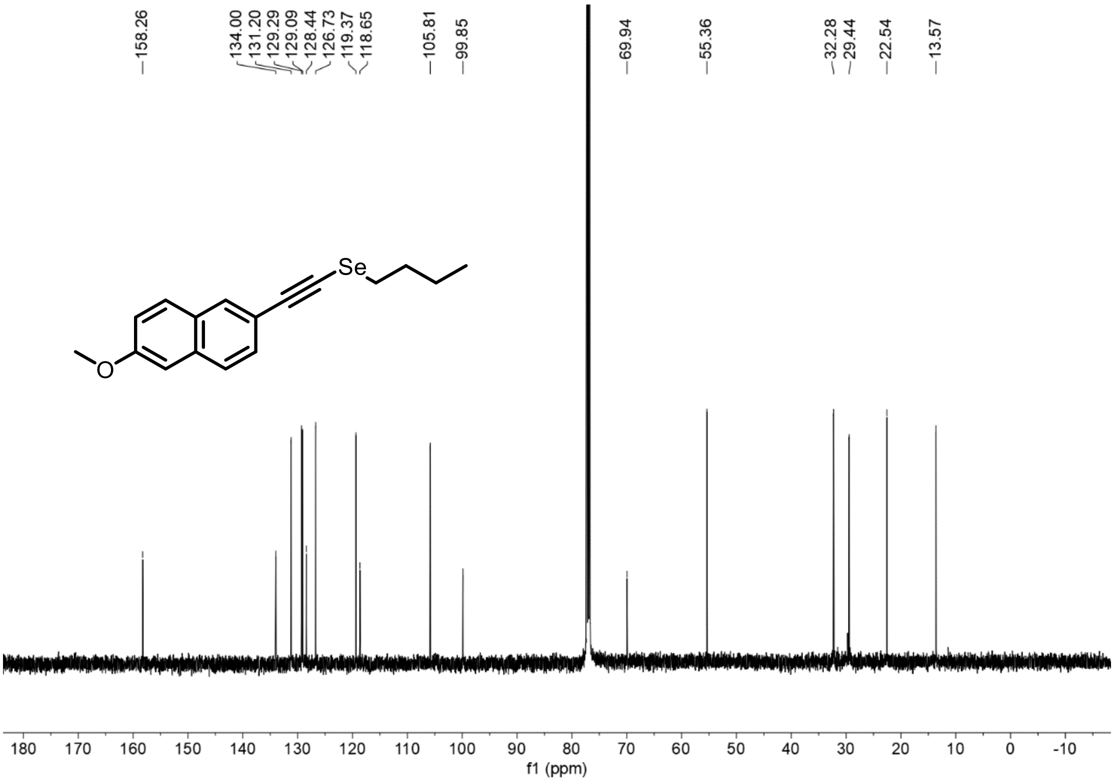

500 MHz, 298 K, $CDCl_3$ as solvent

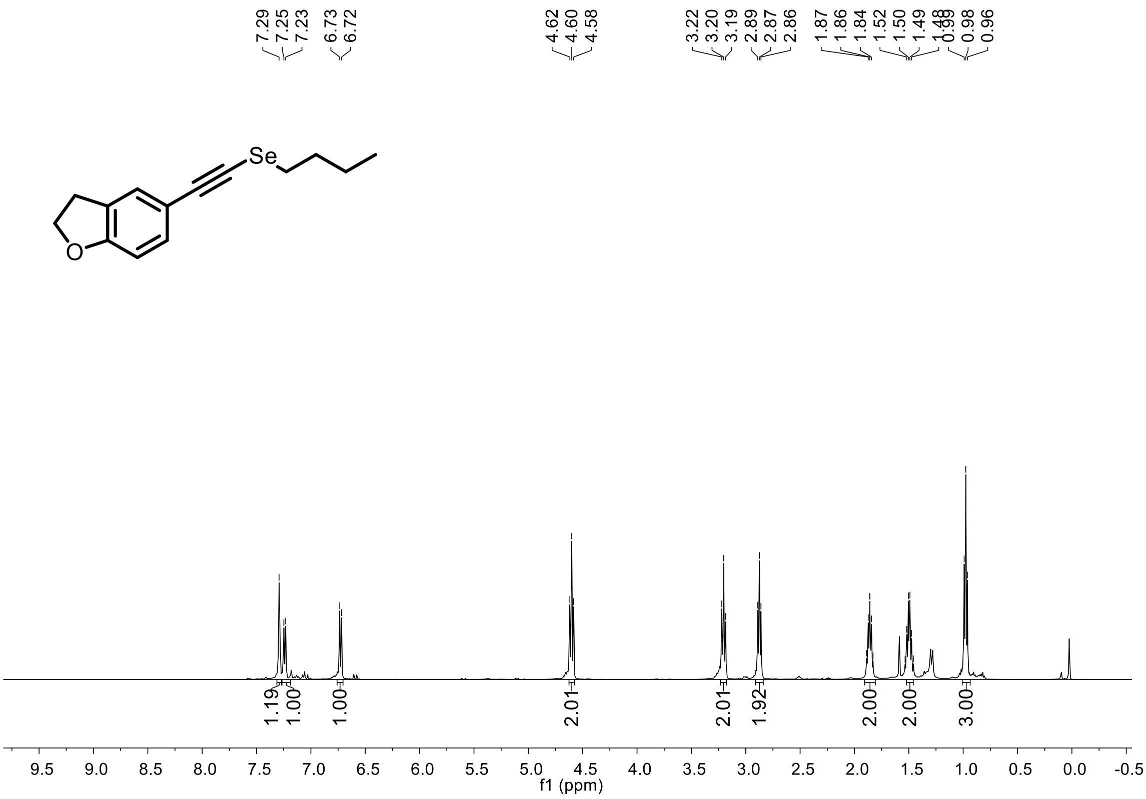

126 MHz, 298 K, $CDCl_3$ as solvent

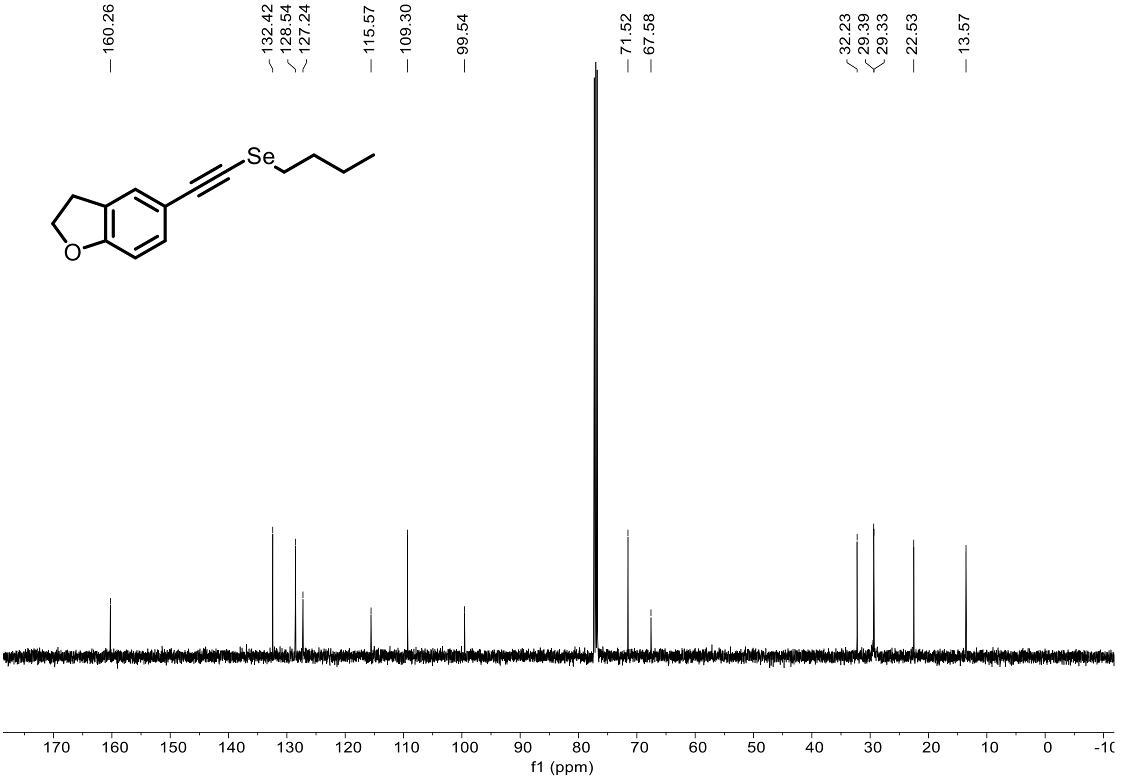

500 MHz, 298 K, CDCl$_3$ as solvent

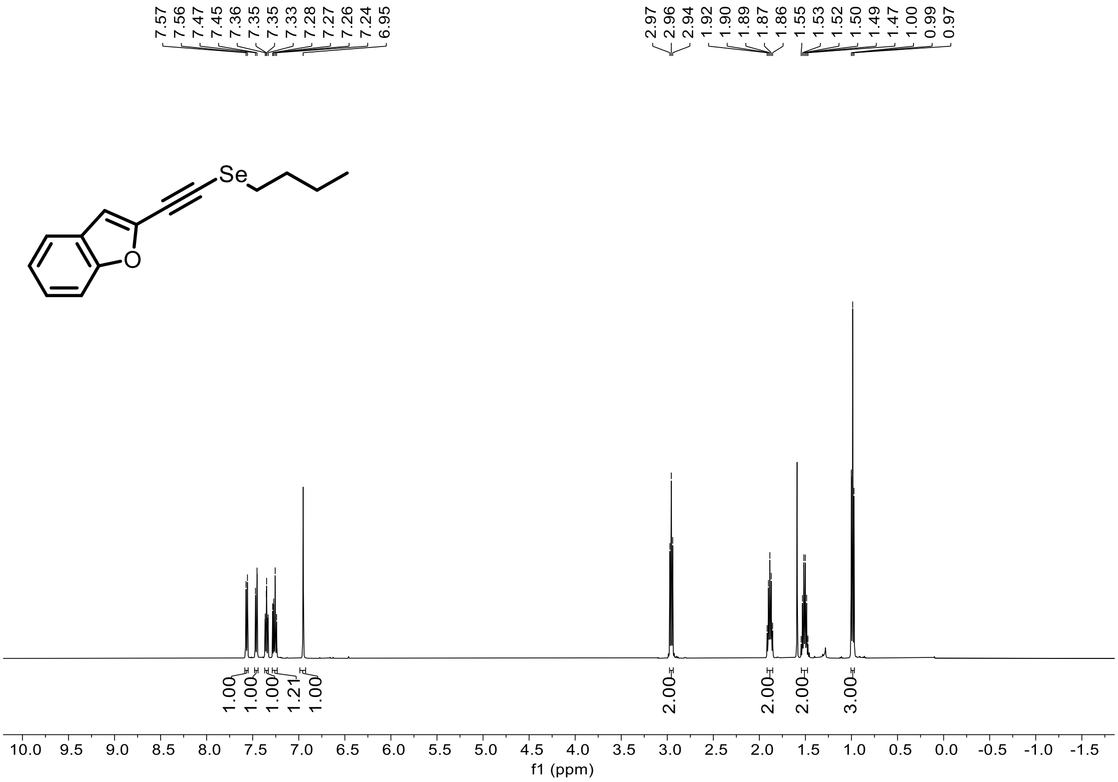

126 MHz, 298 K, CDCl$_3$ as solvent

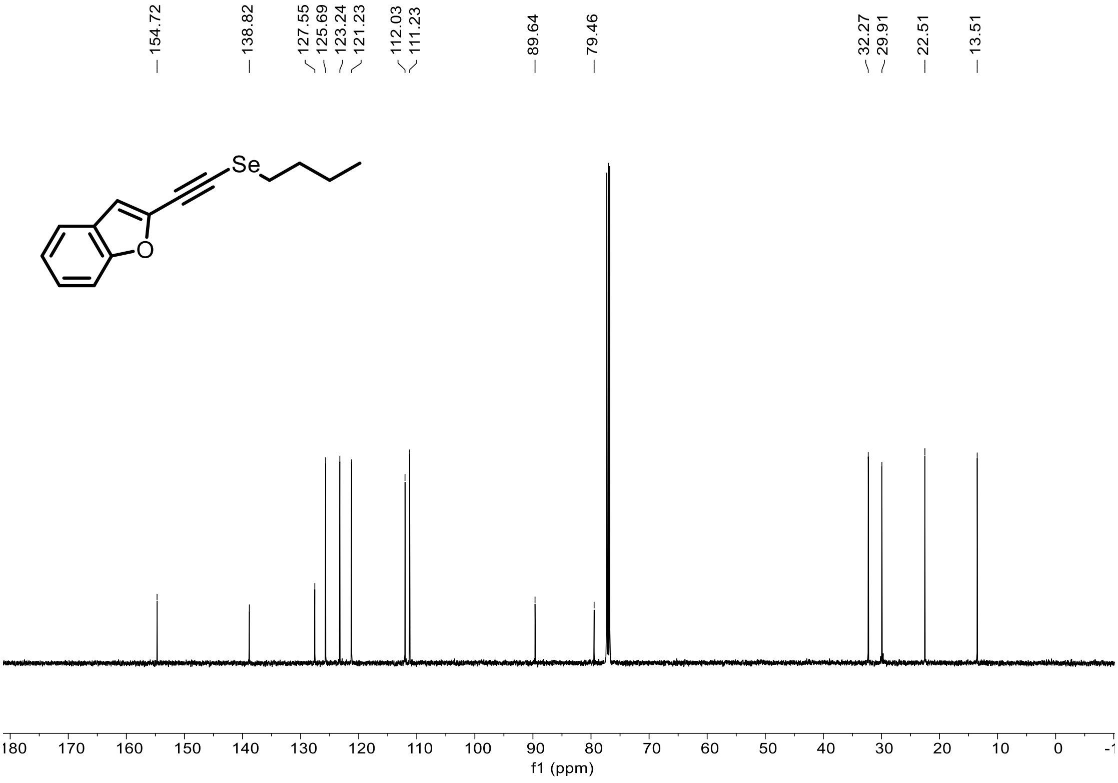

500 MHz, 298 K, $CDCl_3$ as solvent

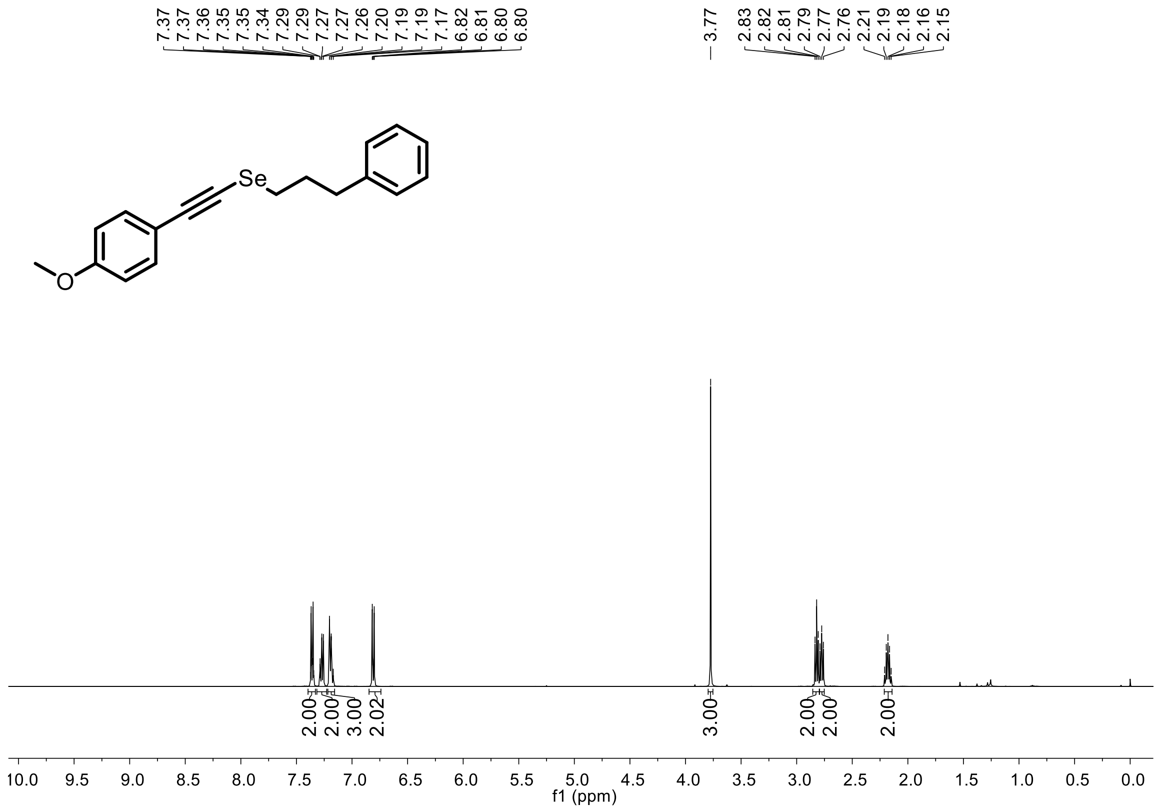

126 MHz, 298 K, $CDCl_3$ as solvent

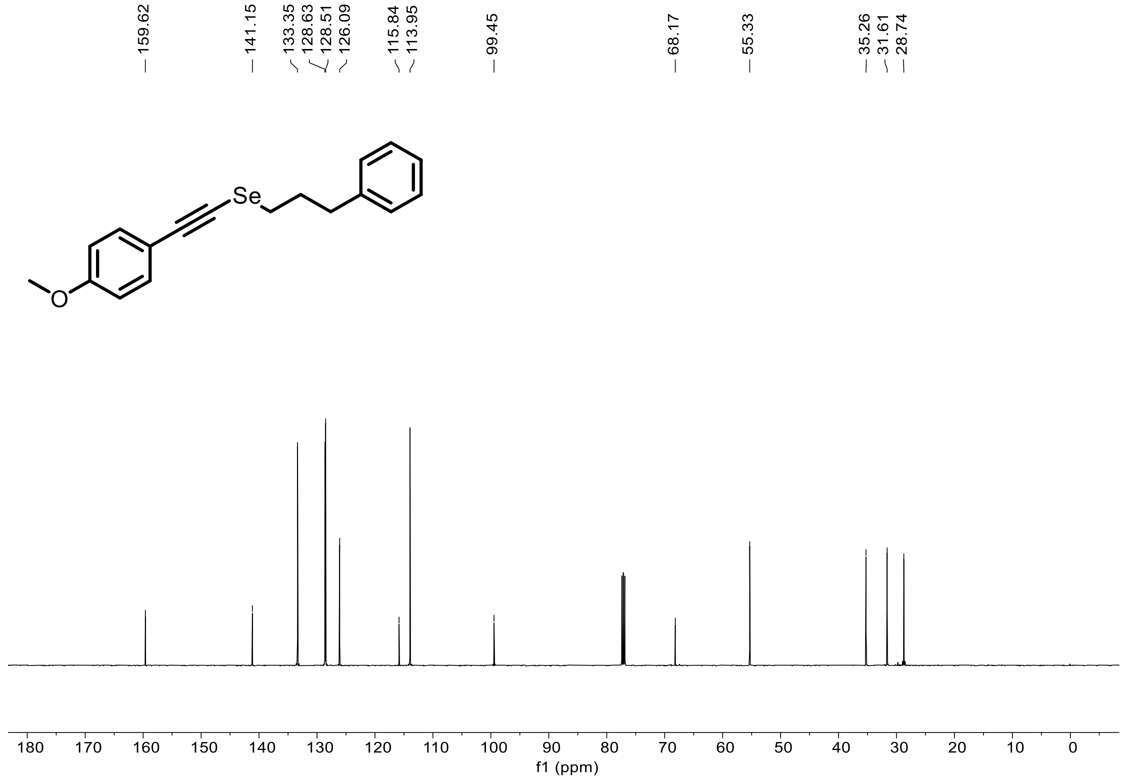

500 MHz, 298 K, CDCl3 as solvent

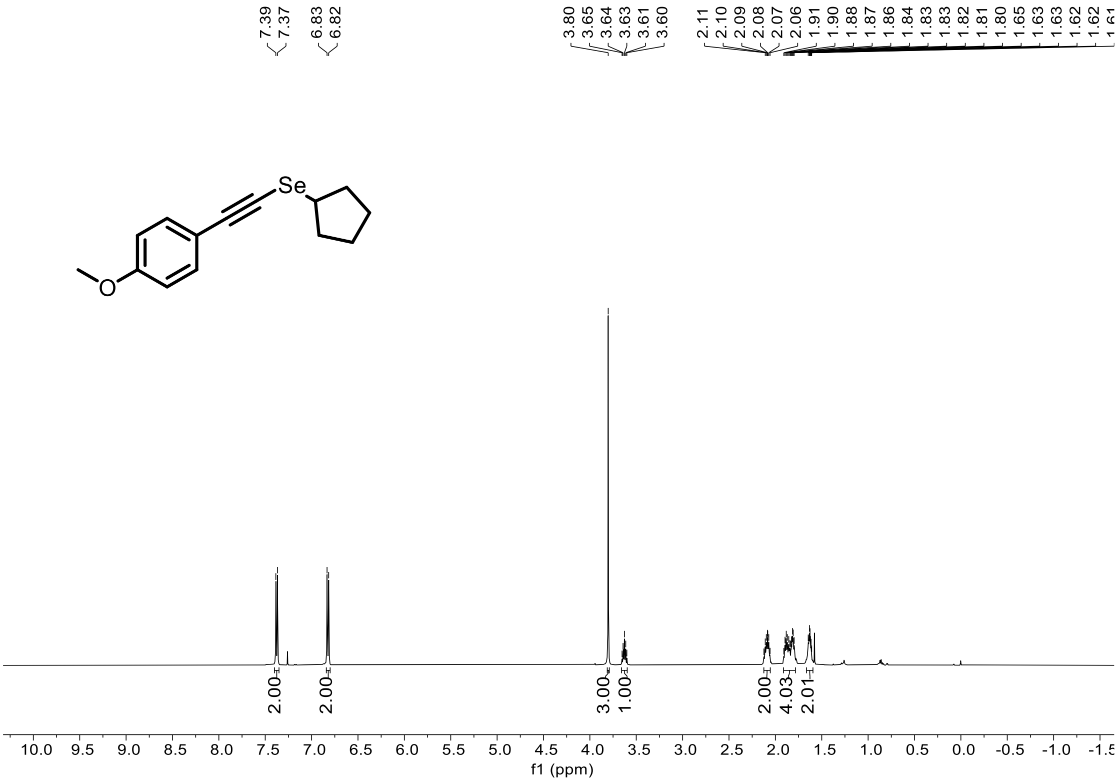

126 MHz, 298 K, CDCl3 as solvent

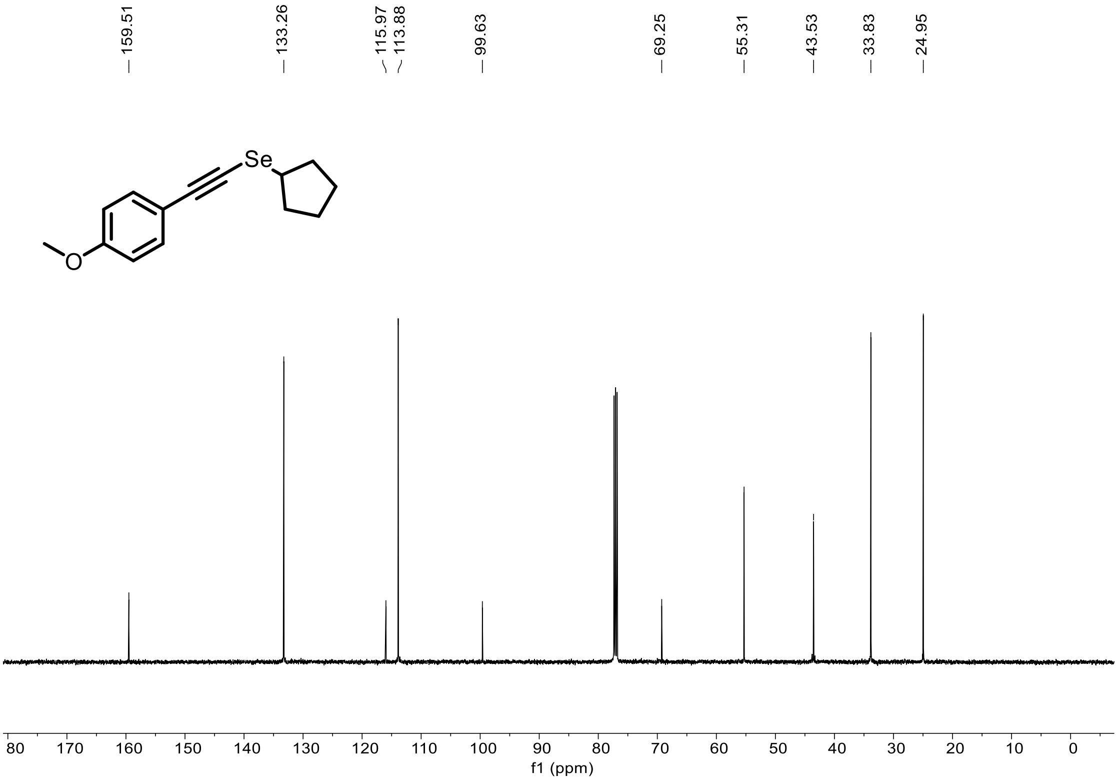

500 MHz, 298 K, $CDCl_3$ as solvent

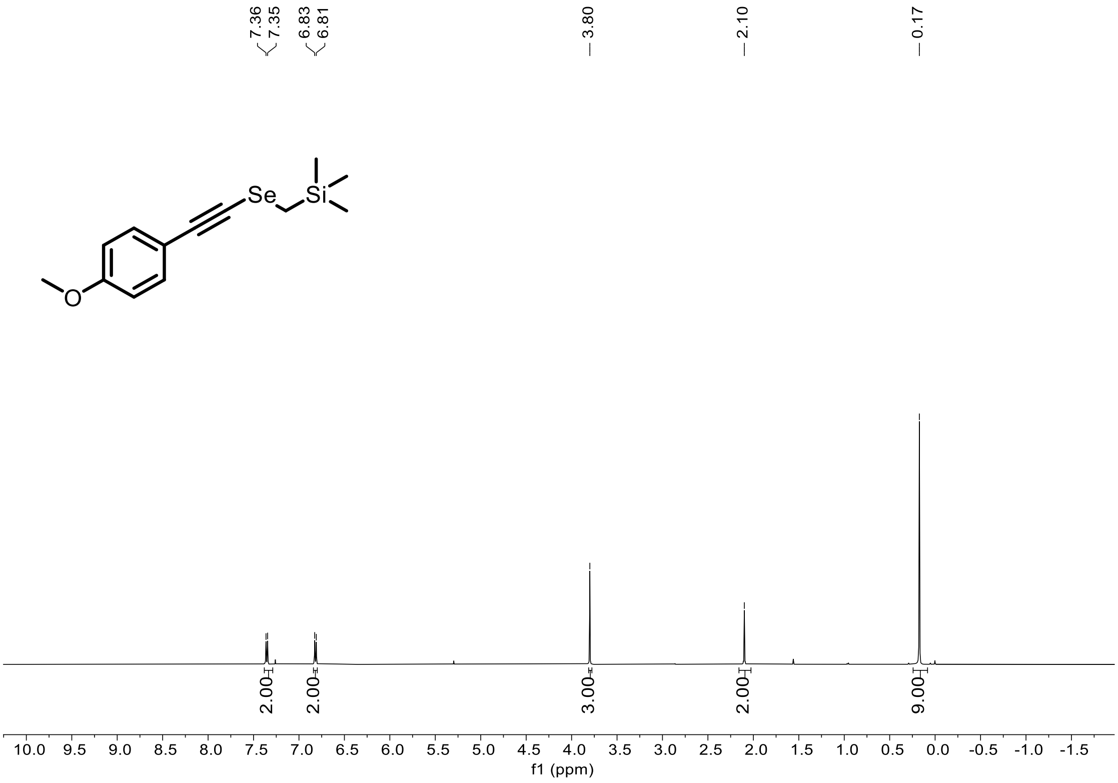

126 MHz, 298 K, $CDCl_3$ as solvent

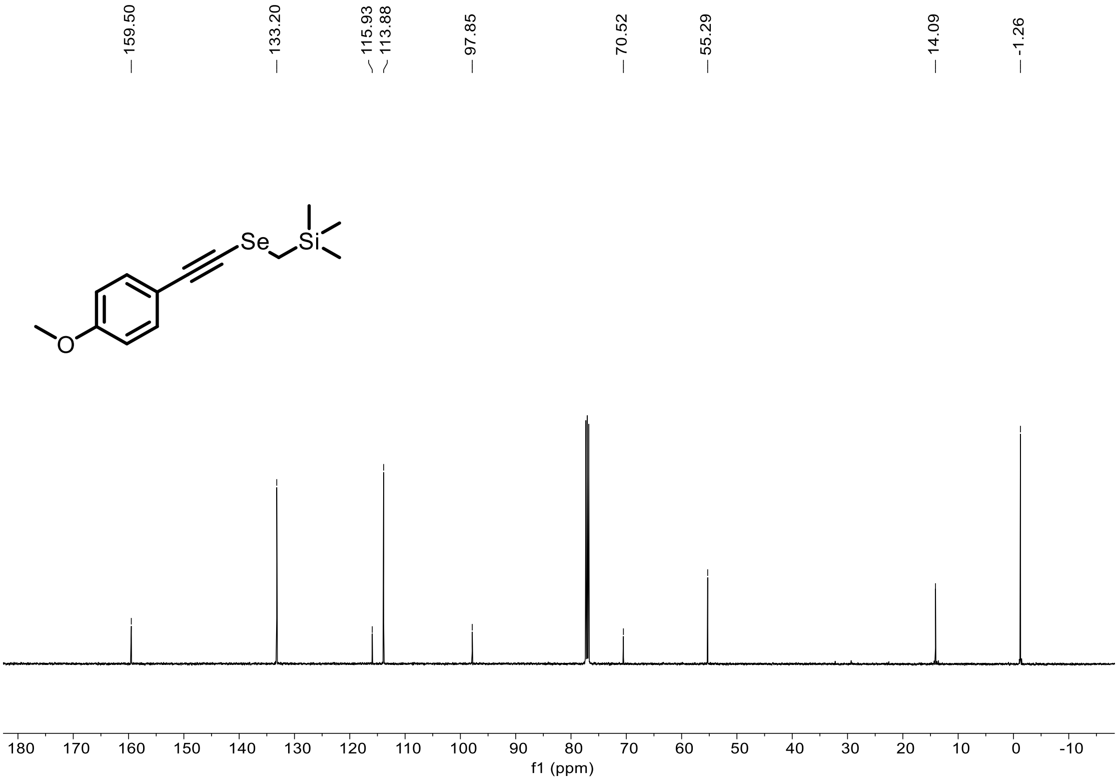

500 MHz, 298 K, $CDCl_3$ as solvent

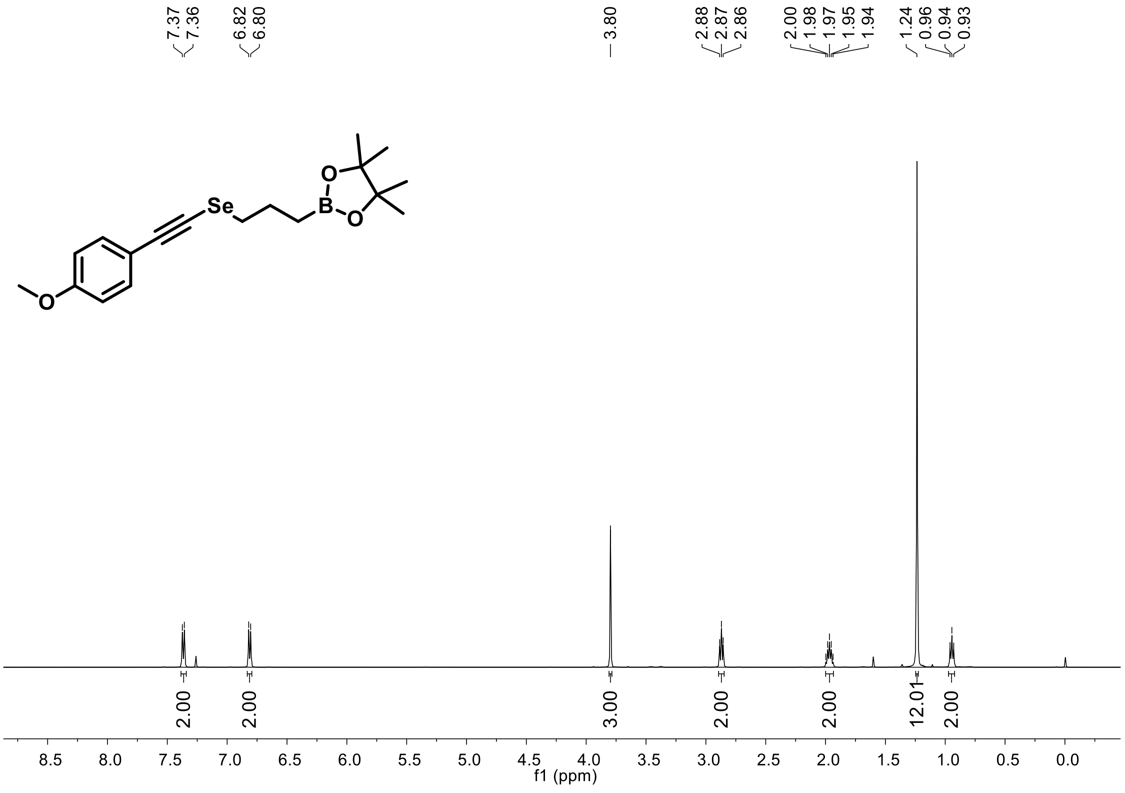

126 MHz, 298 K, $CDCl_3$ as solvent

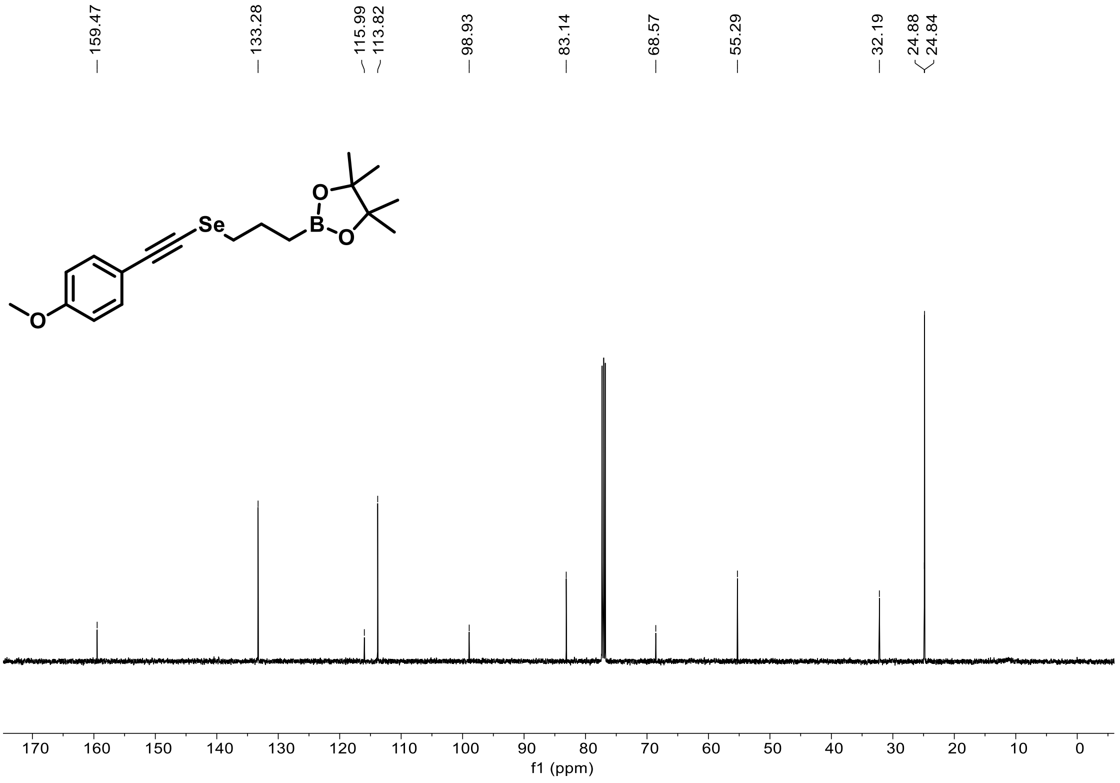

500 MHz, 298 K, CDCl3 as solvent

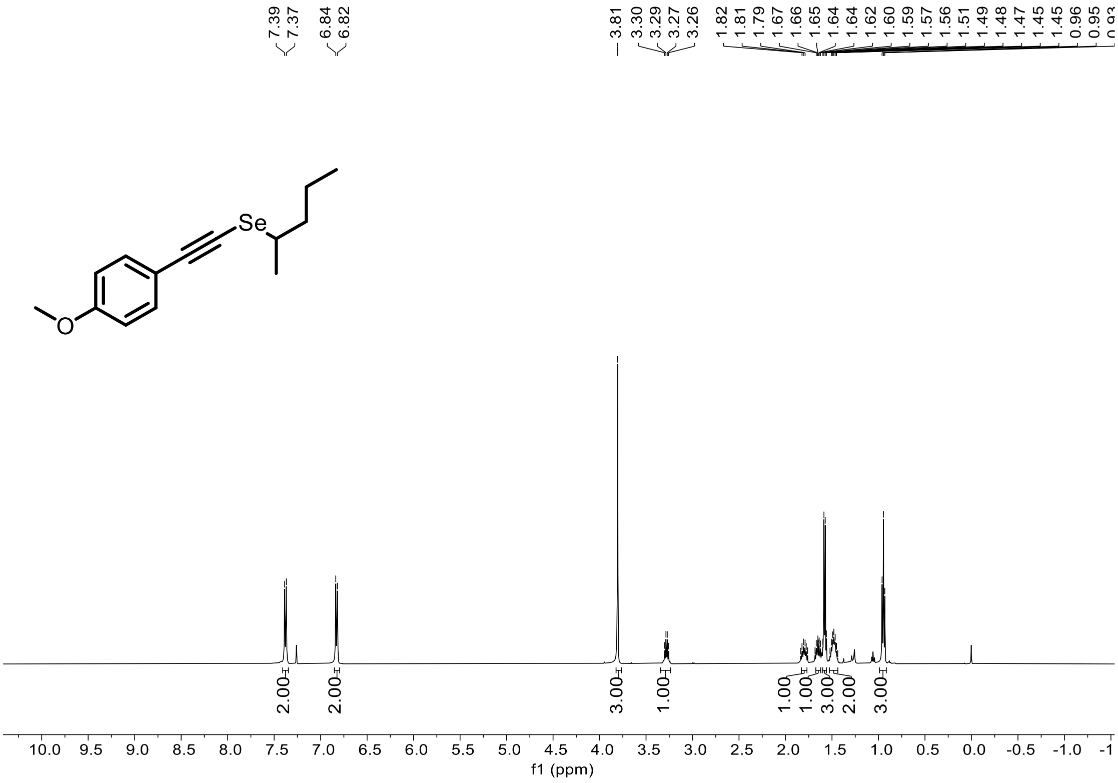

126 MHz, 298 K, CDCl3 as solvent

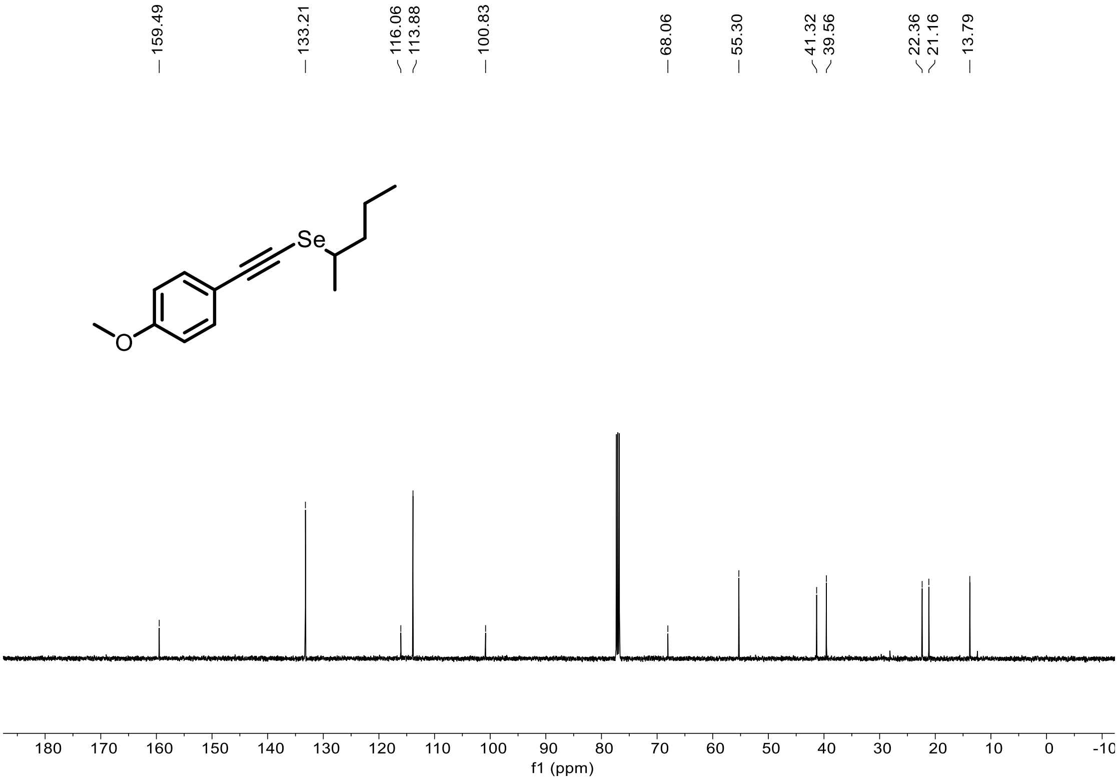

500 MHz, 298 K, $CDCl_3$ as solvent

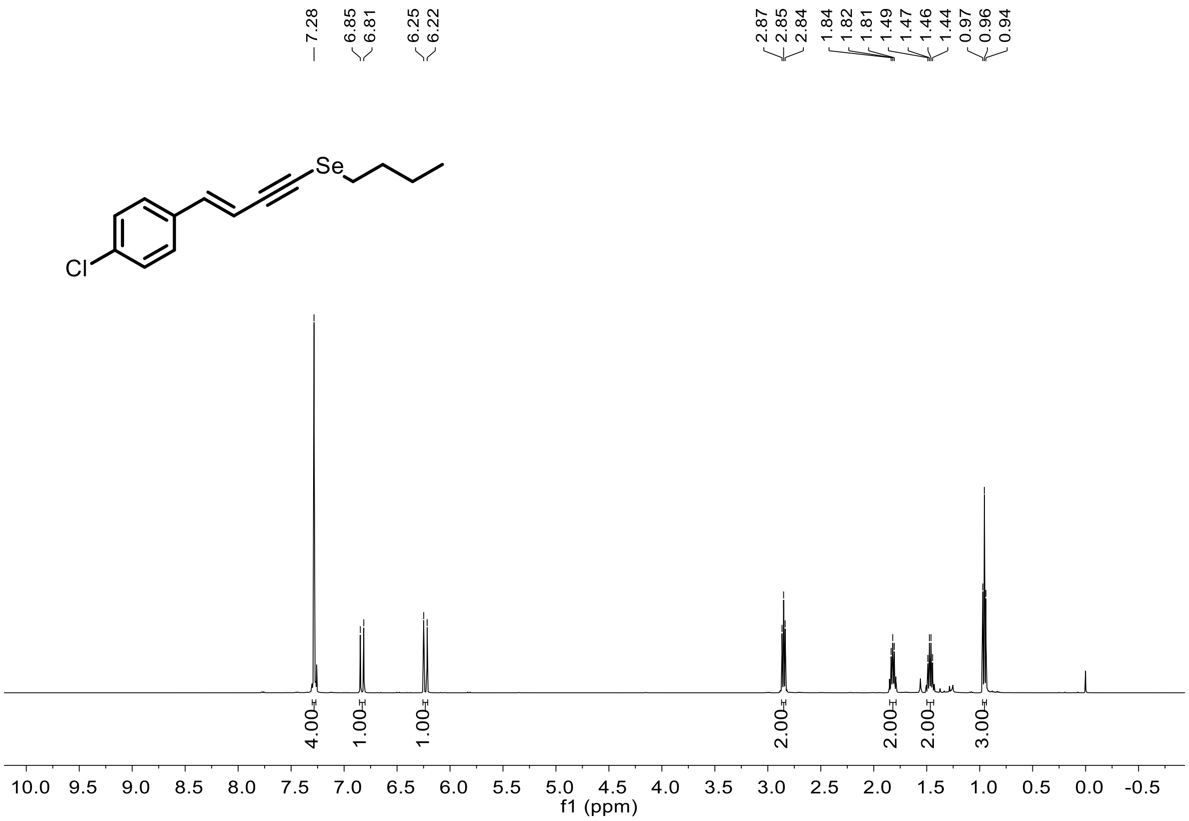

126 MHz, 298 K, $CDCl_3$ as solvent

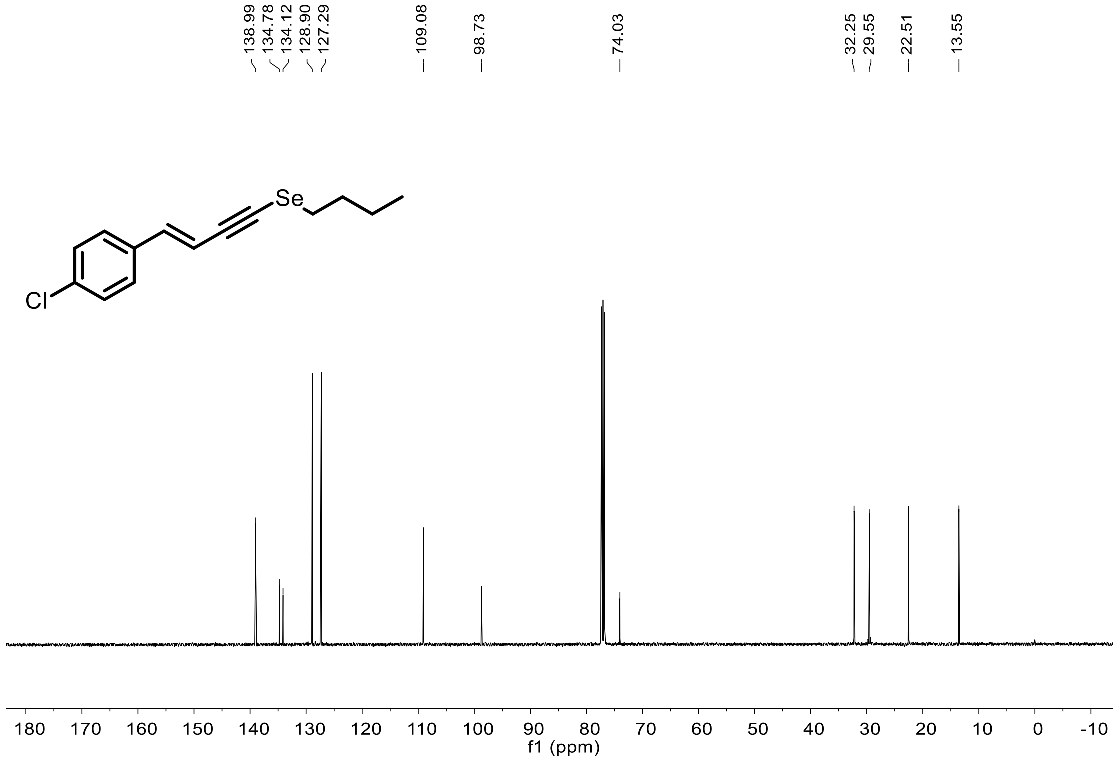

500 MHz, 298 K, $CDCl_3$ as solvent

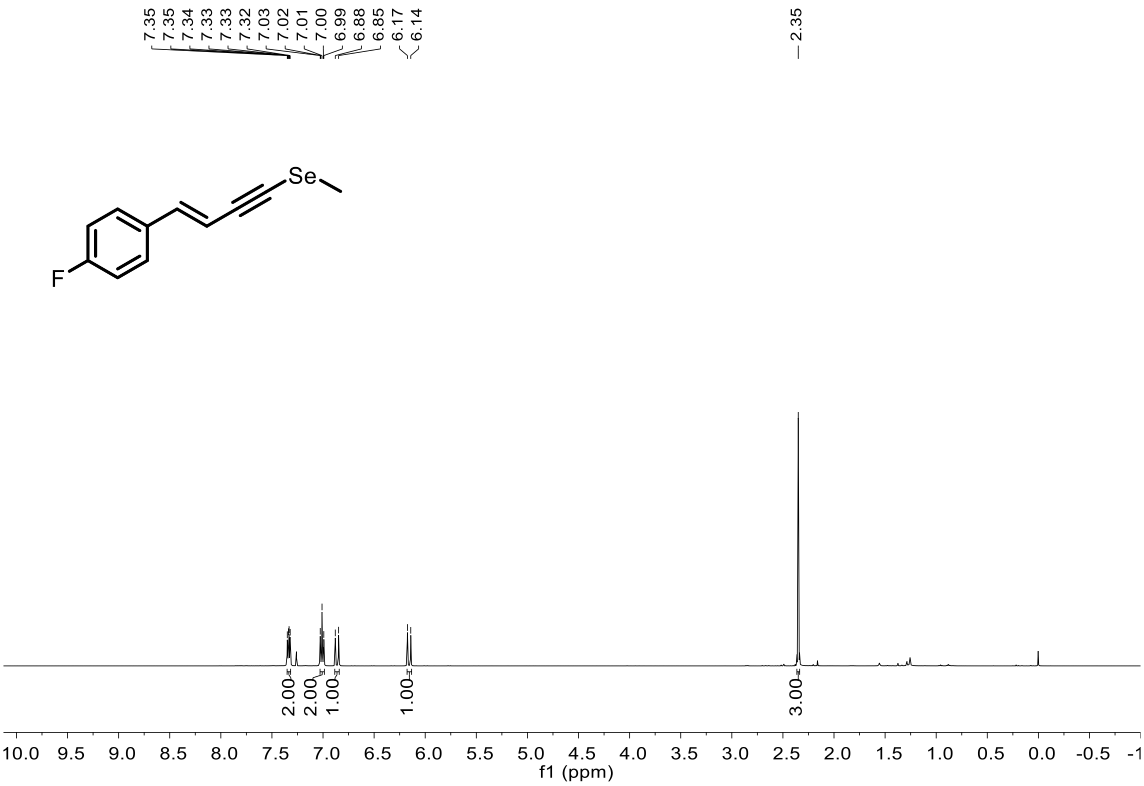

126 MHz, 298 K, $CDCl_3$ as solvent

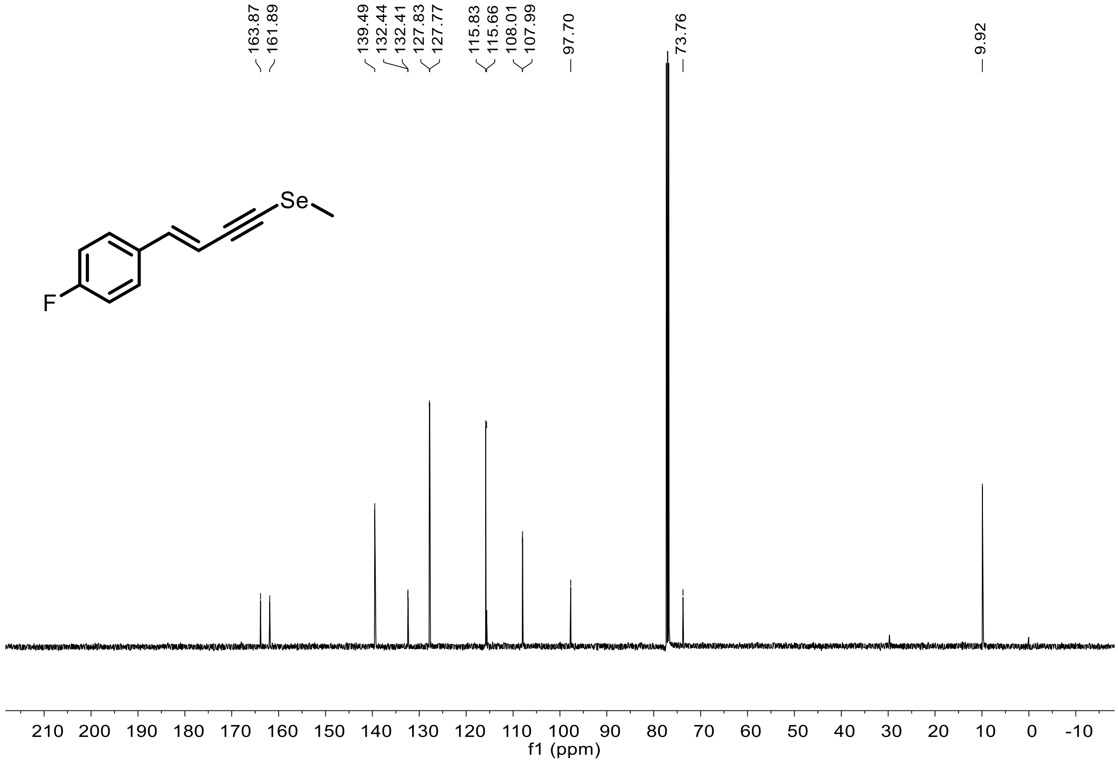

500 MHz, 298 K, $CDCl_3$ as solvent

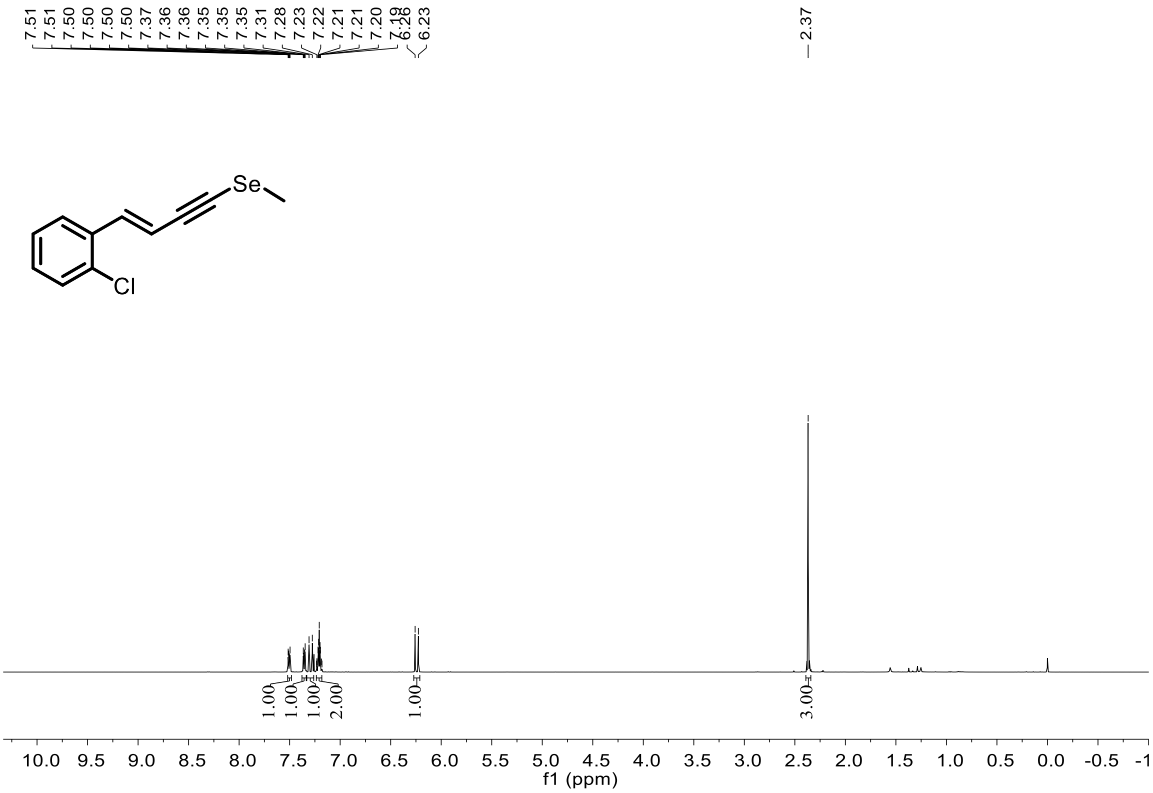

126 MHz, 298 K, $CDCl_3$ as solvent

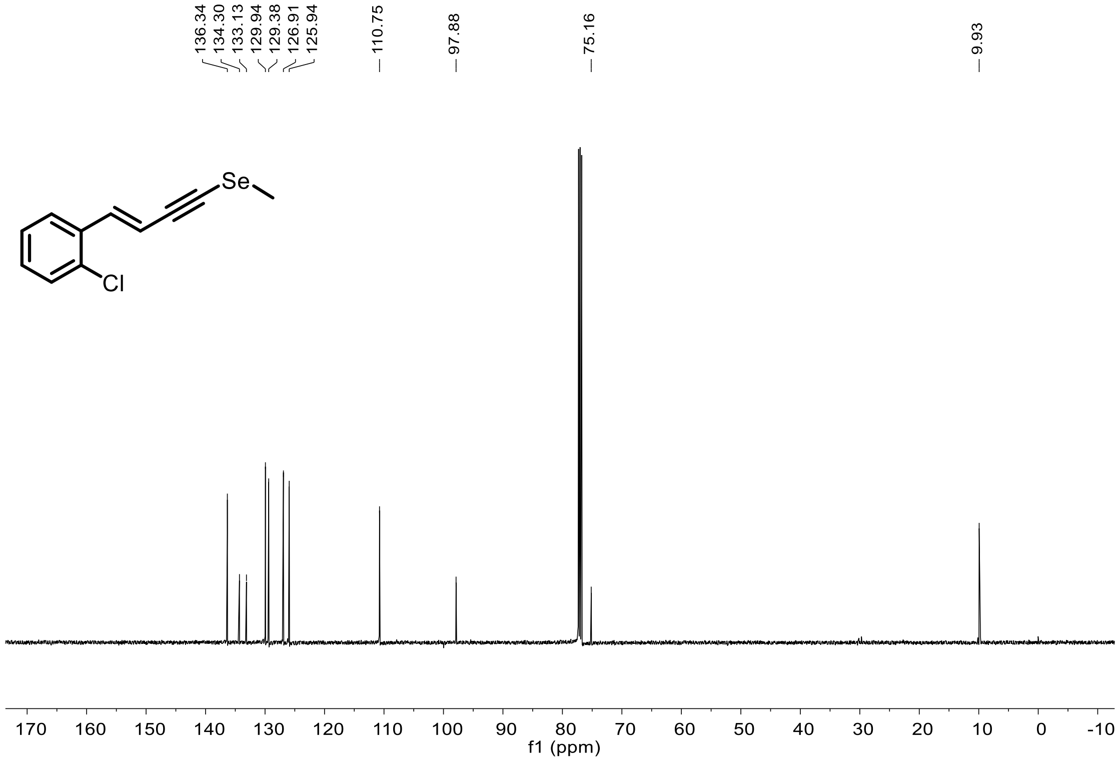

500 MHz, 298 K, CDCl3 as solvent

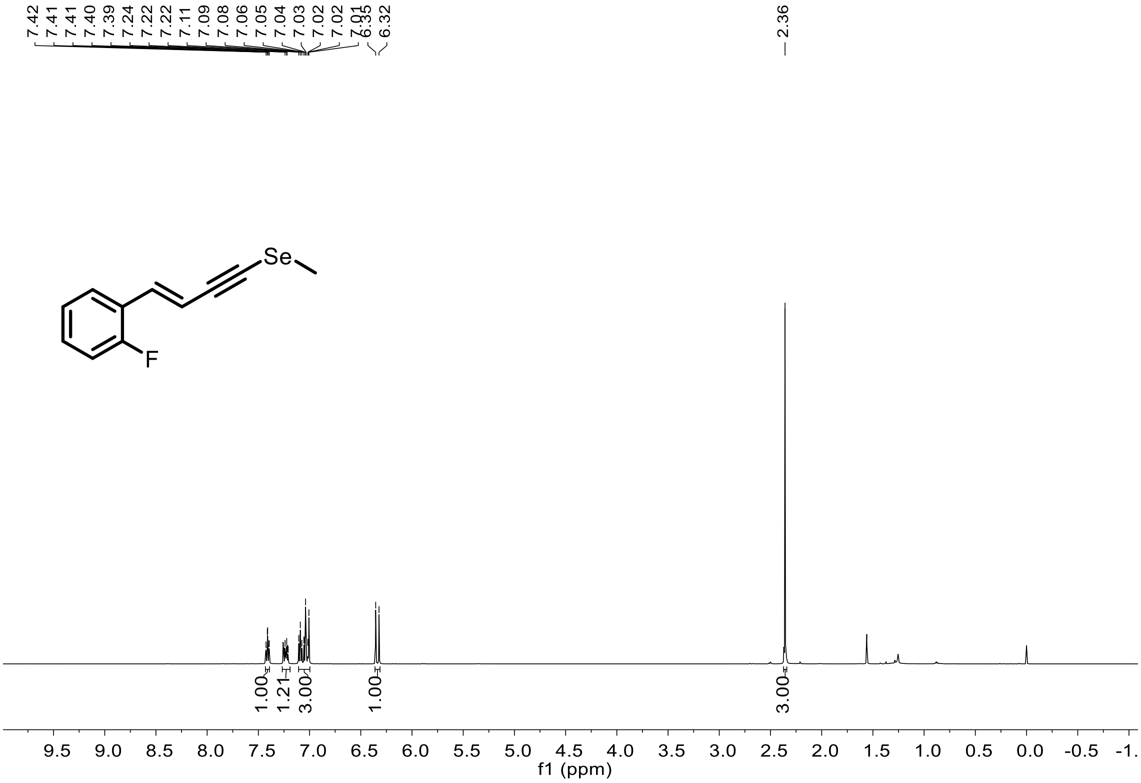

126 MHz, 298 K, CDCl3 as solvent

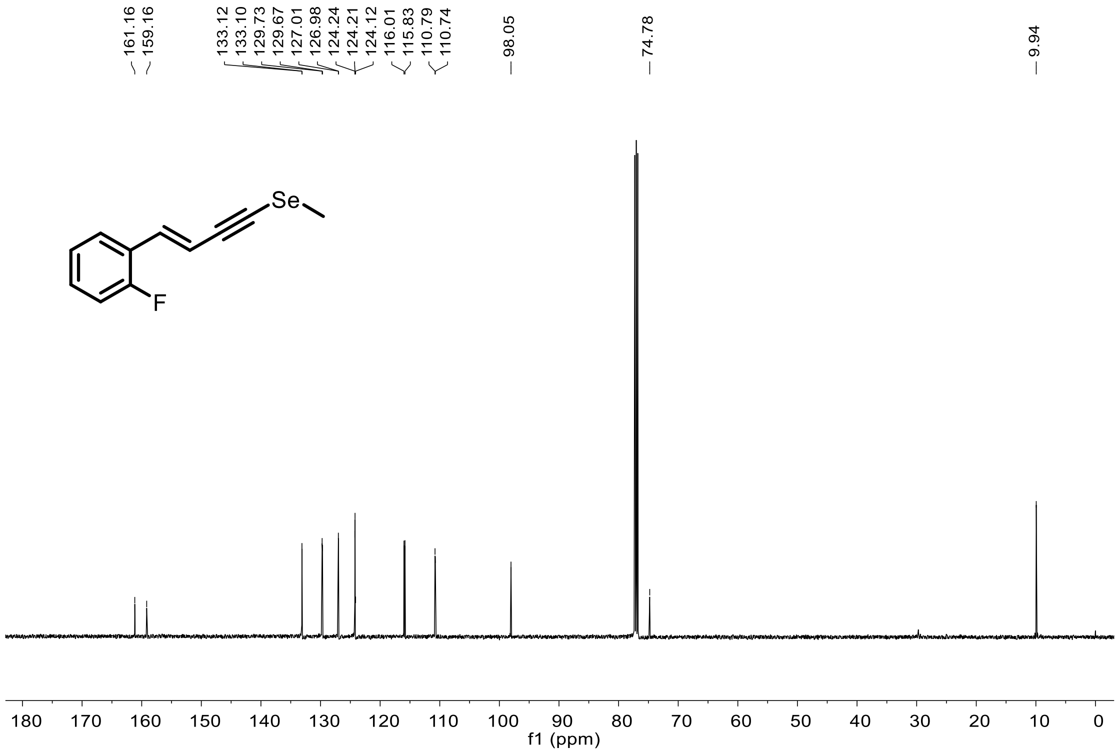

500 MHz, 298 K, $CDCl_3$ as solvent

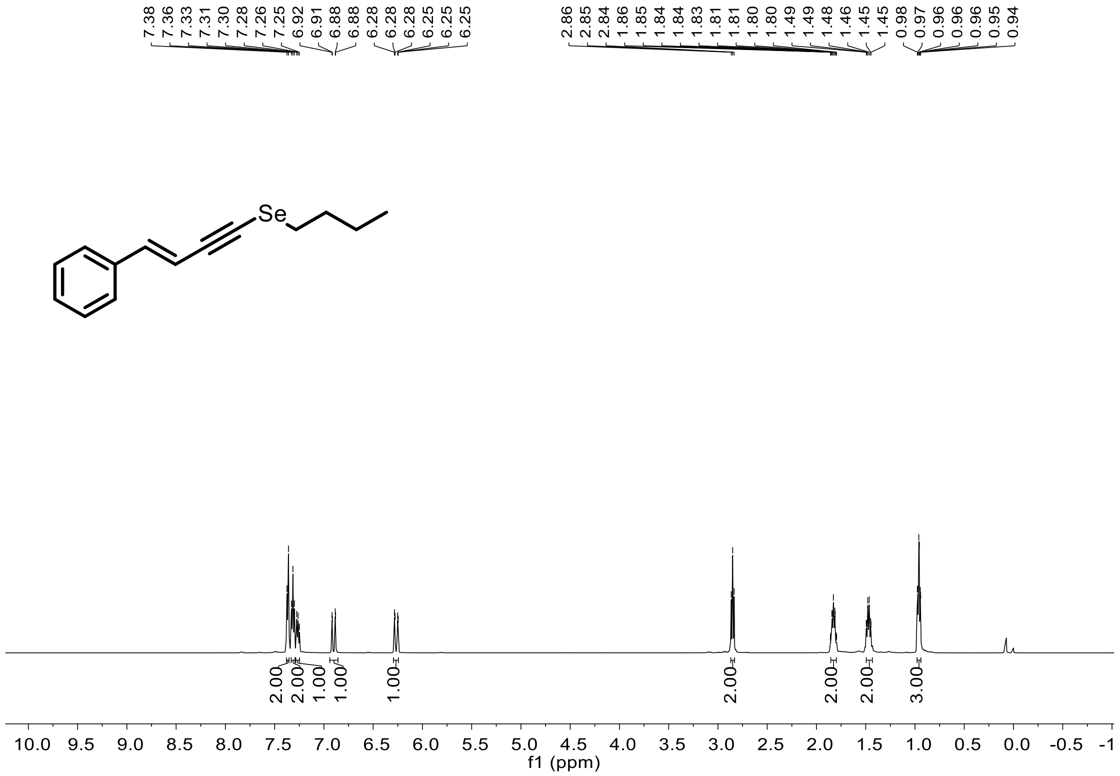

126 MHz, 298 K, $CDCl_3$ as solvent

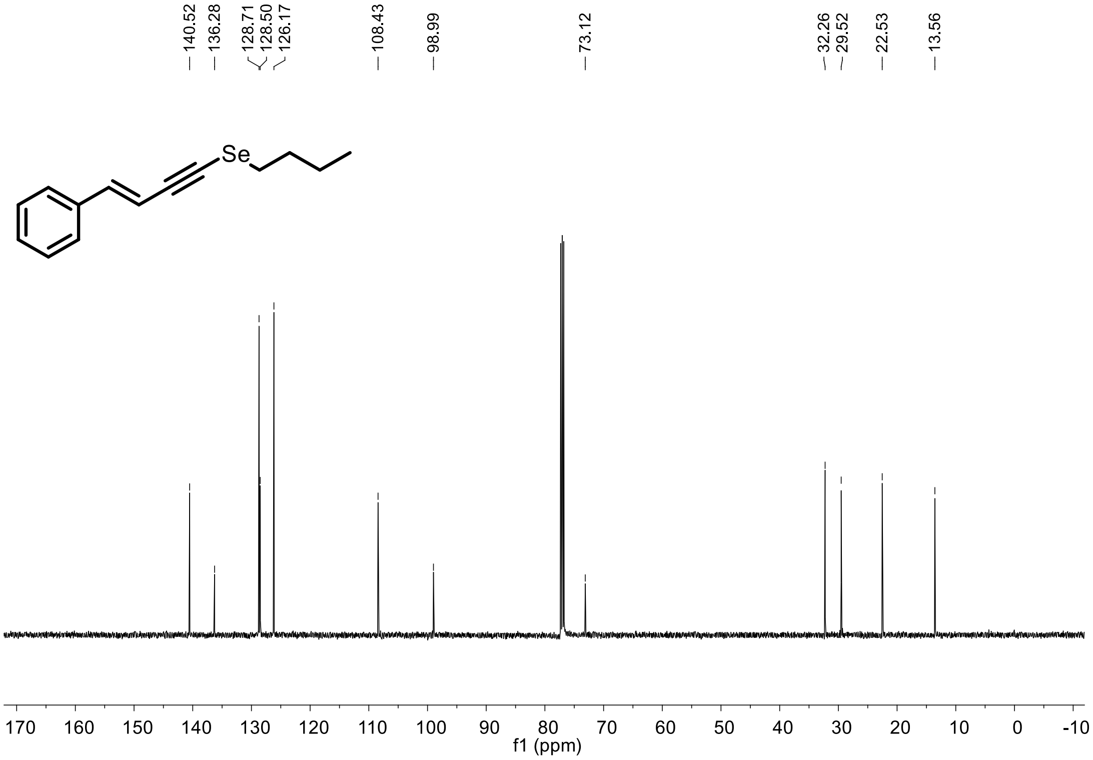

500 MHz, 298 K, $CDCl_3$ as solvent

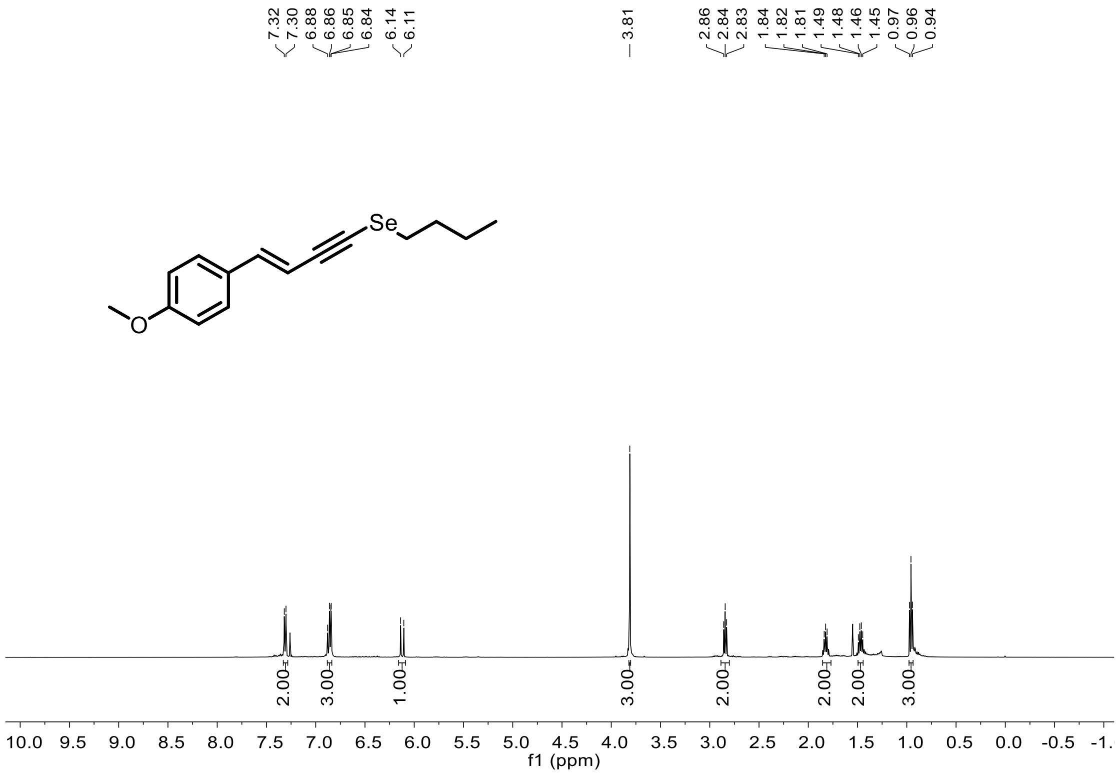

126 MHz, 298 K, $CDCl_3$ as solvent

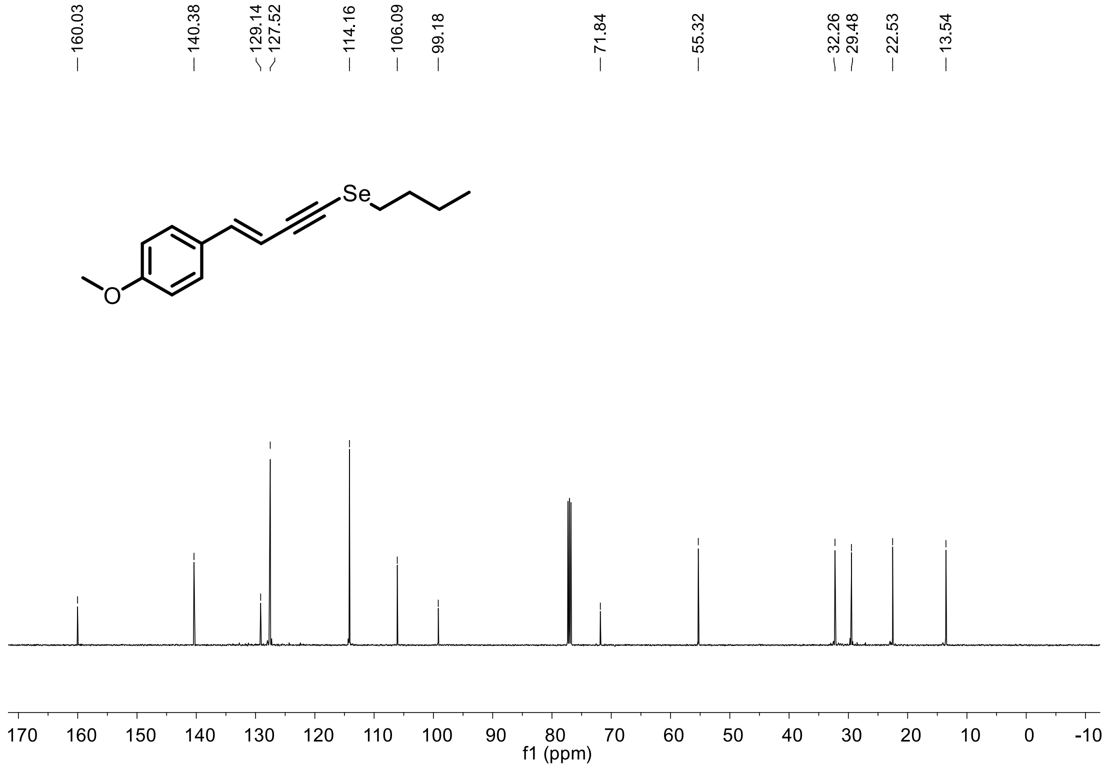

500 MHz, 298 K, $CDCl_3$ as solvent

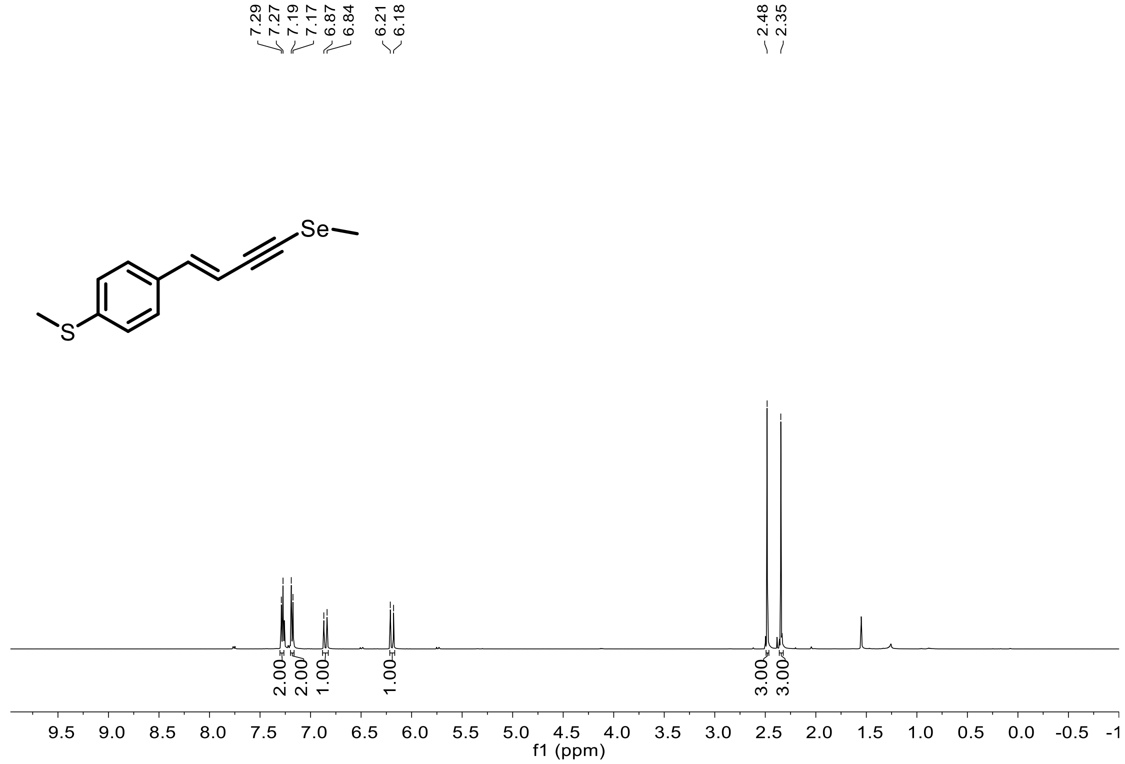

126 MHz, 298 K, $CDCl_3$ as solvent

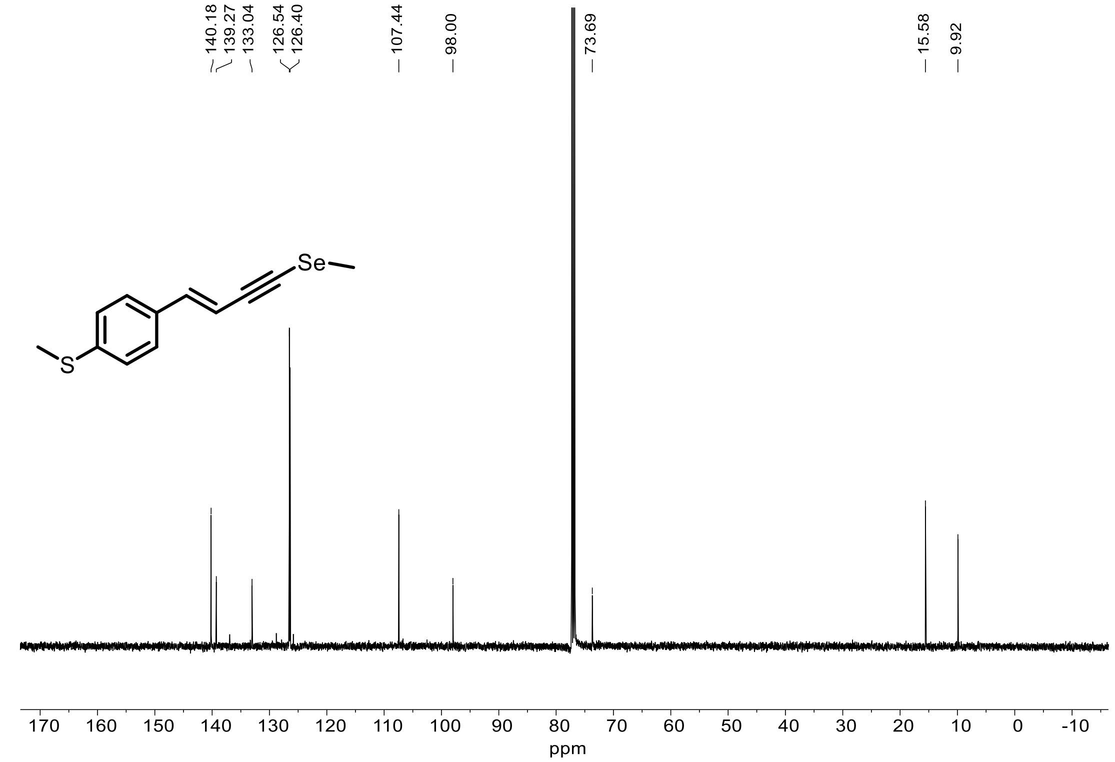

500 MHz, 298 K, $CDCl_3$ as solvent

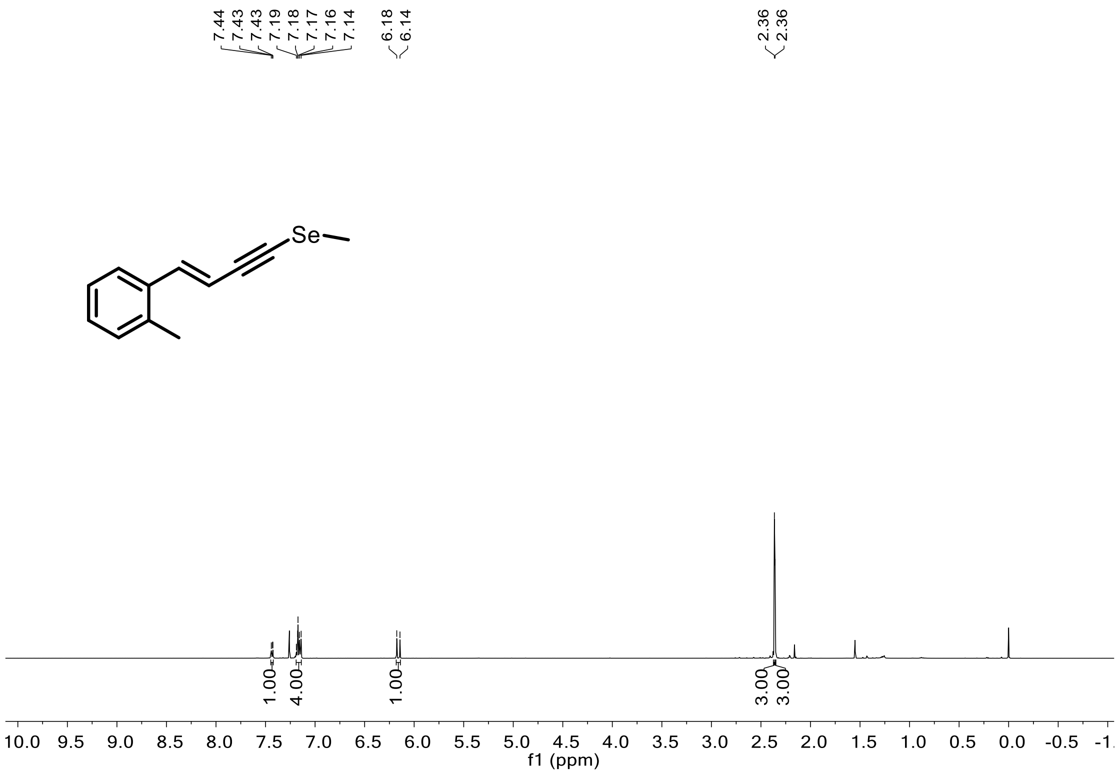

126 MHz, 298 K, $CDCl_3$ as solvent

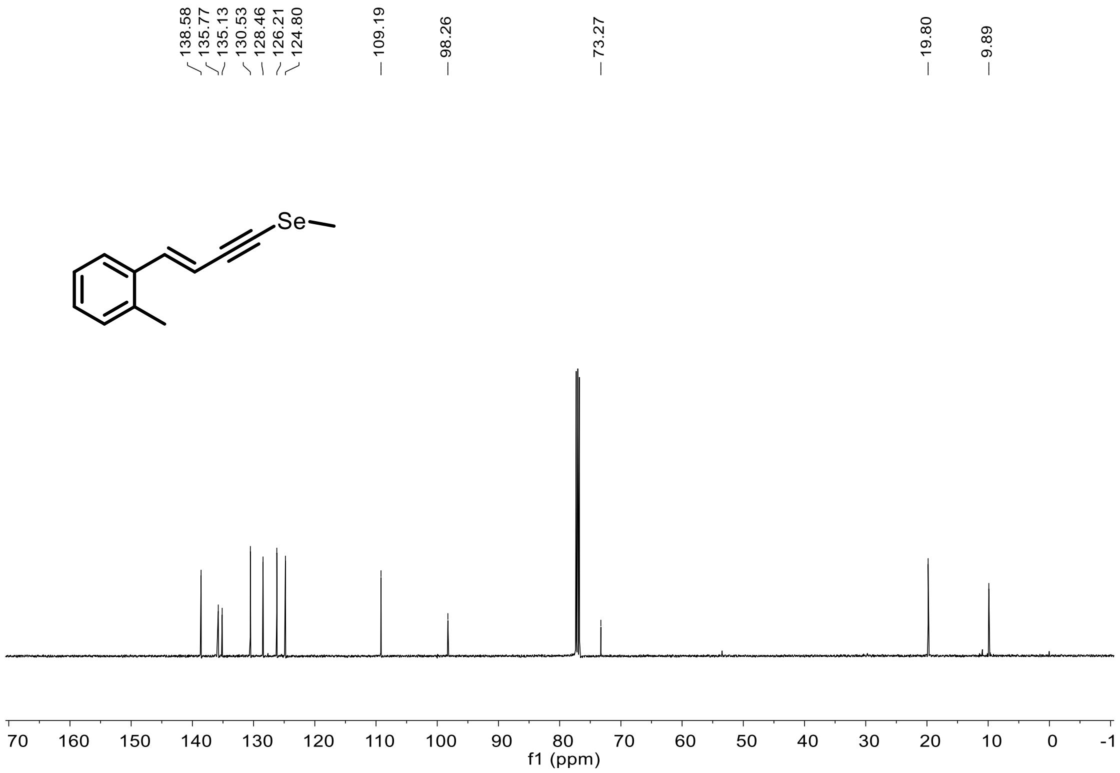

500 MHz, 298 K, $CDCl_3$ as solvent

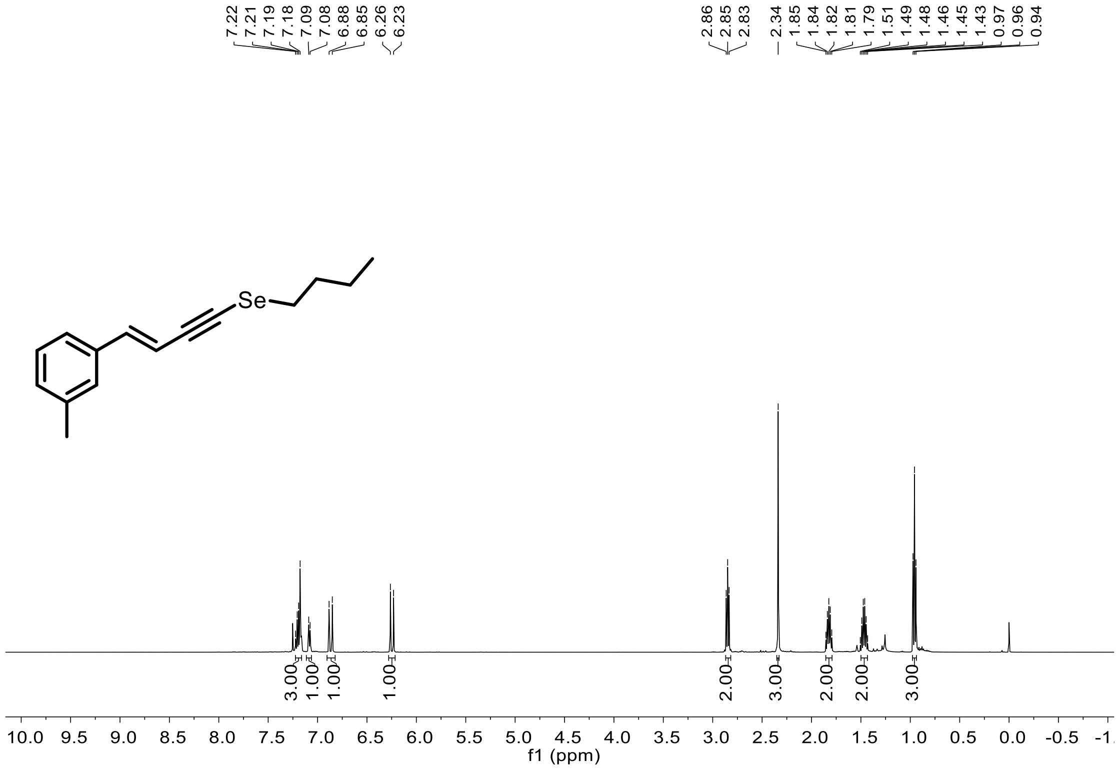

126 MHz, 298 K, $CDCl_3$ as solvent

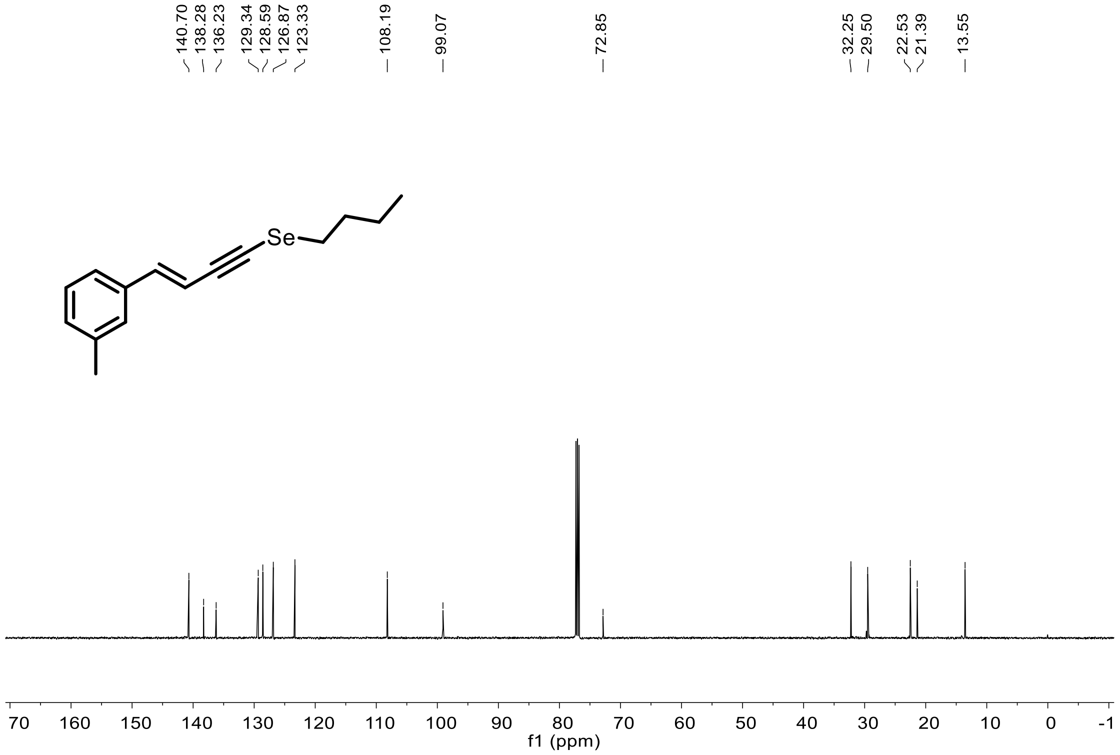

500 MHz, 298 K, CDCl3 as solvent

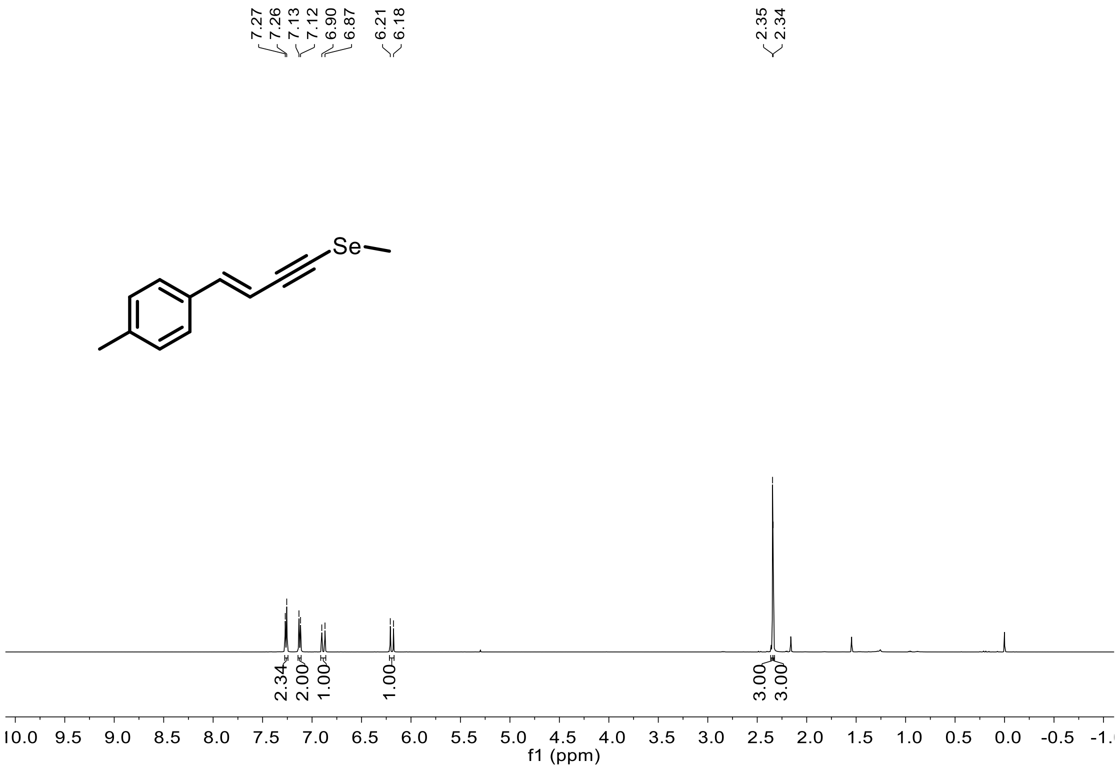

126 MHz, 298 K, CDCl3 as solvent

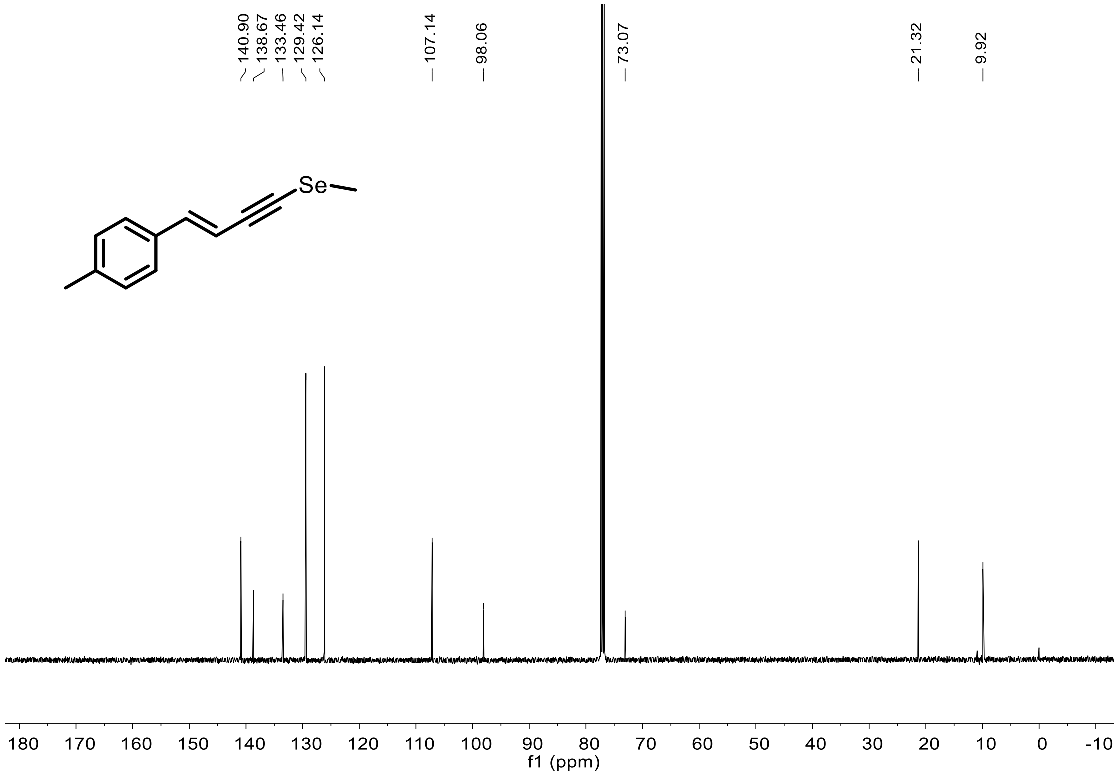

500 MHz, 298 K, CDCl3 as solvent

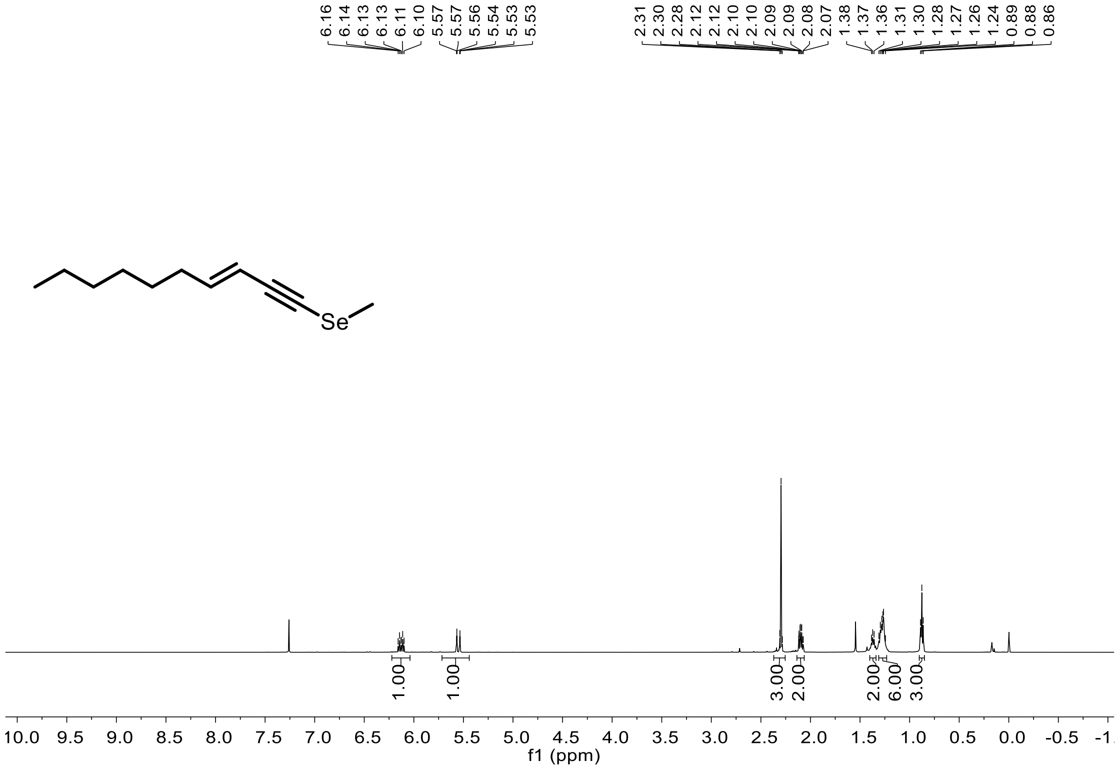

126 MHz, 298 K, CDCl3 as solvent

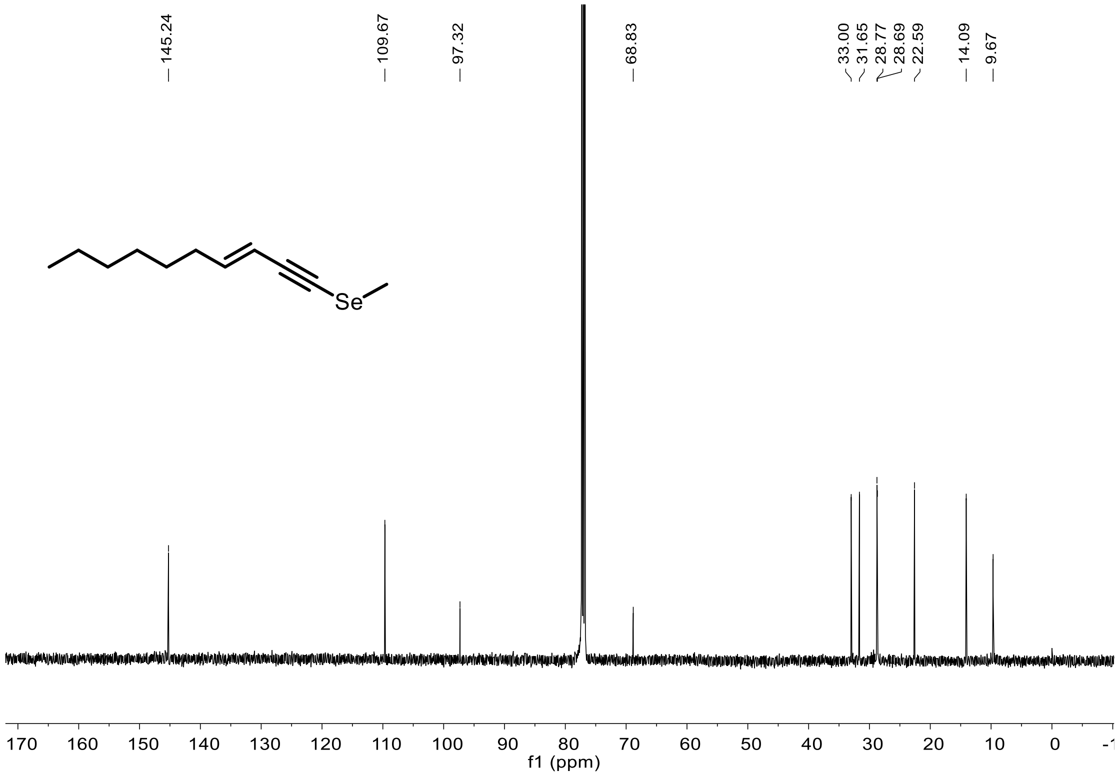

500 MHz, 298 K, $CDCl_3$ as solvent

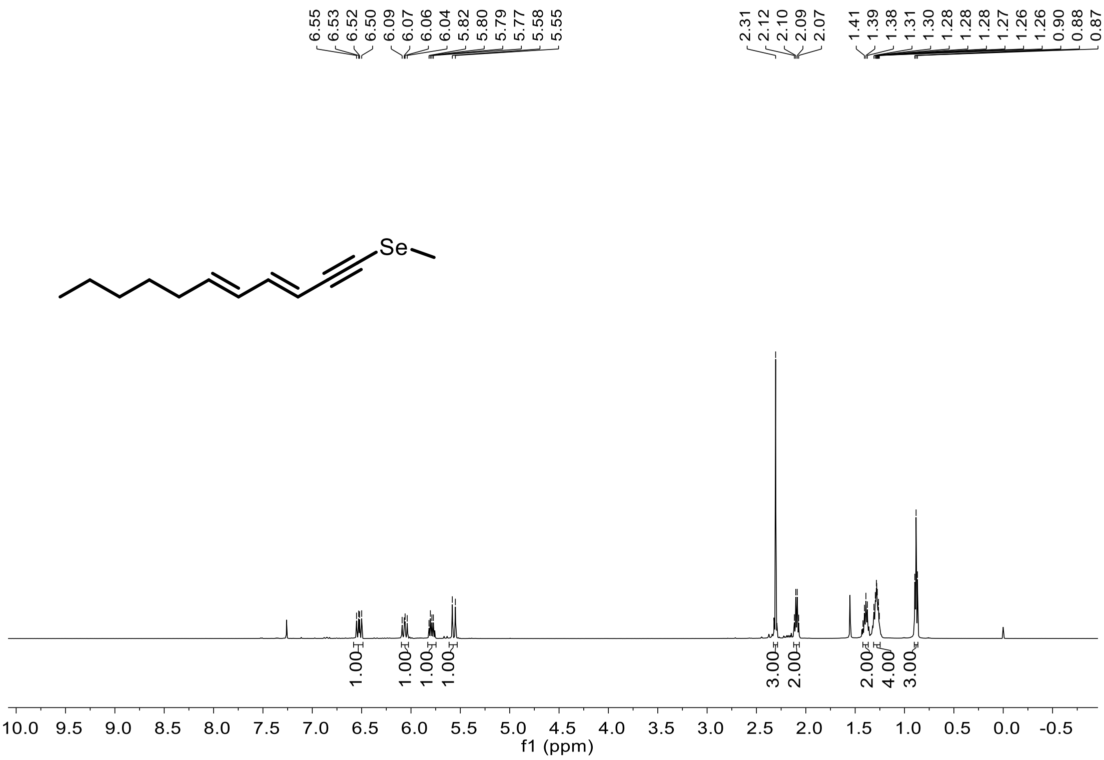

126 MHz, 298 K, $CDCl_3$ as solvent

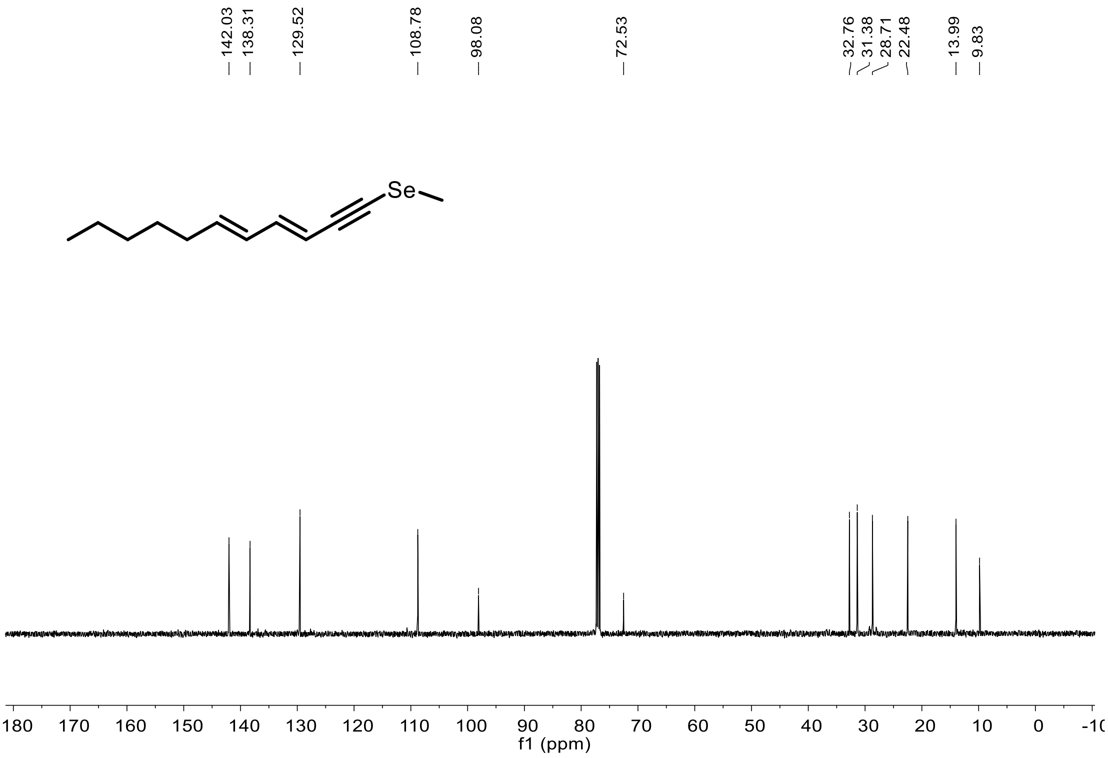

500 MHz, 298 K, CDCl3 as solvent

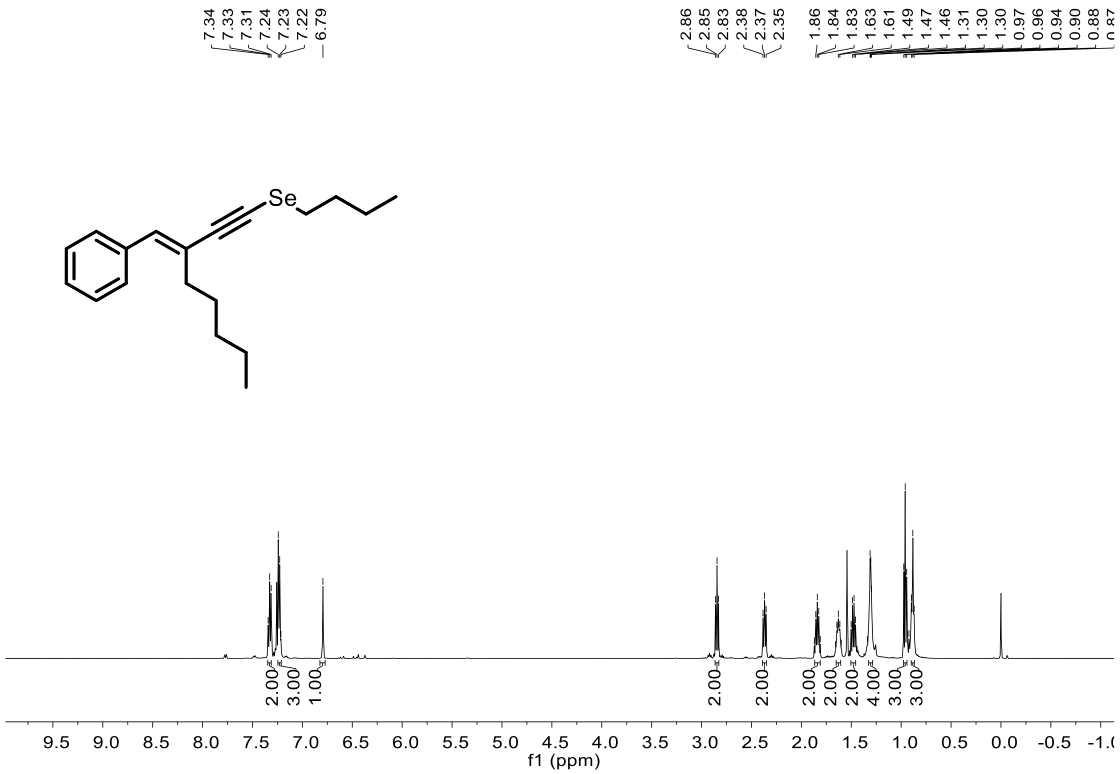

126 MHz, 298 K, CDCl3 as solvent

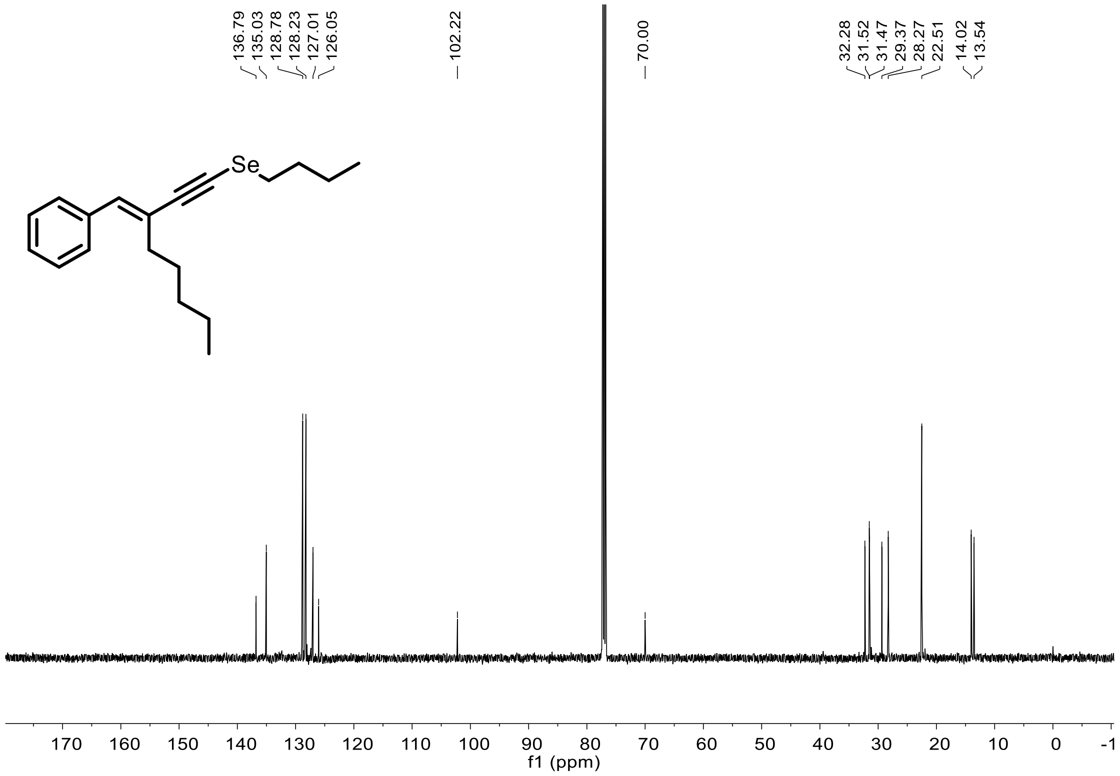

500 MHz, 298 K, $CDCl_3$ as solvent

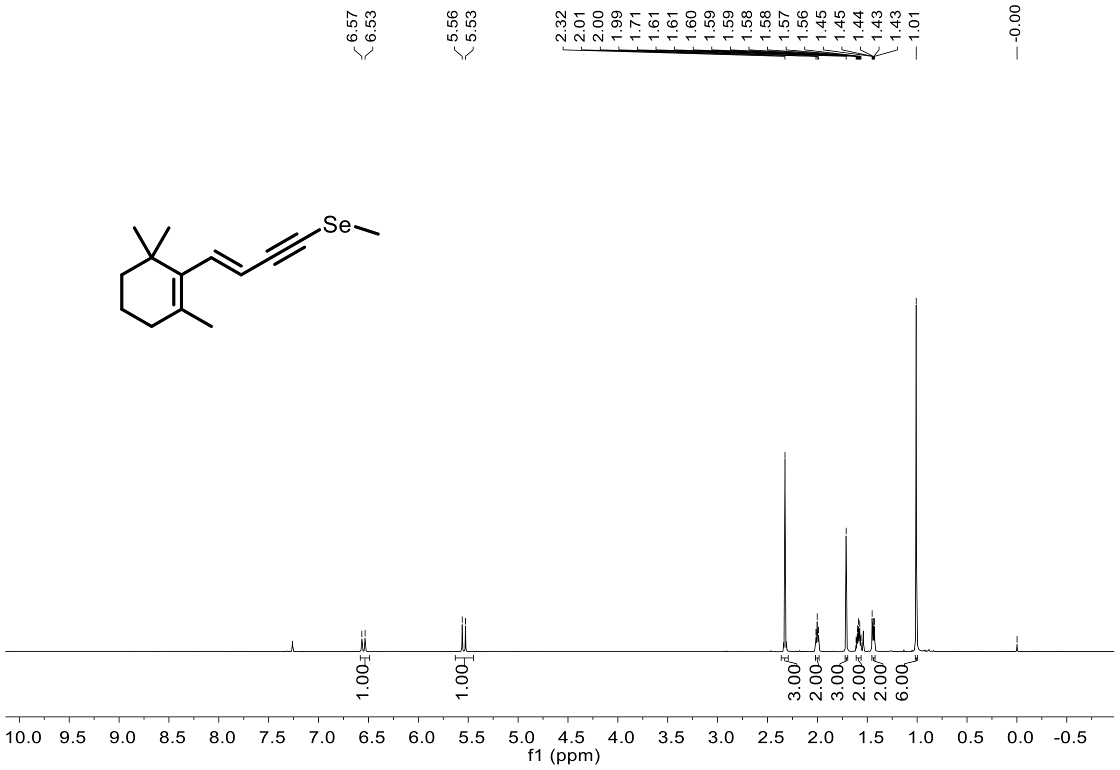

126 MHz, 298 K, $CDCl_3$ as solvent

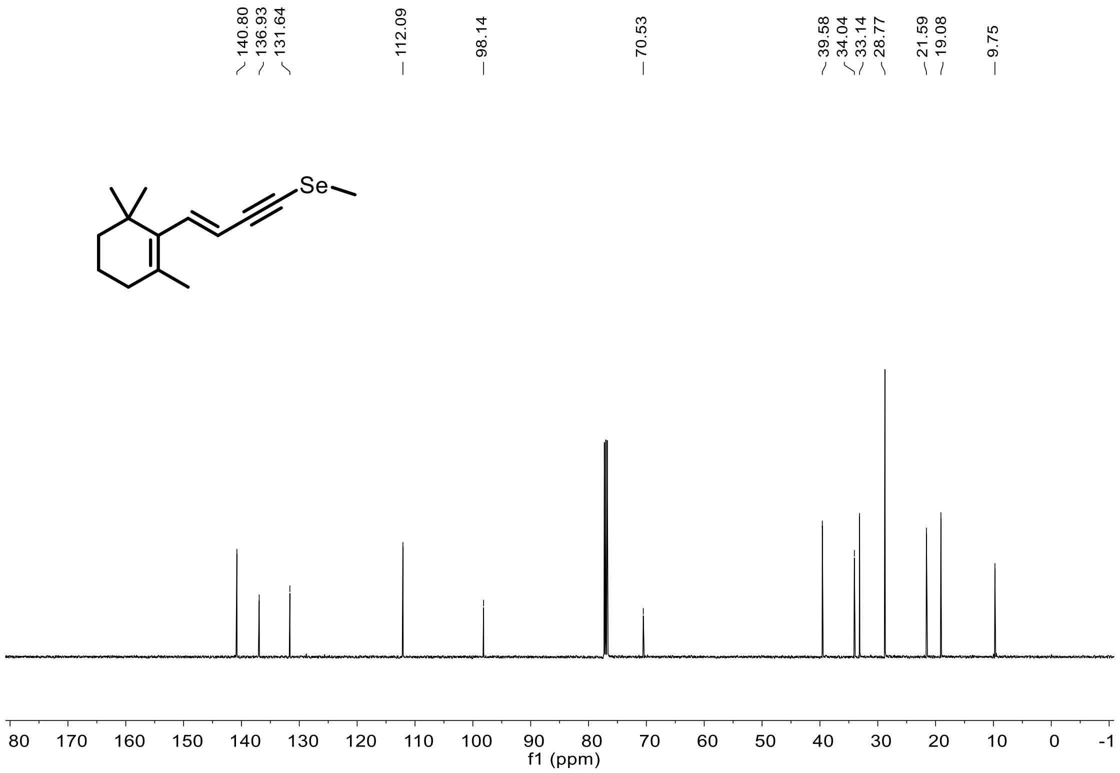

500 MHz, 298 K, $CDCl_3$ as solvent

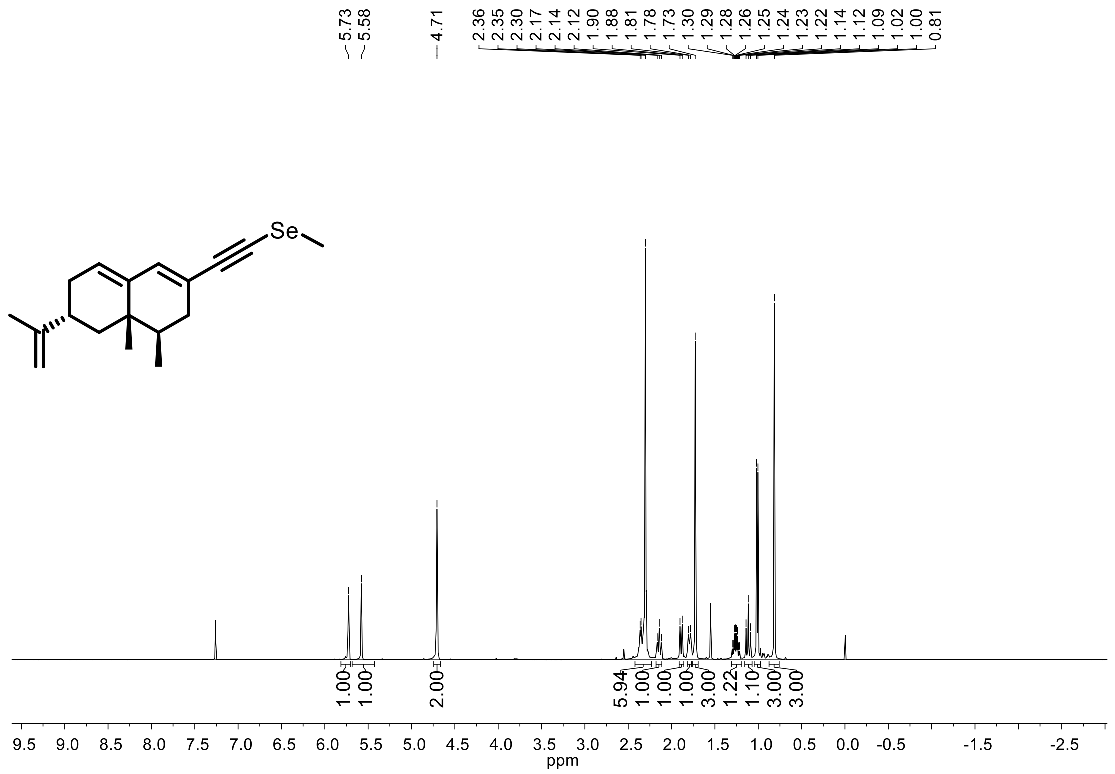

126 MHz, 298 K, $CDCl_3$ as solvent

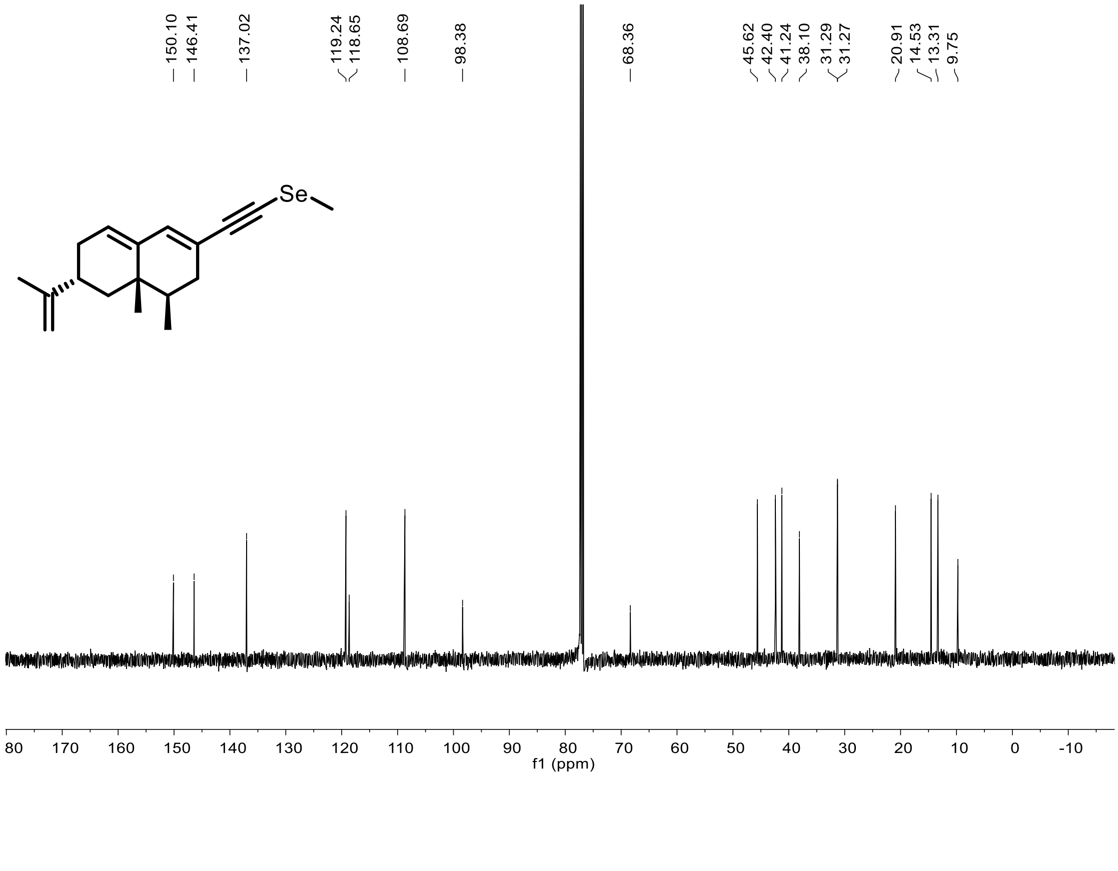

500 MHz, 298 K, CDCl3 as solvent

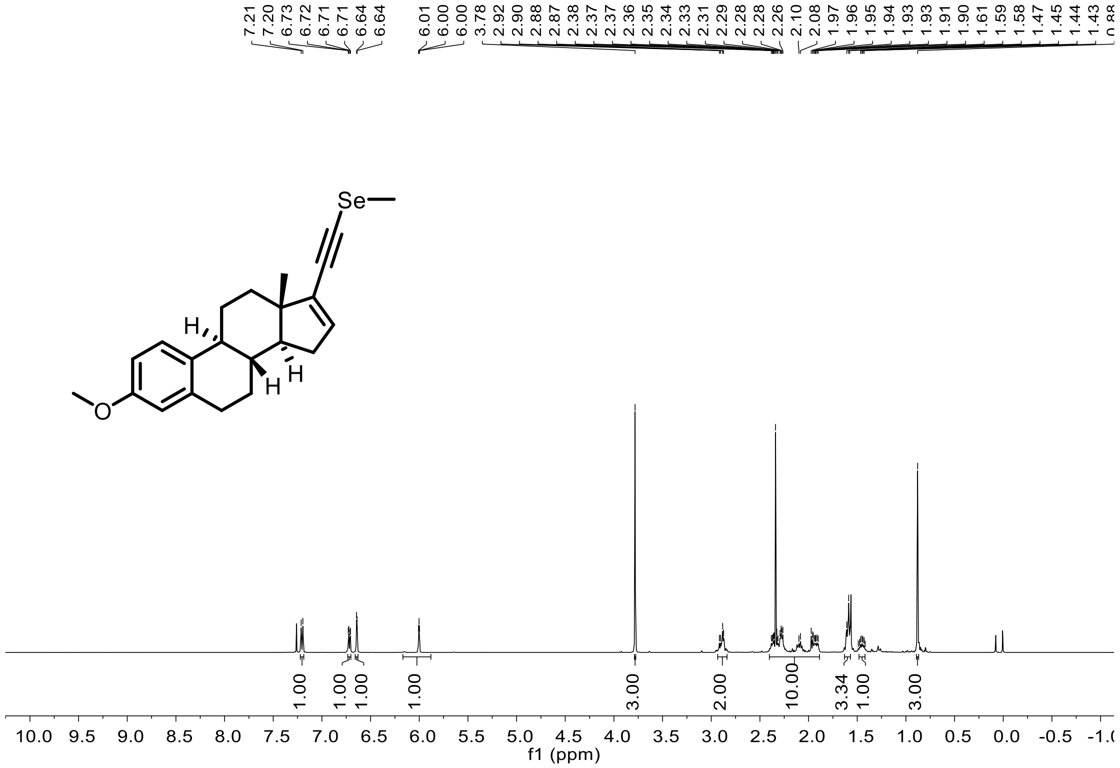

126 MHz, 298 K, CDCl3 as solvent

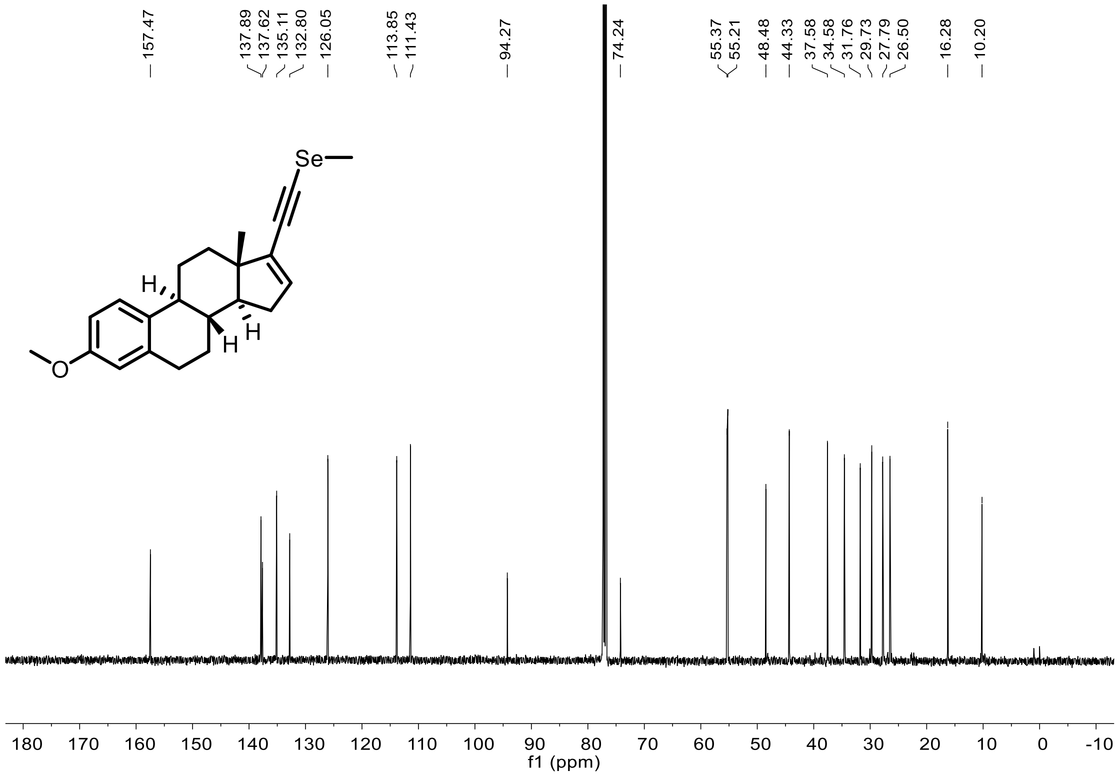

500 MHz, 298 K, $CDCl_3$ as solvent

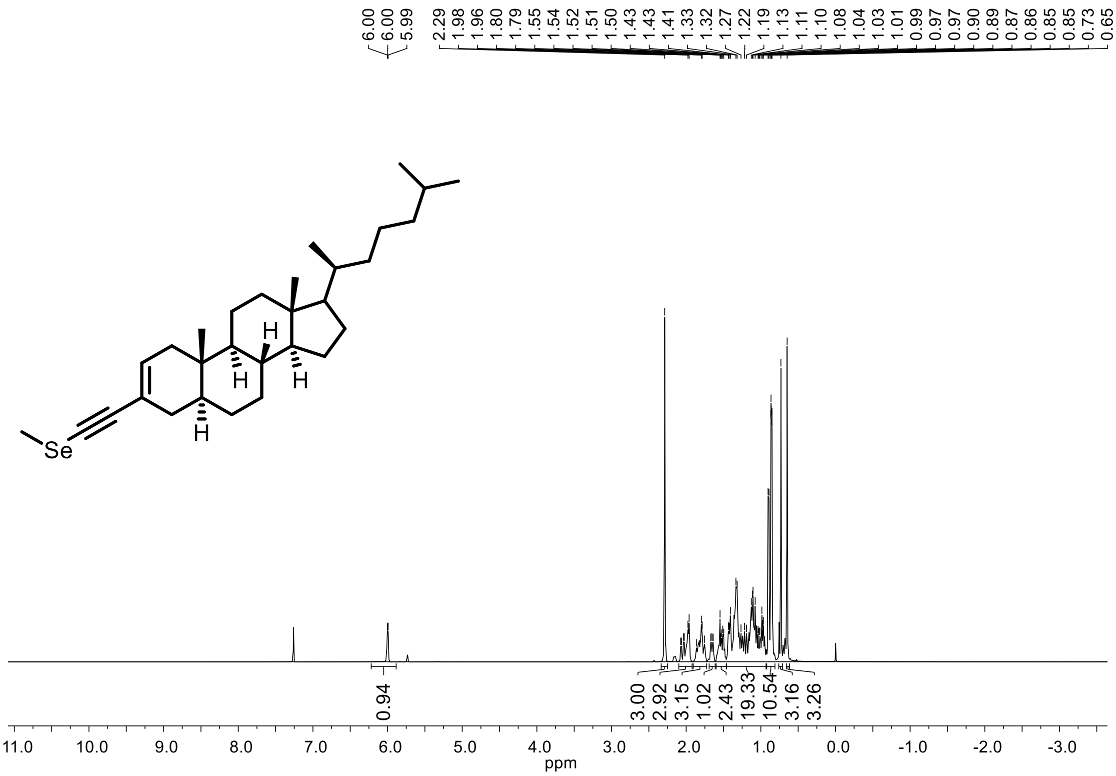

126 MHz, 298 K, $CDCl_3$ as solvent

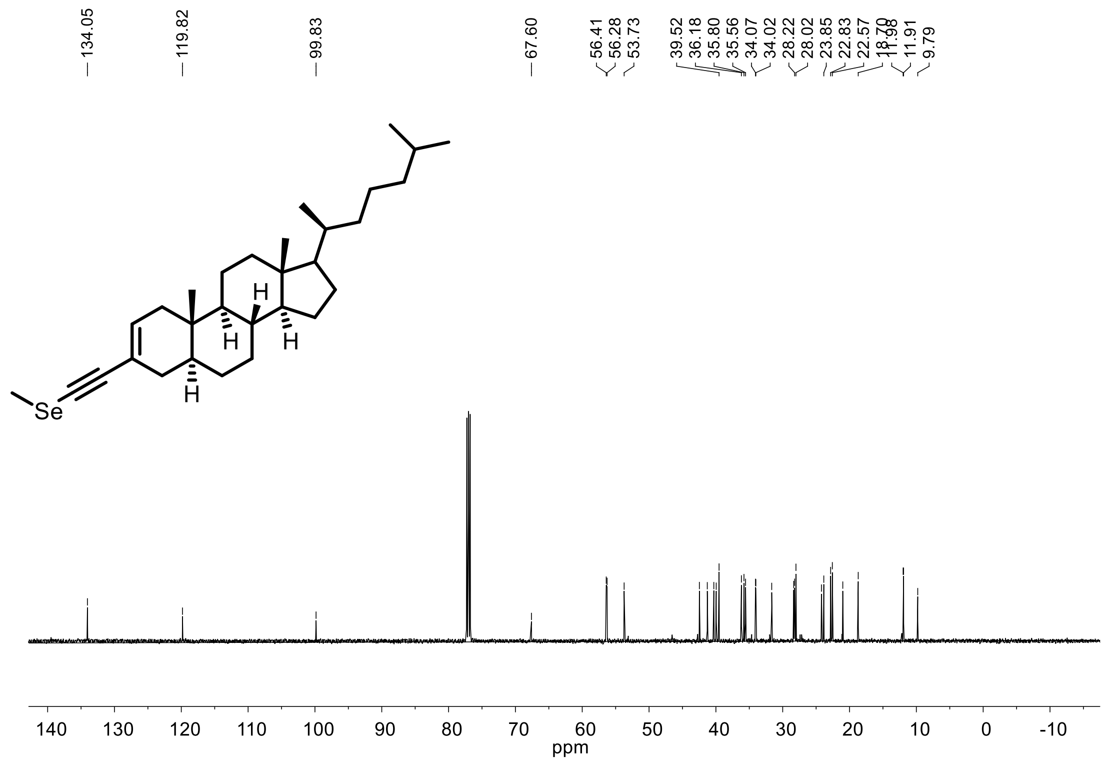

500 MHz, 298 K, $CDCl_3$ as solvent

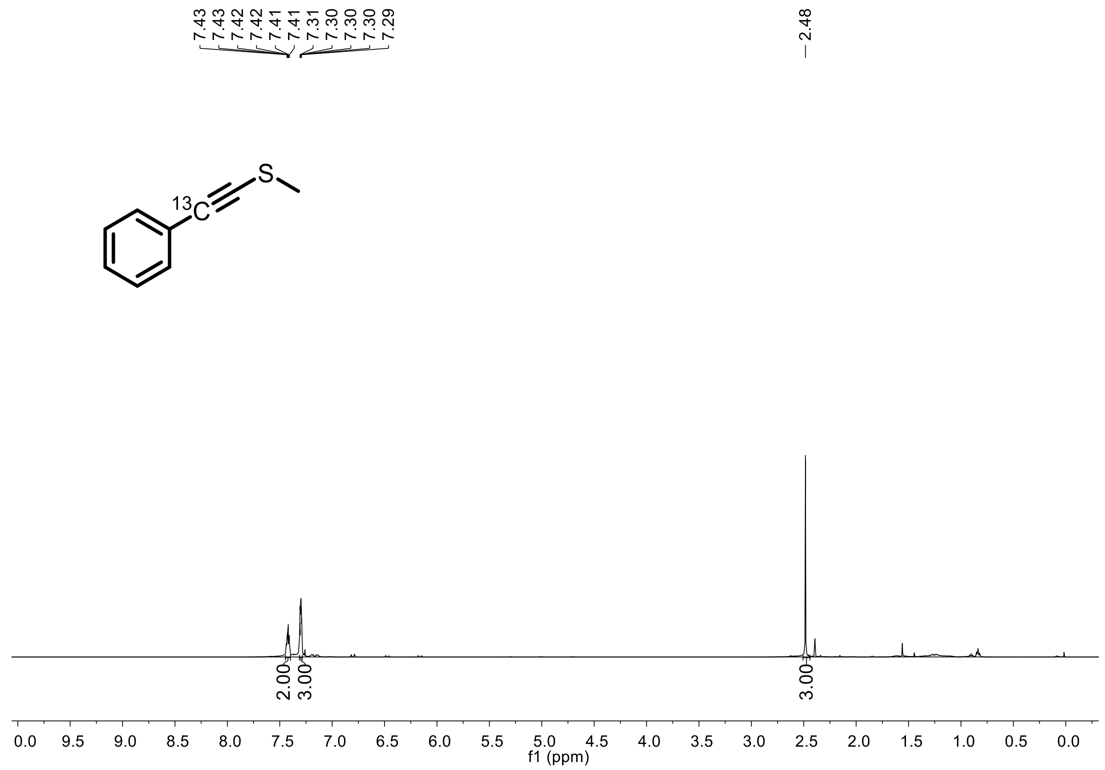

126 MHz, 298 K, $CDCl_3$ as solvent

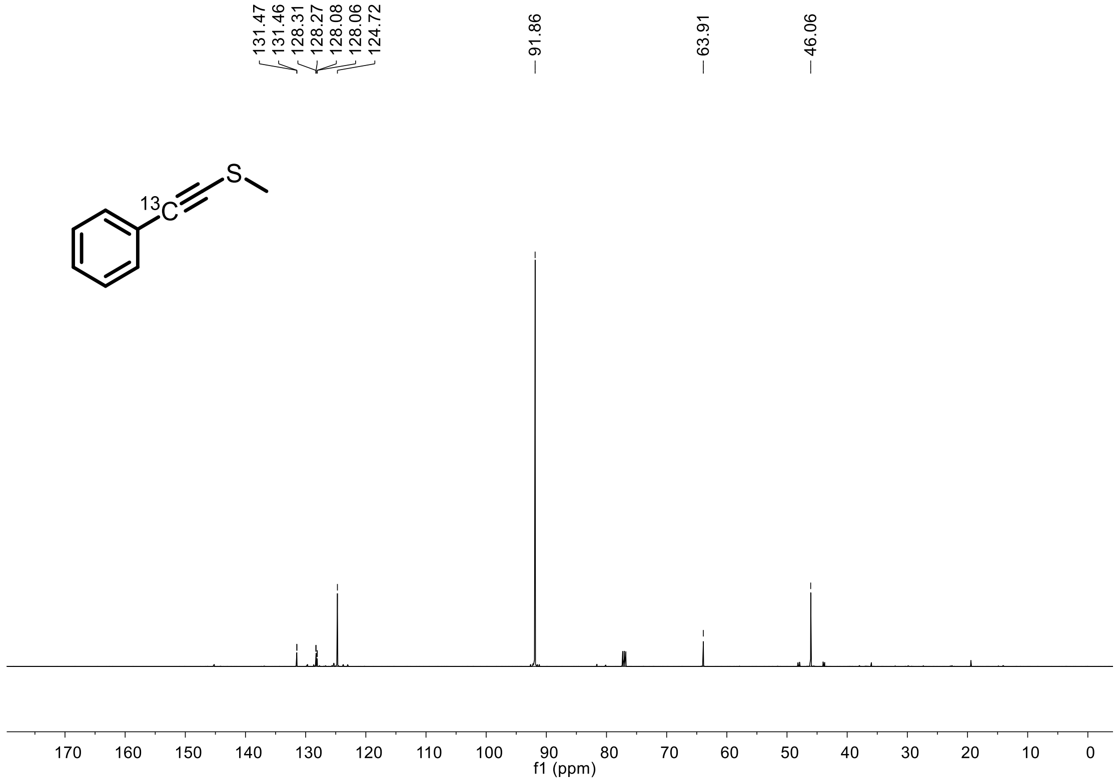

500 MHz, 298 K, $CDCl_3$ as solvent

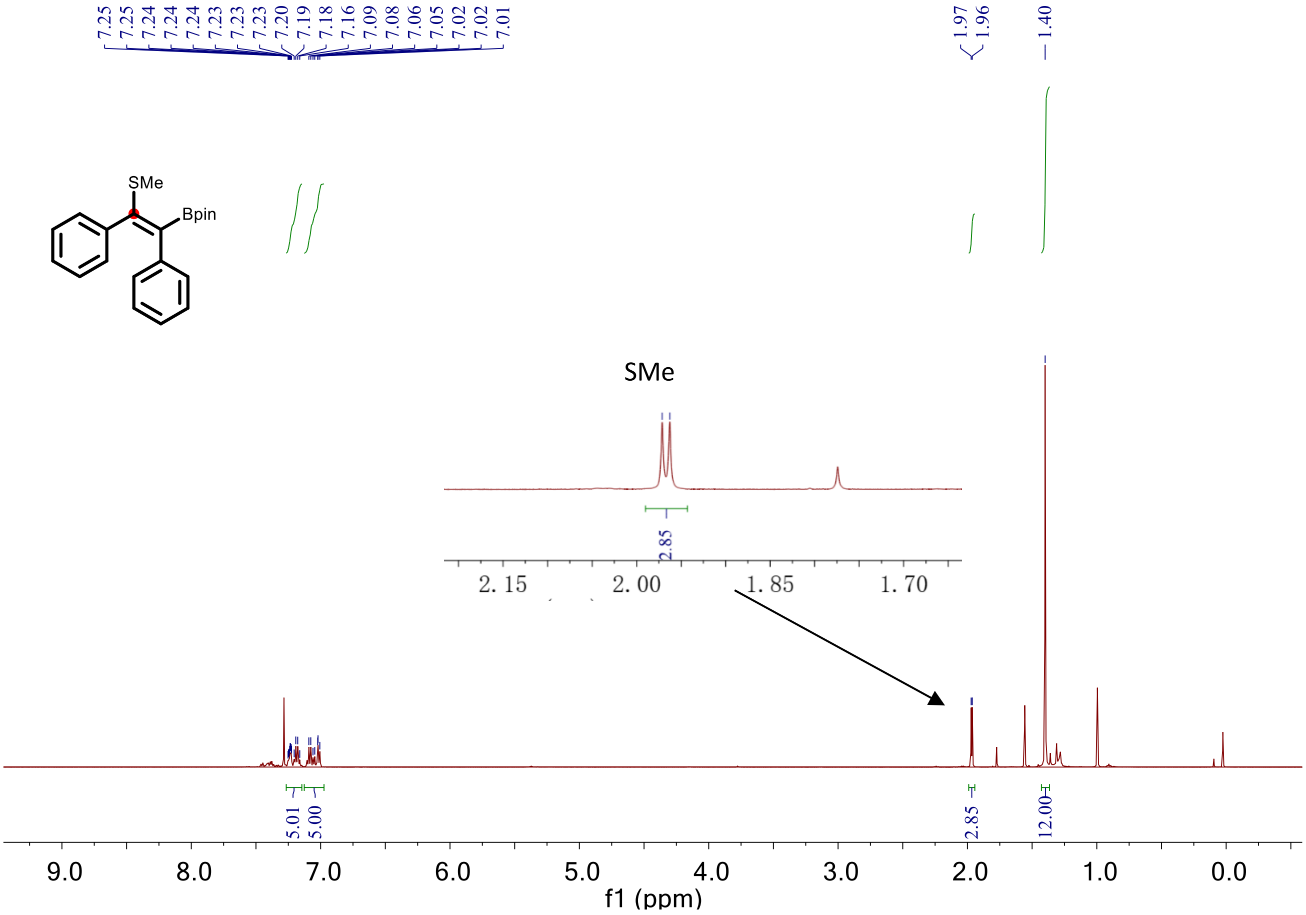

500 MHz, 298 K, CDCl3 as solvent

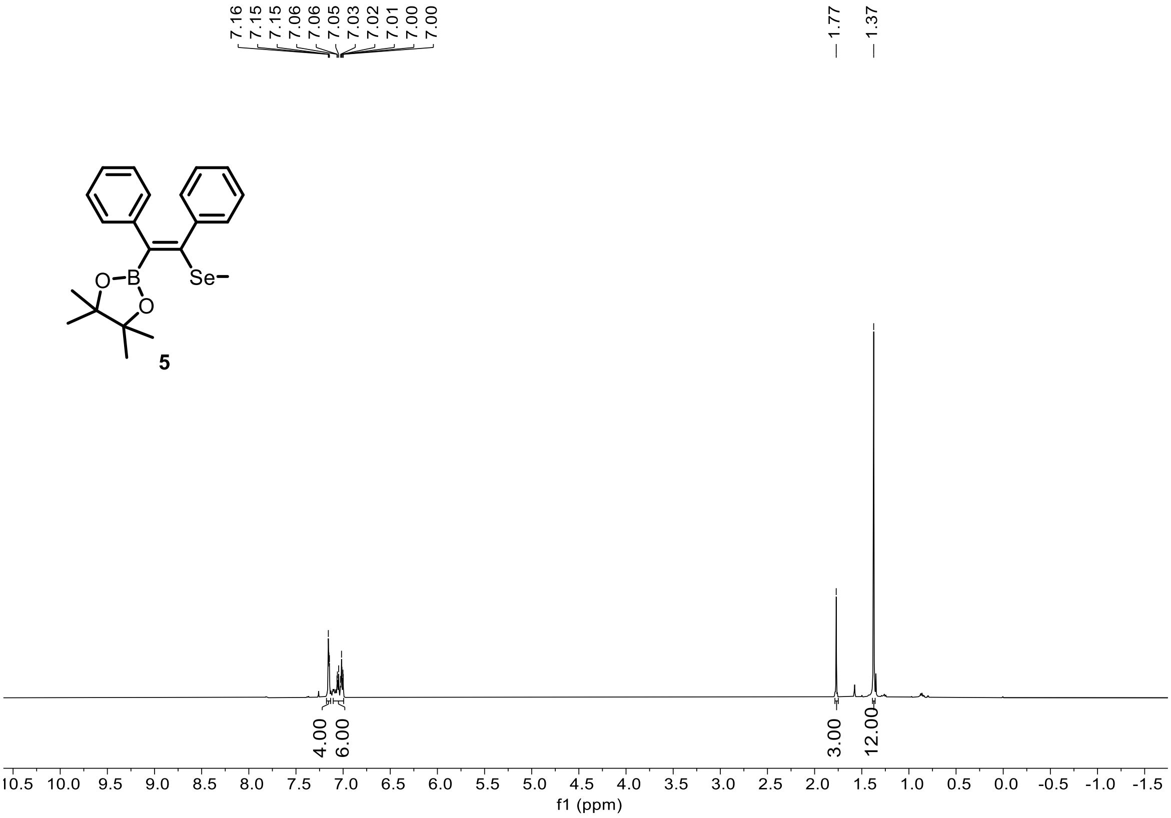

126 MHz, 298 K, CDCl3 as solvent

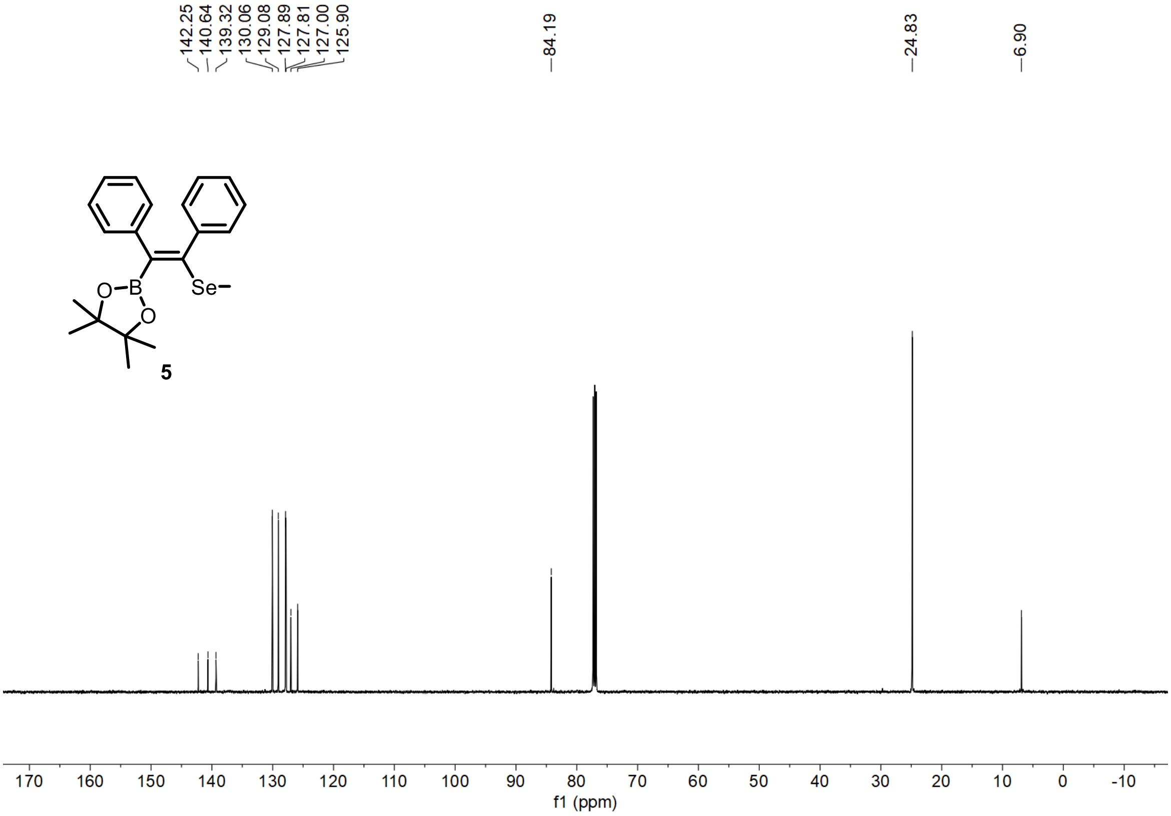

500 MHz, 298 K, $CDCl_3$ as solvent

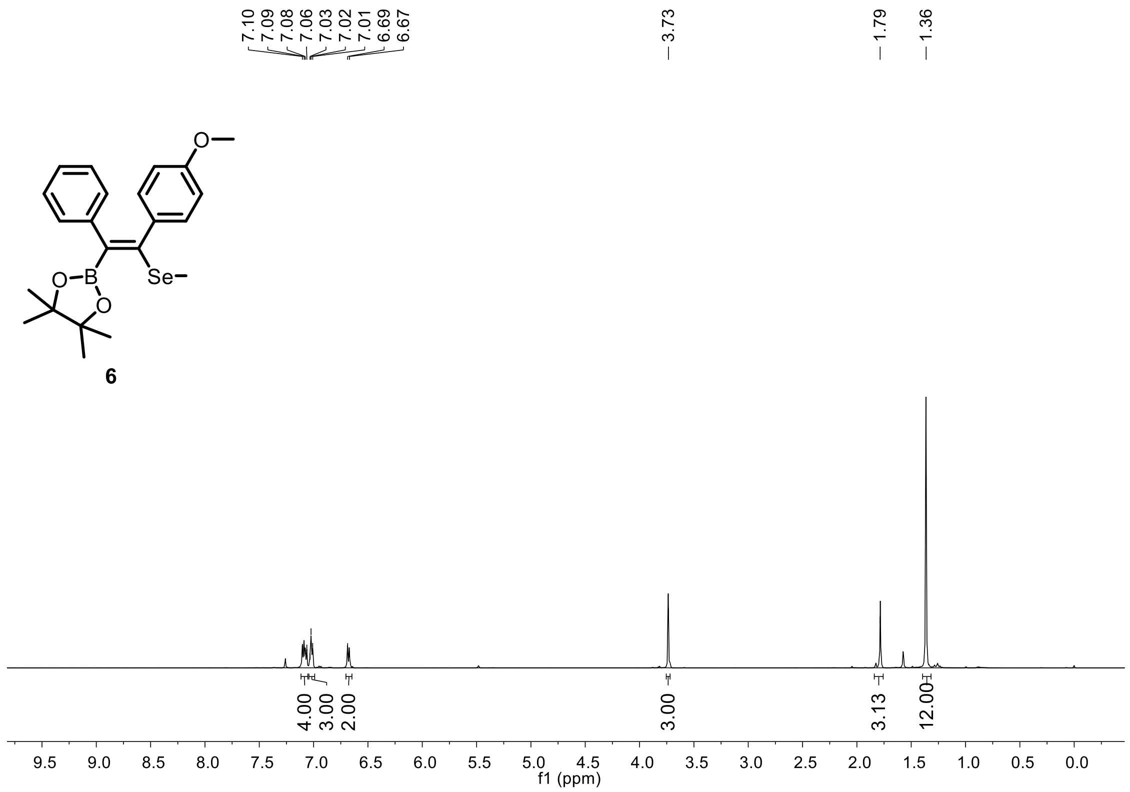

126 MHz, 298 K, $CDCl_3$ as solvent

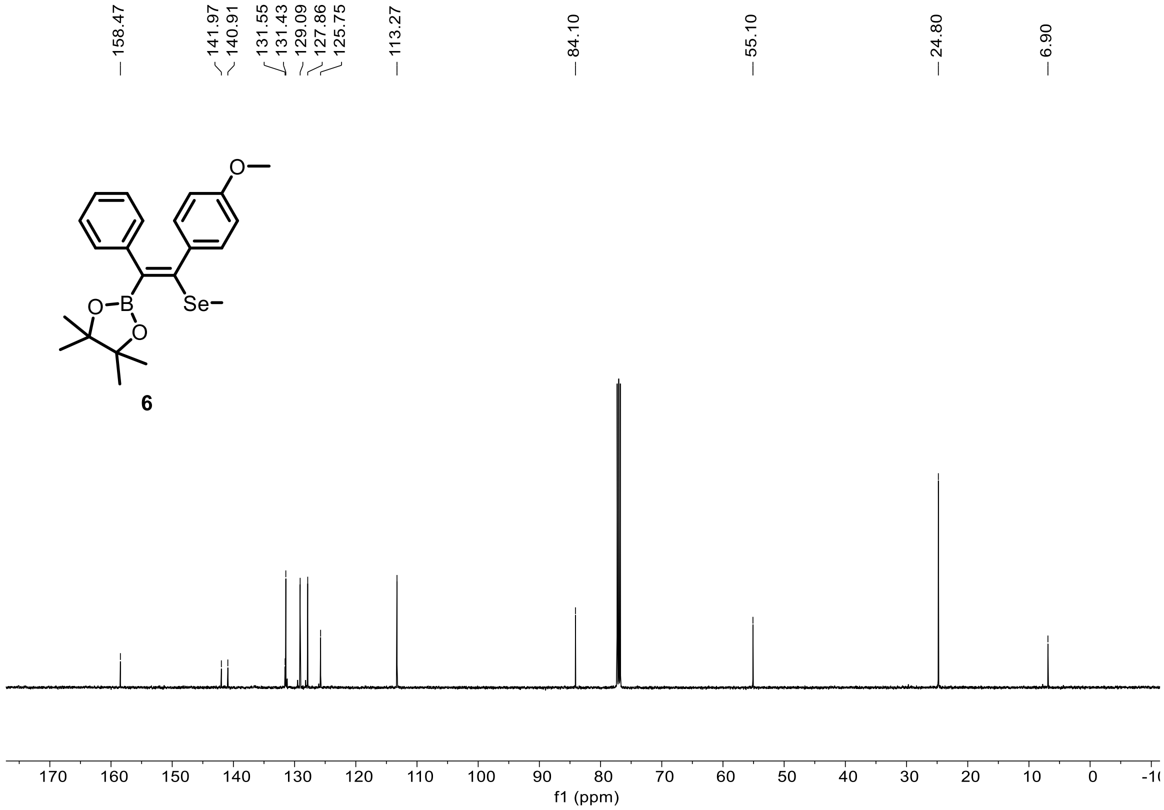

500 MHz, 298 K, $CDCl_3$ as solvent

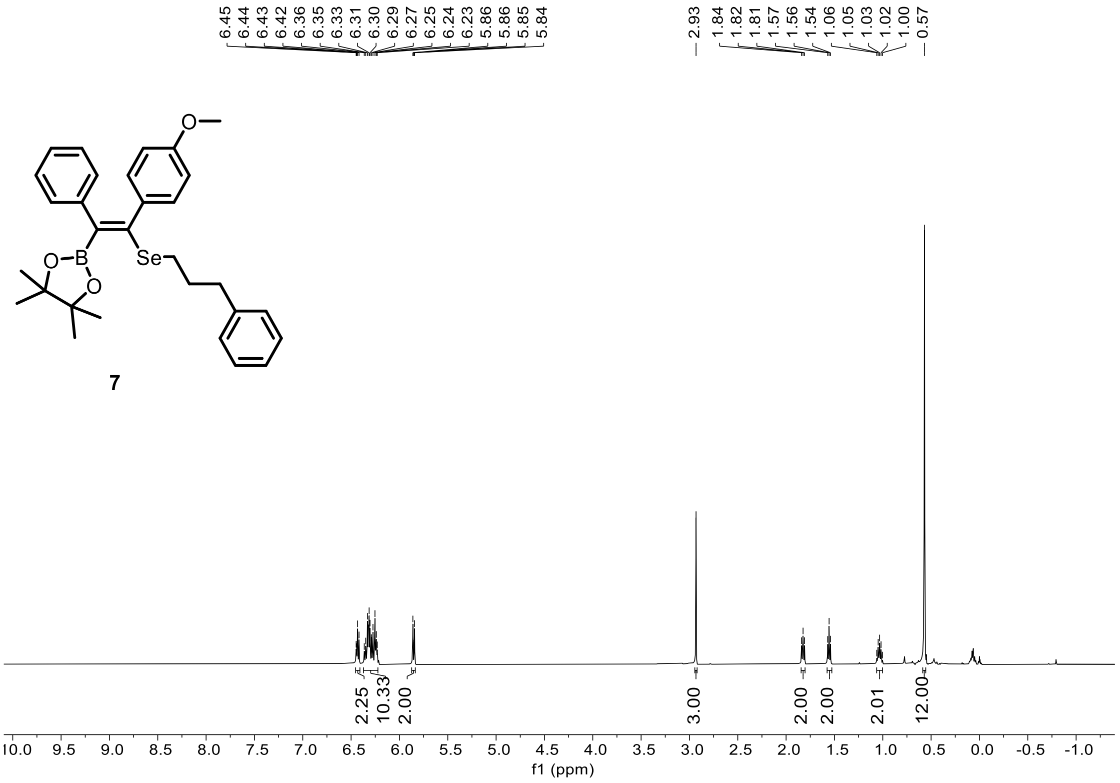

126 MHz, 298 K, $CDCl_3$ as solvent

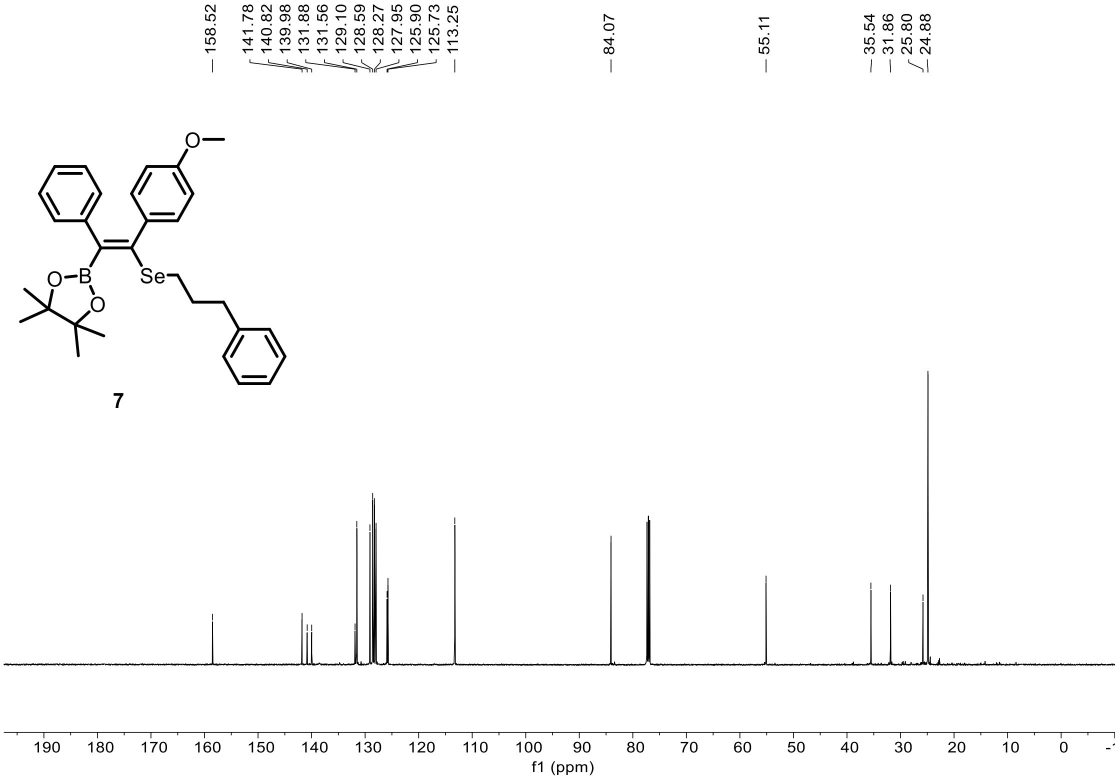

500 MHz, 298 K, $CDCl_3$ as solvent

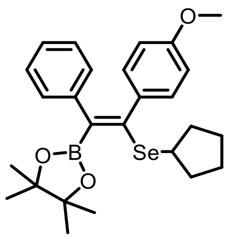

8

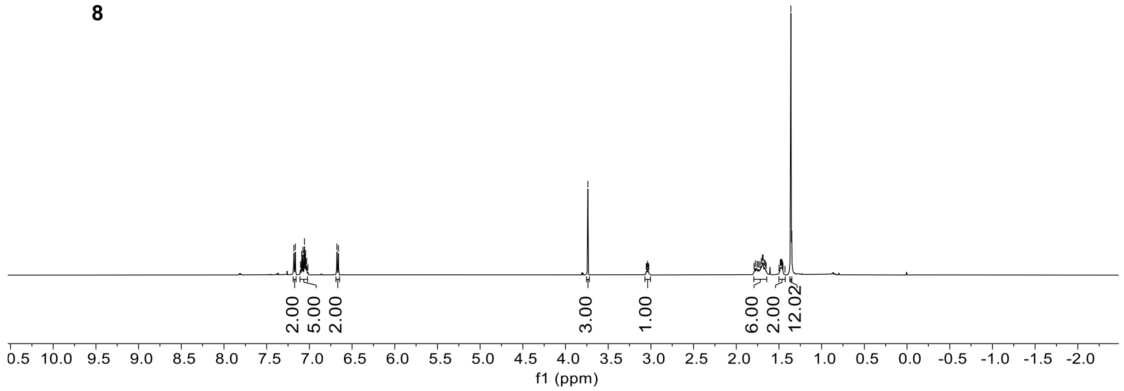

126 MHz, 298 K, $CDCl_3$ as solvent

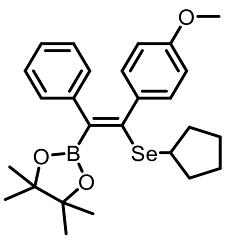

8

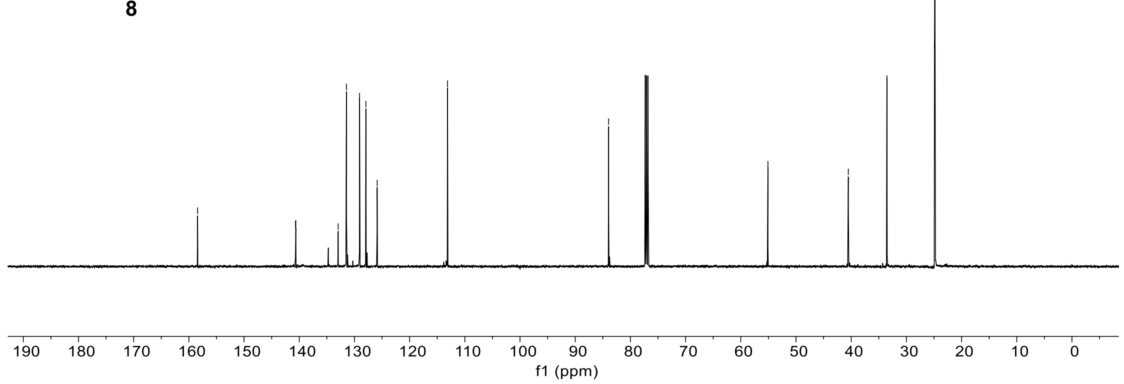

500 MHz, 298 K, $CDCl_3$ as solvent

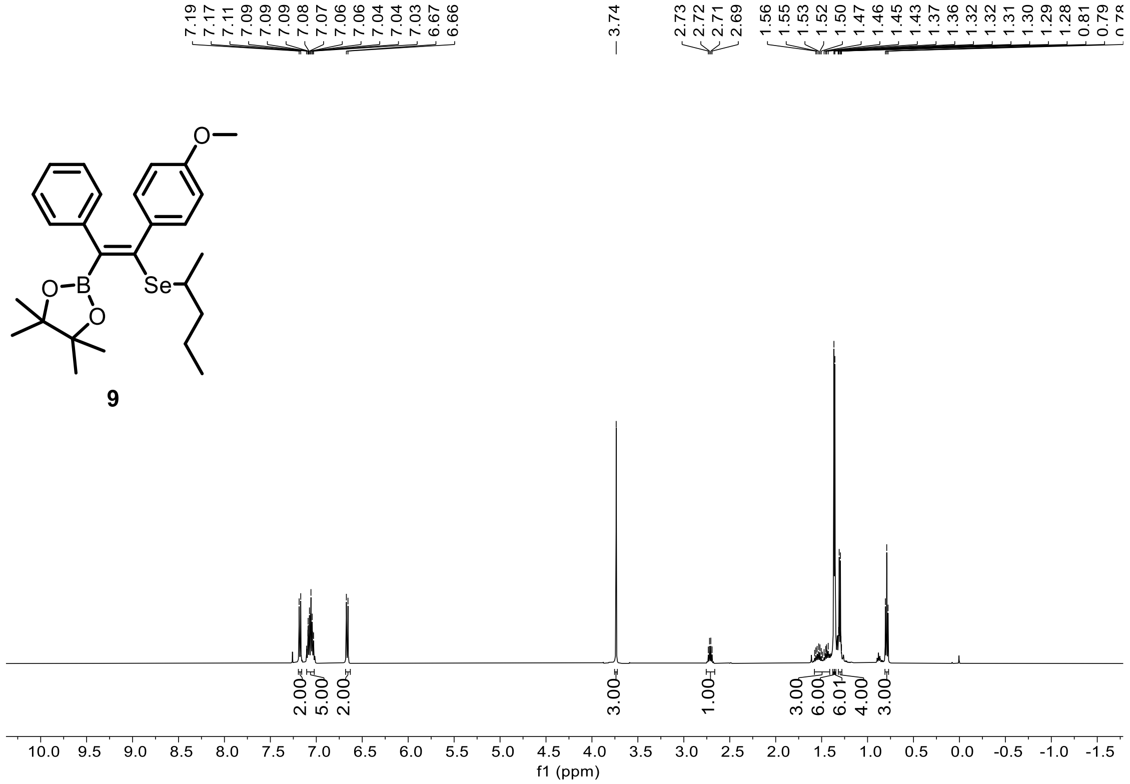

126 MHz, 298 K, $CDCl_3$ as solvent

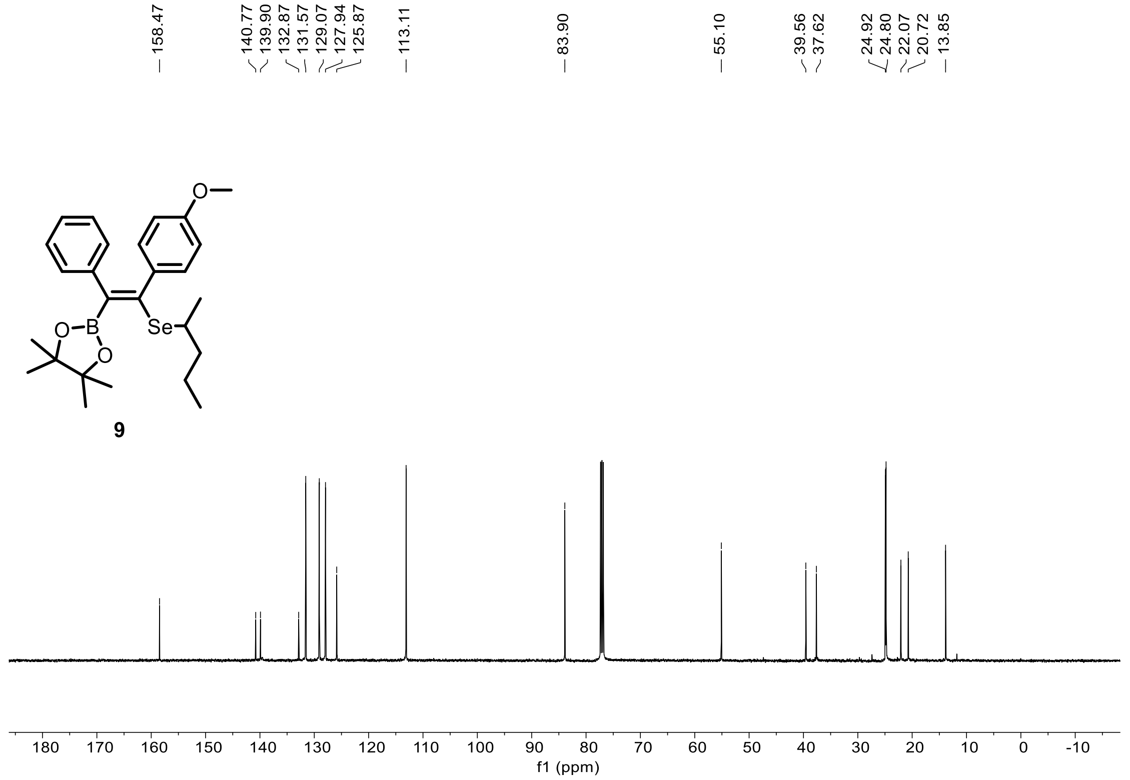

500 MHz, 298 K, $CDCl_3$ as solvent

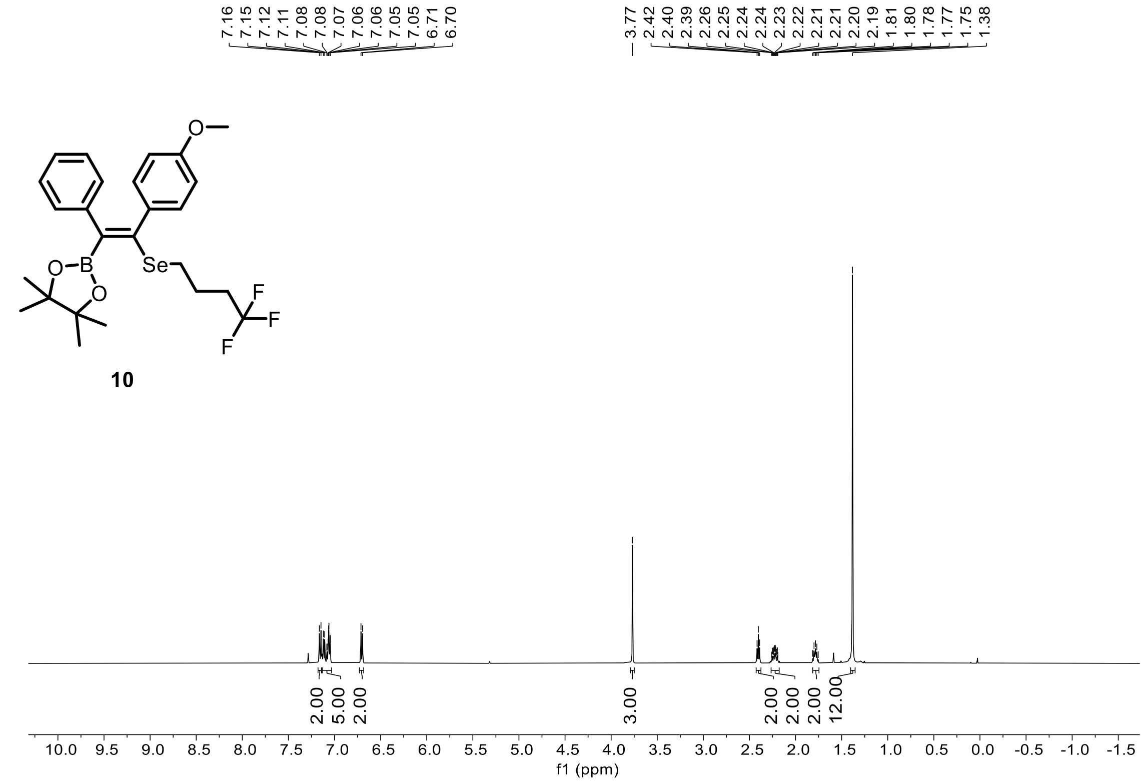

126 MHz, 298 K, $CDCl_3$ as solvent

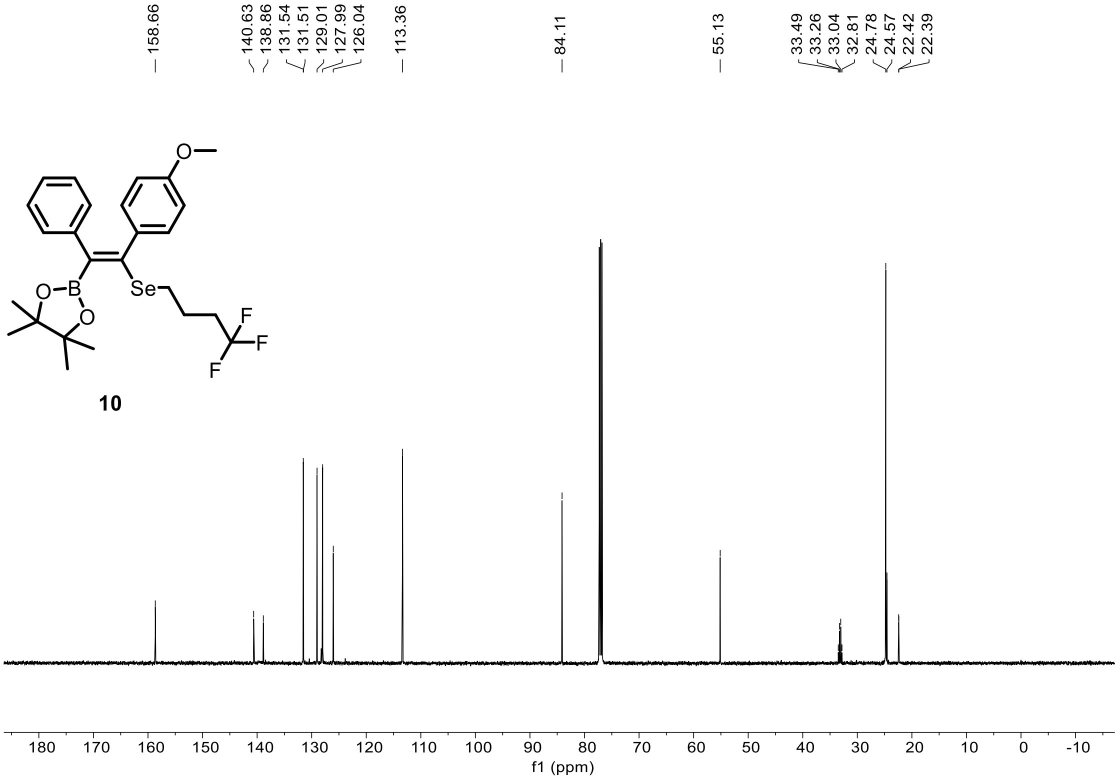

500 MHz, 298 K, $CDCl_3$ as solvent

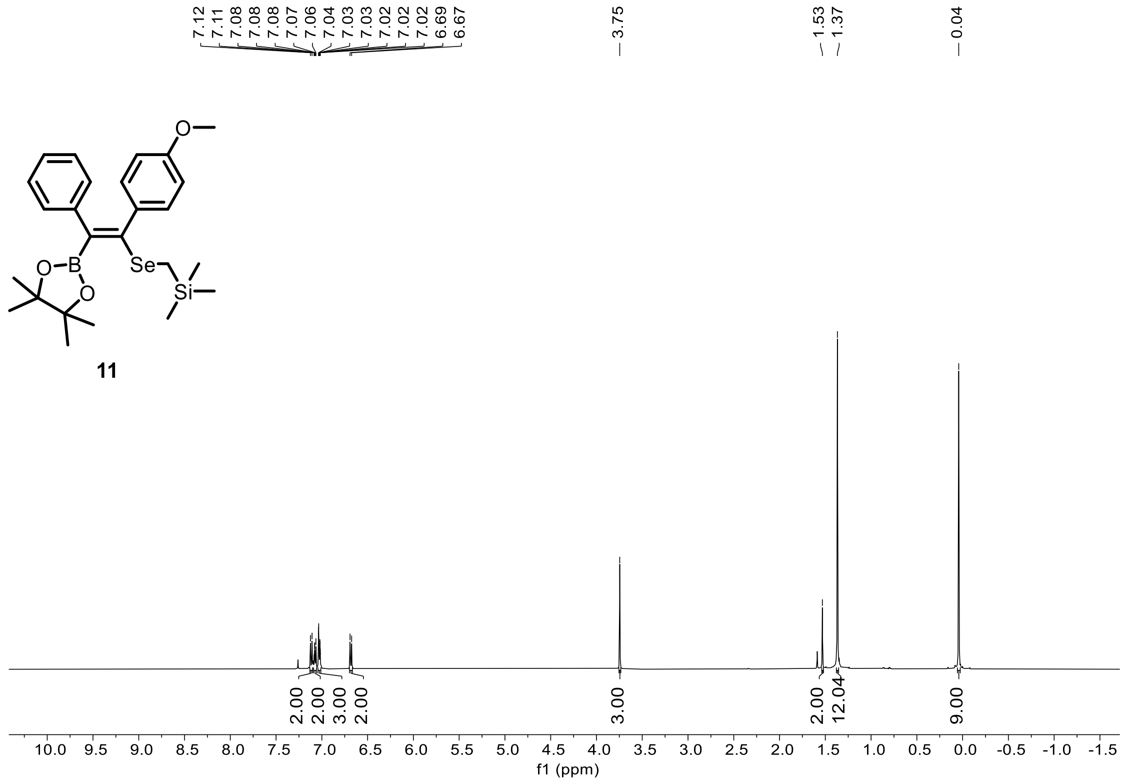

126 MHz, 298 K, $CDCl_3$ as solvent

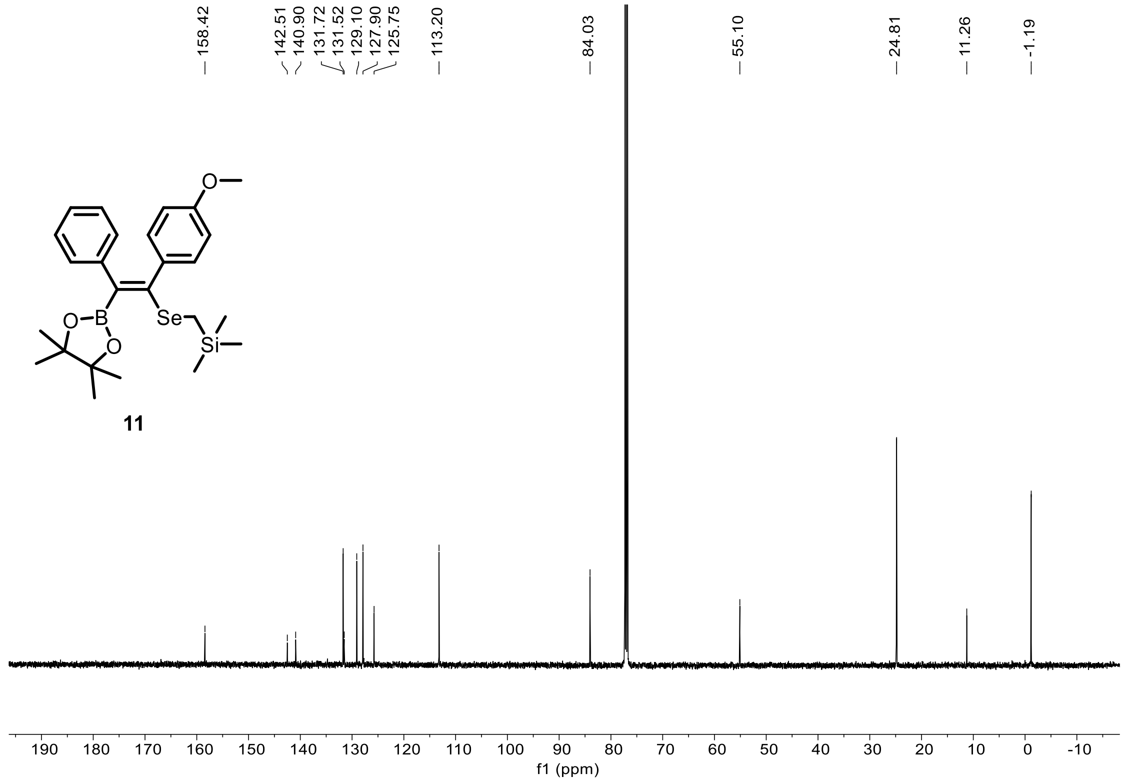

500 MHz, 298 K, $CDCl_3$ as solvent

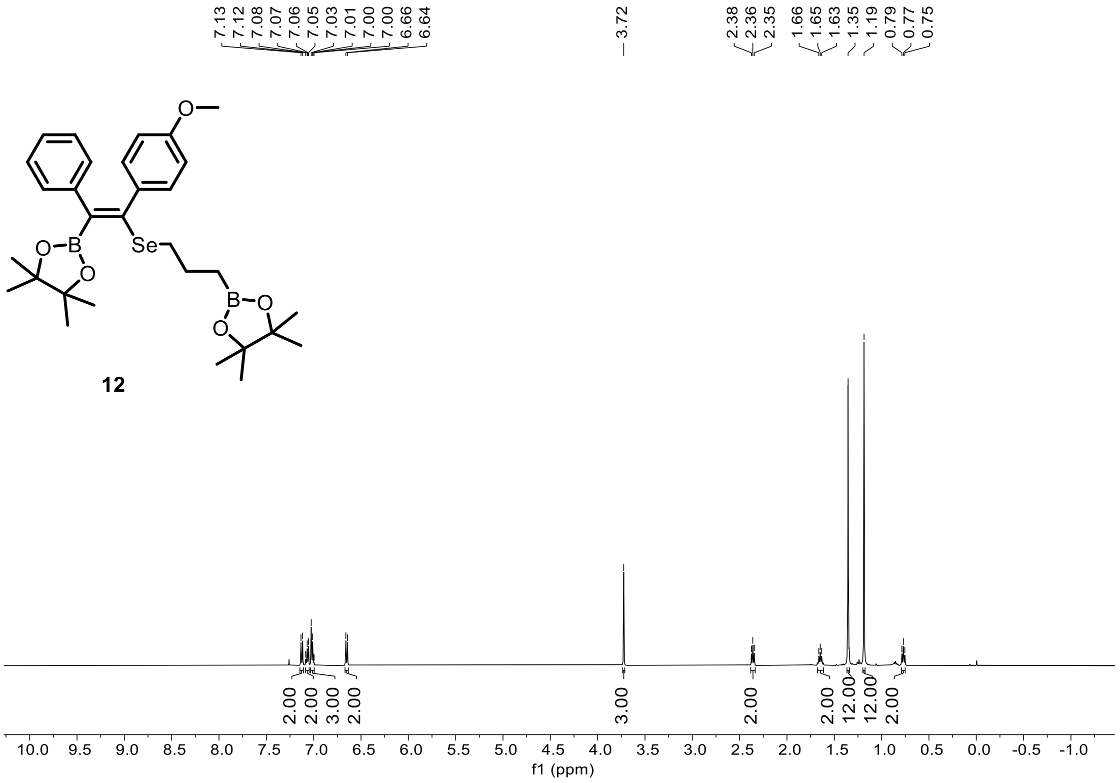

126 MHz, 298 K, $CDCl_3$ as solvent

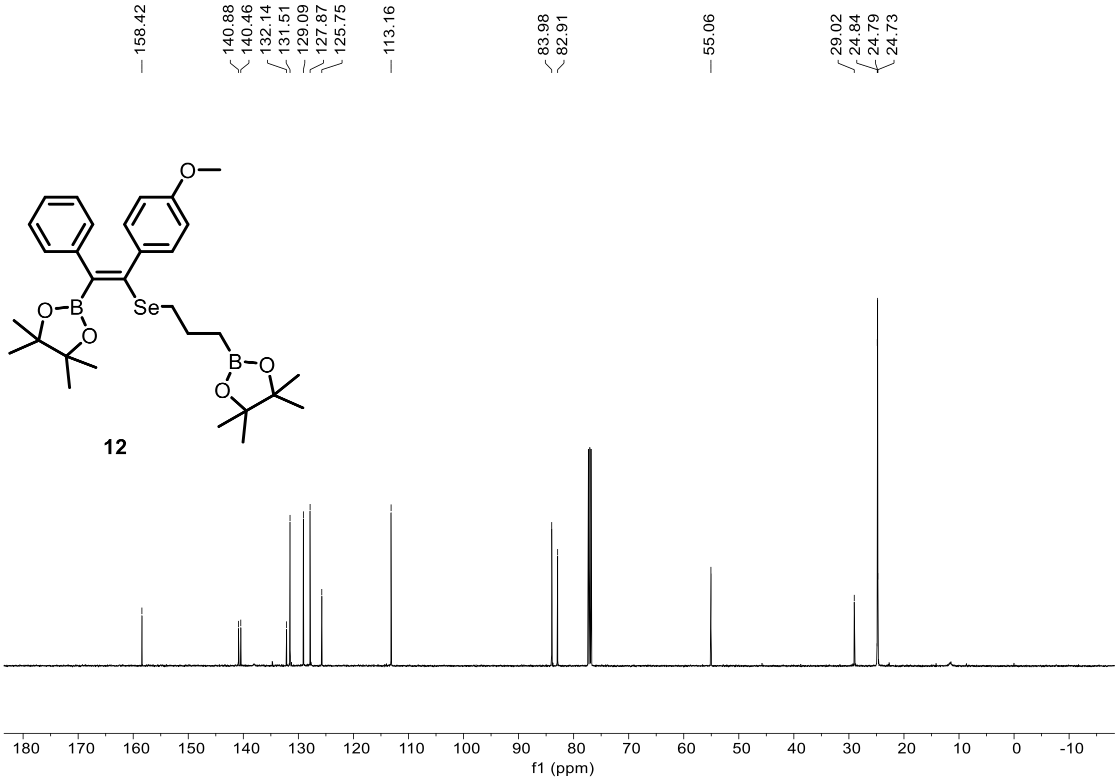

500 MHz, 298 K, CDCl3 as solvent

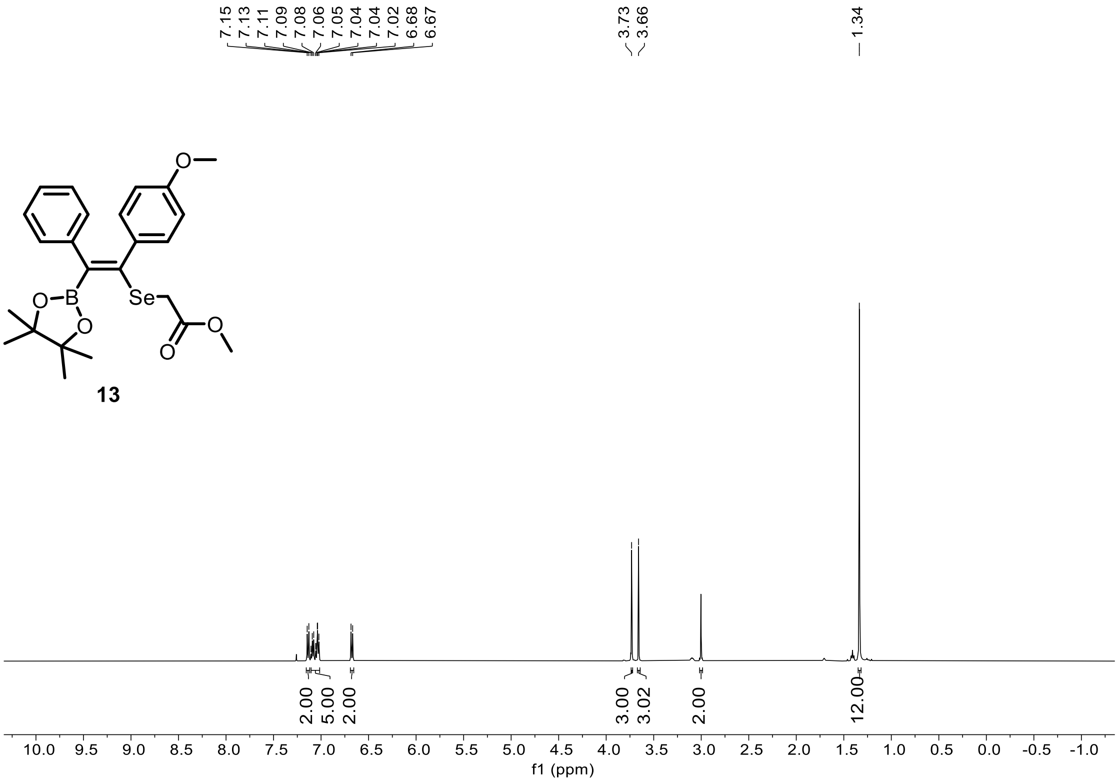

126 MHz, 298 K, CDCl3 as solvent

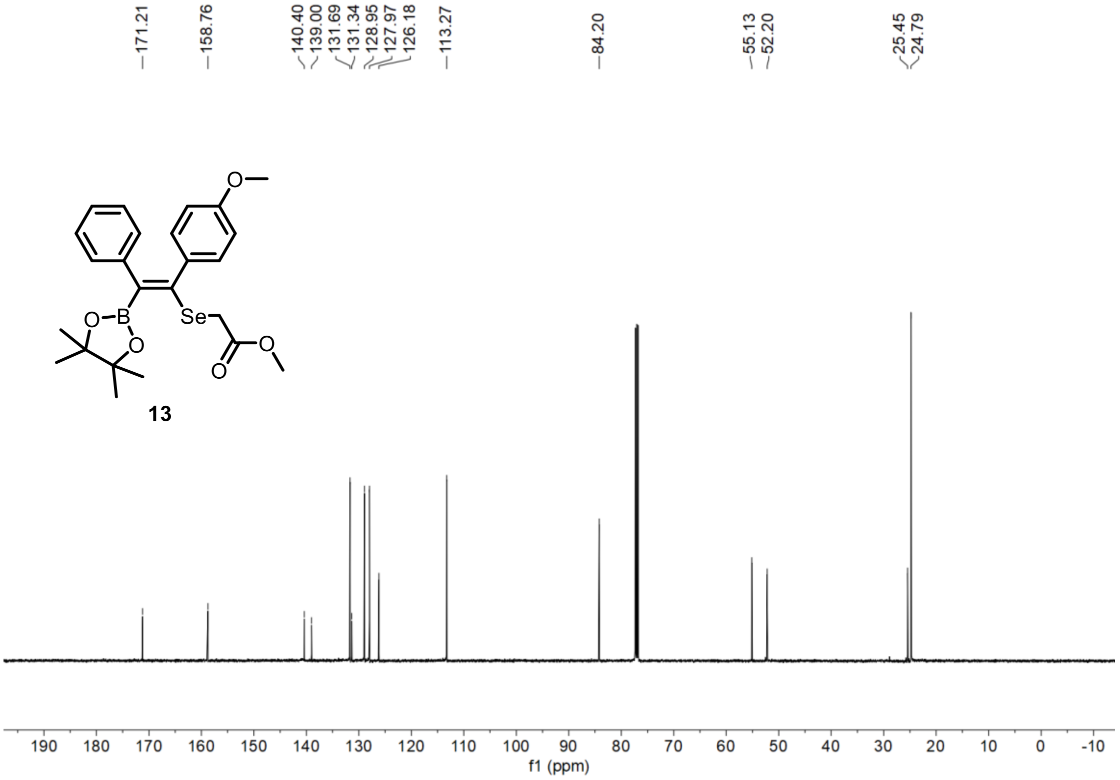

500 MHz, 298 K, $CDCl_3$ as solvent

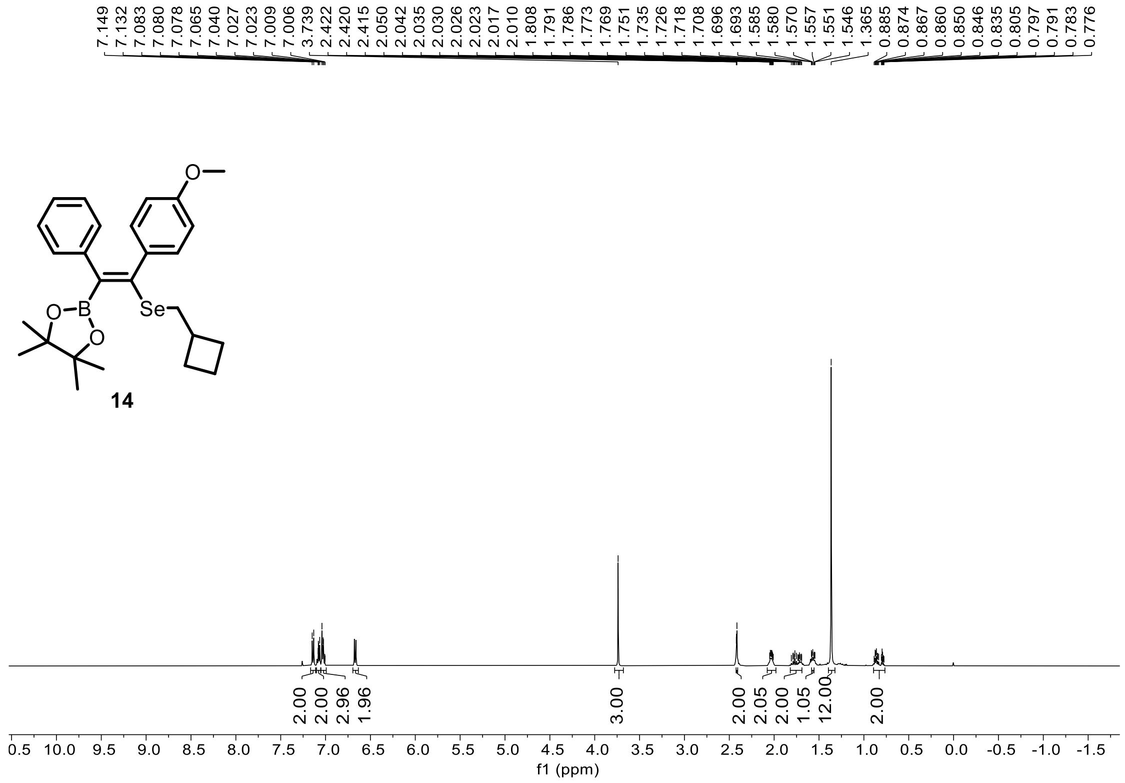

126 MHz, 298 K, $CDCl_3$ as solvent

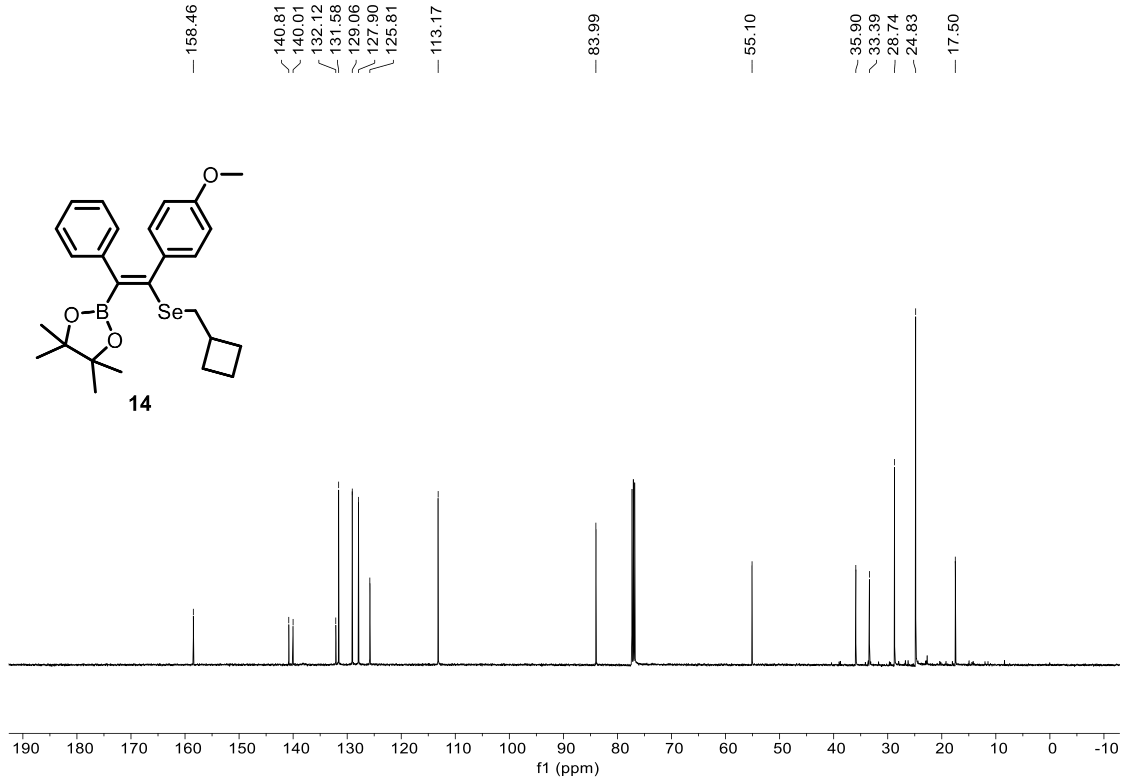

500 MHz, 298 K, $CDCl_3$ as solvent

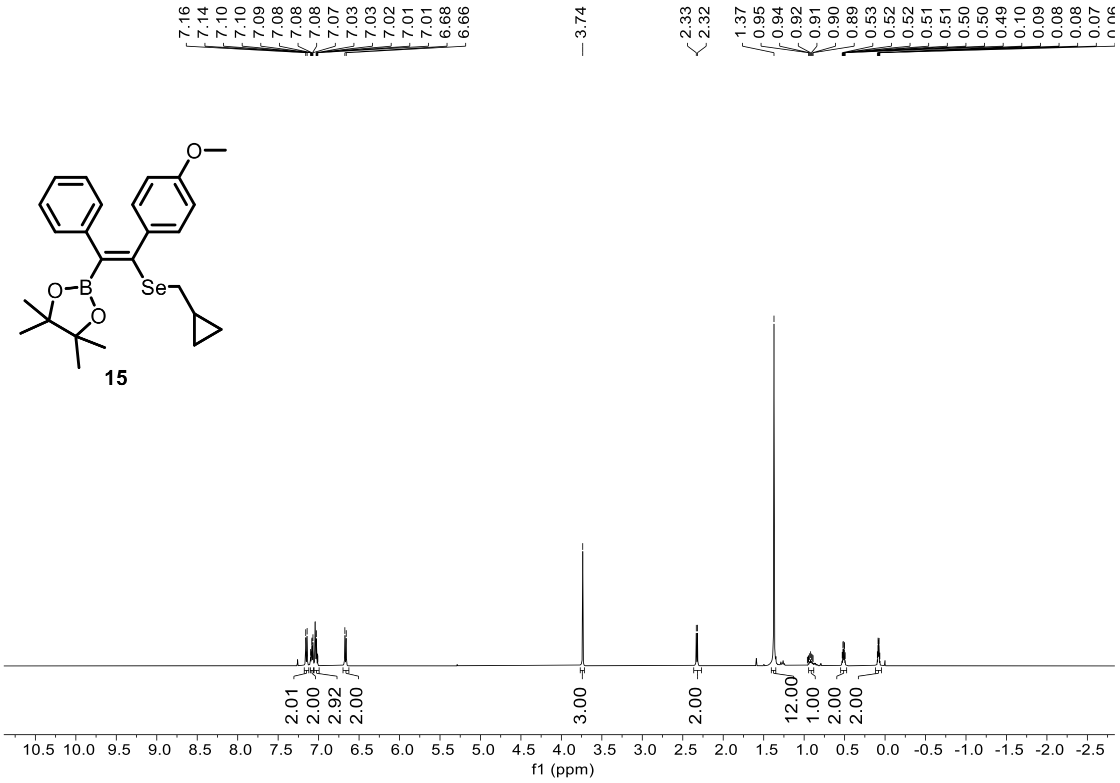

126 MHz, 298 K, $CDCl_3$ as solvent

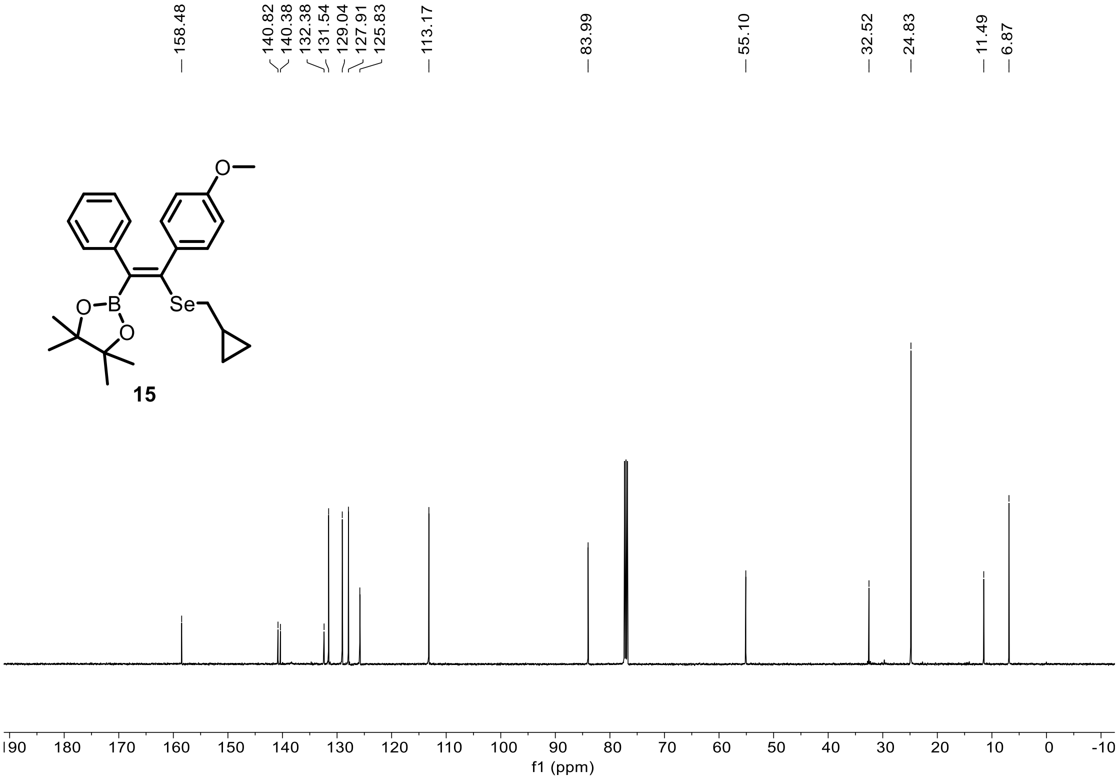

500 MHz, 298 K, CD_2Cl_2 as solvent

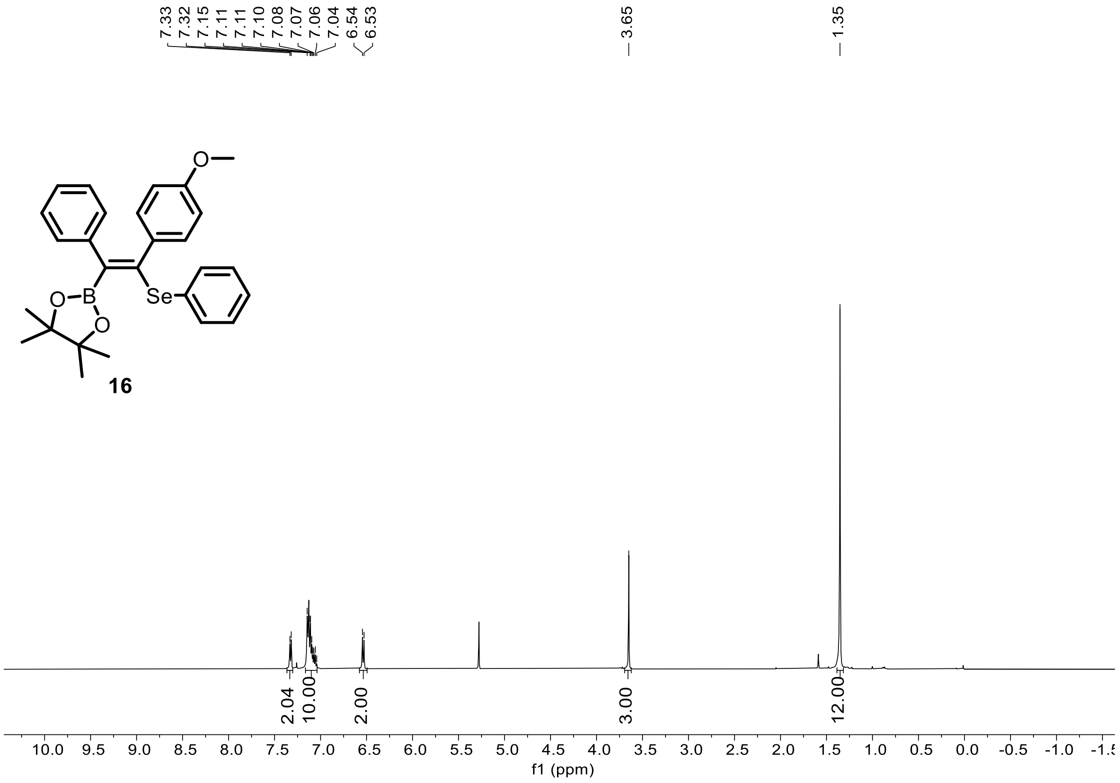

126 MHz, 298 K, $CDCl_3$ as solvent

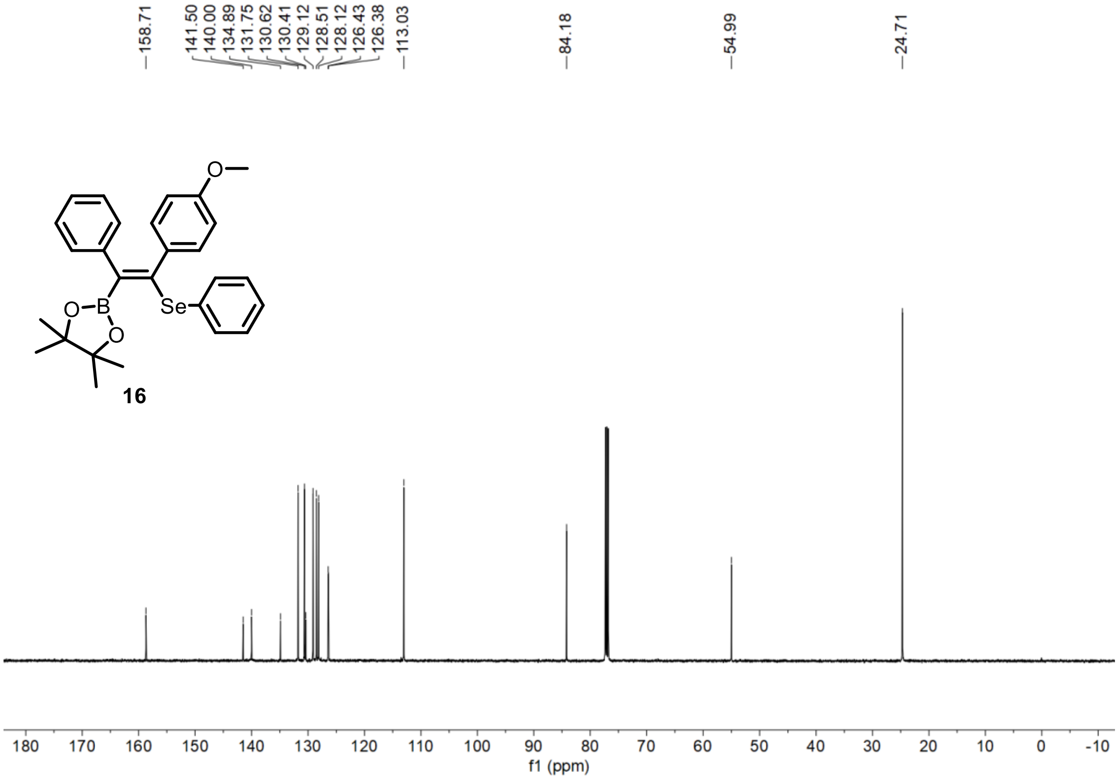

500 MHz, 298 K, $CDCl_3$ as solvent

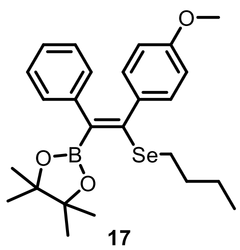

17

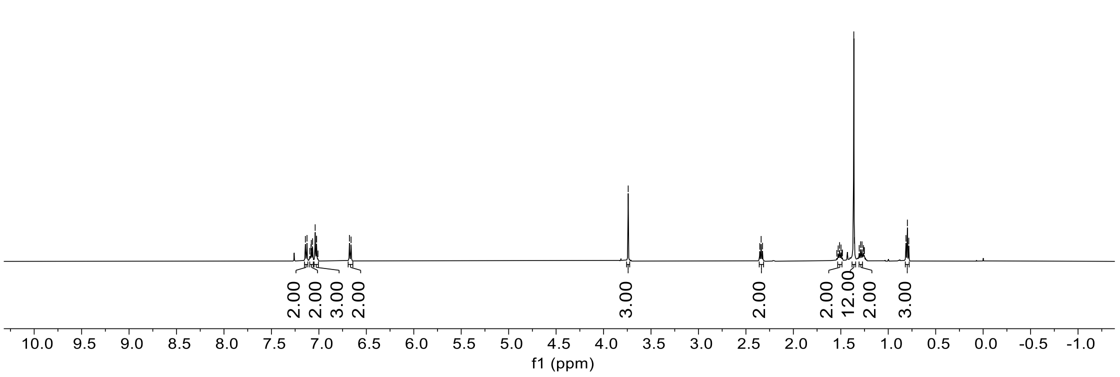

126 MHz, 298 K, $CDCl_3$ as solvent

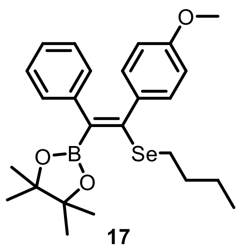

17

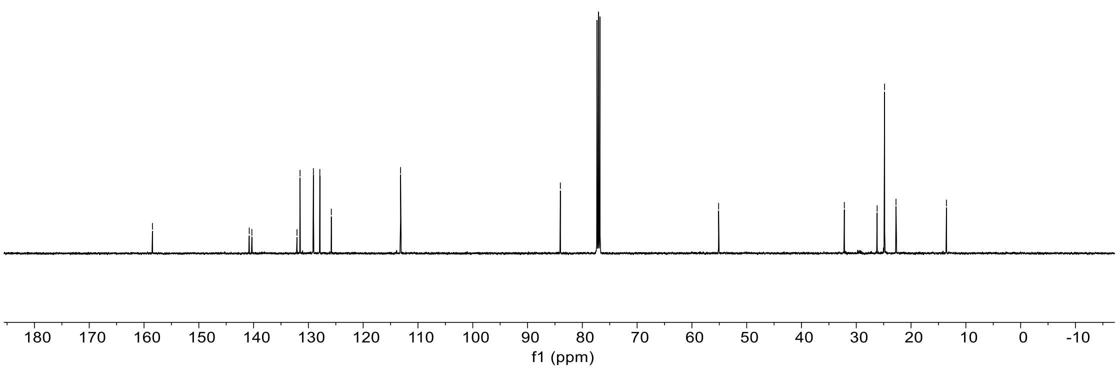

500 MHz, 298 K, $CDCl_3$ as solvent

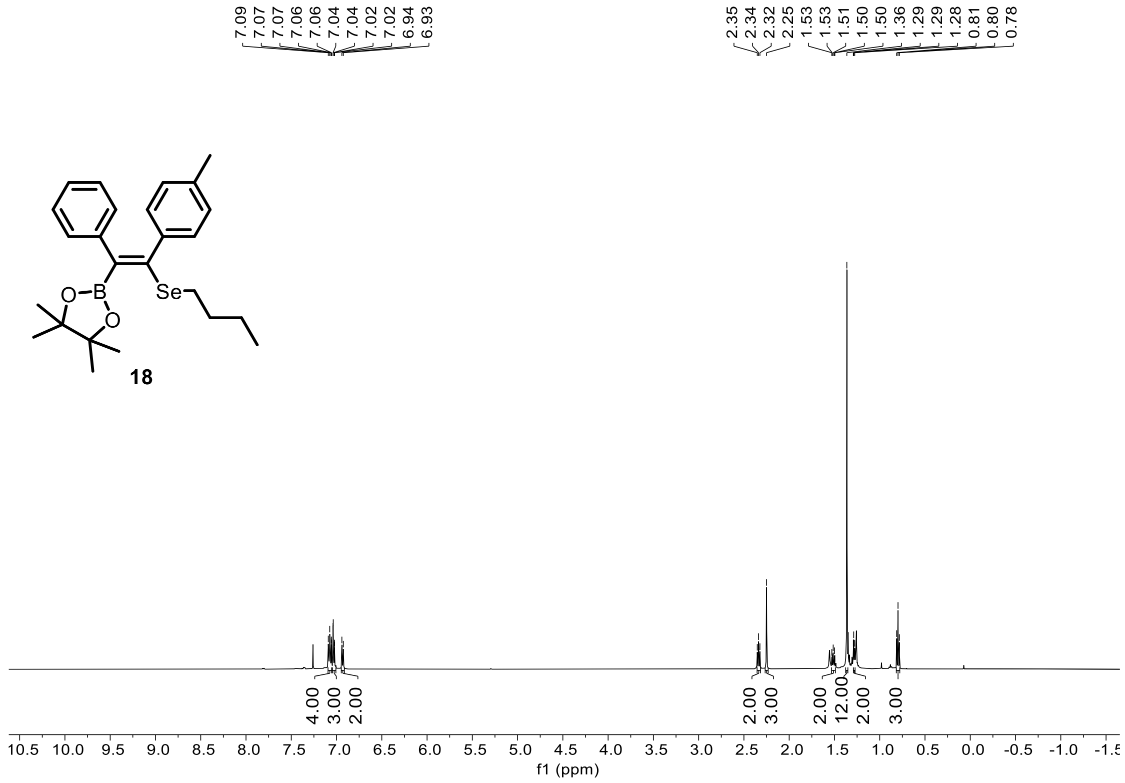

126 MHz, 298 K, $CDCl_3$ as solvent

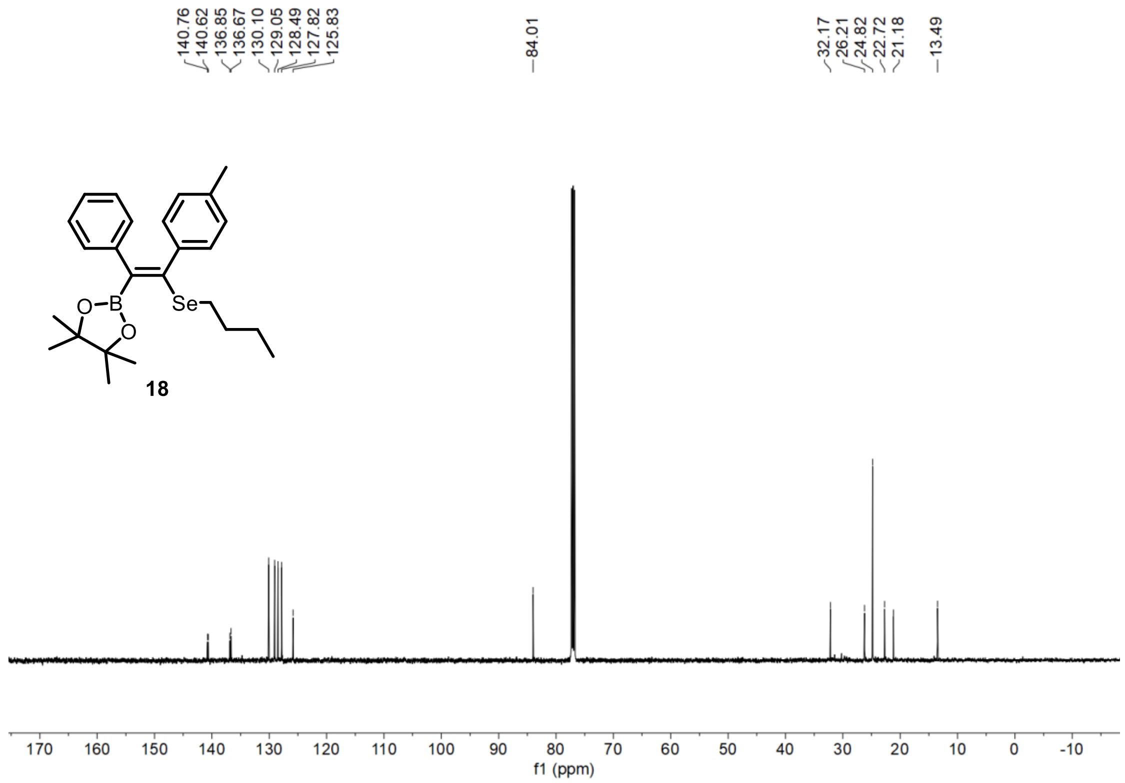

500 MHz, 298 K, $CDCl_3$ as solvent

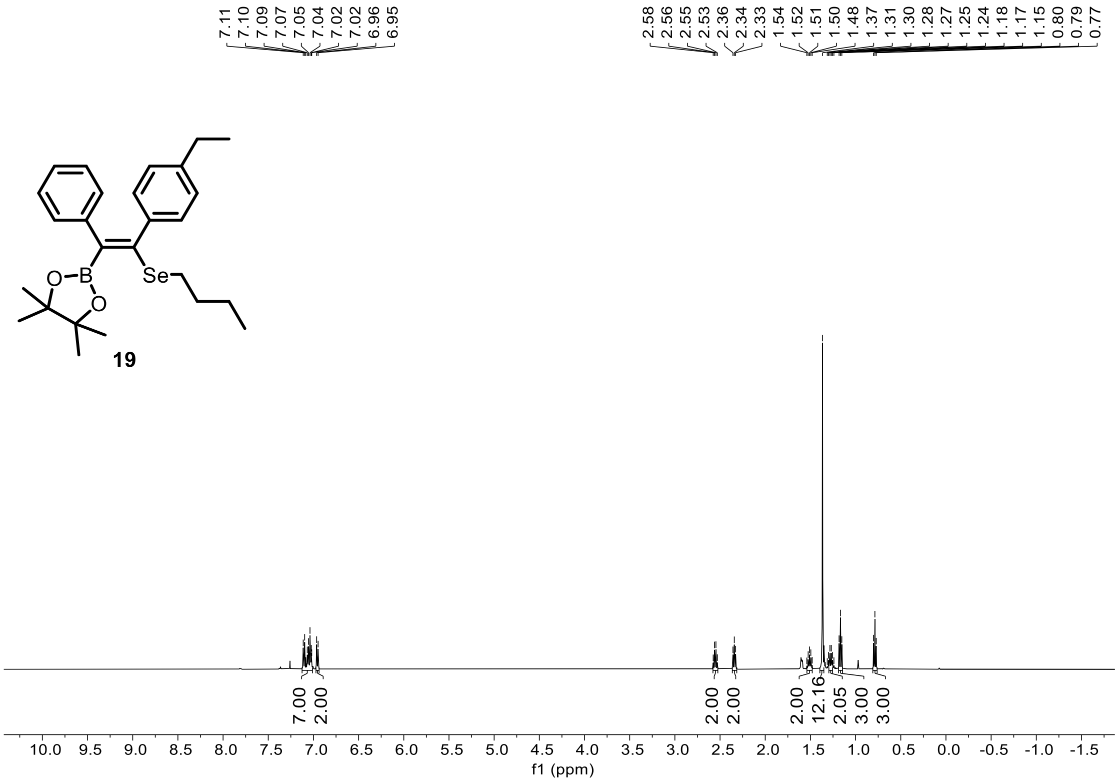

126 MHz, 298 K, $CDCl_3$ as solvent

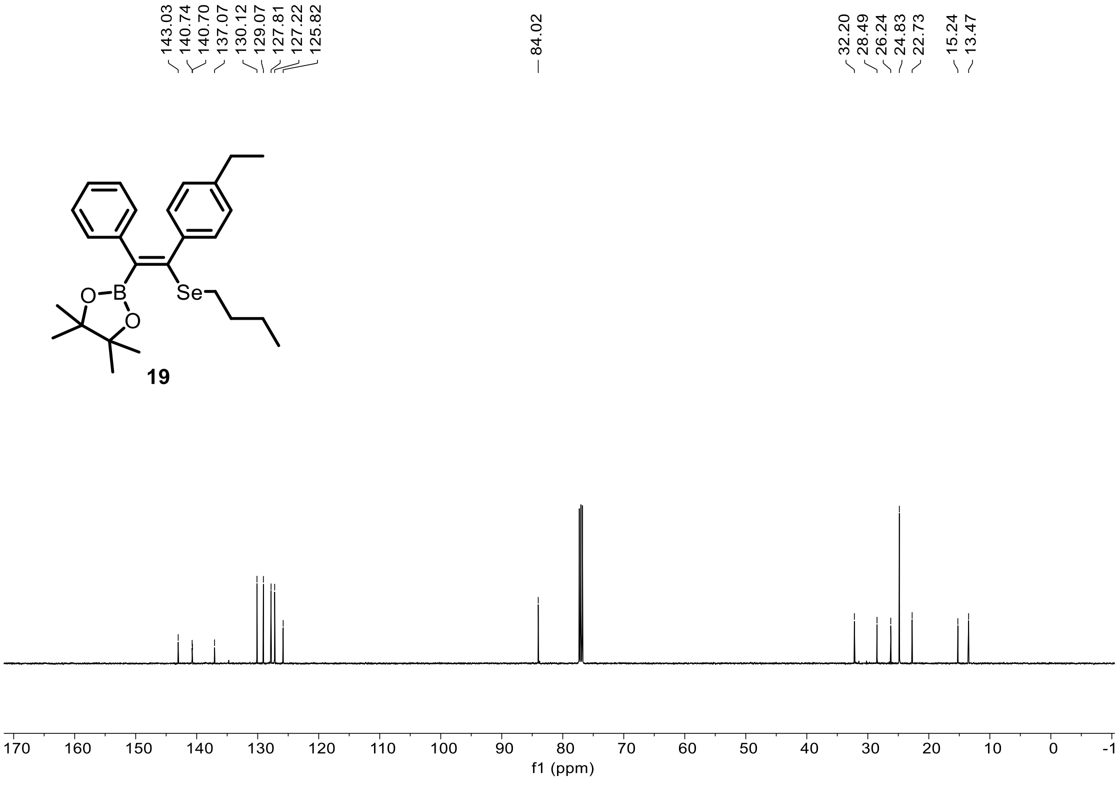

500 MHz, 298 K, $CDCl_3$ as solvent

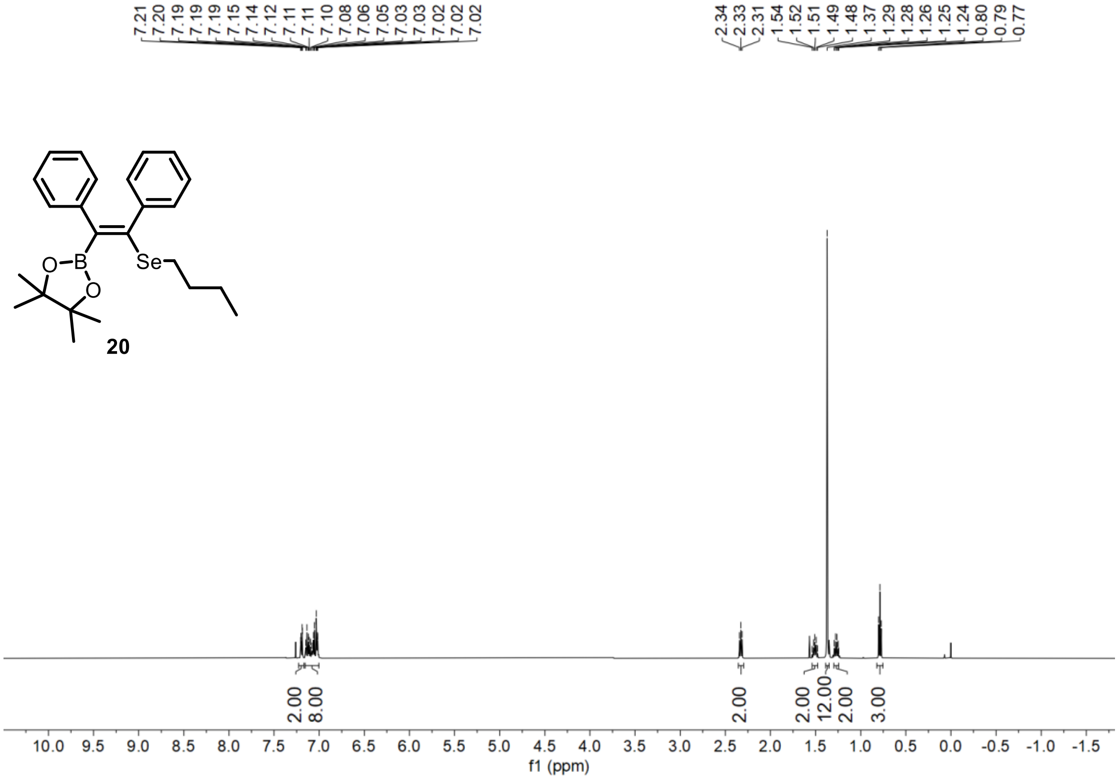

126 MHz, 298 K, $CDCl_3$ as solvent

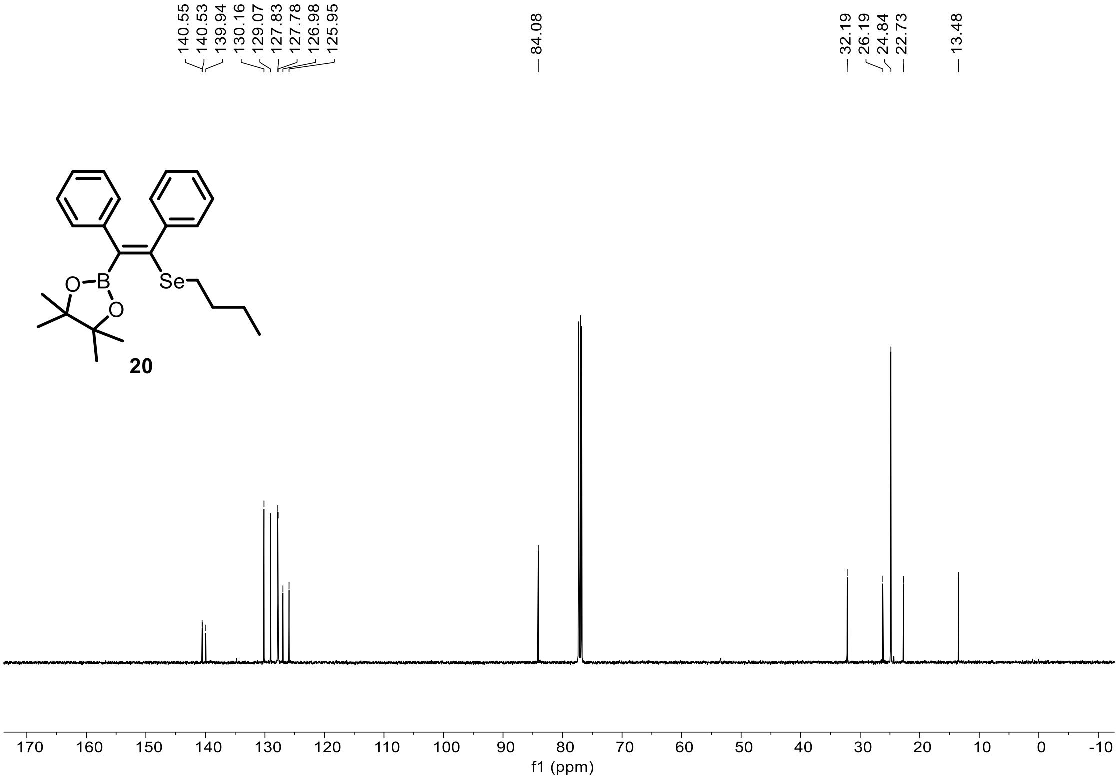

500 MHz, 298 K, $CDCl_3$ as solvent

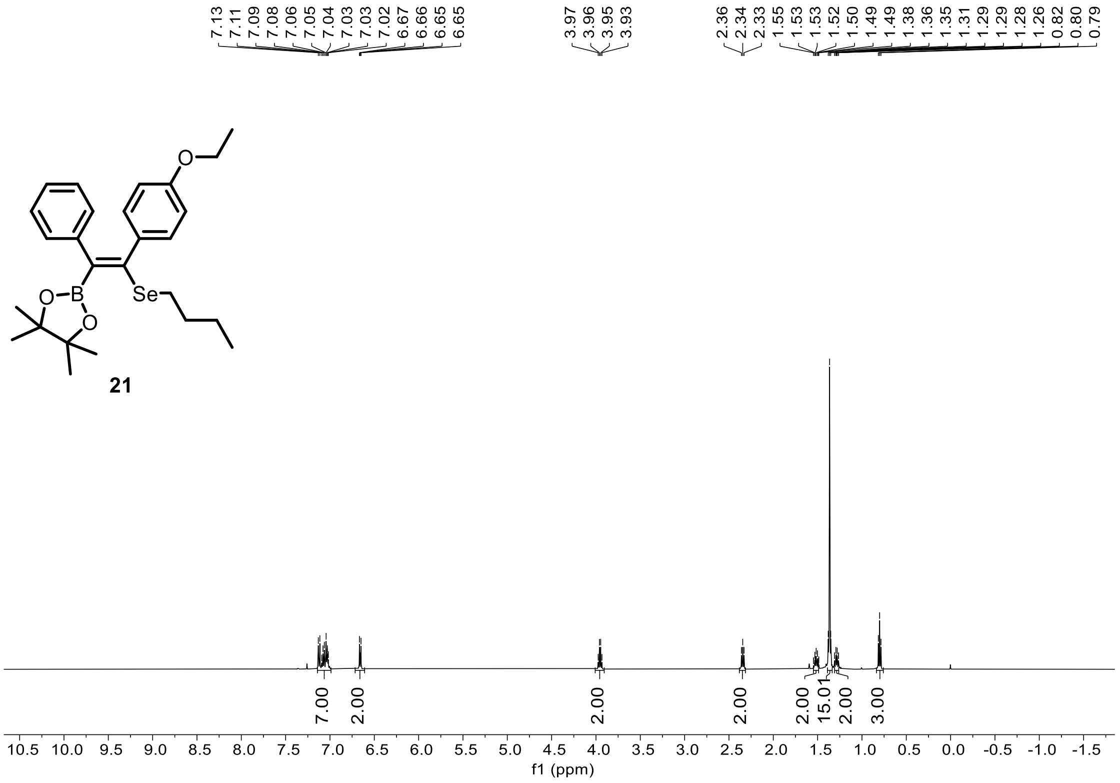

126 MHz, 298 K, $CDCl_3$ as solvent

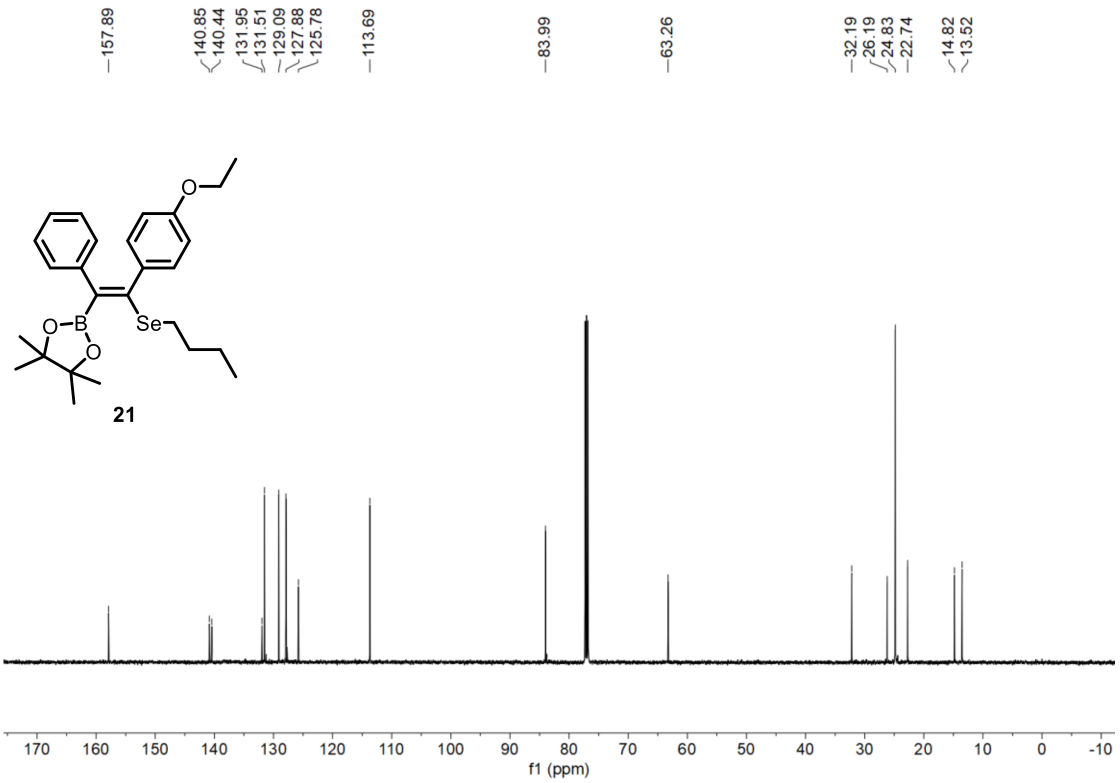

500 MHz, 298 K, $CDCl_3$ as solvent

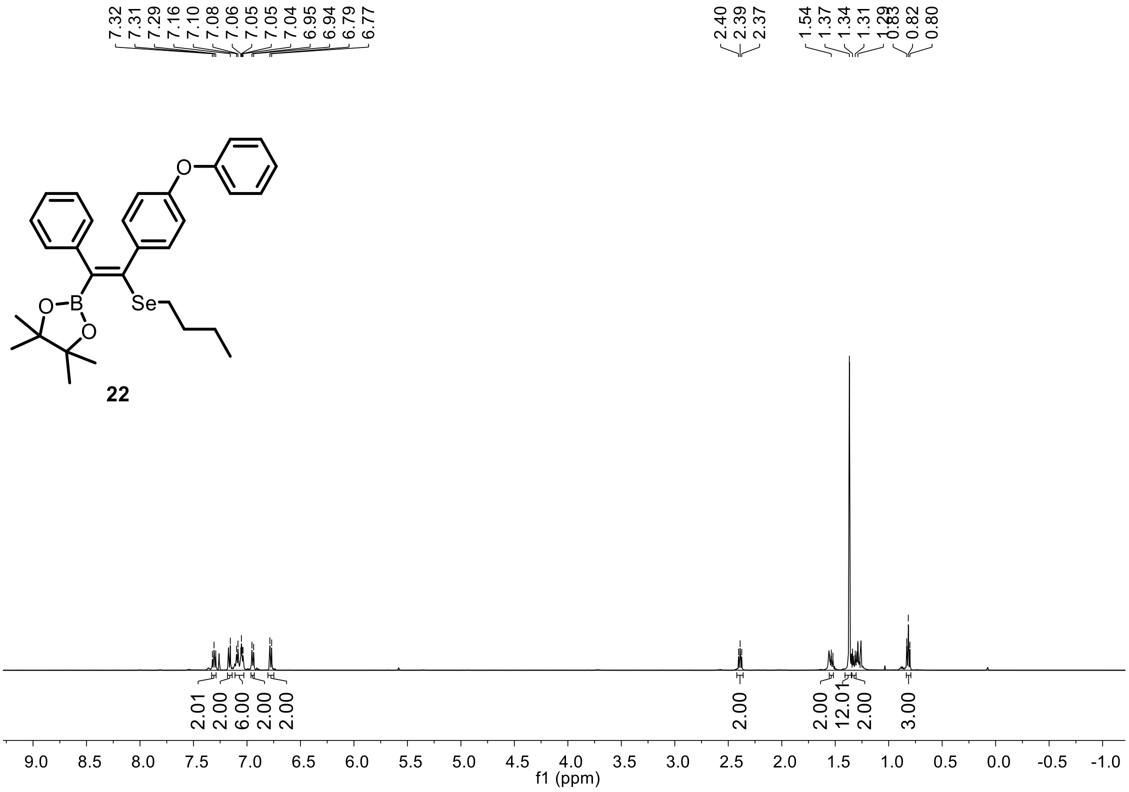

126 MHz, 298 K, $CDCl_3$ as solvent

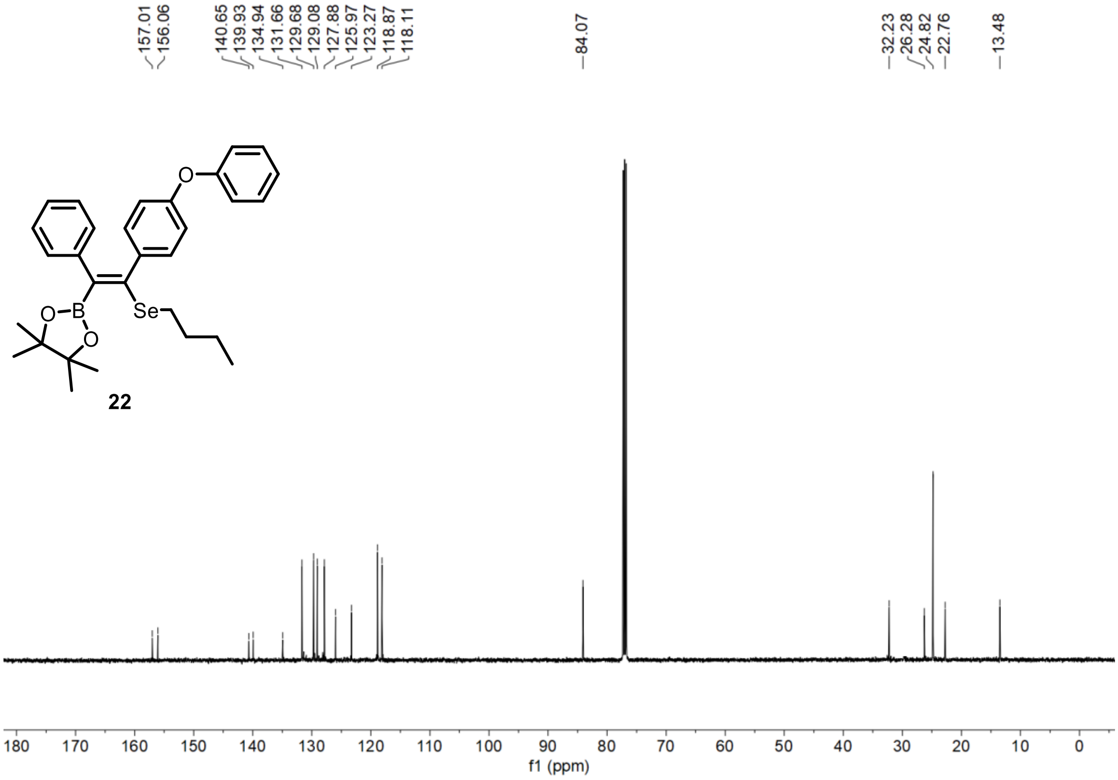

500 MHz, 298 K, $CDCl_3$ as solvent

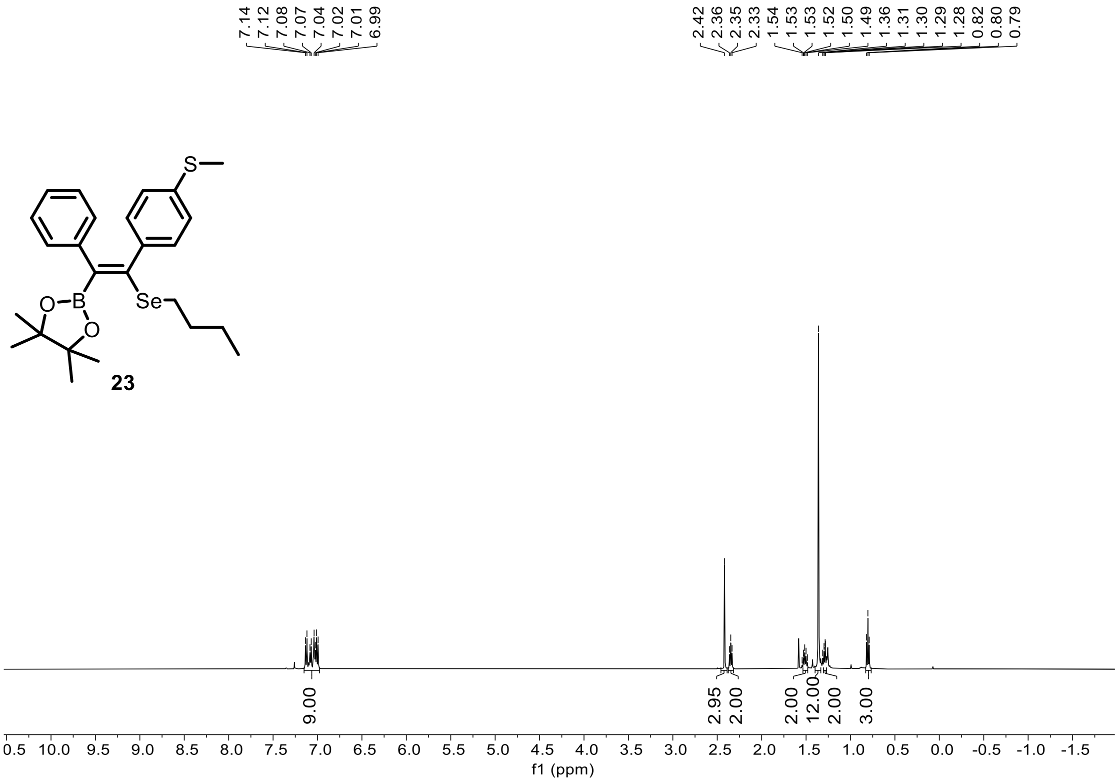

126 MHz, 298 K, $CDCl_3$ as solvent

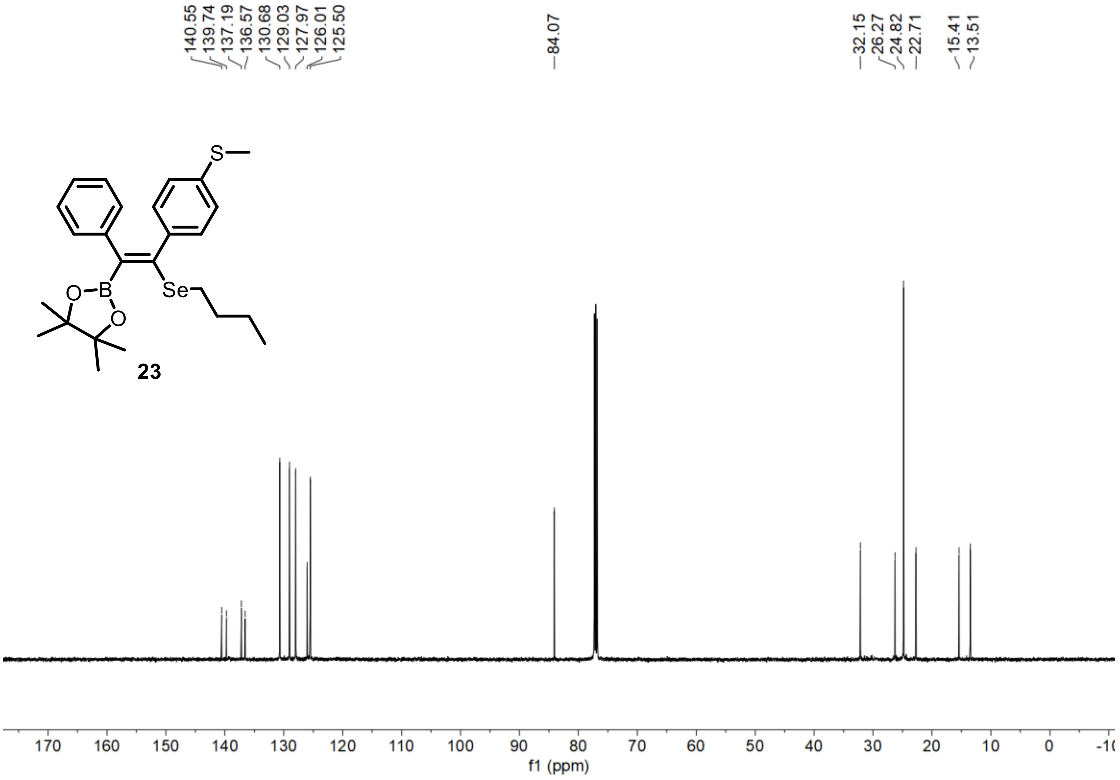

500 MHz, 298 K, $CDCl_3$ as solvent

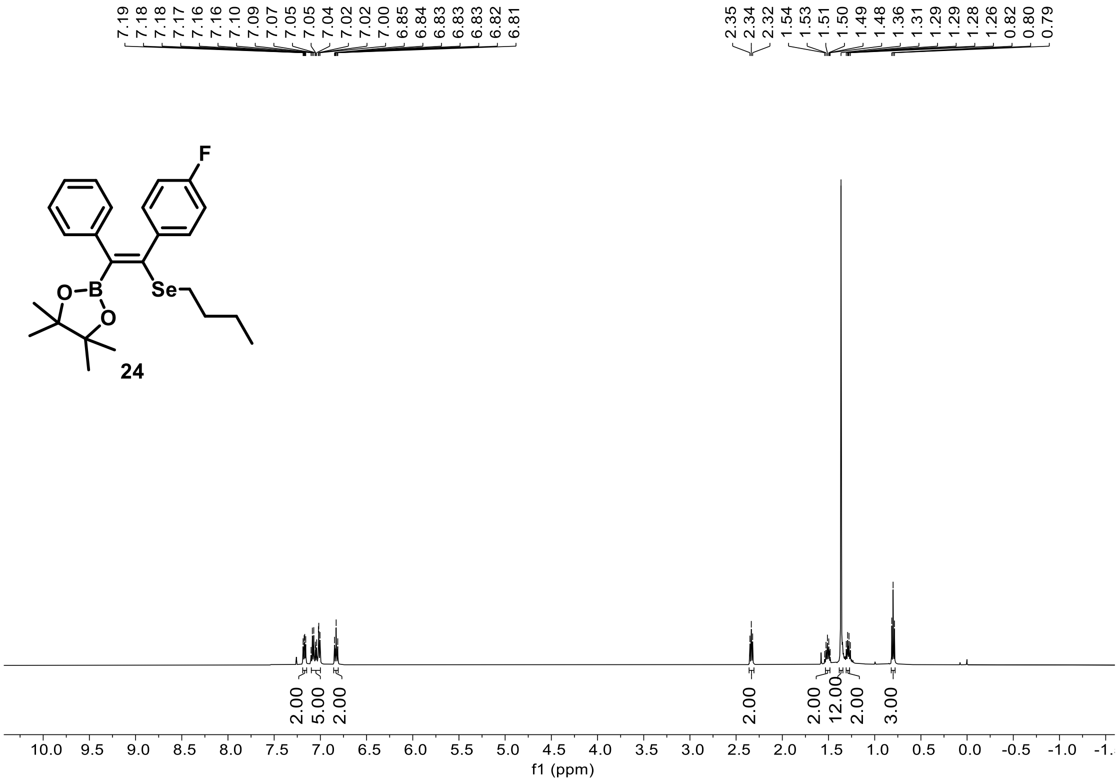

126 MHz, 298 K, $CDCl_3$ as solvent

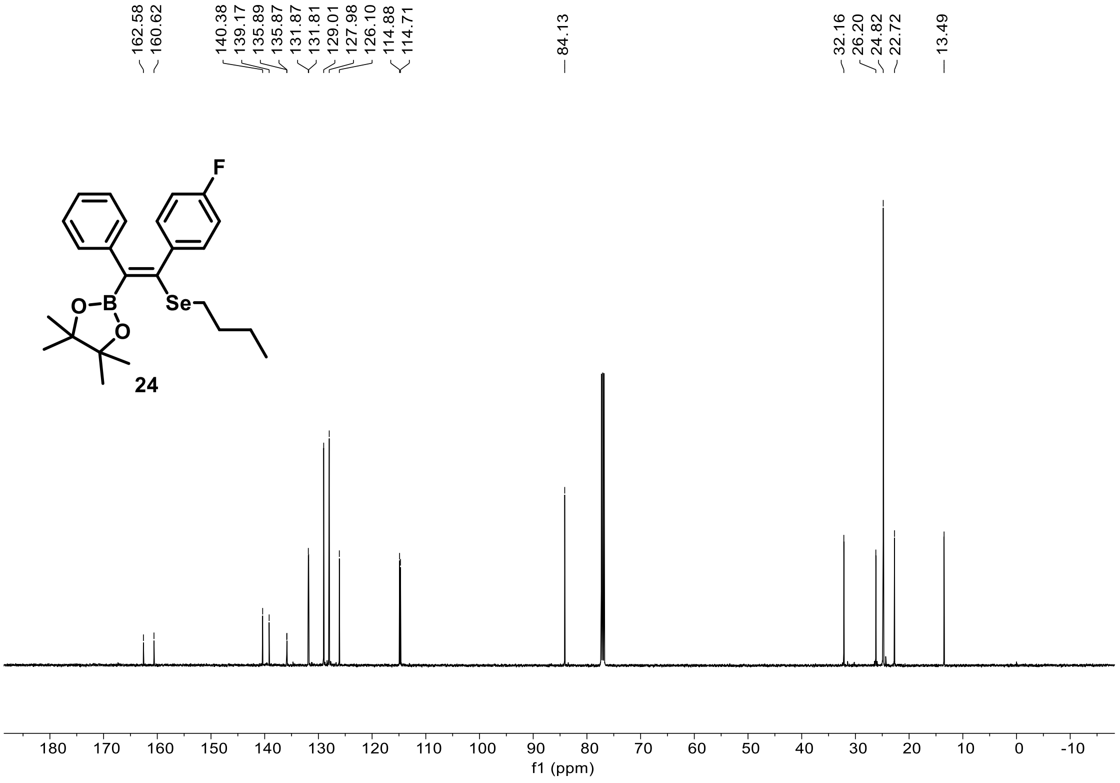

500 MHz, 298 K, $CDCl_3$ as solvent

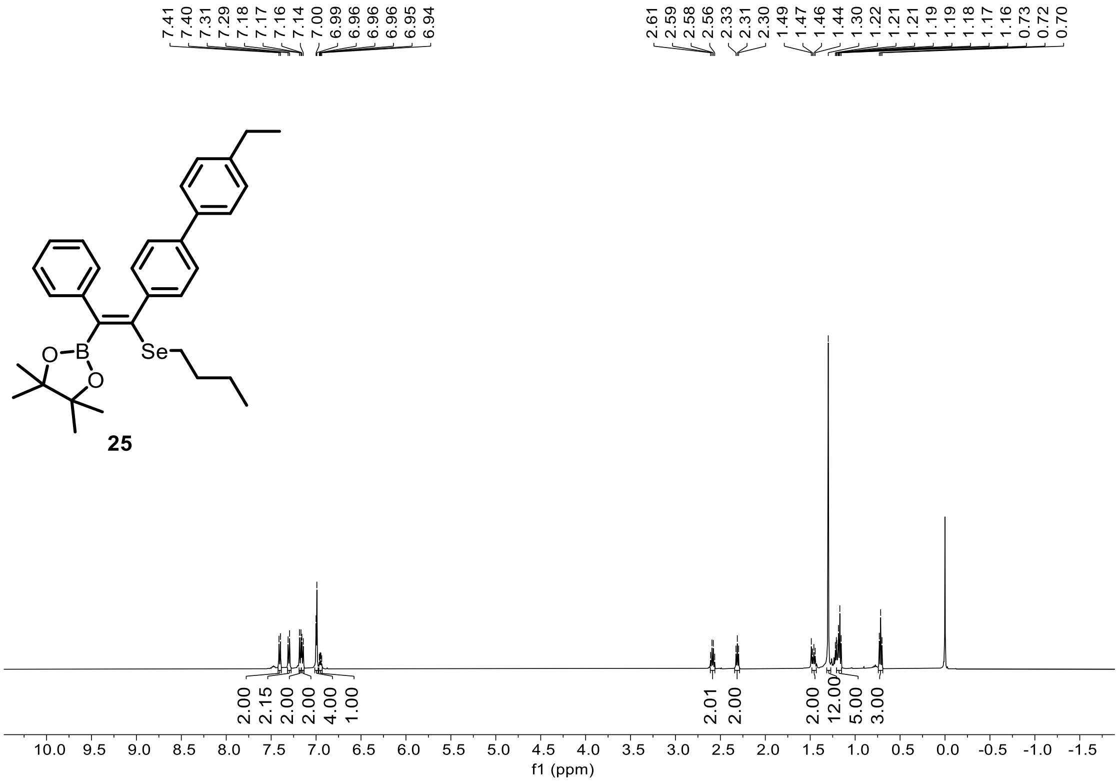

126 MHz, 298 K, $CDCl_3$ as solvent

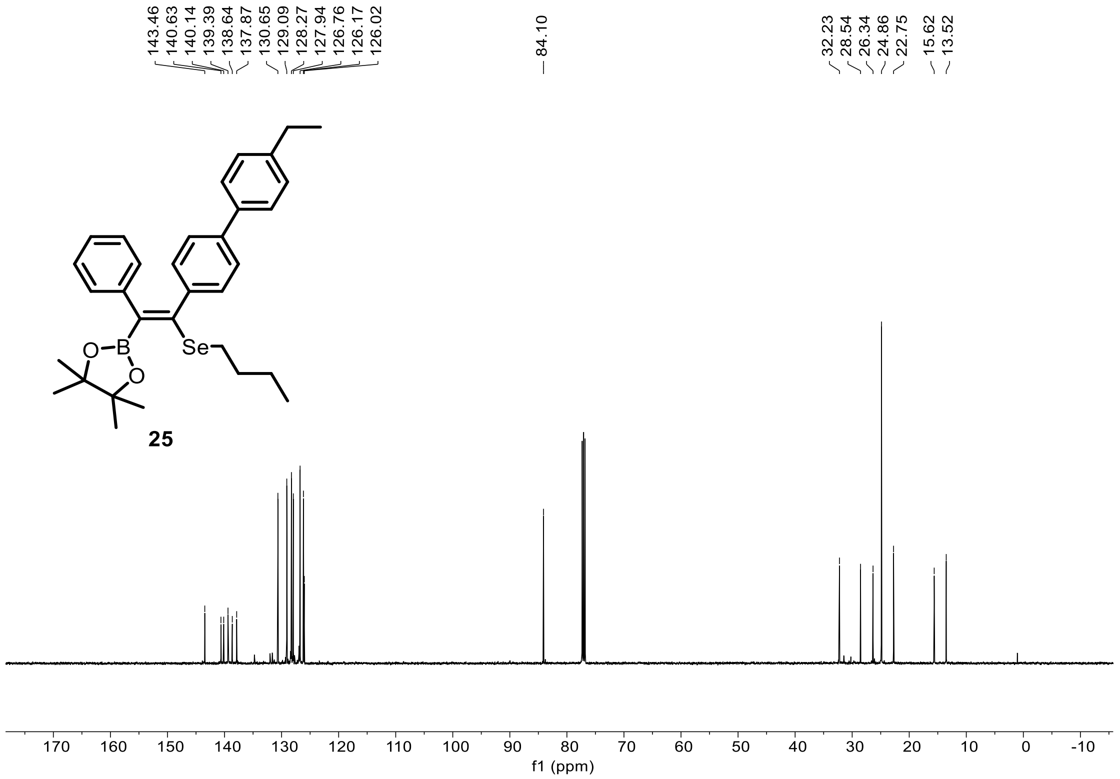

500 MHz, 298 K, $CDCl_3$ as solvent

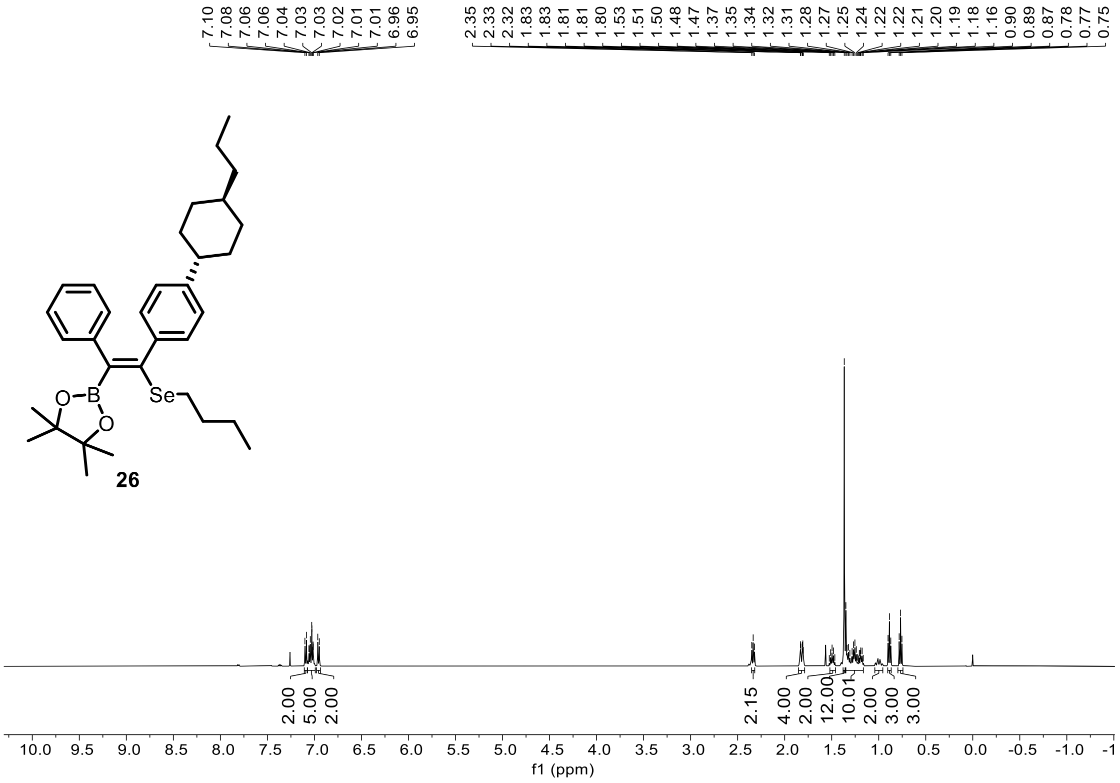

126 MHz, 298 K, $CDCl_3$ as solvent

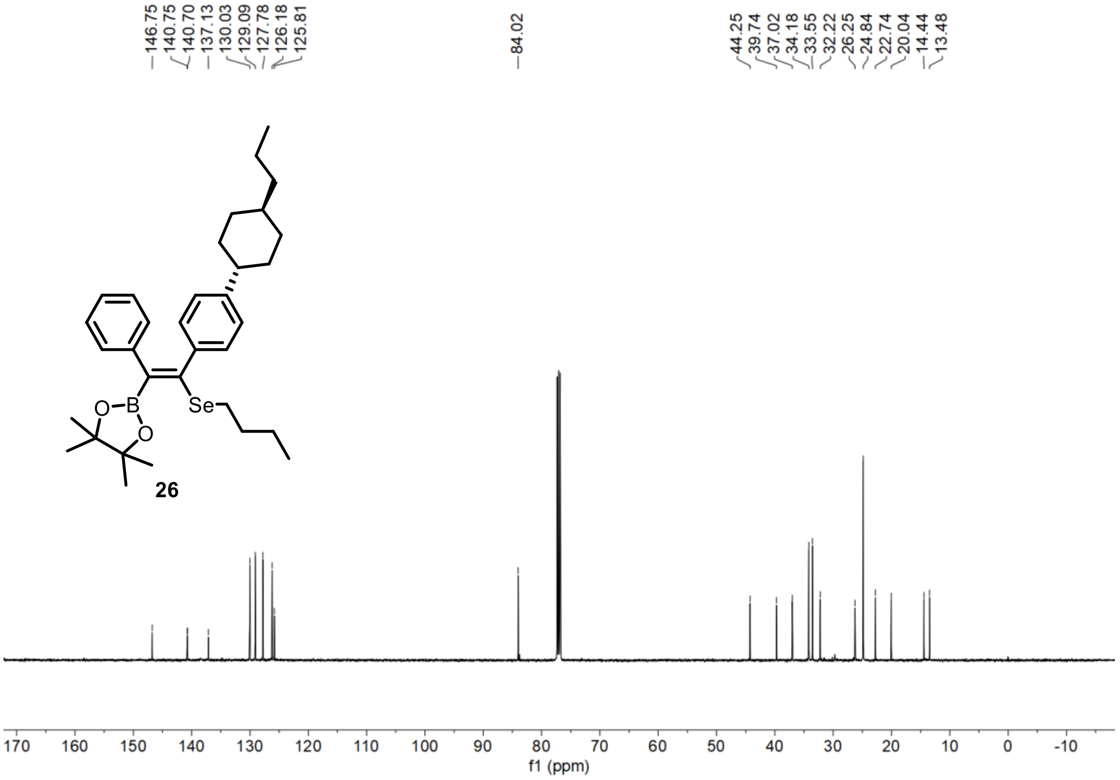

500 MHz, 298 K, $CDCl_3$ as solvent

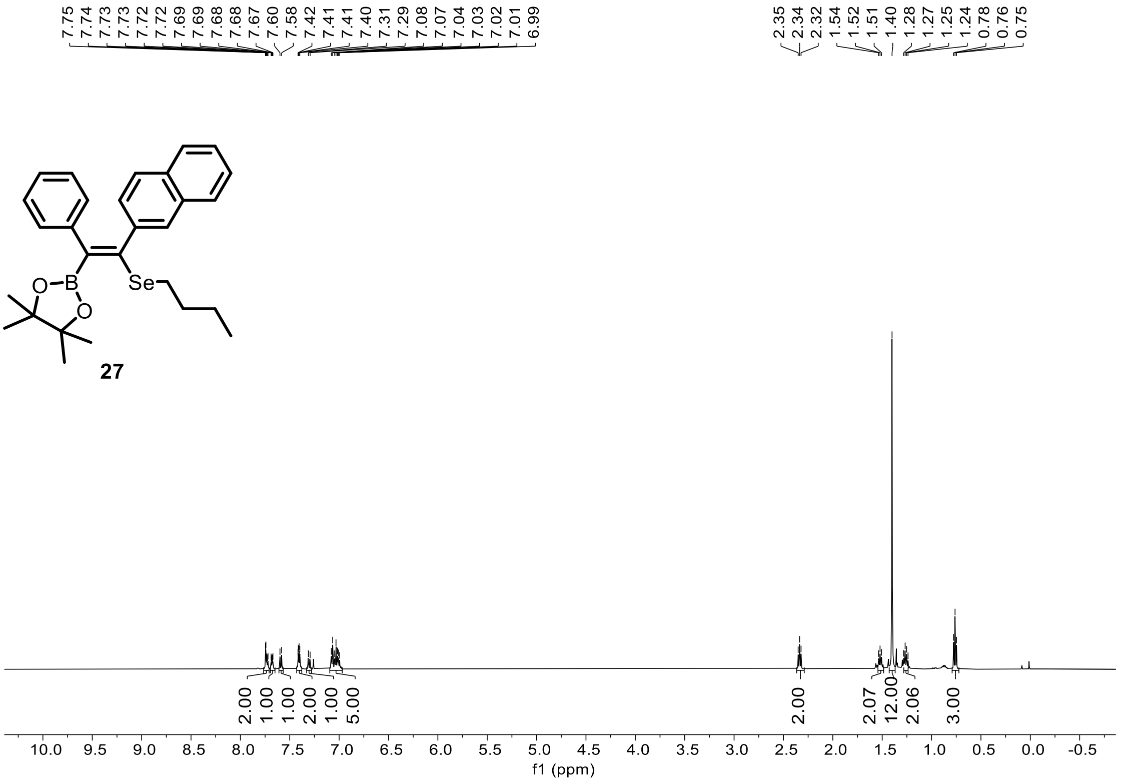

126 MHz, 298 K, $CDCl_3$ as solvent

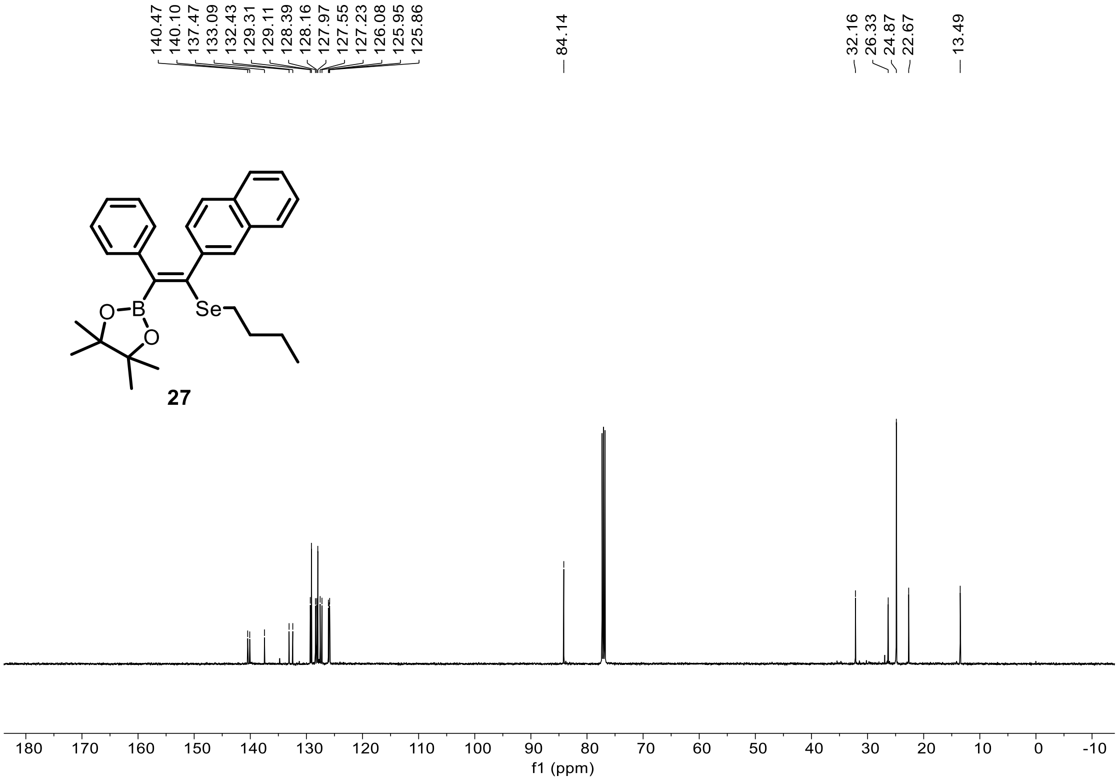

500 MHz, 298 K, $CDCl_3$ as solvent

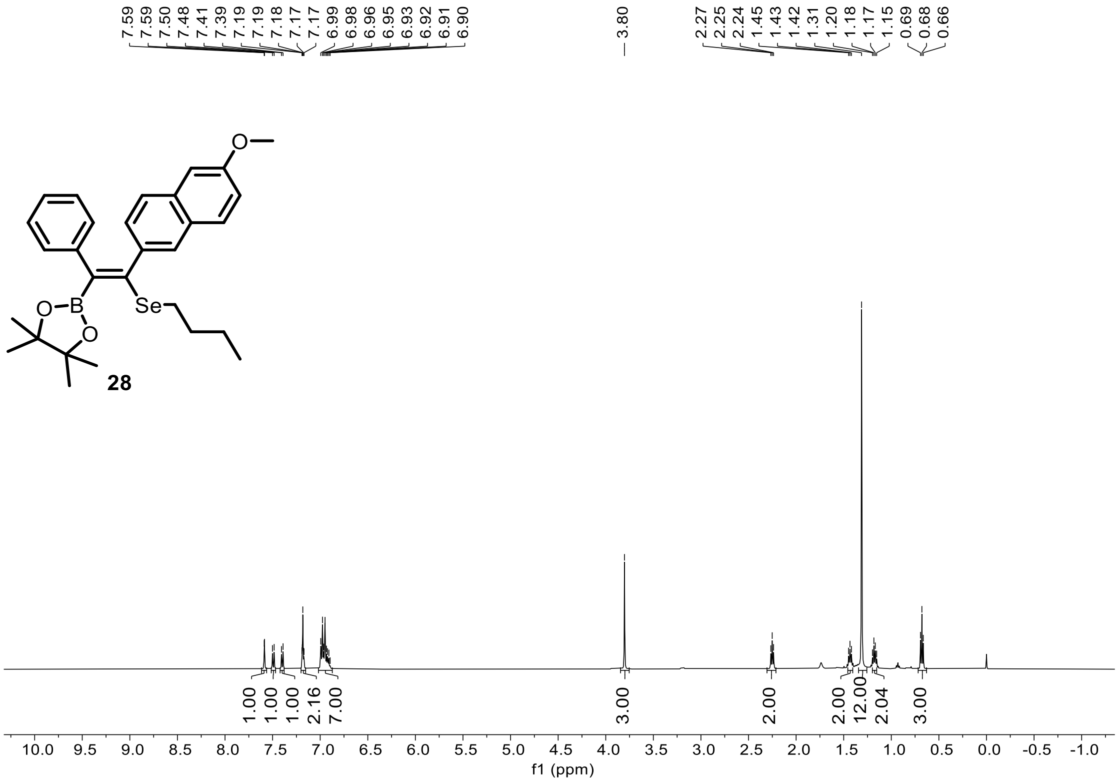

126 MHz, 298 K, $CDCl_3$ as solvent

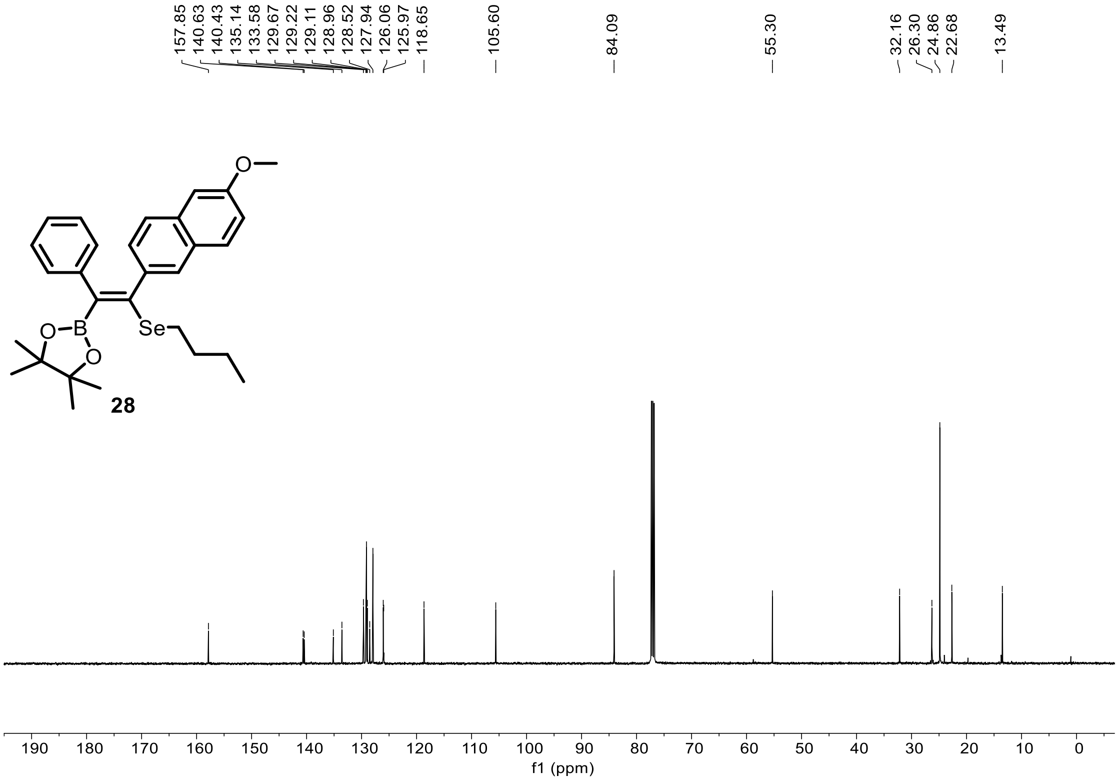

500 MHz, 298 K, $CDCl_3$ as solvent

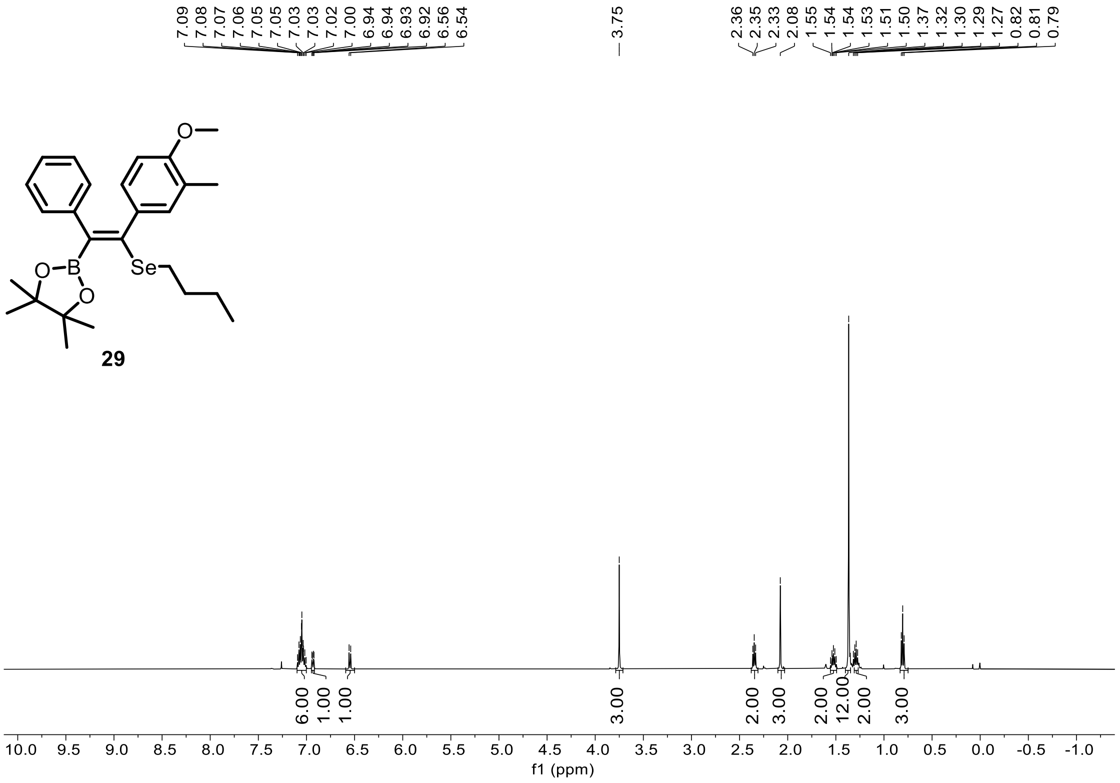

126 MHz, 298 K, $CDCl_3$ as solvent

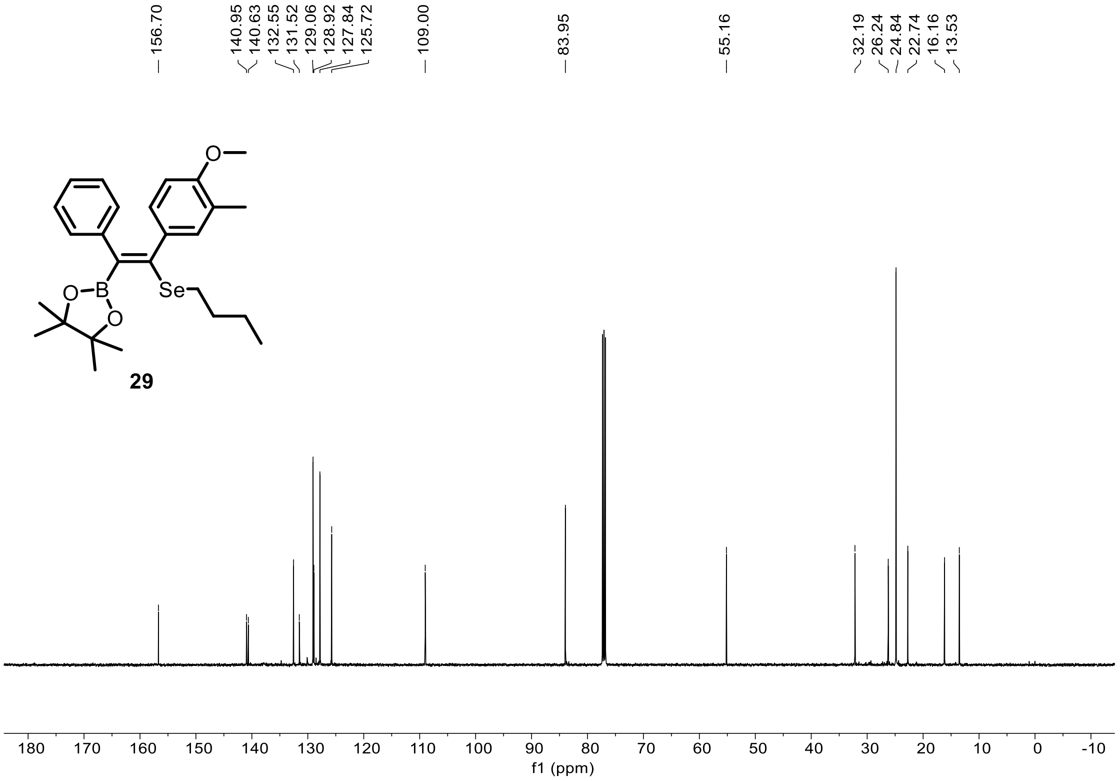

500 MHz, 298 K, $CDCl_3$ as solvent

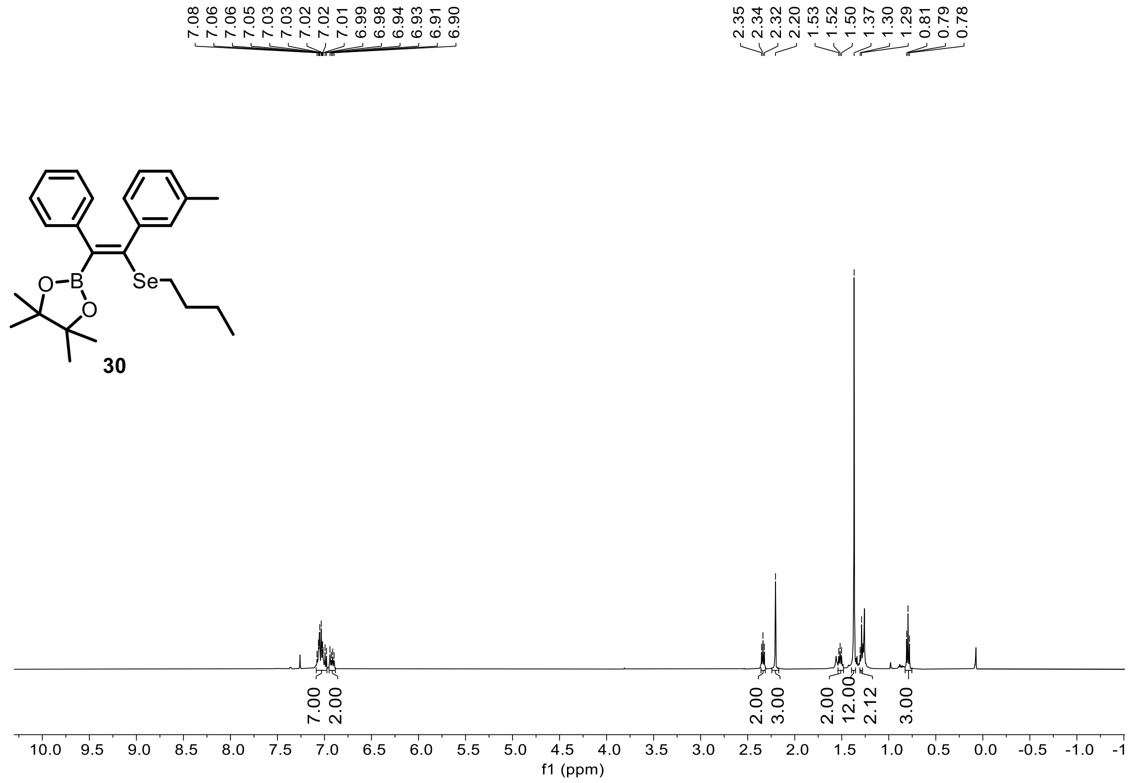

126 MHz, 298 K, $CDCl_3$ as solvent

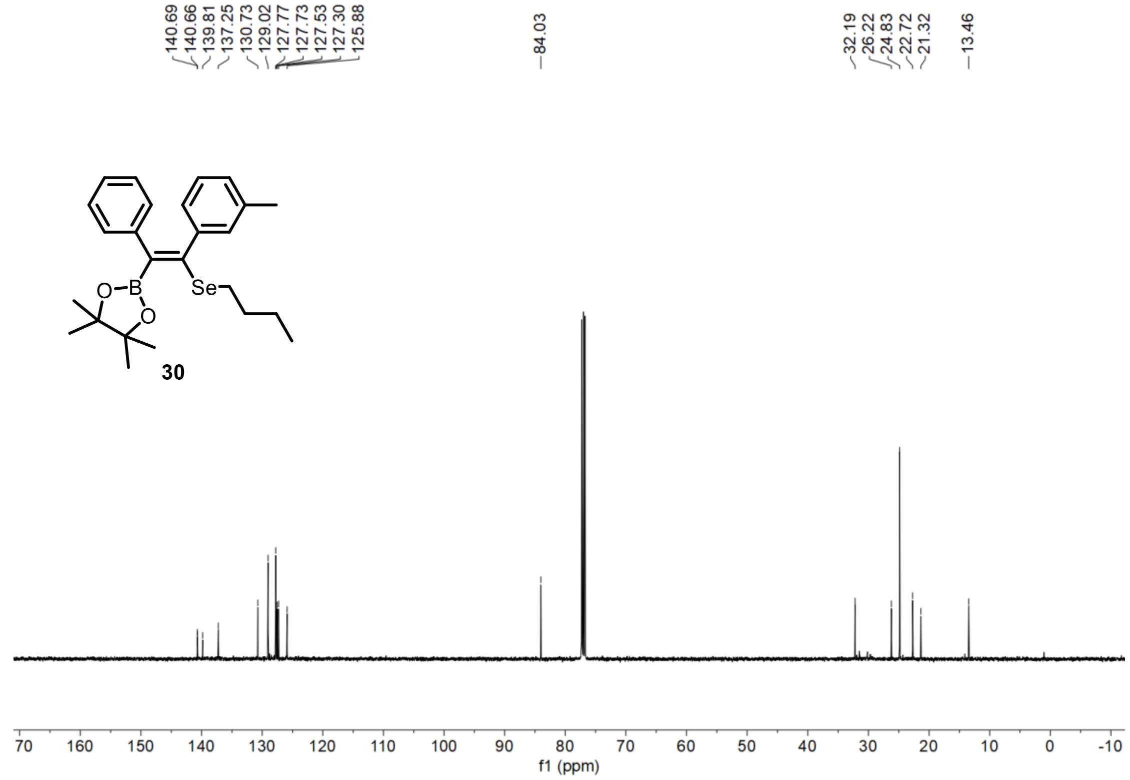

500 MHz, 298 K, $CDCl_3$ as solvent

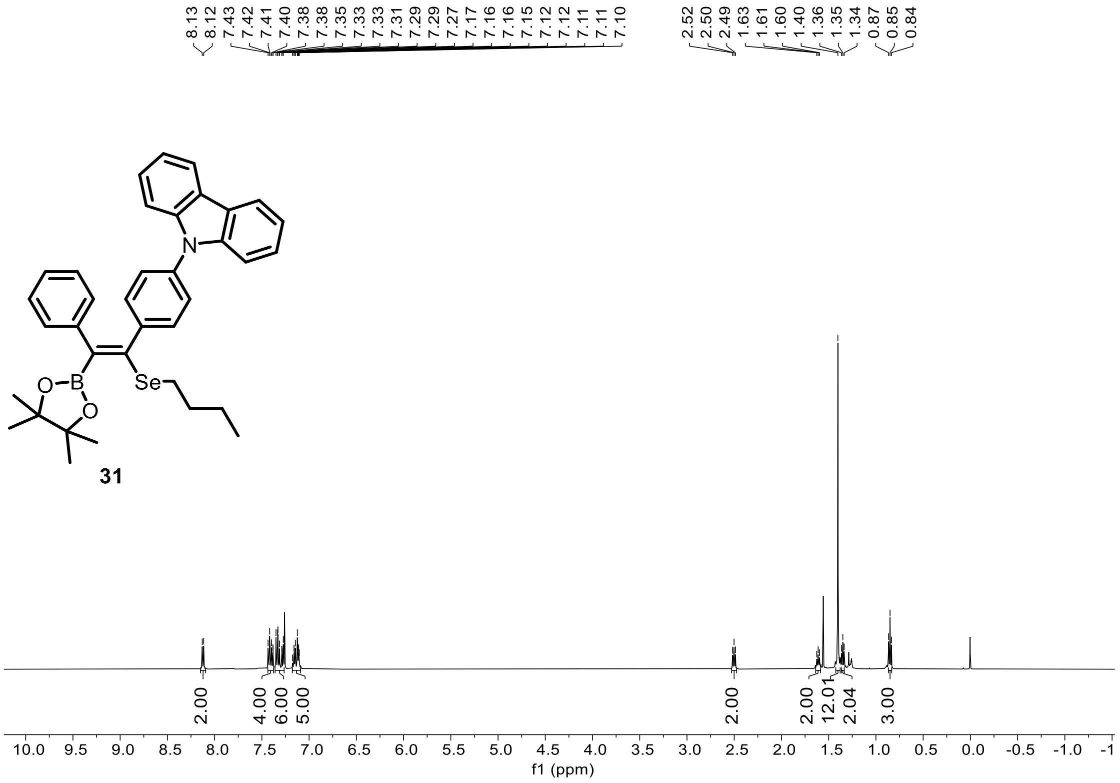

126 MHz, 298 K, $CDCl_3$ as solvent

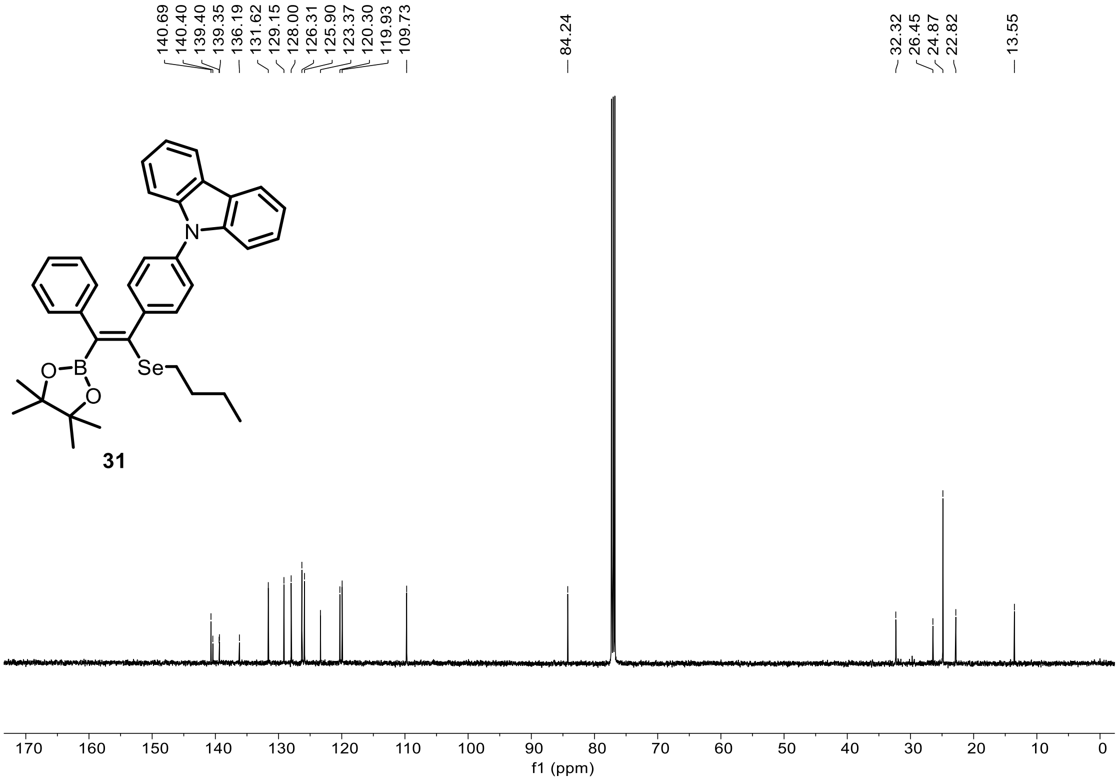

500 MHz, 298 K, $CDCl_3$ as solvent

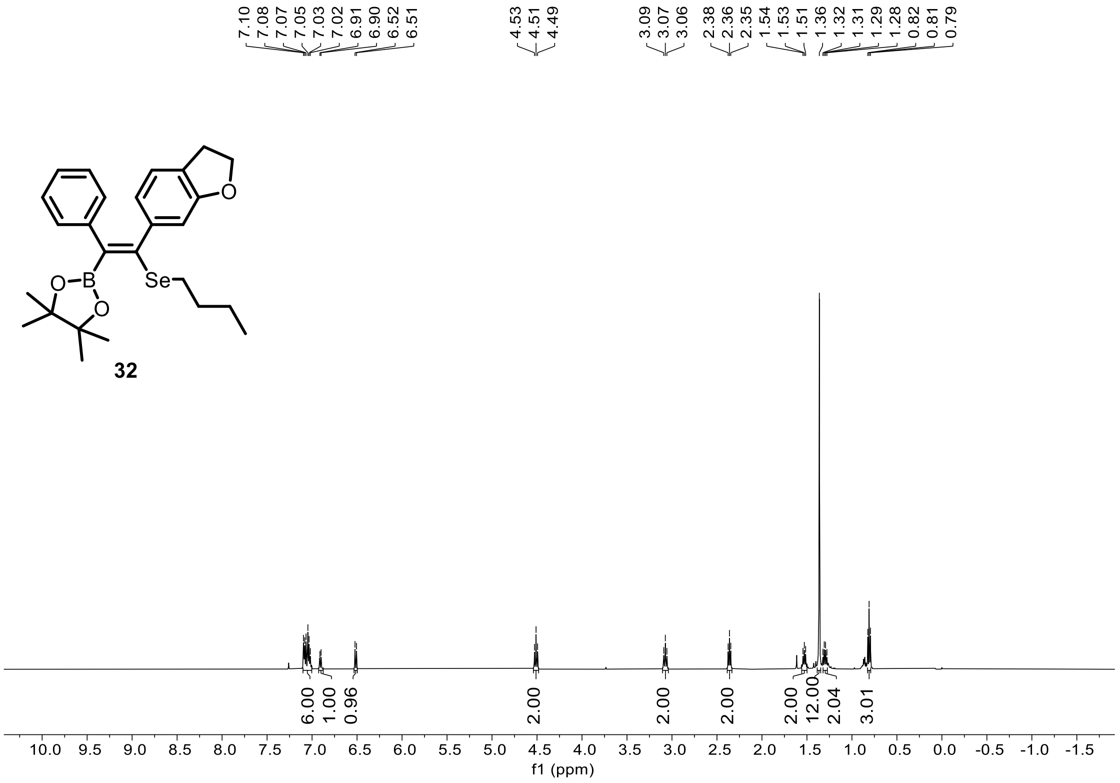

126 MHz, 298 K, $CDCl_3$ as solvent

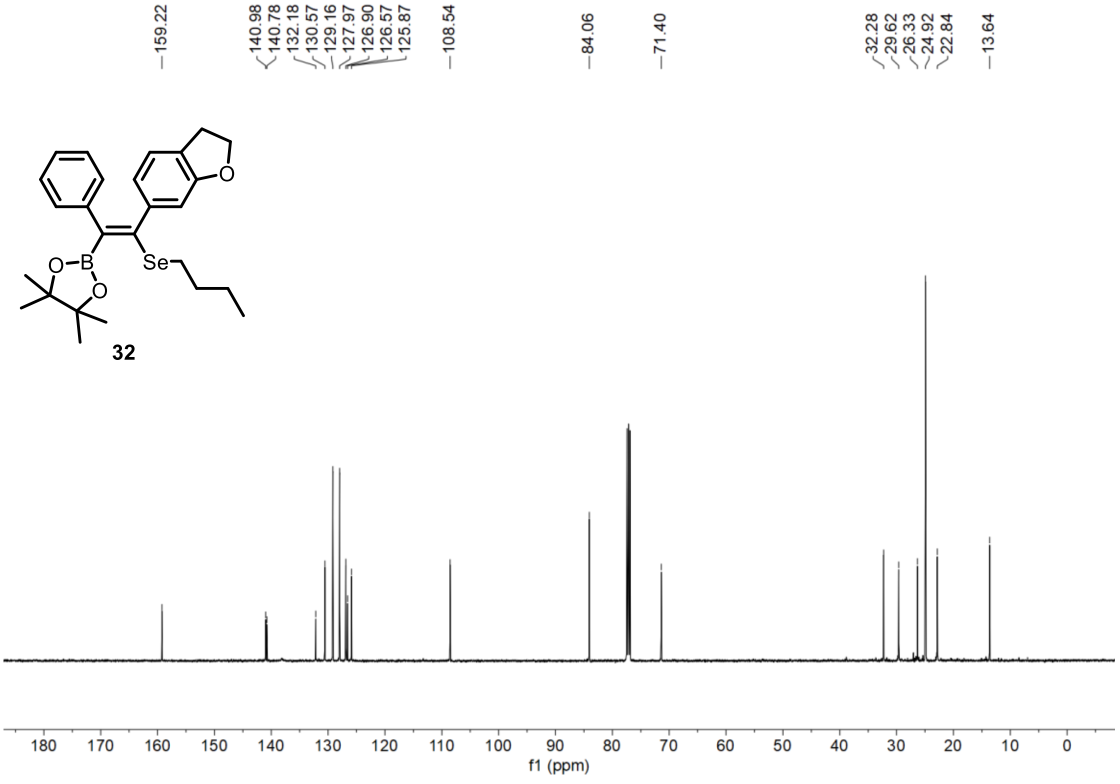

500 MHz, 298 K, $CDCl_3$ as solvent

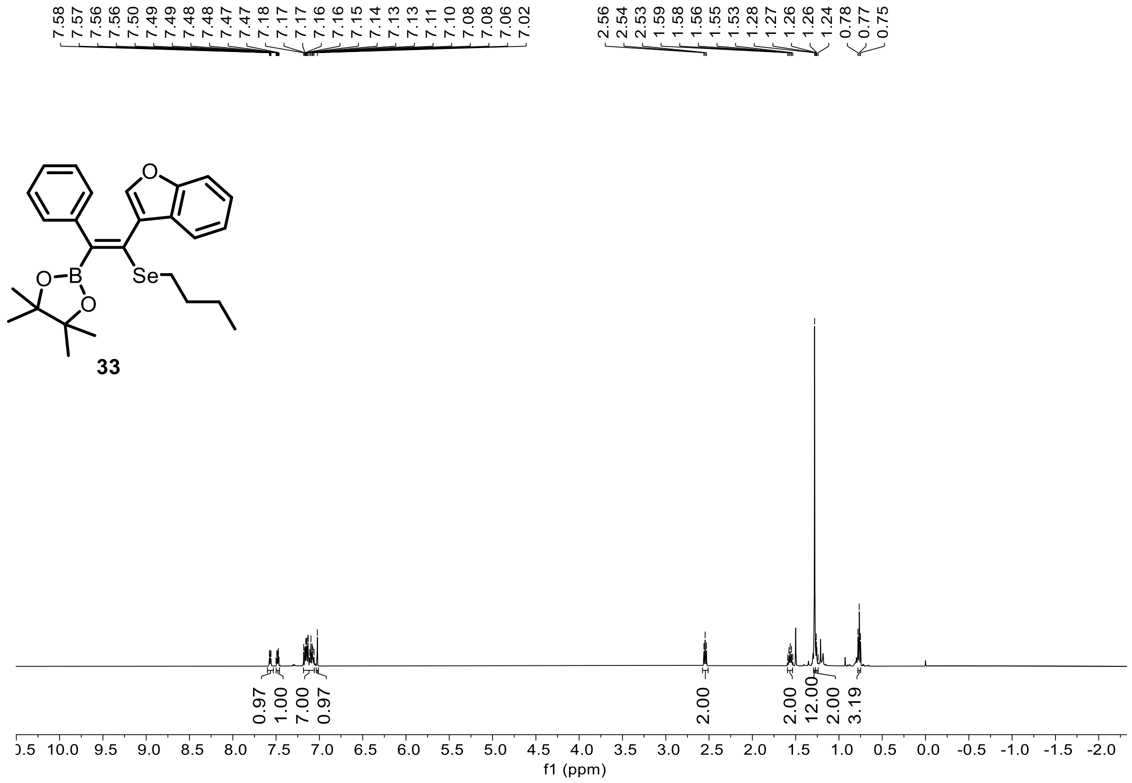

126 MHz, 298 K, $CDCl_3$ as solvent

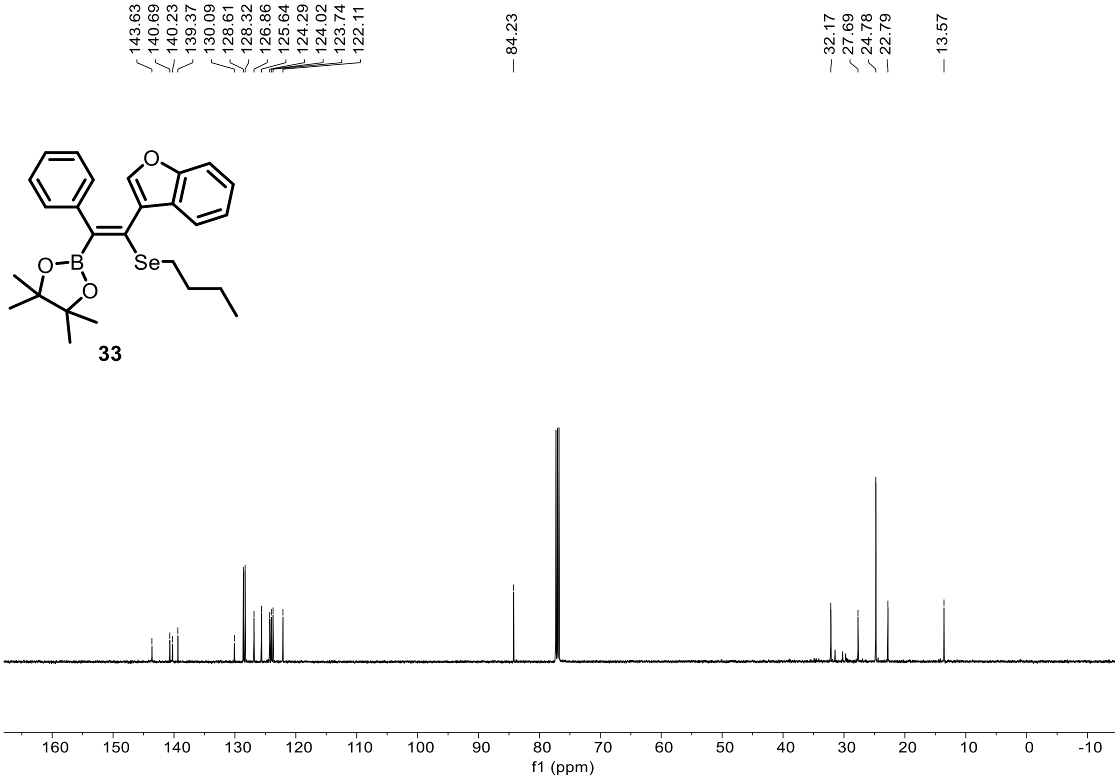

500 MHz, 298 K, $CDCl_3$ as solvent

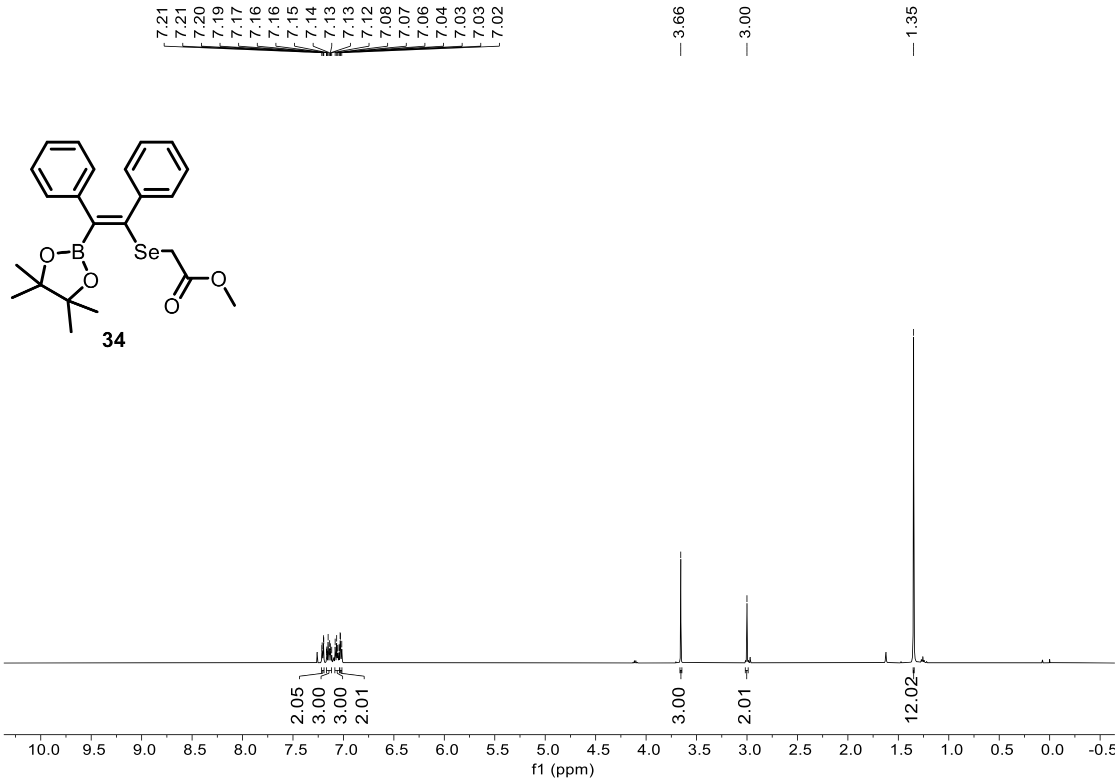

126 MHz, 298 K, $CDCl_3$ as solvent

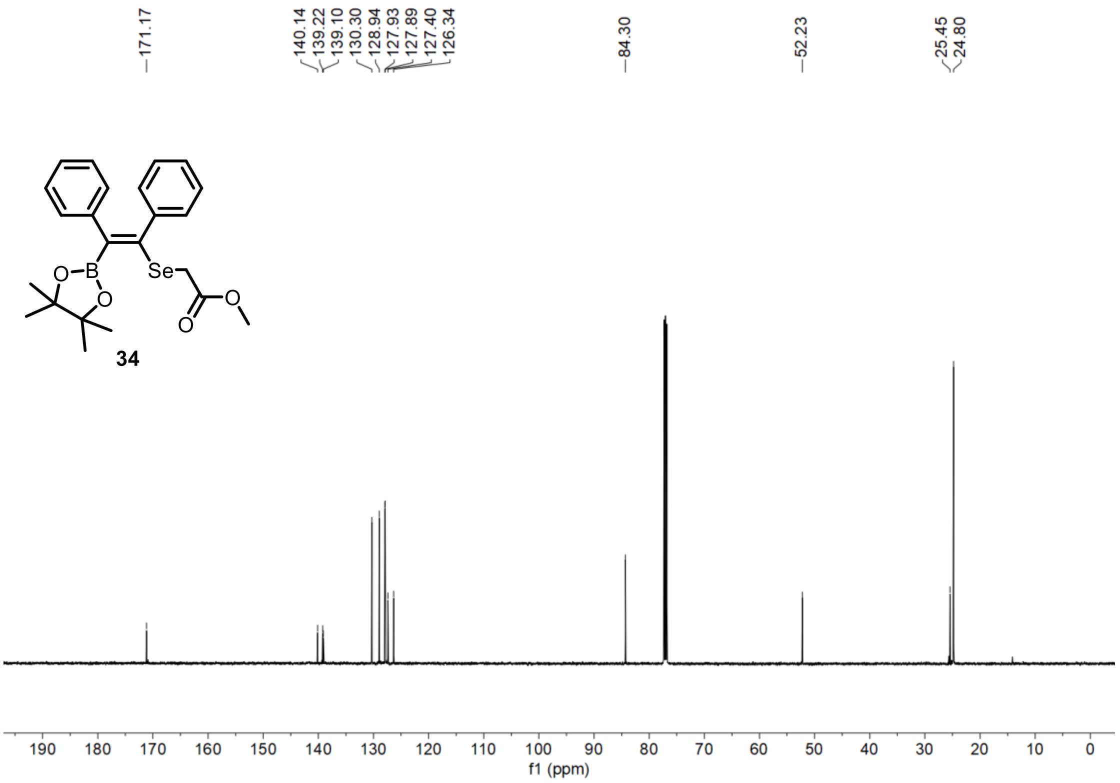

500 MHz, 298 K, CDCl3 as solvent

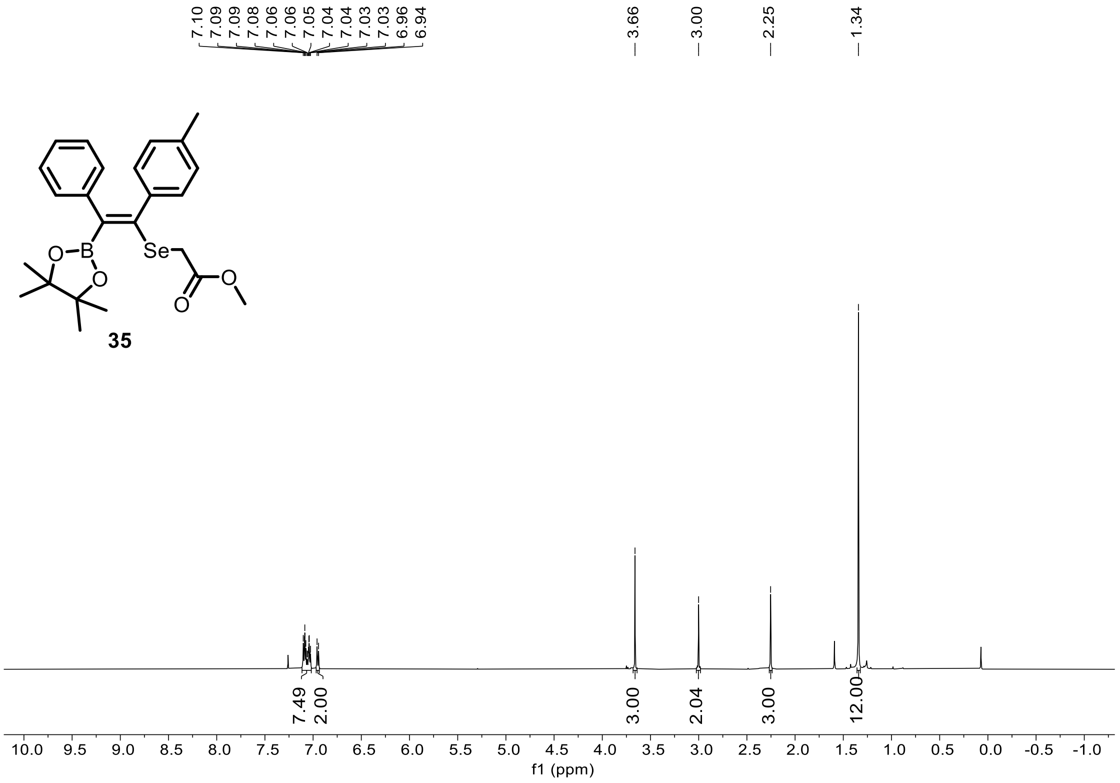

126 MHz, 298 K, CDCl3 as solvent

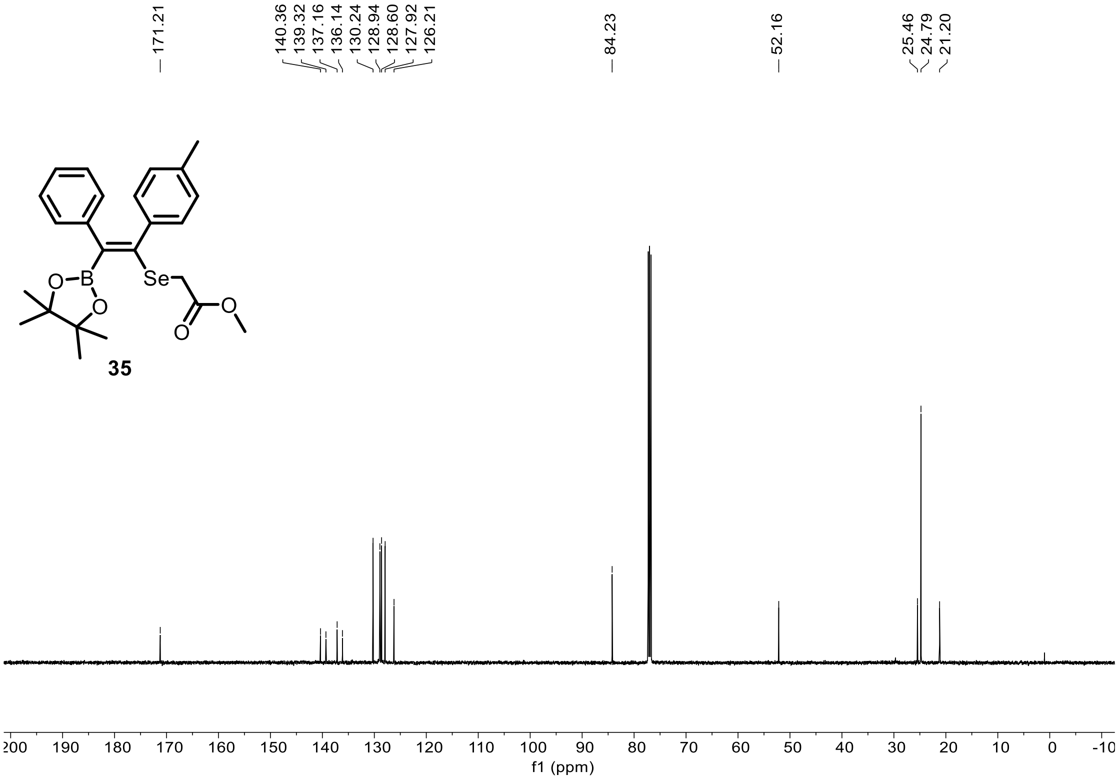

500 MHz, 298 K, $CDCl_3$ as solvent

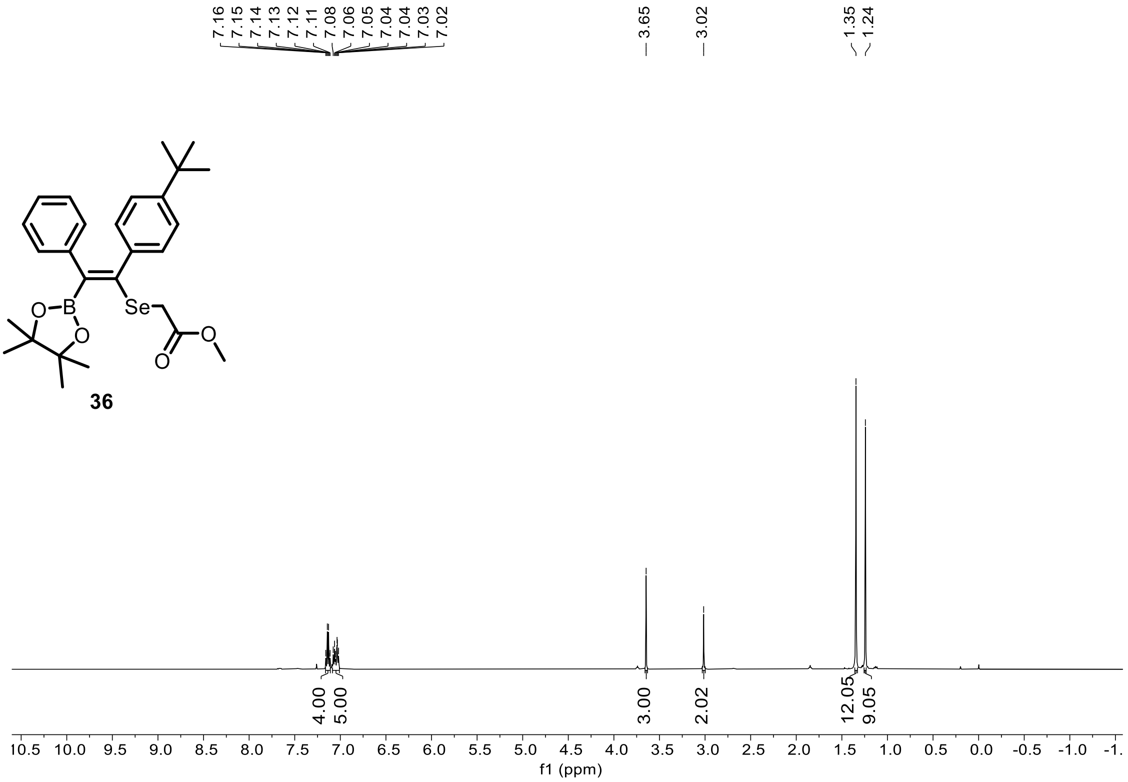

126 MHz, 298 K, $CDCl_3$ as solvent

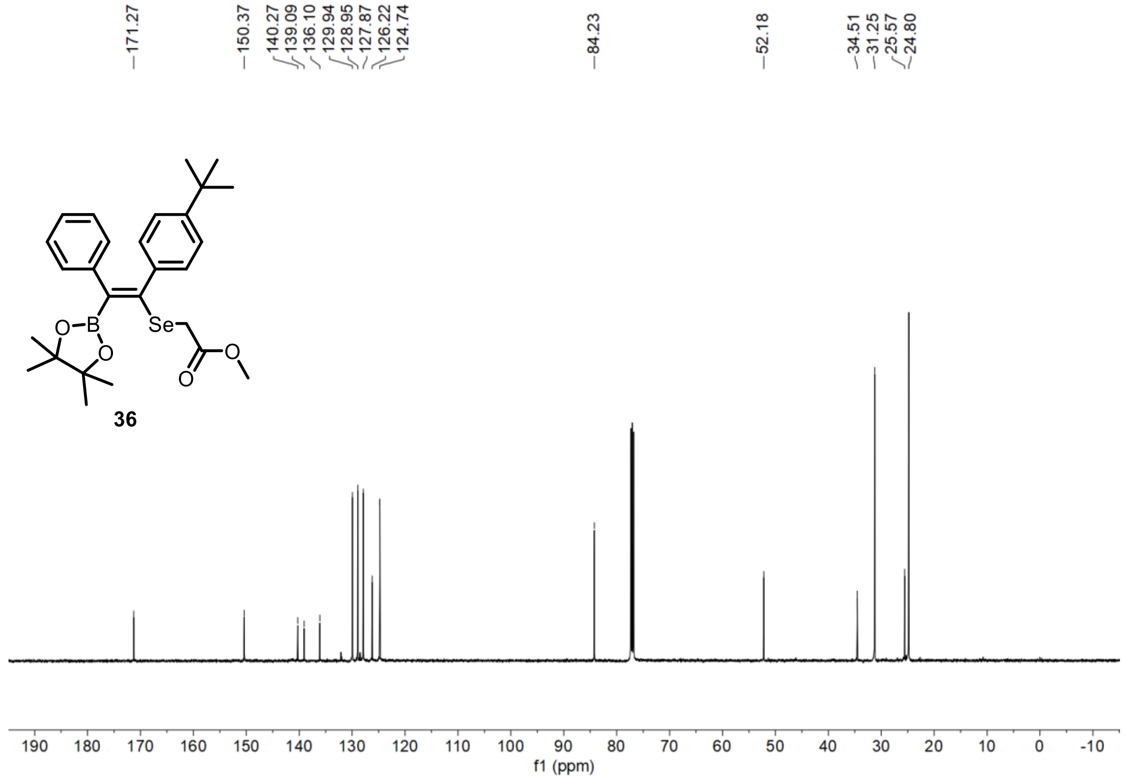

500 MHz, 298 K, $CDCl_3$ as solvent

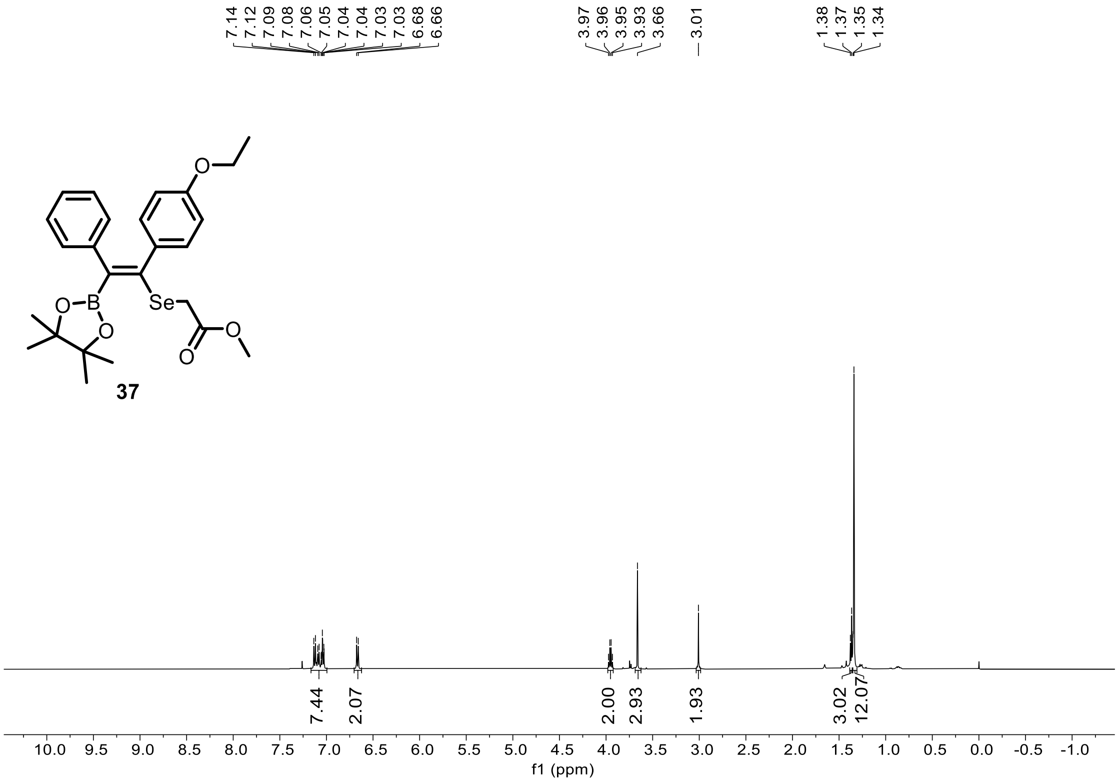

126 MHz, 298 K, $CDCl_3$ as solvent

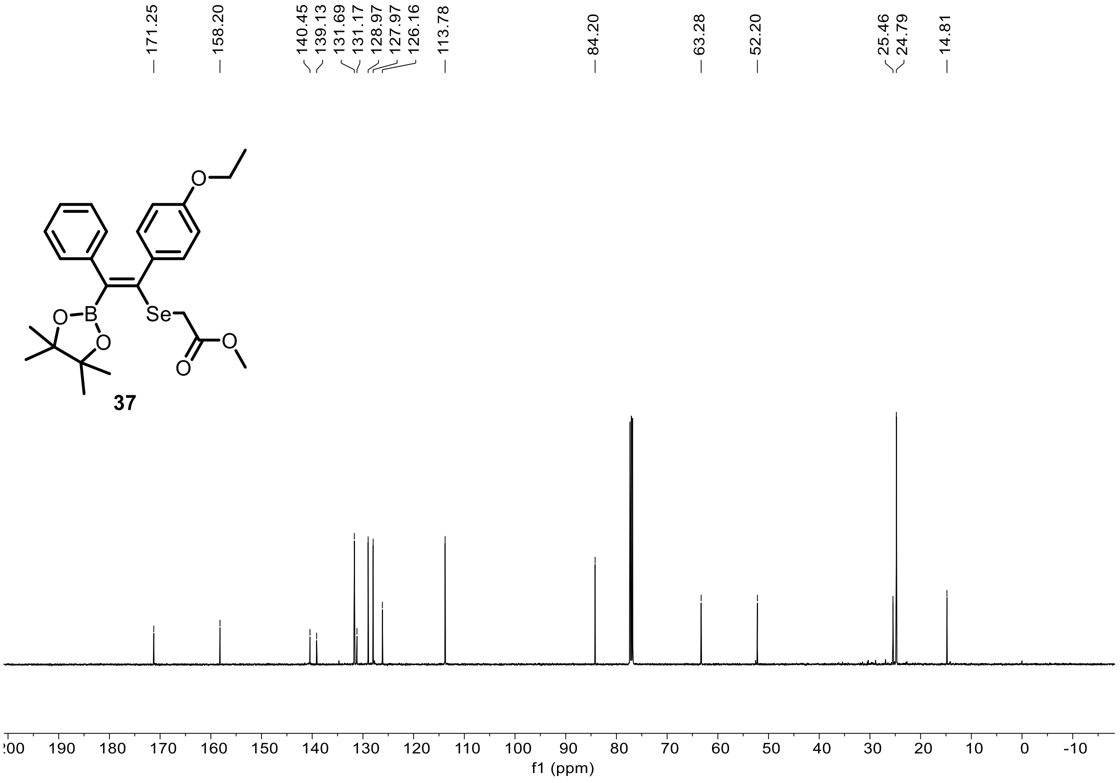

500 MHz, 298 K, CDCl3 as solvent

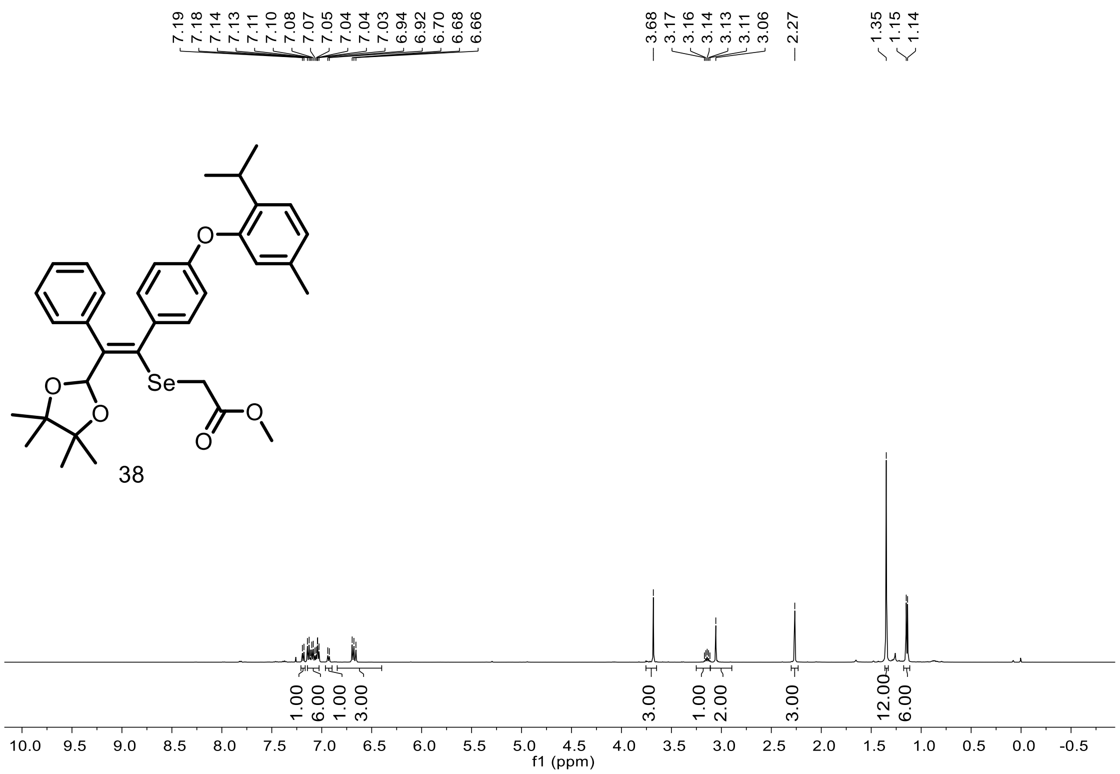

126 MHz, 298 K, CDCl3 as solvent

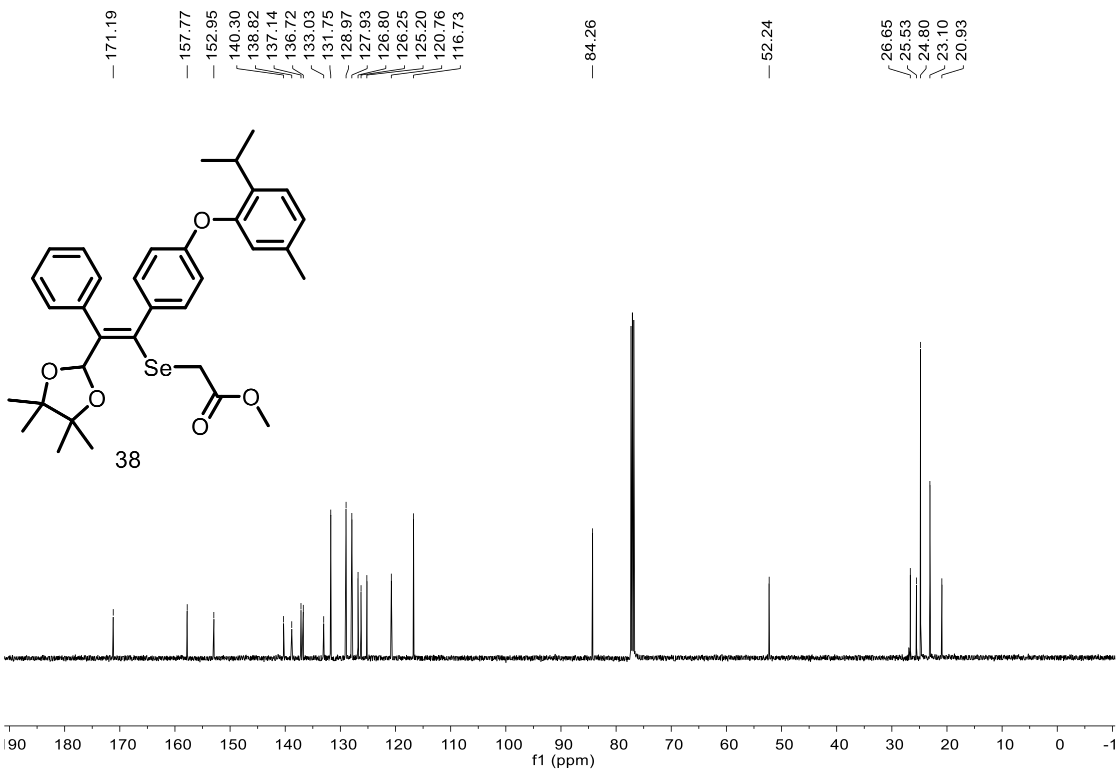

500 MHz, 298 K, $CDCl_3$ as solvent

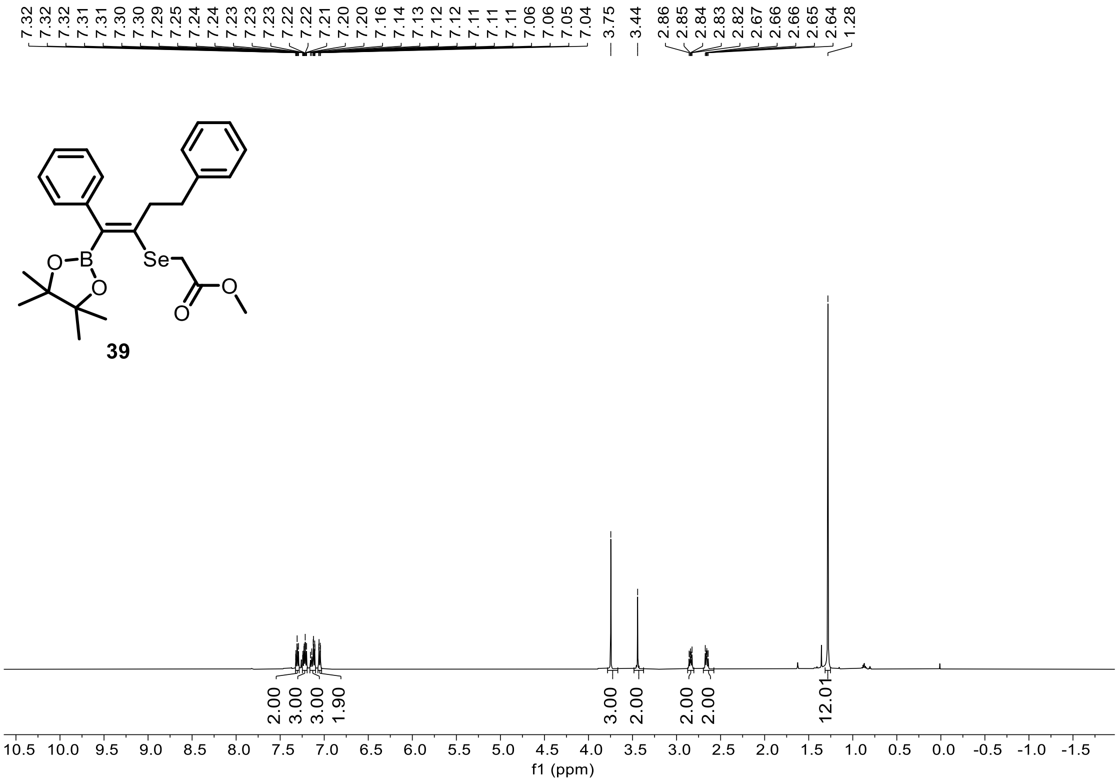

126 MHz, 298 K, $CDCl_3$ as solvent

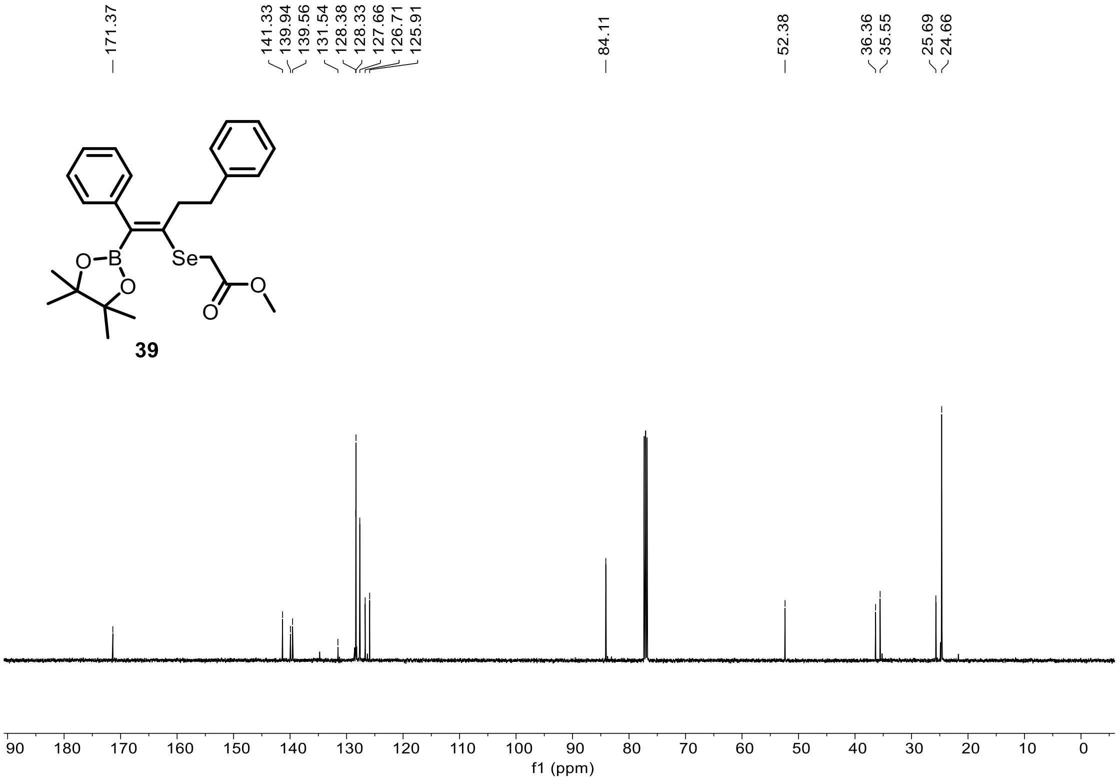

500 MHz, 298 K, $CDCl_3$ as solvent

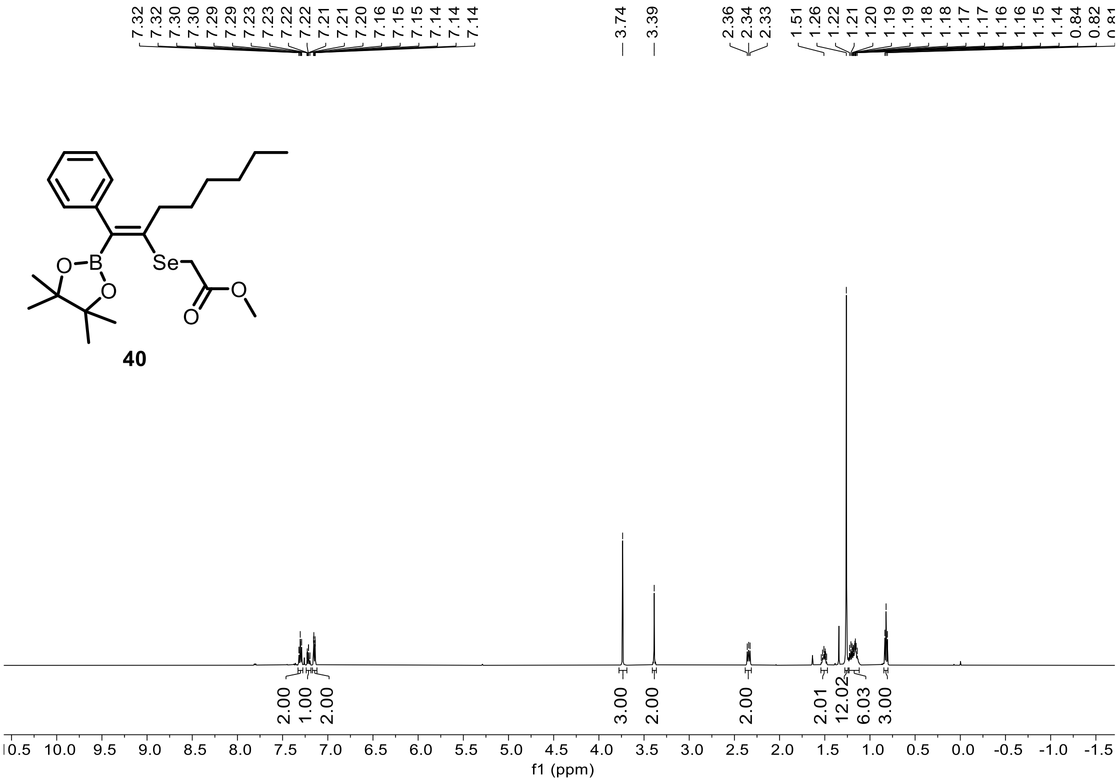

126 MHz, 298 K, $CDCl_3$ as solvent

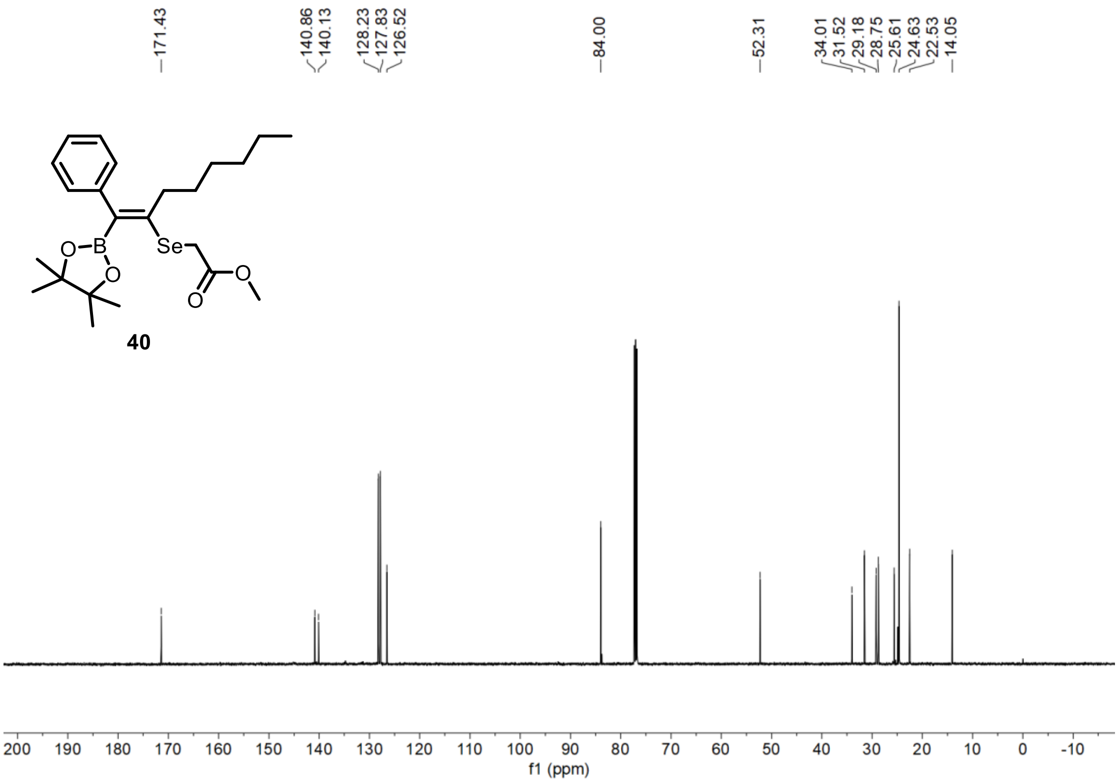

500 MHz, 298 K, $CDCl_3$ as solvent

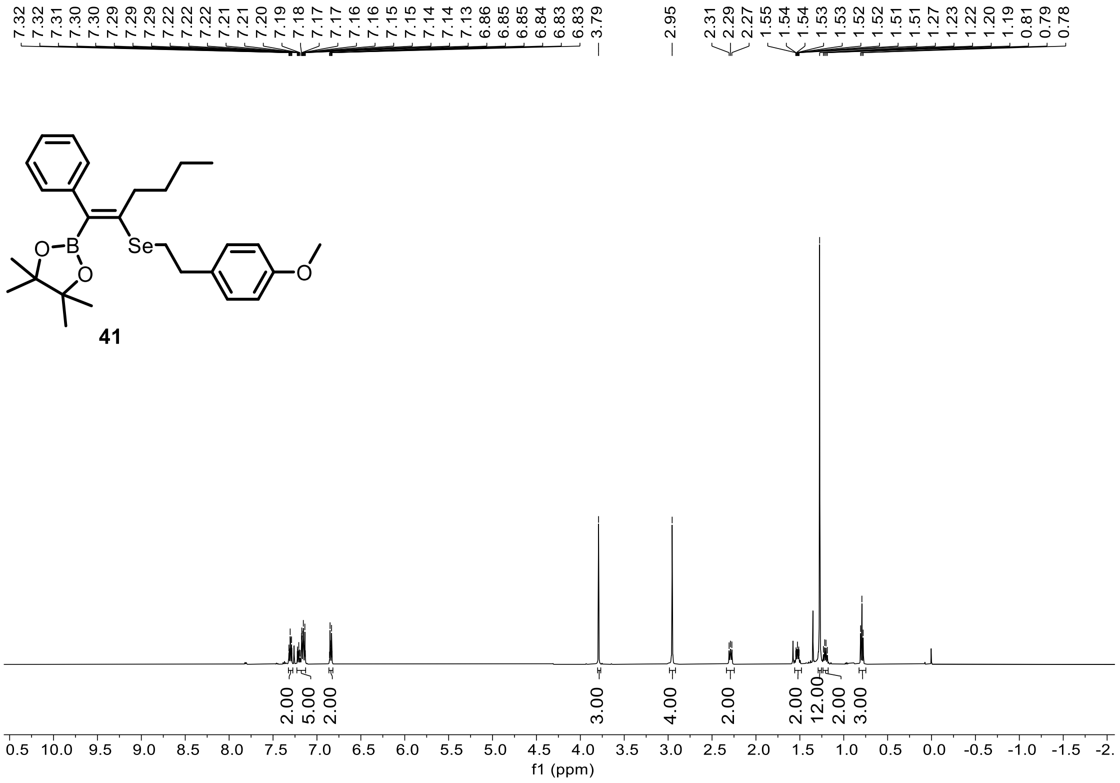

41

126 MHz, 298 K, $CDCl_3$ as solvent

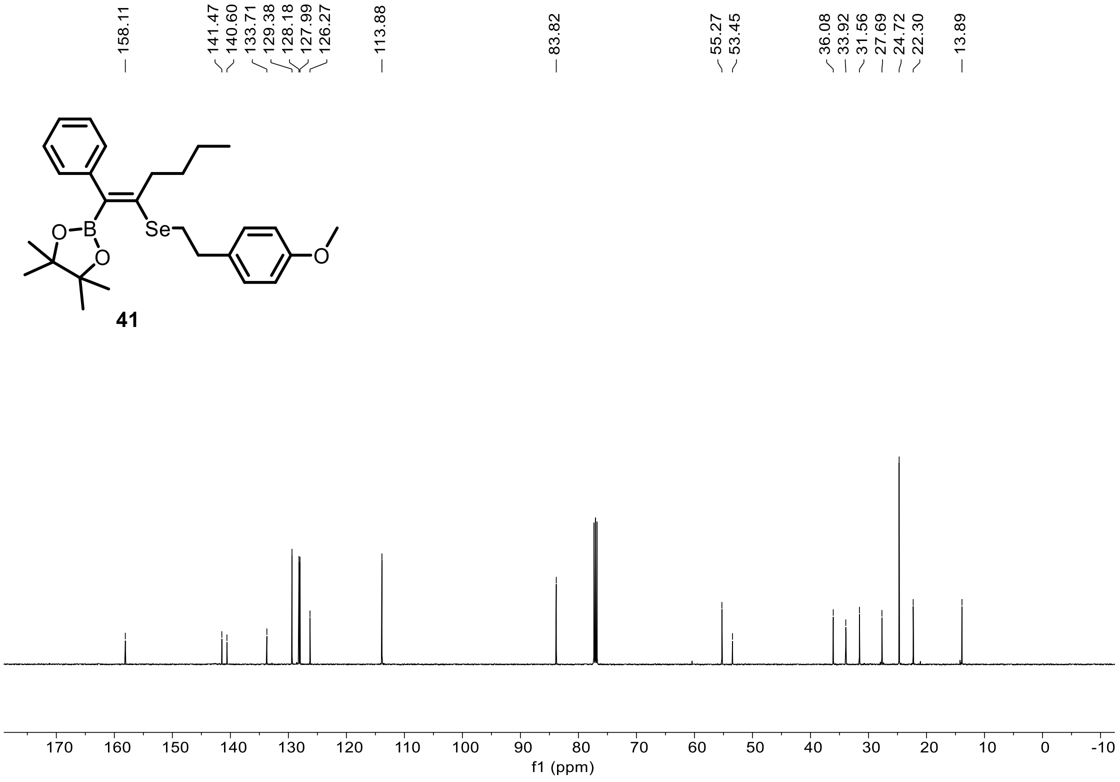

41

500 MHz, 298 K, $CDCl_3$ as solvent

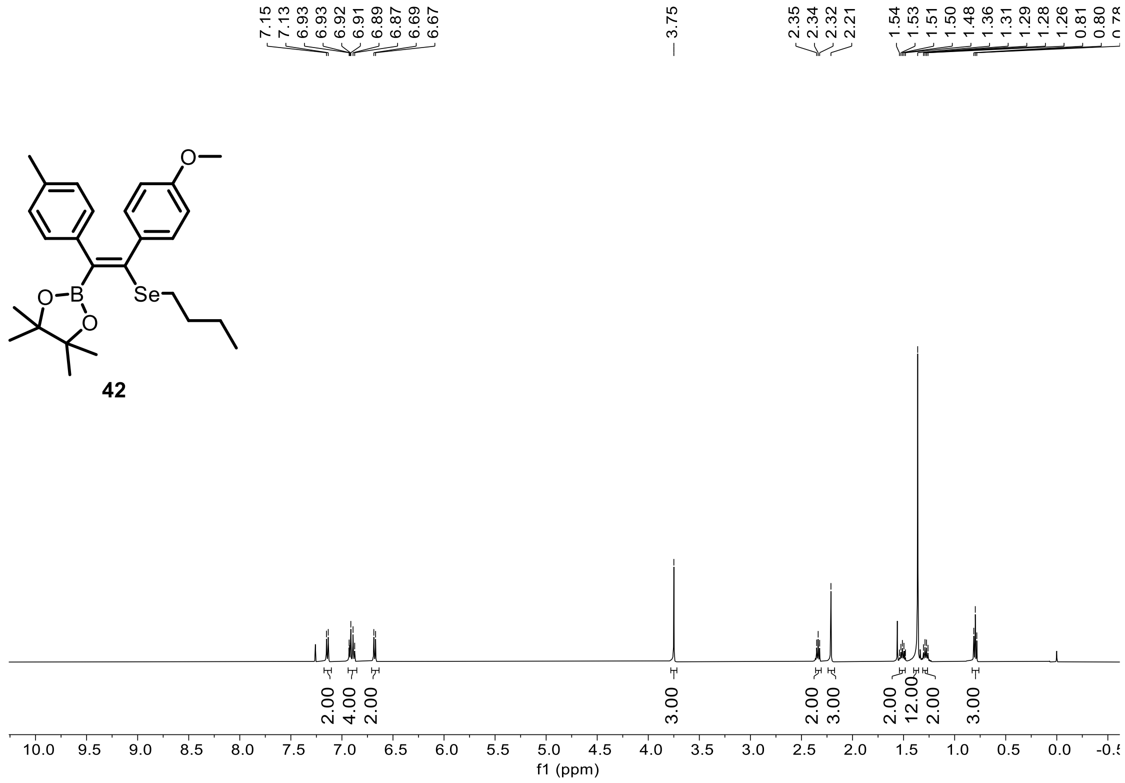

126 MHz, 298 K, $CDCl_3$ as solvent

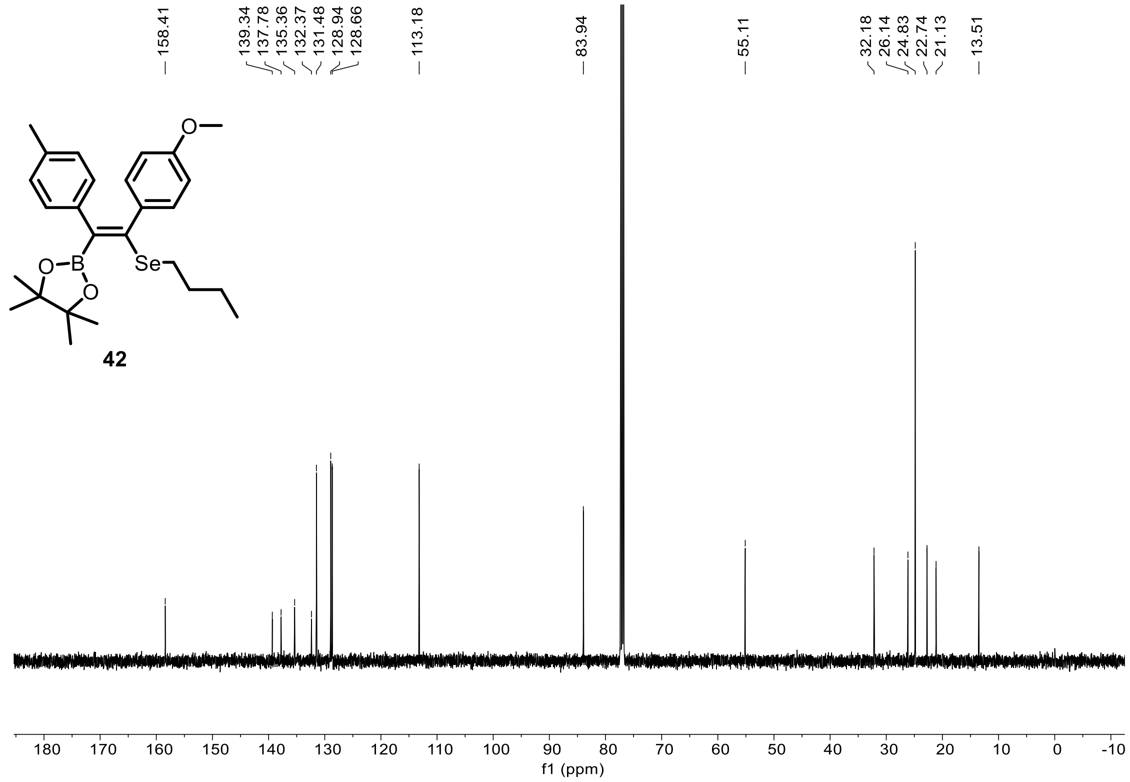

500 MHz, 298 K, $CDCl_3$ as solvent

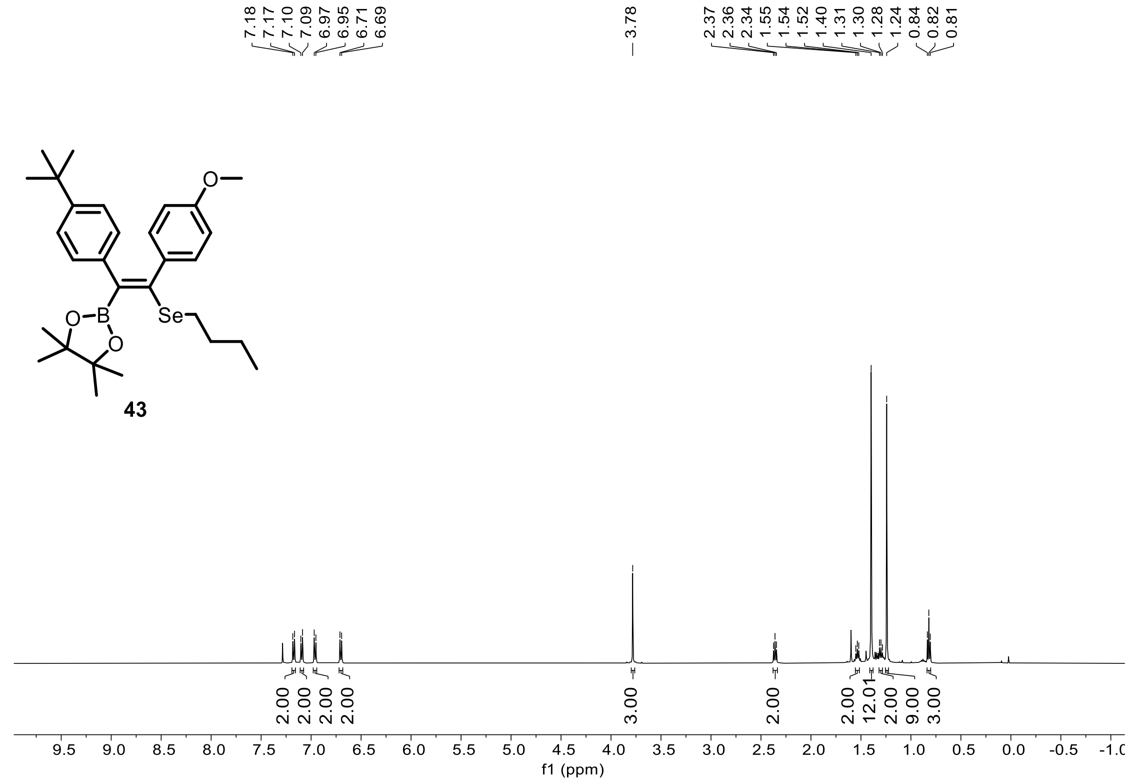

126 MHz, 298 K, $CDCl_3$ as solvent

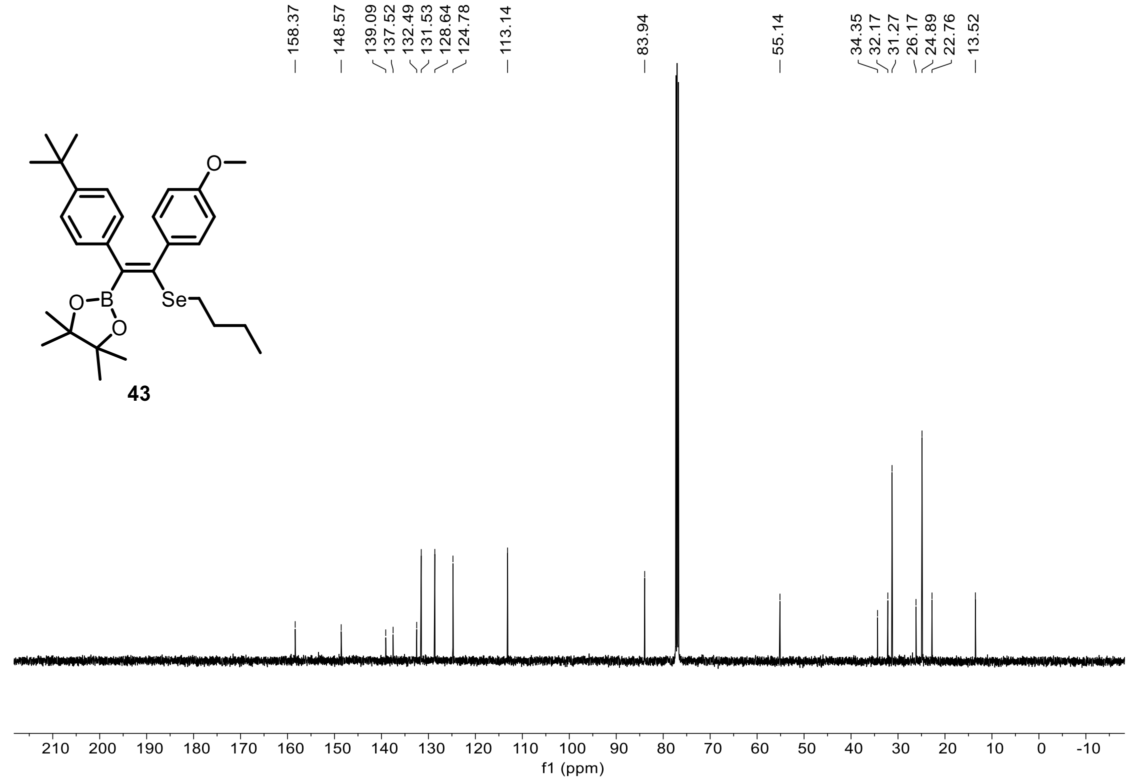

500 MHz, 298 K, $CDCl_3$ as solvent

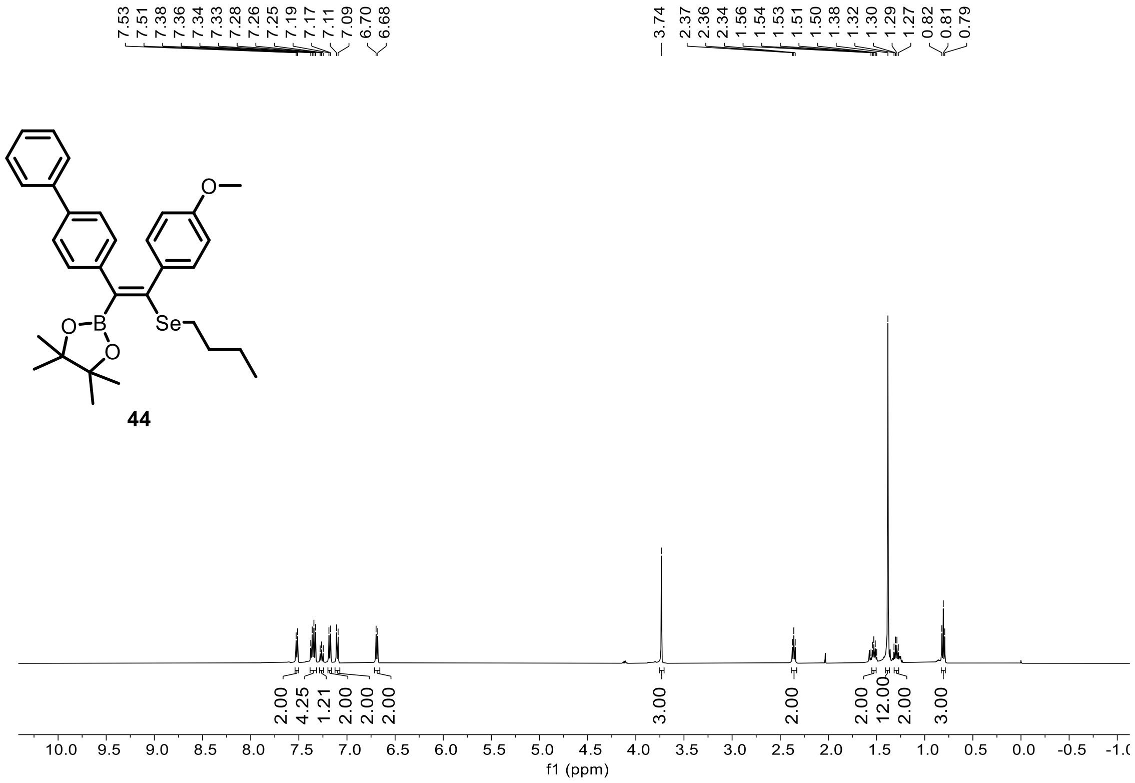

126 MHz, 298 K, $CDCl_3$ as solvent

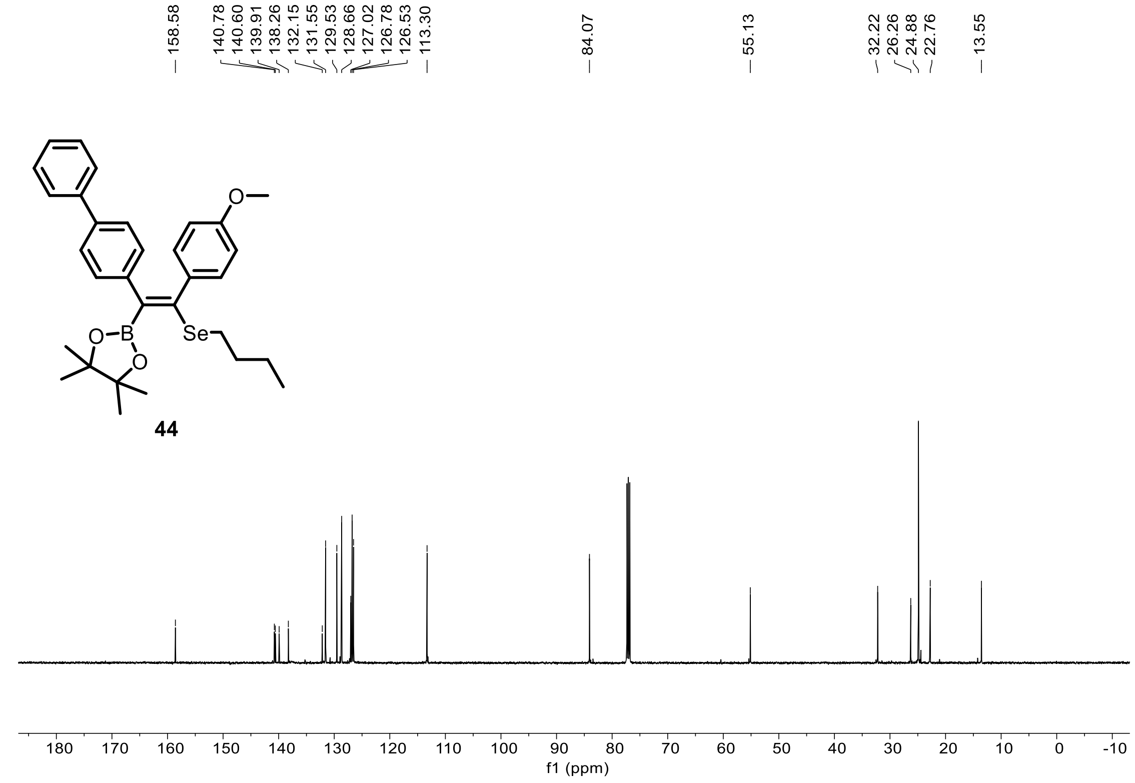

500 MHz, 298 K, $CDCl_3$ as solvent

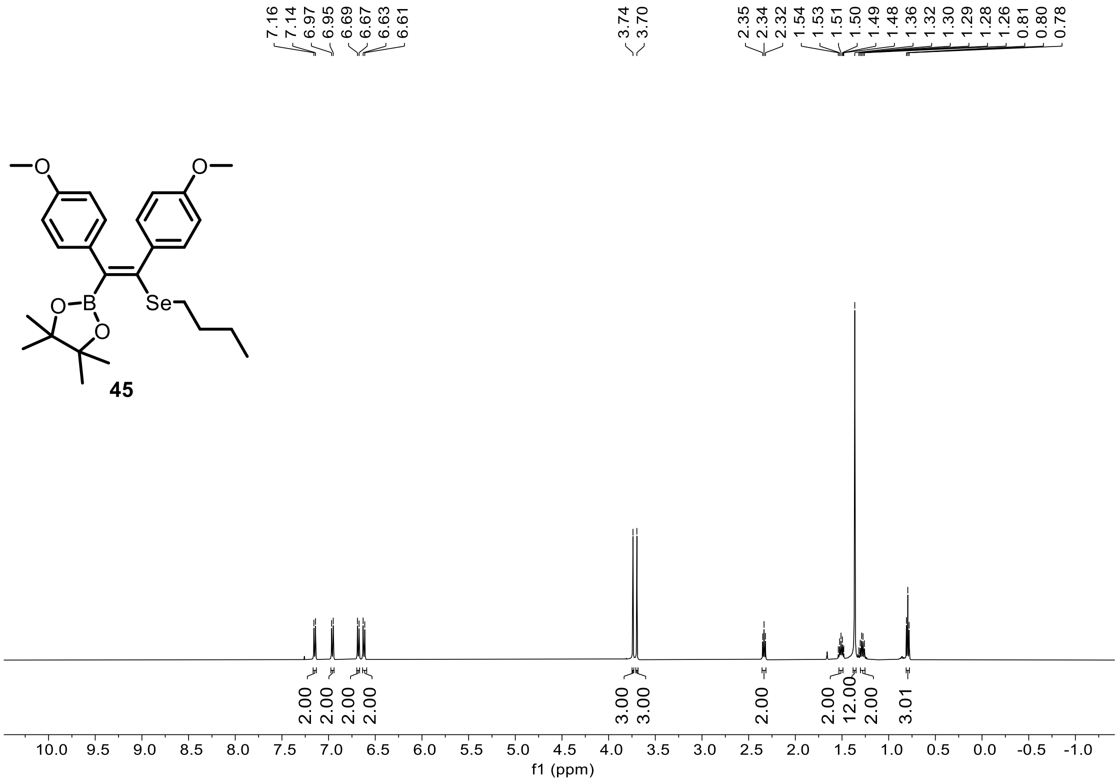

126 MHz, 298 K, $CDCl_3$ as solvent

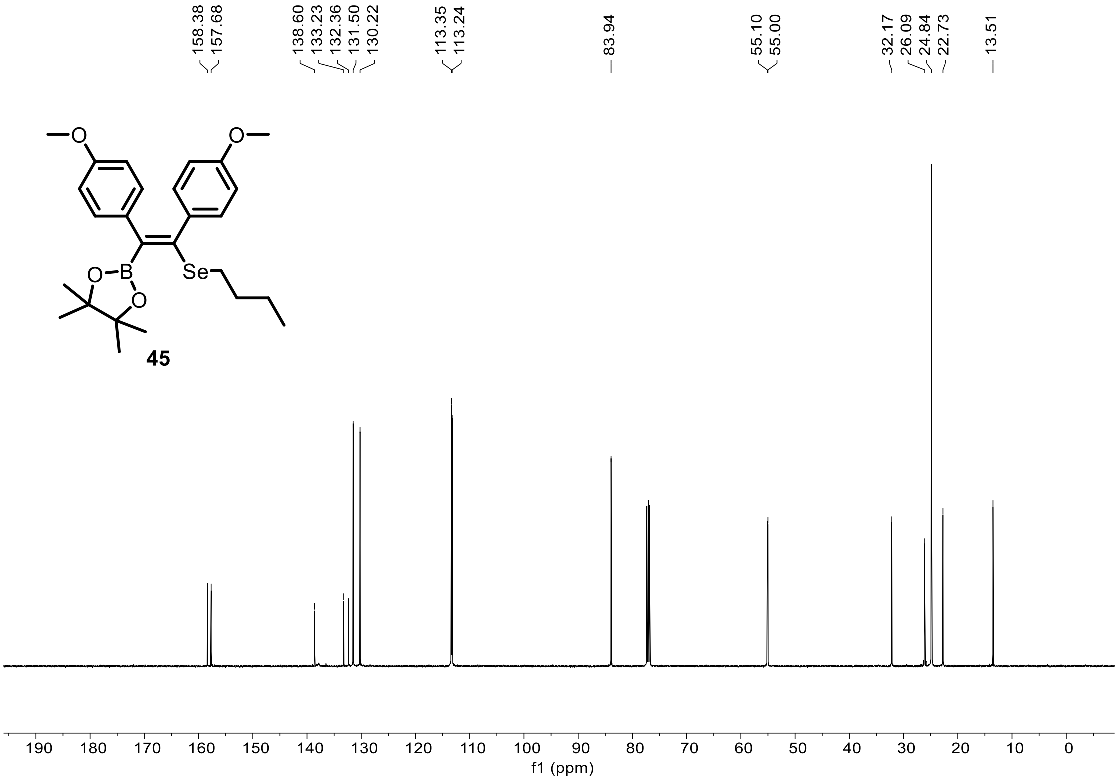

500 MHz, 298 K, $CDCl_3$ as solvent

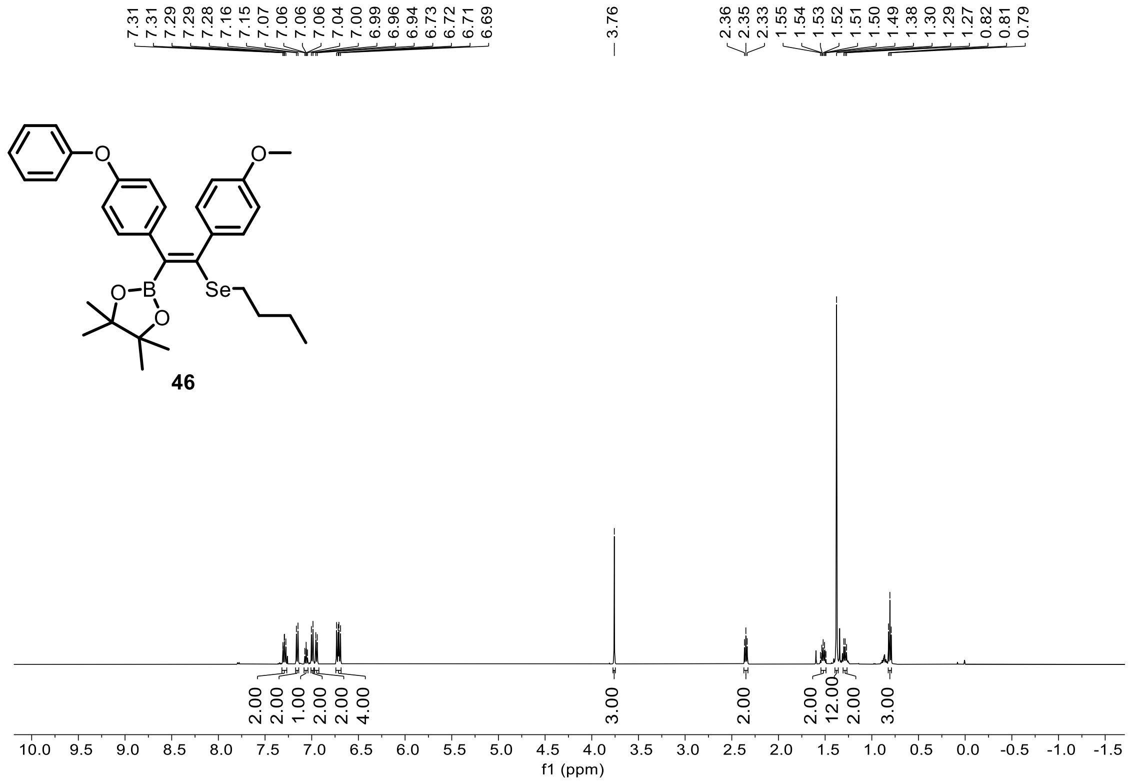

126 MHz, 298 K, $CDCl_3$ as solvent

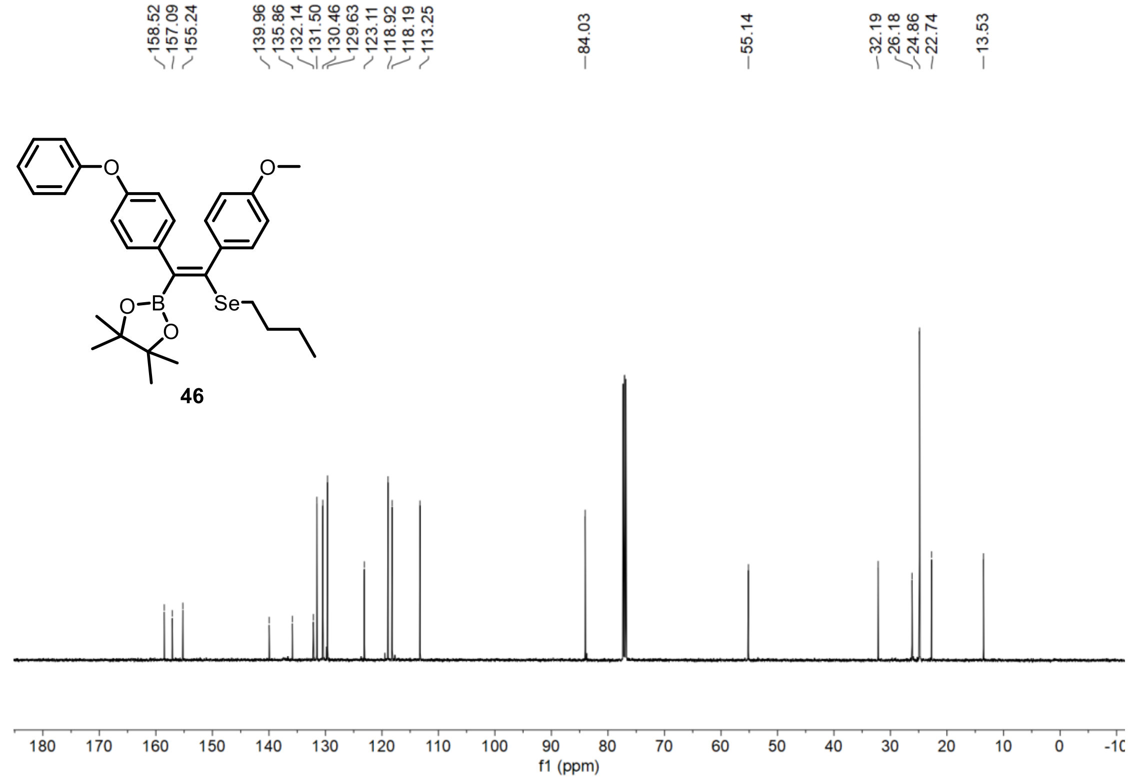

500 MHz, 298 K, $CDCl_3$ as solvent

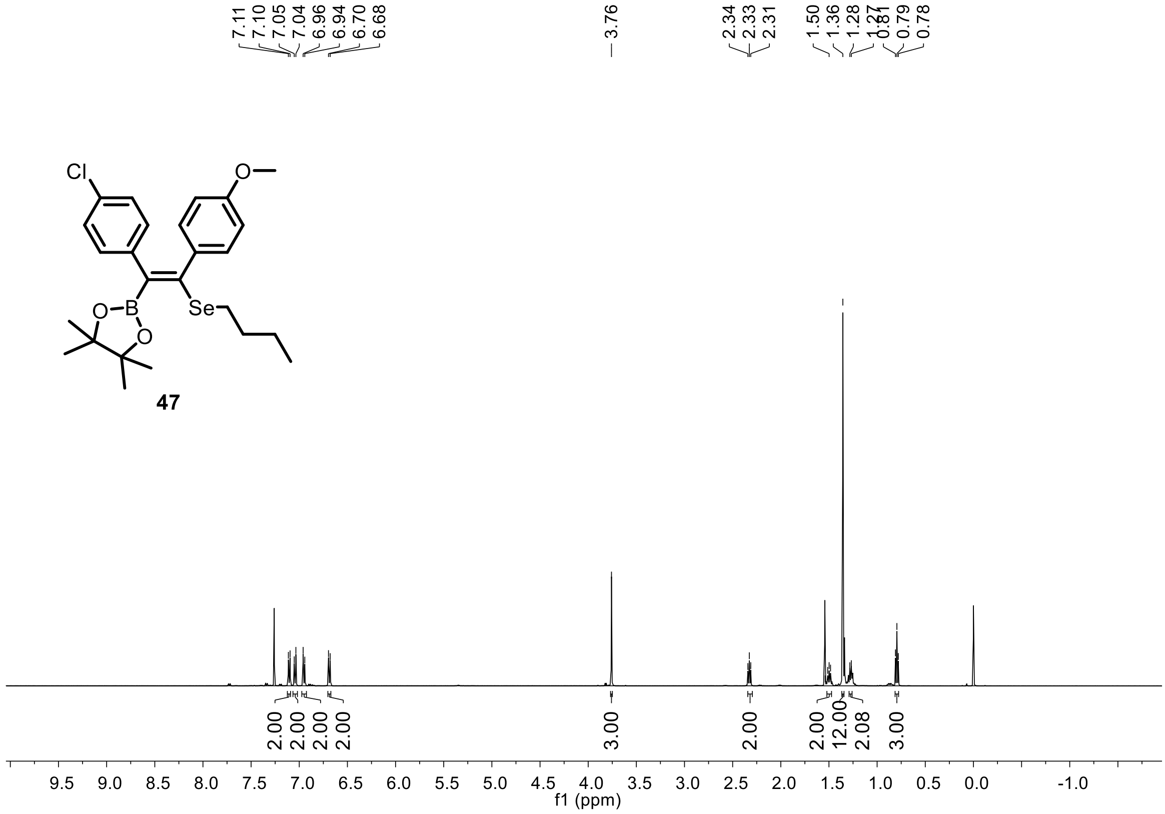

126 MHz, 298 K, $CDCl_3$ as solvent

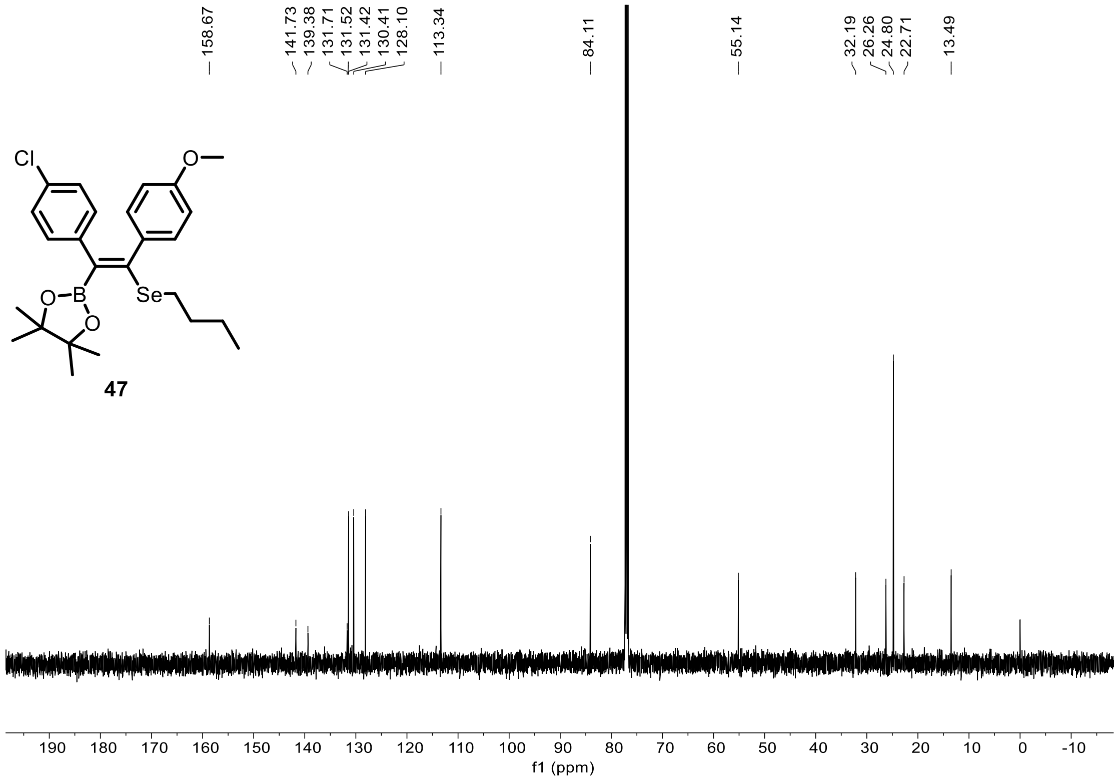

500 MHz, 298 K, $CDCl_3$ as solvent

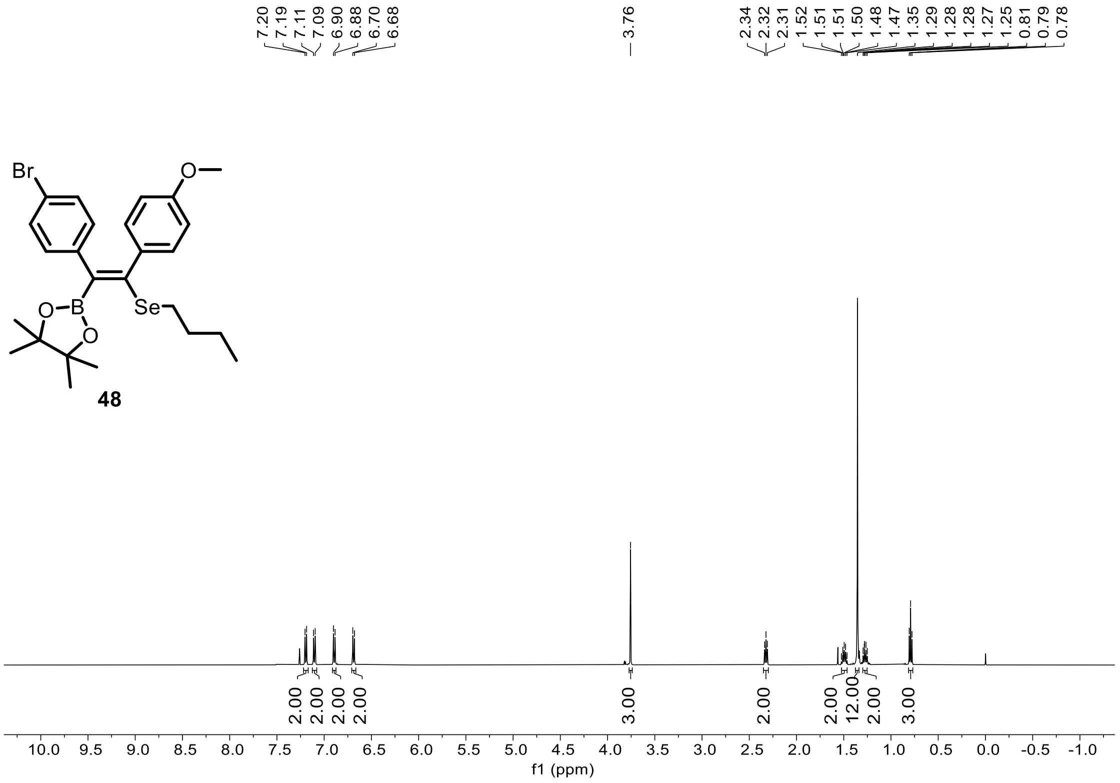

126 MHz, 298 K, $CDCl_3$ as solvent

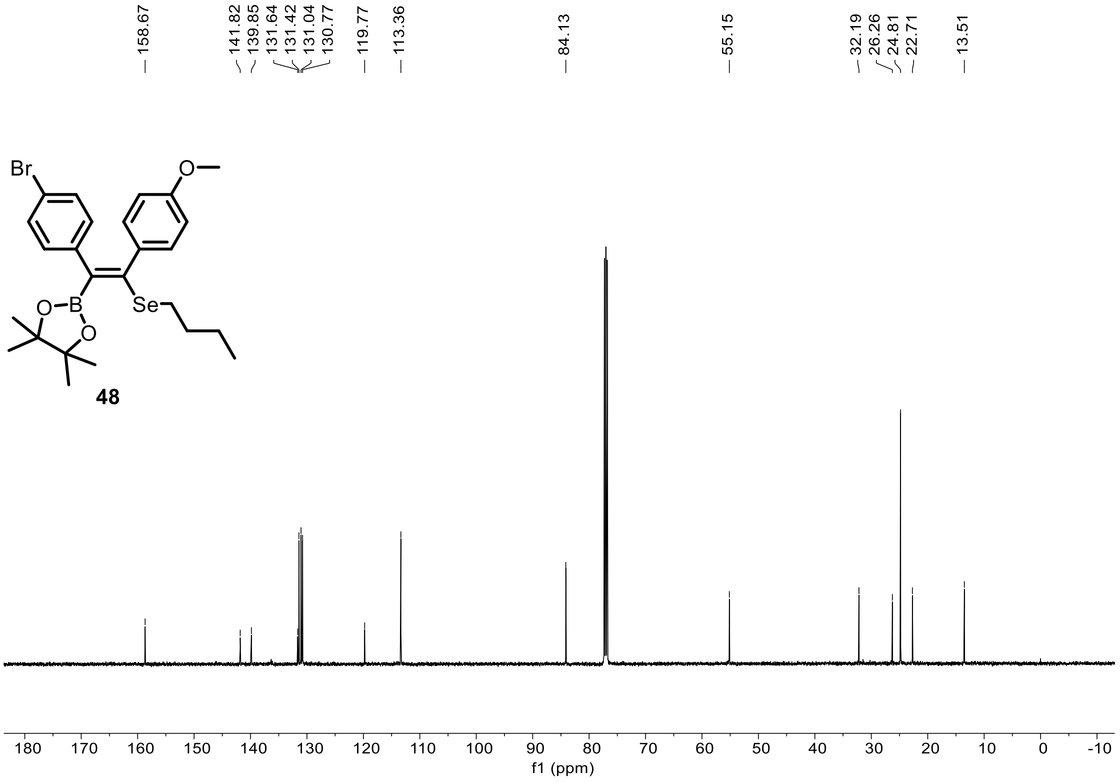

500 MHz, 298 K, $CDCl_3$ as solvent

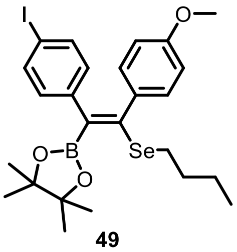

49

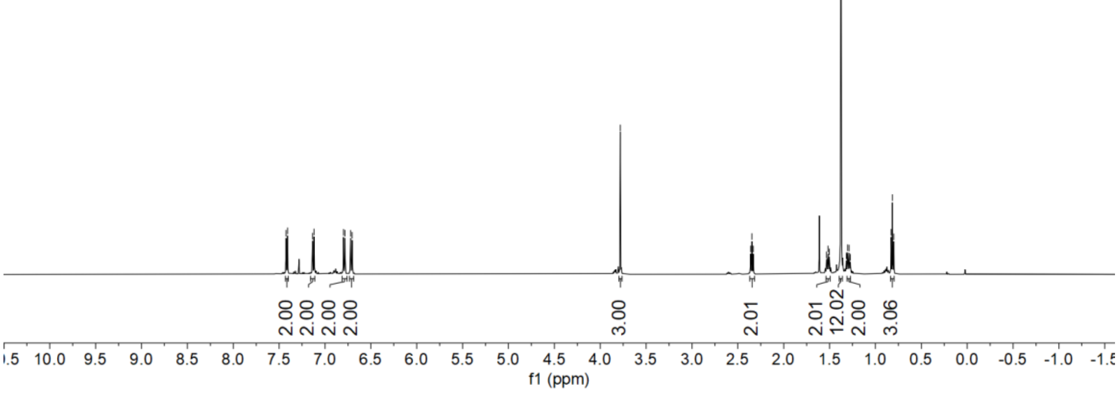

126 MHz, 298 K, $CDCl_3$ as solvent

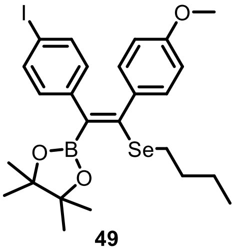

49

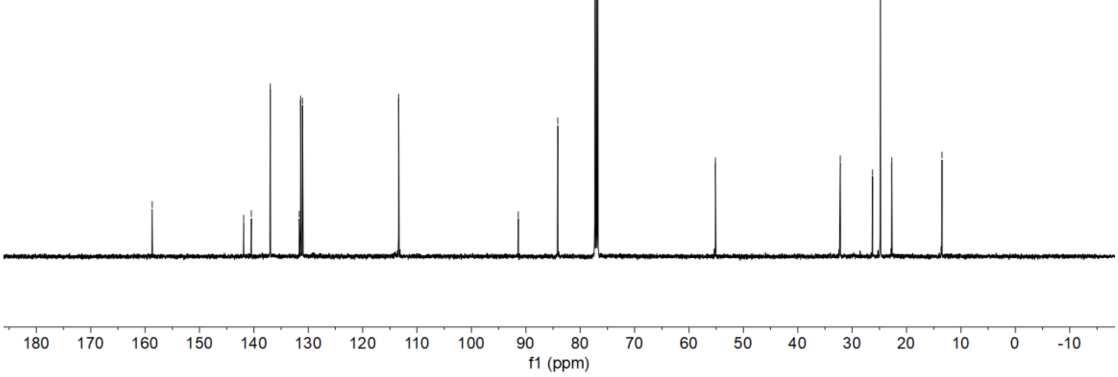

500 MHz, 298 K, CD_2Cl_2 as solvent

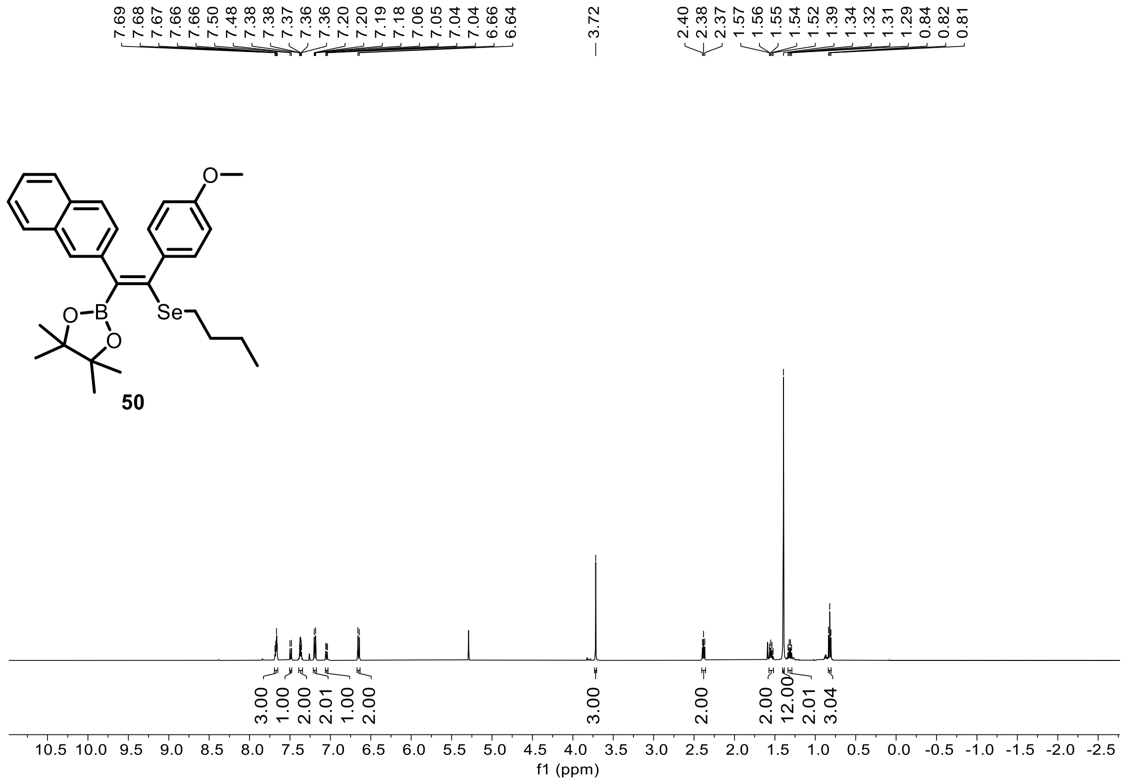

126 MHz, 298 K, $CDCl_3$ as solvent

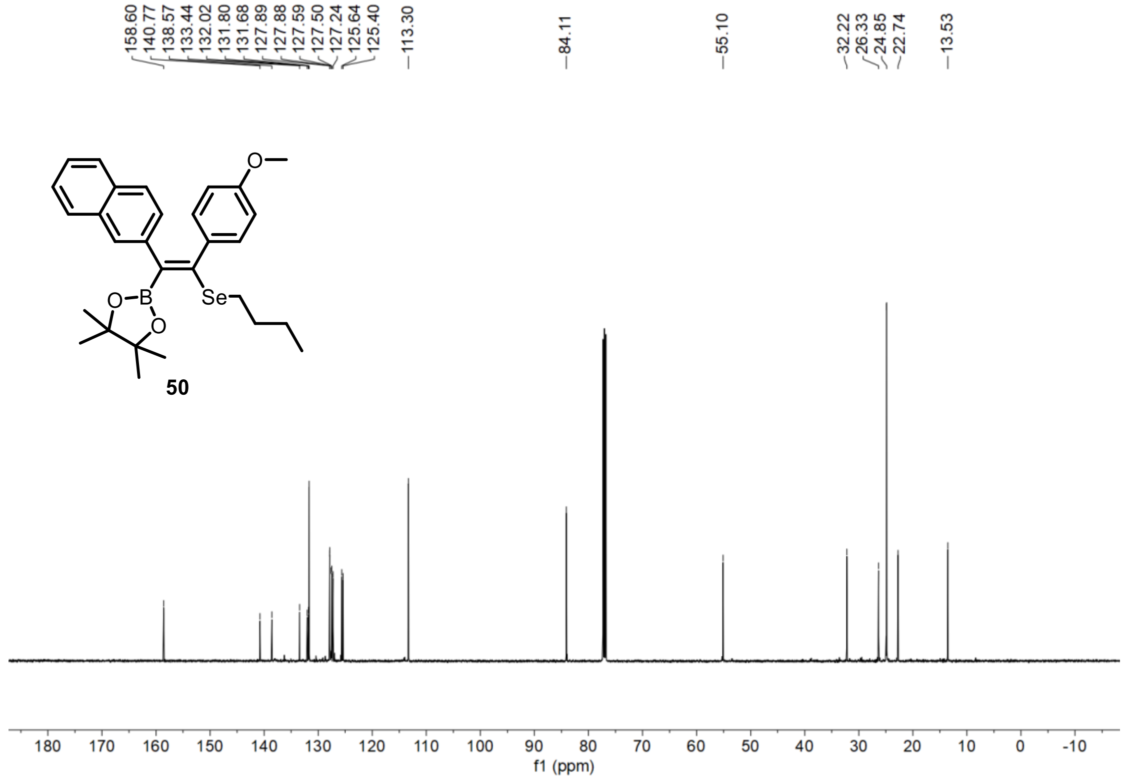

500 MHz, 298 K, CDCl3 as solvent

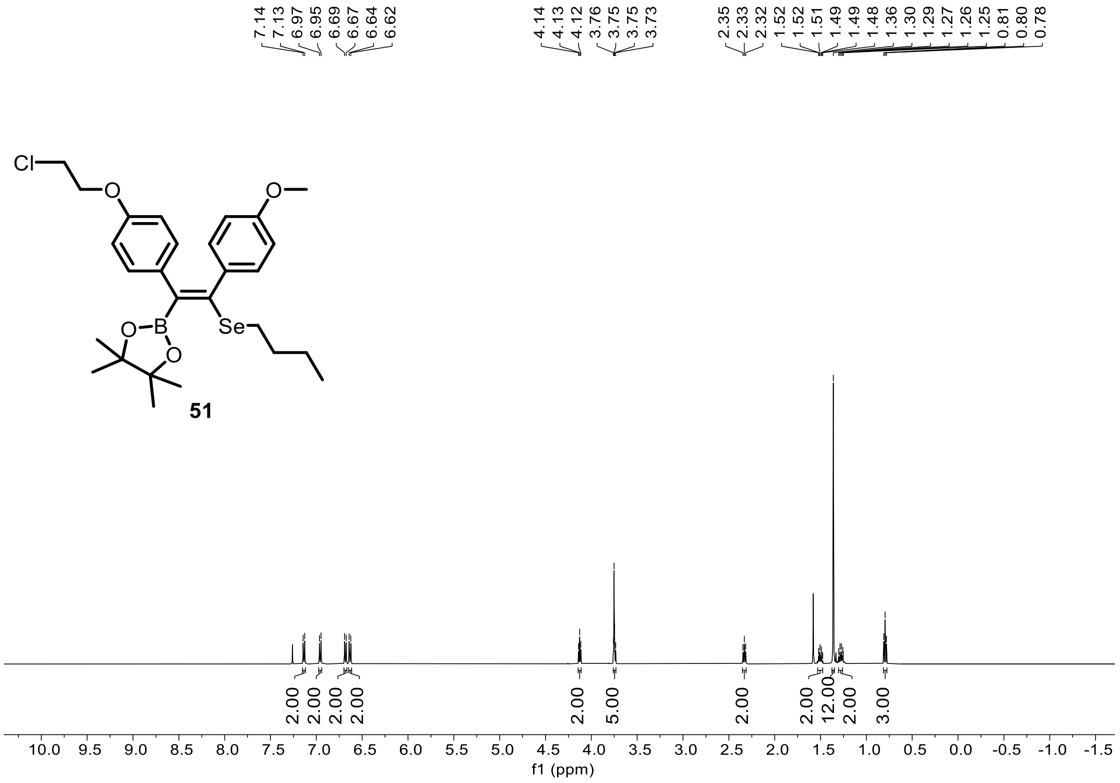

126 MHz, 298 K, CDCl3 as solvent

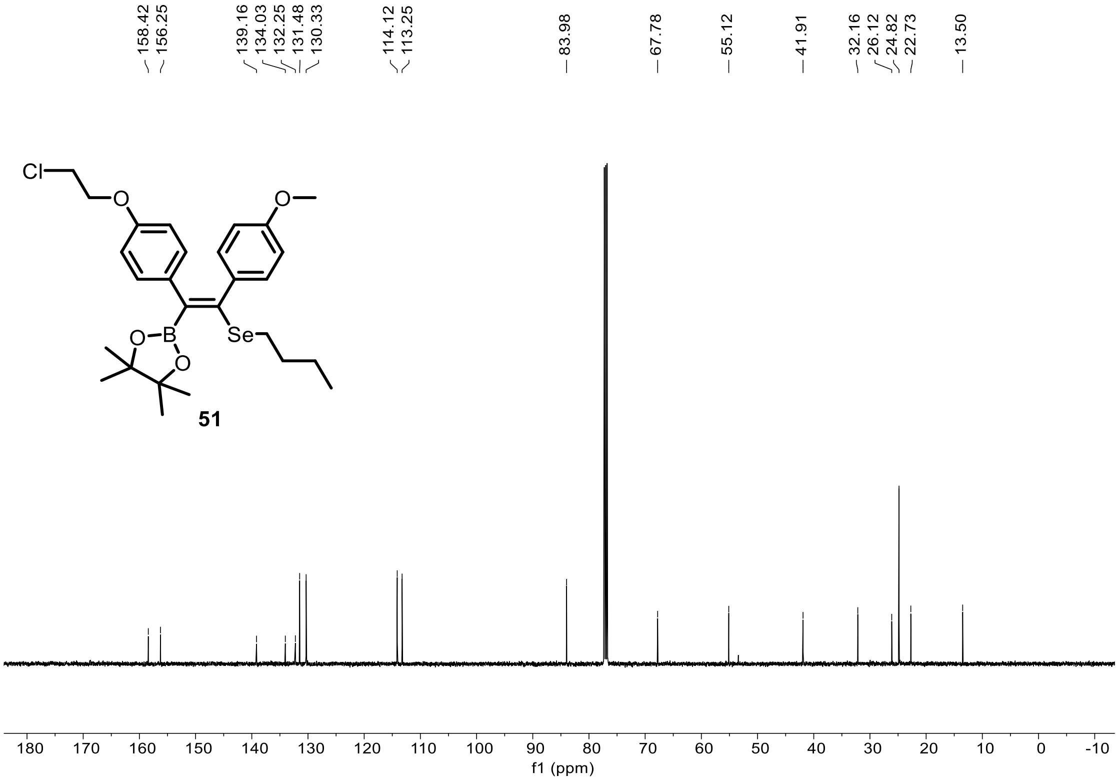

500 MHz, 298 K, $CDCl_3$ as solvent

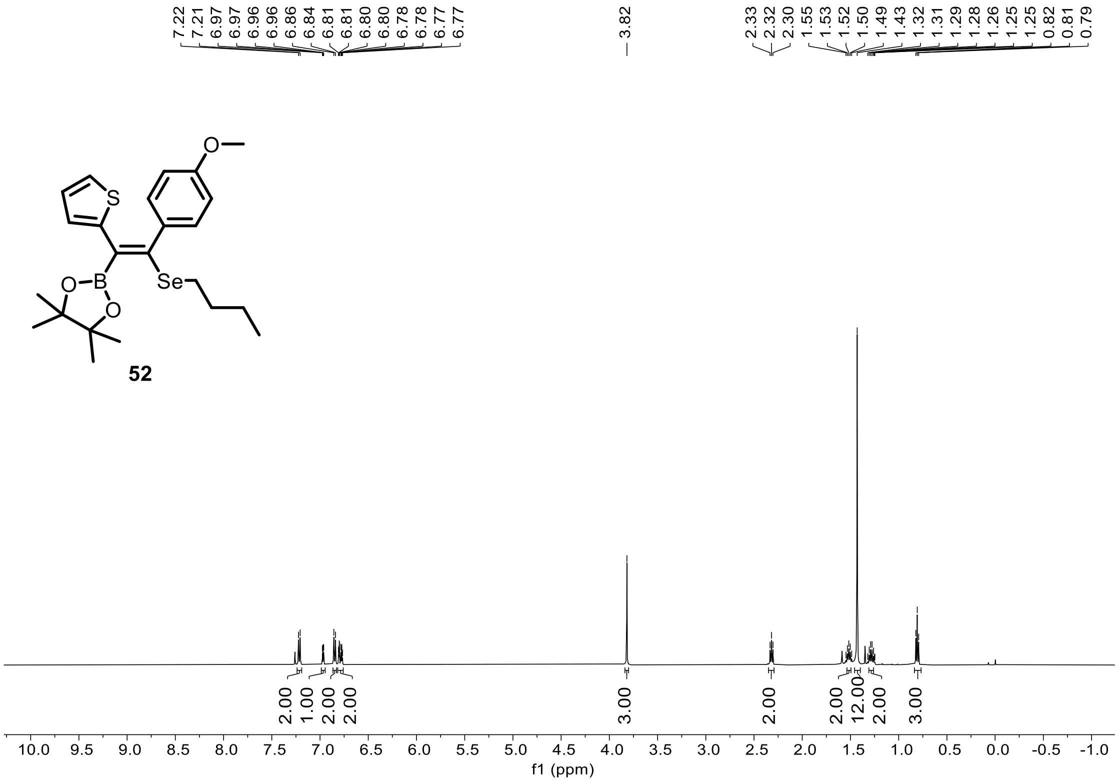

126 MHz, 298 K, $CDCl_3$ as solvent

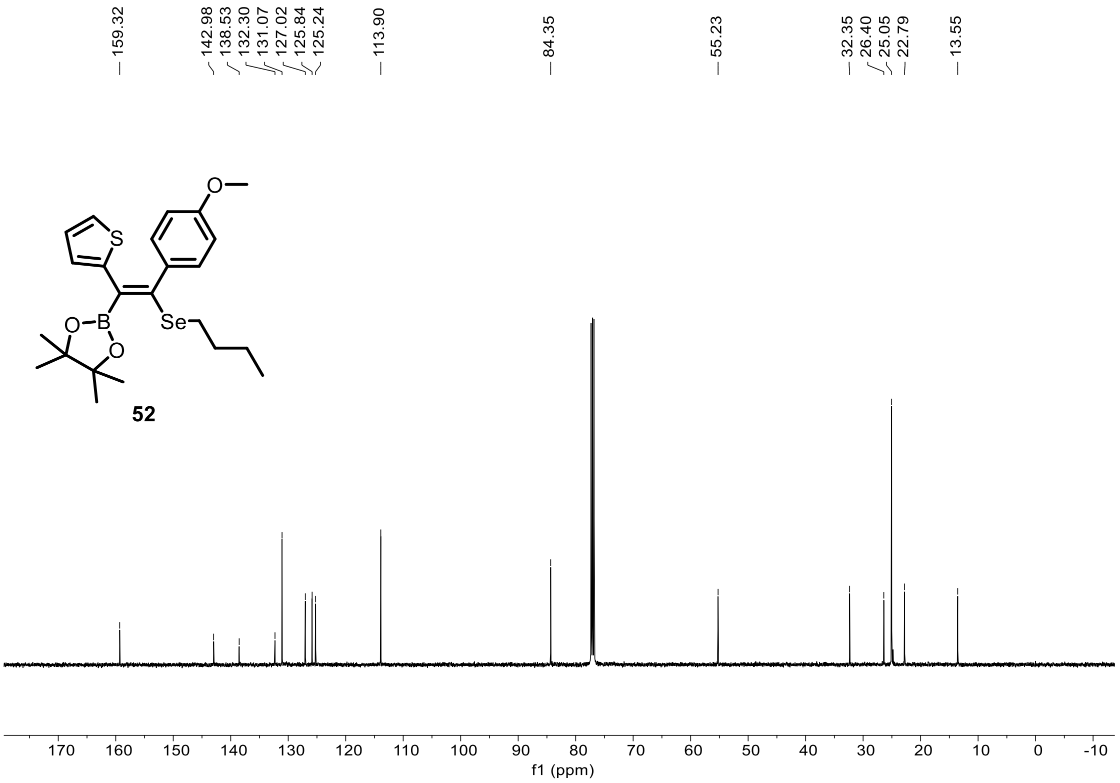

500 MHz, 298 K, $CDCl_3$ as solvent

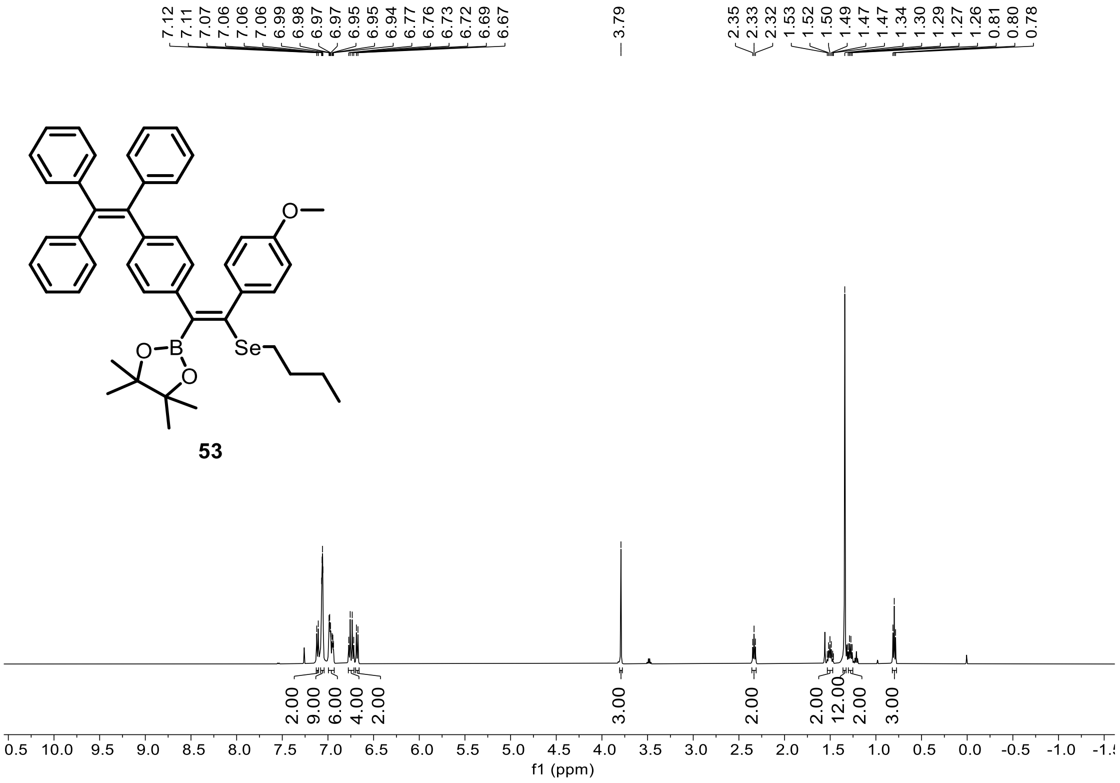

126 MHz, 298 K, $CDCl_3$ as solvent

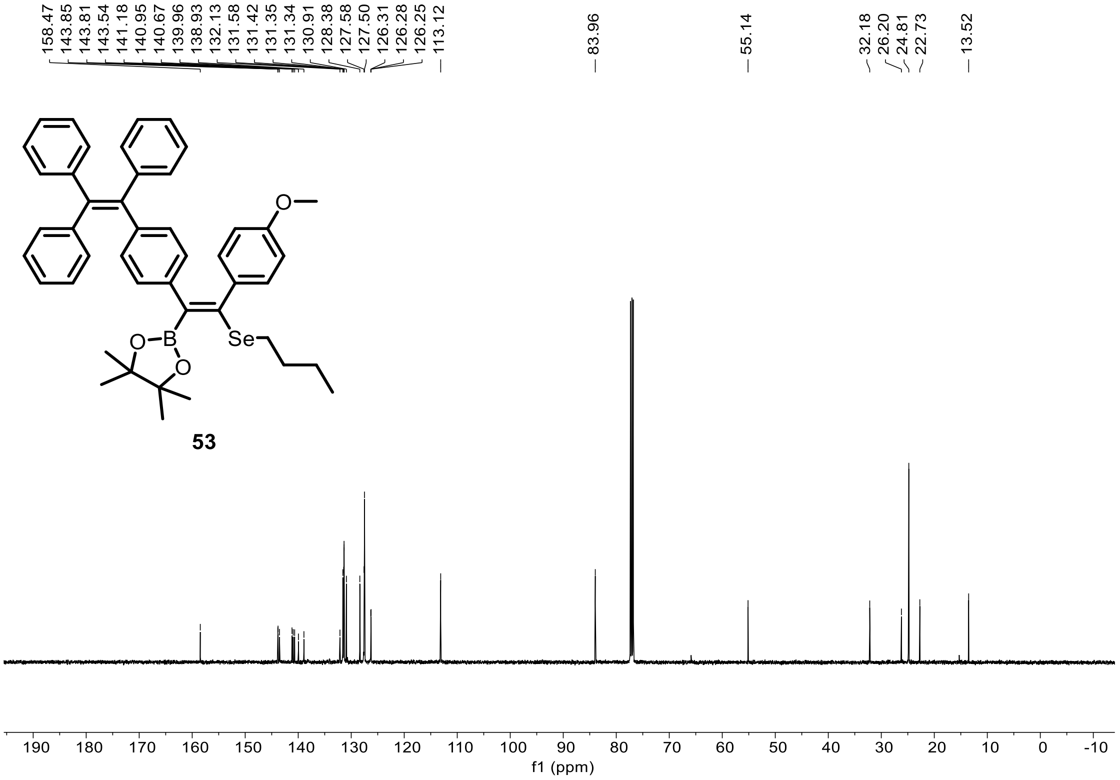

500 MHz, 298 K, $CDCl_3$ as solvent

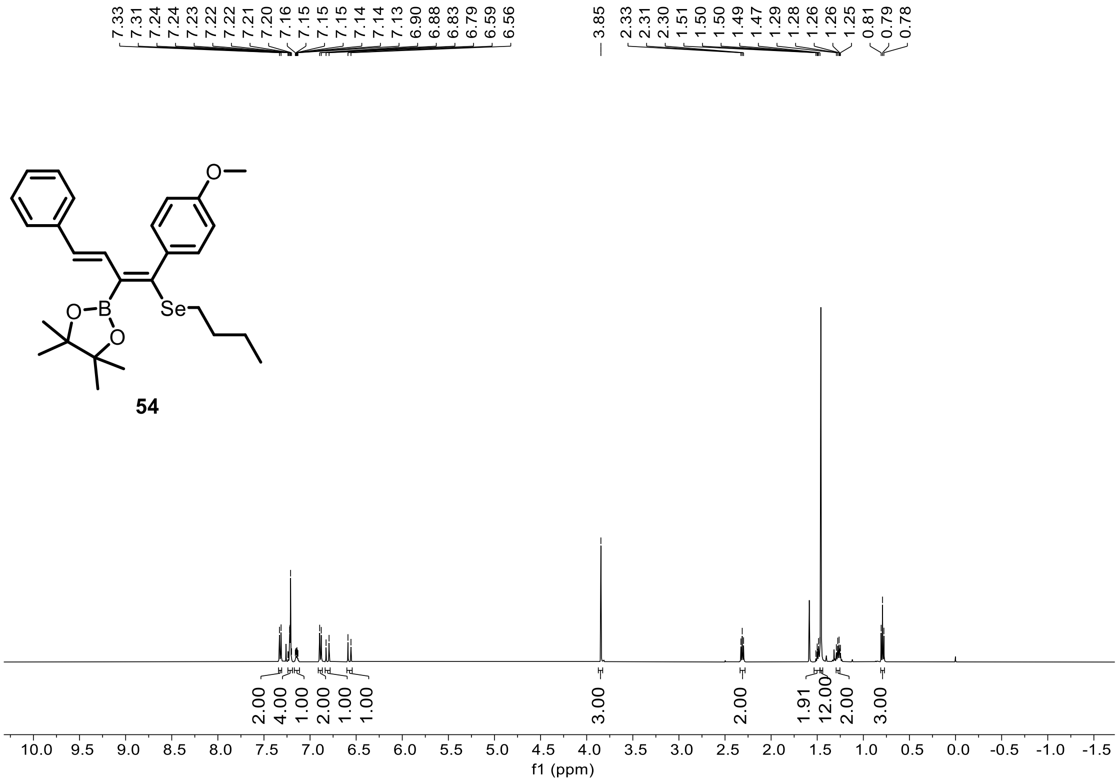

126 MHz, 298 K, $CDCl_3$ as solvent

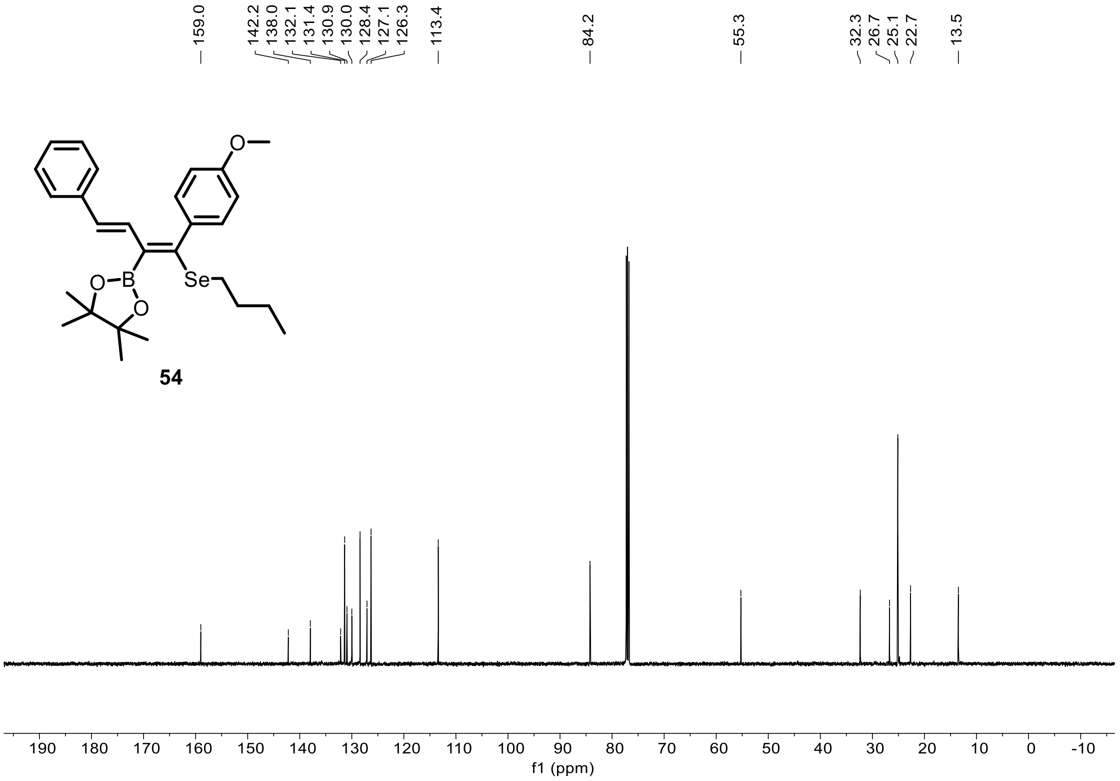

500 MHz, 298 K, $CDCl_3$ as solvent

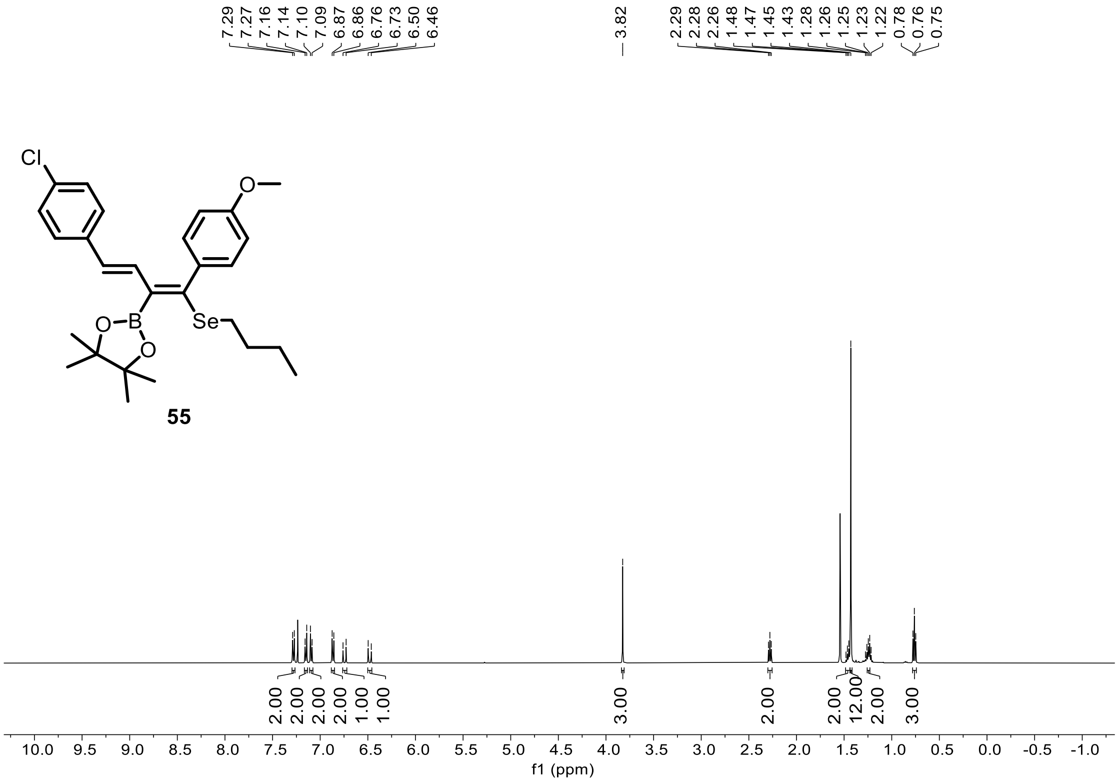

126 MHz, 298 K, $CDCl_3$ as solvent

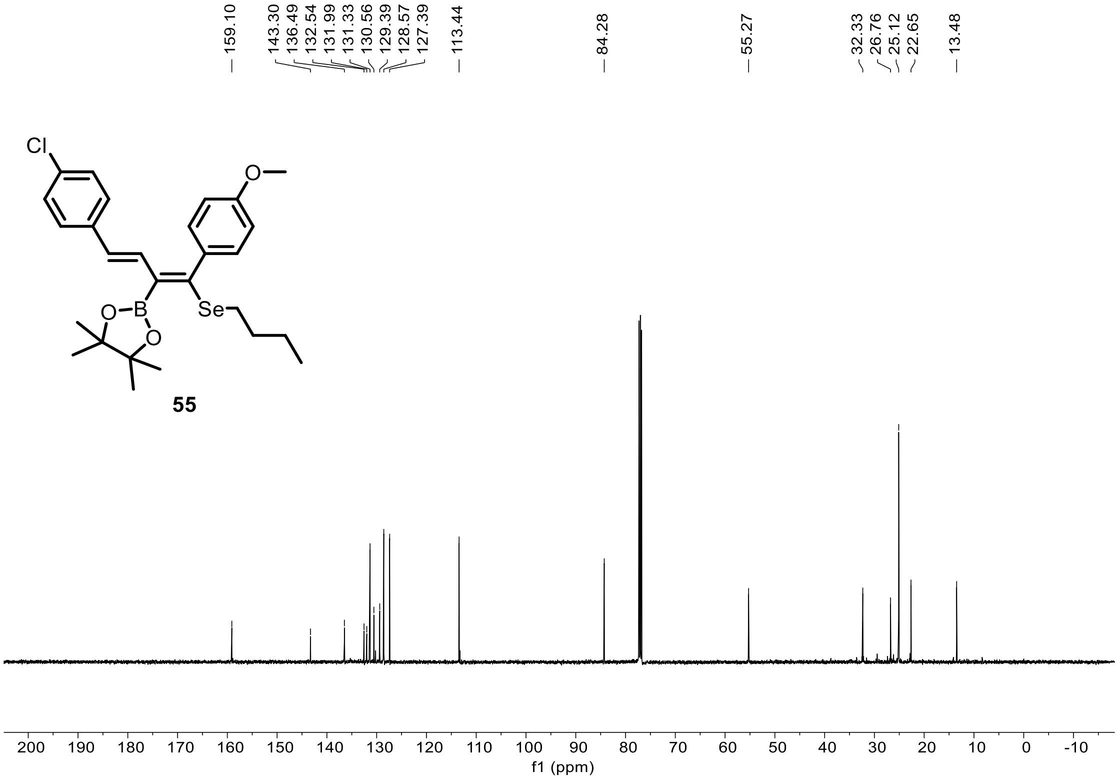

500 MHz, 298 K, $CDCl_3$ as solvent

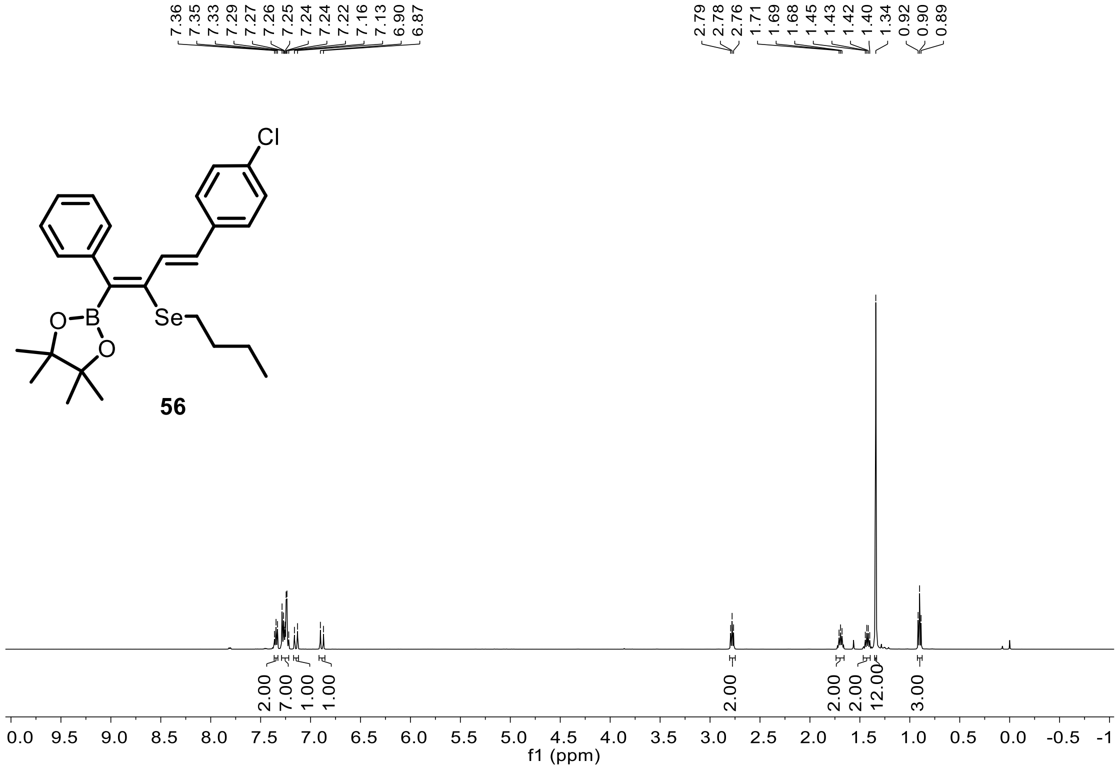

126 MHz, 298 K, $CDCl_3$ as solvent

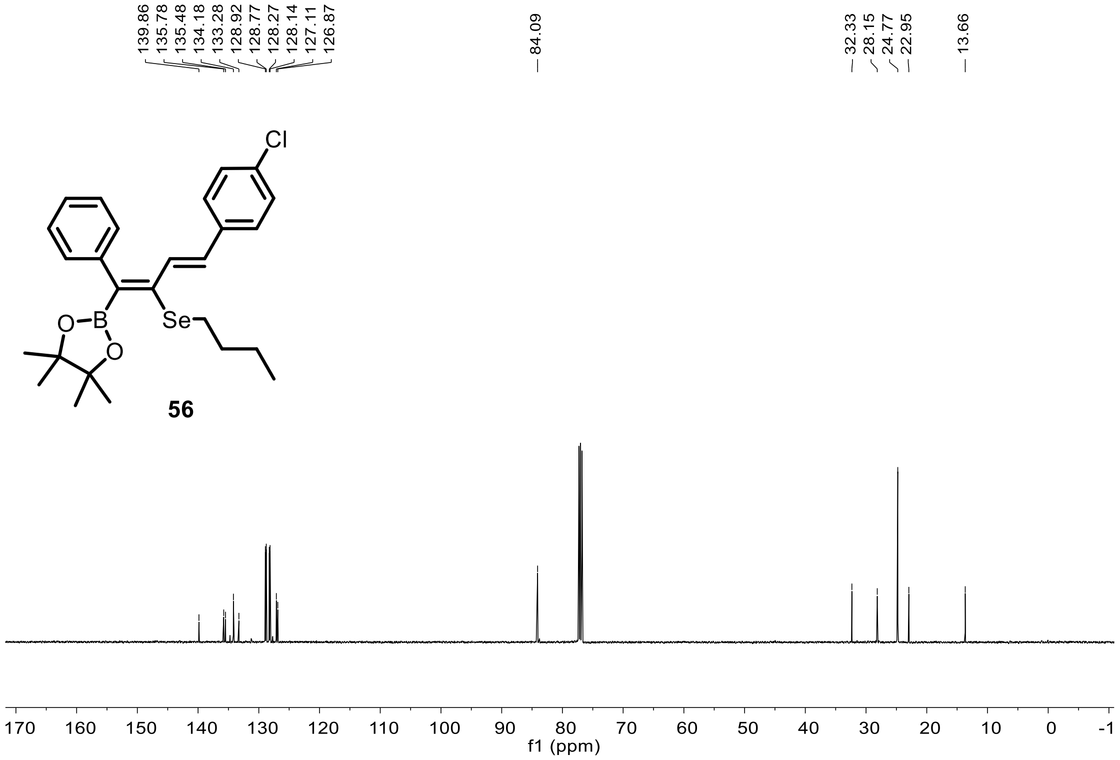

500 MHz, 298 K, $CDCl_3$ as solvent

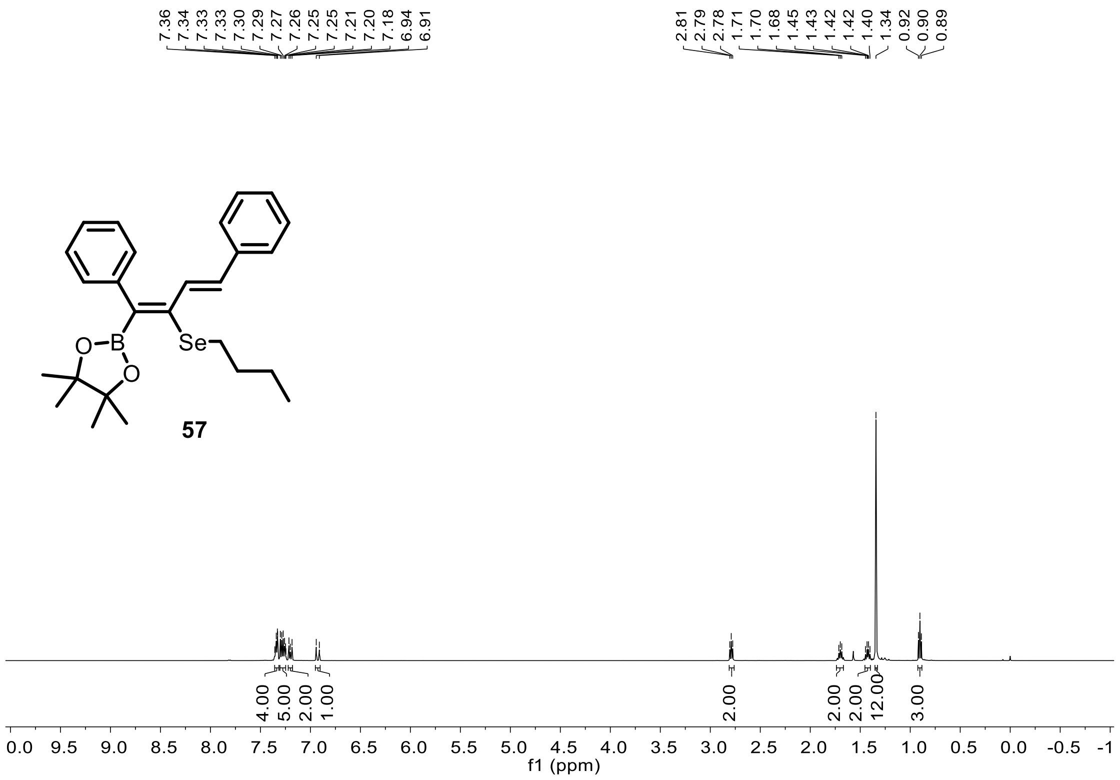

126 MHz, 298 K, $CDCl_3$ as solvent

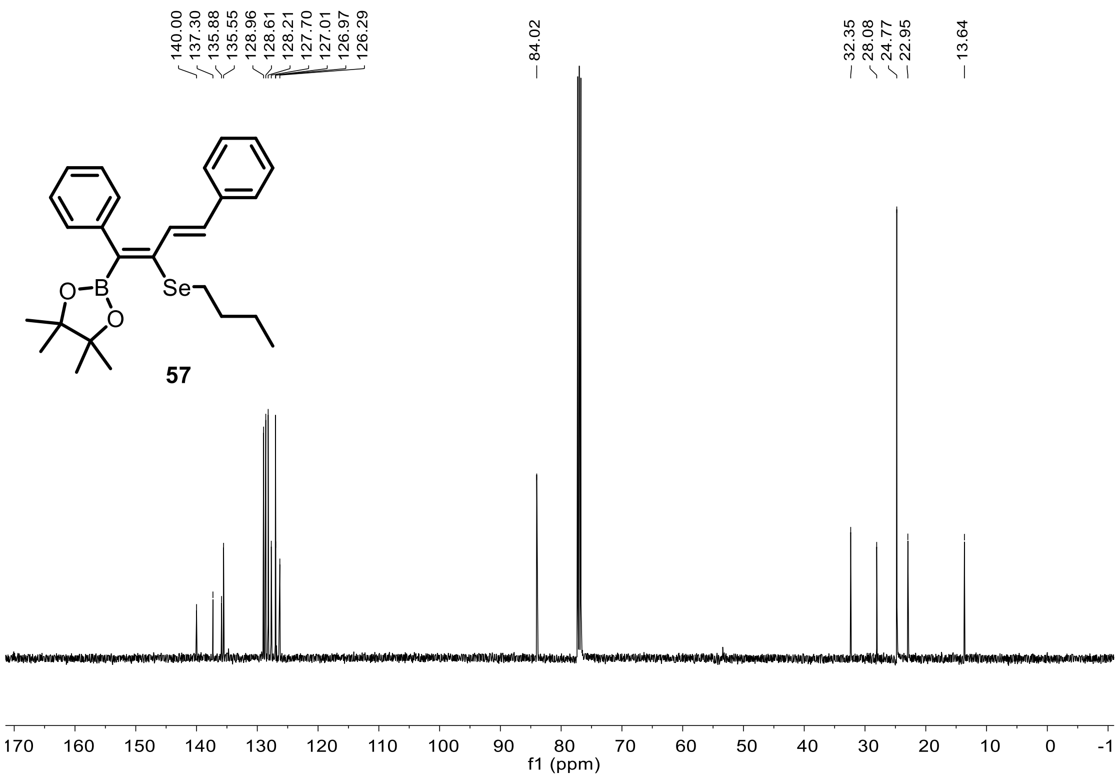

500 MHz, 298 K, $CDCl_3$ as solvent

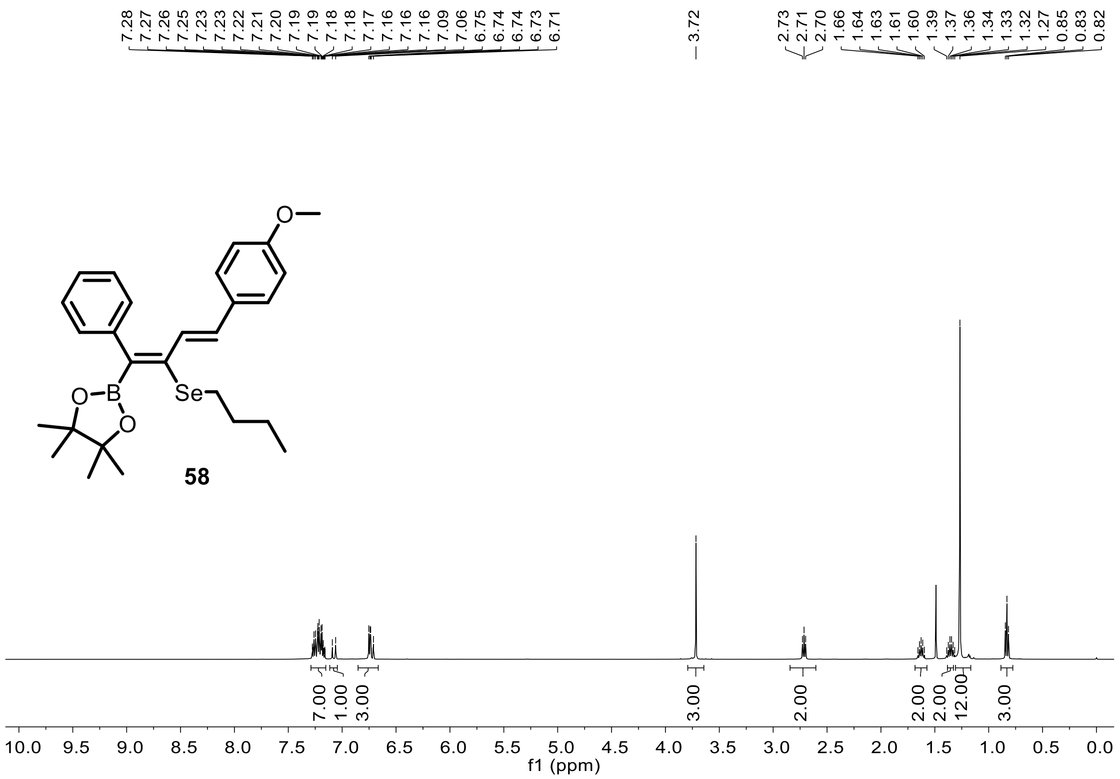

126 MHz, 298 K, $CDCl_3$ as solvent

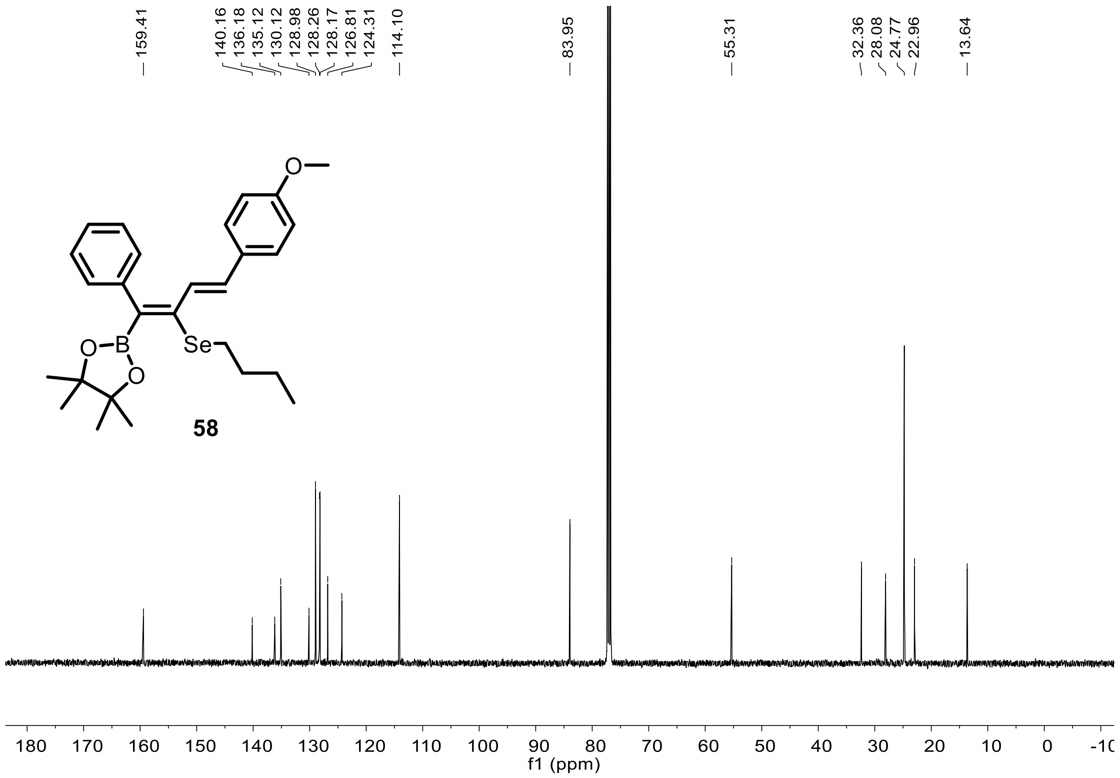

500 MHz, 298 K, CDCl₃ as solvent

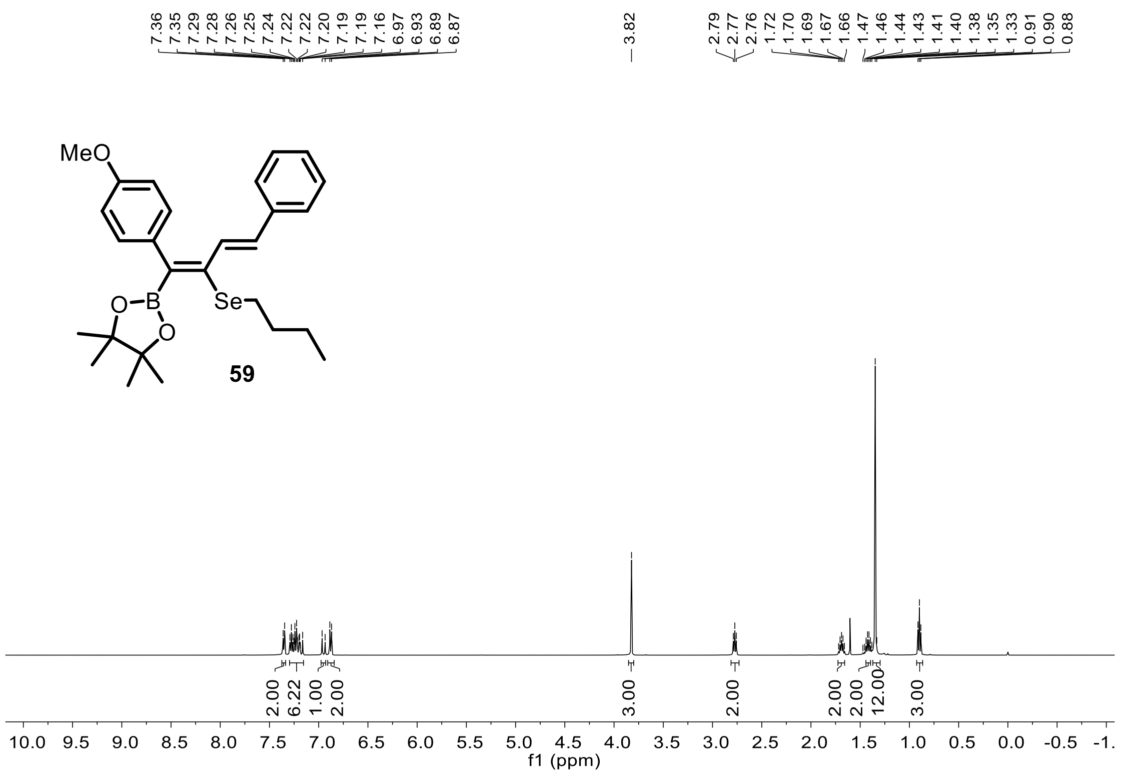

126 MHz, 298 K, CDCl₃ as solvent

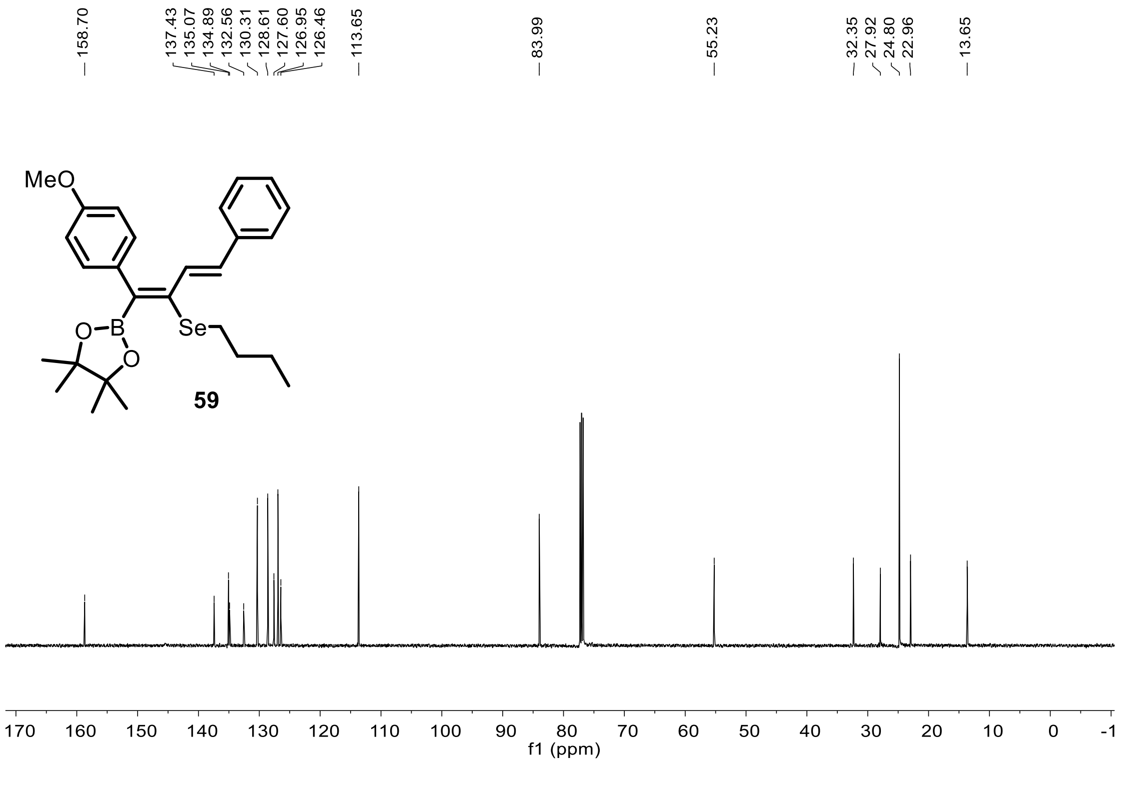

500 MHz, 298 K, $CDCl_3$ as solvent

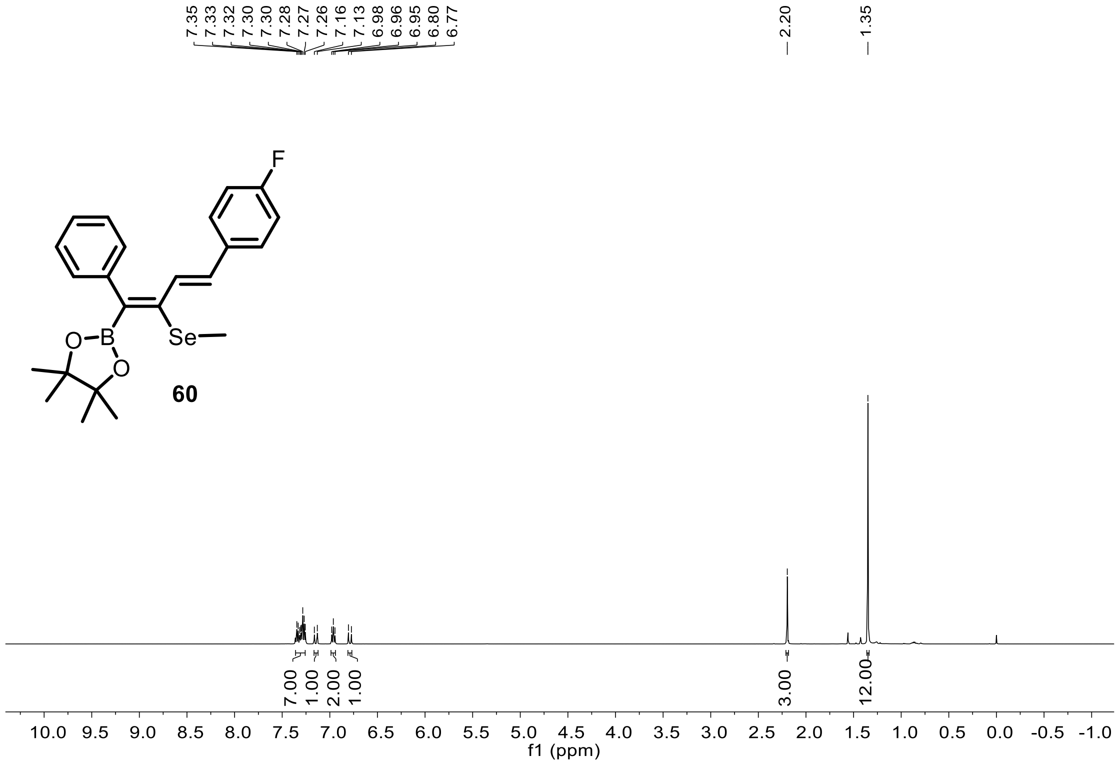

126 MHz, 298 K, $CDCl_3$ as solvent

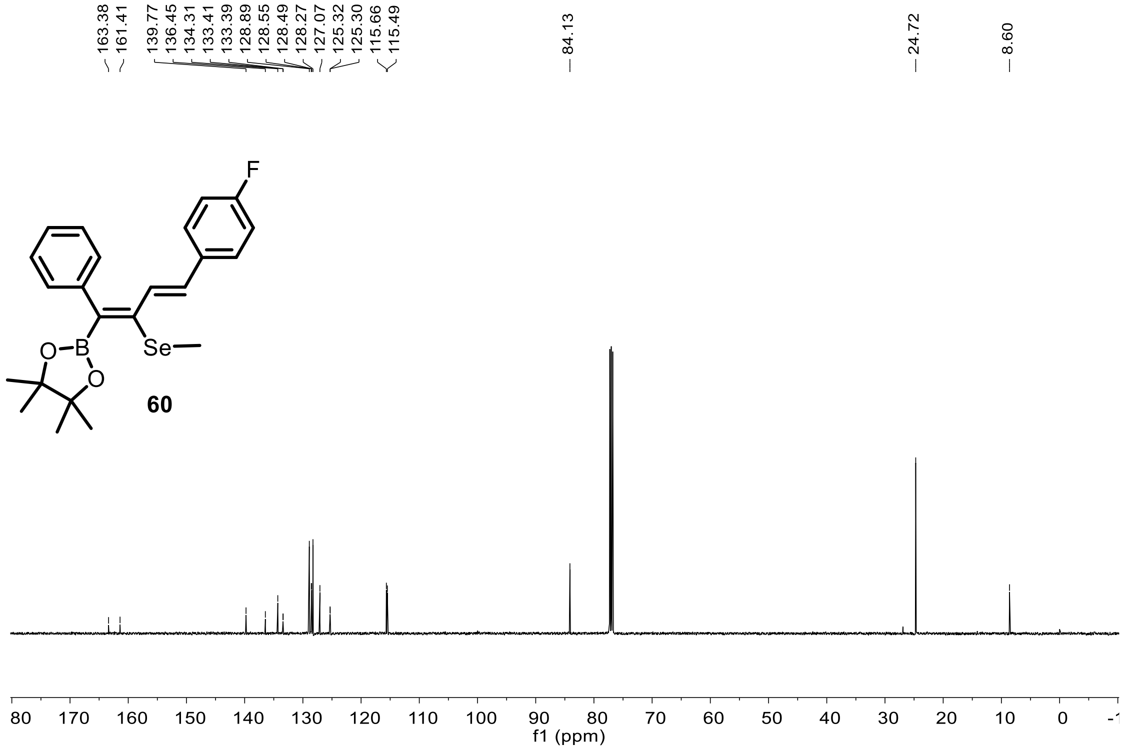

500 MHz, 298 K, $CDCl_3$ as solvent

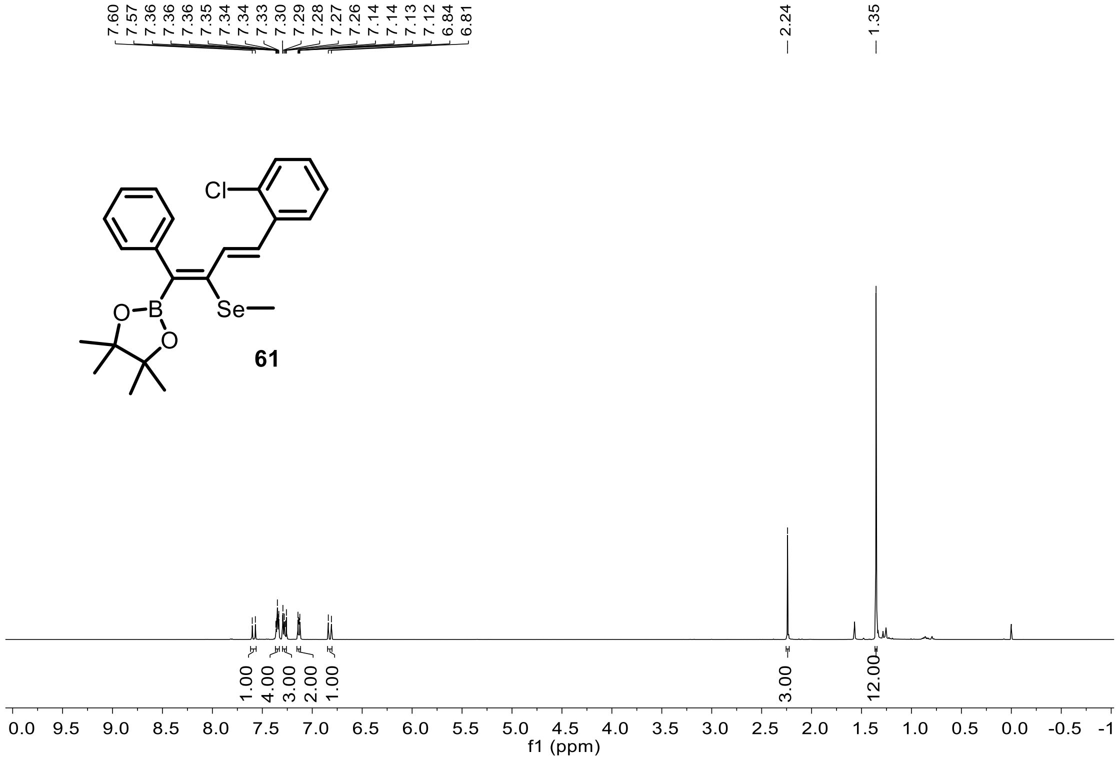

126 MHz, 298 K, $CDCl_3$ as solvent

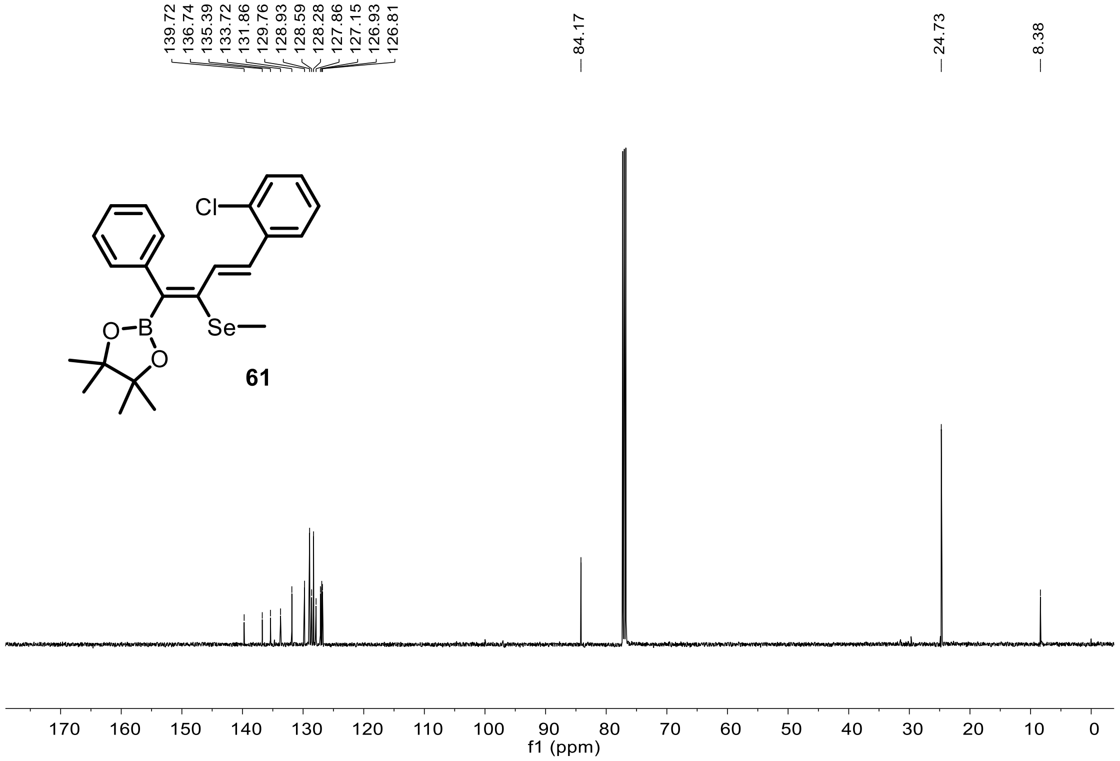

500 MHz, 298 K, $CDCl_3$ as solvent

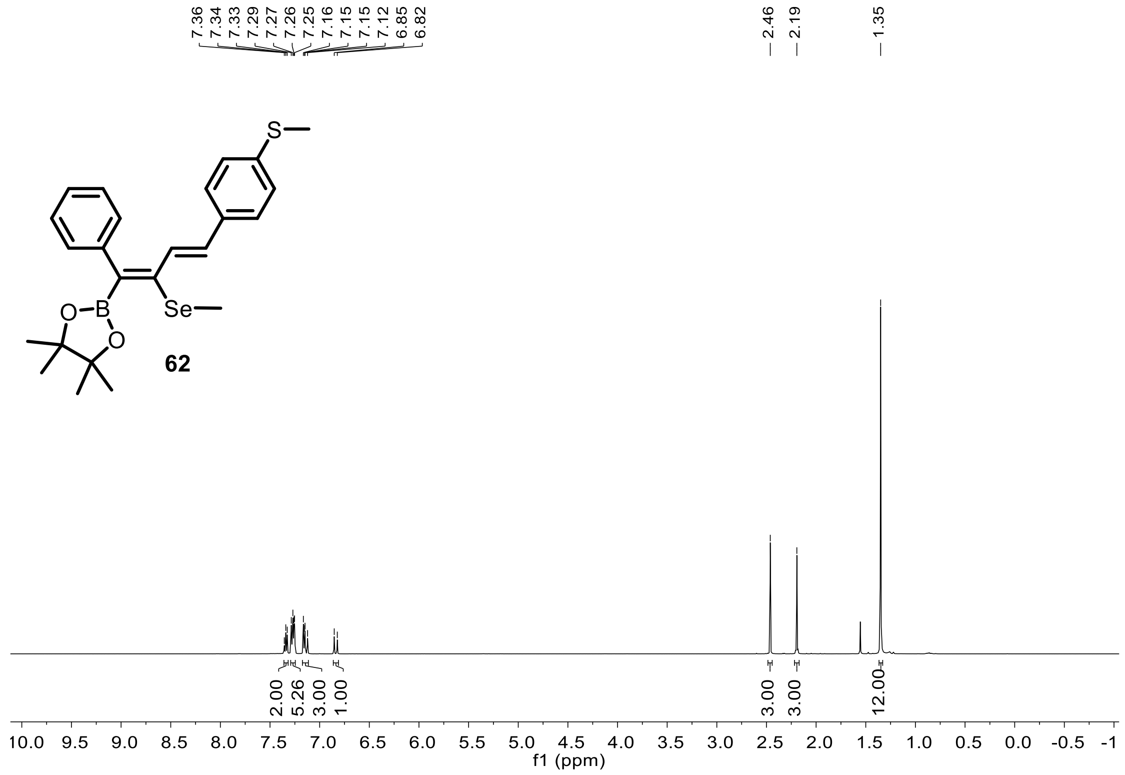

126 MHz, 298 K, $CDCl_3$ as solvent

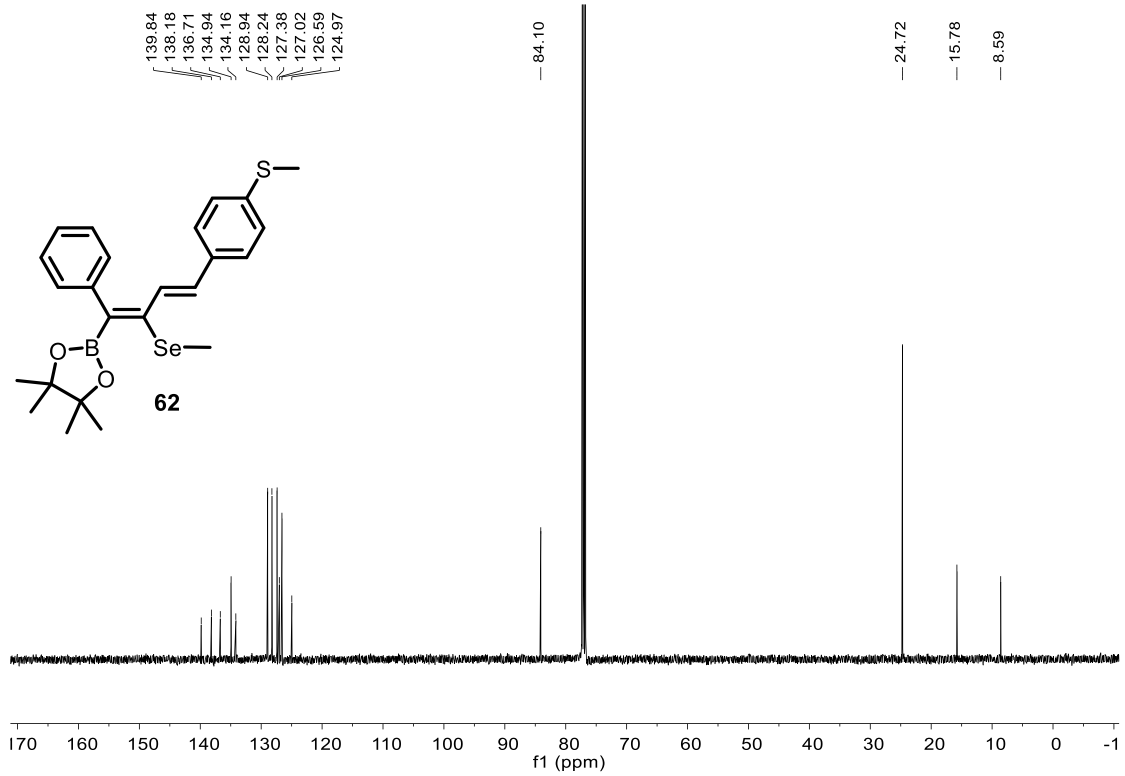

500 MHz, 298 K, CDCl3 as solvent

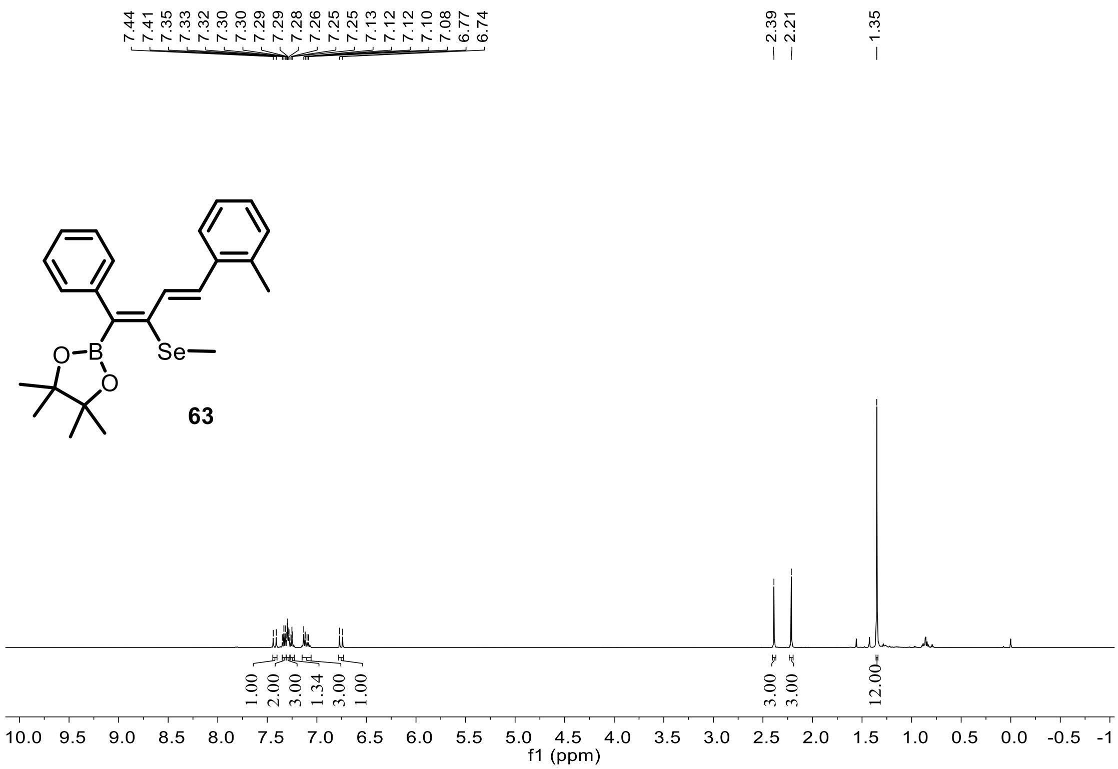

126 MHz, 298 K, CDCl3 as solvent

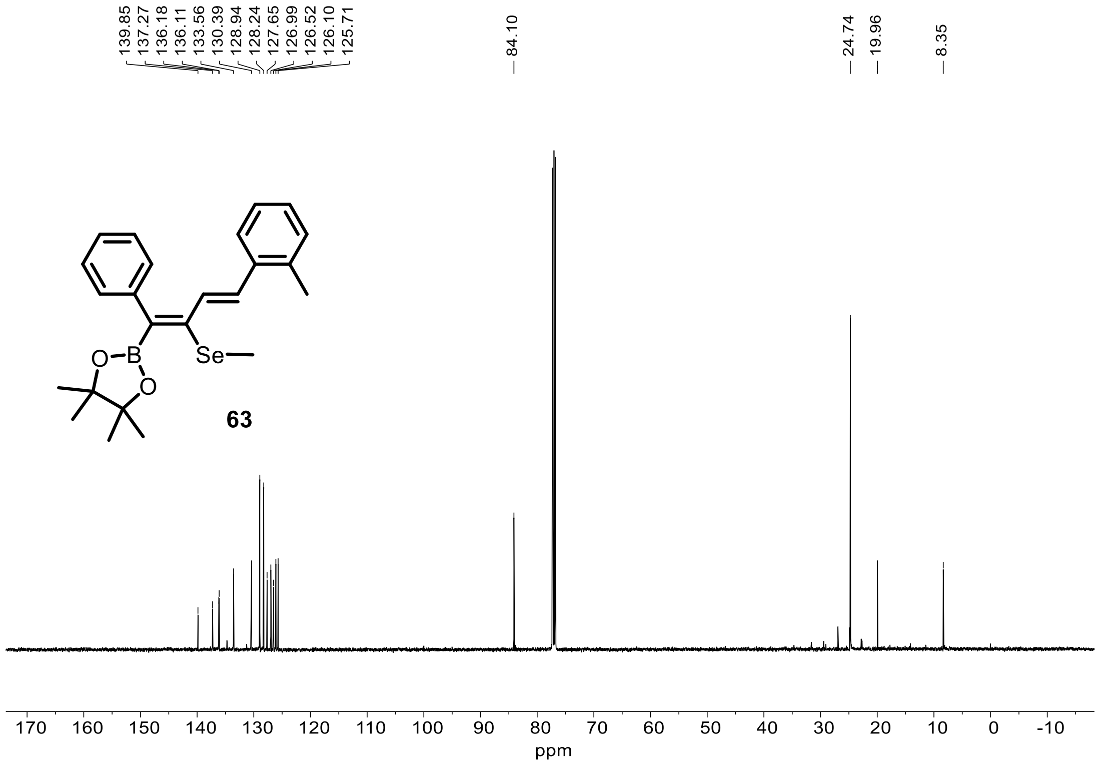

500 MHz, 298 K, $CDCl_3$ as solvent

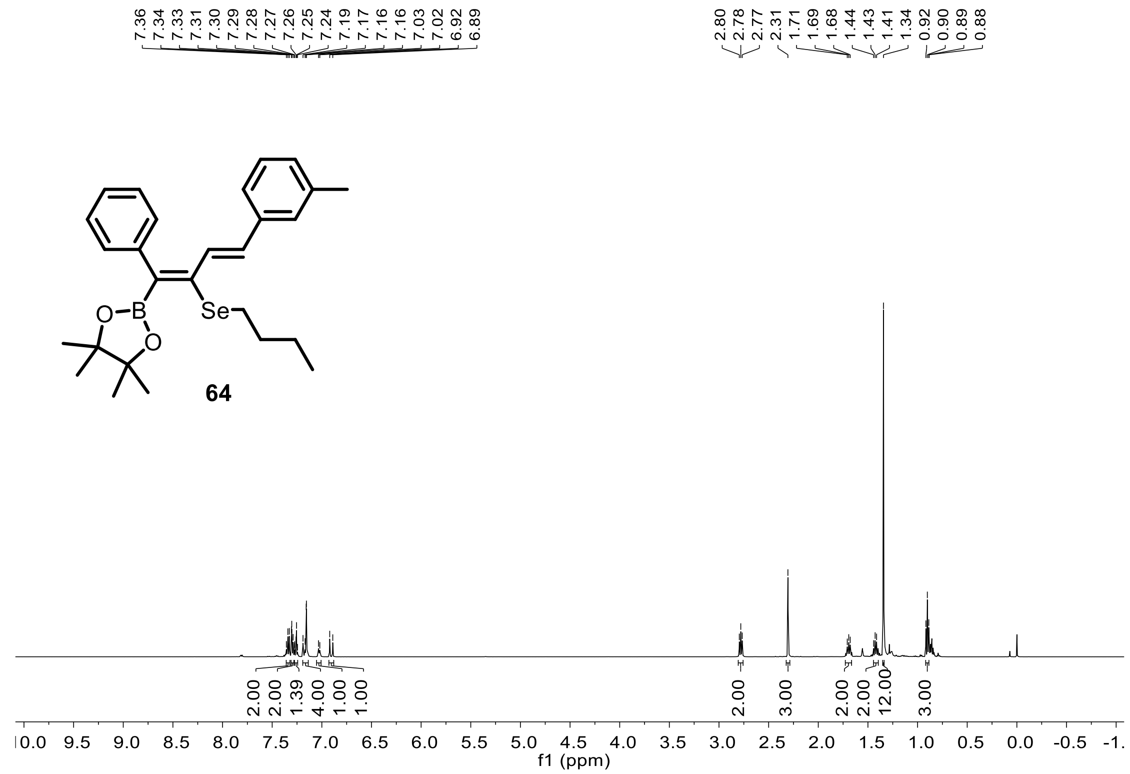

126 MHz, 298 K, $CDCl_3$ as solvent

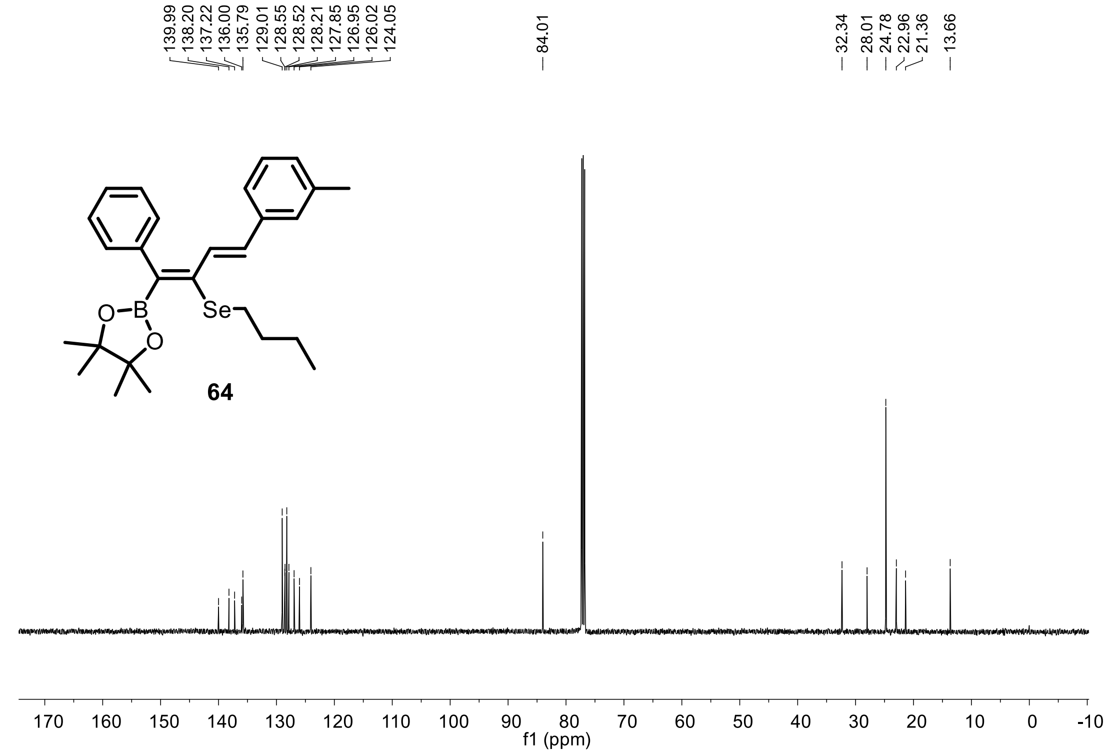

500 MHz, 298 K, $CDCl_3$ as solvent

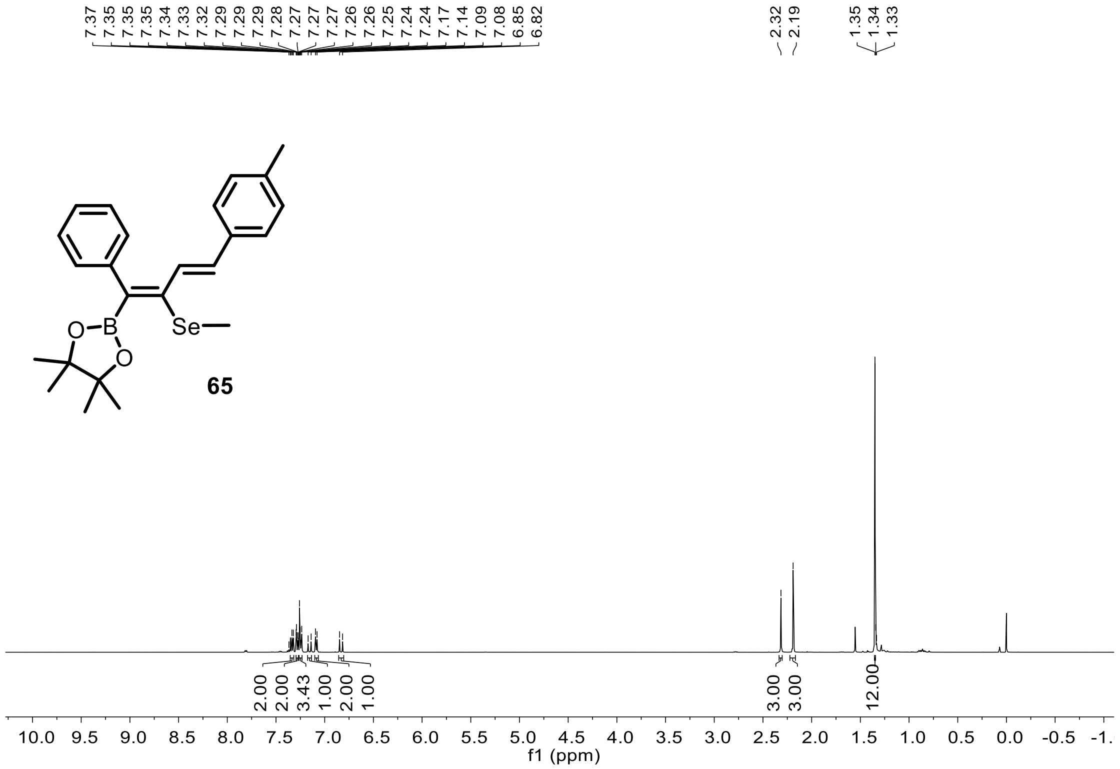

126 MHz, 298 K, $CDCl_3$ as solvent

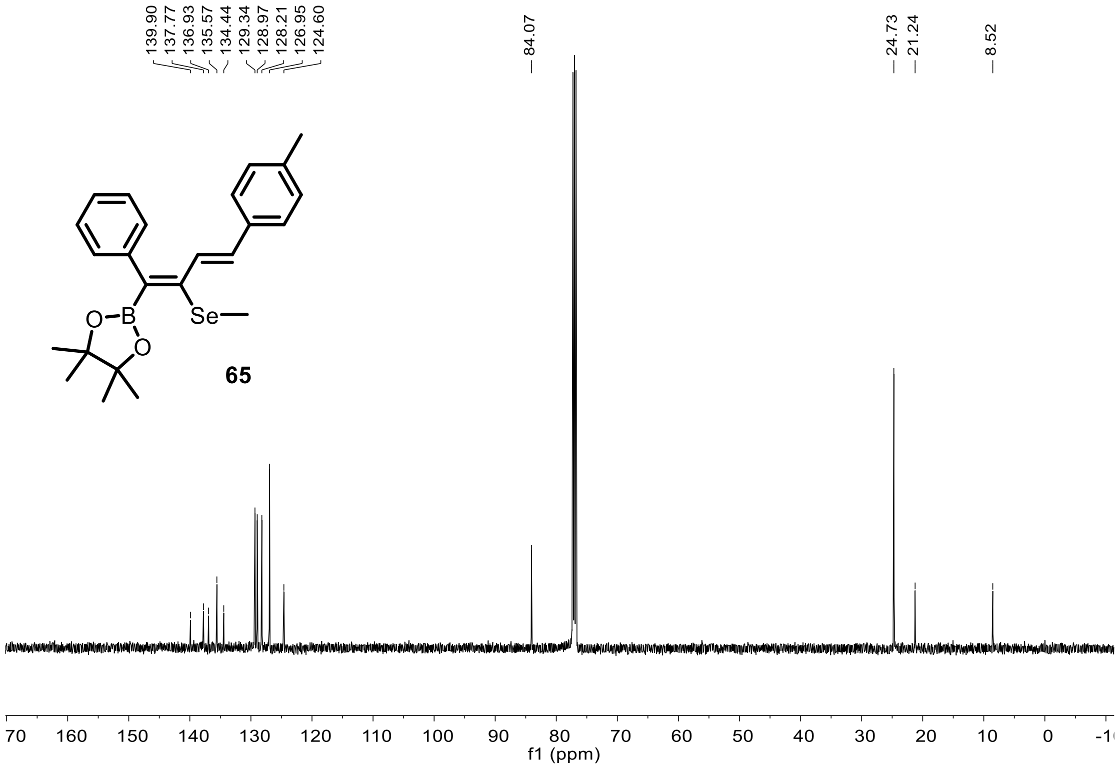

500 MHz, 298 K, CDCl3 as solvent

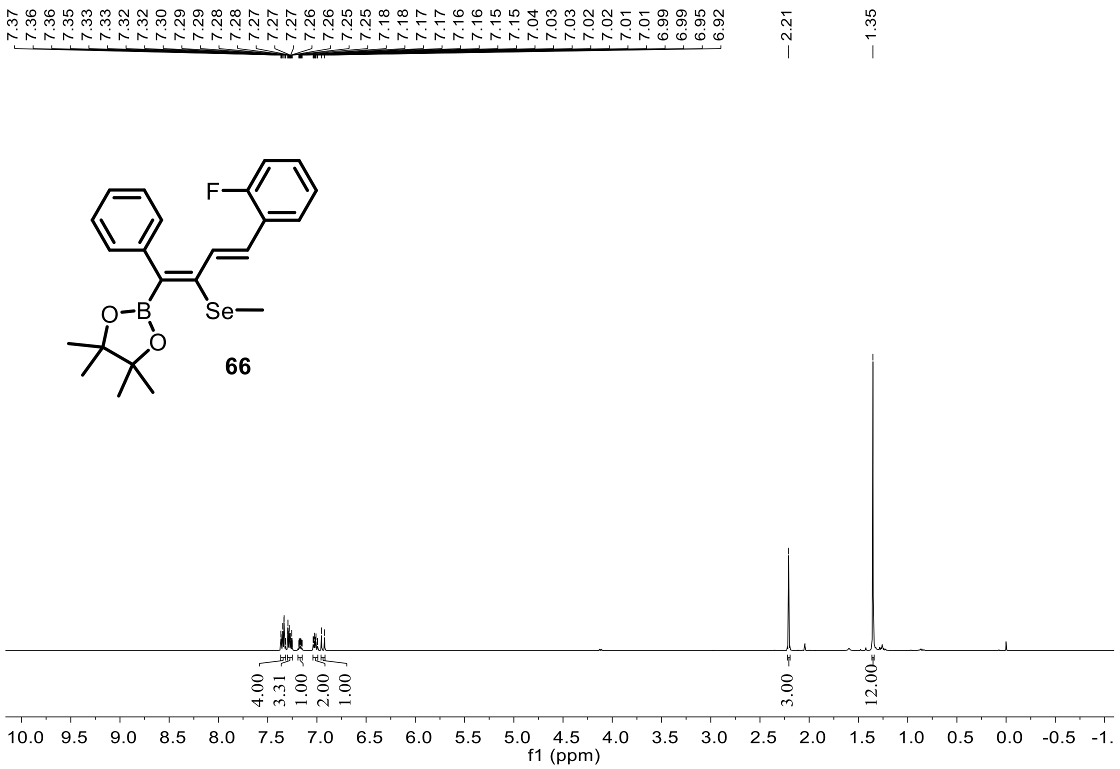

126 MHz, 298 K, CDCl3 as solvent

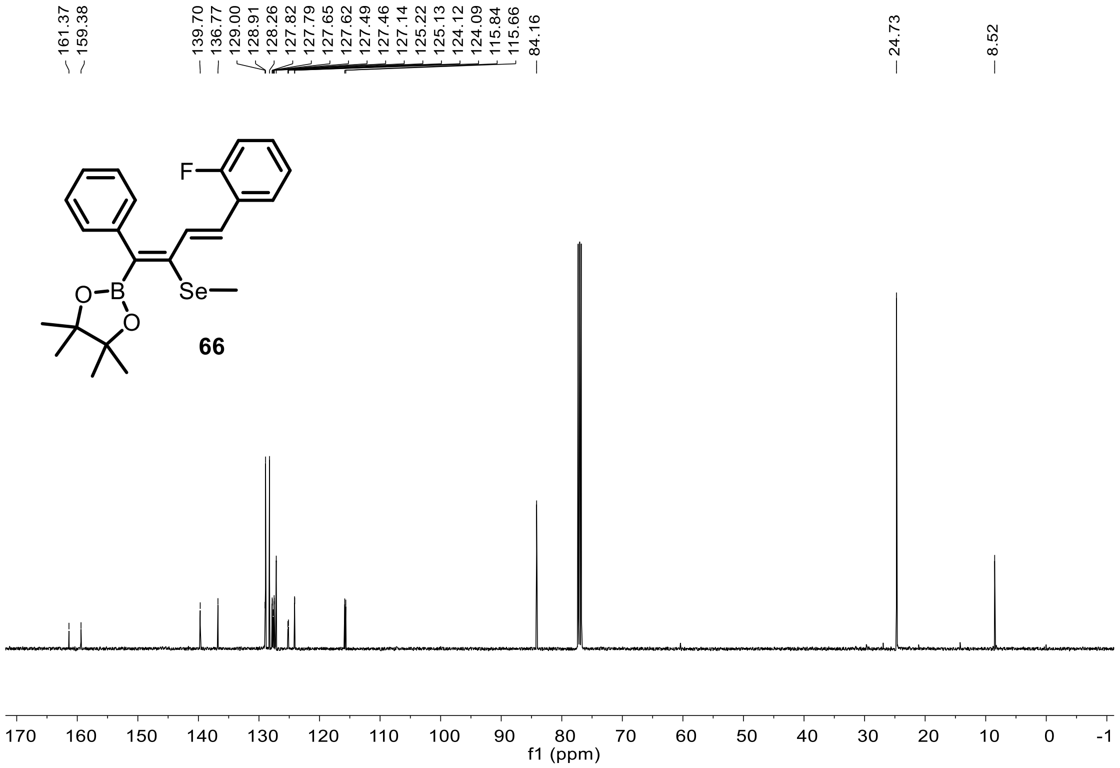

500 MHz, 298 K, $CDCl_3$ as solvent

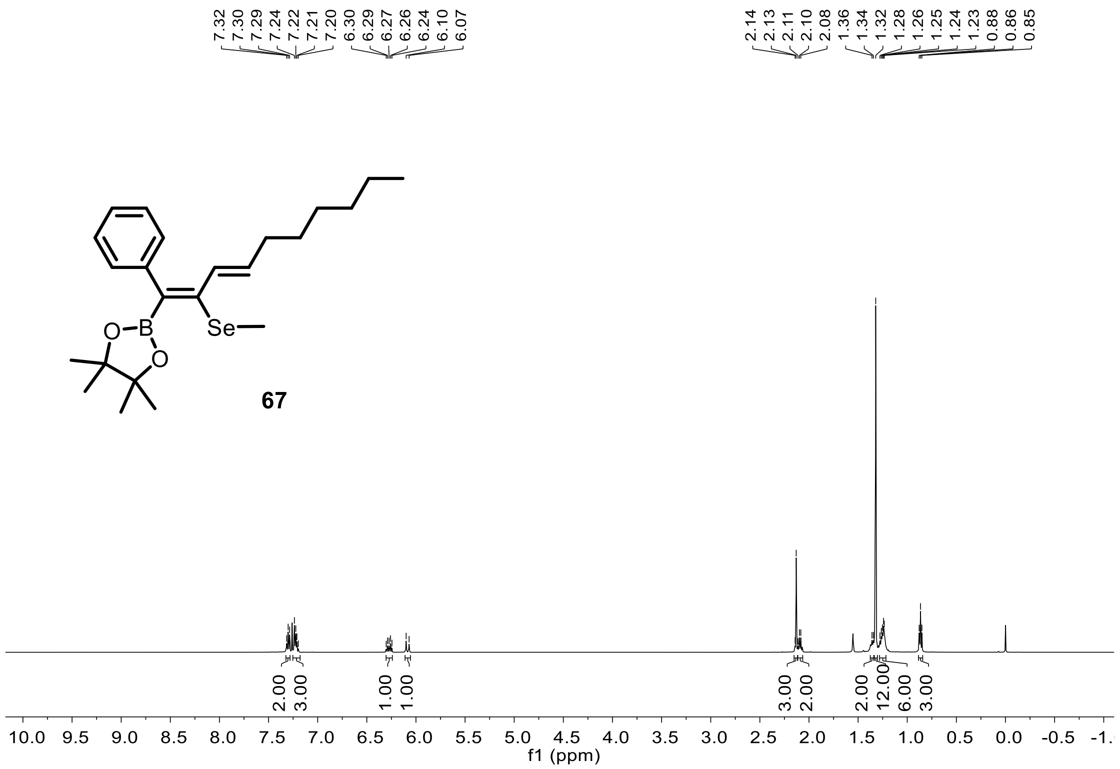

126 MHz, 298 K, $CDCl_3$ as solvent

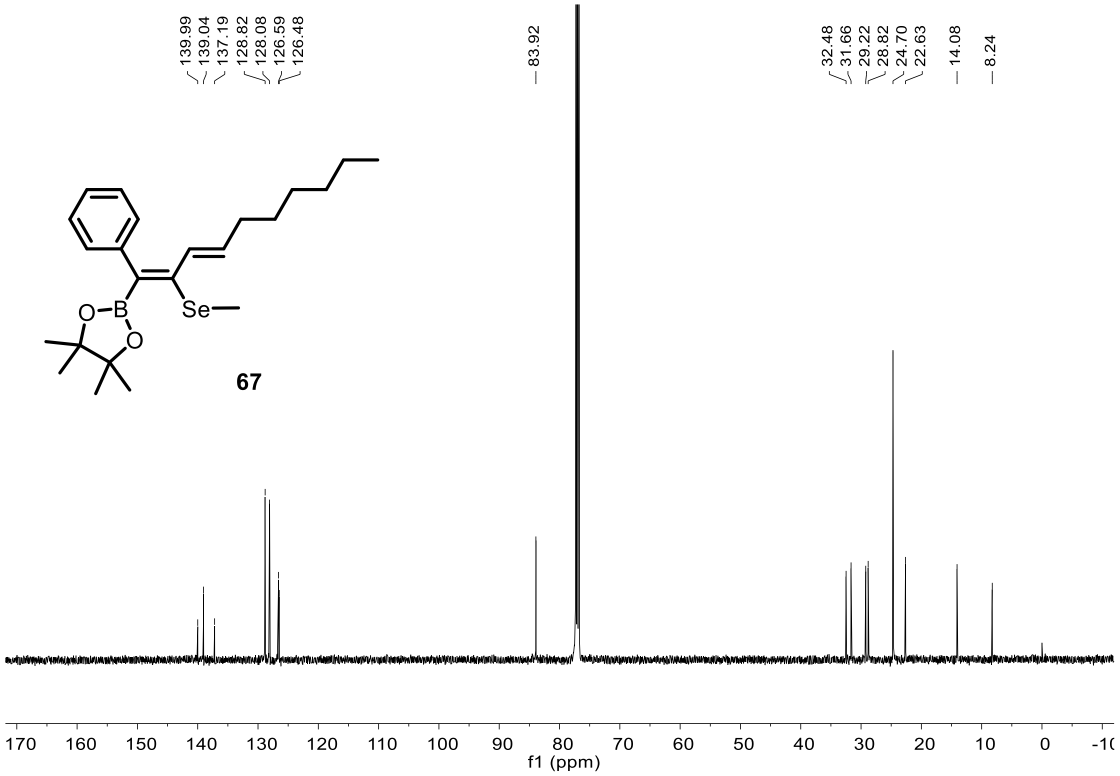

500 MHz, 298 K, $CDCl_3$ as solvent

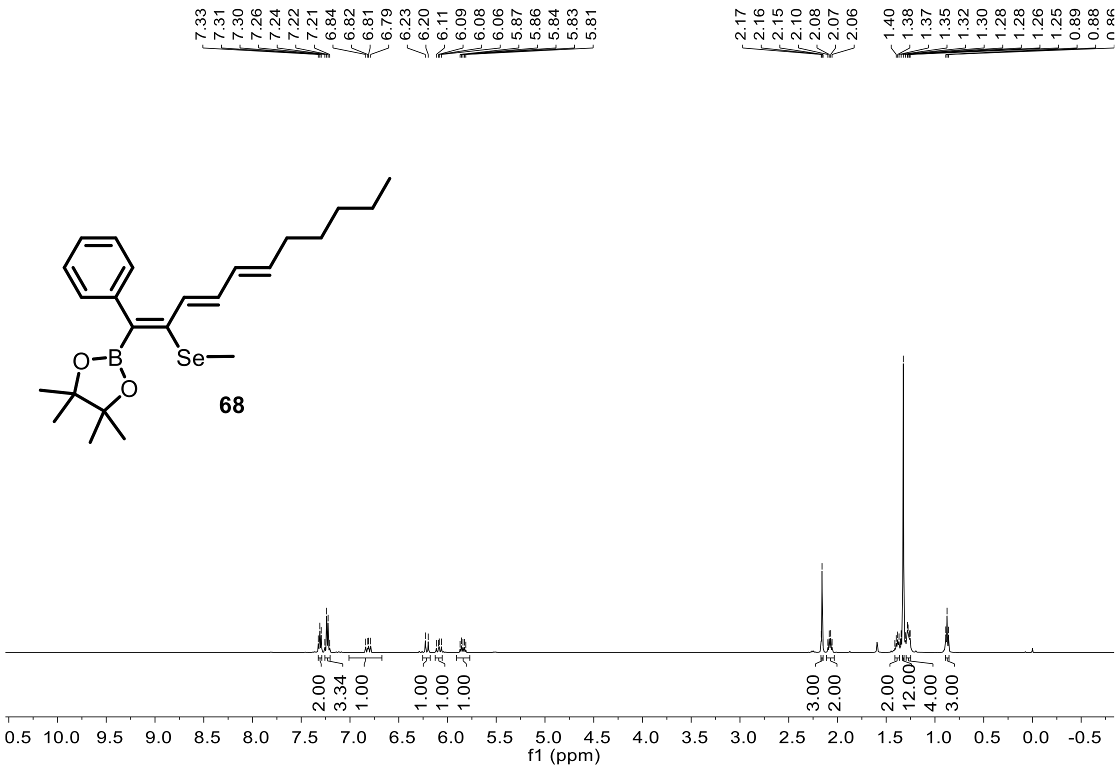

126 MHz, 298 K, $CDCl_3$ as solvent

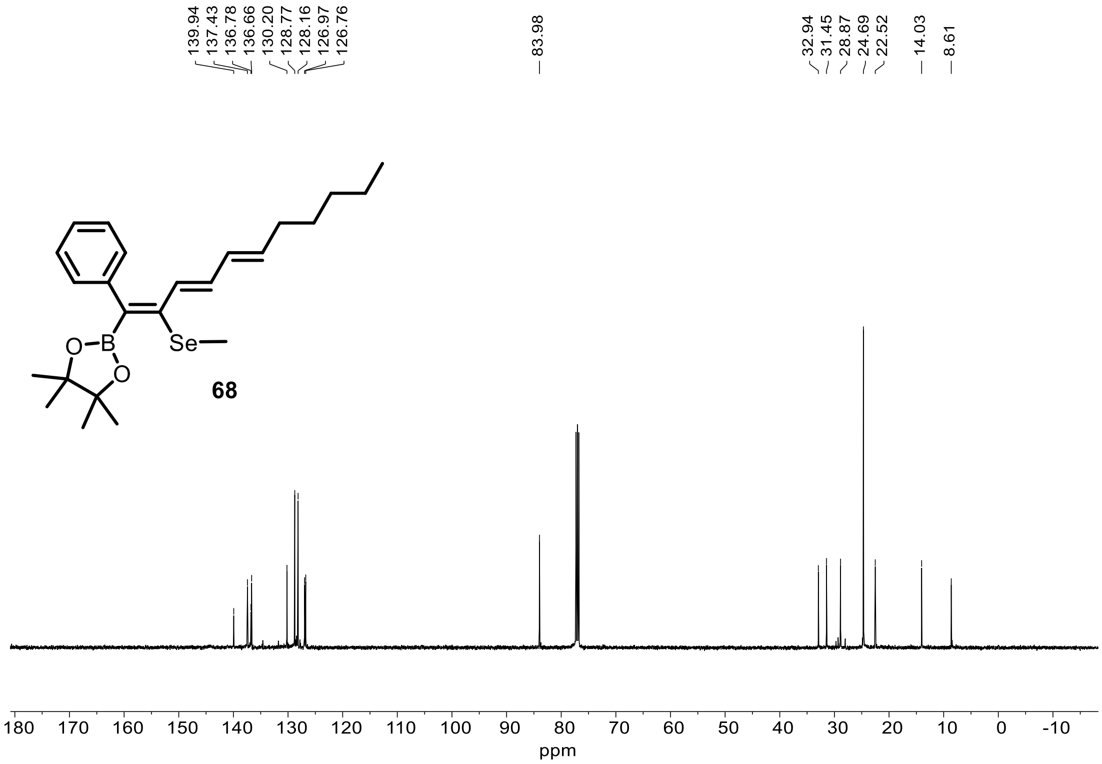

500 MHz, 298 K, $CDCl_3$ as solvent

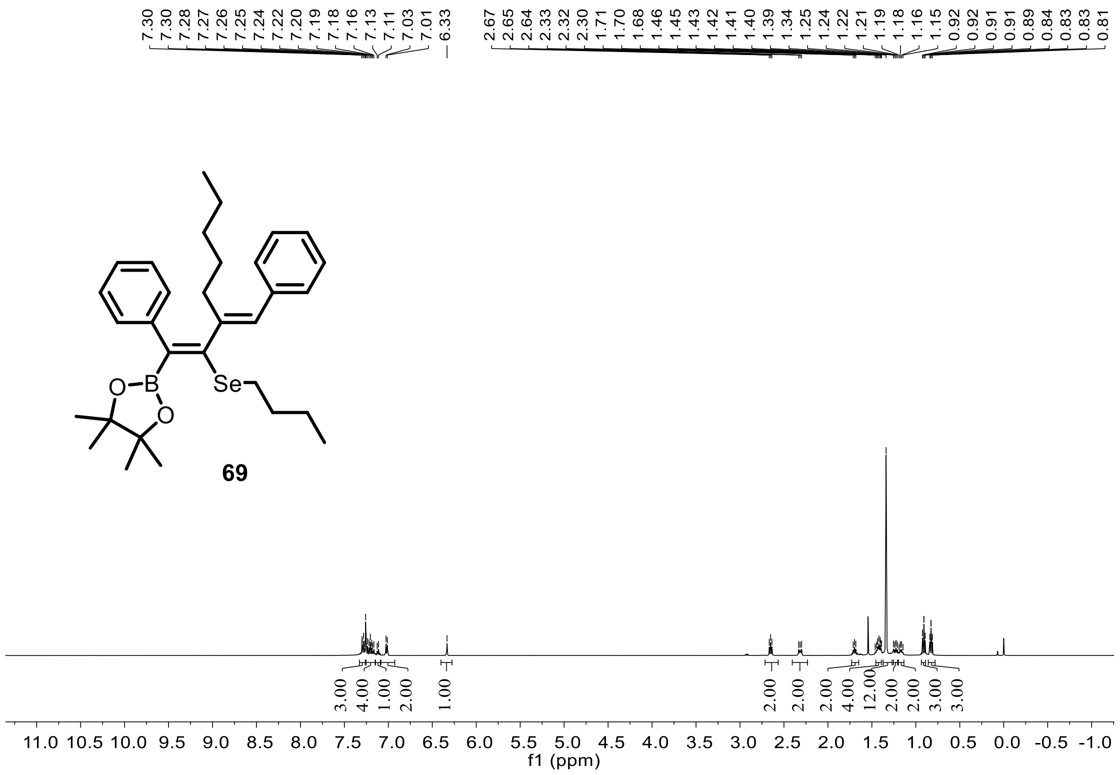

126 MHz, 298 K, $CDCl_3$ as solvent

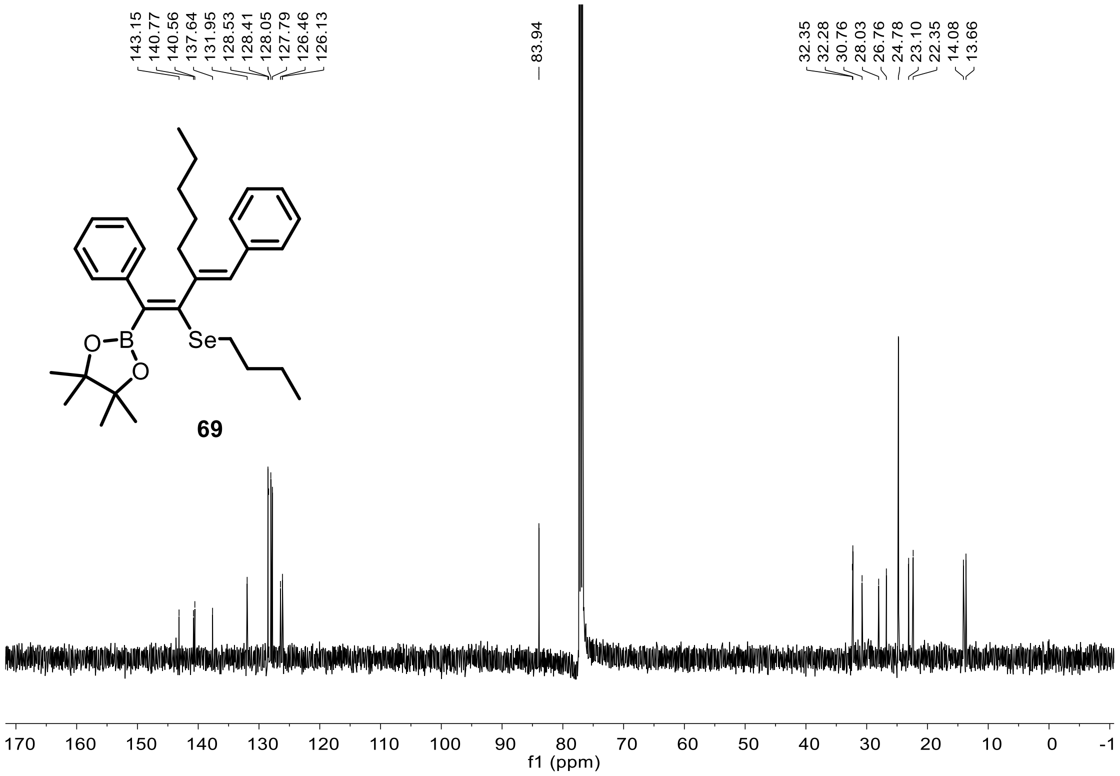

500 MHz, 298 K, $CDCl_3$ as solvent

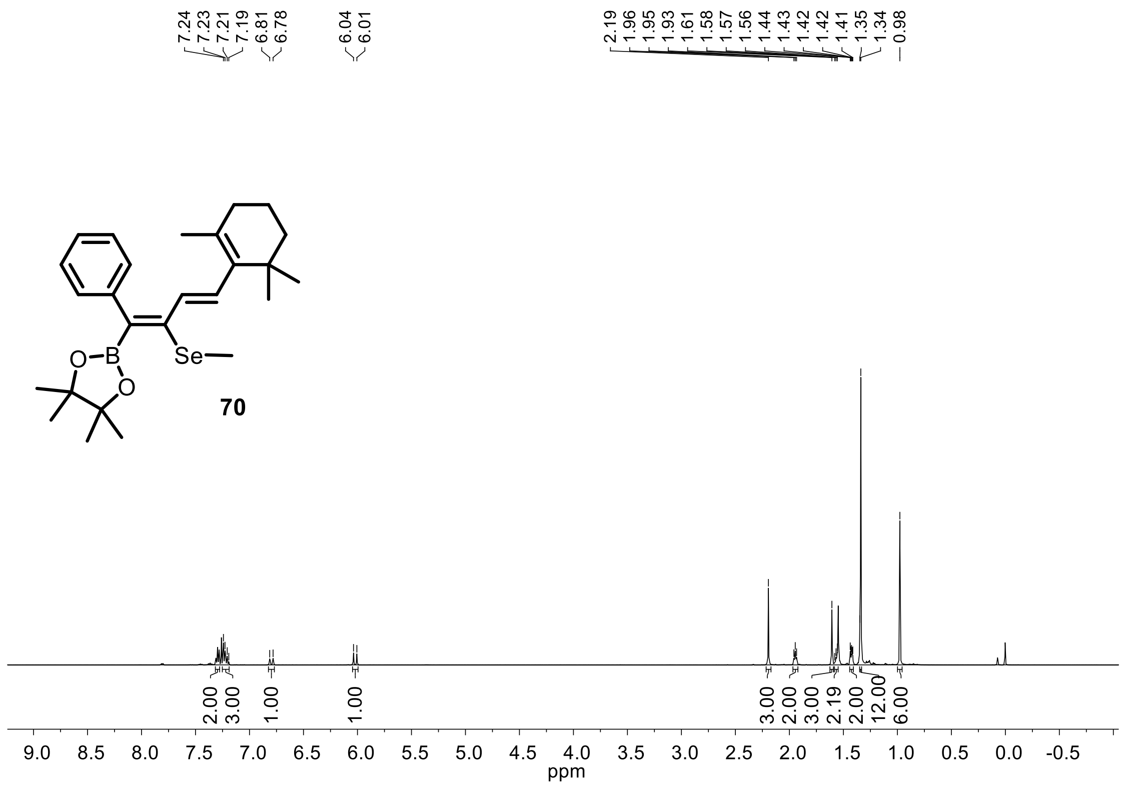

126 MHz, 298 K, $CDCl_3$ as solvent

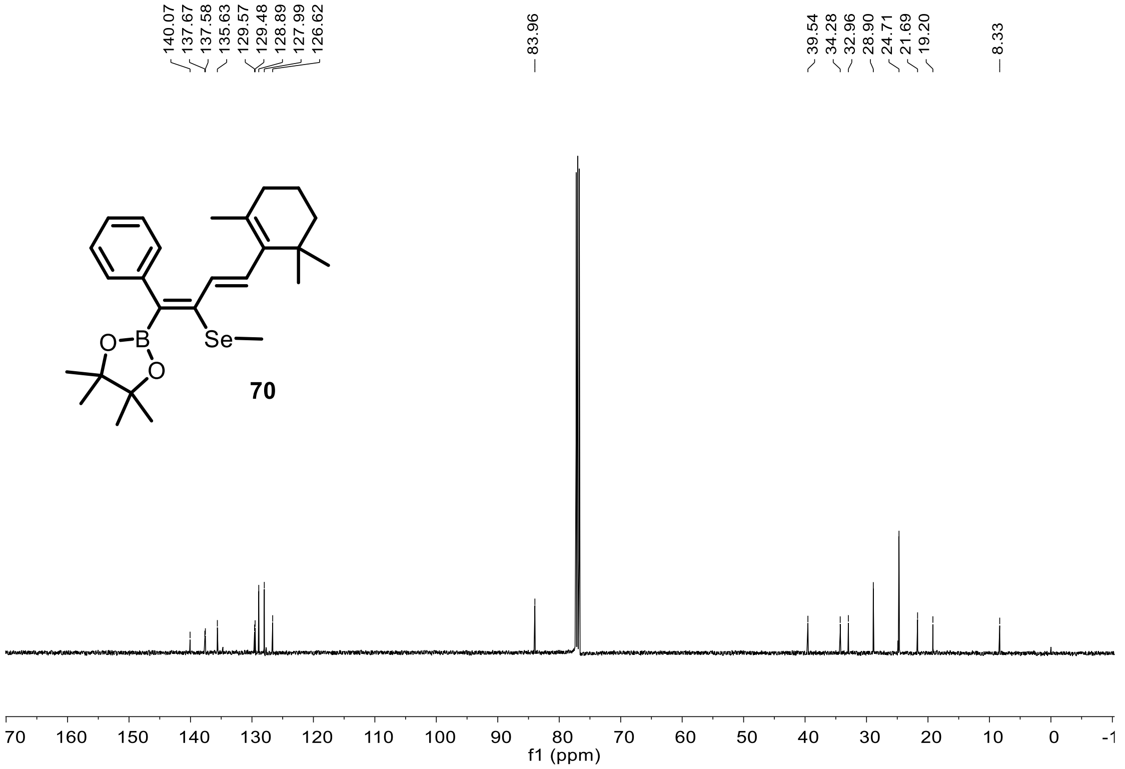

500 MHz, 298 K, $CDCl_3$ as solvent

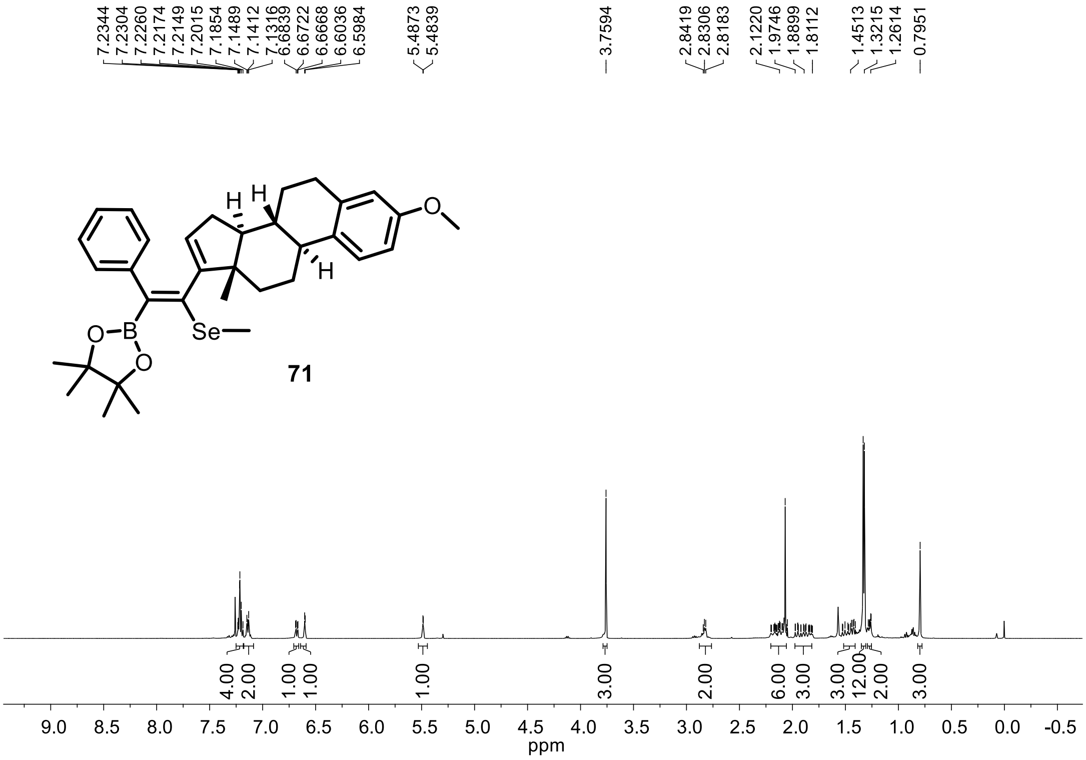

126 MHz, 298 K, $CDCl_3$ as solvent

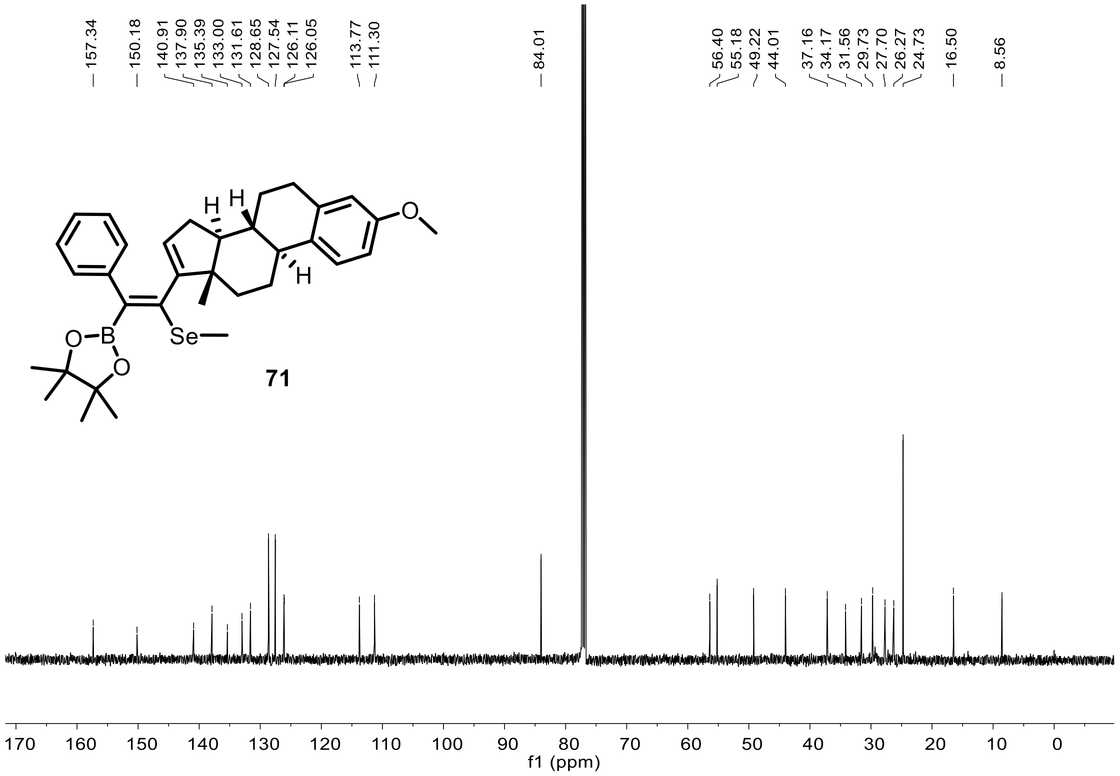

500 MHz, 298 K, $CDCl_3$ as solvent

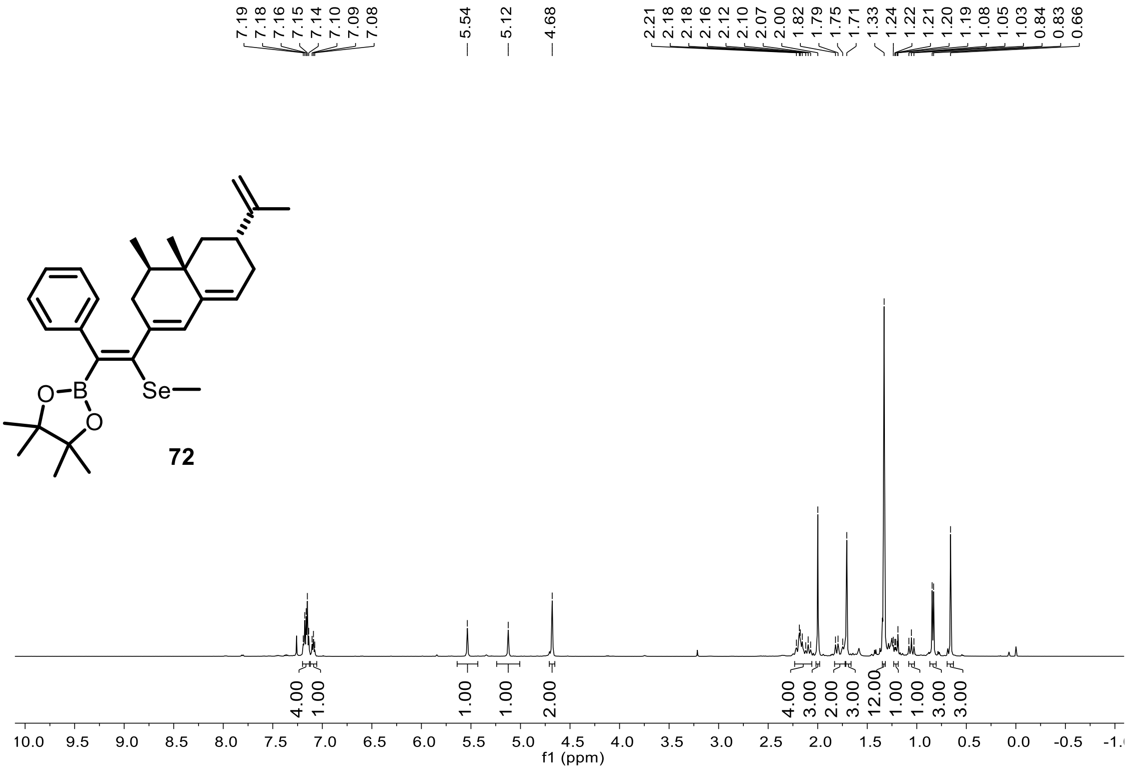

126 MHz, 298 K, $CDCl_3$ as solvent

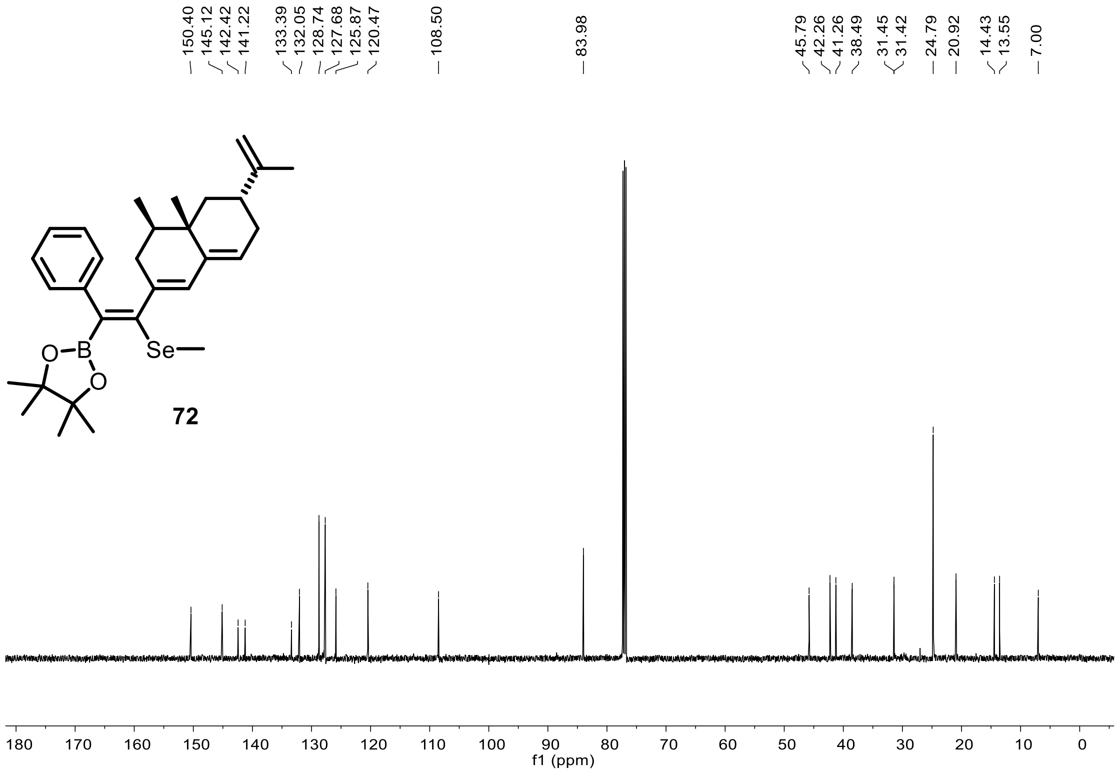

500 MHz, 298 K, $CDCl_3$ as solvent

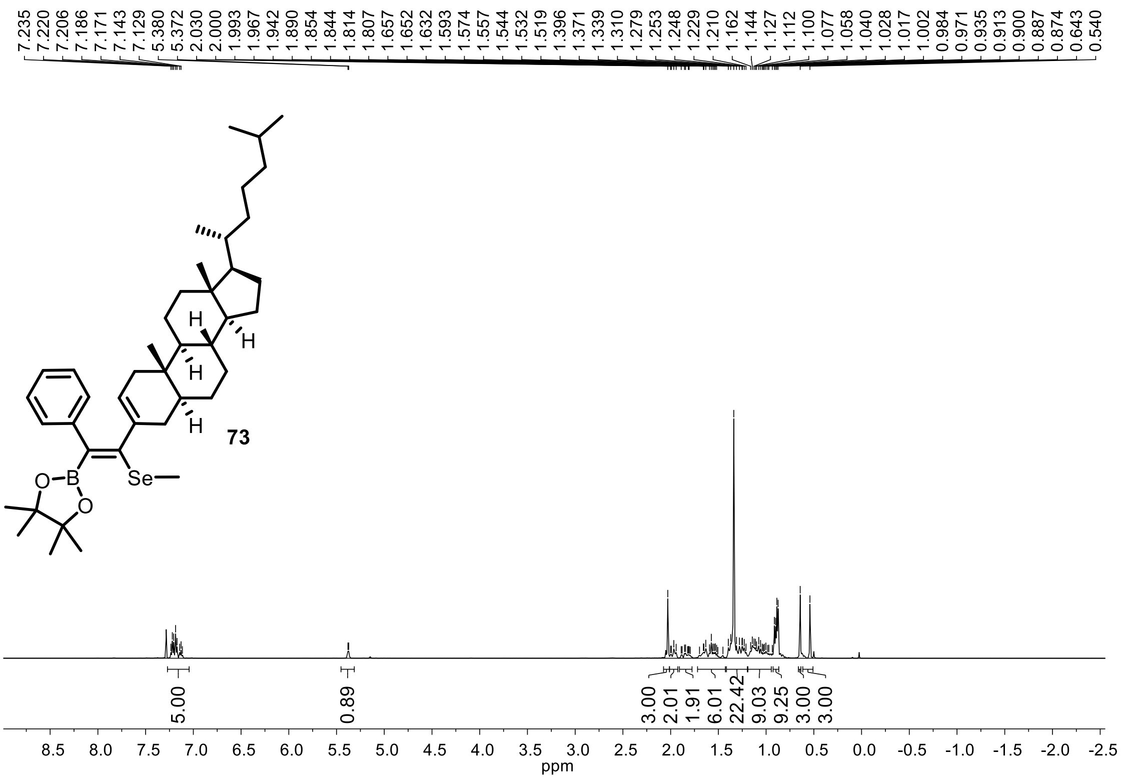

126 MHz, 298 K, $CDCl_3$ as solvent

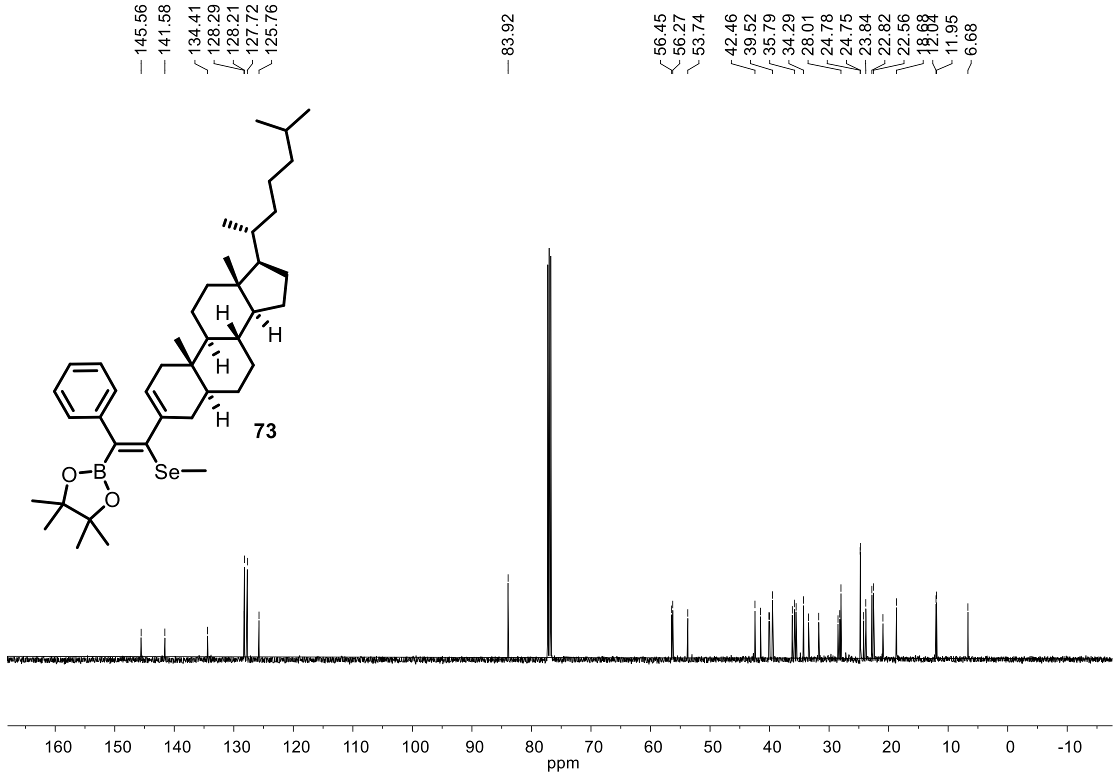

500 MHz, 298 K, $CDCl_3$ as solvent

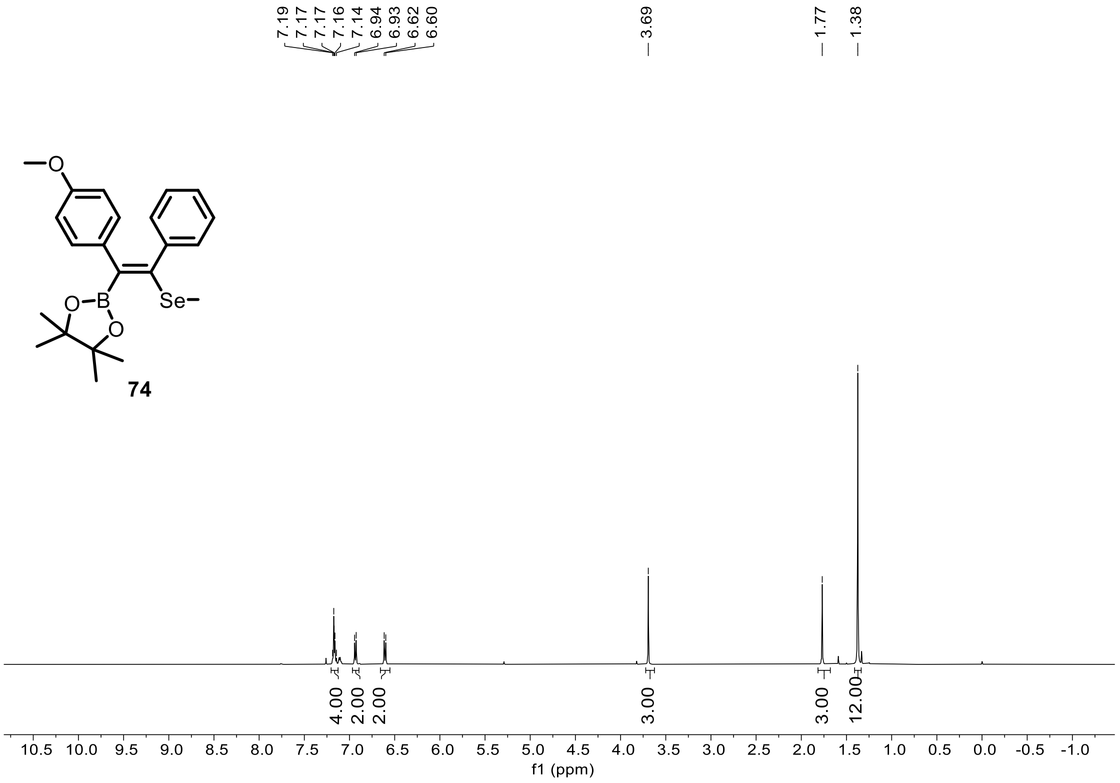

126 MHz, 298 K, $CDCl_3$ as solvent

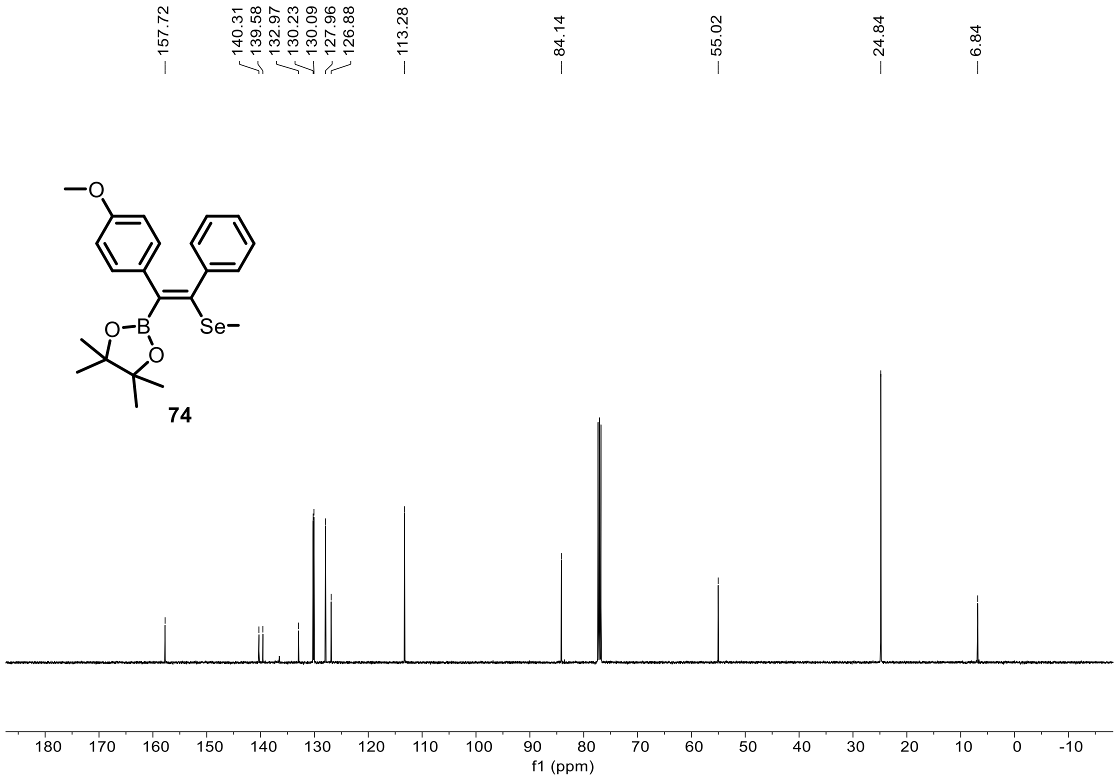

500 MHz, 298 K, CDCl3 as solvent

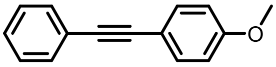

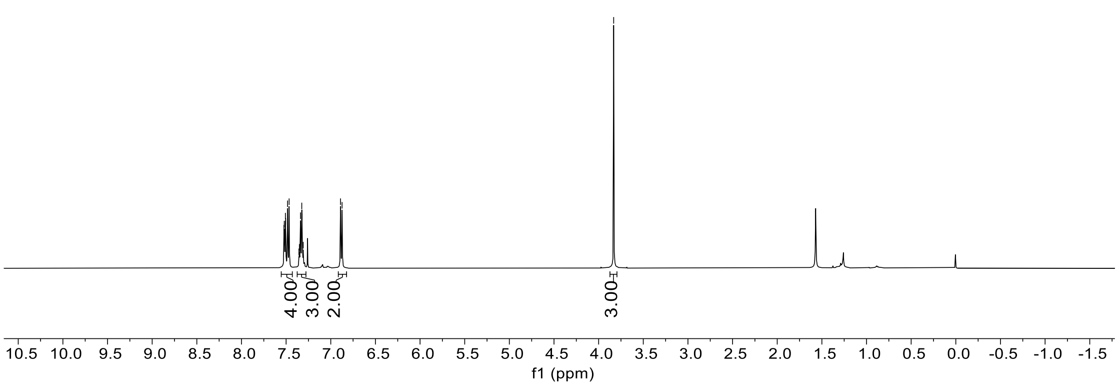

75

126 MHz, 298 K, CDCl3 as solvent

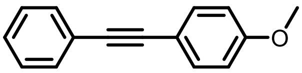

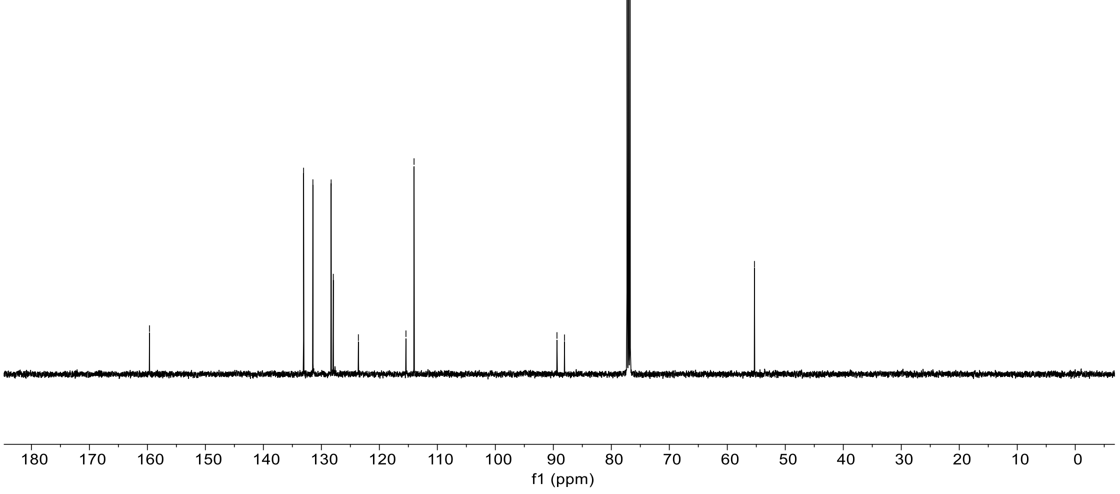

75

500 MHz, 298 K, $CDCl_3$ as solvent

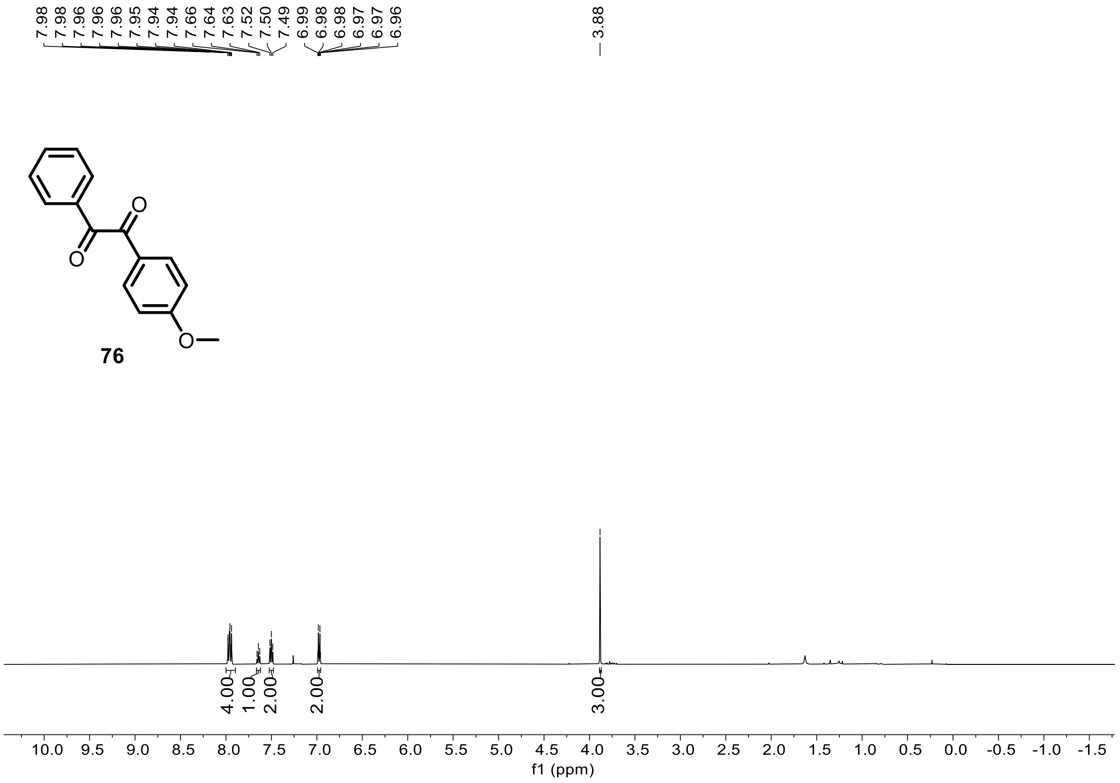

126 MHz, 298 K, $CDCl_3$ as solvent

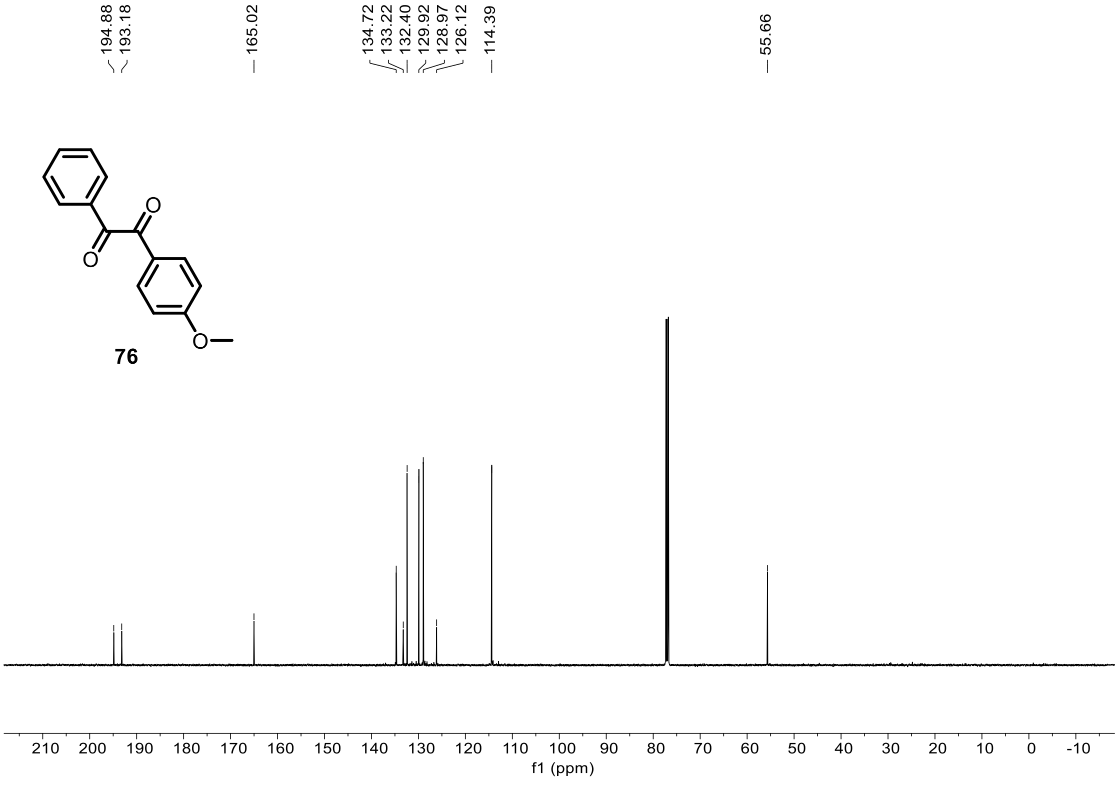

500 MHz, 298 K, CDCl3 as solvent

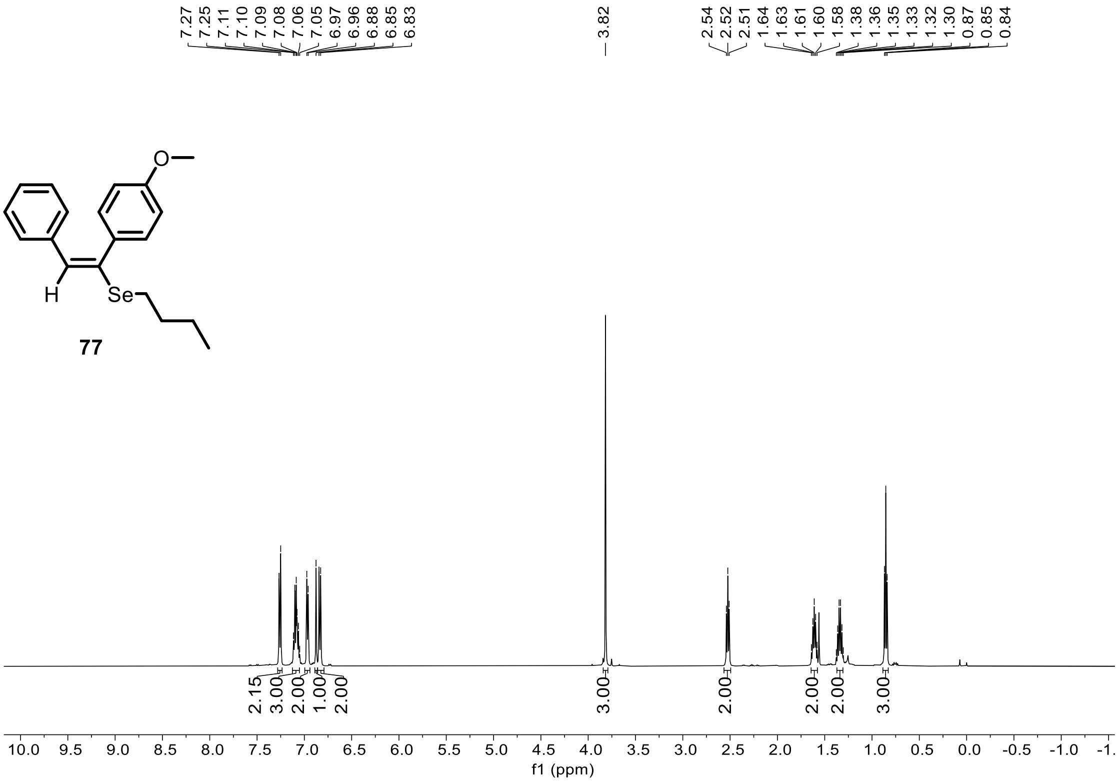

126 MHz, 298 K, CDCl3 as solvent

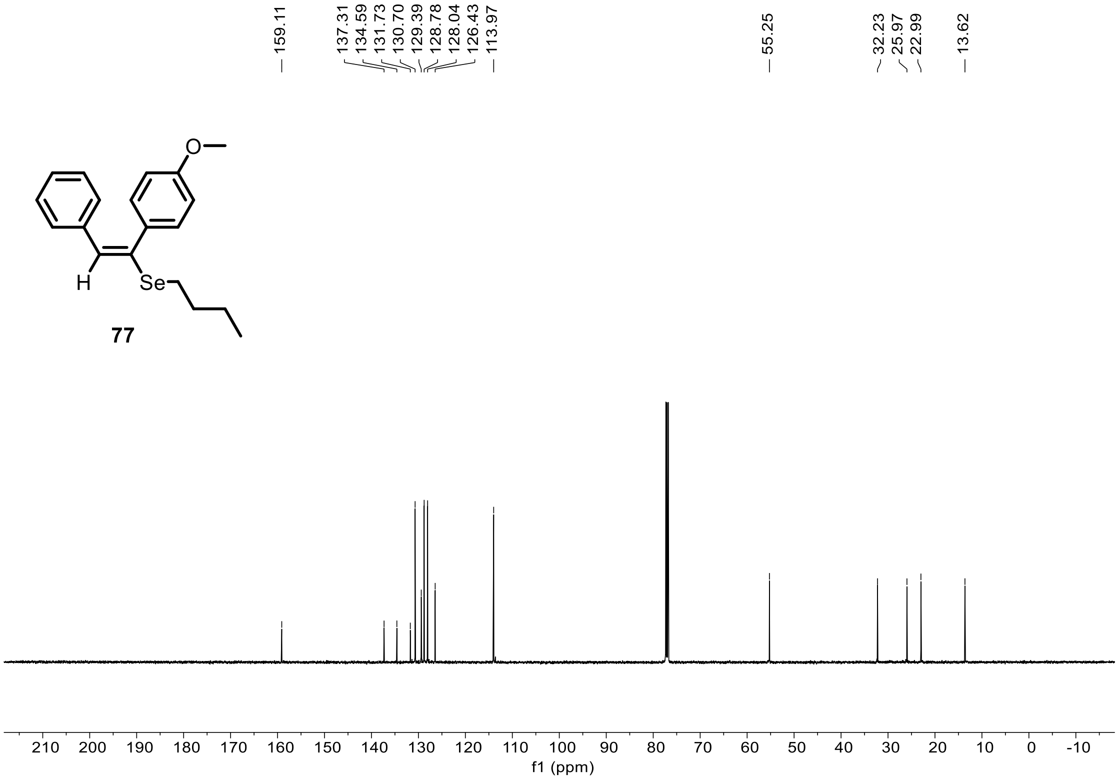

500 MHz, 298 K, CDCl3 as solvent

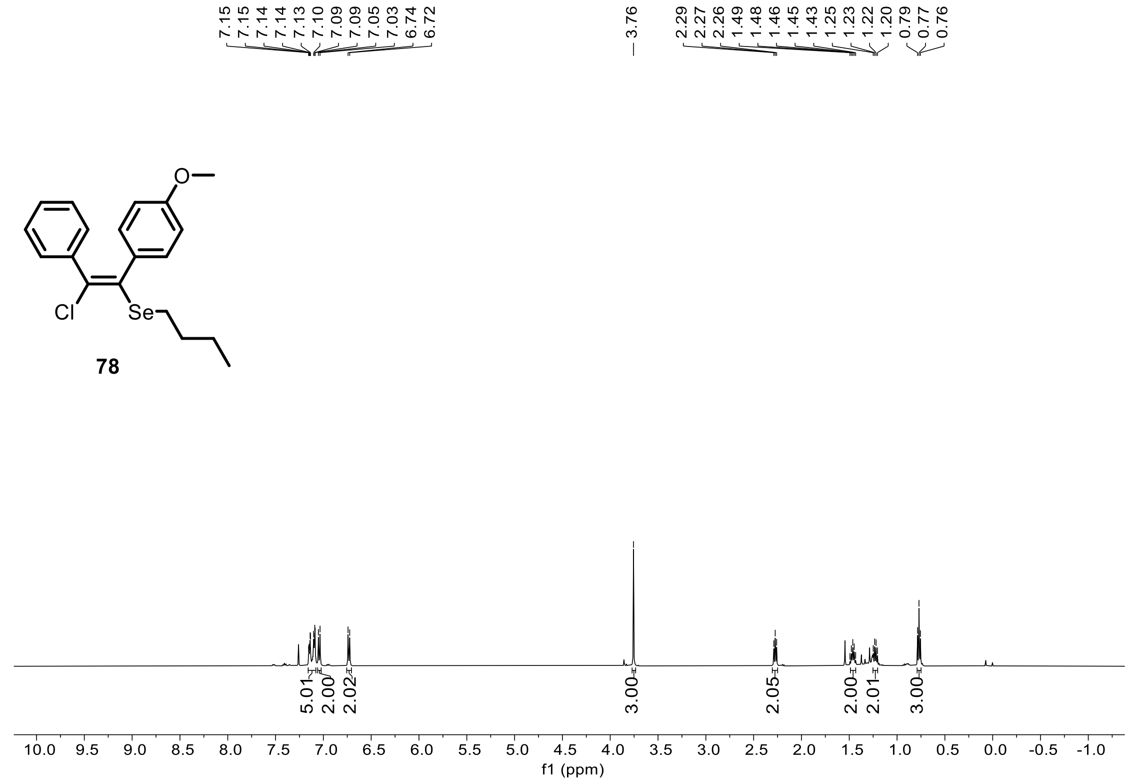

126 MHz, 298 K, CDCl3 as solvent

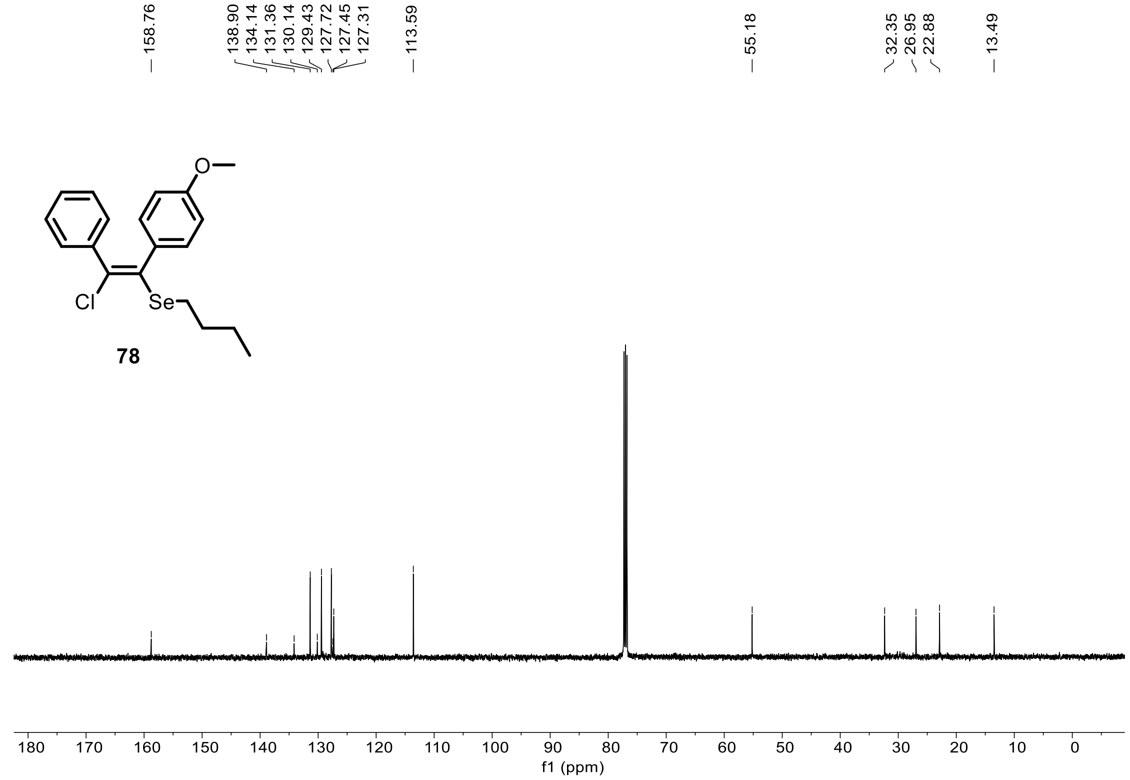

500 MHz, 298 K, CDCl₃ as solvent

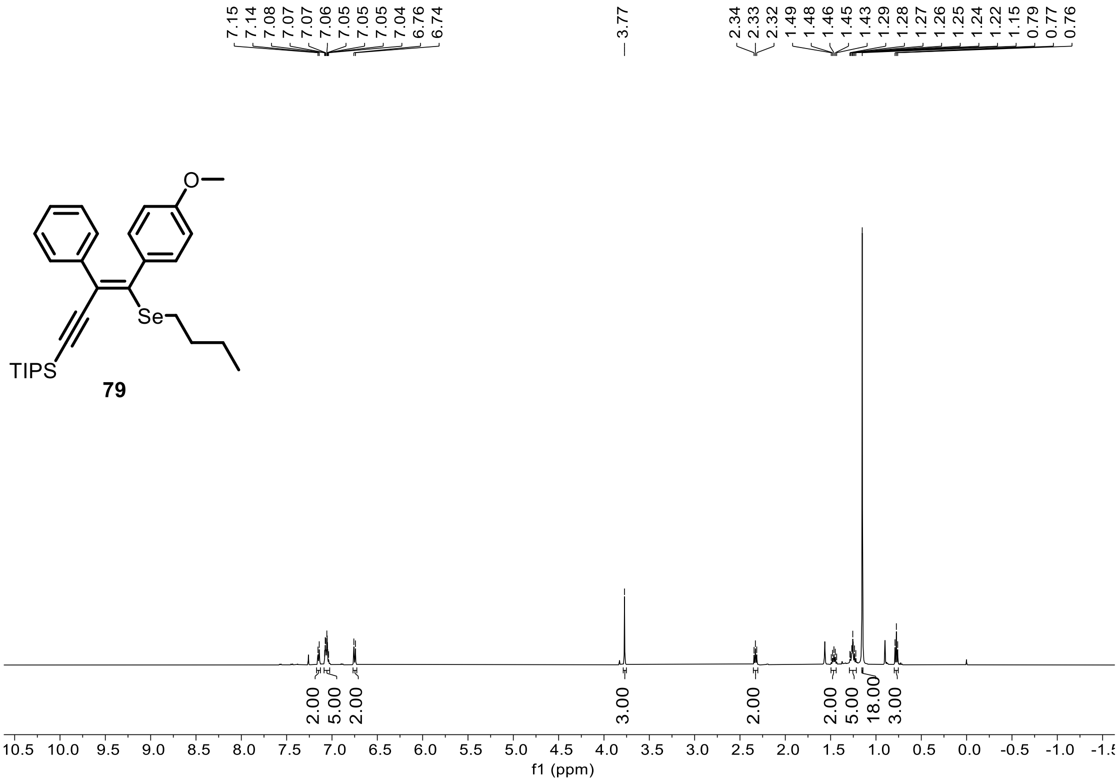

126 MHz, 298 K, CDCl₃ as solvent

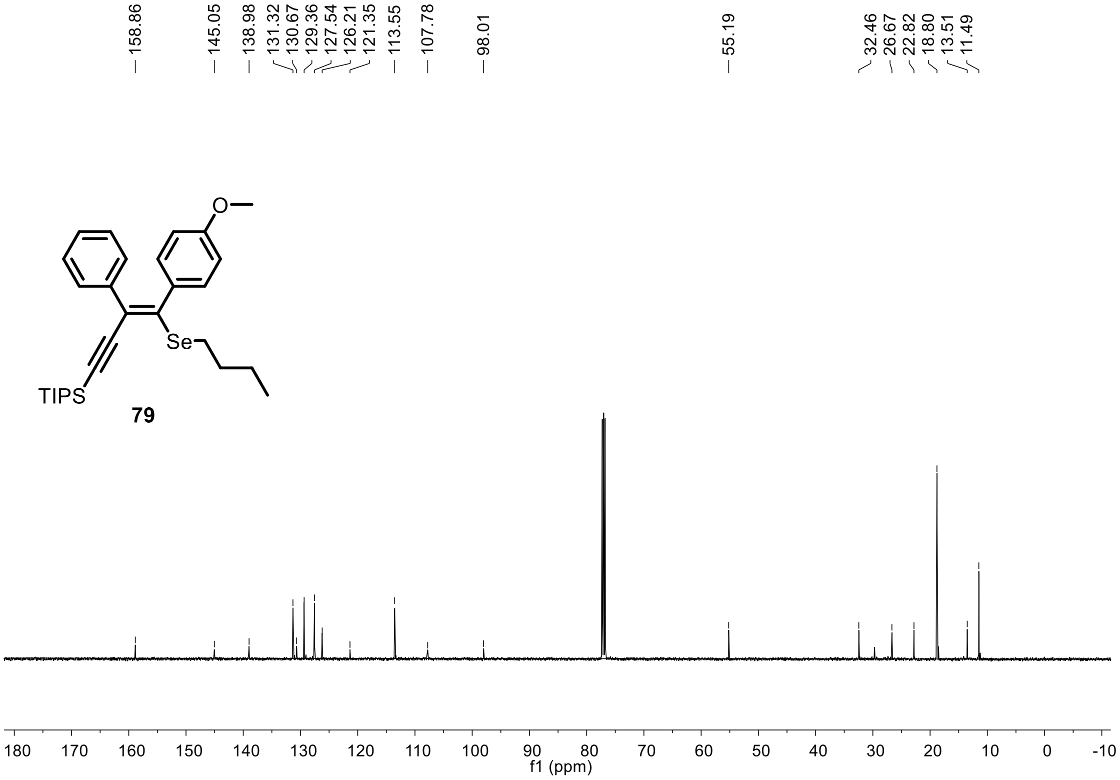

500 MHz, 298 K, CD_2Cl_2 as solvent

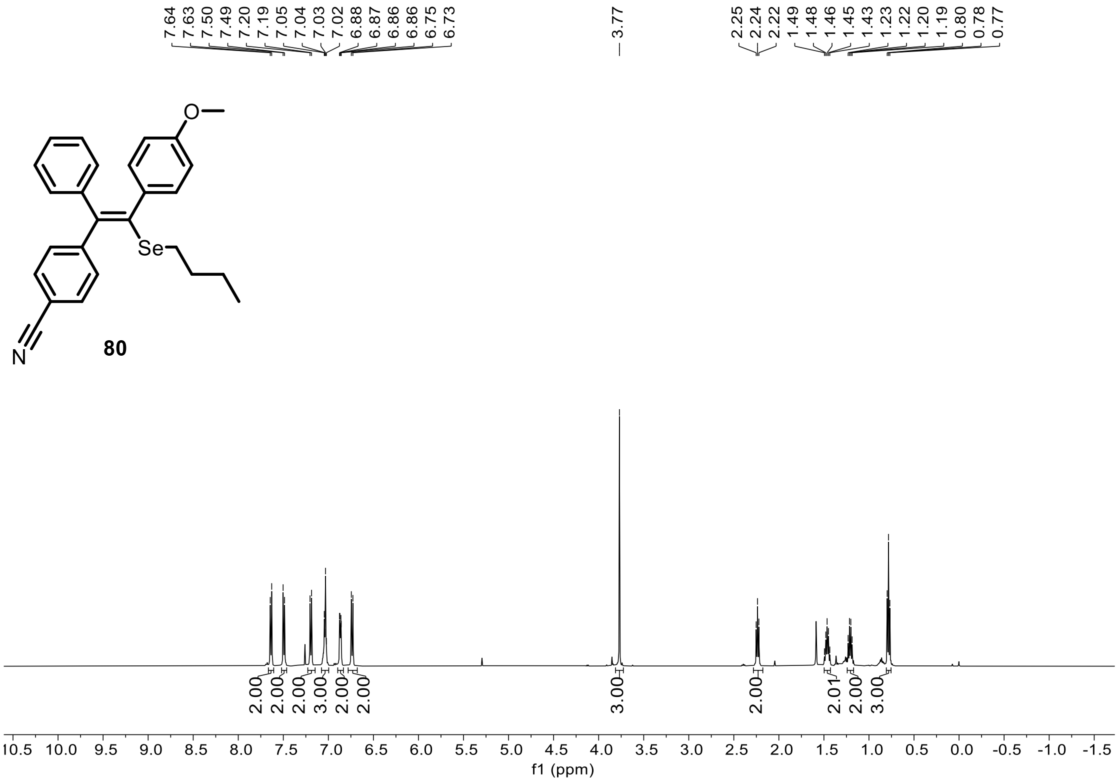

126 MHz, 298 K, $CDCl_3$ as solvent

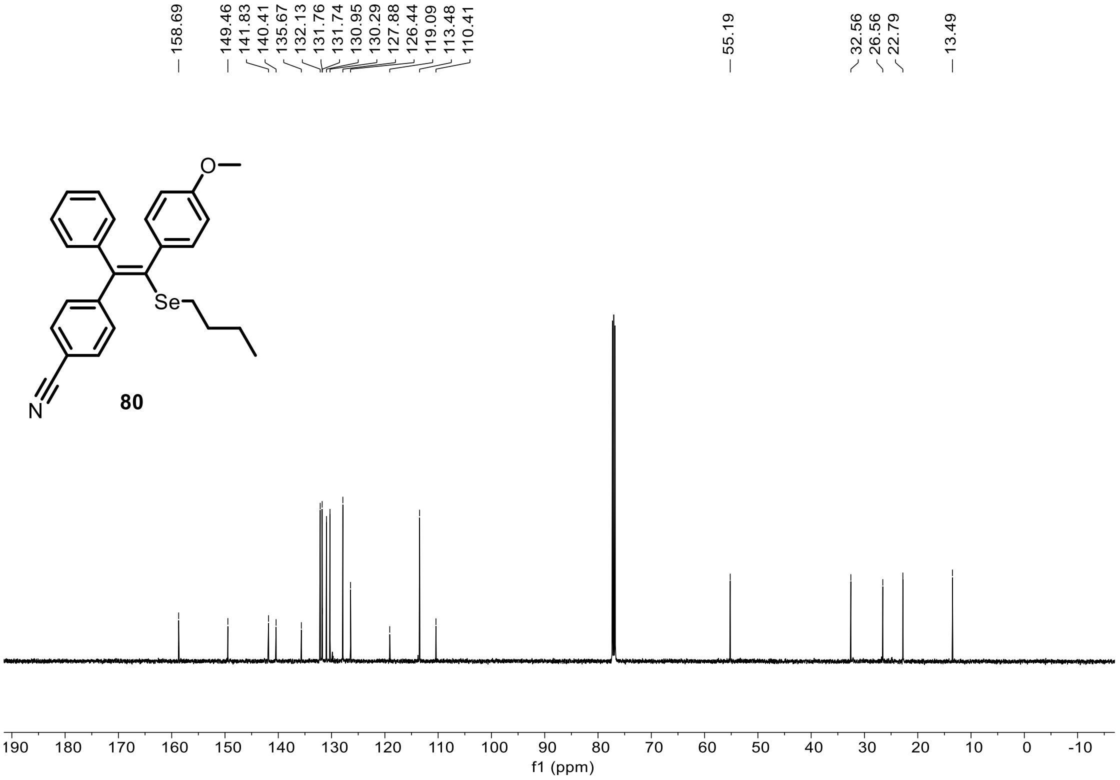

500 MHz, 298 K, CDCl3 as solvent

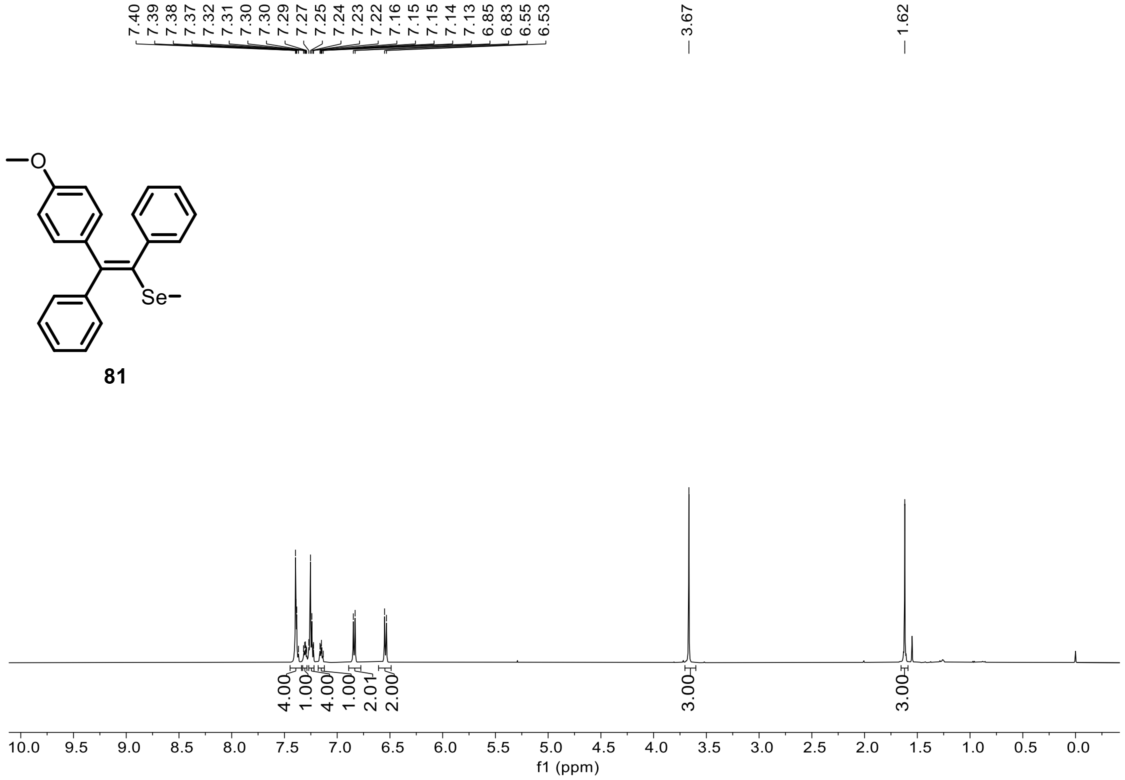

126 MHz, 298 K, CDCl3 as solvent

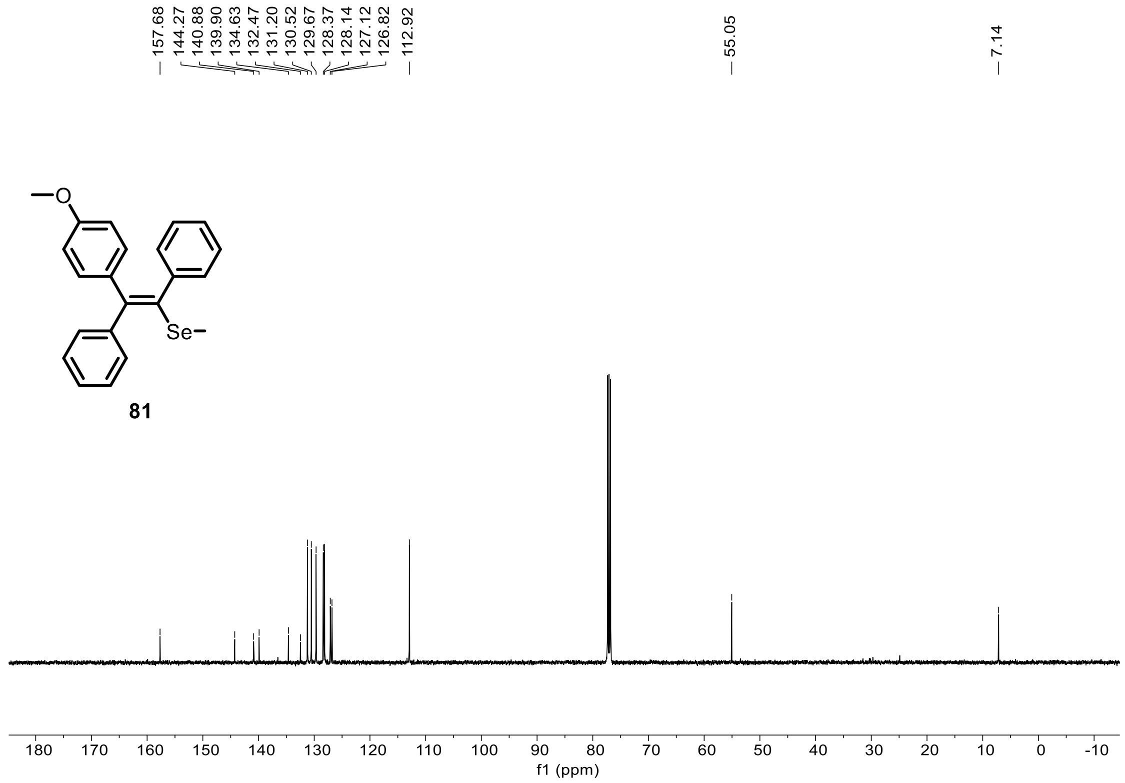

500 MHz, 298 K, $CDCl_3$ as solvent

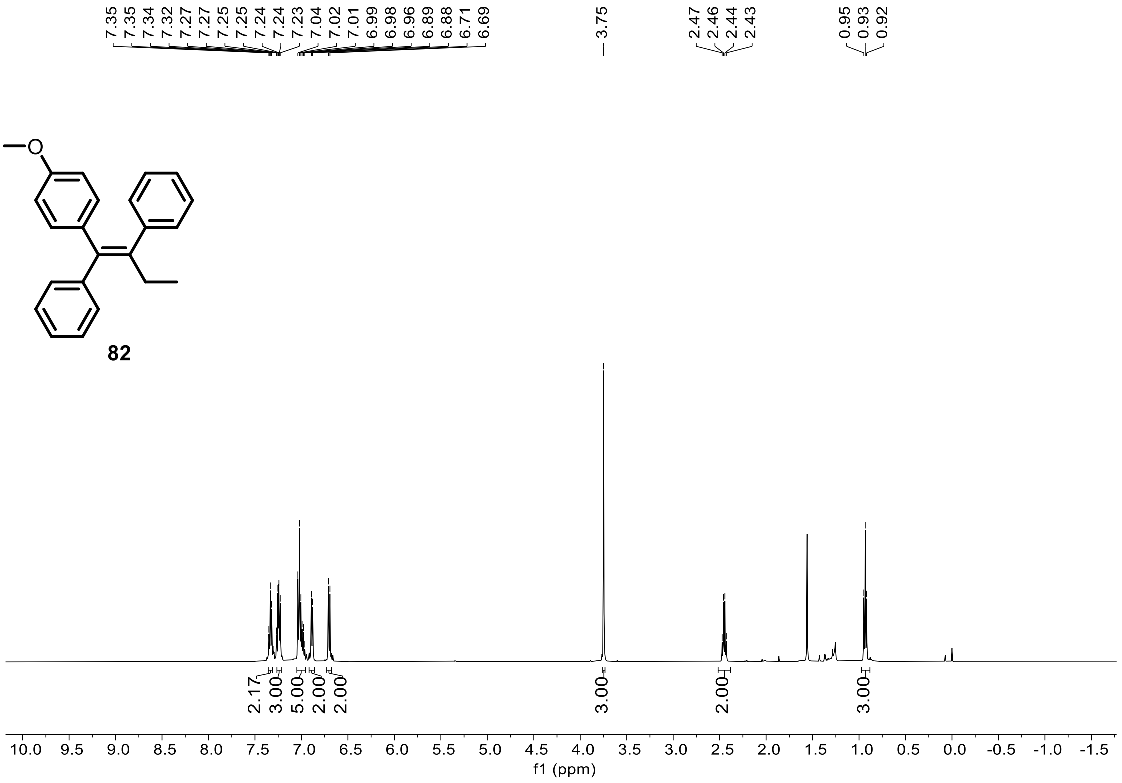

126 MHz, 298 K, $CDCl_3$ as solvent

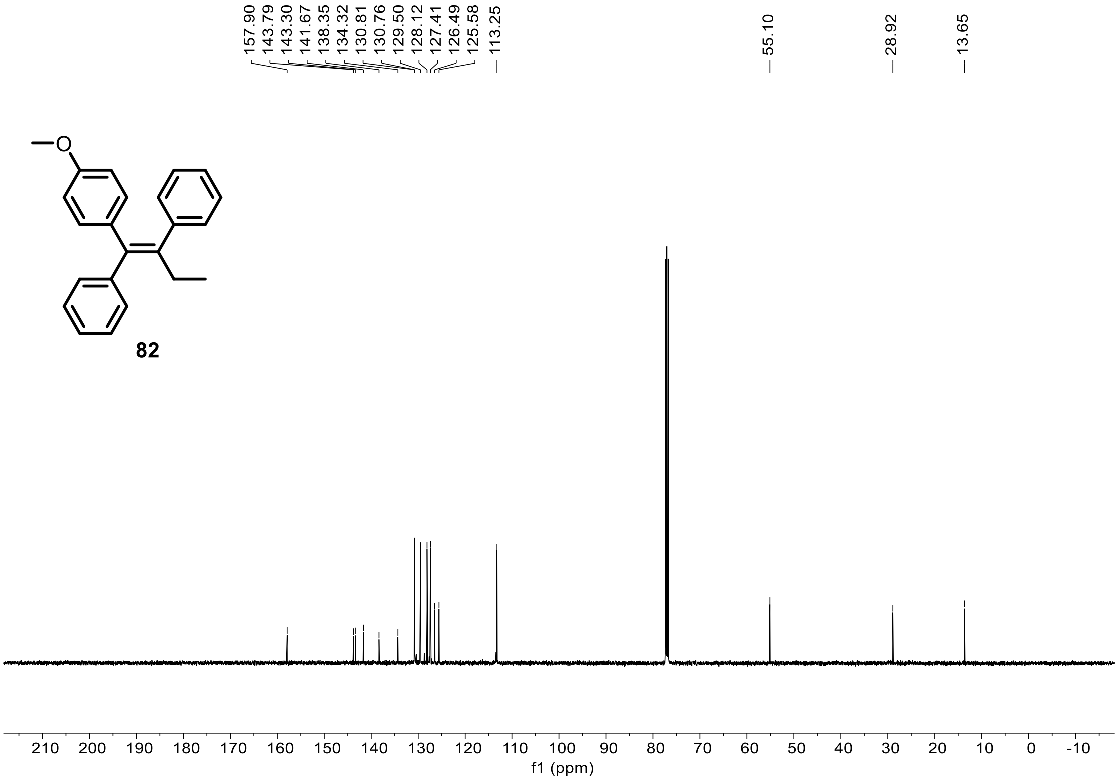

500 MHz, 298 K, $CDCl_3$ as solvent

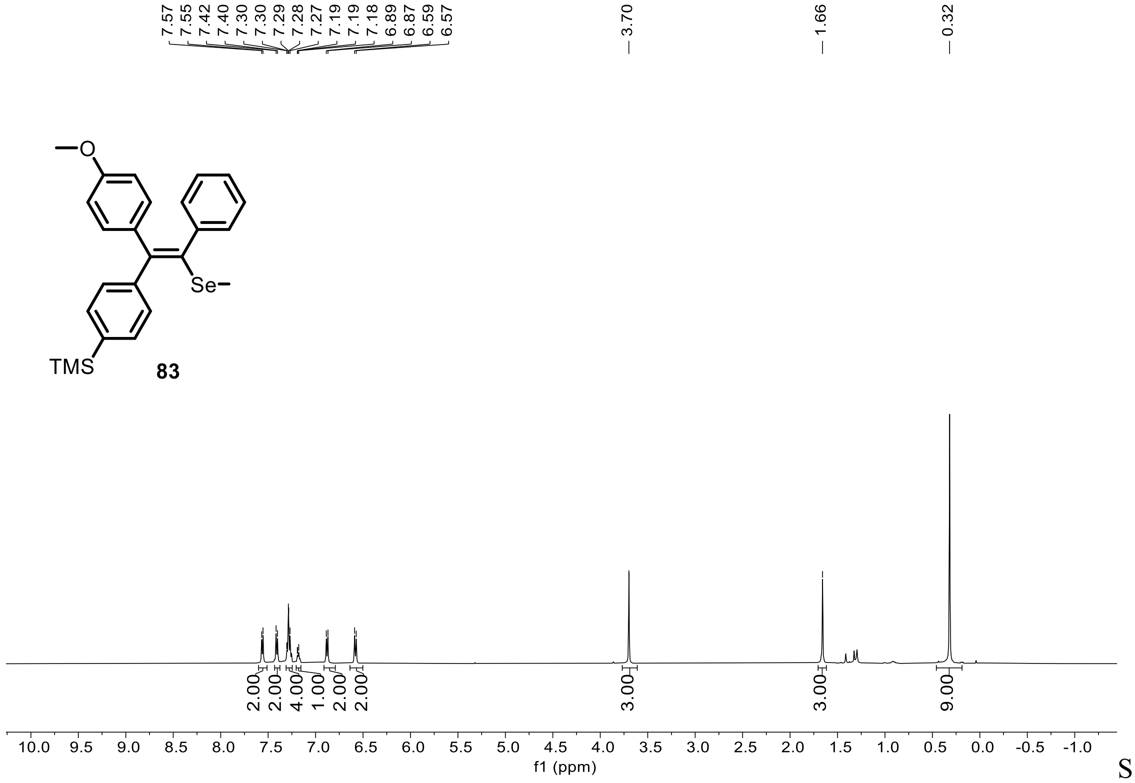

126 MHz, 298 K, $CDCl_3$ as solvent

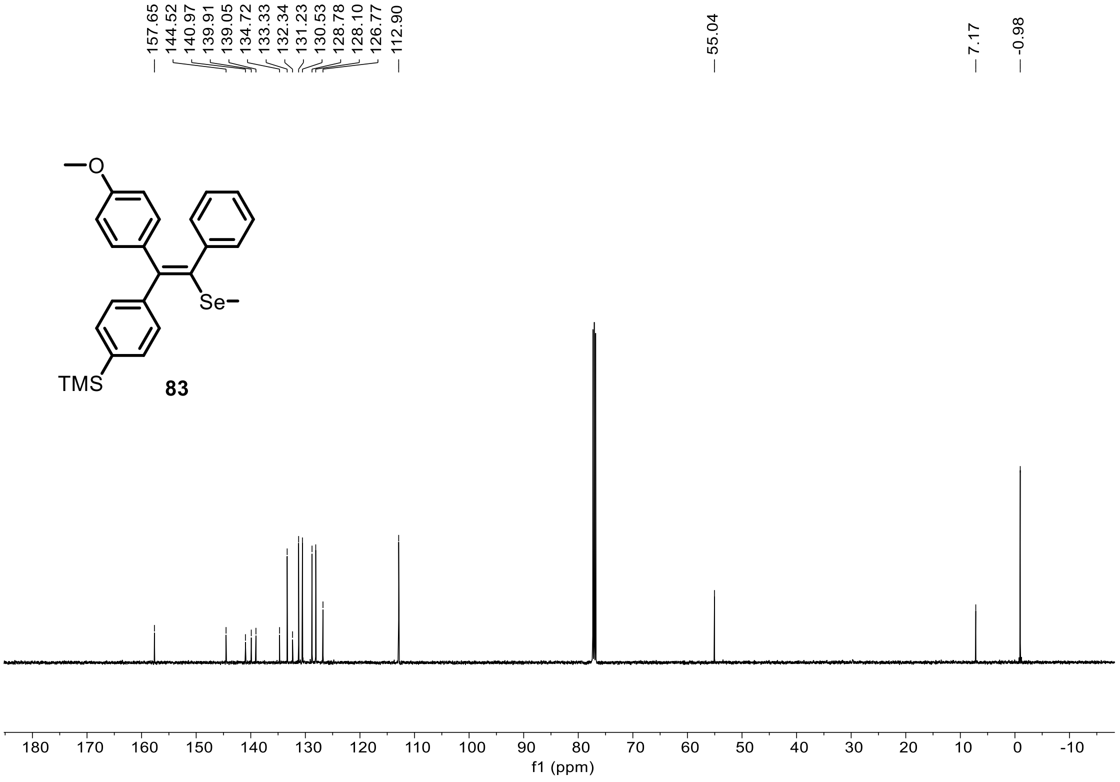

500 MHz, 298 K, $CDCl_3$ as solvent

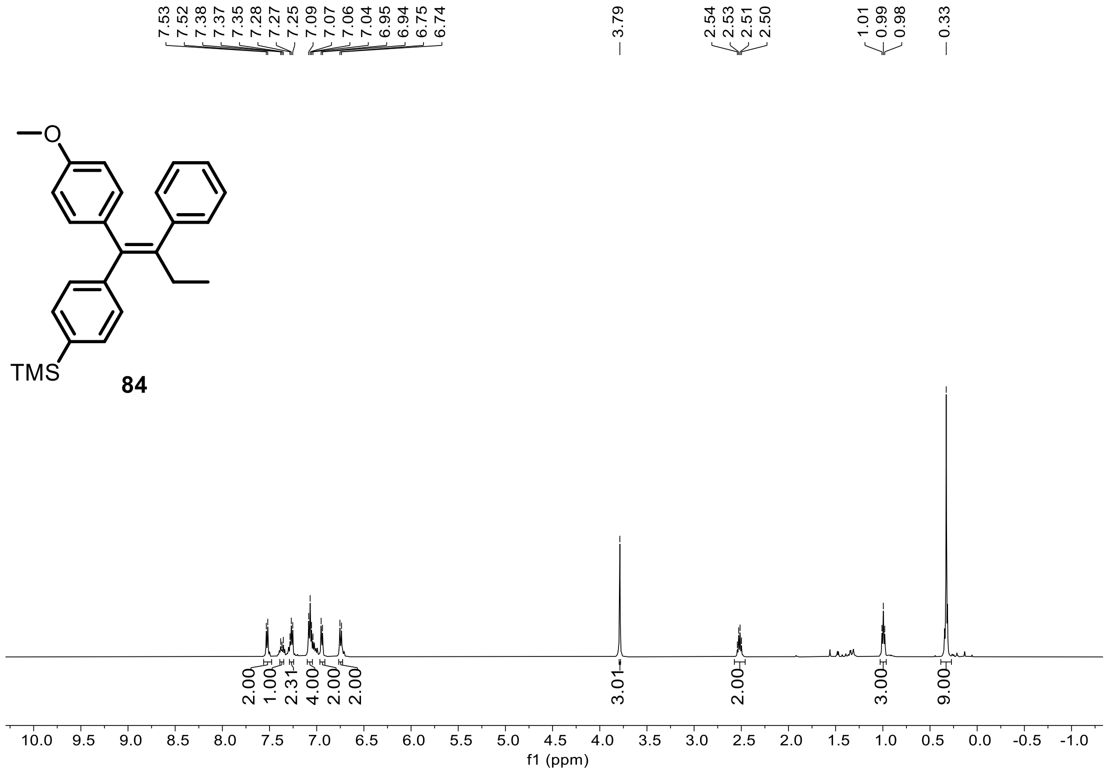

84

126 MHz, 298 K, $CDCl_3$ as solvent

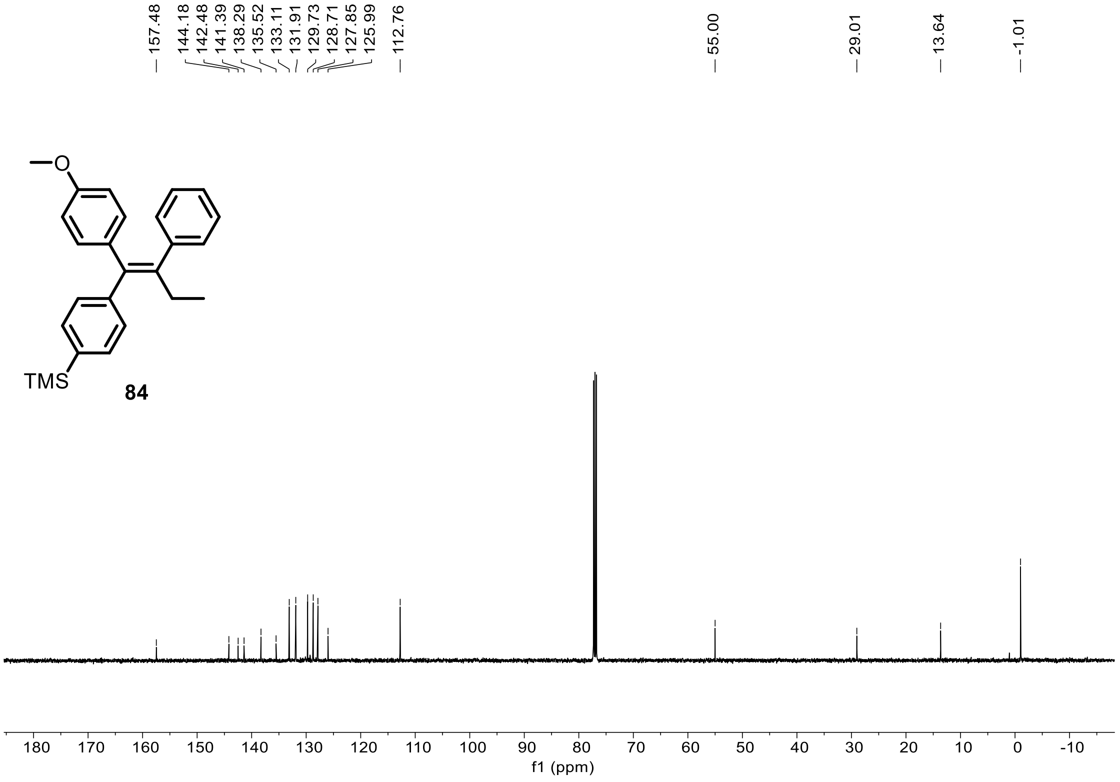

84

500 MHz, 298 K, $CDCl_3$ as solvent

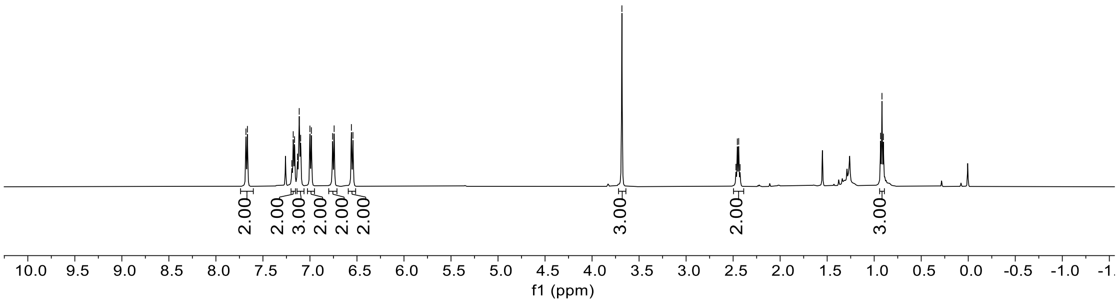

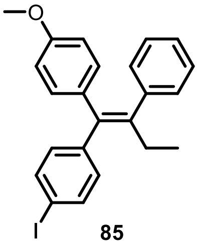

85

126 MHz, 298 K, $CDCl_3$ as solvent

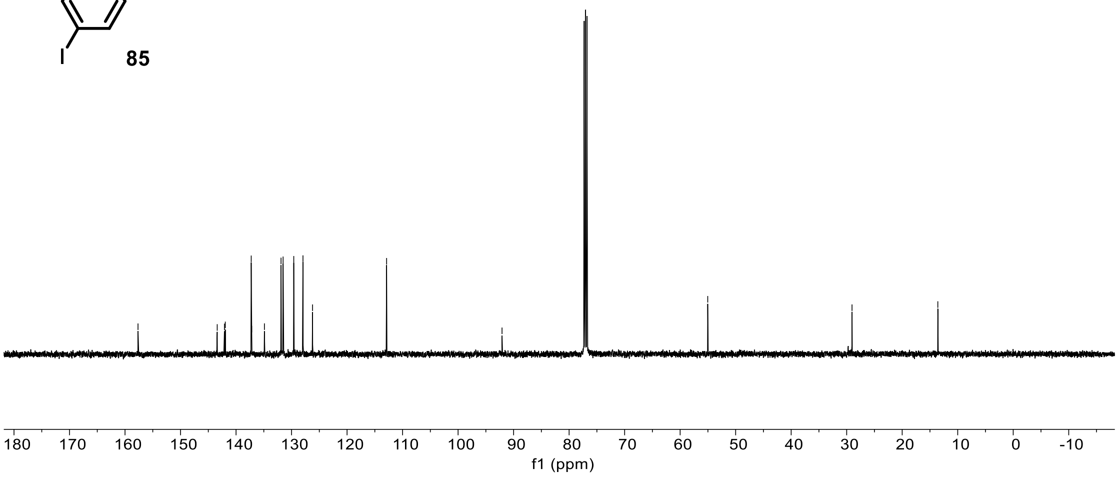

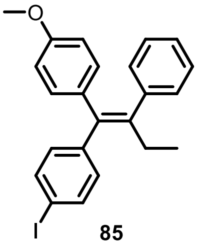

85

500 MHz, 298 K, CD_2Cl_2 as solvent

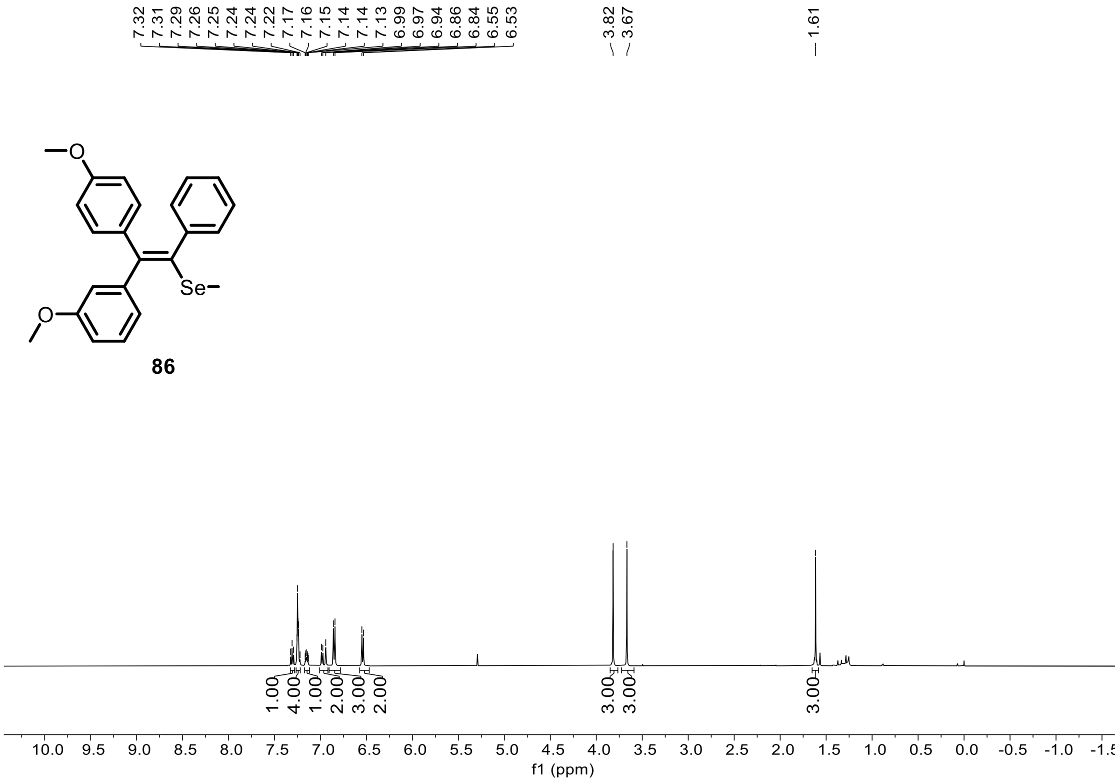

126 MHz, 298 K, $CDCl_3$ as solvent

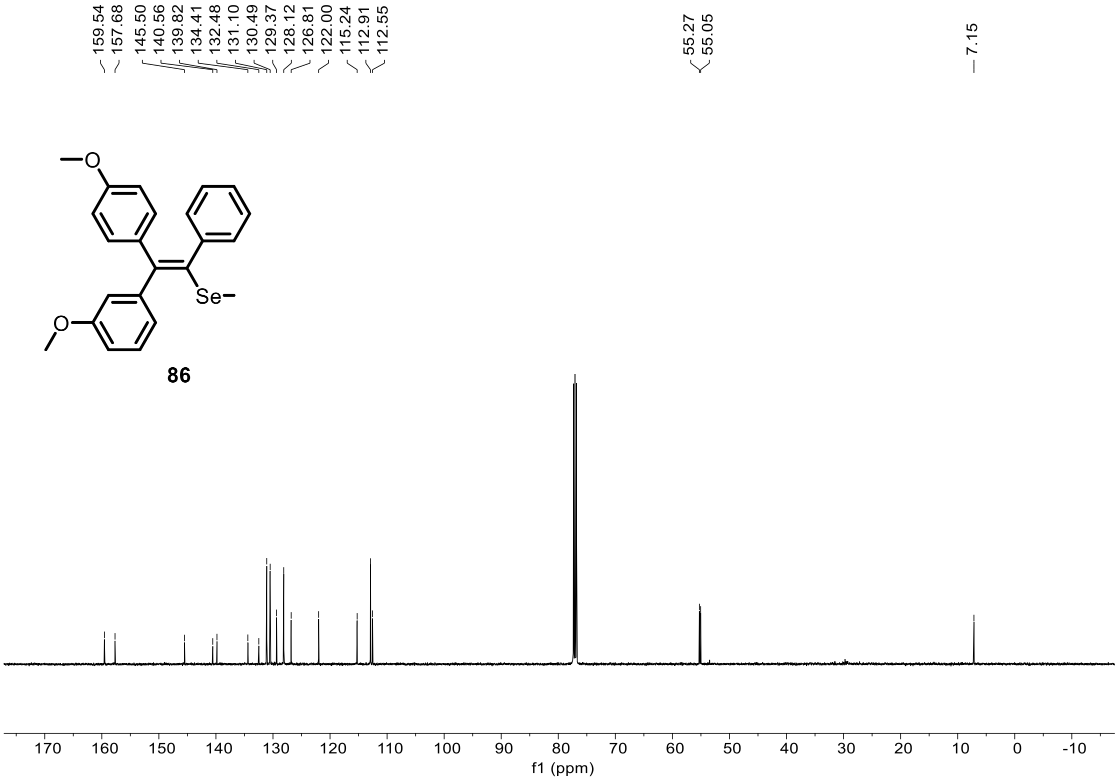

500 MHz, 298 K, $CDCl_3$ as solvent

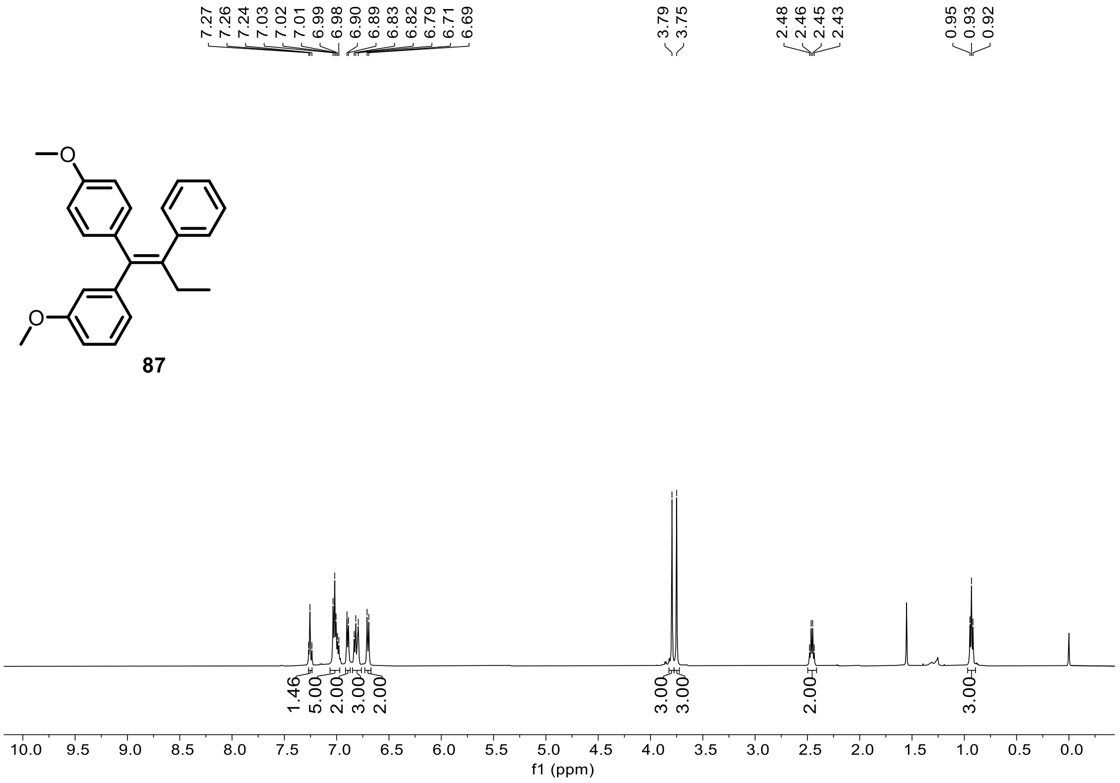

126 MHz, 298 K, $CDCl_3$ as solvent

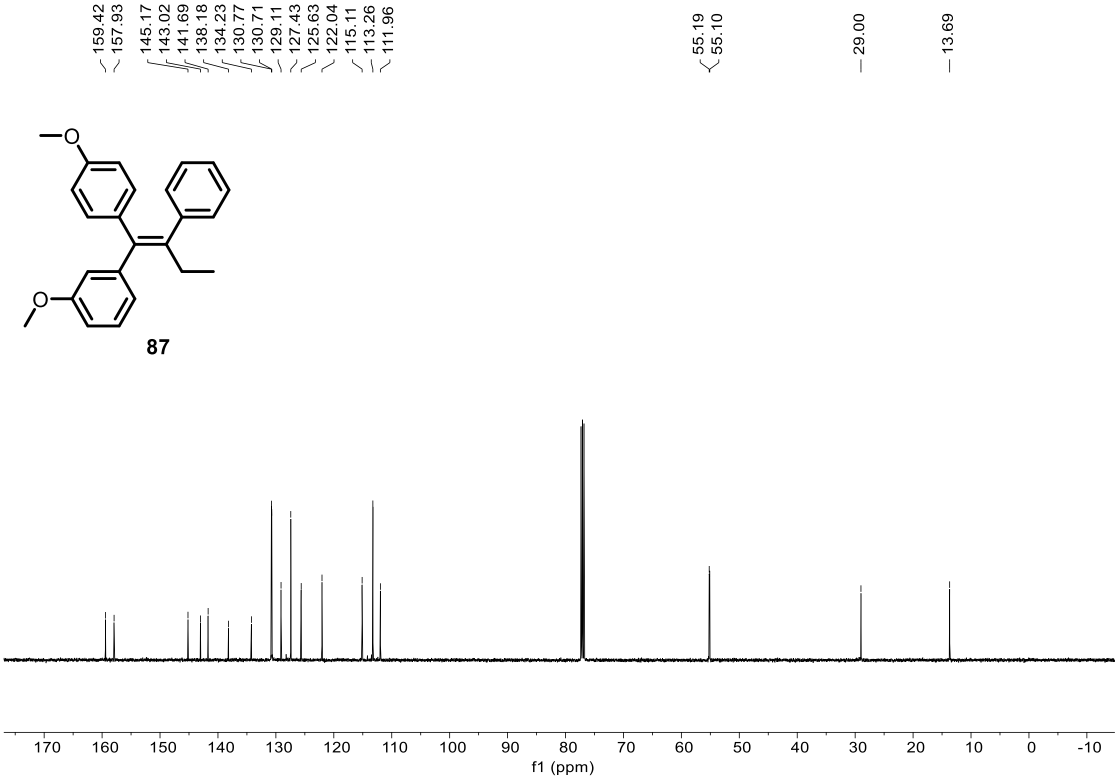

500 MHz, 298 K, $CDCl_3$ as solvent

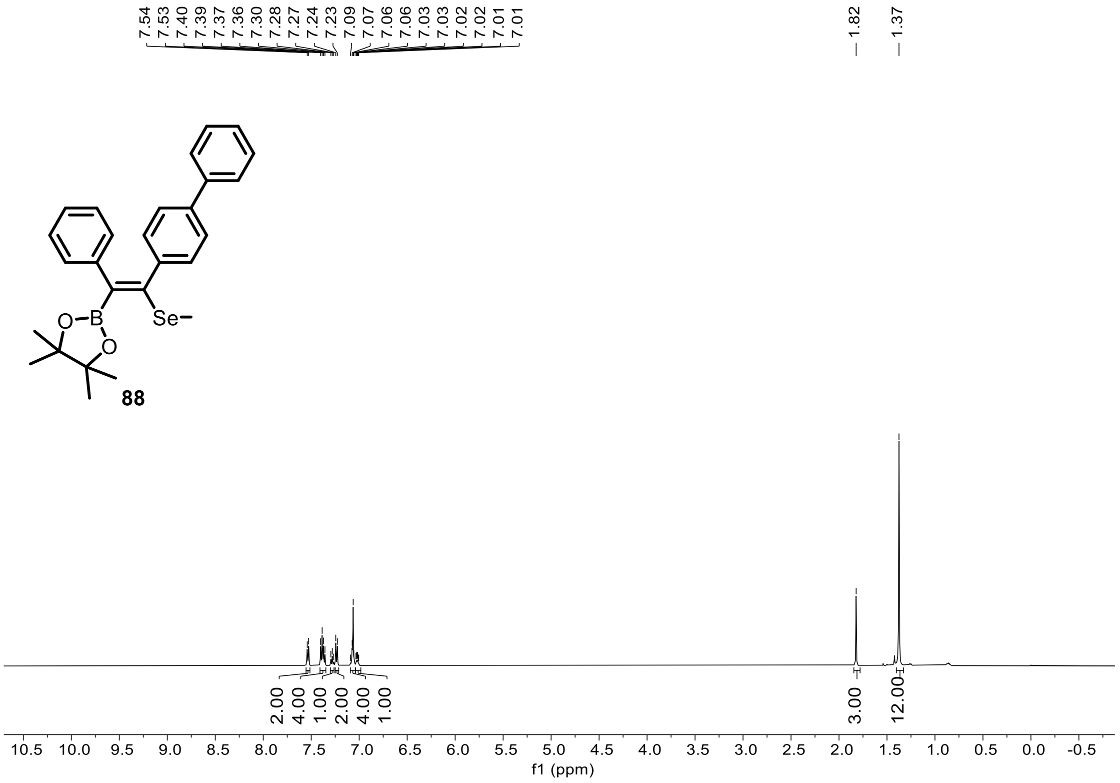

126 MHz, 298 K, $CDCl_3$ as solvent

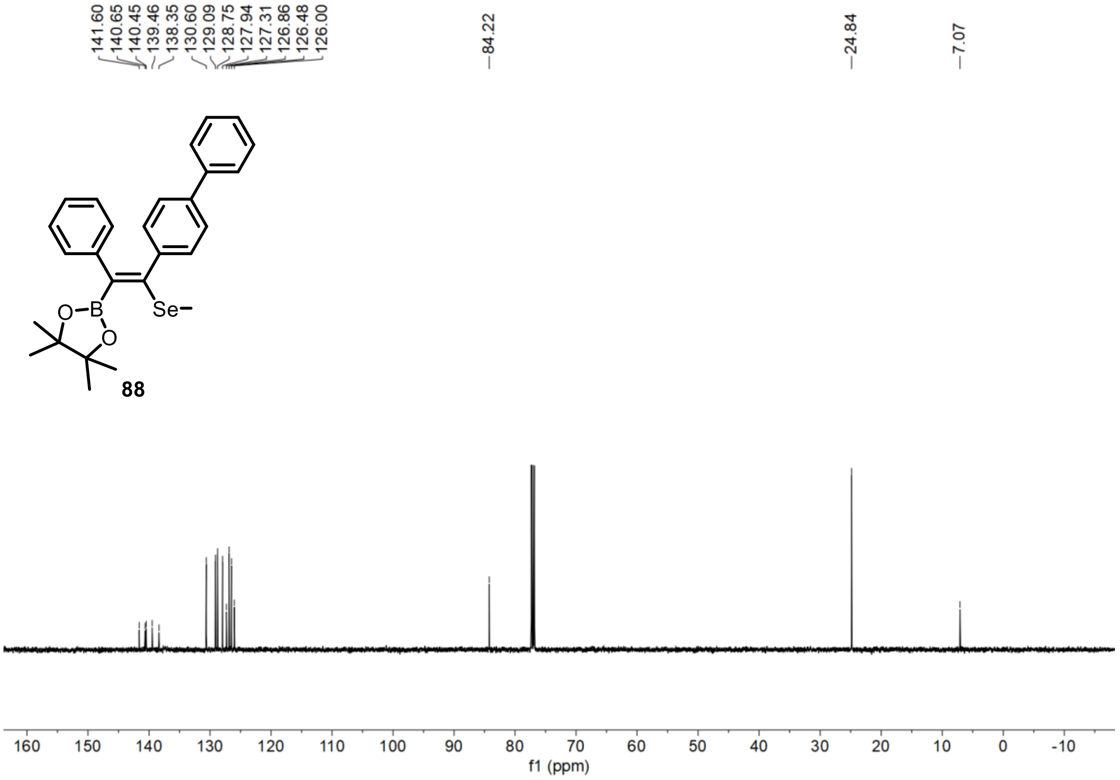

500 MHz, 298 K, $CDCl_3$ as solvent

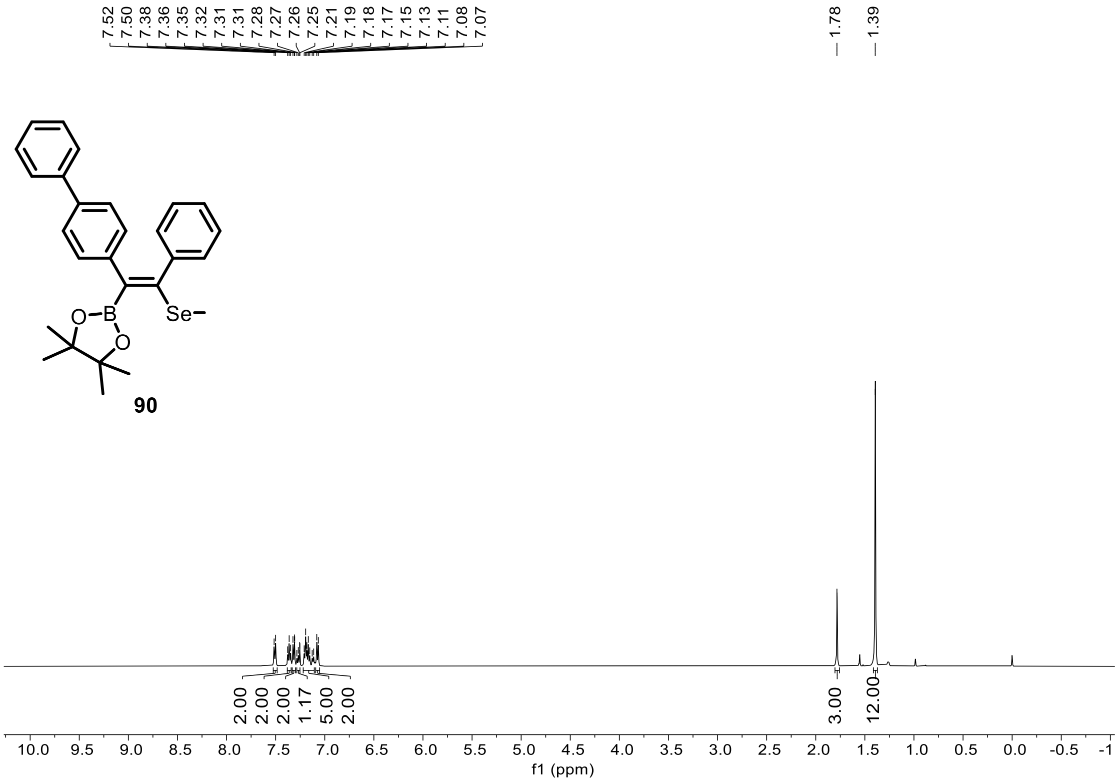

126 MHz, 298 K, $CDCl_3$ as solvent

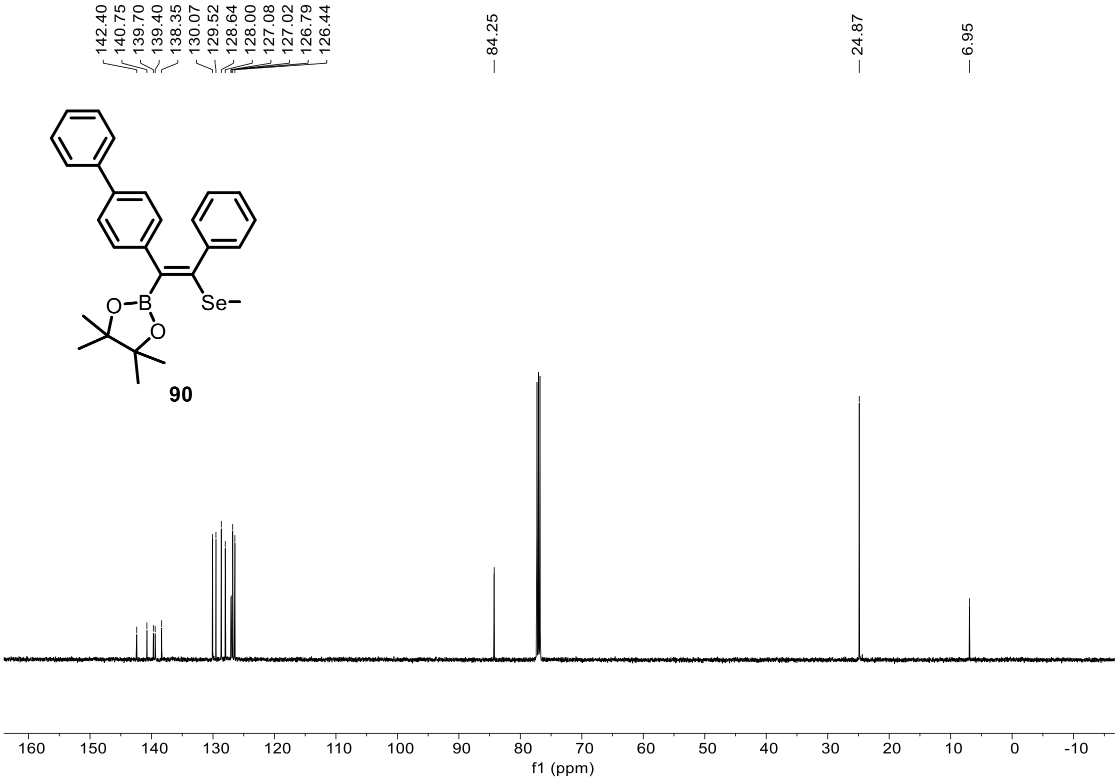

500 MHz, 298 K, $CDCl_3$ as solvent

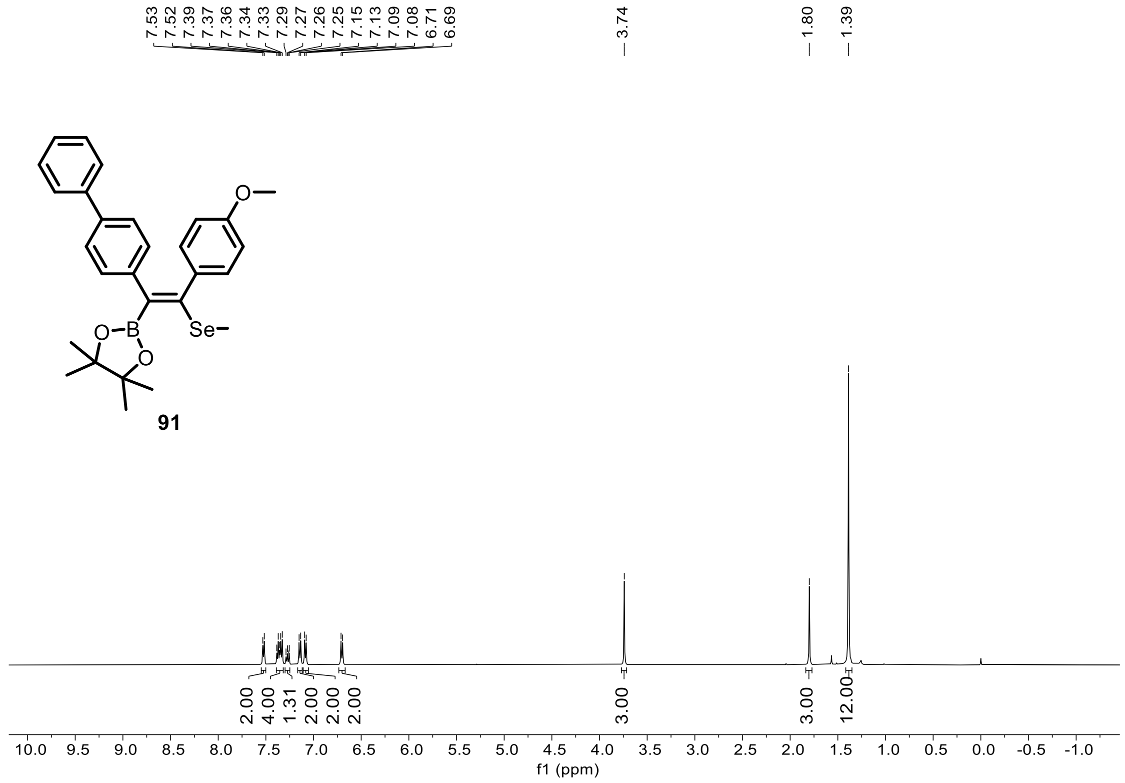

126 MHz, 298 K, $CDCl_3$ as solvent

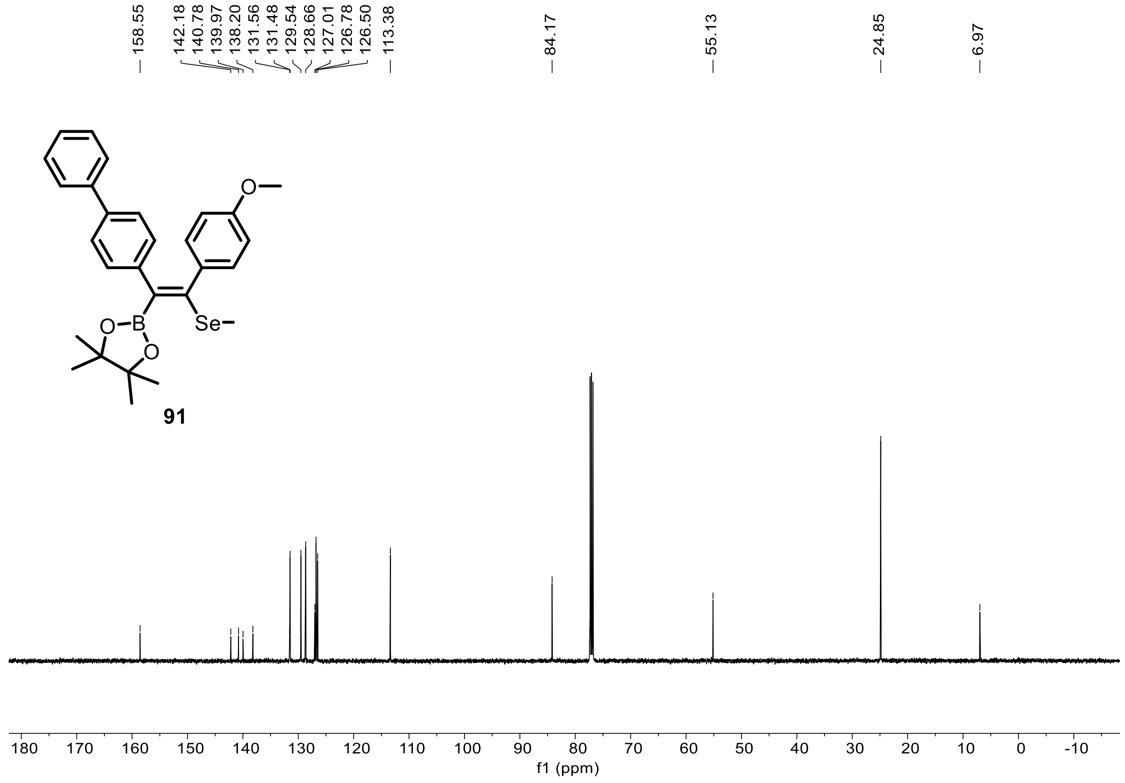

500 MHz, 298 K, CDCl3 as solvent

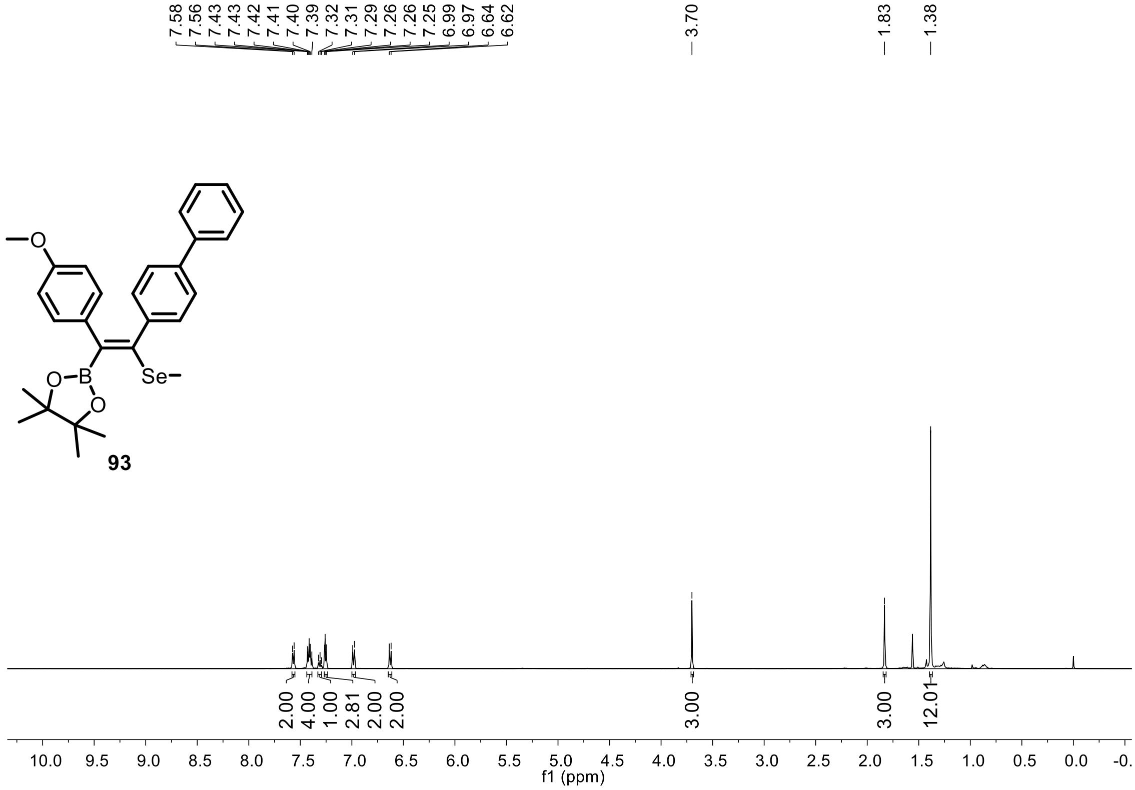

126 MHz, 298 K, CDCl3 as solvent

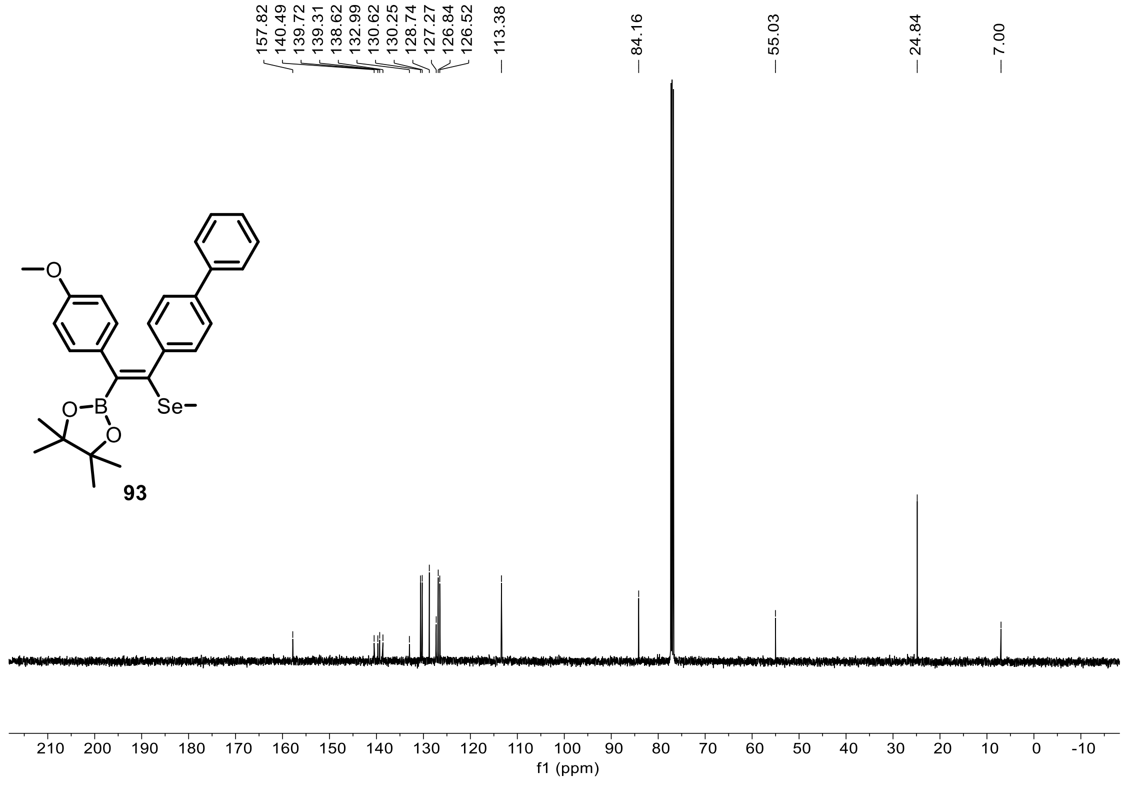

500 MHz, 298 K, Chloroform-*d* as solvent

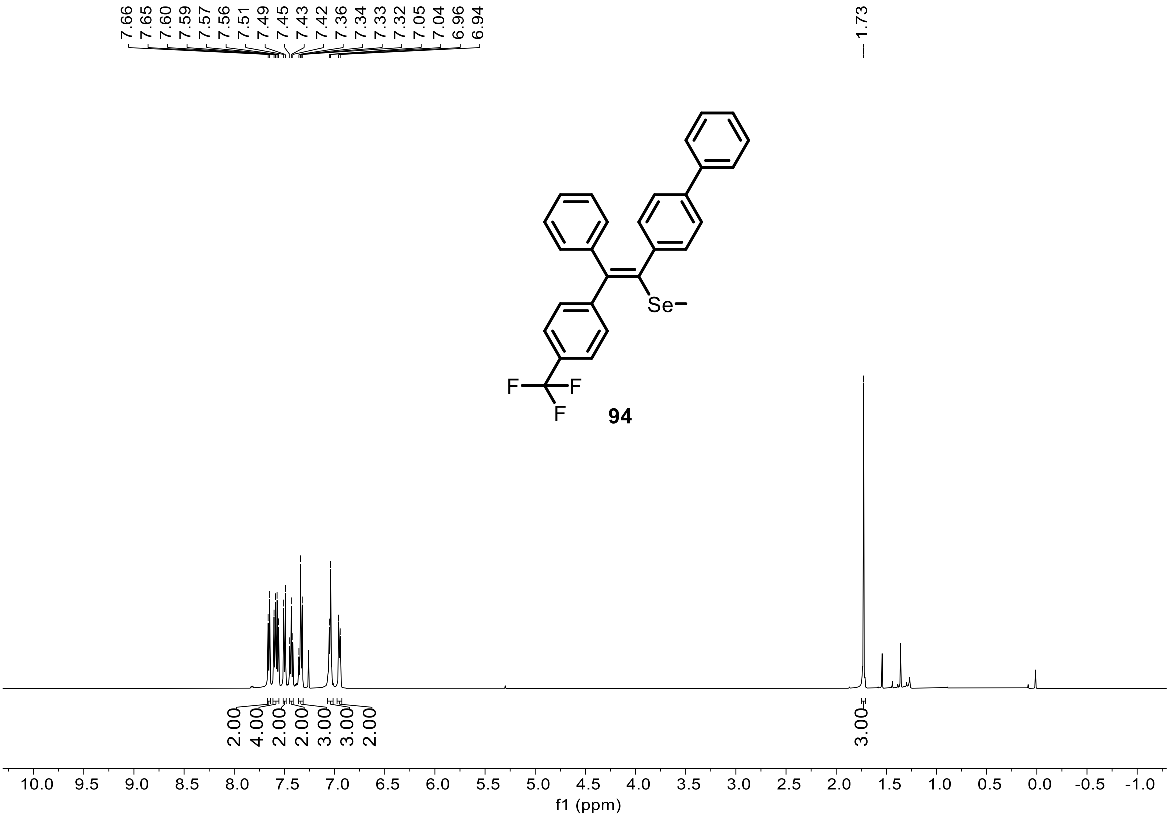

126 MHz, 298 K, $CDCl_3$ as solvent

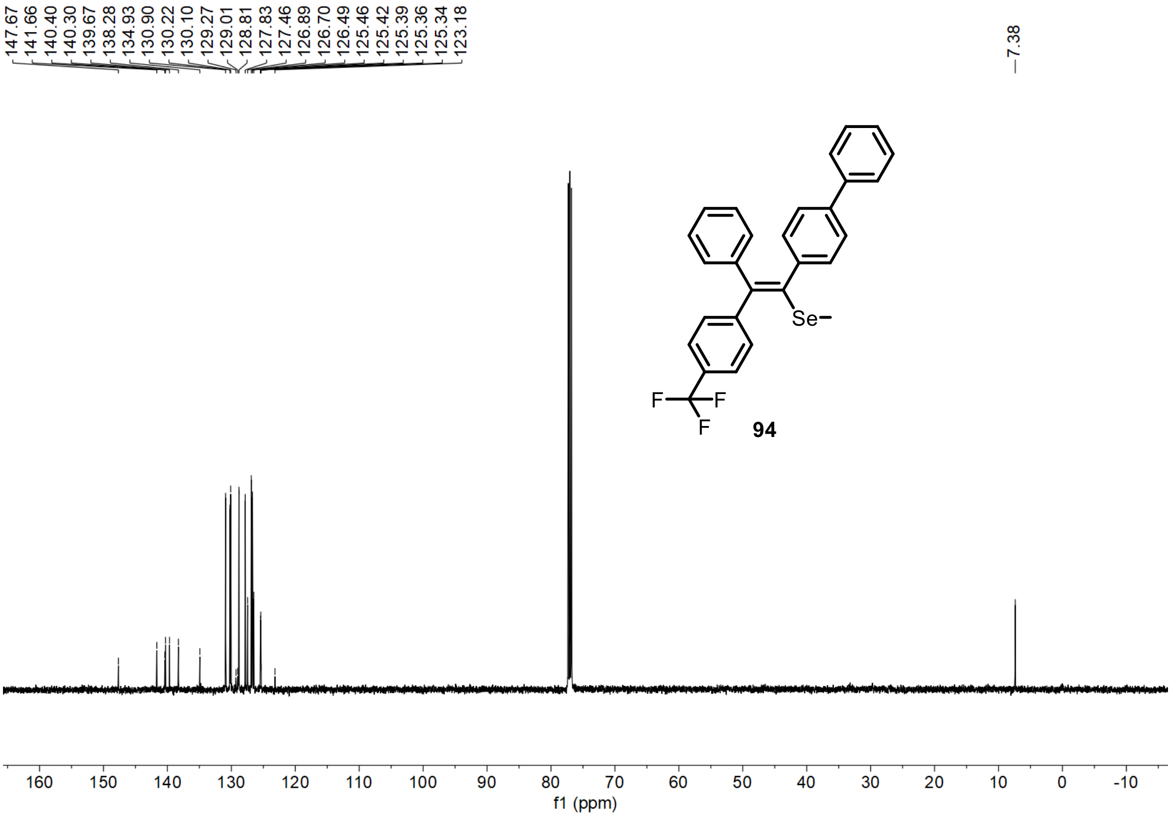

500 MHz, 298 K, $CDCl_3$ as solvent

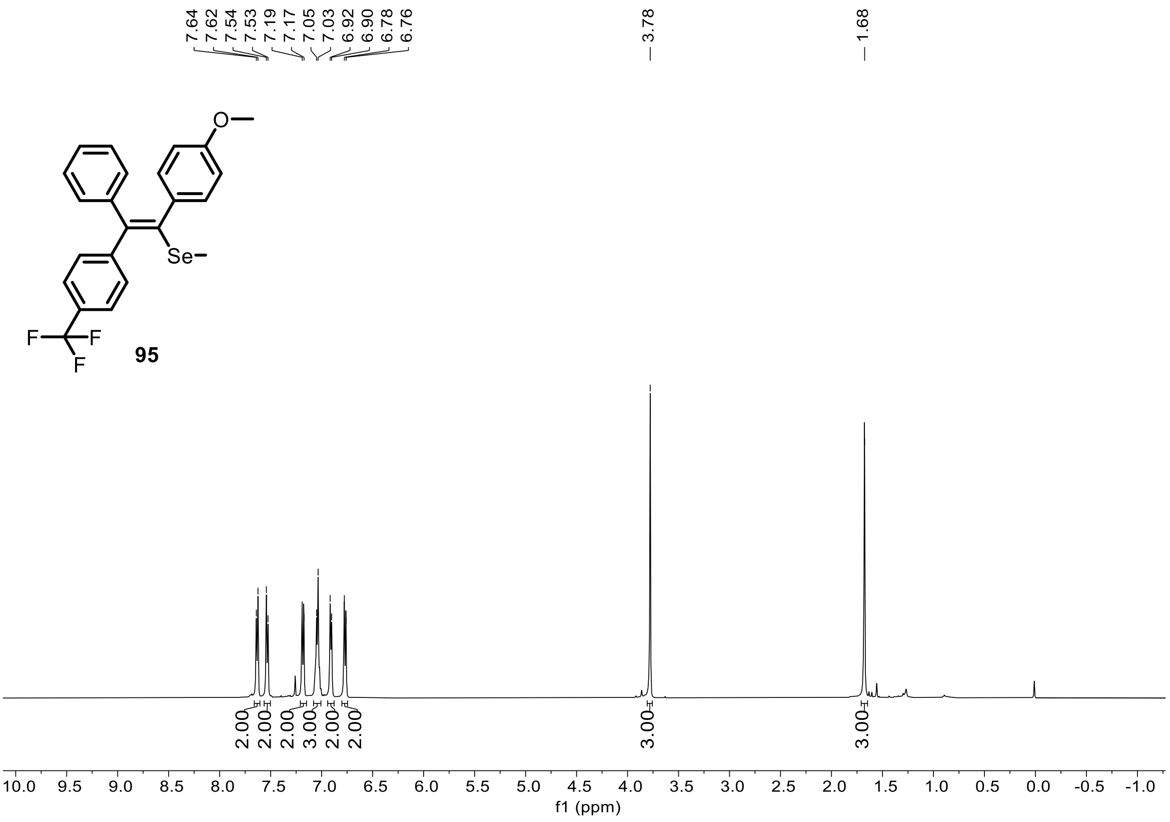

126 MHz, 298 K, $CDCl_3$ as solvent

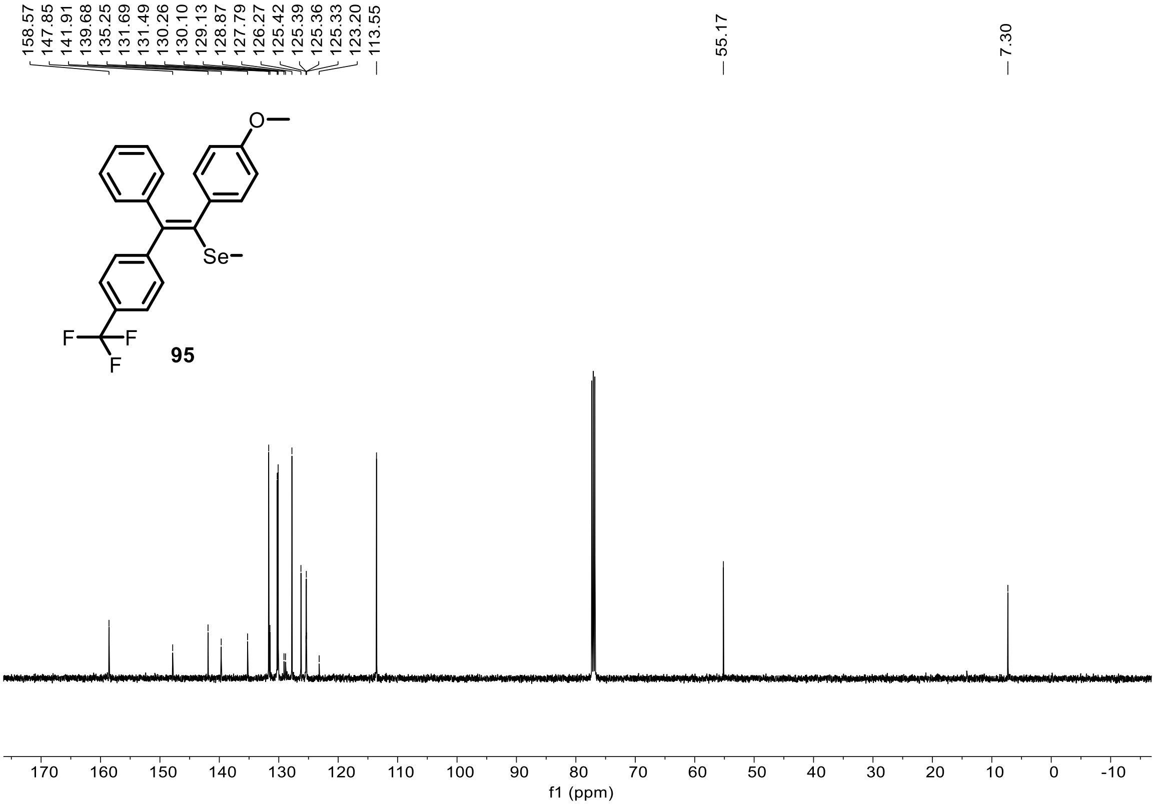

500 MHz, 298 K, $CDCl_3$ as solvent

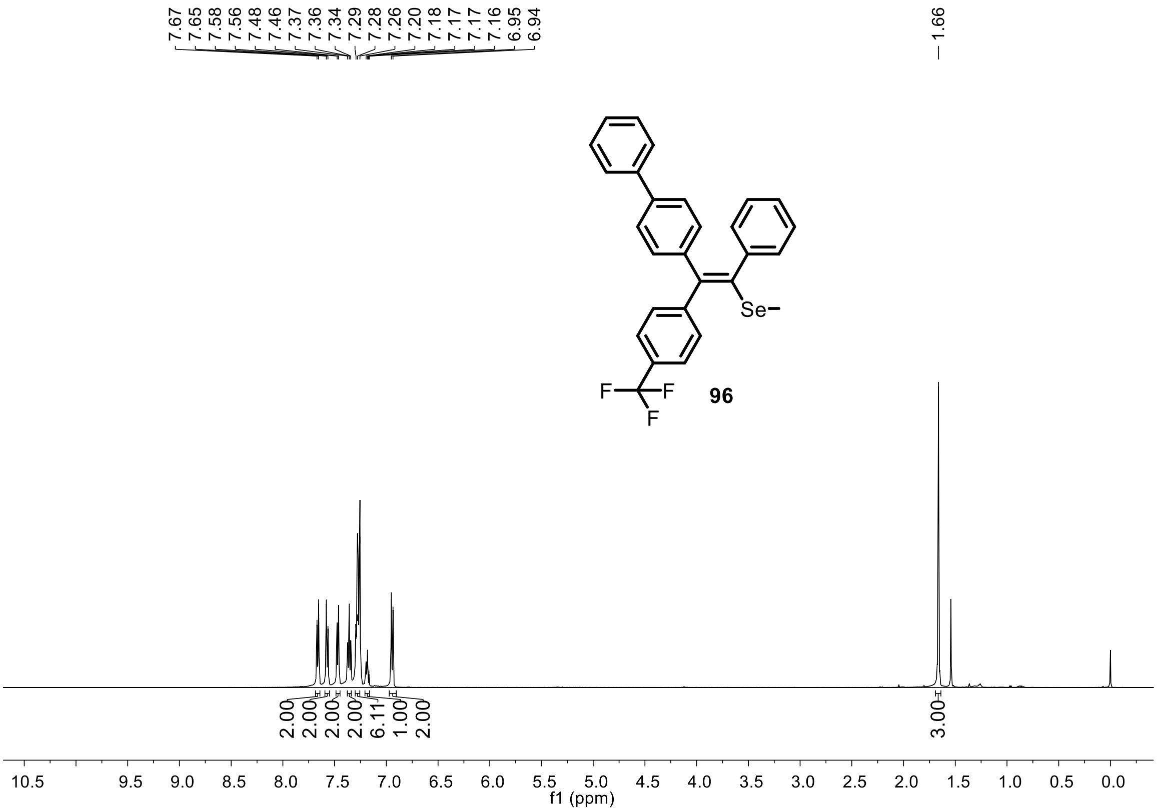

126 MHz, 298 K, $CDCl_3$ as solvent

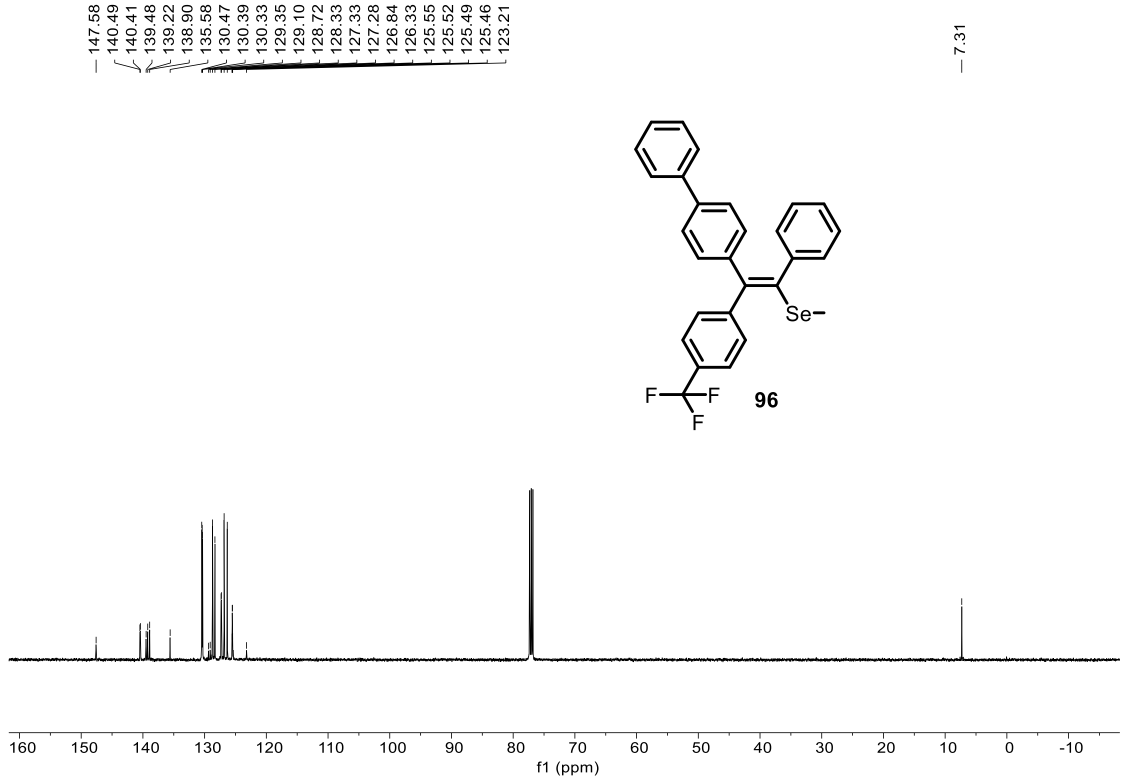

500 MHz, 298 K, $CDCl_3$ as solvent

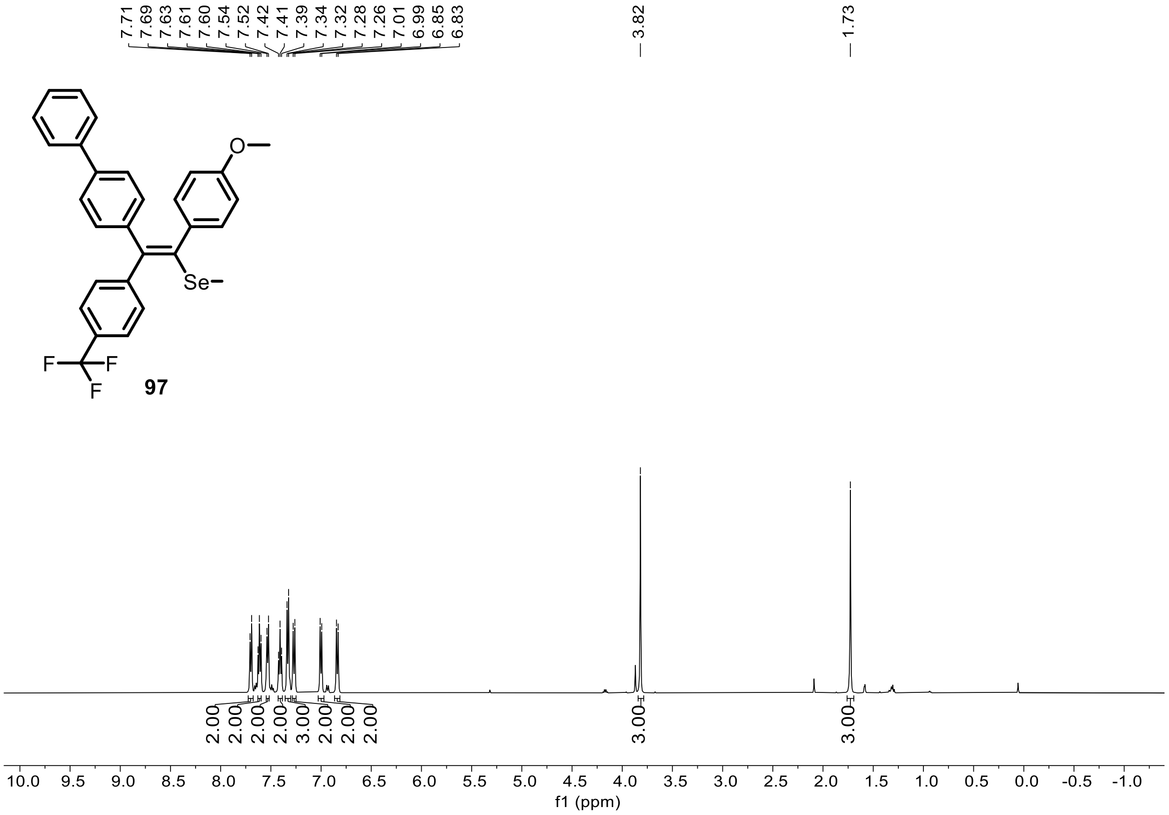

126 MHz, 298 K, CD_2Cl_2 as solvent

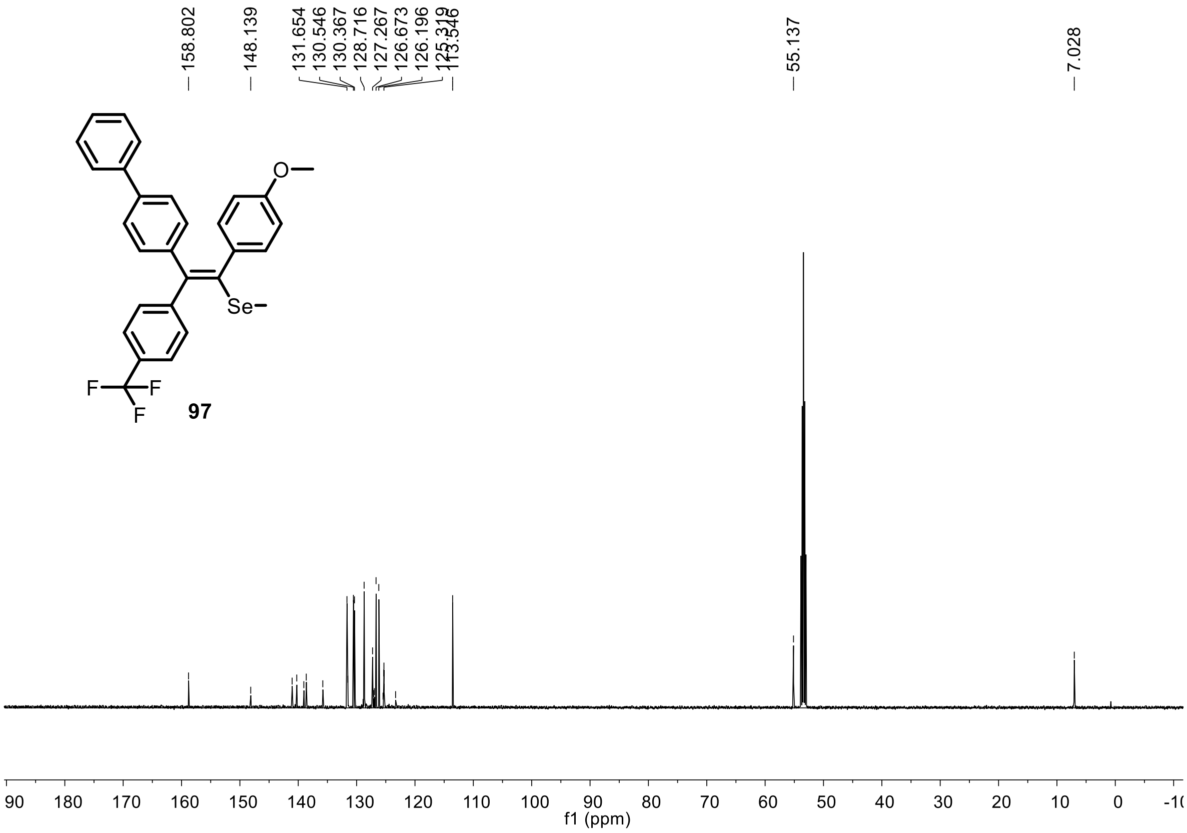

500 MHz, 298 K, $CDCl_3$ as solvent

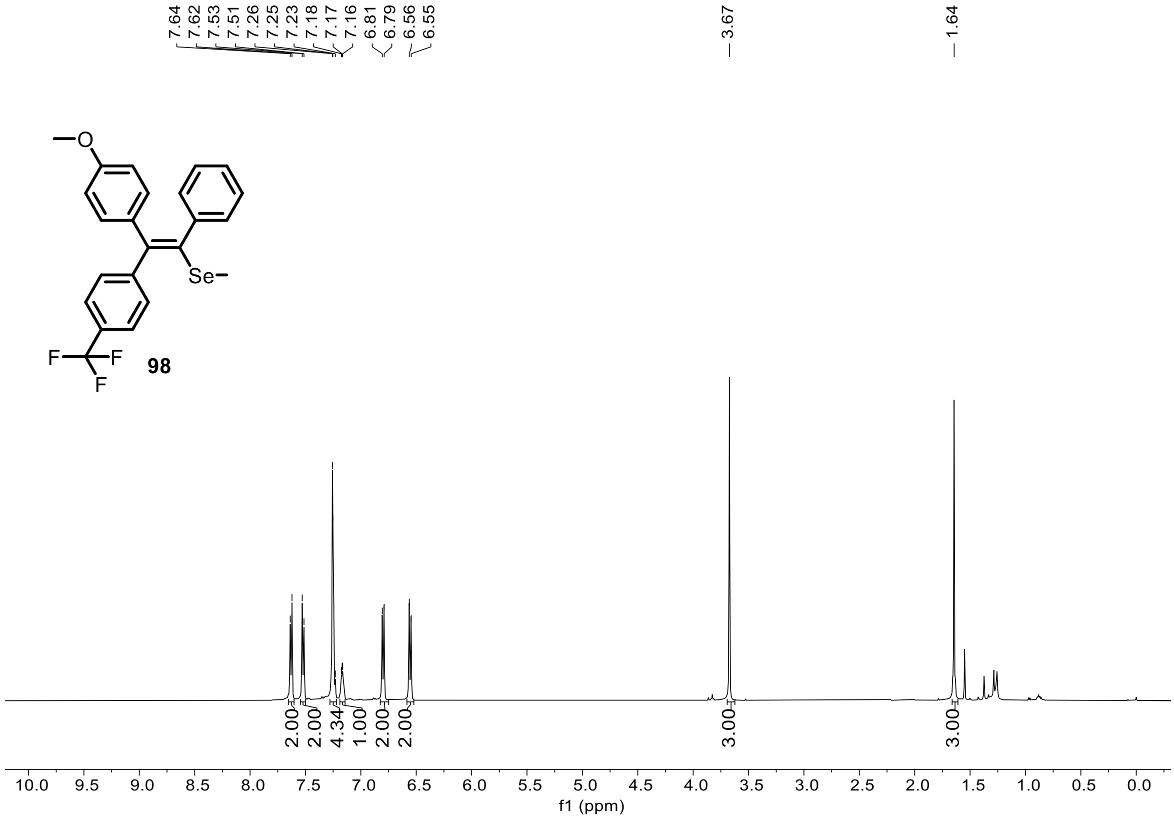

126 MHz, 298 K, $CDCl_3$ as solvent

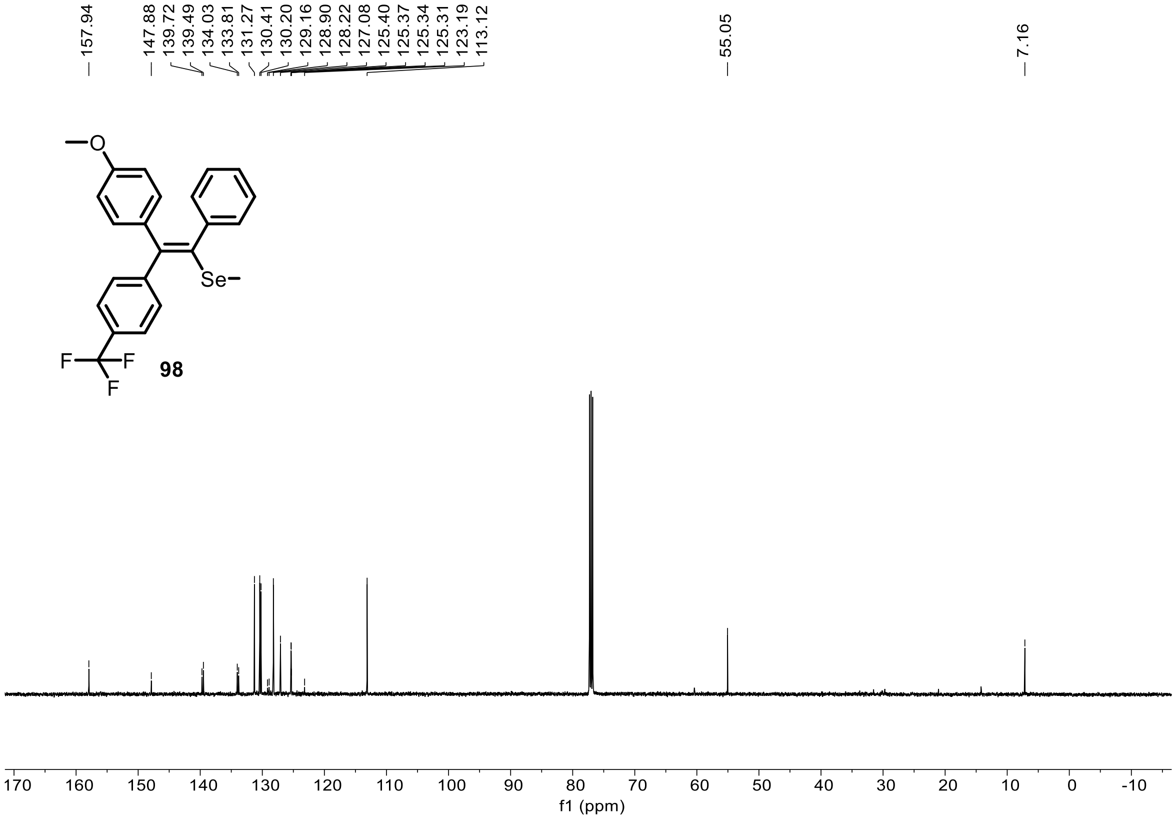

500 MHz, 298 K, CDCl3 as solvent

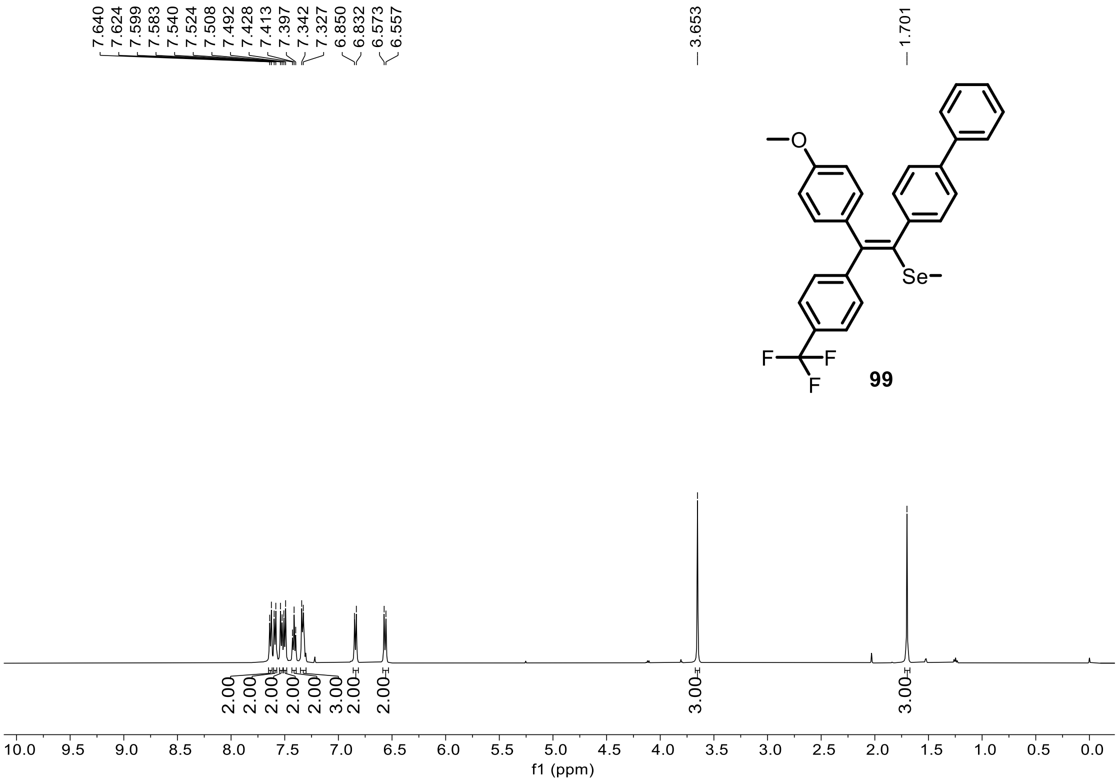

126 MHz, 298 K, CDCl3 as solvent

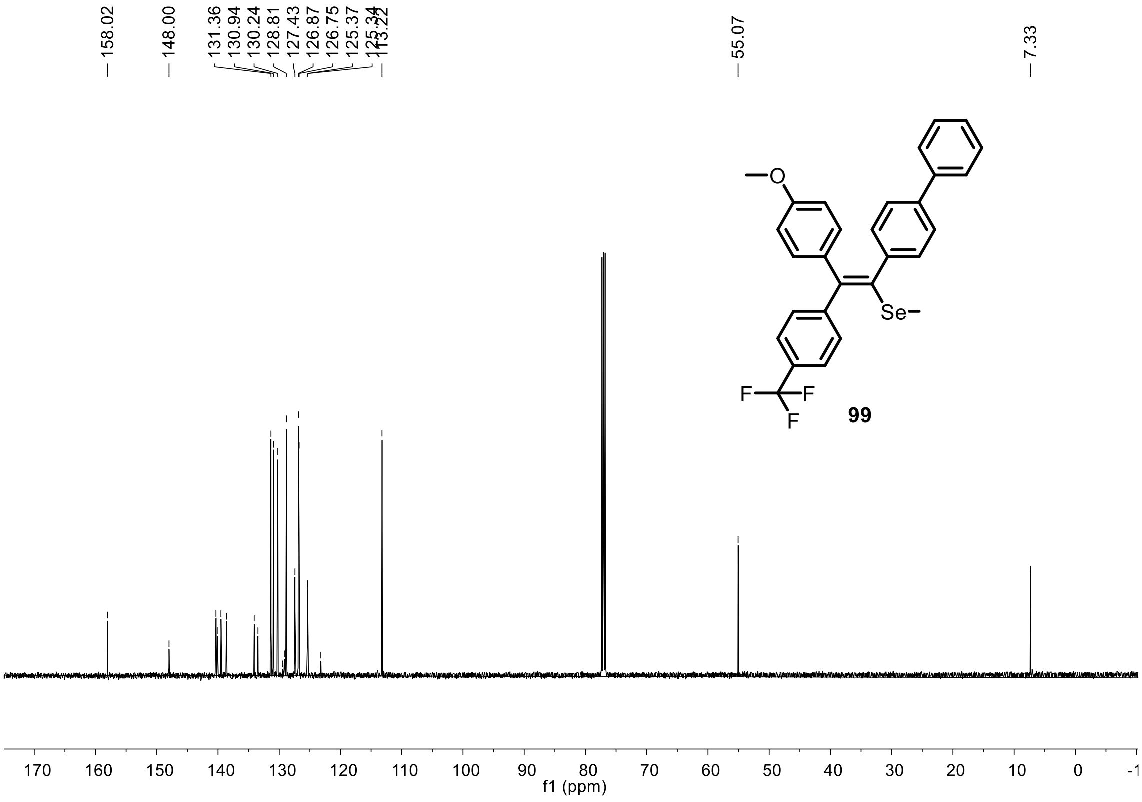

500 MHz, 298 K, $CDCl_3$ as solvent

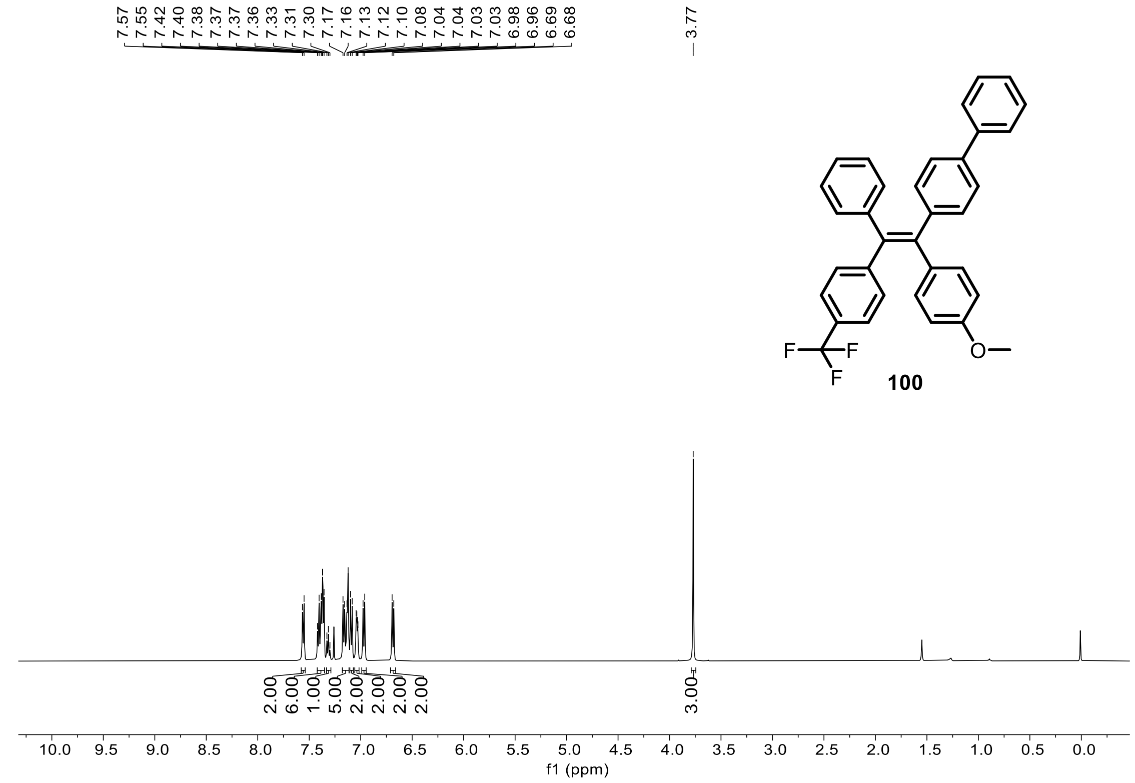

126 MHz, 298 K, $CDCl_3$ as solvent

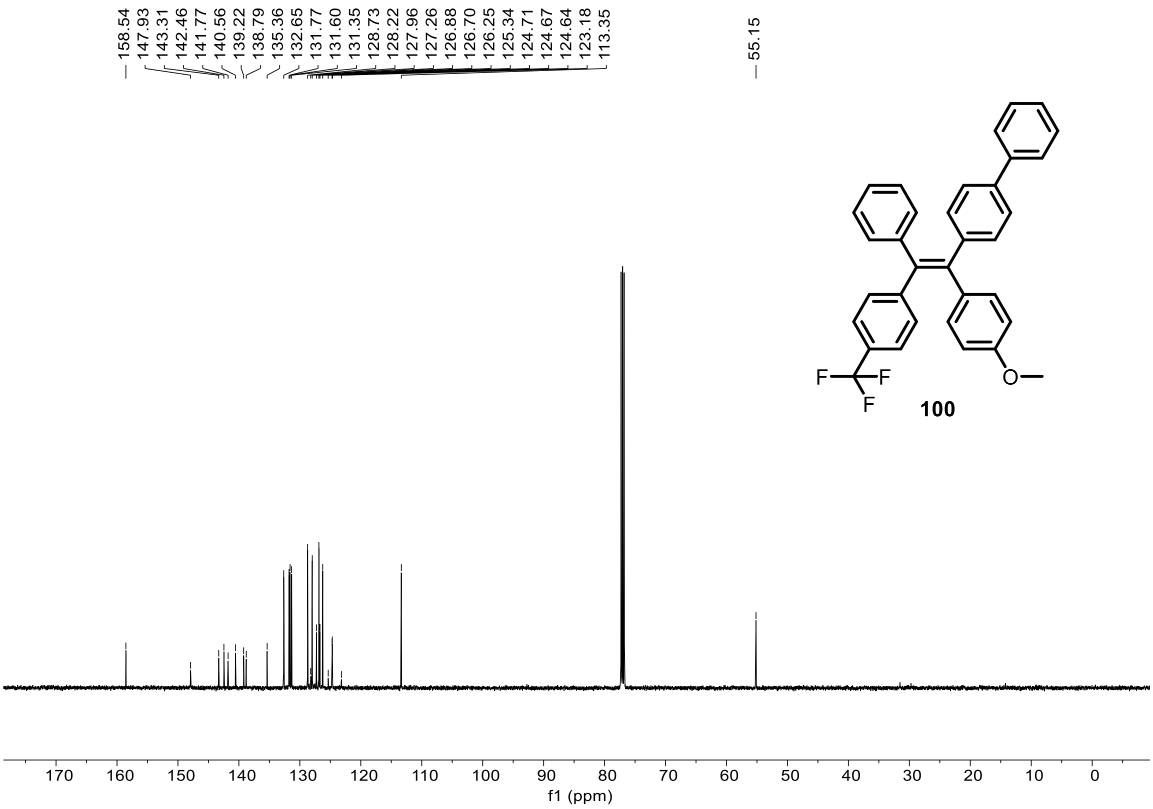

500 MHz, 298 K, CDCl3 as solvent

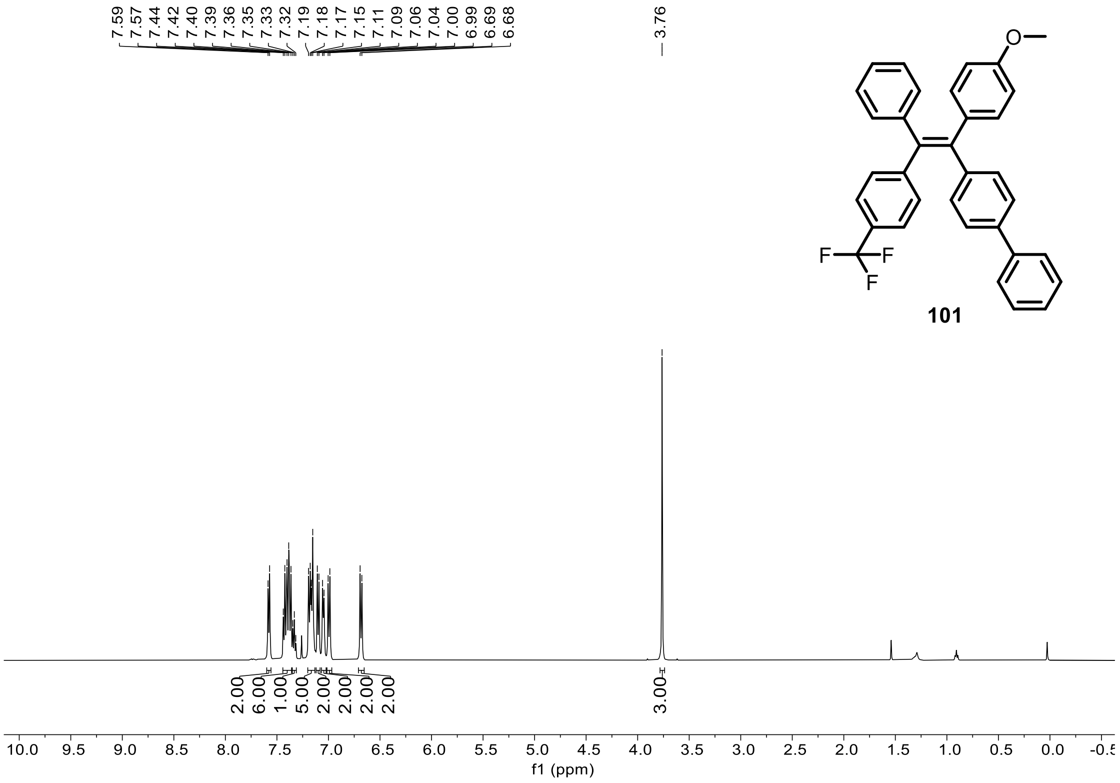

126 MHz, 298 K, CDCl3 as solvent

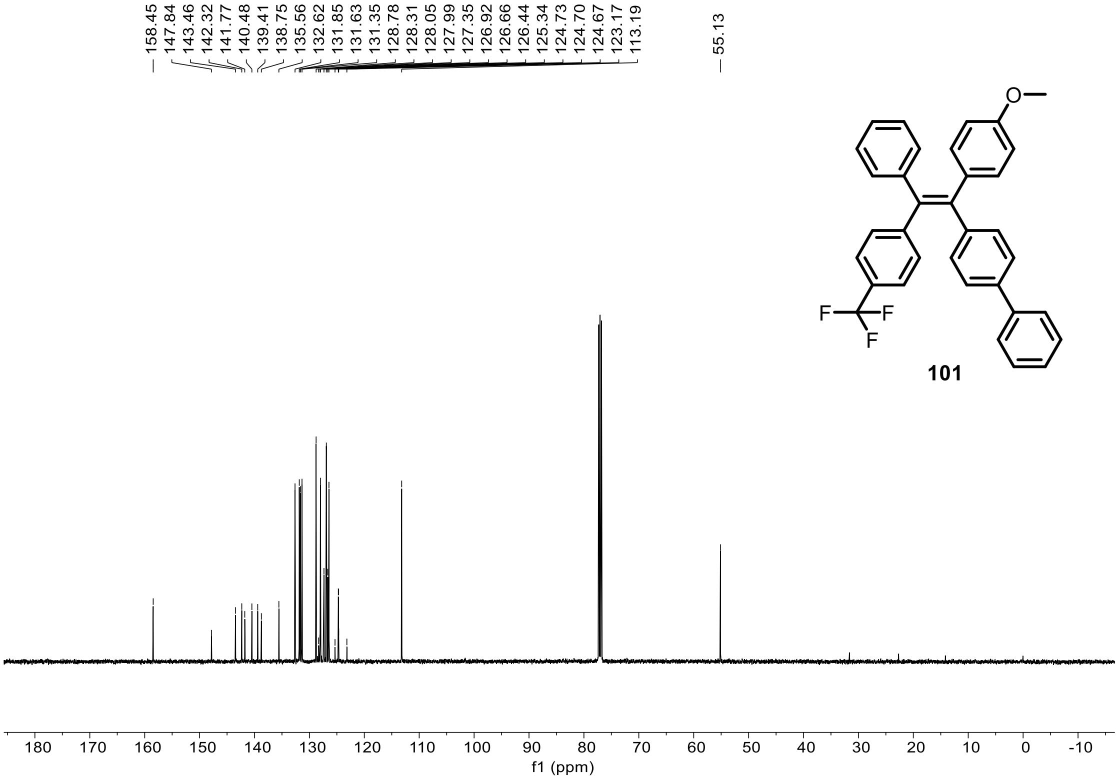

500 MHz, 298 K, $CDCl_3$ as solvent

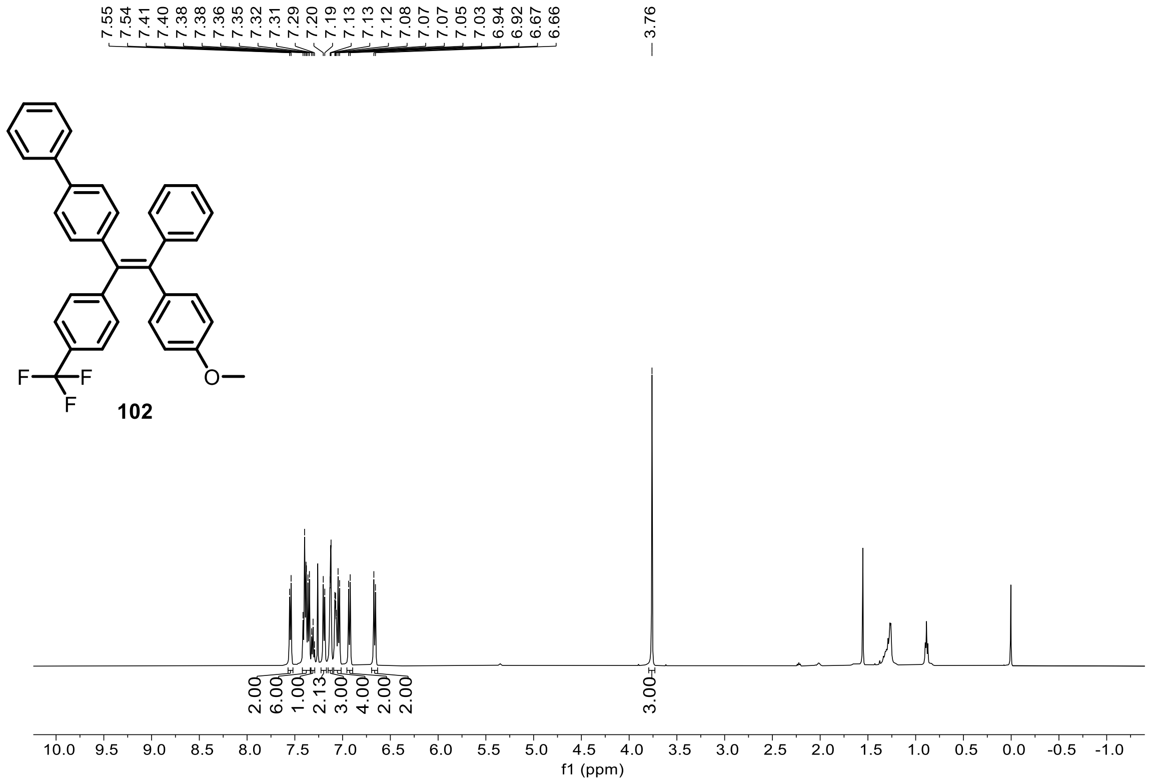

126 MHz, 298 K, $CDCl_3$ as solvent

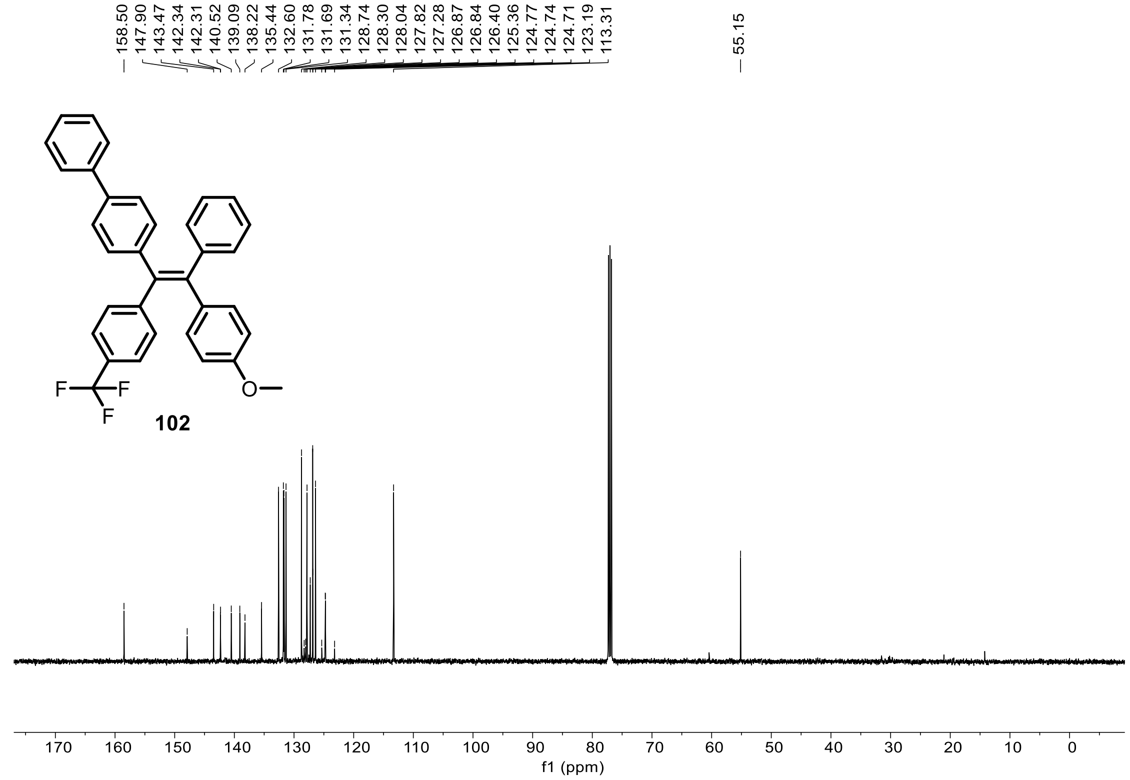

500 MHz, 298 K, $CDCl_3$ as solvent

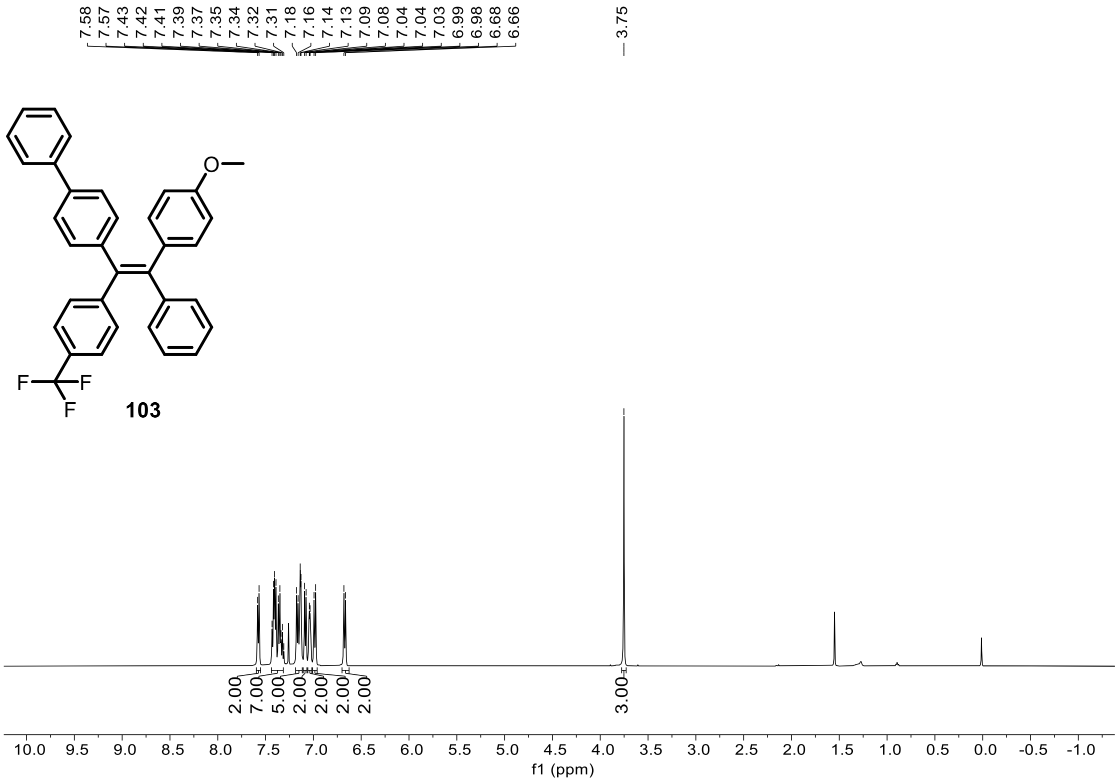

126 MHz, 298 K, $CDCl_3$ as solvent

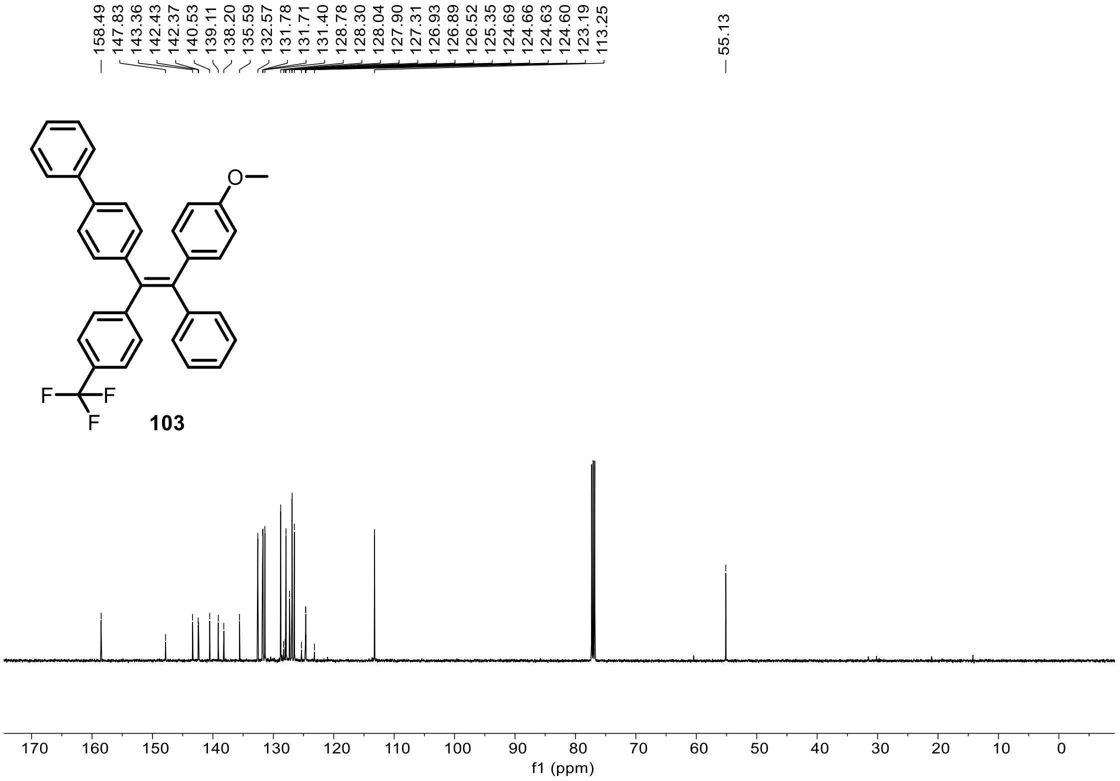

500 MHz, 298 K, $CDCl_3$ as solvent

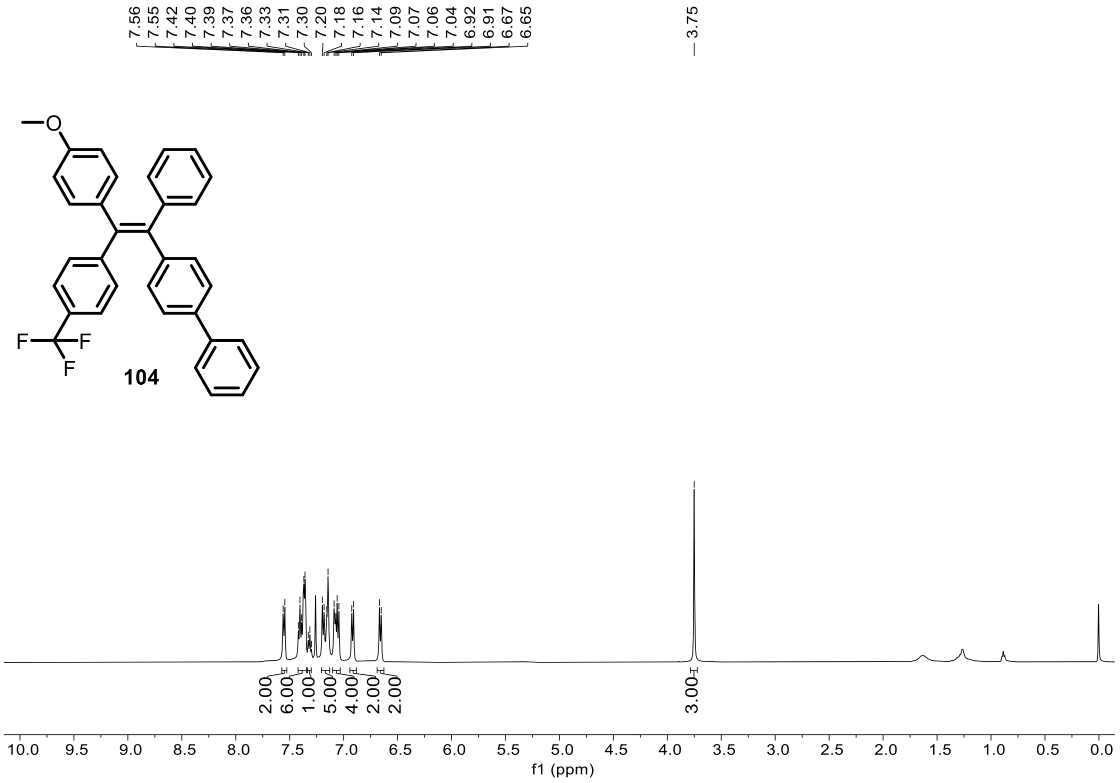

126 MHz, 298 K, $CDCl_3$ as solvent

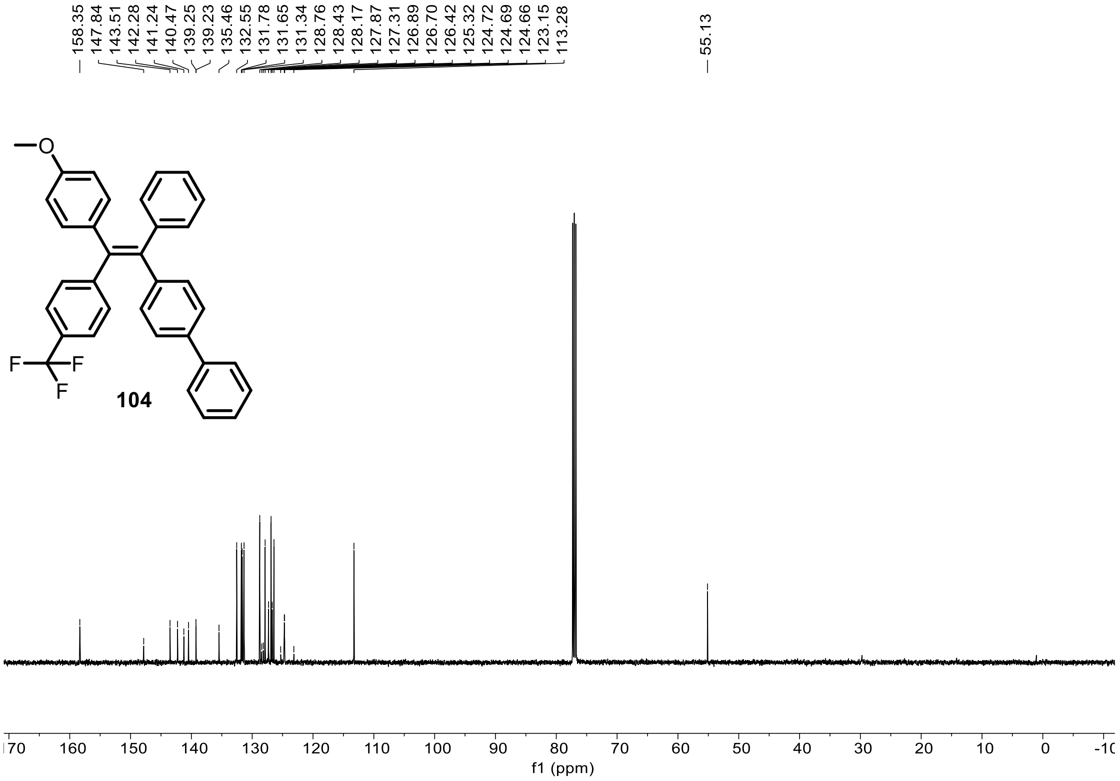

500 MHz, 298 K, $CDCl_3$ as solvent

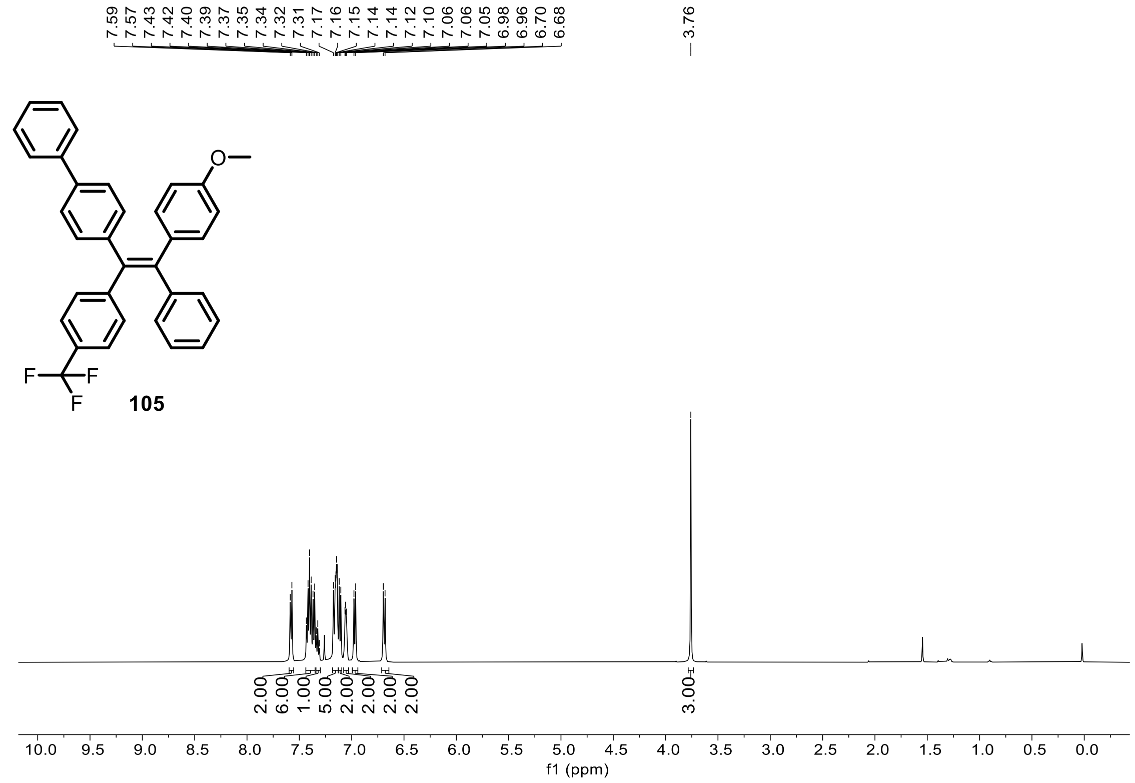

126 MHz, 298 K, $CDCl_3$ as solvent

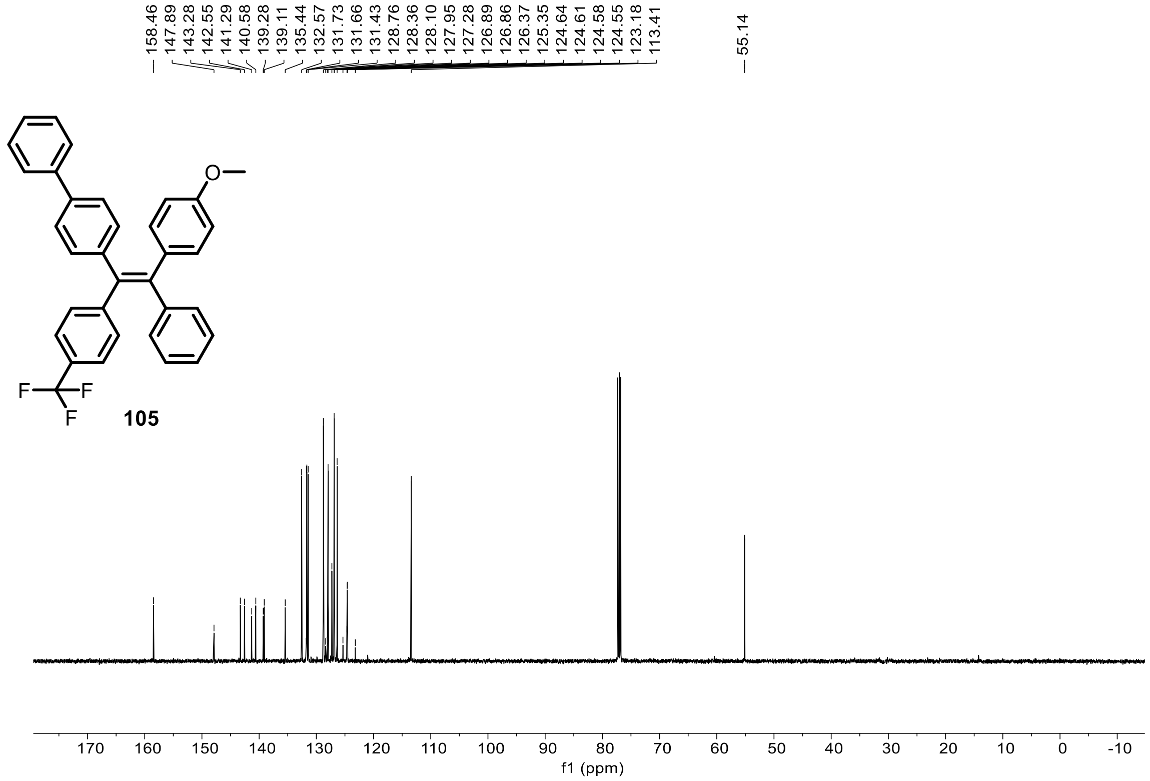

500 MHz, 298 K, $CDCl_3$ as solvent

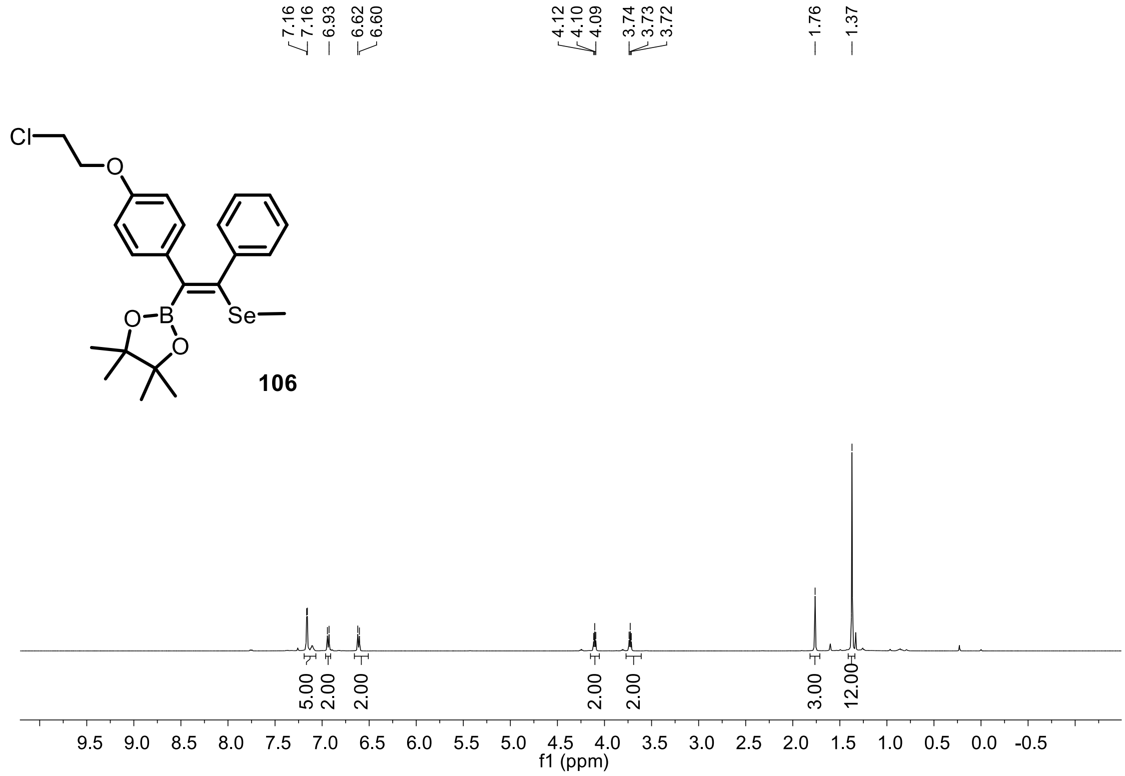

126 MHz, 298 K, $CDCl_3$ as solvent

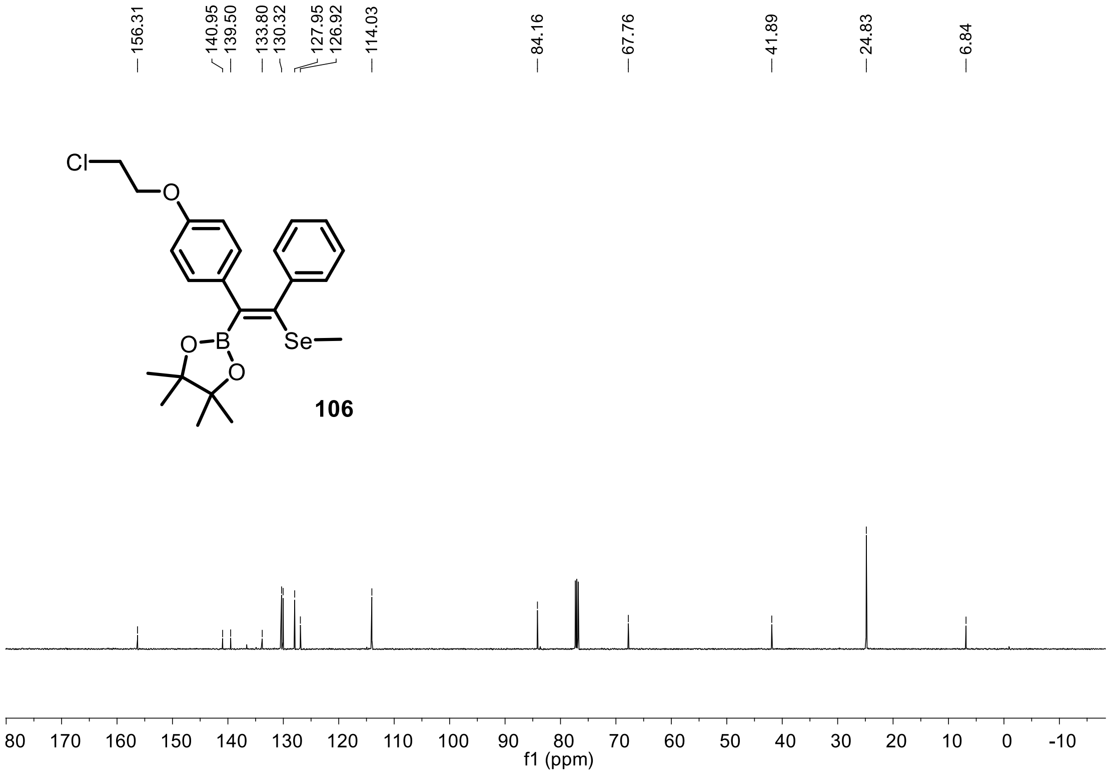

500 MHz, 298 K, CDCl$_3$ as solvent

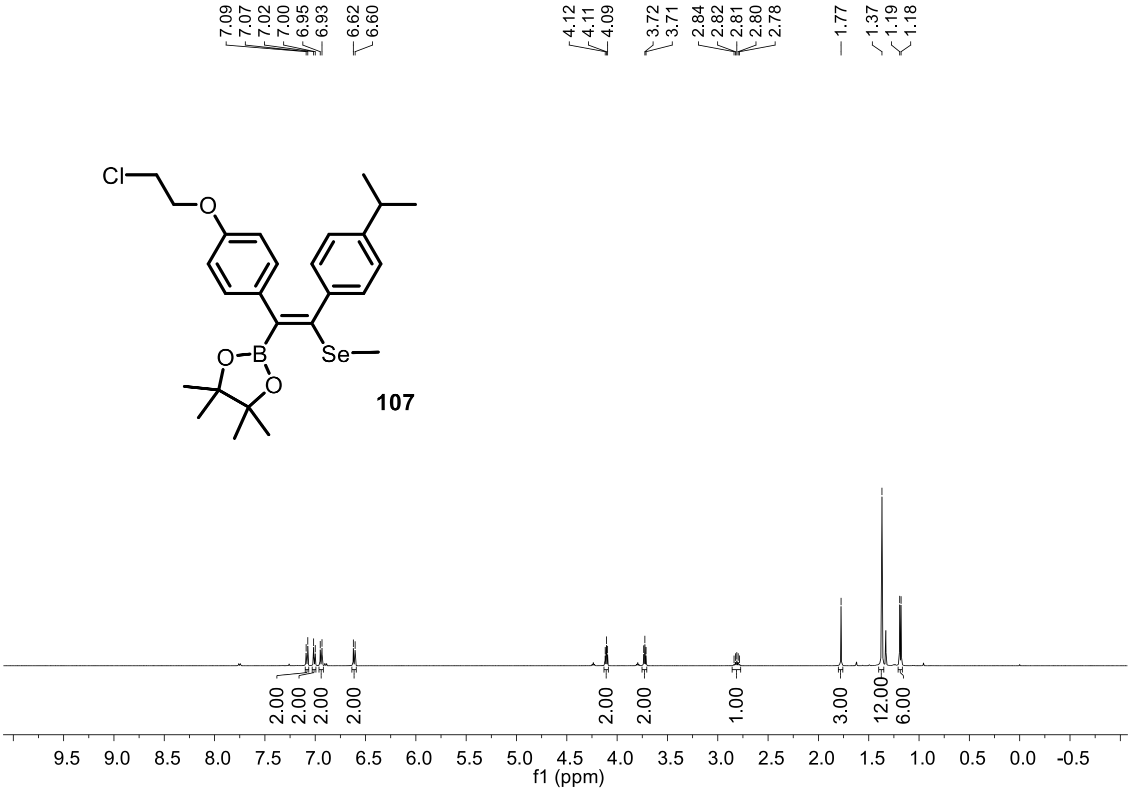

126 MHz, 298 K, CDCl$_3$ as solvent

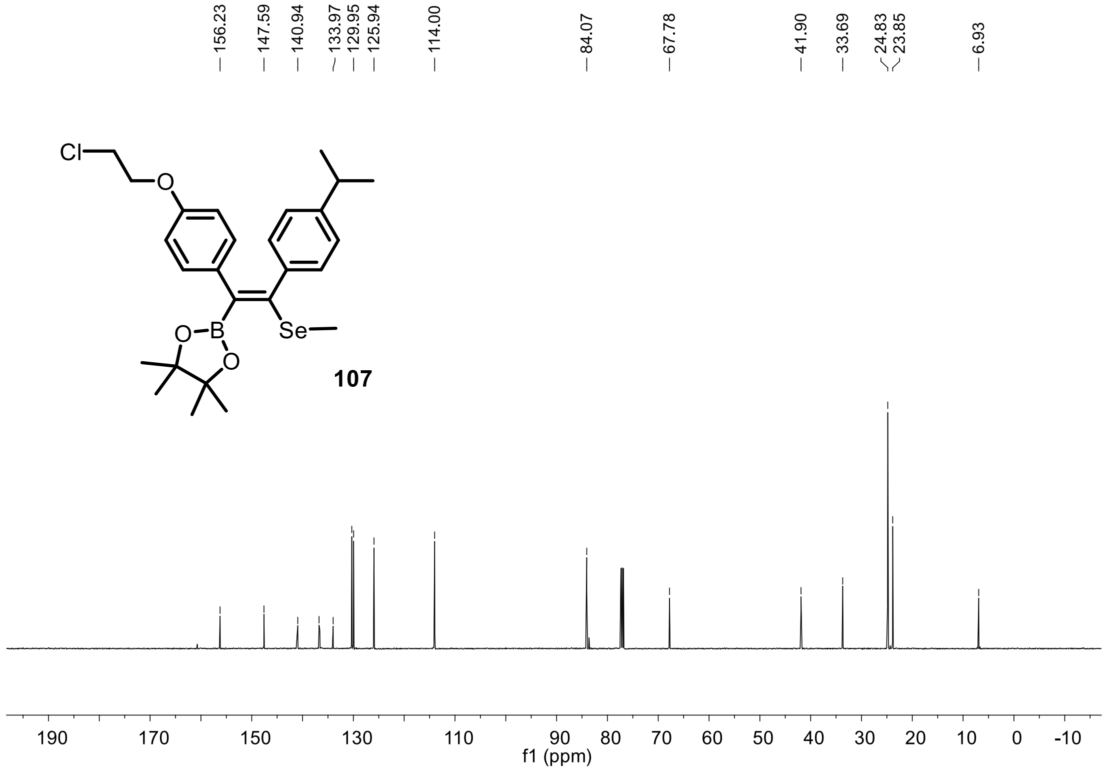

500 MHz, 298 K, $CDCl_3$ as solvent

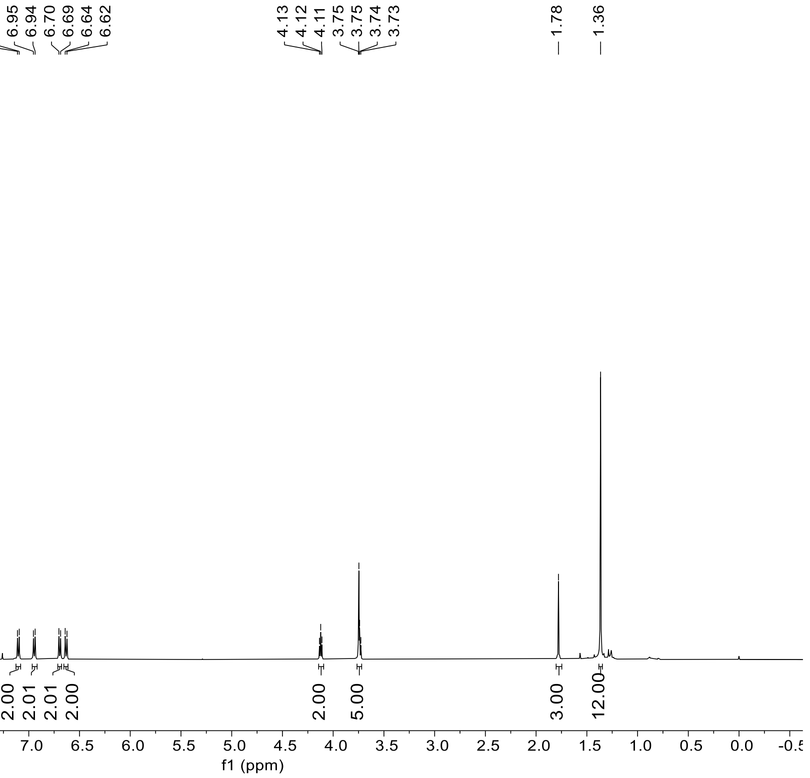

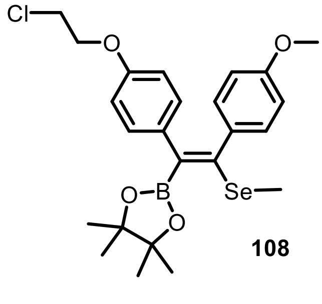

108

126 MHz, 298 K, $CDCl_3$ as solvent

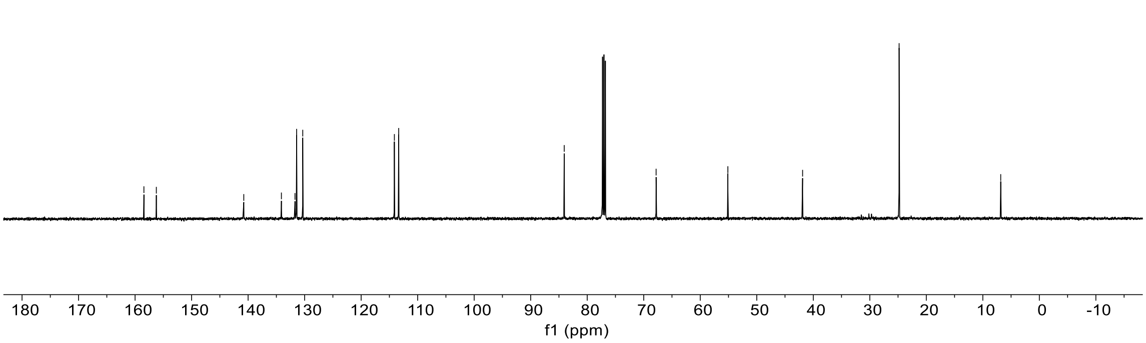

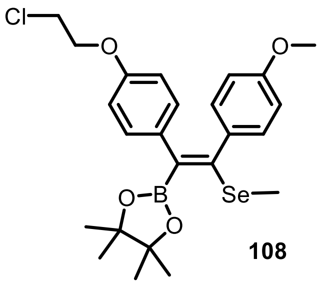

108

500 MHz, 298 K, CDCl3 as solvent

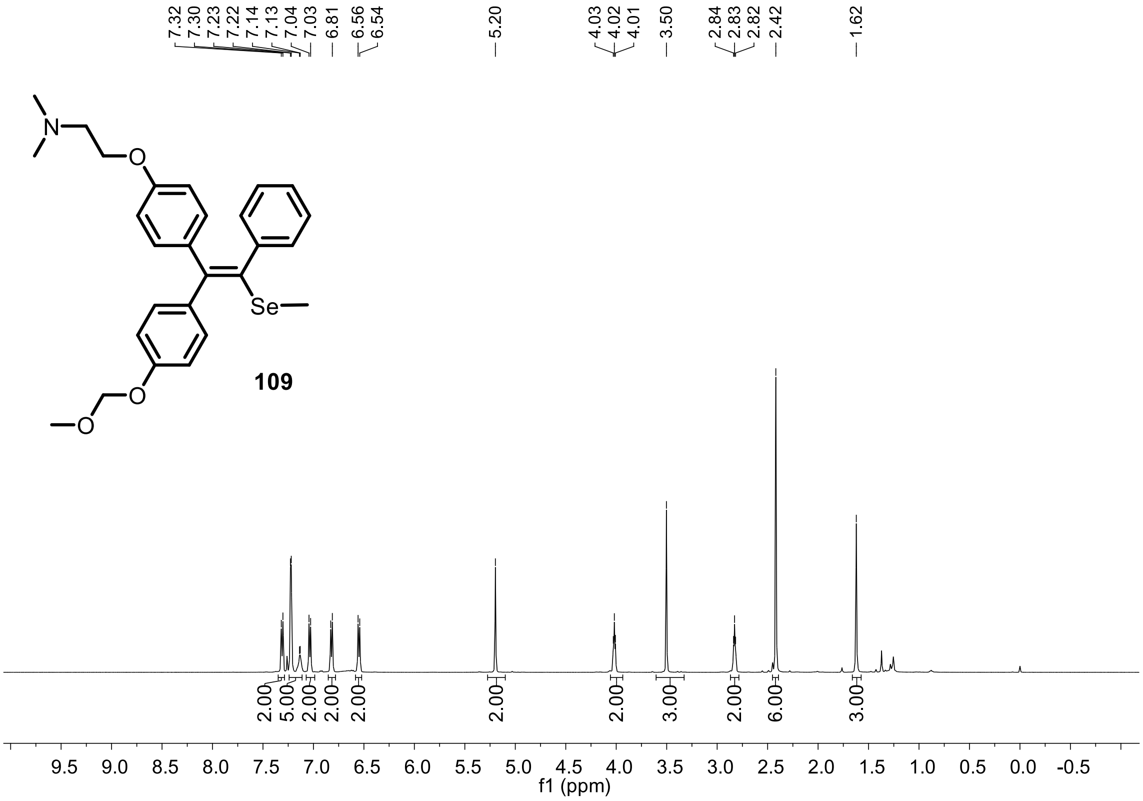

126 MHz, 298 K, CDCl3 as solvent

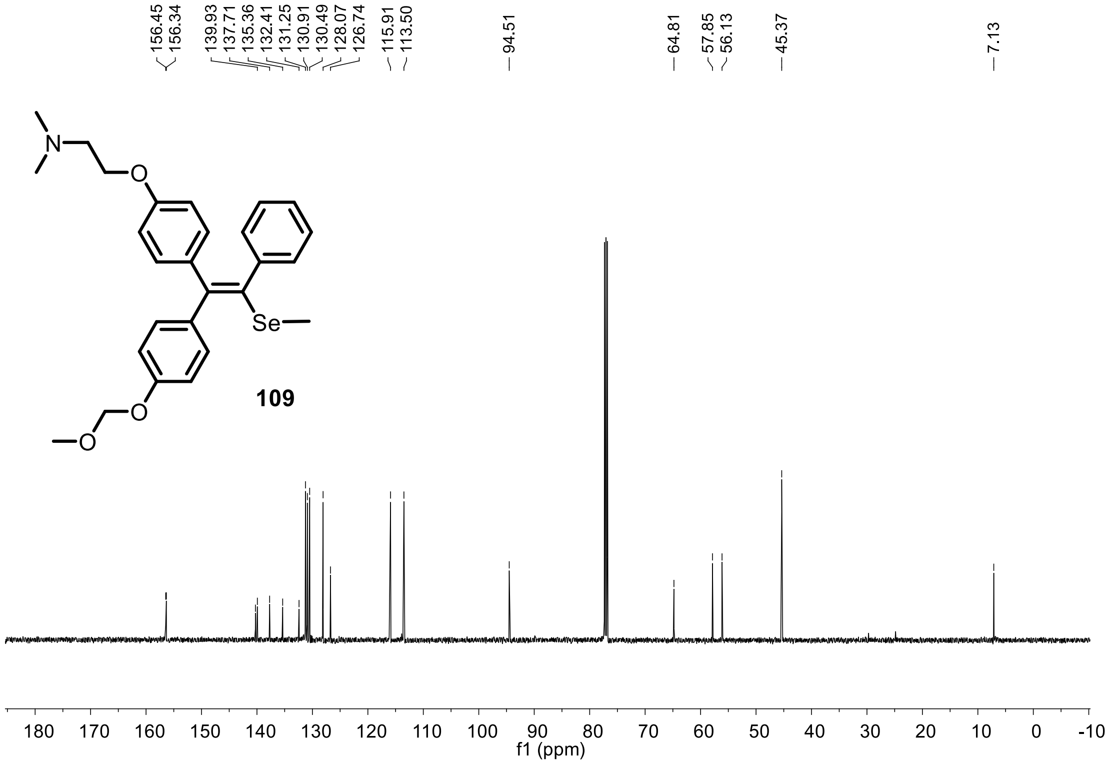

500 MHz, 298 K, $CDCl_3$ as solvent

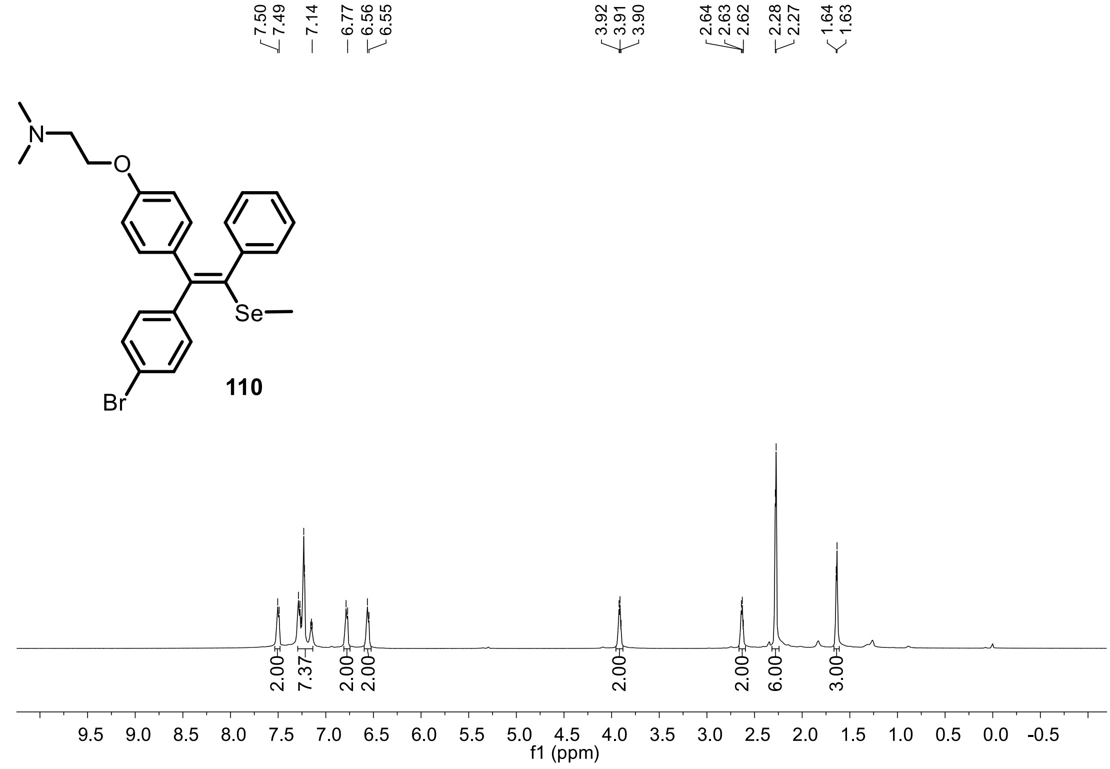

126 MHz, 298 K, $CDCl_3$ as solvent

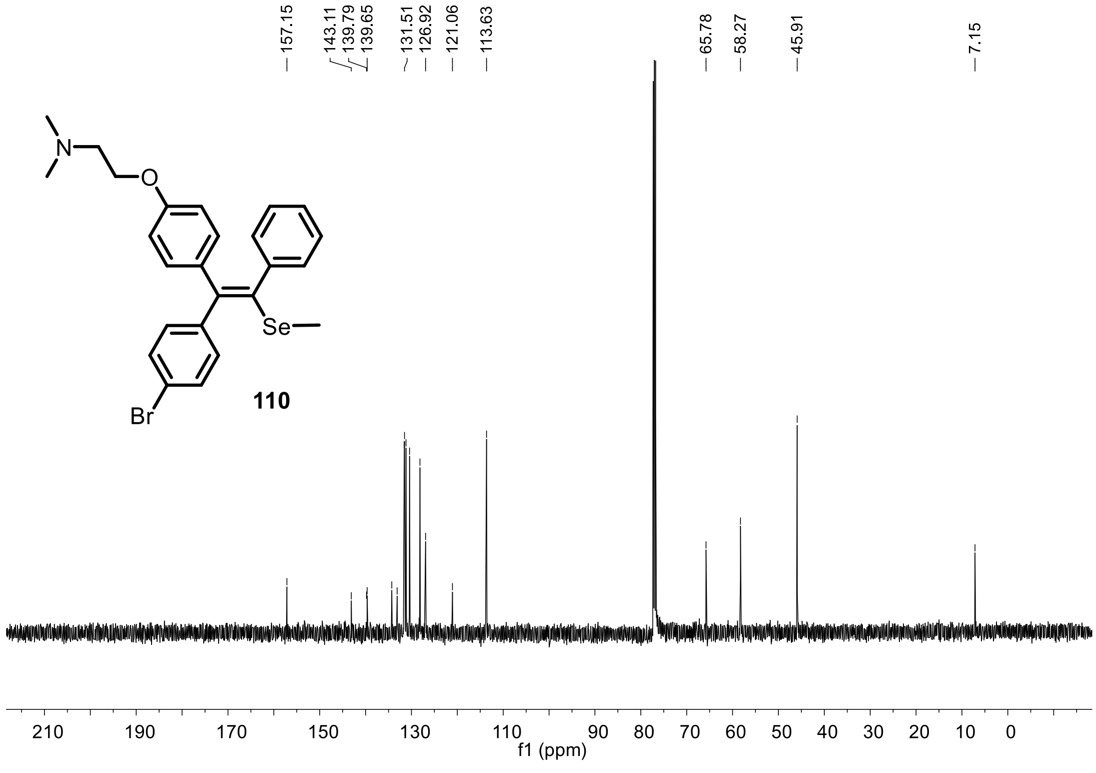

500 MHz, 298 K, $CDCl_3$ as solvent

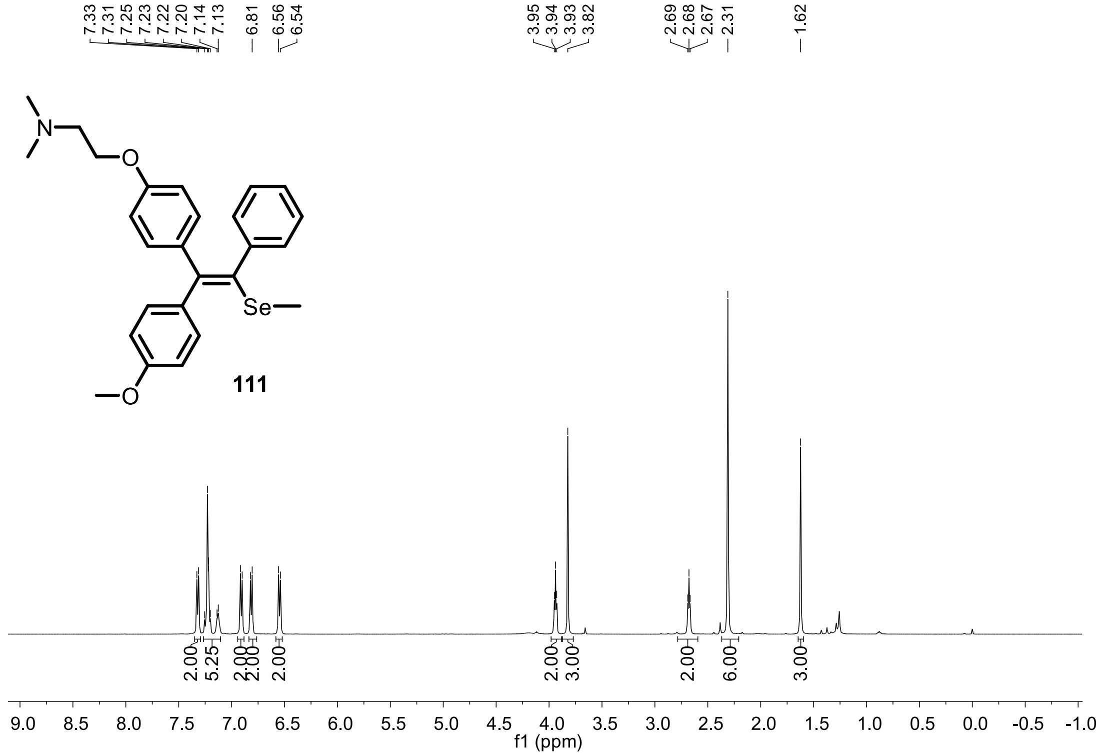

126 MHz, 298 K, $CDCl_3$ as solvent

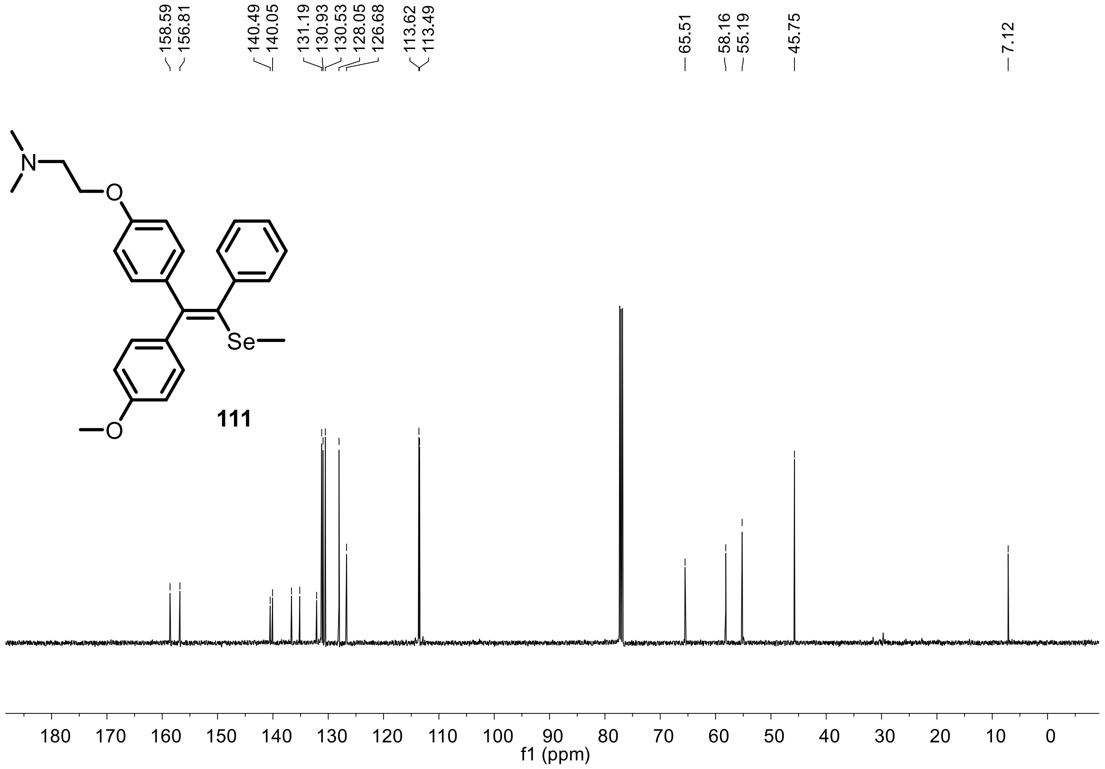

500 MHz, 298 K, $CDCl_3$ as solvent

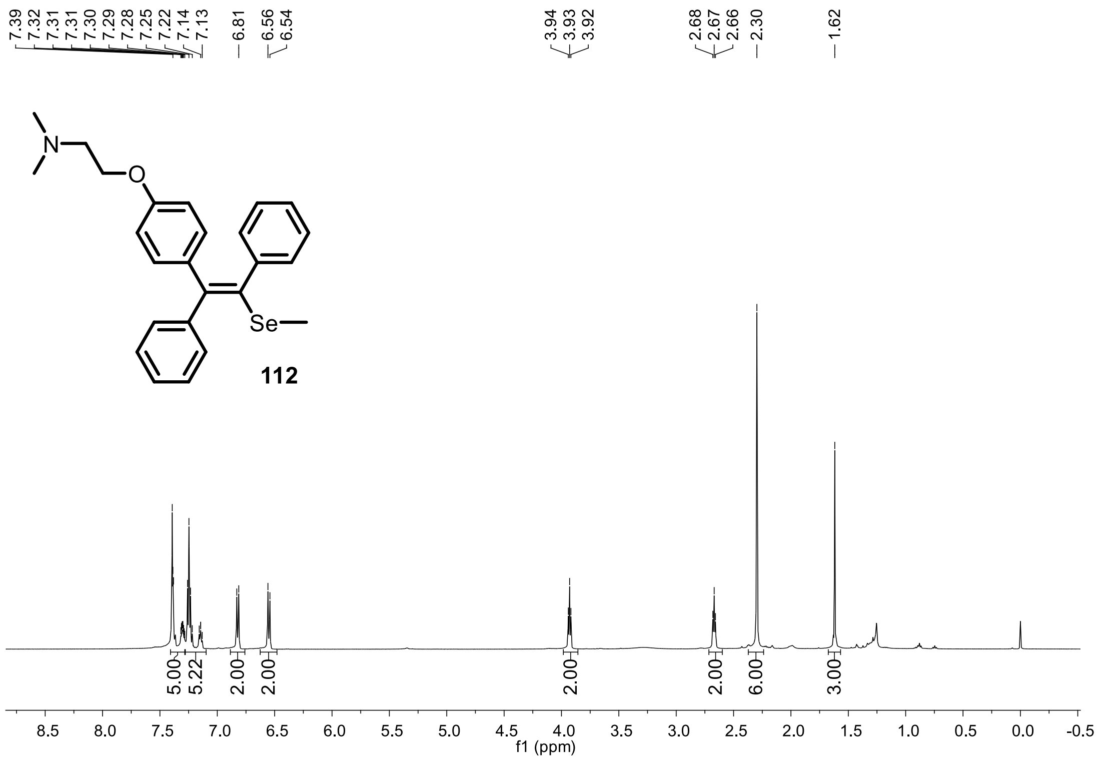

126 MHz, 298 K, $CDCl_3$ as solvent

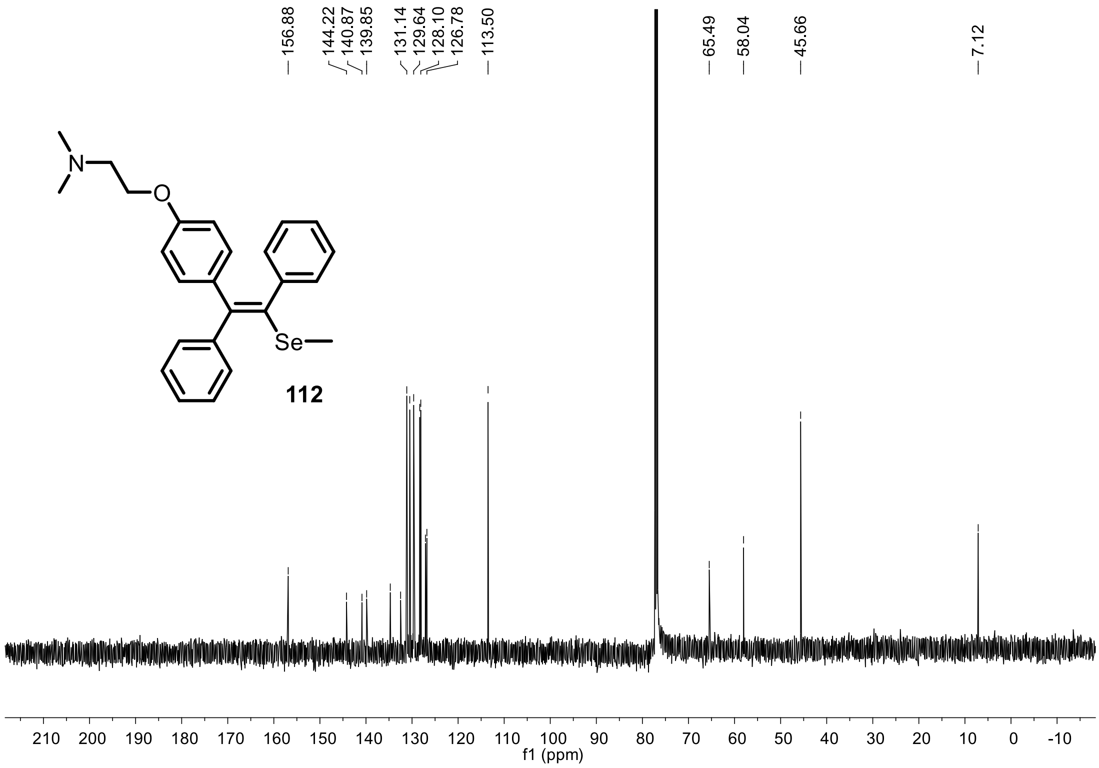

500 MHz, 298 K, MeOD as solvent

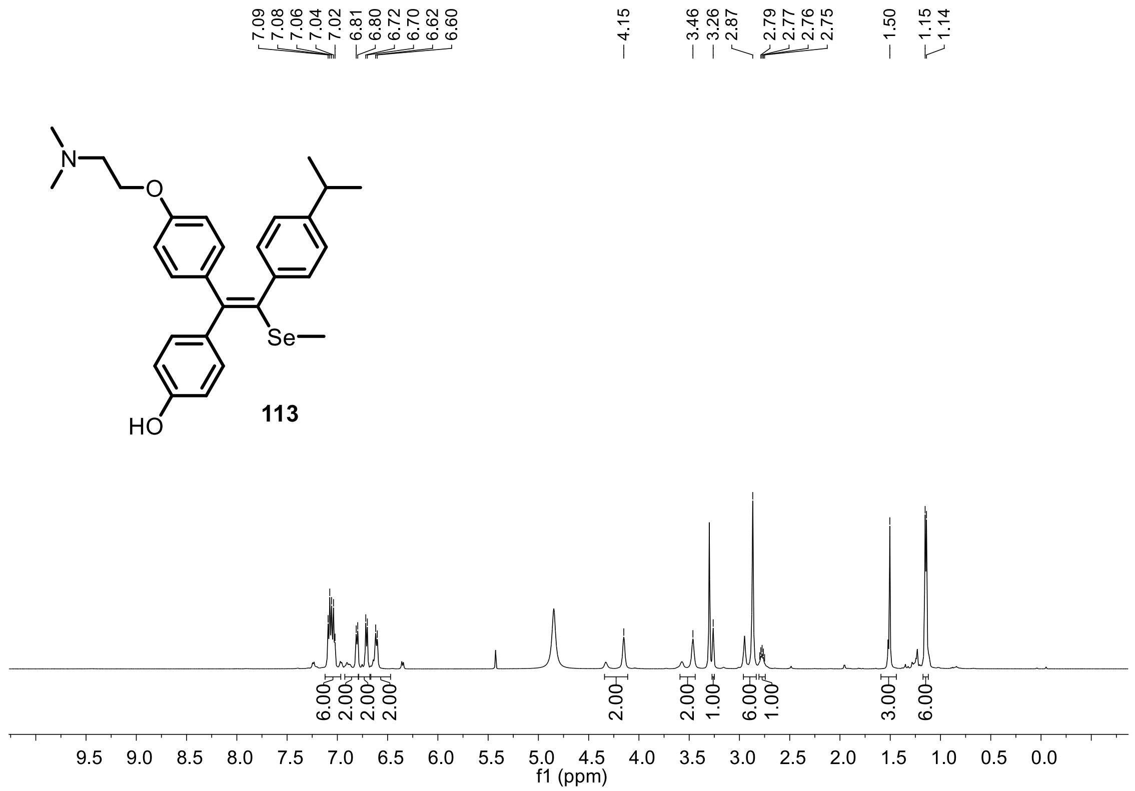

126 MHz, 298 K, MeOD as solvent

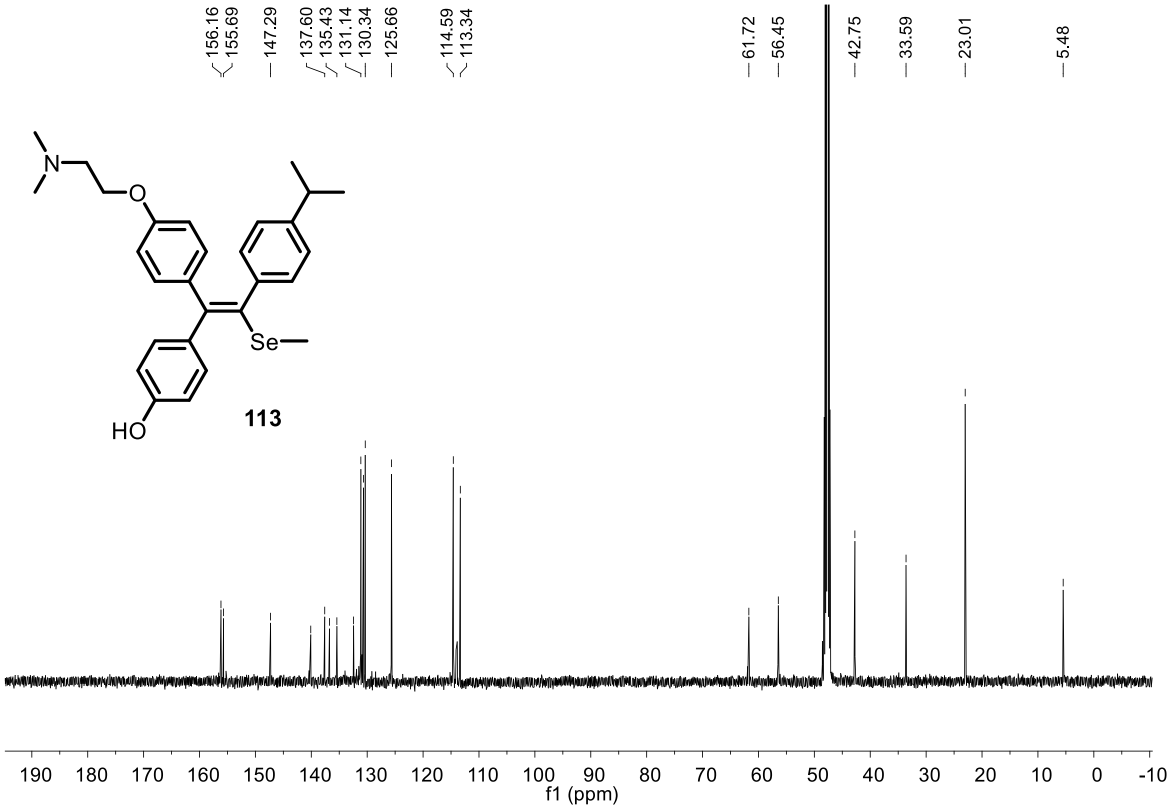

500 MHz, 298 K, $CDCl_3$ as solvent

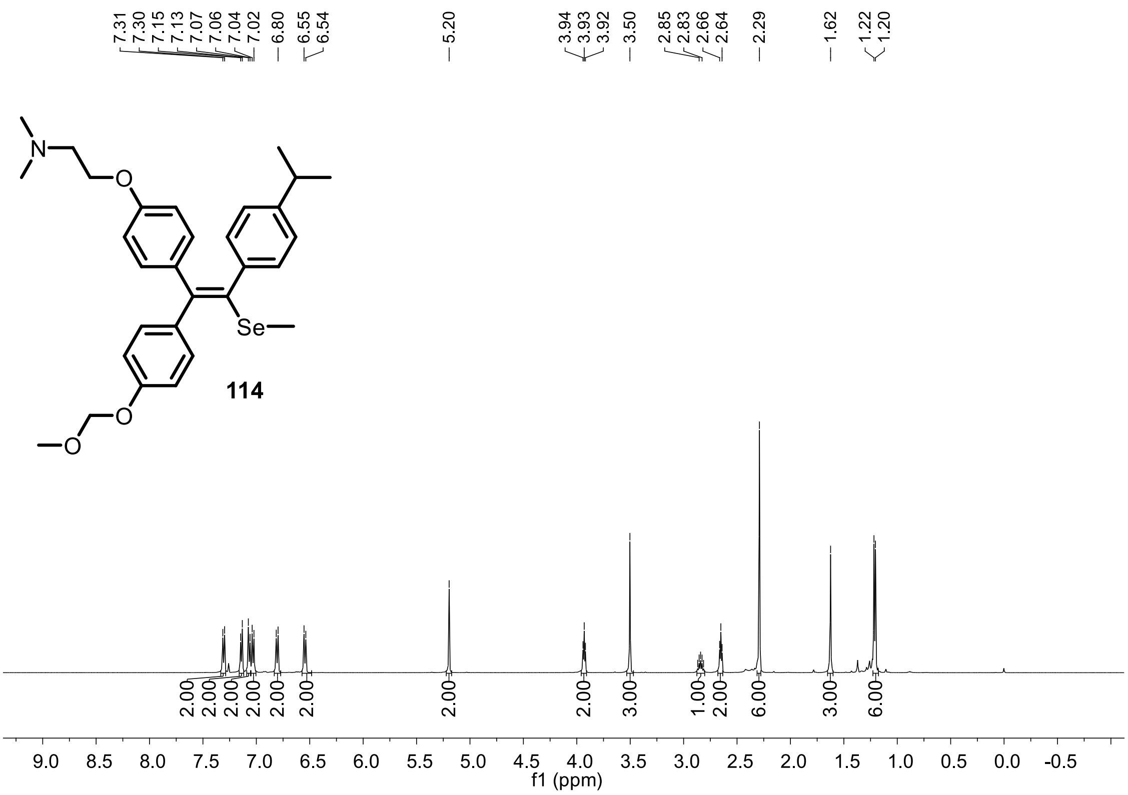

126 MHz, 298 K, $CDCl_3$ as solvent

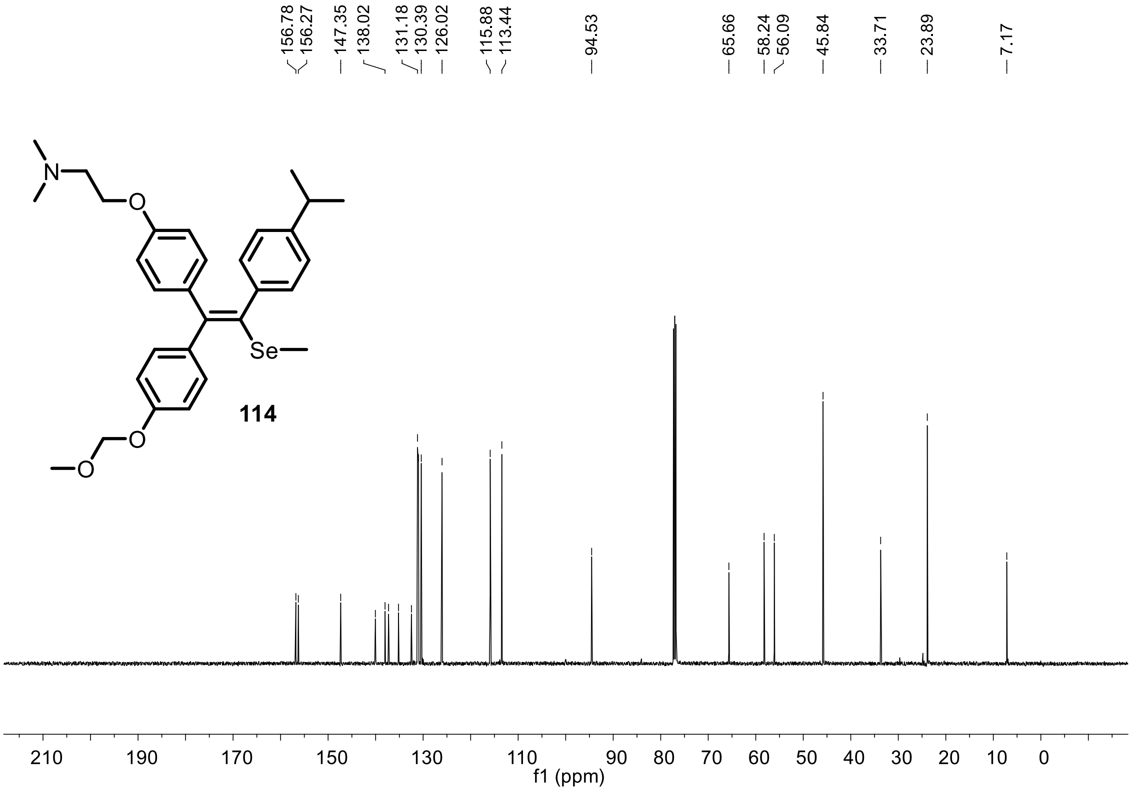

500 MHz, 298 K, CDCl3 as solvent

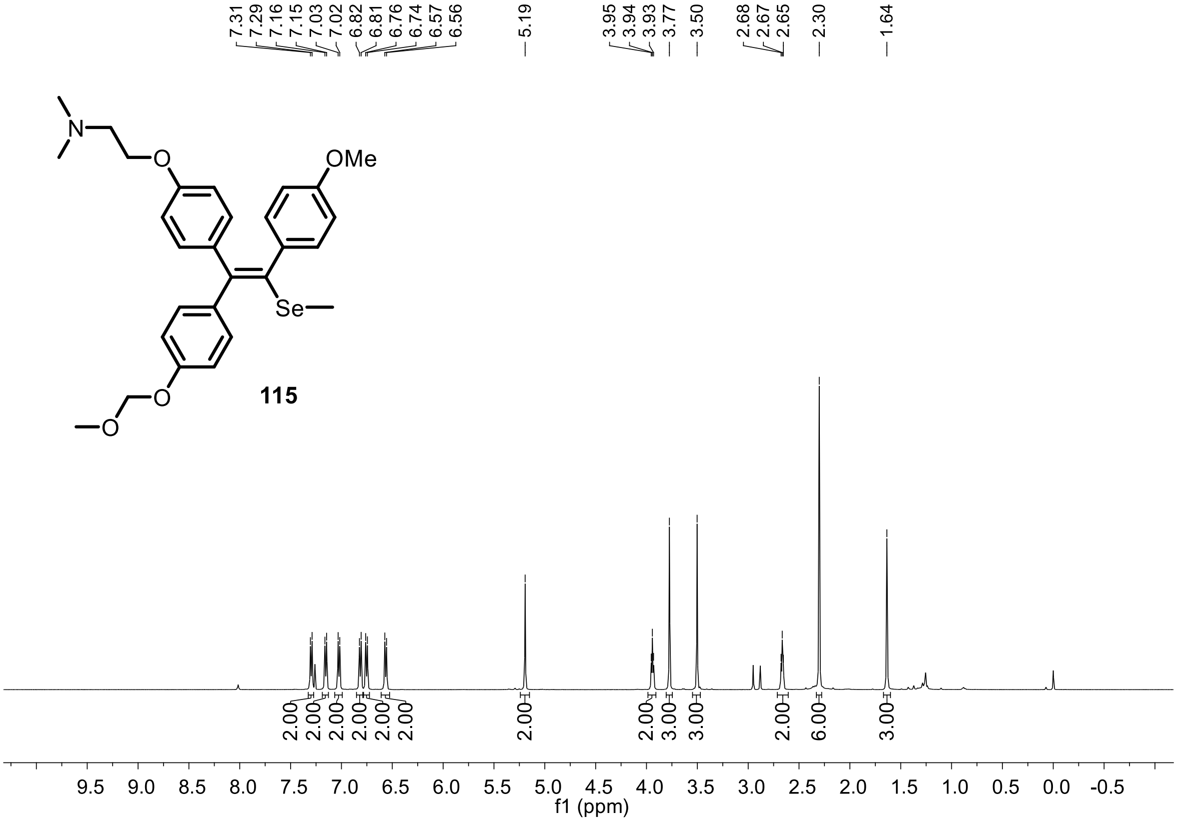

126 MHz, 298 K, CDCl3 as solvent

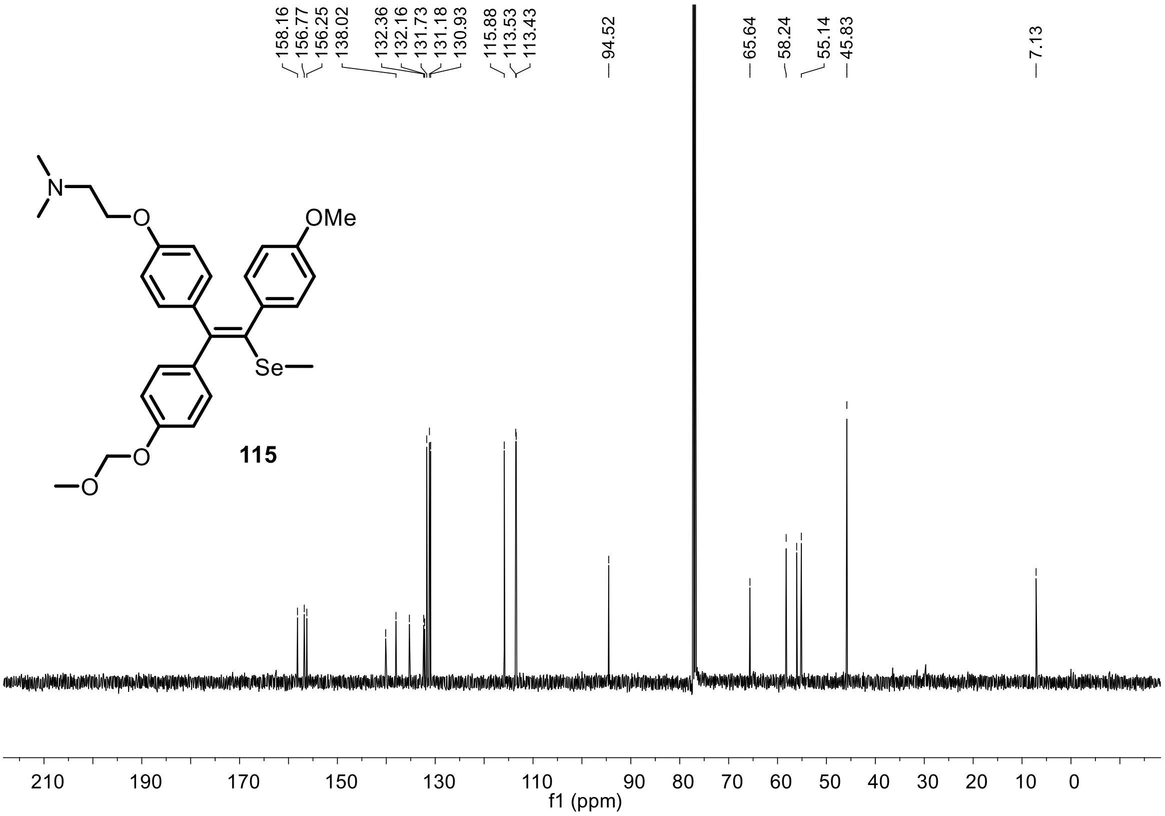

500 MHz, 298 K, $CDCl_3$ as solvent

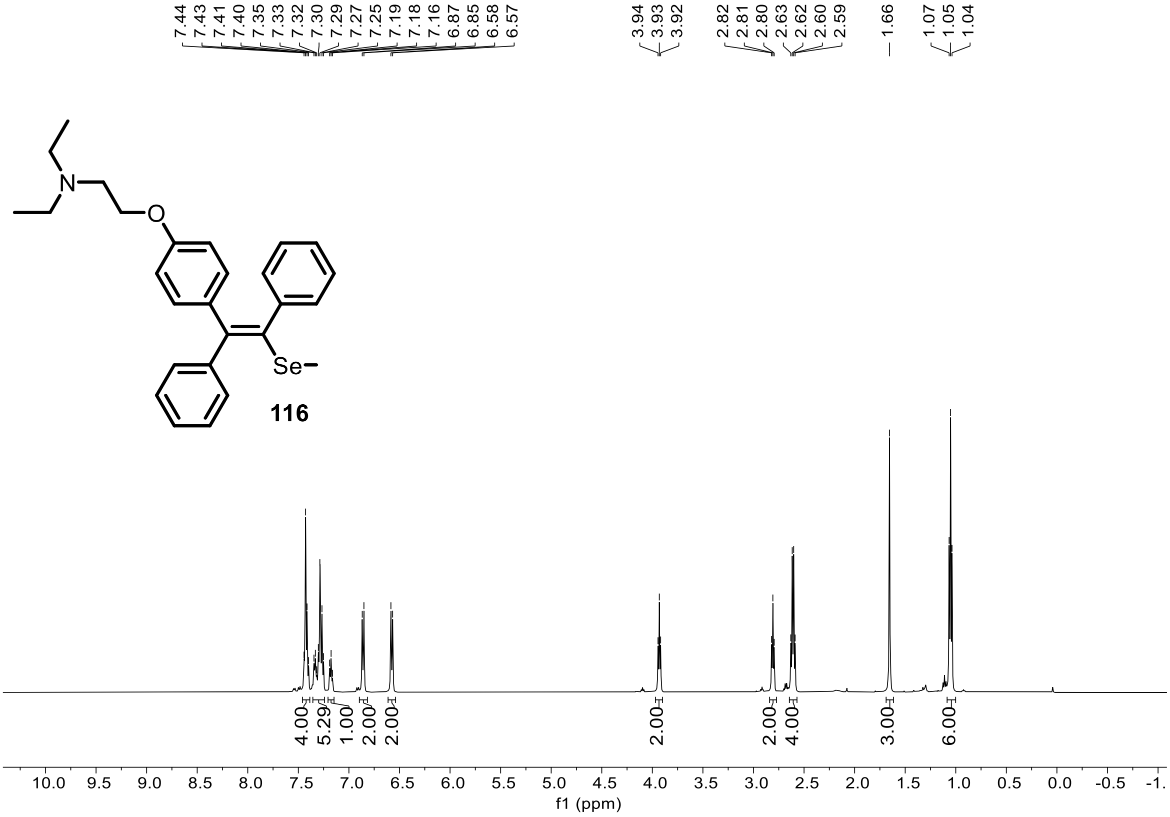

126 MHz, 298 K, $CDCl_3$ as solvent

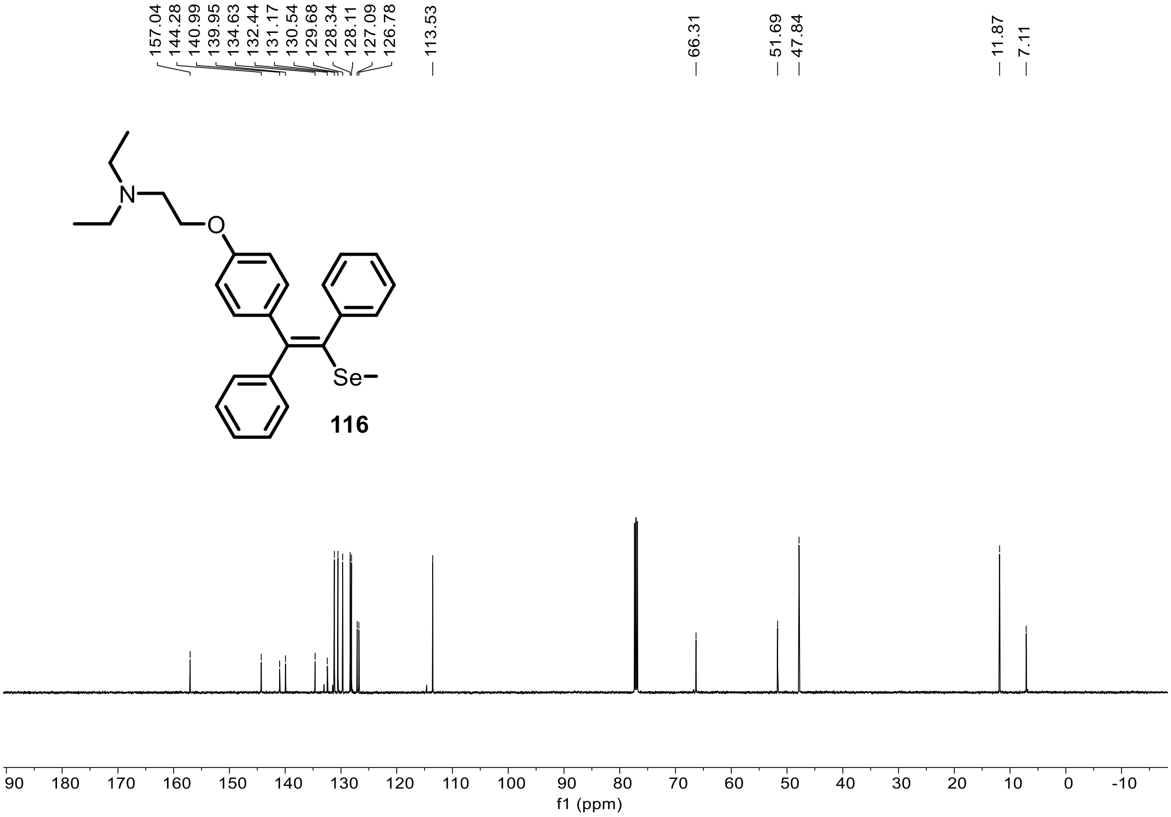

500 MHz, 298 K, CDCl3 as solvent

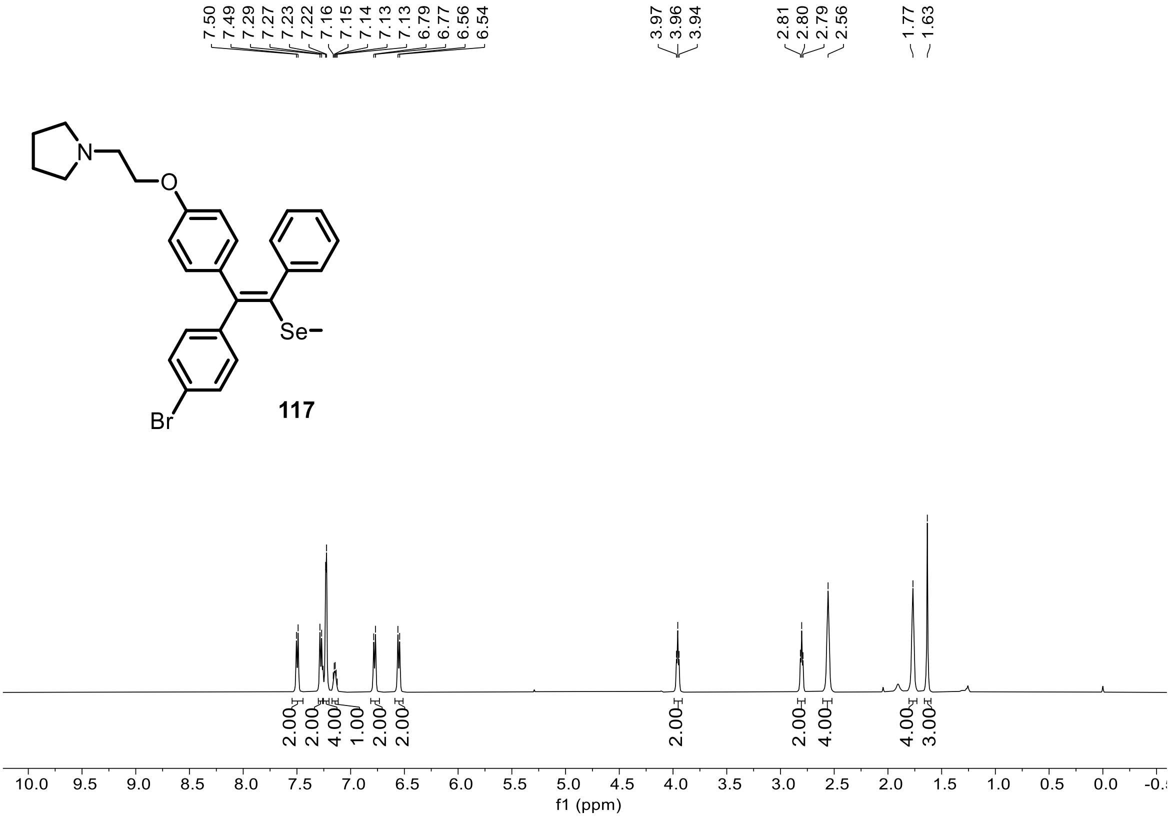

126 MHz, 298 K, CDCl3 as solvent

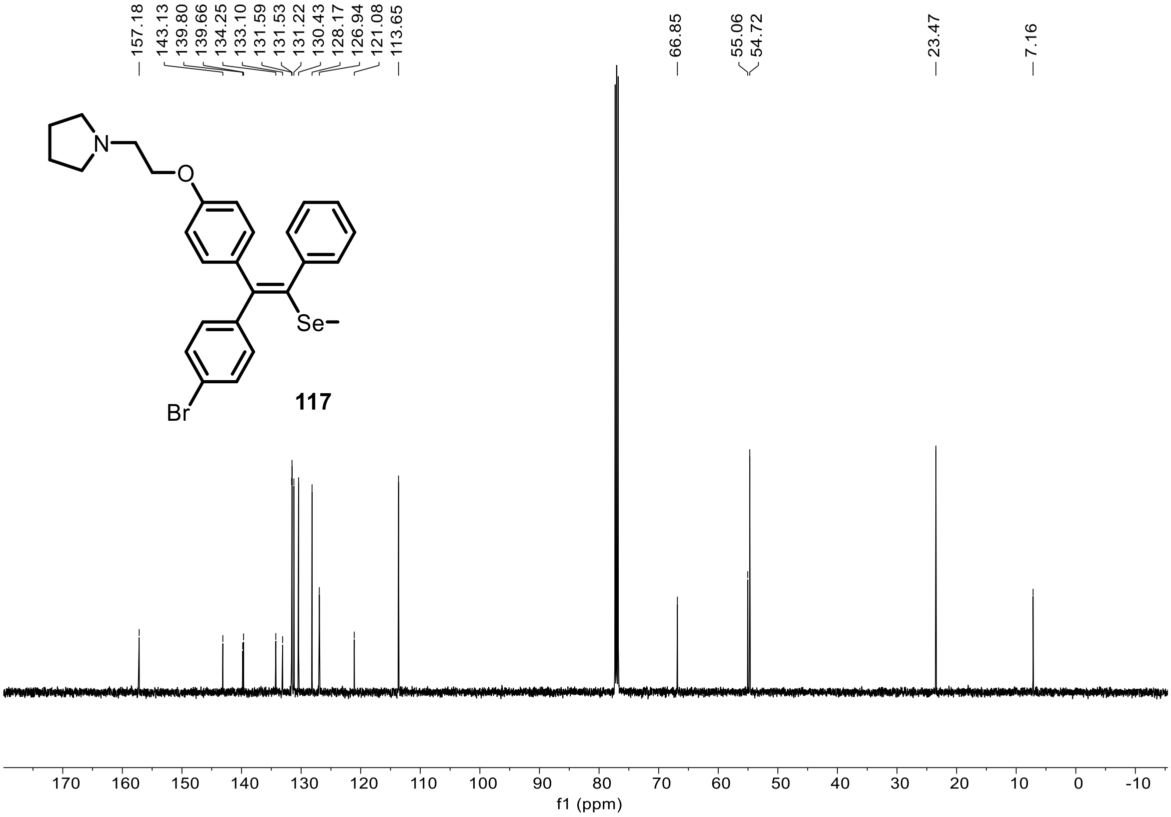

500 MHz, 298 K, $CDCl_3$ as solvent

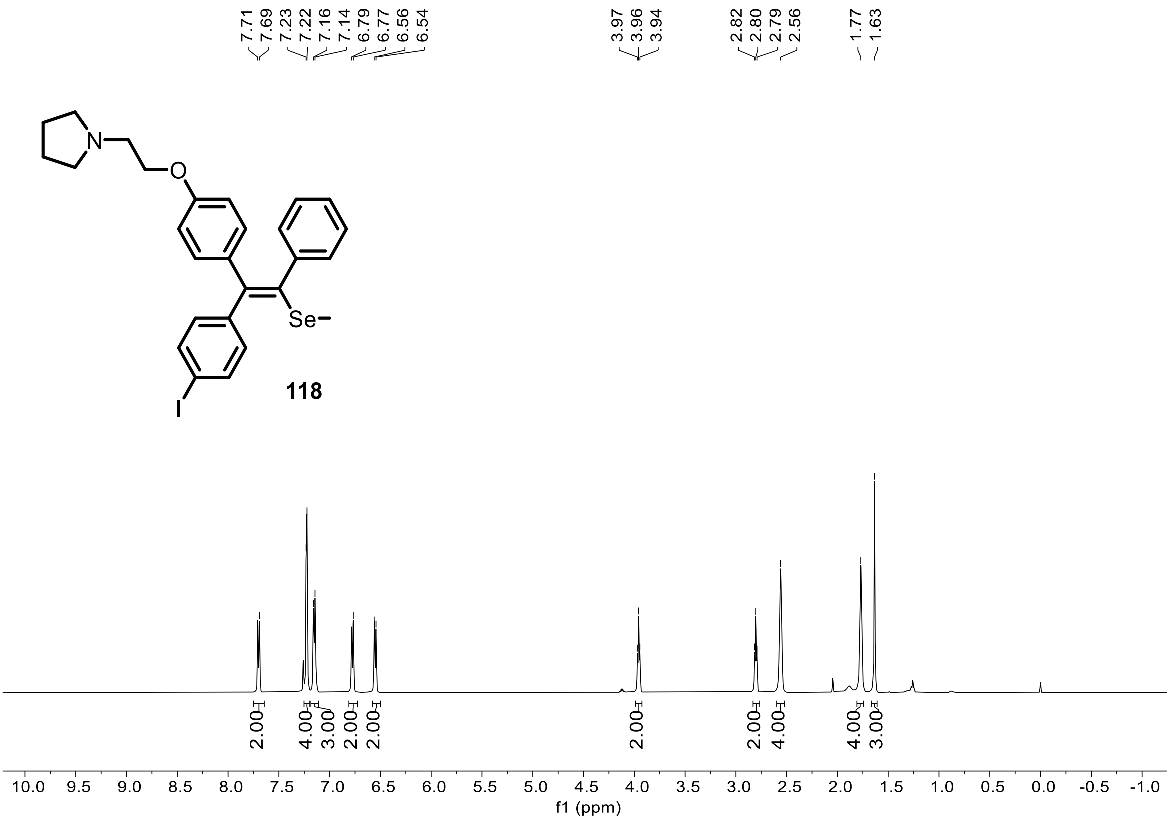

126 MHz, 298 K, $CDCl_3$ as solvent

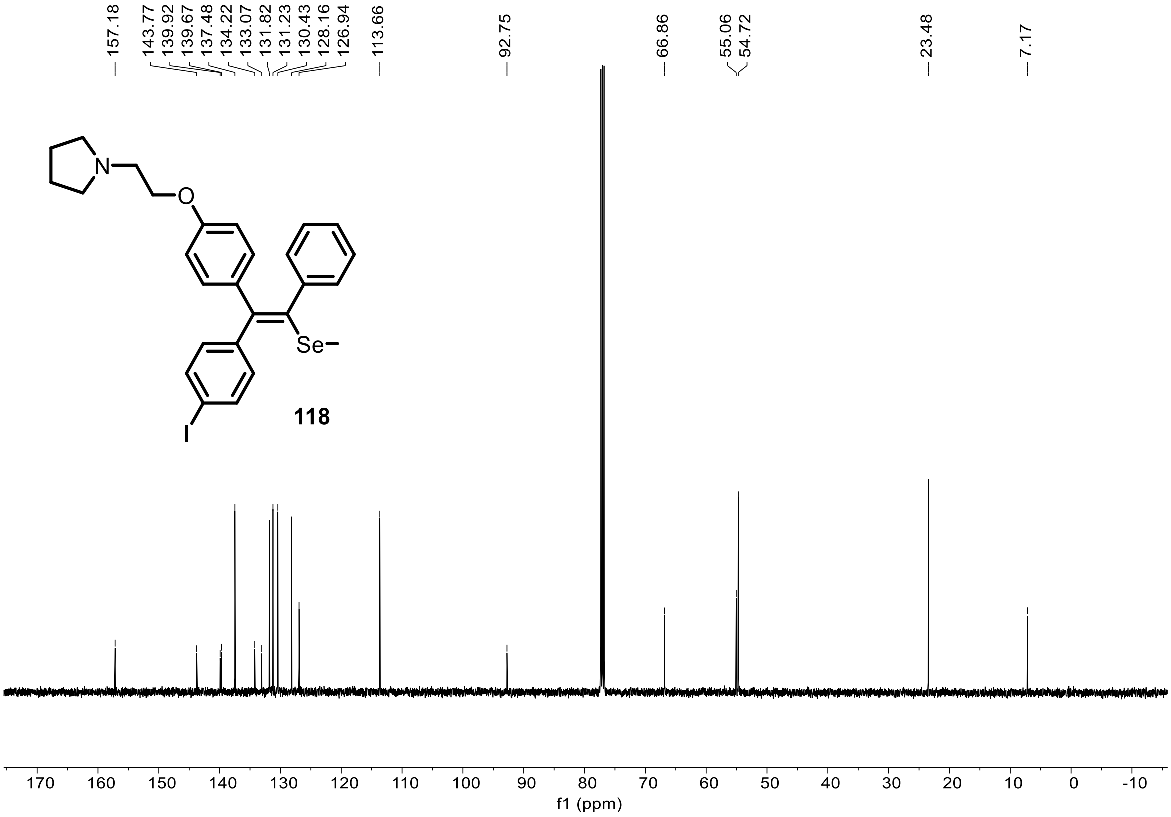

7. Supporting Crystallographic Data

CCDC 2370343

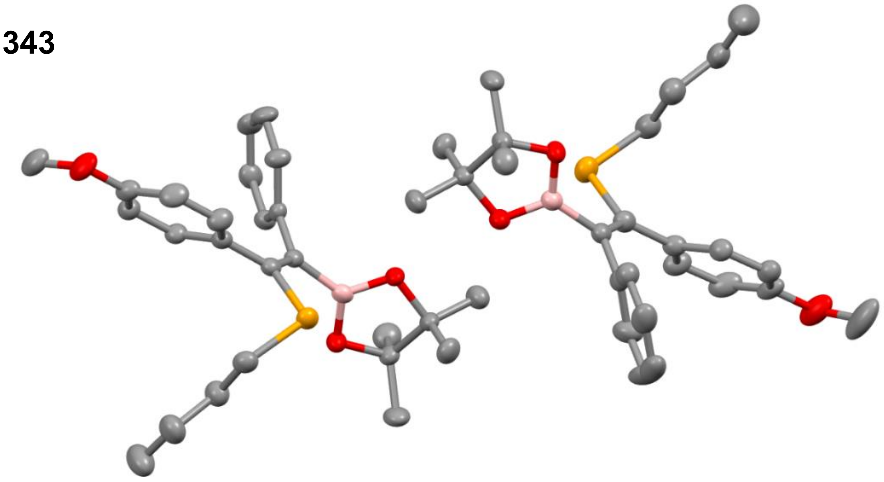

```
Bond precision:     C-C = 0.0051 A          Wavelength=1.34139

Cell:               a=21.8978(8)      b=11.8424(4)       c=18.8803(7)
                    alpha=90          beta=90.155(2)     gamma=90
Temperature:        193 K

                   Calculated                     Reported
Volume             4896.1(3)                      4896.1(3)
Space group        P 21/c                         P 1 21/c 1
Hall group         -P 2ybc                        -P 2ybc
Moiety formula     C25 H33 B O3 Se                C25 H33 B O3 Se
Sum formula        C25 H33 B O3 Se                C25 H33 B O3 Se
Mr                 471.28                         471.28
Dx,g cm-3          1.279                          1.279
Z                  8                              8
Mu (mm-1)          1.475                          1.475
F000               1968.0                         1968.0
F000'              1962.79
h,k,lmax           26,14,22                       26,14,22
Nref               8995                           8955
Tmin,Tmax          0.838,0.863                    0.630,0.751
Tmin'              0.838

Correction method= # Reported T Limits: Tmin=0.630 Tmax=0.751
AbsCorr = MULTI-SCAN

Data completeness= 0.996            Theta(max)= 53.999

                                                       wR2(reflections)=
R(reflections)= 0.0439( 6005)                          0.1146( 8955)

S = 1.032                 Npar= 553
```

CCDC 2369379

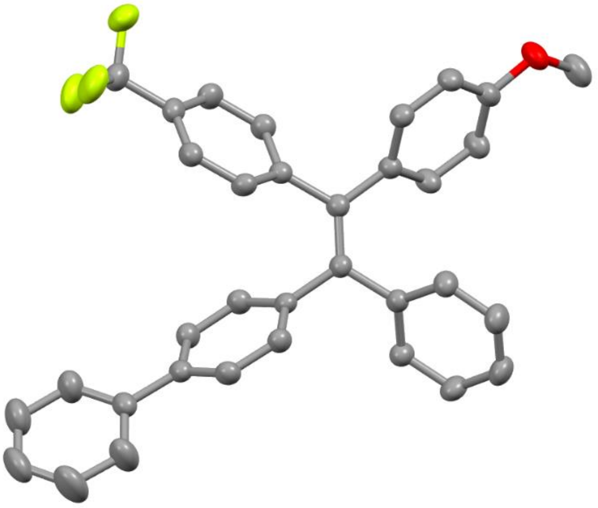

Bond precision:	C-C = 0.0021 A		Wavelength=1.34139
Cell:	a=5.5173(4)	b=17.4702(11)	c=27.0809(18)
	alpha=90	beta=90.490(2)	gamma=90
Temperature:	170 K		

	Calculated	Reported
Volume	2610.2(3)	2610.2(3)
Space group	P 21/n	P 1 21/n 1
Hall group	-P 2yn	-P 2yn
Moiety formula	C34 H25 F3 O	C34 H25 F3 O
Sum formula	C34 H25 F3 O	C34 H25 F3 O
Mr	506.54	506.54
Dx,g cm-3	1.289	1.289
Z	4	4
Mu (mm-1)	0.476	0.476
F000	1056.0	1056.0
F000'	1058.59	
h,k,lmax	7,22,35	7,22,35
Nref	6004	5986
Tmin,Tmax	0.966,0.981	0.658,0.752
Tmin'	0.927	

Correction method= # Reported T Limits: Tmin=0.658 Tmax=0.752
AbsCorr = MULTI-SCAN

Data completeness= 0.997 Theta(max)= 60.724

R(reflections)= 0.0476(4828) wR2(reflections)= 0.1340(5986)

S = 1.042 Npar= 344

CCDC 2369378

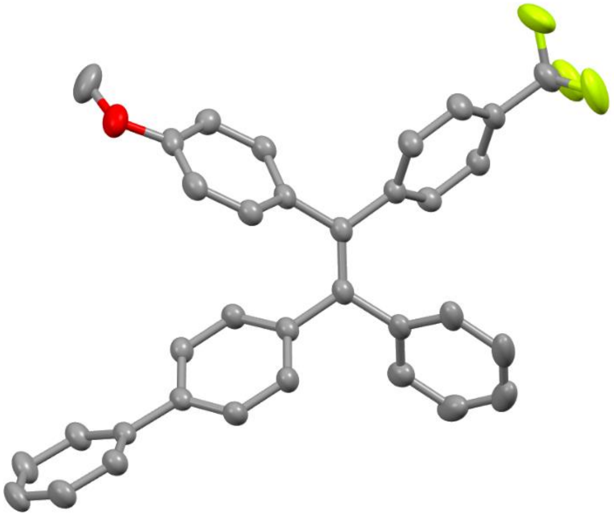

```
Bond precision:    C-C = 0.0025 A           Wavelength=1.34139

Cell:              a=10.9703(8)        b=11.0311(8)        c=11.5616(8)
                   alpha=105.615(3)    beta=105.238(2)     gamma=90.061(3)
Temperature:       170 K

                    Calculated                         Reported
Volume              1296.22(16)                        1296.22(16)
Space group         P -1                               P -1
Hall group          -P 1                               -P 1
Moiety formula      C34 H25 F3 O                       C34 H25 F3 O
Sum formula         C34 H25 F3 O                       C34 H25 F3 O
Mr                  506.54                             506.54
Dx,g cm-3           1.298                              1.298
Z                   2                                  2
Mu (mm-1)           0.479                              0.479
F000                528.0                              528.0
F000'               529.30
h,k,lmax            14,14,15                           14,14,15
Nref                6002                               5917
Tmin,Tmax           0.966,0.981                        0.651,0.752
Tmin'               0.940

Correction method= # Reported T Limits: Tmin=0.651 Tmax=0.752
AbsCorr = MULTI-SCAN

Data completeness= 0.986           Theta(max)= 60.847

                                                       wR2(reflections)=
R(reflections)= 0.0564( 4163)                          0.1595( 5917)

S = 1.062                  Npar= 344
```

8. References

1. Zhu, D. & Shi, L. Ni-Catalyzed cross-coupling of aryl thioethers with alkyl Grignard reagents via C-S bond cleavage. *Chem. Commun.,* **54**, 9313−9316 (2018).
2. Chen, J., Chen, S., Xu, X., Tang, Z., Au, C.-T. & Qiu, R. Nickel-catalyzed regioselective cleavage of C_{sp^2}−S bonds: method for the synthesis of tri- and tetrasubstituted alkenes. *J. Org. Chem.,* **81**, 3246−3255 (2016).
3. Someya, C. I., Irran, E. & Enthaler, S. Synthesis of Ni(II) complexes with unsymmetric [O,N,O']-pincer ligands and their use as precatalysts in carbon–carbon bond formations to access diarylmethanes. *Inorganica Chimica Acta* **421**, 136–144 (2014).
4. Greiner, R., Ziegler, D. S., Cibu, D., Jakowetz, A. C., Auras, F., Bein, T. & Knoche, P. Preparation of polyfunctional naphthyridines by cobalt-catalyzed cross-couplings of halogenated naphthyridines with magnesium and zinc organometallics. *Org. Lett.* **19**, 6384−6387 (2017).
5. Tang, S.-Q., Bricard, J., Schmitt, M. & Bihel, F. Fukuyama cross-coupling approach to isoprekinamycin: discovery of the highly active and bench-stable palladium precatalyst POxAP. *Org. Lett.* **21**, 844−848 (2019).
6. Narangoda, C. J., Lex, T. R., Moore, M. A., McMillen, C. D., Kitaygorodskiy, A., Jackson, J. E. & Colorlesshead, D. C. Accessing the rare diazacyclobutene motif. *Org. Lett.* **20**, 8009–8013 (2018).
7. Wang, H., Erchinger, J. E., Lenz, M., Dutta, S., Daniliuc, C. G. & Glorius, F. syn-selective difunctionalization of bicyclobutanes enabled by photoredox-mediated C–S σ-bond scission *J. Am. Chem. Soc.* **145**, 23771–23780 (2023).
8. Heredia, A. A. & Peñéñory, A. B. Transition-metal-free one-pot synthesis of alkynyl selenides from terminal alkynes under aerobic and sustainable conditions. *Beilstein J. Org. Chem*. **13**, 910–918 (2017).
9. de Oliveira, I. M., Esteves, H. A., Darbem, M. P., Sartorelli, A., Correra, T. C., Rodrigues-Oliveira, A. F., Pimenta, D. C., Zukerman-Schpector, J., Manarin, F. & Stefani, H. A. Stereo- and regioselective Cu-catalyzed hydroboration of alkynyl chalcogenoethers. *Chemcatchem* **12**, 3545–3552 (2020).
10. Kadikova, R. N., Ramazanov, I. R., Vyatkin, A. V. & Dzhemilev, U. M. Zirconium-catalyzed alkyne carbo- and cycloalumination reactions in stereoselective preparation of 1-alkenyl selenides. *Synthesis* **49**, 4523–4534 (2017).
11. Gaussian 09, Revision B.0, M J Frisch, G. W Trucks, H B. Schlegel, G. E. Scuseria, M A. Robb,I R. Cheeseman, G. Scalmani, V Barone, B. Mennucci, G. A. Petersson, H Nakatsuji, M Caricato, XLi H P Hratchian, A. F Izmaylov, J. Bloino, G. Zheng, J L. Sonnenberg, M. Hada, M. Ehara, K. ToyotaR. Fukuda, J. Hasegawa, M. Ishida, I: Nakajima, Y Honda, 0. Kitao, H Nakai, T Veven, JI AMontgomery, J. J. E. Peralta, E Ogliaro, M Bearpark, J. J Heyd, E. Brothers, K N Kudin, V NStarovero T. Keith, R. Kobayashi, J. Normand, K. Raghavachari, A. Rendell, J C. Burant, S. S IyengarI Tomasi, M Cossi, N Rega J M Millam, M Klene, J. E. Knox, J B. Cross, V Bakken, C. Adamo, JJaramillo, R. Gomperts, R. E. Stratmann, 0. Yazyev A. J. Austin, R. Cammi, C. Pomelli, J. W. OchterskiR. L. Martin, K. Morokuma, G. Zakrzewski, G. A. Voth, P. Salvador; J. J Dannenberg, S. Dapprich, A.D. Daniels, 0. Farkas, J. B. Foresman, V Ortiz, Cioslowski, and D. J. Fox, Gaussian, Inc..Wallingford C7, 2013.
12. Becke, A. D. Density-functional thermochemistry. III. The role of exact exchange. *J. Chem. Phys*. **98**, 5648–5652 (1993).
13. Lee, C., Yang, W. & Parr, R. G. Development of the Colle-Salvetti correlation-energy formula into a functional of the electron density. *Phys. Rev. B* **37**, 785-789 (1988).
14. Grimme, S., Ehrlich, S. & Goerigk, L. Effect of the damping function in dispersion corrected density

functional theory. *J. Comput. Chem*. **32**, 1456–1465 (2011).
15. Weigend, F. & Ahlrichs, R. Balanced basis sets of split valence, triple zeta valence and quadruple zeta valence quality for H to Rn: Design and assessment of accuracy. *Phys. Chem. Chem. Phys.* **7**, 3297–3305 (2005).
16. Tomasi, J. & Persico, M. Molecular interactions in solution: an overview of methods based on continuous distributions of the solvent. *Chem. Rev*. **94**, 2027–2094 (1994).
17. Fukui, K. Formulation of the reaction coordinate. *J. Phys. Chem*. **74**, 4161–4163 (1970).
18. Fukui, K. The path of chemical reactions - the IRC approach. *Acc. Chem. Res*. **14**, 363–368 (1981).
19. Zhao, Y., Schultz, N. E. & Truhlar, D. G. Design of density functionals by combining the method of constraint satisfaction with parametrization for thermochemistry, thermochemical kinetics, and noncovalent interactions. *J. Chem. Theory. Comput*. **2**, 364 (2006);
20. Zhao, Y. & Truhlar, D. G. A new local density functional for main-group thermochemistry, transition metal bonding, thermochemical kinetics, and noncovalent interactions. *J. Chem. Phys*. **125**, 194101 (2006).
21. Zhao, Y. & Truhlar, D. G. Density functional for spectroscopy: no long-range self-interaction error, good performance for Rydberg and charge-transfer states, and better performance on average than B3LYP for ground states. *J. Phys. Chem. A* **110**, 13126 (2006).
22. Weigend, F. Accurate coulomb-fitting basis sets for H to Rn. *Phys. Chem. Chem. Phys.* **8**, 1057–1065 (2006).
23. Marenich, A. V., Cramer, C. J. & Truhlar, D. G. Universal solvation model based on solute electron density and on a continuum model of the solvent defined by the bulk dielectric constant and atomic surface tensions. *J. Phys. Chem. B* **113**, 6378–6396 (2009).
24. CYLview, 1.0b; C. Y. Legault, Université de Sherbrooke, 2009 (http://www.cylview.org).
25. Lu, T. & Chen, F. Multiwfn-Multiwfn: A multifunctional wavefunction analyzer. *J. Comput. Chem*. **33**, 580–592 (2012).
26. Humphrey, W., Dalke, A. & Schulten, K. VMD: visual molecular dynamics. *J. Mol. Graph*. **14**, 33–38 (1996).
27. Bickelhaupt,F. M., Nibbering,,N. M. M., Van Wezenbeek, E. M. & Baerends, E. J. *J. Phys. Chem*., **96**, 4864−4873 (1992).
28. Ess, D. H., Houk, K. N. *J. Am. Chem. Soc*. **129**, 10646–10647 (2007).
