# Supplementary material for: Diversity-oriented synthesis of stereodefined tetrasubstituted alkenes via a modular alkyne gem-addition strategy
Source: Nat Commun. 2025 Jan 25;16:1025. doi: 10.1038/s41467-025-56184-3 (PMC11763084; doi:10.1038/s41467-025-56184-3)
Supplement: Supplementary file 2 — NCOMMS-24-50497-T-s02 [file 41467_2025_56184_MOESM2_ESM.pdf]

Description of Additional Supplementary Files

File Name: Supplementary Data 1

Description: Cartesian coordinates of all the optimized geometries

File Name: Supplementary Data 2

Description: in vivo experiment data (mouse body weight, tumor volume and tumor weight)

File Name: Supplementary Data 3

Description: in vitro experiment data (the measurement of IC_{50} values)
