# Supplementary material for: Diversity-oriented synthesis of stereodefined tetrasubstituted alkenes via a modular alkyne gem-addition strategy
Source: Nat Commun. 2025 Jan 25;16:1025. doi: 10.1038/s41467-025-56184-3 (PMC11763084; doi:10.1038/s41467-025-56184-3)
Supplement: Supplementary file 3 — NCOMMS-24-50497-T-s03 [file 41467_2025_56184_MOESM3_ESM.pdf]

Cartesian coordinates of all the optimized geometries

$PhBCl_2$

C	-14.262424000000	0.994701000000	-0.263139000000
C	-13.729491000000	-0.247591000000	0.068829000000
C	-14.575563000000	-1.315850000000	0.352573000000
C	-15.950016000000	-1.141218000000	0.304024000000
C	-16.512287000000	0.103520000000	-0.028709000000
C	-15.637208000000	1.166914000000	-0.310931000000
B	-18.035823000000	0.295630000000	-0.082582000000
Cl	-18.764458000000	1.850673000000	-0.496190000000
Cl	-19.154134000000	-1.026329000000	0.266580000000
H	-13.604989000000	1.825505000000	-0.483739000000
H	-12.656090000000	-0.383036000000	0.106386000000
H	-14.161593000000	-2.281818000000	0.610768000000
H	-16.600086000000	-1.976757000000	0.525890000000
H	-16.042775000000	2.135655000000	-0.569758000000

1a

C	-10.970170000000	0.481941000000	-0.133194000000
C	-10.396779000000	-0.741075000000	0.201840000000
C	-11.215579000000	-1.826745000000	0.497619000000
C	-12.595759000000	-1.696445000000	0.460407000000
C	-13.182206000000	-0.466492000000	0.123291000000
C	-12.349053000000	0.623500000000	-0.173752000000
C	-14.594657000000	-0.327274000000	0.083363000000
C	-15.799634000000	-0.204297000000	0.048396000000
S	-17.473777000000	-0.111640000000	0.020162000000
C	-17.731196000000	1.635695000000	-0.437901000000
H	-10.339996000000	1.330938000000	-0.364453000000
H	-9.320479000000	-0.847188000000	0.232202000000
H	-10.777112000000	-2.781090000000	0.759073000000
H	-13.230999000000	-2.540993000000	0.690681000000
H	-12.793491000000	1.574500000000	-0.434462000000
H	-18.810308000000	1.774387000000	-0.476439000000
H	-17.300484000000	2.287885000000	0.317457000000
H	-17.296890000000	1.834395000000	-1.414287000000

1b

C	-10.553067000000	-0.046555000000	-1.216554000000
C	-10.310525000000	-0.920847000000	-0.150928000000
C	-11.385383000000	-1.516073000000	0.512366000000
C	-12.682461000000	-1.234645000000	0.108396000000
C	-12.942159000000	-0.362922000000	-0.955787000000

C -11.845933000000 0.226515000000 -1.611373000000
C -14.273796000000 -0.081141000000 -1.363071000000
C -15.409417000000 0.165650000000 -1.710043000000
S -16.976628000000 0.432542000000 -2.247925000000
C -17.459767000000 1.906789000000 -1.283947000000
H -9.709945000000 0.406682000000 -1.720443000000
H -11.224220000000 -2.193812000000 1.336708000000
H -13.512075000000 -1.697947000000 0.625172000000
H -12.025352000000 0.902721000000 -2.436225000000
H -18.479688000000 2.138050000000 -1.587797000000
H -17.431812000000 1.685026000000 -0.220093000000
H -16.804736000000 2.741256000000 -1.521325000000
O -9.009590000000 -1.127459000000 0.163434000000
C -8.701439000000 -2.009352000000 1.240089000000
H -9.125500000000 -1.646424000000 2.178892000000
H -9.067710000000 -3.018367000000 1.038573000000
H -7.617552000000 -2.023386000000 1.311873000000

1c

C -10.961667000000 0.481414000000 -0.129888000000
C -10.370495000000 -0.735152000000 0.197630000000
C -11.172727000000 -1.832986000000 0.493873000000
C -12.554752000000 -1.720853000000 0.464360000000
C -13.158933000000 -0.497717000000 0.135118000000
C -12.342609000000 0.604447000000 -0.162291000000
C -14.574419000000 -0.377058000000 0.103481000000
C -15.780395000000 -0.268085000000 0.075380000000
Se -17.606543000000 -0.178591000000 0.053338000000
C -17.791872000000 1.713561000000 -0.463790000000
H -10.343977000000 1.339451000000 -0.361342000000
H -9.292703000000 -0.826921000000 0.221846000000
H -10.719879000000 -2.782177000000 0.749418000000
H -13.177702000000 -2.574473000000 0.694718000000
H -12.801550000000 1.550206000000 -0.416816000000
H -18.862467000000 1.898764000000 -0.514492000000
H -17.332950000000 2.339586000000 0.294813000000
H -17.332932000000 1.866587000000 -1.435352000000

IN1A

C 3.448279000000 -0.625229000000 -2.328143000000
C 4.441985000000 -0.133257000000 -1.488006000000
C 4.235641000000 -0.115715000000 -0.112461000000
C 3.047661000000 -0.585077000000 0.424447000000
C 2.043485000000 -1.087292000000 -0.414900000000

C	2.257905000000	-1.101517000000	-1.800915000000
C	0.822947000000	-1.565985000000	0.129058000000
C	-0.216381000000	-1.988944000000	0.585389000000
B	-0.982275000000	1.149918000000	-0.577504000000
Se	-1.743508000000	-2.654684000000	1.333003000000
C	-2.859767000000	-2.687154000000	-0.289103000000
Cl	-1.223190000000	0.481420000000	-2.197203000000
Cl	-2.344103000000	1.014148000000	0.538787000000
C	0.318157000000	1.864015000000	-0.183591000000
C	0.564573000000	2.236550000000	1.147980000000
C	1.743988000000	2.870351000000	1.506359000000
C	2.699778000000	3.157565000000	0.536580000000
C	2.476415000000	2.800384000000	-0.788495000000
C	1.302430000000	2.155134000000	-1.141866000000
H	3.600234000000	-0.636800000000	-3.399656000000
H	5.368738000000	0.240381000000	-1.903135000000
H	5.000033000000	0.276898000000	0.545181000000
H	2.880367000000	-0.557189000000	1.492168000000
H	1.481765000000	-1.478889000000	-2.452061000000
H	-3.817607000000	-3.096457000000	0.024057000000
H	-2.978813000000	-1.674153000000	-0.658524000000
H	-2.395347000000	-3.329093000000	-1.030877000000
H	-0.173982000000	2.016931000000	1.907004000000
H	1.920500000000	3.141557000000	2.539056000000
H	3.621612000000	3.651691000000	0.815677000000
H	3.224635000000	3.010609000000	-1.540630000000
H	1.144434000000	1.865561000000	-2.171000000000

TS1A

C	-12.029773000000	-1.098406000000	-1.890322000000
C	-11.013688000000	-0.884204000000	-0.960714000000
C	-11.294561000000	-0.842976000000	0.402941000000
C	-12.592293000000	-1.016824000000	0.846006000000
C	-13.627021000000	-1.220891000000	-0.084132000000
C	-13.333044000000	-1.261588000000	-1.460278000000
C	-14.953194000000	-1.333605000000	0.349902000000
C	-16.139917000000	-1.268906000000	0.687327000000
B	-16.469654000000	0.763533000000	0.553042000000
Se	-17.657101000000	-2.250530000000	1.135151000000
C	-16.980581000000	-4.016343000000	0.596737000000
Cl	-17.286841000000	0.830105000000	-1.077801000000
Cl	-17.646551000000	0.956062000000	1.945981000000
C	-15.096043000000	1.511880000000	0.709017000000
C	-14.528703000000	1.717343000000	1.973391000000

C	-13.283820000000	2.314589000000	2.120223000000
C	-12.570488000000	2.724060000000	0.998560000000
C	-13.114705000000	2.533329000000	-0.265425000000
C	-14.361431000000	1.935490000000	-0.404472000000
H	-11.801066000000	-1.126340000000	-2.946881000000
H	-9.996233000000	-0.746114000000	-1.302303000000
H	-10.499378000000	-0.669701000000	1.114863000000
H	-12.830944000000	-0.974990000000	1.898540000000
H	-14.138171000000	-1.408573000000	-2.166301000000
H	-17.789063000000	-4.715551000000	0.800754000000
H	-16.748068000000	-4.007463000000	-0.463887000000
H	-16.105880000000	-4.267166000000	1.189388000000
H	-15.068548000000	1.398327000000	2.855340000000
H	-12.867913000000	2.457865000000	3.109613000000
H	-11.597571000000	3.185601000000	1.109642000000
H	-12.566151000000	2.844856000000	-1.145268000000
H	-14.767916000000	1.786202000000	-1.395543000000

IN2A

C	2.554042000000	-2.585524000000	-2.132350000000
C	2.455978000000	-2.112030000000	-3.441248000000
C	1.478919000000	-1.183610000000	-3.805291000000
C	0.593356000000	-0.717563000000	-2.857922000000
C	0.676132000000	-1.192976000000	-1.527355000000
C	1.670377000000	-2.135651000000	-1.174513000000
C	-0.188532000000	-0.708954000000	-0.567824000000
C	-0.920194000000	-0.186798000000	0.318762000000
B	-0.189910000000	1.169127000000	1.052121000000
Se	-2.659327000000	-0.736571000000	0.847943000000
C	-2.941177000000	-2.160050000000	-0.471809000000
Cl	-1.236687000000	2.636026000000	0.518336000000
Cl	-0.377850000000	0.904239000000	2.904351000000
C	1.335117000000	1.270632000000	0.584897000000
C	1.700794000000	1.974287000000	-0.567998000000
C	2.994310000000	1.919140000000	-1.075412000000
C	3.963358000000	1.156160000000	-0.434464000000
C	3.627478000000	0.459869000000	0.722006000000
C	2.331974000000	0.520091000000	1.220594000000
H	3.322428000000	-3.299480000000	-1.871405000000
H	3.153360000000	-2.468427000000	-4.187955000000
H	1.424251000000	-0.826721000000	-4.824080000000
H	-0.163647000000	0.012136000000	-3.106979000000
H	1.728174000000	-2.477653000000	-0.151523000000
H	-2.905009000000	-1.746046000000	-1.475678000000

H	-2.194642000000	-2.938724000000	-0.341472000000
H	-3.932857000000	-2.557585000000	-0.267535000000
H	0.955644000000	2.563743000000	-1.085878000000
H	3.244390000000	2.469422000000	-1.974056000000
H	4.970458000000	1.107500000000	-0.828819000000
H	4.374964000000	-0.134282000000	1.233119000000
H	2.084979000000	-0.036614000000	2.115361000000

TS2A

C	2.188841000000	-3.307221000000	-1.398439000000
C	2.051179000000	-2.920566000000	-2.732975000000
C	1.108239000000	-1.966400000000	-3.119817000000
C	0.293233000000	-1.388146000000	-2.171197000000
C	0.420173000000	-1.773051000000	-0.814495000000
C	1.380346000000	-2.742452000000	-0.436341000000
C	-0.362140000000	-1.171820000000	0.145140000000
C	-1.038532000000	-0.563216000000	1.021129000000
B	-0.432903000000	0.769909000000	1.789010000000
Se	-2.796495000000	-1.325486000000	1.394042000000
C	-3.574746000000	-1.163366000000	-0.402217000000
Cl	-1.591182000000	2.191685000000	1.356914000000
Cl	-0.562216000000	0.395634000000	3.634781000000
C	1.070200000000	0.994136000000	1.285940000000
C	1.343721000000	1.727123000000	0.124677000000
C	2.621986000000	1.779859000000	-0.420039000000
C	3.668659000000	1.095422000000	0.187797000000
C	3.423345000000	0.366575000000	1.346592000000
C	2.141831000000	0.319686000000	1.883698000000
H	2.929492000000	-4.044278000000	-1.122358000000
H	2.690977000000	-3.367343000000	-3.482539000000
H	1.023089000000	-1.679716000000	-4.158369000000
H	-0.438945000000	-0.639749000000	-2.438336000000
H	1.468649000000	-3.015688000000	0.605178000000
H	-3.454923000000	-0.140496000000	-0.746290000000
H	-3.116943000000	-1.871211000000	-1.085892000000
H	-4.631093000000	-1.395123000000	-0.278324000000
H	0.539160000000	2.260713000000	-0.364682000000
H	2.800798000000	2.354858000000	-1.320363000000
H	4.665025000000	1.132934000000	-0.234195000000
H	4.231078000000	-0.167371000000	1.832015000000
H	1.967121000000	-0.257806000000	2.782329000000

IN3A

C	0.704290000000	-3.569682000000	-2.757636000000

C	1.901988000000	-3.030868000000	-3.215357000000
C	2.378039000000	-1.833421000000	-2.686376000000
C	1.663462000000	-1.170488000000	-1.702410000000
C	0.456030000000	-1.715978000000	-1.241201000000
C	-0.020406000000	-2.916110000000	-1.771814000000
C	-0.263767000000	-1.006608000000	-0.219505000000
C	-0.399736000000	-0.014623000000	0.582623000000
B	-0.023569000000	1.372677000000	1.253403000000
Se	-1.960248000000	-1.302847000000	0.732716000000
C	-3.241149000000	-0.508108000000	-0.527097000000
Cl	-1.443095000000	2.574840000000	0.887714000000
Cl	0.093104000000	1.112414000000	3.119452000000
C	1.364936000000	1.790178000000	0.569917000000
C	1.394720000000	2.491603000000	-0.641334000000
C	2.588127000000	2.726432000000	-1.315176000000
C	3.788567000000	2.255940000000	-0.793749000000
C	3.782762000000	1.556076000000	0.408200000000
C	2.585153000000	1.326205000000	1.076107000000
H	0.334519000000	-4.499513000000	-3.168098000000
H	2.465376000000	-3.542842000000	-3.984110000000
H	3.309793000000	-1.414726000000	-3.042359000000
H	2.027329000000	-0.240589000000	-1.288628000000
H	-0.951522000000	-3.330741000000	-1.409269000000
H	-4.226290000000	-0.663223000000	-0.092933000000
H	-3.013858000000	0.550551000000	-0.603000000000
H	-3.154377000000	-1.014796000000	-1.482865000000
H	0.468015000000	2.856425000000	-1.064567000000
H	2.580678000000	3.273833000000	-2.249558000000
H	4.718950000000	2.433694000000	-1.317909000000
H	4.711512000000	1.186121000000	0.824496000000
H	2.596324000000	0.773820000000	2.006627000000

TS3A

C	1.196092000000	-3.040596000000	-0.895441000000
C	1.732894000000	-3.428597000000	-2.117966000000
C	1.563947000000	-2.618467000000	-3.236733000000
C	0.863755000000	-1.425307000000	-3.134649000000
C	0.344648000000	-1.020760000000	-1.902508000000
C	0.504276000000	-1.842729000000	-0.785852000000
C	-0.362111000000	0.258602000000	-1.784770000000
C	-0.312811000000	1.224207000000	-0.918723000000
B	-0.102797000000	2.381839000000	0.000658000000
Se	-1.676817000000	0.817321000000	-3.111517000000
C	-2.966440000000	-0.624566000000	-2.778422000000

Cl	0.550011000000	3.943666000000	-0.746272000000
Cl	-1.390645000000	2.693685000000	1.285629000000
C	1.100877000000	1.299553000000	0.467404000000
C	0.922232000000	0.502712000000	1.609450000000
C	1.969982000000	-0.253524000000	2.107121000000
C	3.194784000000	-0.265801000000	1.444610000000
C	3.375098000000	0.487868000000	0.287980000000
C	2.334691000000	1.260215000000	-0.200337000000
H	1.312781000000	-3.672145000000	-0.024727000000
H	2.273807000000	-4.362012000000	-2.201403000000
H	1.975436000000	-2.917933000000	-4.191527000000
H	0.722252000000	-0.798148000000	-4.004163000000
H	0.081493000000	-1.545139000000	0.161412000000
H	-2.475534000000	-1.584233000000	-2.912825000000
H	-3.370240000000	-0.524847000000	-1.775326000000
H	-3.754824000000	-0.496922000000	-3.517091000000
H	-0.034429000000	0.500350000000	2.112205000000
H	1.831241000000	-0.845084000000	3.002276000000
H	4.007466000000	-0.870189000000	1.825941000000
H	4.324277000000	0.468860000000	-0.230548000000
H	2.469750000000	1.844469000000	-1.099948000000

E

C	1.129569000000	-4.061265000000	-0.499828000000
C	1.386447000000	-4.326991000000	-1.842116000000
C	1.174698000000	-3.335151000000	-2.793454000000
C	0.713401000000	-2.084113000000	-2.406478000000
C	0.471616000000	-1.800732000000	-1.058779000000
C	0.674001000000	-2.811346000000	-0.110009000000
C	-0.019937000000	-0.491950000000	-0.630387000000
C	0.231753000000	0.287817000000	0.439878000000
B	-0.808466000000	1.478153000000	0.419974000000
Se	-1.336840000000	0.485212000000	-1.674373000000
C	-2.911570000000	-0.644449000000	-1.384746000000
Cl	-0.196314000000	3.162316000000	0.159218000000
Cl	-2.273982000000	1.363974000000	1.486614000000
C	1.304014000000	0.123488000000	1.430537000000
C	1.097055000000	0.522853000000	2.756257000000
C	2.105383000000	0.393655000000	3.702519000000
C	3.345180000000	-0.118083000000	3.337035000000
C	3.571486000000	-0.496478000000	2.016106000000
C	2.562479000000	-0.377802000000	1.073049000000
H	1.278153000000	-4.833066000000	0.244131000000
H	1.740774000000	-5.303633000000	-2.144585000000

H	1.366356000000	-3.535876000000	-3.839397000000
H	0.546345000000	-1.316541000000	-3.150947000000
H	0.468773000000	-2.609196000000	0.931955000000
H	-2.674576000000	-1.644801000000	-1.736697000000
H	-3.149205000000	-0.642244000000	-0.325149000000
H	-3.723055000000	-0.218242000000	-1.968474000000
H	0.136103000000	0.924866000000	3.047684000000
H	1.923731000000	0.698596000000	4.724973000000
H	4.133441000000	-0.212835000000	4.072453000000
H	4.539513000000	-0.880073000000	1.720486000000
H	2.749453000000	-0.663576000000	0.047389000000

IN1B

C	1.448117000000	-2.868991000000	3.735887000000
C	2.749278000000	-2.407582000000	3.912857000000
C	3.372183000000	-1.685135000000	2.899777000000
C	2.701582000000	-1.424048000000	1.714457000000
C	1.388264000000	-1.878358000000	1.529711000000
C	0.768799000000	-2.605472000000	2.556346000000
C	0.685806000000	-1.585096000000	0.328321000000
C	0.082266000000	-1.326368000000	-0.689198000000
B	0.018737000000	1.643998000000	1.033779000000
Se	-0.833745000000	-0.878408000000	-2.208752000000
C	-0.919378000000	-2.676516000000	-3.016358000000
Cl	-0.340229000000	1.003742000000	2.638167000000
Cl	1.715269000000	1.991751000000	0.686037000000
C	-1.091710000000	1.936263000000	0.010315000000
C	-0.799851000000	2.543227000000	-1.222445000000
C	-1.801026000000	2.813047000000	-2.142869000000
C	-3.119161000000	2.476483000000	-1.852950000000
C	-3.432459000000	1.870978000000	-0.639689000000
C	-2.430441000000	1.606579000000	0.280281000000
H	0.960239000000	-3.430382000000	4.521969000000
H	3.275246000000	-2.609925000000	4.836673000000
H	4.383569000000	-1.324362000000	3.034033000000
H	3.179942000000	-0.859468000000	0.926664000000
H	-0.246745000000	-2.951160000000	2.419378000000
H	-1.463020000000	-3.343649000000	-2.354714000000
H	-1.456947000000	-2.550185000000	-3.953741000000
H	0.086893000000	-3.038570000000	-3.202093000000
H	0.223235000000	2.801741000000	-1.458641000000
H	-1.555406000000	3.280326000000	-3.087400000000
H	-3.901194000000	2.683248000000	-2.572242000000
H	-4.456949000000	1.604610000000	-0.415307000000

H -2.681727000000 1.133605000000 1.219736000000

TS1B

C	-14.171055000000	-2.928348000000	4.499309000000
C	-12.778390000000	-2.960696000000	4.538956000000
C	-12.025374000000	-2.463647000000	3.477023000000
C	-12.660946000000	-1.931187000000	2.370156000000
C	-14.067068000000	-1.893716000000	2.320083000000
C	-14.817799000000	-2.398792000000	3.397824000000
C	-14.712159000000	-1.340127000000	1.207602000000
C	-15.244397000000	-0.719967000000	0.281961000000
B	-15.174864000000	1.215862000000	0.959304000000
Se	-15.942891000000	-0.857112000000	-1.438868000000
C	-15.372142000000	-2.704891000000	-1.793394000000
Cl	-15.127405000000	1.240027000000	2.787310000000
Cl	-13.602804000000	1.764357000000	0.211976000000
C	-16.518704000000	1.719475000000	0.312993000000
C	-16.535348000000	2.421268000000	-0.898743000000
C	-17.730590000000	2.838950000000	-1.473594000000
C	-18.939833000000	2.551387000000	-0.853032000000
C	-18.946507000000	1.849306000000	0.349748000000
C	-17.751241000000	1.441834000000	0.922421000000
H	-14.745807000000	-3.314357000000	5.330030000000
H	-12.276699000000	-3.374611000000	5.403758000000
H	-10.945137000000	-2.490814000000	3.519176000000
H	-12.093910000000	-1.534048000000	1.540062000000
H	-15.896886000000	-2.358922000000	3.352216000000
H	-15.836154000000	-3.373484000000	-1.074296000000
H	-15.719269000000	-2.932314000000	-2.799371000000
H	-14.289071000000	-2.766347000000	-1.745172000000
H	-15.600837000000	2.646389000000	-1.394224000000
H	-17.716250000000	3.387078000000	-2.407068000000
H	-19.871808000000	2.871770000000	-1.300994000000
H	-19.885082000000	1.621514000000	0.838906000000
H	-17.768884000000	0.895904000000	1.856664000000

IN2B

C	3.959142000000	-1.381338000000	-2.817412000000
C	5.041599000000	-1.124016000000	-1.974464000000
C	4.851289000000	-0.694291000000	-0.659715000000
C	3.572490000000	-0.519014000000	-0.176502000000
C	2.463784000000	-0.775018000000	-1.019205000000
C	2.673559000000	-1.208709000000	-2.350672000000
C	1.181377000000	-0.599613000000	-0.545833000000

C	0.008951000000	-0.379775000000	-0.137293000000
B	-0.647051000000	1.170754000000	-0.362611000000
Se	-1.123323000000	-1.653693000000	0.716112000000
C	-0.032222000000	-3.262880000000	0.459091000000
Cl	0.742652000000	2.367265000000	-0.827341000000
Cl	-1.803127000000	0.993011000000	-1.831144000000
C	-1.367803000000	1.589637000000	0.996047000000
C	-2.751495000000	1.486318000000	1.174233000000
C	-3.351093000000	1.792311000000	2.392581000000
C	-2.574652000000	2.201993000000	3.469084000000
C	-1.194293000000	2.304976000000	3.316879000000
C	-0.604614000000	2.000275000000	2.097954000000
H	4.130143000000	-1.711168000000	-3.832412000000
H	6.048269000000	-1.258734000000	-2.347802000000
H	5.704702000000	-0.498944000000	-0.025836000000
H	3.394742000000	-0.183543000000	0.835166000000
H	1.816527000000	-1.394370000000	-2.982118000000
H	-0.602834000000	-4.076462000000	0.901458000000
H	0.114092000000	-3.443828000000	-0.602207000000
H	0.918804000000	-3.153609000000	0.973105000000
H	-3.369581000000	1.168389000000	0.345432000000
H	-4.425764000000	1.710639000000	2.498808000000
H	-3.038196000000	2.440301000000	4.418042000000
H	-0.579770000000	2.623933000000	4.149679000000
H	0.469862000000	2.085853000000	1.994636000000

TS2B

C	3.956633000000	-1.719208000000	-2.566642000000
C	4.990160000000	-1.438247000000	-1.671523000000
C	4.735980000000	-0.857977000000	-0.426877000000
C	3.441885000000	-0.552158000000	-0.066724000000
C	2.382372000000	-0.833269000000	-0.963860000000
C	2.655235000000	-1.422953000000	-2.222652000000
C	1.085243000000	-0.532632000000	-0.613080000000
C	-0.110614000000	-0.269077000000	-0.307209000000
B	-0.949804000000	1.130689000000	-0.489335000000
Se	-0.987949000000	-1.771869000000	0.595337000000
C	-0.605573000000	-3.205161000000	-0.690333000000
Cl	0.256860000000	2.429549000000	-1.181178000000
Cl	-2.263818000000	0.777038000000	-1.791524000000
C	-1.537793000000	1.558817000000	0.932482000000
C	-2.886338000000	1.411295000000	1.271491000000
C	-3.355699000000	1.733571000000	2.541981000000
C	-2.479088000000	2.203295000000	3.511895000000

C	-1.129635000000	2.348566000000	3.200809000000
C	-0.671279000000	2.027978000000	1.930146000000
H	4.177627000000	-2.166217000000	-3.525510000000
H	6.009491000000	-1.673433000000	-1.948079000000
H	5.552951000000	-0.648060000000	0.248830000000
H	3.215144000000	-0.100175000000	0.888213000000
H	1.835198000000	-1.626488000000	-2.896325000000
H	-1.235635000000	-4.037534000000	-0.382260000000
H	-0.887020000000	-2.871621000000	-1.684739000000
H	0.439266000000	-3.496361000000	-0.647667000000
H	-3.580180000000	1.043569000000	0.527686000000
H	-4.407300000000	1.615832000000	2.772955000000
H	-2.841221000000	2.454003000000	4.500992000000
H	-0.436986000000	2.712882000000	3.949526000000
H	0.380768000000	2.149317000000	1.702575000000

IN3B

C	4.308820000000	1.561281000000	0.556540000000
C	4.950715000000	0.899593000000	1.600789000000
C	4.391328000000	-0.250133000000	2.147907000000
C	3.191727000000	-0.742536000000	1.655239000000
C	2.547436000000	-0.079469000000	0.609348000000
C	3.110096000000	1.079868000000	0.056897000000
C	1.301058000000	-0.551874000000	0.072231000000
C	0.305407000000	-0.463045000000	-0.731017000000
Se	0.103384000000	-2.051661000000	0.514699000000
C	-0.958775000000	-1.279840000000	1.976558000000
C	-2.118256000000	0.408414000000	-0.987325000000
B	-0.746443000000	0.128097000000	-1.763938000000
Cl	0.084912000000	1.709222000000	-2.411615000000
Cl	-0.927280000000	-1.110490000000	-3.179760000000
C	-3.123668000000	-0.561986000000	-0.910399000000
C	-4.250970000000	-0.379605000000	-0.116325000000
C	-4.399863000000	0.785441000000	0.627702000000
C	-3.414383000000	1.765193000000	0.565127000000
C	-2.290078000000	1.573730000000	-0.229846000000
H	4.744770000000	2.455121000000	0.130888000000
H	4.889829000000	-0.764516000000	2.958286000000
H	2.752057000000	-1.637580000000	2.074351000000
H	2.602080000000	1.585753000000	-0.752917000000
H	-1.500638000000	-0.425831000000	1.582444000000
H	-1.647435000000	-2.061763000000	2.288422000000
H	-0.292566000000	-1.002525000000	2.787148000000
H	-3.021442000000	-1.475750000000	-1.480936000000

H	-5.013230000000	-1.148009000000	-0.078440000000
H	-5.275453000000	0.930017000000	1.247749000000
H	-3.520376000000	2.678079000000	1.138052000000
H	-1.528643000000	2.342330000000	-0.261302000000
H	5.886740000000	1.280942000000	1.986602000000

TS3B

C	4.019264000000	0.722390000000	-0.118320000000
C	4.727763000000	-0.271476000000	0.550345000000
C	4.076049000000	-1.426671000000	0.969582000000
C	2.720912000000	-1.594506000000	0.720967000000
C	2.009465000000	-0.600798000000	0.048495000000
C	2.664848000000	0.563601000000	-0.368531000000
C	0.570690000000	-0.759864000000	-0.235749000000
C	-0.029922000000	-0.243755000000	-1.254176000000
Se	-0.612567000000	-1.789144000000	0.912683000000
C	-0.536815000000	-0.563910000000	2.440468000000
C	-2.234042000000	0.054322000000	-1.628411000000
B	-0.824939000000	0.438814000000	-2.356967000000
Cl	-0.477392000000	2.282643000000	-2.410239000000
Cl	-0.589905000000	-0.332232000000	-4.039217000000
C	-2.921200000000	-1.118484000000	-1.969217000000
C	-4.113138000000	-1.445740000000	-1.341278000000
C	-4.618706000000	-0.625820000000	-0.336298000000
C	-3.935152000000	0.531401000000	0.028872000000
C	-2.747526000000	0.862346000000	-0.603765000000
H	4.520692000000	1.625879000000	-0.438697000000
H	4.623386000000	-2.199722000000	1.492341000000
H	2.210357000000	-2.490779000000	1.045052000000
H	2.106166000000	1.342627000000	-0.870680000000
H	-1.013909000000	0.373140000000	2.167821000000
H	-1.096911000000	-1.053673000000	3.234578000000
H	0.495435000000	-0.413874000000	2.742281000000
H	-2.520572000000	-1.766267000000	-2.736340000000
H	-4.644413000000	-2.344563000000	-1.626130000000
H	-5.542250000000	-0.888803000000	0.163158000000
H	-4.328451000000	1.170386000000	0.808818000000
H	-2.212727000000	1.755647000000	-0.311929000000
H	5.783621000000	-0.143318000000	0.748729000000

Z

C	3.679868000000	-1.493729000000	-1.512304000000
C	4.564583000000	-1.408142000000	-0.442411000000
C	4.171386000000	-0.767916000000	0.727945000000

C	2.899562000000	-0.216780000000	0.827131000000
C	2.005783000000	-0.298453000000	-0.240762000000
C	2.408525000000	-0.942439000000	-1.410940000000
C	0.633002000000	0.289636000000	-0.129706000000
C	-0.361731000000	-0.512001000000	0.340860000000
B	0.464344000000	1.749307000000	-0.552651000000
Se	0.087174000000	-2.308459000000	0.825485000000
C	-1.657698000000	-3.007353000000	1.384485000000
Cl	1.871202000000	2.626490000000	-1.197463000000
Cl	-1.019103000000	2.705256000000	-0.461750000000
C	-1.780020000000	-0.143967000000	0.513693000000
C	-2.679457000000	-0.311532000000	-0.538359000000
C	-4.018273000000	0.016163000000	-0.370969000000
C	-4.470680000000	0.504277000000	0.850247000000
C	-3.578770000000	0.656660000000	1.906739000000
C	-2.240017000000	0.328097000000	1.742366000000
H	3.979727000000	-1.989701000000	-2.426517000000
H	5.555356000000	-1.836591000000	-0.520966000000
H	4.855032000000	-0.696852000000	1.564161000000
H	2.592793000000	0.281758000000	1.737962000000
H	1.719193000000	-1.008968000000	-2.243169000000
H	-1.465078000000	-4.045885000000	1.645489000000
H	-2.025278000000	-2.465538000000	2.249690000000
H	-2.367054000000	-2.952643000000	0.565082000000
H	-2.322972000000	-0.687202000000	-1.488514000000
H	-4.707843000000	-0.107366000000	-1.195831000000
H	-5.513764000000	0.761664000000	0.979134000000
H	-3.925296000000	1.034047000000	2.859976000000
H	-1.543000000000	0.448543000000	2.561373000000

IN1C

C	1.087144000000	-4.561862000000	-0.376048000000
C	0.388140000000	-5.130491000000	0.684656000000
C	0.004314000000	-4.336782000000	1.761330000000
C	0.312255000000	-2.985120000000	1.781254000000
C	1.011463000000	-2.403914000000	0.713446000000
C	1.397884000000	-3.210766000000	-0.366800000000
C	1.297066000000	-1.013849000000	0.713965000000
C	1.526861000000	0.175553000000	0.719096000000
B	-1.829241000000	-0.097222000000	-0.121428000000
Se	1.892793000000	1.962584000000	0.680604000000
C	0.964467000000	2.463355000000	2.342801000000
Cl	-1.835859000000	-1.571722000000	-1.088791000000
Cl	-2.285928000000	-0.263537000000	1.578743000000

C	-1.497379000000	1.272673000000	-0.733933000000
C	-1.756968000000	2.462028000000	-0.035348000000
C	-1.450256000000	3.697728000000	-0.584116000000
C	-0.865781000000	3.770814000000	-1.843999000000
C	-0.595392000000	2.604950000000	-2.554167000000
C	-0.913995000000	1.372073000000	-2.007255000000
H	1.388248000000	-5.173354000000	-1.216753000000
H	0.144086000000	-6.184533000000	0.672006000000
H	-0.540241000000	-4.772390000000	2.589010000000
H	0.006004000000	-2.365859000000	2.612657000000
H	1.931809000000	-2.766038000000	-1.195516000000
H	1.459612000000	1.998904000000	3.189612000000
H	1.034974000000	3.546881000000	2.404325000000
H	-0.072908000000	2.154685000000	2.271374000000
H	-2.208914000000	2.414447000000	0.946122000000
H	-1.661909000000	4.603079000000	-0.030419000000
H	-0.619276000000	4.734307000000	-2.271404000000
H	-0.136680000000	2.660452000000	-3.532706000000
H	-0.699921000000	0.470543000000	-2.565071000000

TS1C

C	0.877762000000	-5.892029000000	-0.887719000000
C	0.433548000000	-6.373125000000	0.339275000000
C	-0.081616000000	-5.489338000000	1.281384000000
C	-0.157330000000	-4.131734000000	1.001310000000
C	0.290397000000	-3.648669000000	-0.229964000000
C	0.805034000000	-4.536377000000	-1.177127000000
C	0.227402000000	-2.228008000000	-0.521990000000
C	0.849083000000	-1.159118000000	-0.429315000000
B	-1.547243000000	-1.631545000000	-1.400498000000
Se	1.643044000000	0.435021000000	-0.426467000000
C	1.010246000000	1.099116000000	1.317873000000
Cl	-1.561346000000	-2.781984000000	-2.819462000000
Cl	-2.691771000000	-2.126040000000	-0.063819000000
C	-1.496533000000	-0.098704000000	-1.751198000000
C	-1.941687000000	0.872398000000	-0.846386000000
C	-1.848888000000	2.229076000000	-1.133057000000
C	-1.308483000000	2.650571000000	-2.342502000000
C	-0.864362000000	1.704030000000	-3.259288000000
C	-0.958111000000	0.350052000000	-2.963933000000
H	1.280690000000	-6.573577000000	-1.625387000000
H	0.488470000000	-7.430987000000	0.559950000000
H	-0.428078000000	-5.856099000000	2.238729000000
H	-0.562748000000	-3.444079000000	1.729275000000

H	1.142252000000	-4.161500000000	-2.132707000000
H	1.373024000000	0.442233000000	2.100851000000
H	1.436666000000	2.094135000000	1.417139000000
H	-0.072663000000	1.144879000000	1.290648000000
H	-2.368510000000	0.560645000000	0.097804000000
H	-2.201263000000	2.957010000000	-0.413040000000
H	-1.236713000000	3.706539000000	-2.569883000000
H	-0.443556000000	2.021122000000	-4.205181000000
H	-0.604719000000	-0.373319000000	-3.686658000000

IN2C

C	4.366700000000	0.606821000000	-1.522459000000
C	5.030427000000	-0.514851000000	-1.040786000000
C	4.322935000000	-1.485901000000	-0.337261000000
C	2.963808000000	-1.332196000000	-0.122623000000
C	2.285011000000	-0.203887000000	-0.605147000000
C	3.004405000000	0.766936000000	-1.305621000000
C	0.836981000000	-0.071836000000	-0.358737000000
C	0.077701000000	-0.988447000000	0.117280000000
B	-0.100806000000	1.331595000000	-0.618632000000
Se	-1.036108000000	-2.220205000000	0.651817000000
C	-0.957956000000	-1.966287000000	2.607881000000
Cl	0.800934000000	2.706511000000	0.304991000000
Cl	-0.032605000000	1.638941000000	-2.478737000000
C	-1.604181000000	1.159027000000	-0.094831000000
C	-2.629157000000	0.701124000000	-0.929845000000
C	-3.899374000000	0.420448000000	-0.439173000000
C	-4.181888000000	0.596657000000	0.910772000000
C	-3.185375000000	1.069029000000	1.758190000000
C	-1.918503000000	1.345592000000	1.257891000000
H	4.910462000000	1.365825000000	-2.069242000000
H	6.092855000000	-0.632586000000	-1.209248000000
H	4.831595000000	-2.360911000000	0.045350000000
H	2.420868000000	-2.087046000000	0.431295000000
H	2.500625000000	1.639782000000	-1.685660000000
H	-1.609071000000	-2.730884000000	3.023680000000
H	0.067521000000	-2.101900000000	2.933531000000
H	-1.331309000000	-0.970810000000	2.822088000000
H	-2.425691000000	0.548553000000	-1.981276000000
H	-4.669006000000	0.061427000000	-1.111230000000
H	-5.169485000000	0.377658000000	1.296466000000
H	-3.395127000000	1.221776000000	2.809763000000
H	-1.153953000000	1.708678000000	1.932583000000

TS2C

C	4.160994000000	0.129349000000	-1.186167000000
C	4.595808000000	-1.148202000000	-0.851507000000
C	3.697691000000	-2.066464000000	-0.312655000000
C	2.373913000000	-1.710220000000	-0.109030000000
C	1.923224000000	-0.425744000000	-0.443069000000
C	2.835683000000	0.488654000000	-0.984023000000
C	0.544263000000	0.002958000000	-0.247285000000
C	-0.512786000000	-0.628942000000	0.233957000000
B	-0.227963000000	1.375552000000	-0.498724000000
Se	-1.135788000000	-2.224450000000	0.931281000000
C	-0.725502000000	-1.889427000000	2.825543000000
Cl	0.430802000000	2.786510000000	0.554938000000
Cl	-0.271478000000	1.884515000000	-2.308049000000
C	-1.695949000000	0.817280000000	0.036099000000
C	-2.651884000000	0.289280000000	-0.879047000000
C	-3.898108000000	-0.111996000000	-0.456909000000
C	-4.249746000000	0.039340000000	0.886444000000
C	-3.380888000000	0.649669000000	1.793292000000
C	-2.134103000000	1.052942000000	1.370746000000
H	4.854481000000	0.846747000000	-1.605091000000
H	5.629228000000	-1.428835000000	-1.008501000000
H	4.032744000000	-3.061703000000	-0.050726000000
H	1.682147000000	-2.428682000000	0.307902000000
H	2.496300000000	1.480782000000	-1.244207000000
H	-1.031553000000	-2.785843000000	3.360137000000
H	0.339802000000	-1.716957000000	2.938712000000
H	-1.303566000000	-1.033296000000	3.160547000000
H	-2.354514000000	0.152469000000	-1.909357000000
H	-4.598006000000	-0.555833000000	-1.151566000000
H	-5.219428000000	-0.302324000000	1.225366000000
H	-3.685889000000	0.791747000000	2.821147000000
H	-1.443132000000	1.510264000000	2.065217000000

IN1A'

C	3.051130000000	-0.882574000000	-2.259601000000
C	4.038916000000	-0.409498000000	-1.401856000000
C	3.820002000000	-0.417370000000	-0.028141000000
C	2.626051000000	-0.892942000000	0.489461000000
C	1.627678000000	-1.376830000000	-0.367974000000
C	1.854802000000	-1.365470000000	-1.752257000000
C	0.401178000000	-1.859849000000	0.155556000000
C	-0.643576000000	-2.288403000000	0.595549000000
B	-1.360014000000	0.875385000000	-0.456967000000

S	-2.052825000000	-2.893483000000	1.267723000000
C	-3.138005000000	-2.984964000000	-0.195338000000
Cl	-1.620498000000	0.260601000000	-2.095299000000
Cl	-2.706022000000	0.695501000000	0.672400000000
C	-0.057556000000	1.582855000000	-0.057862000000
C	0.198589000000	1.927410000000	1.279388000000
C	1.380156000000	2.554561000000	1.642336000000
C	2.329072000000	2.861841000000	0.672002000000
C	2.096415000000	2.531801000000	-0.658532000000
C	0.919726000000	1.894345000000	-1.016929000000
H	3.212380000000	-0.874500000000	-3.329810000000
H	4.970305000000	-0.030780000000	-1.801668000000
H	4.579428000000	-0.039438000000	0.643722000000
H	2.449277000000	-0.883513000000	1.555972000000
H	1.083862000000	-1.728474000000	-2.417640000000
H	-4.084329000000	-3.385004000000	0.164779000000
H	-3.287017000000	-1.991517000000	-0.608640000000
H	-2.709938000000	-3.654346000000	-0.937002000000
H	-0.534265000000	1.691504000000	2.039042000000
H	1.563792000000	2.804669000000	2.679104000000
H	3.252755000000	3.350467000000	0.954651000000
H	2.839389000000	2.757513000000	-1.411411000000
H	0.754308000000	1.626420000000	-2.050752000000

TS1A'

C	-12.017237000000	-1.101016000000	-1.879063000000
C	-11.005002000000	-0.907271000000	-0.940249000000
C	-11.292145000000	-0.878014000000	0.422764000000
C	-12.593275000000	-1.044647000000	0.856702000000
C	-13.625757000000	-1.228300000000	-0.082294000000
C	-13.323901000000	-1.255398000000	-1.458623000000
C	-14.951560000000	-1.341016000000	0.340741000000
C	-16.150289000000	-1.280293000000	0.657524000000
B	-16.476182000000	0.693266000000	0.573278000000
S	-17.537604000000	-2.207867000000	1.027896000000
C	-16.944595000000	-3.872981000000	0.600044000000
Cl	-17.331590000000	0.805336000000	-1.045213000000
Cl	-17.632821000000	0.878665000000	1.991728000000
C	-15.112959000000	1.473031000000	0.713367000000
C	-14.528654000000	1.677355000000	1.970050000000
C	-13.290237000000	2.291478000000	2.102808000000
C	-12.599875000000	2.719712000000	0.973912000000
C	-13.162031000000	2.532294000000	-0.282750000000
C	-14.402583000000	1.918124000000	-0.407091000000

H	-11.781792000000	-1.119285000000	-2.934261000000
H	-9.984277000000	-0.775558000000	-1.274531000000
H	-10.498697000000	-0.720666000000	1.140183000000
H	-12.837996000000	-1.013941000000	1.908186000000
H	-14.126932000000	-1.386230000000	-2.170089000000
H	-17.774566000000	-4.546614000000	0.808430000000
H	-16.685867000000	-3.923027000000	-0.455076000000
H	-16.089992000000	-4.139612000000	1.217926000000
H	-15.049869000000	1.343759000000	2.857818000000
H	-12.861334000000	2.432797000000	3.086966000000
H	-11.631860000000	3.194022000000	1.073593000000
H	-12.632408000000	2.859578000000	-1.168533000000
H	-14.822423000000	1.771392000000	-1.393054000000

IN2A'

C	2.071287000000	-2.840203000000	-1.898105000000
C	2.149865000000	-2.281334000000	-3.174608000000
C	1.310575000000	-1.235147000000	-3.560563000000
C	0.383240000000	-0.739246000000	-2.669597000000
C	0.286932000000	-1.297915000000	-1.372646000000
C	1.149050000000	-2.355359000000	-0.995820000000
C	-0.633426000000	-0.801819000000	-0.473122000000
C	-1.418782000000	-0.289893000000	0.378693000000
B	-0.622667000000	0.971579000000	1.234576000000
S	-3.068015000000	-0.674964000000	0.759939000000
C	-3.402098000000	-2.038467000000	-0.388539000000
Cl	-1.997124753099	1.881502681144	2.133601945927
Cl	0.475939921559	0.089130716407	2.475590055934
C	0.190096192996	1.900274704344	0.220863093816
C	-0.428989042378	2.970321791056	-0.435903798459
C	0.216818213264	3.678236597691	-1.442416316696
C	1.510661884462	3.333609100698	-1.817833027038
C	2.152546962771	2.285128572872	-1.167961649900
C	1.498416217904	1.583097803282	-0.162644322514
H	2.735147000000	-3.645961000000	-1.618244000000
H	2.878373000000	-2.663876000000	-3.877407000000
H	1.392679000000	-0.813161000000	-4.552260000000
H	-0.272247000000	0.076871000000	-2.937204000000
H	1.072866000000	-2.761542000000	0.002420000000
H	-3.300029000000	-1.705200000000	-1.419614000000
H	-2.737324000000	-2.877295000000	-0.190951000000
H	-4.431916000000	-2.339229000000	-0.204038000000
H	-1.440033798388	3.243859054729	-0.163726586473
H	-0.291401578731	4.496385312939	-1.937587246123

H	2.015065992706	3.879380235428	-2.605122738713
H	3.162149689789	2.010943865852	-1.447936162092
H	2.007755823037	0.762293878630	0.325079926676

TS2A'

C	2.345597000000	-3.343526000000	-1.271352000000
C	2.333694000000	-2.886268000000	-2.590677000000
C	1.423496000000	-1.917053000000	-3.017014000000
C	0.513868000000	-1.395312000000	-2.123946000000
C	0.512263000000	-1.852556000000	-0.783321000000
C	1.443120000000	-2.834375000000	-0.364047000000
C	-0.368461000000	-1.311244000000	0.122078000000
C	-1.129702000000	-0.744958000000	0.961193000000
B	-0.512688000000	0.582622000000	1.756254000000
S	-2.771627000000	-1.423512000000	1.243799000000
C	-3.639278000000	-0.764647000000	-0.211526000000
Cl	-1.729701000000	1.983998000000	1.430594000000
Cl	-0.556648000000	0.125373000000	3.583119000000
C	0.959293000000	0.875381000000	1.201990000000
C	1.154342000000	1.634964000000	0.041517000000
C	2.402109000000	1.736075000000	-0.562108000000
C	3.497577000000	1.076444000000	-0.014934000000
C	3.331981000000	0.327295000000	1.144751000000
C	2.079783000000	0.232102000000	1.741381000000
H	3.063725000000	-4.090355000000	-0.963999000000
H	3.048031000000	-3.288665000000	-3.296810000000
H	1.437942000000	-1.574732000000	-4.041969000000
H	-0.195303000000	-0.636144000000	-2.420441000000
H	1.433636000000	-3.161421000000	0.665540000000
H	-3.540873000000	0.317124000000	-0.243140000000
H	-3.268176000000	-1.218316000000	-1.127285000000
H	-4.685593000000	-1.034084000000	-0.069590000000
H	0.310179000000	2.146626000000	-0.402590000000
H	2.519637000000	2.327288000000	-1.461953000000
H	4.470402000000	1.149744000000	-0.484402000000
H	4.178567000000	-0.186451000000	1.583355000000
H	1.965828000000	-0.363388000000	2.637909000000

IN3A'

C	0.440915000000	-3.506539000000	-3.014073000000
C	1.636663000000	-2.900663000000	-3.385589000000
C	2.072799000000	-1.750009000000	-2.732324000000
C	1.319442000000	-1.199961000000	-1.708808000000
C	0.114703000000	-1.812957000000	-1.334992000000

C	-0.322551000000	-2.966186000000	-1.989899000000
C	-0.648788000000	-1.227849000000	-0.269717000000
C	-0.840190000000	-0.327000000000	0.633831000000
B	-0.509035000000	0.997242000000	1.442960000000
S	-2.200686000000	-1.585070000000	0.570186000000
C	-3.499147000000	-0.861886000000	-0.475422000000
Cl	-1.939521000000	2.198793000000	1.116129000000
Cl	-0.480093000000	0.561955000000	3.280632000000
C	0.900892000000	1.502395000000	0.875031000000
C	0.978069000000	2.338999000000	-0.244438000000
C	2.198041000000	2.654122000000	-0.833191000000
C	3.378418000000	2.130771000000	-0.316789000000
C	3.325880000000	1.297472000000	0.795667000000
C	2.102118000000	0.988644000000	1.378827000000
H	0.103612000000	-4.399947000000	-3.521765000000
H	2.230212000000	-3.324431000000	-4.184727000000
H	3.003155000000	-1.280653000000	-3.022462000000
H	1.650644000000	-0.306946000000	-1.197245000000
H	-1.251737000000	-3.433550000000	-1.692644000000
H	-3.140762000000	0.080933000000	-0.879056000000
H	-3.740958000000	-1.567754000000	-1.266685000000
H	-4.364110000000	-0.697414000000	0.163970000000
H	0.067873000000	2.747051000000	-0.664001000000
H	2.226730000000	3.305686000000	-1.697756000000
H	4.329326000000	2.370960000000	-0.775120000000
H	4.238616000000	0.885369000000	1.207810000000
H	2.077700000000	0.333748000000	2.240163000000

TS3A'

C	2.263718000000	-2.602352000000	-1.508120000000
C	2.408207000000	-2.739204000000	-2.884812000000
C	1.531254000000	-2.080825000000	-3.740658000000
C	0.516554000000	-1.287813000000	-3.224508000000
C	0.380806000000	-1.131316000000	-1.842609000000
C	1.256223000000	-1.804133000000	-0.987606000000
C	-0.677551000000	-0.266426000000	-1.307986000000
C	-0.764905000000	0.513021000000	-0.266473000000
B	-0.778271000000	1.537984000000	0.821633000000
S	-2.257236000000	-0.077435000000	-2.134347000000
C	-2.930949000000	-1.751723000000	-1.930991000000
Cl	-0.954864000000	3.306629000000	0.298895000000
Cl	-1.764981000000	1.148001000000	2.334889000000
C	0.813606000000	1.007217000000	0.874482000000
C	1.217344000000	0.099167000000	1.866978000000

C	2.557161000000	-0.211511000000	2.029094000000
C	3.507513000000	0.336928000000	1.170834000000
C	3.117865000000	1.206092000000	0.156069000000
C	1.780477000000	1.534997000000	0.005582000000
H	2.934733000000	-3.120972000000	-0.836290000000
H	3.195819000000	-3.361469000000	-3.288876000000
H	1.635562000000	-2.186386000000	-4.812349000000
H	-0.169390000000	-0.779221000000	-3.887784000000
H	1.141620000000	-1.708643000000	0.080654000000
H	-2.260662000000	-2.485254000000	-2.373515000000
H	-3.095071000000	-1.966010000000	-0.877465000000
H	-3.883301000000	-1.757785000000	-2.459076000000
H	0.476845000000	-0.338641000000	2.521008000000
H	2.862619000000	-0.890340000000	2.814214000000
H	4.552111000000	0.080095000000	1.289046000000
H	3.856112000000	1.622797000000	-0.515860000000
H	1.474369000000	2.209242000000	-0.782275000000

E'

C	1.979963000000	-3.190005000000	-1.885585000000
C	1.835415000000	-3.184432000000	-3.270502000000
C	0.982055000000	-2.265445000000	-3.870791000000
C	0.280866000000	-1.353593000000	-3.093323000000
C	0.433869000000	-1.339090000000	-1.703315000000
C	1.283913000000	-2.278775000000	-1.106891000000
C	-0.309250000000	-0.393729000000	-0.871932000000
C	-0.057948000000	0.281330000000	0.272032000000
B	-1.462852000000	0.929830000000	0.616190000000
S	-1.976562000000	0.123897000000	-1.295723000000
C	-2.969777000000	-1.377886000000	-1.097578000000
Cl	-1.597506000000	2.749779000000	0.587341000000
Cl	-2.476262000000	0.143972000000	1.919064000000
C	1.204339000000	0.452887000000	0.990146000000
C	1.184715000000	0.681448000000	2.371950000000
C	2.365693000000	0.867165000000	3.078062000000
C	3.586592000000	0.849711000000	2.413310000000
C	3.618491000000	0.648992000000	1.035424000000
C	2.441373000000	0.451333000000	0.331086000000
H	2.631451000000	-3.912221000000	-1.411209000000
H	2.378724000000	-3.897723000000	-3.876133000000
H	0.860118000000	-2.258734000000	-4.945939000000
H	-0.382951000000	-0.640910000000	-3.565540000000
H	1.390684000000	-2.291524000000	-0.031248000000
H	-2.652100000000	-2.095248000000	-1.851083000000

H	-2.837749000000	-1.776948000000	-0.095824000000
H	-4.007874000000	-1.099992000000	-1.266685000000
H	0.237176000000	0.701725000000	2.893234000000
H	2.331670000000	1.032508000000	4.147071000000
H	4.507151000000	1.001185000000	2.961744000000
H	4.564387000000	0.651889000000	0.509319000000
H	2.475070000000	0.307763000000	-0.739705000000

IN1B'

C	3.509839000000	-0.510680000000	-2.940306000000
C	4.552840000000	-0.667583000000	-2.032490000000
C	4.291066000000	-1.174610000000	-0.763062000000
C	2.999268000000	-1.522595000000	-0.399574000000
C	1.942200000000	-1.361814000000	-1.307368000000
C	2.213838000000	-0.851558000000	-2.585529000000
C	0.612117000000	-1.679185000000	-0.932093000000
C	-0.529570000000	-1.938799000000	-0.619326000000
B	-0.206282000000	1.482502000000	-0.275928000000
S	-2.130417000000	-2.282526000000	-0.277010000000
C	-2.064294000000	-2.528750000000	1.527810000000
Cl	1.513190000000	1.845131000000	-0.115190000000
Cl	-0.873710000000	1.530391000000	-1.911136000000
C	-1.090863000000	1.214503000000	0.953856000000
C	-2.481854000000	1.068307000000	0.828306000000
C	-3.283247000000	0.854826000000	1.939929000000
C	-2.709705000000	0.774192000000	3.204467000000
C	-1.331701000000	0.906742000000	3.353667000000
C	-0.534293000000	1.126524000000	2.241319000000
H	3.706054000000	-0.115089000000	-3.928405000000
H	5.562292000000	-0.396170000000	-2.311867000000
H	5.097558000000	-1.298594000000	-0.052026000000
H	2.794318000000	-1.910878000000	0.588827000000
H	1.400417000000	-0.715673000000	-3.284696000000
H	-3.090635000000	-2.714706000000	1.838974000000
H	-1.441928000000	-3.387631000000	1.765322000000
H	-1.691477000000	-1.629983000000	2.008909000000
H	-2.935151000000	1.123284000000	-0.151233000000
H	-4.353231000000	0.745376000000	1.821770000000
H	-3.333929000000	0.604139000000	4.072262000000
H	-0.883547000000	0.838604000000	4.336200000000
H	0.534849000000	1.231377000000	2.365798000000

TS1B'

C	-12.125403000000	-1.343973000000	-2.138633000000

C	-11.001660000000	-1.153887000000	-1.336129000000
C	-11.130898000000	-0.958313000000	0.038090000000
C	-12.385364000000	-0.951756000000	0.617671000000
C	-13.528799000000	-1.142186000000	-0.182980000000
C	-13.385595000000	-1.338858000000	-1.571009000000
C	-14.801288000000	-1.123870000000	0.389864000000
C	-15.951622000000	-0.972011000000	0.830540000000
B	-16.324138000000	0.944393000000	0.494700000000
S	-17.291560000000	-1.801713000000	1.498589000000
C	-16.757990000000	-3.525325000000	1.276080000000
Cl	-14.761978000000	1.903084000000	0.345647000000
Cl	-17.216626000000	0.844402000000	-1.104404000000
C	-17.171836000000	1.268762000000	1.785437000000
C	-18.571328000000	1.291729000000	1.764665000000
C	-19.305601000000	1.555316000000	2.915651000000
C	-18.653530000000	1.788098000000	4.120300000000
C	-17.261852000000	1.761514000000	4.165296000000
C	-16.534790000000	1.505982000000	3.012246000000
H	-12.011900000000	-1.491603000000	-3.203780000000
H	-10.017236000000	-1.156649000000	-1.785334000000
H	-10.251201000000	-0.809307000000	0.648944000000
H	-12.507653000000	-0.795098000000	1.679995000000
H	-14.270531000000	-1.476426000000	-2.176104000000
H	-17.563235000000	-4.139872000000	1.676062000000
H	-16.617603000000	-3.744833000000	0.220171000000
H	-15.841763000000	-3.712824000000	1.831745000000
H	-19.090903000000	1.105317000000	0.834711000000
H	-20.387164000000	1.576540000000	2.871066000000
H	-19.223321000000	1.989488000000	5.018492000000
H	-16.746369000000	1.941582000000	5.100234000000
H	-15.453629000000	1.488225000000	3.059844000000

IN2B'

C	3.772205000000	-1.546990000000	-2.668478000000
C	4.798126000000	-1.393853000000	-1.734519000000
C	4.523458000000	-1.101858000000	-0.397186000000
C	3.216333000000	-0.960326000000	0.016589000000
C	2.163713000000	-1.111849000000	-0.918128000000
C	2.459527000000	-1.407260000000	-2.270797000000
C	0.853646000000	-0.970050000000	-0.513047000000
C	-0.342838000000	-0.760522000000	-0.164315000000
B	-0.848497000000	0.878921000000	-0.319865000000
S	-1.540512000000	-1.867232000000	0.439903000000
C	-0.680013000000	-3.457366000000	0.302964000000

Cl	0.630337000000	1.979990000000	-0.722527000000
Cl	-2.010103000000	0.870838000000	-1.786544000000
C	-1.520501000000	1.271109000000	1.069602000000
C	-2.905820000000	1.352892000000	1.240970000000
C	-3.466196000000	1.649329000000	2.480026000000
C	-2.648986000000	1.859213000000	3.583666000000
C	-1.266572000000	1.773751000000	3.437862000000
C	-0.715725000000	1.482497000000	2.197933000000
H	4.007997000000	-1.770722000000	-3.699348000000
H	5.826463000000	-1.502076000000	-2.053572000000
H	5.333861000000	-0.985513000000	0.308577000000
H	2.974223000000	-0.729828000000	1.044243000000
H	1.645128000000	-1.514246000000	-2.972990000000
H	-1.382025000000	-4.207931000000	0.662029000000
H	-0.422805000000	-3.666072000000	-0.733630000000
H	0.211597000000	-3.466021000000	0.927286000000
H	-3.555096000000	1.188364000000	0.391642000000
H	-4.542388000000	1.715180000000	2.581773000000
H	-3.082470000000	2.087883000000	4.549048000000
H	-0.620607000000	1.935668000000	4.291913000000
H	0.361031000000	1.420889000000	2.099113000000

TS2B'

C	-1.771462000000	-6.359704000000	9.590723000000
C	-3.162443000000	-6.255608000000	9.656036000000
C	-3.903261000000	-5.728387000000	8.596178000000
C	-3.255954000000	-5.297050000000	7.459233000000
C	-1.844605000000	-5.397519000000	7.378375000000
C	-1.106815000000	-5.936893000000	8.460421000000
C	-1.196050000000	-4.963197000000	6.246672000000
C	-0.654970000000	-4.494184000000	5.205746000000
B	-0.272067000000	-2.865830000000	5.124934000000
S	-0.412785000000	-5.502934000000	3.743351000000
C	-2.142343000000	-5.803960000000	3.272372000000
Cl	-0.501507000000	-2.084745000000	6.835727000000
Cl	1.545982000000	-2.796817000000	4.668451000000
C	-1.262371000000	-2.250860000000	4.036125000000
C	-2.603109000000	-2.007720000000	4.365633000000
C	-3.514689000000	-1.556421000000	3.420367000000
C	-3.106805000000	-1.341062000000	2.106904000000
C	-1.783783000000	-1.581248000000	1.755354000000
C	-0.877681000000	-2.035172000000	2.708869000000
H	-1.220163000000	-6.769602000000	10.425011000000
H	-3.677058000000	-6.589155000000	10.547517000000

H	-4.978984000000	-5.656126000000	8.671268000000
H	-3.801126000000	-4.878845000000	6.625080000000
H	-0.031238000000	-6.002387000000	8.381527000000
H	-2.650419000000	-6.432646000000	3.999508000000
H	-2.658364000000	-4.854321000000	3.149797000000
H	-2.095898000000	-6.319999000000	2.313872000000
H	-2.936037000000	-2.172335000000	5.382926000000
H	-4.543044000000	-1.372295000000	3.706004000000
H	-3.813976000000	-0.989252000000	1.366390000000
H	-1.455614000000	-1.417141000000	0.736355000000
H	0.147200000000	-2.222875000000	2.419097000000

IN3B'

C	-1.173400000000	-3.668001000000	3.075666000000
C	-1.761370000000	-2.942875000000	4.106801000000
C	-1.728983000000	-1.550137000000	4.092048000000
C	-1.111776000000	-0.877910000000	3.050376000000
C	-0.519634000000	-1.610031000000	2.012345000000
C	-0.551825000000	-3.005962000000	2.027296000000
C	0.113629000000	-0.894309000000	0.941271000000
C	0.475052000000	0.188607000000	0.340936000000
B	0.549605000000	1.722109000000	-0.066168000000
S	0.929316000000	-1.345995000000	-0.599500000000
C	-0.409518000000	-1.606661000000	-1.802130000000
Cl	-0.293176000000	2.642314000000	1.361076000000
Cl	2.376498000000	2.204846000000	-0.138606000000
C	-0.206541000000	1.890275000000	-1.468337000000
C	-1.600359000000	2.013448000000	-1.523727000000
C	-2.282834000000	2.012360000000	-2.734945000000
C	-1.583293000000	1.880309000000	-3.929866000000
C	-0.198607000000	1.757356000000	-3.899263000000
C	0.476056000000	1.761074000000	-2.683246000000
H	-1.198472000000	-4.749163000000	3.088574000000
H	-2.245339000000	-3.461975000000	4.923348000000
H	-2.186539000000	-0.988389000000	4.895230000000
H	-1.080373000000	0.203007000000	3.026107000000
H	-0.089349000000	-3.562701000000	1.223268000000
H	-0.777783000000	-2.624193000000	-1.694228000000
H	-1.195029000000	-0.877983000000	-1.620613000000
H	0.019994000000	-1.457020000000	-2.790180000000
H	-2.159794000000	2.110681000000	-0.602186000000
H	-3.361118000000	2.112701000000	-2.746287000000
H	-2.111586000000	1.877138000000	-4.874829000000
H	0.357312000000	1.658559000000	-4.823485000000

H	1.553646000000	1.661339000000	-2.676644000000

TS3B'

C	3.784744000000	1.449075000000	0.695615000000
C	4.517959000000	0.703092000000	1.615205000000
C	3.926643000000	-0.412645000000	2.203294000000
C	2.626818000000	-0.774957000000	1.885400000000
C	1.882299000000	-0.036378000000	0.949966000000
C	2.487505000000	1.090033000000	0.361942000000
C	0.530396000000	-0.381816000000	0.624634000000
C	-0.313014000000	-0.377558000000	-0.472295000000
S	-0.235766000000	-1.942507000000	0.454778000000
C	-1.570161000000	-2.045962000000	1.693275000000
C	-1.895869000000	0.474189000000	-0.384105000000
B	-0.663798000000	0.012238000000	-1.874270000000
Cl	0.024552000000	1.515245000000	-2.585818000000
Cl	-1.420745000000	-1.142810000000	-3.020378000000
C	-3.108973000000	-0.207939000000	-0.415101000000
C	-4.245569000000	0.372131000000	0.136917000000
C	-4.170877000000	1.635722000000	0.709986000000
C	-2.955750000000	2.321876000000	0.737575000000
C	-1.821124000000	1.739028000000	0.200625000000
H	4.229970000000	2.318252000000	0.227151000000
H	5.530344000000	0.986578000000	1.870228000000
H	4.481876000000	-1.003695000000	2.921160000000
H	2.176140000000	-1.638989000000	2.358288000000
H	1.933095000000	1.674117000000	-0.360293000000
H	-1.132905000000	-2.561253000000	2.547675000000
H	-1.885551000000	-1.044808000000	1.967915000000
H	-2.391914000000	-2.629020000000	1.282766000000
H	-3.173882000000	-1.184418000000	-0.875326000000
H	-5.186369000000	-0.162199000000	0.115861000000
H	-5.055813000000	2.088881000000	1.137109000000
H	-2.898335000000	3.306458000000	1.182873000000
H	-0.871906000000	2.256458000000	0.244915000000

IN4B'

C	3.788413000000	1.321456000000	0.403780000000
C	4.689220000000	0.345218000000	0.820793000000
C	4.190880000000	-0.858169000000	1.314384000000
C	2.824606000000	-1.084177000000	1.389083000000
C	1.902422000000	-0.109423000000	0.971911000000
C	2.420608000000	1.103545000000	0.478747000000
C	0.467837000000	-0.265686000000	1.123902000000

C	-0.544866000000	-0.110996000000	0.041378000000
S	-0.296412000000	-1.832215000000	0.766252000000
C	-1.561901000000	-2.044026000000	2.042515000000
C	-1.859396000000	0.535914000000	0.334588000000
B	0.022521000000	-0.006131000000	-1.412062000000
Cl	-0.166052000000	1.510295000000	-2.279656000000
Cl	0.990457000000	-1.265378000000	-2.155157000000
C	-2.976788000000	0.245168000000	-0.449098000000
C	-4.193028000000	0.861498000000	-0.194017000000
C	-4.300367000000	1.786802000000	0.841508000000
C	-3.188639000000	2.085698000000	1.620728000000
C	-1.973400000000	1.457618000000	1.371550000000
H	4.153400000000	2.264586000000	0.015505000000
H	5.755608000000	0.517101000000	0.760822000000
H	4.874469000000	-1.633296000000	1.639097000000
H	2.458451000000	-2.030431000000	1.768614000000
H	1.737787000000	1.883613000000	0.165835000000
H	-1.078421000000	-2.551713000000	2.875428000000
H	-1.930921000000	-1.072417000000	2.354456000000
H	-2.361222000000	-2.658458000000	1.632474000000
H	-2.892901000000	-0.474867000000	-1.254566000000
H	-5.056857000000	0.623165000000	-0.800676000000
H	-5.247698000000	2.271750000000	1.038159000000
H	-3.267174000000	2.806868000000	2.423939000000
H	-1.101577000000	1.667782000000	1.976104000000

TS4B'

C	3.962636000000	0.757343000000	0.187211000000
C	4.814633000000	-0.211701000000	0.707681000000
C	4.259398000000	-1.321210000000	1.344234000000
C	2.885685000000	-1.465397000000	1.452324000000
C	2.009756000000	-0.495766000000	0.930888000000
C	2.585832000000	0.625312000000	0.301078000000
C	0.573589000000	-0.564246000000	1.073756000000
C	-0.380429000000	-0.362386000000	-0.001988000000
S	-0.294004000000	-2.106265000000	1.072974000000
C	-1.382432000000	-2.053713000000	2.518667000000
C	-1.688383000000	0.288318000000	0.240939000000
B	0.155641000000	-0.473217000000	-1.475426000000
Cl	0.109677000000	0.935928000000	-2.520937000000
Cl	0.895393000000	-1.938535000000	-2.078826000000
C	-2.760478000000	0.080787000000	-0.633619000000
C	-3.977887000000	0.704829000000	-0.415244000000
C	-4.139106000000	1.555330000000	0.677158000000

C	-3.078422000000	1.772388000000	1.548421000000
C	-1.859107000000	1.141229000000	1.332645000000
H	4.372130000000	1.629689000000	-0.307490000000
H	4.906424000000	-2.089189000000	1.750617000000
H	2.474203000000	-2.346281000000	1.929323000000
H	1.941872000000	1.409059000000	-0.077809000000
H	-0.781726000000	-1.822123000000	3.395322000000
H	-2.138578000000	-1.283808000000	2.369839000000
H	-1.856978000000	-3.031181000000	2.603020000000
H	-2.641765000000	-0.584109000000	-1.480704000000
H	-4.802450000000	0.531883000000	-1.094225000000
H	-5.088170000000	2.048056000000	0.843469000000
H	-3.198374000000	2.435960000000	2.394862000000
H	-1.021232000000	1.300884000000	1.997506000000
H	5.887808000000	-0.107177000000	0.619873000000

Z''

C	3.826187000000	0.507370000000	1.407660000000
C	4.321513000000	-0.731803000000	1.016386000000
C	3.446662000000	-1.716699000000	0.569854000000
C	2.083036000000	-1.462794000000	0.509762000000
C	1.586626000000	-0.215763000000	0.884486000000
C	2.462861000000	0.764212000000	1.346266000000
C	0.131956000000	0.052145000000	0.837027000000
C	-0.538478000000	0.616378000000	-0.211856000000
S	-0.789820000000	-0.393704000000	2.261970000000
C	0.435323000000	-1.054237000000	3.423990000000
C	-2.014426000000	0.821227000000	-0.056953000000
B	0.088240000000	1.053623000000	-1.531544000000
Cl	-0.942706000000	1.828683000000	-2.759594000000
Cl	1.779999000000	0.867460000000	-2.014168000000
C	-2.913013000000	-0.166559000000	-0.459698000000
C	-4.282497000000	0.022375000000	-0.318079000000
C	-4.770595000000	1.204006000000	0.229506000000
C	-3.881662000000	2.194409000000	0.634197000000
C	-2.512889000000	2.002796000000	0.491915000000
H	4.501870000000	1.276798000000	1.757677000000
H	3.826429000000	-2.683179000000	0.265226000000
H	1.400201000000	-2.225057000000	0.157886000000
H	2.074432000000	1.729699000000	1.642947000000
H	0.911457000000	-1.949375000000	3.034061000000
H	1.183811000000	-0.307962000000	3.675400000000
H	-0.141405000000	-1.305709000000	4.312718000000
H	-2.532937000000	-1.086036000000	-0.886953000000

H	-4.968158000000	-0.752493000000	-0.636290000000
H	-5.837048000000	1.352967000000	0.338590000000
H	-4.254225000000	3.117275000000	1.060048000000
H	-1.820834000000	2.773878000000	0.806332000000
H	5.384323000000	-0.930085000000	1.061315000000

IN1C'

C	3.354846000000	-1.435158000000	-2.391170000000
C	4.269587000000	-0.388157000000	-2.327460000000
C	4.258001000000	0.471233000000	-1.232921000000
C	3.338930000000	0.292755000000	-0.210580000000
C	2.412649000000	-0.759045000000	-0.268979000000
C	2.433778000000	-1.624611000000	-1.372474000000
C	1.449052000000	-0.922109000000	0.758250000000
C	0.623477000000	-1.049132000000	1.636240000000
B	-0.598517000000	1.413492000000	-0.429142000000
S	-0.478153000000	-1.158365000000	2.889993000000
C	-1.622321000000	-2.423755000000	2.248154000000
Cl	0.083388000000	2.510365000000	0.776620000000
Cl	0.253309000000	1.308457000000	-1.970837000000
C	-1.909056000000	0.647459000000	-0.179861000000
C	-2.389066000000	-0.288890000000	-1.111302000000
C	-3.579290000000	-0.966715000000	-0.898040000000
C	-4.323446000000	-0.717528000000	0.252143000000
C	-3.867845000000	0.203779000000	1.189217000000
C	-2.671986000000	0.873567000000	0.977175000000
H	3.358052000000	-2.106895000000	-3.239781000000
H	4.985805000000	-0.242758000000	-3.125316000000
H	4.965257000000	1.288595000000	-1.177928000000
H	3.319335000000	0.968791000000	0.633069000000
H	1.718278000000	-2.433959000000	-1.424674000000
H	-2.409291000000	-2.517090000000	2.994313000000
H	-2.046942000000	-2.092431000000	1.305710000000
H	-1.104026000000	-3.372173000000	2.132955000000
H	-1.818791000000	-0.485092000000	-2.009028000000
H	-3.929656000000	-1.687542000000	-1.625047000000
H	-5.254306000000	-1.244189000000	0.418851000000
H	-4.441990000000	0.393966000000	2.086438000000
H	-2.321963000000	1.582670000000	1.713875000000

TS1C'

C	3.182704000000	-2.664089000000	-2.787146000000
C	4.062258000000	-1.587393000000	-2.825863000000
C	3.779198000000	-0.437734000000	-2.095362000000

C	2.620488000000	-0.355922000000	-1.336943000000
C	1.742294000000	-1.441194000000	-1.286409000000
C	2.029103000000	-2.597571000000	-2.018251000000
C	0.533931000000	-1.380617000000	-0.493403000000
C	-0.079764000000	-1.903085000000	0.449768000000
B	-0.855941000000	0.117045000000	-1.090517000000
S	-0.941687000000	-2.333072000000	1.764182000000
C	-2.072759000000	-3.619536000000	1.133065000000
Cl	-0.269776000000	1.491032000000	-0.043189000000
Cl	-0.375754000000	0.289350000000	-2.838633000000
C	-2.300254000000	-0.443620000000	-0.834305000000
C	-2.895844000000	-1.331458000000	-1.742610000000
C	-4.163814000000	-1.850873000000	-1.523307000000
C	-4.875585000000	-1.494217000000	-0.380759000000
C	-4.306758000000	-0.615891000000	0.533186000000
C	-3.035061000000	-0.099745000000	0.306675000000
H	3.395791000000	-3.561110000000	-3.353738000000
H	4.962671000000	-1.643248000000	-3.423262000000
H	4.459380000000	0.403440000000	-2.122132000000
H	2.389373000000	0.542942000000	-0.783864000000
H	1.342985000000	-3.432792000000	-1.985136000000
H	-2.632049000000	-3.960054000000	2.002031000000
H	-2.742168000000	-3.179303000000	0.401407000000
H	-1.497541000000	-4.437924000000	0.710758000000
H	-2.356112000000	-1.616658000000	-2.635882000000
H	-4.598809000000	-2.534041000000	-2.241732000000
H	-5.865128000000	-1.897223000000	-0.207208000000
H	-4.852206000000	-0.331242000000	1.423954000000
H	-2.606553000000	0.584279000000	1.026408000000

IN2C'

C	4.124101000000	-1.698175000000	-0.654537000000
C	4.769064000000	-0.643162000000	-1.294218000000
C	4.086703000000	0.546022000000	-1.521758000000
C	2.765982000000	0.688475000000	-1.118376000000
C	2.112078000000	-0.362291000000	-0.470141000000
C	2.808829000000	-1.558382000000	-0.245164000000
C	0.713894000000	-0.243318000000	-0.022298000000
C	0.137944000000	-1.057389000000	0.789103000000
B	-0.399010000000	0.943490000000	-0.513181000000
S	-0.603658000000	-1.941924000000	1.886189000000
C	-1.380820000000	-3.302434000000	0.942162000000
Cl	0.263888000000	2.567642000000	0.184254000000
Cl	-0.314510000000	0.963029000000	-2.397714000000

C	-1.881972000000	0.625986000000	0.002600000000
C	-2.696096000000	-0.286642000000	-0.682321000000
C	-3.938683000000	-0.669546000000	-0.191053000000
C	-4.405063000000	-0.145721000000	1.009961000000
C	-3.620913000000	0.770100000000	1.702097000000
C	-2.380120000000	1.148608000000	1.200683000000
H	4.645755000000	-2.629544000000	-0.478266000000
H	5.796760000000	-0.750375000000	-1.615623000000
H	4.582422000000	1.369113000000	-2.019192000000
H	2.246762000000	1.615412000000	-1.296702000000
H	2.308793000000	-2.385851000000	0.241656000000
H	-1.834876000000	-3.951268000000	1.688052000000
H	-2.136361000000	-2.872706000000	0.291187000000
H	-0.615943000000	-3.831925000000	0.382665000000
H	-2.348701000000	-0.705974000000	-1.617874000000
H	-4.543227000000	-1.376669000000	-0.745721000000
H	-5.371684000000	-0.441808000000	1.397307000000
H	-3.975387000000	1.190810000000	2.634975000000
H	-1.781886000000	1.857441000000	1.757355000000

TS2C'

C	4.170699000000	0.127739000000	-1.192365000000
C	4.612901000000	-1.145353000000	-0.849997000000
C	3.722644000000	-2.063443000000	-0.297698000000
C	2.398764000000	-1.711500000000	-0.087862000000
C	1.940439000000	-0.431187000000	-0.429405000000
C	2.845313000000	0.482789000000	-0.984366000000
C	0.563001000000	-0.006768000000	-0.228464000000
C	-0.486907000000	-0.642561000000	0.273344000000
B	-0.229168000000	1.347558000000	-0.482275000000
S	-1.020447000000	-2.119294000000	0.916594000000
C	-0.733411000000	-1.878641000000	2.698698000000
Cl	0.400611000000	2.776363000000	0.561445000000
Cl	-0.311796000000	1.852032000000	-2.288849000000
C	-1.689803000000	0.747937000000	0.077244000000
C	-2.650657000000	0.227463000000	-0.837560000000
C	-3.919680000000	-0.109230000000	-0.428234000000
C	-4.289380000000	0.097629000000	0.903146000000
C	-3.407694000000	0.685016000000	1.811971000000
C	-2.136835000000	1.022788000000	1.401788000000
H	4.858530000000	0.844315000000	-1.621841000000
H	5.646456000000	-1.422833000000	-1.011611000000
H	4.064263000000	-3.054950000000	-0.030271000000
H	1.712404000000	-2.429098000000	0.339359000000

H	2.499066000000	1.471205000000	-1.250427000000
H	-1.039834000000	-2.805519000000	3.180817000000
H	0.319053000000	-1.685162000000	2.887298000000
H	-1.348506000000	-1.057163000000	3.058649000000
H	-2.343246000000	0.051610000000	-1.858974000000
H	-4.626473000000	-0.539976000000	-1.124174000000
H	-5.281161000000	-0.186498000000	1.231185000000
H	-3.722656000000	0.864940000000	2.830847000000
H	-1.439690000000	1.468932000000	2.097404000000

INT1A''

C	0.908383000000	-3.093232000000	-0.285895000000
C	1.830728000000	-2.793987000000	-1.293515000000
C	1.592982000000	-1.721488000000	-2.153410000000
C	0.453932000000	-0.950002000000	-1.988612000000
C	-0.469233000000	-1.224500000000	-0.974502000000
C	-0.221131000000	-2.318397000000	-0.127414000000
C	-1.607330000000	-0.399510000000	-0.782762000000
C	-2.575893000000	0.306713000000	-0.600544000000
B	-0.287948000000	1.463808000000	1.670871000000
S	-3.872556000000	1.327368000000	-0.299470000000
C	-5.228856000000	0.461802000000	-1.164247000000
Cl	-1.235560000000	2.950220000000	1.537844000000
Cl	-0.888220000000	0.245113000000	2.799754000000
C	1.019105000000	1.266709000000	0.888460000000
C	1.851051000000	0.162850000000	1.137506000000
C	3.031271000000	-0.017355000000	0.434916000000
C	3.402937000000	0.898837000000	-0.543028000000
C	2.591442000000	1.995817000000	-0.814428000000
C	1.415663000000	2.178853000000	-0.102991000000
H	1.107901000000	-3.927841000000	0.372726000000
H	2.294080000000	-1.467759000000	-2.933287000000
H	0.283373000000	-0.106115000000	-2.642618000000
H	-0.920610000000	-2.539560000000	0.666892000000
H	-5.012422000000	0.390336000000	-2.227171000000
H	-5.377987000000	-0.525490000000	-0.734393000000
H	-6.117092000000	1.072002000000	-1.007191000000
H	1.561829000000	-0.562479000000	1.884557000000
H	3.653704000000	-0.879019000000	0.635892000000
H	4.319594000000	0.753010000000	-1.100136000000
H	2.876554000000	2.705398000000	-1.580148000000
H	0.789867000000	3.033850000000	-0.320011000000
O	2.929584000000	-3.586235000000	-1.348793000000
C	3.924464000000	-3.296115000000	-2.326817000000

H	3.522518000000	-3.390524000000	-3.338009000000
H	4.330111000000	-2.291659000000	-2.186675000000
H	4.711110000000	-4.030629000000	-2.178522000000

TS1A''

C	1.050026000000	-3.318825000000	-0.160752000000
C	1.914406000000	-2.983732000000	-1.216118000000
C	1.709438000000	-1.807341000000	-1.948385000000
C	0.660943000000	-0.975429000000	-1.616794000000
C	-0.199353000000	-1.284344000000	-0.548689000000
C	0.015201000000	-2.481543000000	0.170902000000
C	-1.199255000000	-0.394382000000	-0.162281000000
C	-1.973512000000	0.445039000000	0.311624000000
B	-0.646039000000	1.397129000000	1.720398000000
S	-3.552146000000	1.073768000000	0.402588000000
C	-4.468935000000	-0.190323000000	-0.533294000000
Cl	-1.312432000000	3.089538000000	1.603159000000
Cl	-1.176554000000	0.513425000000	3.217603000000
C	0.818514000000	1.180303000000	1.209152000000
C	1.617371000000	0.138874000000	1.696336000000
C	2.893615000000	-0.089868000000	1.197376000000
C	3.404995000000	0.723721000000	0.193995000000
C	2.631156000000	1.766849000000	-0.304016000000
C	1.356085000000	1.988483000000	0.198454000000
H	1.230446000000	-4.233472000000	0.386832000000
H	2.367758000000	-1.536646000000	-2.758804000000
H	0.508472000000	-0.056369000000	-2.163821000000
H	-0.637924000000	-2.721076000000	0.998299000000
H	-4.101555000000	-0.242065000000	-1.555700000000
H	-4.380322000000	-1.158204000000	-0.045393000000
H	-5.509629000000	0.131105000000	-0.530313000000
H	1.229558000000	-0.508024000000	2.471354000000
H	3.485222000000	-0.908443000000	1.587122000000
H	4.397375000000	0.545056000000	-0.200036000000
H	3.020513000000	2.405071000000	-1.087240000000
H	0.763155000000	2.799358000000	-0.203777000000
O	2.913246000000	-3.849629000000	-1.448795000000
C	3.848044000000	-3.567793000000	-2.493903000000
H	3.347207000000	-3.527329000000	-3.462475000000
H	4.368692000000	-2.627736000000	-2.304617000000
H	4.557615000000	-4.389446000000	-2.481967000000

INT2A''

C	1.229178000000	-3.164807000000	-0.758643000000

C	1.780294000000	-2.859909000000	-2.023346000000
C	1.387078000000	-1.697713000000	-2.718101000000
C	0.464200000000	-0.859697000000	-2.151963000000
C	-0.103767000000	-1.147018000000	-0.881047000000
C	0.310339000000	-2.328288000000	-0.198005000000
C	-0.991945000000	-0.285344000000	-0.312218000000
C	-1.745584000000	0.571520000000	0.257703000000
B	-0.889913000000	1.704574000000	1.165994000000
S	-3.485724000000	0.662405000000	0.174666000000
C	-3.884376000000	-0.735995000000	-0.907831000000
Cl	-1.340467000000	3.389967000000	0.442770000000
Cl	-1.575432000000	1.584417000000	2.921404000000
C	0.677355000000	1.396150000000	1.090404000000
C	1.276963000000	0.478459000000	1.960935000000
C	2.595005000000	0.069271000000	1.794685000000
C	3.355336000000	0.574011000000	0.745467000000
C	2.786157000000	1.497617000000	-0.124410000000
C	1.467188000000	1.900444000000	0.050807000000
H	1.561224000000	-4.062152000000	-0.256202000000
H	1.810783000000	-1.461578000000	-3.681183000000
H	0.159697000000	0.042556000000	-2.662791000000
H	-0.110631000000	-2.540807000000	0.774121000000
H	-3.426110000000	-0.606952000000	-1.886910000000
H	-3.561684000000	-1.674060000000	-0.459399000000
H	-4.967957000000	-0.737331000000	-1.011617000000
H	0.695985000000	0.067186000000	2.776250000000
H	3.027898000000	-0.647350000000	2.481854000000
H	4.381060000000	0.254757000000	0.610163000000
H	3.369277000000	1.902112000000	-0.942695000000
H	1.036244000000	2.611412000000	-0.642077000000
O	2.670405000000	-3.727209000000	-2.481631000000
C	3.297577000000	-3.509143000000	-3.757152000000
H	2.551452000000	-3.500569000000	-4.551309000000
H	3.857684000000	-2.574468000000	-3.750652000000
H	3.973562000000	-4.346900000000	-3.891085000000

TS2A''

C	1.147192000000	-4.165011000000	-1.228522000000
C	1.799949000000	-3.963106000000	-2.455568000000
C	1.680253000000	-2.732573000000	-3.130329000000
C	0.924318000000	-1.725629000000	-2.594318000000
C	0.261497000000	-1.914511000000	-1.357292000000
C	0.387327000000	-3.151143000000	-0.690843000000
C	-0.482987000000	-0.891875000000	-0.794385000000
C	-1.066462000000	0.075159000000	-0.205402000000

B	-0.722813000000	1.268897000000	0.813819000000
S	-2.609826000000	-0.301619000000	-1.100654000000
C	-3.505935000000	-1.344904000000	0.080324000000
Cl	-1.034554000000	2.879202000000	-0.160396000000
Cl	-1.973913000000	1.177992000000	2.231461000000
C	0.800490000000	1.159033000000	1.285829000000
C	1.188457000000	0.987950000000	2.616839000000
C	2.528157000000	0.854846000000	2.973800000000
C	3.517886000000	0.882877000000	1.999288000000
C	3.156211000000	1.049788000000	0.665321000000
C	1.818058000000	1.186905000000	0.320719000000
H	1.237109000000	-5.101019000000	-0.700161000000
H	2.197619000000	-2.606820000000	-4.070988000000
H	0.828370000000	-0.778527000000	-3.106034000000
H	-0.112739000000	-3.293189000000	0.256823000000
H	-3.015864000000	-2.311609000000	0.174792000000
H	-3.569837000000	-0.842572000000	1.040595000000
H	-4.500278000000	-1.481248000000	-0.344271000000
H	0.430512000000	0.958412000000	3.388349000000
H	2.797133000000	0.727671000000	4.015365000000
H	4.559950000000	0.777882000000	2.273913000000
H	3.917901000000	1.075726000000	-0.104330000000
H	1.557807000000	1.328554000000	-0.721671000000
O	2.558724000000	-4.879537000000	-3.058618000000
C	2.739620000000	-6.162744000000	-2.445171000000
H	3.222588000000	-6.059503000000	-1.472988000000
H	1.783525000000	-6.676032000000	-2.338253000000
H	3.383276000000	-6.718241000000	-3.119595000000

INT3A''

C	1.812097000000	-2.573503000000	-0.956050000000
C	1.343630000000	-3.485680000000	-1.908494000000
C	0.105100000000	-3.273323000000	-2.527307000000
C	-0.651932000000	-2.169071000000	-2.201120000000
C	-0.189803000000	-1.251407000000	-1.249029000000
C	1.048319000000	-1.466835000000	-0.630575000000
C	-0.948195000000	-0.094444000000	-0.888057000000
C	-1.130696000000	0.990393000000	-0.210548000000
B	-0.763543000000	2.074023000000	0.886567000000
S	-2.531057000000	0.625034000000	-1.360678000000
C	-3.783244000000	-0.247681000000	-0.372426000000
Cl	-0.781807000000	3.776750000000	0.064294000000
Cl	-2.137090000000	2.012759000000	2.196152000000
C	0.675537000000	1.642940000000	1.443998000000
C	0.805184000000	0.733727000000	2.500235000000

C	2.046319000000	0.236245000000	2.883013000000
C	3.196031000000	0.634321000000	2.209457000000
C	3.091466000000	1.537891000000	1.157099000000
C	1.846710000000	2.030975000000	0.781889000000
H	2.763379000000	-2.718045000000	-0.467983000000
H	-0.240525000000	-3.988939000000	-3.260417000000
H	-1.606842000000	-2.008911000000	-2.683571000000
H	1.410997000000	-0.763001000000	0.105725000000
H	-4.072615000000	-1.151801000000	-0.903039000000
H	-3.364184000000	-0.476477000000	0.603433000000
H	-4.634148000000	0.423151000000	-0.273458000000
H	-0.080916000000	0.406956000000	3.028569000000
H	2.115408000000	-0.465114000000	3.705352000000
H	4.163266000000	0.246115000000	2.502398000000
H	3.979907000000	1.856651000000	0.626134000000
H	1.781347000000	2.727720000000	-0.043802000000
O	2.012508000000	-4.592028000000	-2.291555000000
C	3.281045000000	-4.869140000000	-1.697070000000
H	3.993701000000	-4.069110000000	-1.906269000000
H	3.185243000000	-5.005665000000	-0.618165000000
H	3.624812000000	-5.792896000000	-2.153032000000

TS3A''

C	1.643414000000	-2.089168000000	-3.171993000000
C	2.449196000000	-2.802716000000	-2.283001000000
C	2.237010000000	-2.673527000000	-0.905640000000
C	1.250593000000	-1.837758000000	-0.428400000000
C	0.442016000000	-1.105272000000	-1.308133000000
C	0.647656000000	-1.258089000000	-2.679658000000
C	-0.593936000000	-0.197807000000	-0.816304000000
C	-0.748911000000	0.506729000000	0.274194000000
B	-0.848996000000	1.462100000000	1.411835000000
S	-2.050821000000	0.184989000000	-1.793800000000
C	-2.875724000000	-1.433002000000	-1.797223000000
Cl	-0.811737000000	3.269760000000	1.007108000000
Cl	-2.044697000000	1.070910000000	2.765530000000
C	0.681126000000	0.786706000000	1.617600000000
C	0.876835000000	-0.211805000000	2.585895000000
C	2.155979000000	-0.633433000000	2.908113000000
C	3.253737000000	-0.103734000000	2.233192000000
C	3.072065000000	0.856077000000	1.241962000000
C	1.794832000000	1.295382000000	0.933340000000
H	1.780158000000	-2.173319000000	-4.239099000000
H	2.857308000000	-3.241981000000	-0.225990000000
H	1.095452000000	-1.759862000000	0.635768000000

H	0.026564000000	-0.709263000000	-3.374058000000
H	-2.223699000000	-2.190842000000	-2.225641000000
H	-3.170710000000	-1.706481000000	-0.786739000000
H	-3.762346000000	-1.316832000000	-2.418747000000
H	0.022631000000	-0.633442000000	3.095976000000
H	2.300344000000	-1.383302000000	3.674486000000
H	4.251297000000	-0.446340000000	2.475279000000
H	3.925010000000	1.257405000000	0.711362000000
H	1.649318000000	2.040681000000	0.163778000000
O	3.443543000000	-3.639912000000	-2.655753000000
C	3.705551000000	-3.809840000000	-4.047439000000
H	2.836050000000	-4.223342000000	-4.562659000000
H	3.990888000000	-2.863062000000	-4.510792000000
H	4.533322000000	-4.510381000000	-4.110172000000

E''

C	1.235184000000	-1.770924000000	-3.387635000000
C	1.982199000000	-2.732411000000	-2.707137000000
C	1.919138000000	-2.792412000000	-1.309407000000
C	1.135354000000	-1.900723000000	-0.609281000000
C	0.390943000000	-0.914207000000	-1.276100000000
C	0.446433000000	-0.880837000000	-2.669866000000
C	-0.444485000000	0.014058000000	-0.523685000000
C	-0.371612000000	0.613152000000	0.687188000000
B	-1.793033000000	1.296601000000	0.830809000000
S	-2.000917000000	0.627293000000	-1.183034000000
C	-3.063753000000	-0.838264000000	-1.248526000000
Cl	-1.866509000000	3.121425000000	0.900520000000
Cl	-3.040783000000	0.482356000000	1.894365000000
C	0.754774000000	0.692248000000	1.616821000000
C	0.508304000000	0.840794000000	2.987737000000
C	1.557177000000	0.941705000000	3.892075000000
C	2.872316000000	0.917151000000	3.441984000000
C	3.131382000000	0.794956000000	2.078944000000
C	2.085598000000	0.682532000000	1.176344000000
H	1.257637000000	-1.706747000000	-4.464667000000
H	2.488611000000	-3.554553000000	-0.794589000000
H	1.089325000000	-1.964800000000	0.468720000000
H	-0.127830000000	-0.139188000000	-3.210215000000
H	-2.655973000000	-1.519413000000	-1.992146000000
H	-3.105164000000	-1.306266000000	-0.269057000000
H	-4.052263000000	-0.503962000000	-1.556311000000
H	-0.512928000000	0.866265000000	3.343370000000
H	1.346710000000	1.046109000000	4.948590000000
H	3.690368000000	1.001927000000	4.145396000000

H	4.152459000000	0.792426000000	1.719722000000
H	2.296370000000	0.599227000000	0.119389000000
O	2.780884000000	-3.647626000000	-3.305933000000
C	2.882833000000	-3.633744000000	-4.727613000000
H	1.911882000000	-3.817152000000	-5.192985000000
H	3.285666000000	-2.682778000000	-5.083190000000
H	3.566955000000	-4.437535000000	-4.984558000000

INT1B''

C	3.287542000000	1.402523000000	1.580790000000
C	4.283787000000	0.599496000000	1.014446000000
C	3.961580000000	-0.677622000000	0.550730000000
C	2.656182000000	-1.134828000000	0.652206000000
C	1.648677000000	-0.343471000000	1.214741000000
C	1.993119000000	0.937517000000	1.680048000000
C	0.309959000000	-0.808522000000	1.286881000000
C	-0.837989000000	-1.195921000000	1.338432000000
S	-2.438138000000	-1.699280000000	1.352193000000
C	-2.494616000000	-2.684156000000	2.889804000000
C	-2.129523000000	0.087655000000	-1.658945000000
B	-0.801198000000	0.826599000000	-1.425760000000
Cl	-0.717661000000	2.310017000000	-0.470295000000
Cl	0.709460000000	0.274449000000	-2.152364000000
C	-2.165675000000	-1.136592000000	-2.347101000000
C	-3.361088000000	-1.806010000000	-2.555142000000
C	-4.552688000000	-1.261403000000	-2.085636000000
C	-4.542592000000	-0.049072000000	-1.403784000000
C	-3.344059000000	0.614016000000	-1.189735000000
H	3.552870000000	2.389857000000	1.934249000000
H	4.712157000000	-1.314984000000	0.108989000000
H	2.407408000000	-2.120070000000	0.281954000000
H	1.226368000000	1.565663000000	2.111471000000
H	-1.807023000000	-3.523793000000	2.825132000000
H	-2.257744000000	-2.058626000000	3.746825000000
H	-3.517566000000	-3.049362000000	2.969803000000
H	-1.243851000000	-1.566866000000	-2.714020000000
H	-3.367294000000	-2.751471000000	-3.081487000000
H	-5.487194000000	-1.782798000000	-2.248908000000
H	-5.467922000000	0.373876000000	-1.035261000000
H	-3.343810000000	1.551089000000	-0.650062000000
O	5.523796000000	1.142031000000	0.958347000000
C	6.578096000000	0.371687000000	0.387167000000
H	6.741444000000	-0.549823000000	0.950372000000
H	6.367276000000	0.130130000000	-0.656940000000
H	7.466163000000	0.994868000000	0.444076000000

TS1B''

C	2.805427000000	1.271271000000	2.033337000000
C	3.713017000000	0.442704000000	1.353074000000
C	3.249541000000	-0.668769000000	0.636086000000
C	1.896883000000	-0.941136000000	0.605353000000
C	0.975475000000	-0.121944000000	1.281827000000
C	1.461192000000	0.994602000000	1.998335000000
C	-0.391974000000	-0.390582000000	1.230342000000
C	-1.614179000000	-0.506771000000	1.097299000000
S	-3.085619000000	-1.148047000000	1.661774000000
C	-2.544959000000	-1.875619000000	3.240422000000
C	-3.123215000000	0.064696000000	-1.191817000000
B	-1.951952000000	0.866710000000	-0.527113000000
Cl	-2.343081000000	2.352722000000	0.440804000000
Cl	-0.426056000000	1.024290000000	-1.500958000000
C	-2.855658000000	-1.030536000000	-2.026624000000
C	-3.881008000000	-1.764867000000	-2.602780000000
C	-5.207901000000	-1.417185000000	-2.361785000000
C	-5.496254000000	-0.334033000000	-1.540557000000
C	-4.464418000000	0.392801000000	-0.958299000000
H	3.188865000000	2.124428000000	2.575708000000
H	3.934717000000	-1.311534000000	0.105746000000
H	1.531385000000	-1.792548000000	0.048429000000
H	0.758310000000	1.634198000000	2.513505000000
H	-1.798210000000	-2.646535000000	3.064051000000
H	-2.149396000000	-1.103041000000	3.895670000000
H	-3.434582000000	-2.318349000000	3.686398000000
H	-1.828300000000	-1.309920000000	-2.220523000000
H	-3.649191000000	-2.607886000000	-3.241285000000
H	-6.009978000000	-1.987558000000	-2.812681000000
H	-6.525106000000	-0.055840000000	-1.350487000000
H	-4.701251000000	1.228757000000	-0.314416000000
O	5.005228000000	0.793541000000	1.445014000000
C	5.990710000000	-0.000182000000	0.778358000000
H	5.990333000000	-1.021636000000	1.162077000000
H	5.819367000000	-0.007202000000	-0.299157000000
H	6.943591000000	0.472871000000	0.995128000000

INT2B''

C	3.760376000000	0.999497000000	1.851159000000
C	4.525449000000	0.042322000000	1.147154000000
C	3.896003000000	-0.940400000000	0.354476000000
C	2.529604000000	-0.958268000000	0.270637000000
C	1.739883000000	-0.004229000000	0.969842000000
C	2.400007000000	0.979465000000	1.765000000000

C	0.382369000000	-0.024819000000	0.871449000000
C	-0.884487000000	-0.004746000000	0.737318000000
S	-2.033068000000	-0.867104000000	1.737472000000
C	-0.960586000000	-1.646656000000	2.972718000000
C	-2.472079000000	-0.058622000000	-1.389932000000
B	-1.534429000000	0.896951000000	-0.520334000000
Cl	-2.440399000000	2.330688000000	0.298916000000
Cl	-0.123259000000	1.613636000000	-1.571913000000
C	-1.894409000000	-1.077172000000	-2.160935000000
C	-2.670117000000	-1.963282000000	-2.895355000000
C	-4.058595000000	-1.855198000000	-2.876391000000
C	-4.653552000000	-0.856463000000	-2.116170000000
C	-3.866896000000	0.025608000000	-1.379859000000
H	4.281026000000	1.736851000000	2.445483000000
H	4.479110000000	-1.670238000000	-0.184448000000
H	2.029185000000	-1.699832000000	-0.335872000000
H	1.801327000000	1.708690000000	2.291848000000
H	-0.260627000000	-2.331951000000	2.497666000000
H	-0.423296000000	-0.893907000000	3.546941000000
H	-1.621413000000	-2.204321000000	3.633934000000
H	-0.815581000000	-1.172550000000	-2.188115000000
H	-2.195050000000	-2.738232000000	-3.484429000000
H	-4.667688000000	-2.543063000000	-3.449252000000
H	-5.732340000000	-0.761904000000	-2.094123000000
H	-4.345635000000	0.795985000000	-0.790370000000
O	5.837403000000	0.145548000000	1.290008000000
C	6.712068000000	-0.772134000000	0.610815000000
H	6.525257000000	-1.792462000000	0.944314000000
H	6.581484000000	-0.693380000000	-0.467979000000
H	7.716055000000	-0.468390000000	0.887229000000

TS2B''

C	4.762585000000	-1.118826000000	1.642461000000
C	5.115289000000	0.224446000000	1.872659000000
C	4.148275000000	1.237242000000	1.771737000000
C	2.852206000000	0.906417000000	1.444567000000
C	2.489023000000	-0.434918000000	1.208310000000
C	3.473118000000	-1.445764000000	1.317630000000
C	1.183041000000	-0.751142000000	0.860703000000
C	0.012403000000	-0.979570000000	0.416218000000
S	-0.520193000000	-1.343812000000	2.125142000000
C	-1.211906000000	0.240951000000	2.669284000000
C	-2.164065000000	-0.045781000000	-0.687795000000
B	-0.899235000000	-1.000176000000	-0.905698000000
Cl	0.216013000000	-0.431969000000	-2.332364000000

Cl	-1.376972000000	-2.815020000000	-1.180433000000
C	-3.379797000000	-0.531258000000	-0.193745000000
C	-4.426820000000	0.325193000000	0.132981000000
C	-4.281773000000	1.698365000000	-0.027006000000
C	-3.083932000000	2.204108000000	-0.522873000000
C	-2.043640000000	1.341045000000	-0.845166000000
H	5.529534000000	-1.875990000000	1.725288000000
H	4.407263000000	2.270484000000	1.941197000000
H	2.102840000000	1.680332000000	1.356337000000
H	3.196169000000	-2.475209000000	1.139682000000
H	-0.422508000000	0.986059000000	2.741646000000
H	-1.992300000000	0.563229000000	1.986463000000
H	-1.628437000000	0.054544000000	3.658799000000
H	-3.507252000000	-1.597010000000	-0.058881000000
H	-5.355986000000	-0.080833000000	0.513626000000
H	-5.093868000000	2.367568000000	0.227668000000
H	-2.960770000000	3.271870000000	-0.656544000000
H	-1.116122000000	1.750951000000	-1.224363000000
O	6.396562000000	0.440747000000	2.179346000000
C	6.848140000000	1.779507000000	2.420245000000
H	6.702586000000	2.399583000000	1.535042000000
H	6.327300000000	2.214287000000	3.274101000000
H	7.907499000000	1.693406000000	2.639804000000

INT3B''

C	3.603916000000	-0.022605000000	2.545781000000
C	4.402263000000	0.708989000000	1.657382000000
C	3.922815000000	1.012755000000	0.378085000000
C	2.660895000000	0.589750000000	-0.000390000000
C	1.860847000000	-0.140584000000	0.885609000000
C	2.348268000000	-0.442252000000	2.163528000000
C	0.560061000000	-0.558725000000	0.464285000000
C	-0.406842000000	-0.641161000000	-0.387688000000
S	-0.817220000000	-1.422475000000	1.240784000000
C	-1.708066000000	-0.156337000000	2.195993000000
C	-2.598470000000	0.481396000000	-1.154797000000
B	-1.309111000000	-0.327225000000	-1.655172000000
Cl	-0.185671000000	0.681233000000	-2.805701000000
Cl	-1.740075000000	-1.974655000000	-2.483030000000
C	-3.762977000000	-0.183877000000	-0.754595000000
C	-4.828898000000	0.498143000000	-0.177885000000
C	-4.754890000000	1.872947000000	0.016552000000
C	-3.608703000000	2.556145000000	-0.376131000000
C	-2.547061000000	1.865742000000	-0.949943000000
H	3.991485000000	-0.248429000000	3.529555000000

H	4.522842000000	1.573951000000	-0.321259000000
H	2.285780000000	0.823742000000	-0.987729000000
H	1.734922000000	-1.009027000000	2.851379000000
H	-1.690286000000	0.779379000000	1.643721000000
H	-2.731941000000	-0.505811000000	2.309338000000
H	-1.228955000000	-0.054624000000	3.166989000000
H	-3.835885000000	-1.254268000000	-0.896648000000
H	-5.717948000000	-0.044265000000	0.119541000000
H	-5.582691000000	2.407028000000	0.465563000000
H	-3.540798000000	3.627603000000	-0.233452000000
H	-1.659398000000	2.411206000000	-1.243528000000
O	5.614141000000	1.077850000000	2.119547000000
C	6.475225000000	1.831688000000	1.265275000000
H	6.721611000000	1.268327000000	0.363288000000
H	6.017503000000	2.784473000000	0.992259000000
H	7.378017000000	2.011882000000	1.841355000000

TS3B''

C	3.399627000000	0.944762000000	0.089496000000
C	4.157332000000	0.148108000000	0.944871000000
C	3.523155000000	-0.862630000000	1.671877000000
C	2.161720000000	-1.065670000000	1.551211000000
C	1.382004000000	-0.284398000000	0.681549000000
C	2.032075000000	0.723214000000	-0.041594000000
C	-0.043928000000	-0.474178000000	0.569719000000
C	-0.976600000000	-0.379915000000	-0.451704000000
S	-0.930568000000	-1.982284000000	0.402457000000
C	-2.136017000000	-2.055826000000	1.767045000000
C	-2.487342000000	0.558586000000	-0.194655000000
B	-1.428845000000	0.084105000000	-1.801645000000
Cl	-0.727956000000	1.574156000000	-2.531419000000
Cl	-2.353657000000	-0.984066000000	-2.910691000000
C	-3.737454000000	-0.050893000000	-0.133047000000
C	-4.783075000000	0.575049000000	0.536025000000
C	-4.581374000000	1.812729000000	1.134331000000
C	-3.329272000000	2.426402000000	1.069426000000
C	-2.284641000000	1.797233000000	0.415421000000
H	3.858255000000	1.733903000000	-0.487774000000
H	4.115851000000	-1.477650000000	2.336727000000
H	1.687777000000	-1.846165000000	2.133697000000
H	1.462219000000	1.345225000000	-0.718320000000
H	-1.641638000000	-2.611202000000	2.563321000000
H	-2.373369000000	-1.048195000000	2.091740000000
H	-3.022395000000	-2.589510000000	1.430135000000
H	-3.901297000000	-1.006949000000	-0.611113000000

H	-5.752281000000	0.096380000000	0.586806000000
H	-5.395324000000	2.301640000000	1.653324000000
H	-3.172846000000	3.390797000000	1.534730000000
H	-1.305193000000	2.256152000000	0.387542000000
O	5.503646000000	0.278466000000	1.139056000000
C	6.185848000000	1.291628000000	0.413407000000
H	6.102205000000	1.132540000000	-0.665032000000
H	5.802531000000	2.285001000000	0.661872000000
H	7.229749000000	1.223146000000	0.708752000000

INT4B''

C	3.482826000000	0.958697000000	0.291647000000
C	4.336327000000	-0.043405000000	0.744464000000
C	3.785808000000	-1.181903000000	1.336103000000
C	2.415724000000	-1.315105000000	1.468633000000
C	1.531889000000	-0.320826000000	1.016709000000
C	2.105012000000	0.815566000000	0.430870000000
C	0.088359000000	-0.383916000000	1.230342000000
C	-0.930439000000	-0.265215000000	0.143433000000
S	-0.749847000000	-1.941656000000	0.969460000000
C	-2.004611000000	-2.019791000000	2.270436000000
C	-2.215106000000	0.454246000000	0.403663000000
B	-0.381130000000	-0.275497000000	-1.320094000000
Cl	-0.510099000000	1.194627000000	-2.275814000000
Cl	0.513618000000	-1.621685000000	-2.000892000000
C	-3.354544000000	0.150944000000	-0.342506000000
C	-4.541803000000	0.833467000000	-0.121088000000
C	-4.597373000000	1.838215000000	0.841877000000
C	-3.463192000000	2.149732000000	1.582892000000
C	-2.277469000000	1.455645000000	1.368322000000
H	3.870721000000	1.854622000000	-0.170347000000
H	4.450406000000	-1.962873000000	1.683257000000
H	2.016636000000	-2.212859000000	1.924700000000
H	1.464486000000	1.617481000000	0.085666000000
H	-1.534687000000	-2.506179000000	3.123657000000
H	-2.318589000000	-1.015002000000	2.533427000000
H	-2.840492000000	-2.613409000000	1.905007000000
H	-3.310690000000	-0.630975000000	-1.091539000000
H	-5.423171000000	0.584806000000	-0.697696000000
H	-5.521772000000	2.374768000000	1.011998000000
H	-3.501298000000	2.932282000000	2.329664000000
H	-1.389231000000	1.674459000000	1.945153000000
O	5.701786000000	-0.004409000000	0.654786000000
C	6.293020000000	1.134613000000	0.047729000000
H	5.966680000000	1.246949000000	-0.989978000000

H	6.055719000000	2.048388000000	0.599725000000
H	7.366981000000	0.967342000000	0.072089000000

TS4B''

C	3.973520000000	0.754562000000	0.174480000000
C	4.819768000000	-0.229108000000	0.676131000000
C	4.261721000000	-1.337994000000	1.319123000000
C	2.892246000000	-1.462747000000	1.449549000000
C	2.013258000000	-0.485761000000	0.947516000000
C	2.594176000000	0.623428000000	0.316711000000
C	0.571017000000	-0.542122000000	1.117779000000
C	-0.375250000000	-0.356524000000	0.028317000000
S	-0.292467000000	-2.092712000000	1.079851000000
C	-1.400077000000	-2.059483000000	2.511379000000
C	-1.689274000000	0.292918000000	0.255507000000
B	0.175001000000	-0.458045000000	-1.440522000000
Cl	0.125884000000	0.954317000000	-2.483756000000
Cl	0.925357000000	-1.916511000000	-2.049406000000
C	-2.755600000000	0.067758000000	-0.621221000000
C	-3.977824000000	0.687866000000	-0.417538000000
C	-4.149595000000	1.551897000000	0.662475000000
C	-3.094404000000	1.786135000000	1.535883000000
C	-1.870279000000	1.159033000000	1.334386000000
H	4.367383000000	1.629456000000	-0.321427000000
H	4.923191000000	-2.104726000000	1.702205000000
H	2.486364000000	-2.342429000000	1.933274000000
H	1.958702000000	1.419537000000	-0.050259000000
H	-0.810404000000	-1.836514000000	3.397792000000
H	-2.155657000000	-1.289384000000	2.362128000000
H	-1.873881000000	-3.038724000000	2.578659000000
H	-2.628887000000	-0.608029000000	-1.458564000000
H	-4.797893000000	0.500930000000	-1.098321000000
H	-5.102341000000	2.041185000000	0.817677000000
H	-3.222359000000	2.459773000000	2.373227000000
H	-1.036929000000	1.330289000000	2.001968000000
O	6.185084000000	-0.201182000000	0.592006000000
C	6.785302000000	0.901422000000	-0.071425000000
H	6.460571000000	0.962841000000	-1.113823000000
H	6.553710000000	1.843547000000	0.433206000000
H	7.857949000000	0.727950000000	-0.036841000000

Z''

C	3.232536000000	0.846556000000	0.663717000000
C	3.849467000000	-0.401008000000	0.582291000000
C	3.065530000000	-1.560895000000	0.565802000000
C	1.690733000000	-1.470365000000	0.630098000000

C	1.057620000000	-0.223728000000	0.684072000000
C	1.845506000000	0.922533000000	0.709791000000
C	-0.410221000000	-0.111129000000	0.712059000000
C	-1.149239000000	0.489963000000	-0.267821000000
S	-1.253563000000	-0.755013000000	2.113074000000
C	0.045143000000	-1.036206000000	3.348400000000
C	-2.614467000000	0.692911000000	-0.039538000000
B	-0.599134000000	0.933989000000	-1.620136000000
Cl	-1.494083000000	2.143827000000	-2.570184000000
Cl	0.835814000000	0.275317000000	-2.423481000000
C	-3.540708000000	-0.223739000000	-0.536188000000
C	-4.903243000000	-0.037063000000	-0.329505000000
C	-5.355590000000	1.073480000000	0.373455000000
C	-4.438417000000	1.995424000000	0.869530000000
C	-3.078367000000	1.804876000000	0.665033000000
H	3.813008000000	1.756036000000	0.687395000000
H	3.557884000000	-2.521721000000	0.499096000000
H	1.093039000000	-2.372261000000	0.608912000000
H	1.368486000000	1.892194000000	0.762591000000
H	0.664465000000	-1.893342000000	3.101955000000
H	0.661217000000	-0.148358000000	3.465701000000
H	-0.492350000000	-1.230576000000	4.275227000000
H	-3.189051000000	-1.088283000000	-1.085313000000
H	-5.610083000000	-0.758473000000	-0.719073000000
H	-6.415796000000	1.221752000000	0.532834000000
H	-4.783754000000	2.863824000000	1.415957000000
H	-2.364651000000	2.520837000000	1.052344000000
O	5.190383000000	-0.590463000000	0.520907000000
C	6.037162000000	0.555522000000	0.519586000000
H	5.831709000000	1.194758000000	-0.341975000000
H	5.919590000000	1.132787000000	1.439488000000
H	7.052886000000	0.175203000000	0.456596000000

INT1C''

C	3.795925000000	-0.183639000000	1.457449000000
C	4.442925000000	-0.069692000000	0.226718000000
C	3.789873000000	-0.490200000000	-0.935946000000
C	2.513833000000	-1.011132000000	-0.871107000000
C	1.846589000000	-1.128201000000	0.359985000000
C	2.511754000000	-0.707300000000	1.515988000000
C	0.521746000000	-1.629682000000	0.422416000000
C	-0.613460000000	-2.051854000000	0.468019000000
B	-0.890783000000	1.196561000000	-0.616179000000
S	-2.182448000000	-2.625190000000	0.598906000000
C	-2.578902000000	-2.963702000000	-1.147406000000

Cl	0.370009000000	2.014708000000	0.307222000000
Cl	-0.488022000000	0.750673000000	-2.279476000000
C	-2.287818000000	0.938843000000	-0.029543000000
C	-3.351504000000	0.522570000000	-0.845044000000
C	-4.609201000000	0.278684000000	-0.314870000000
C	-4.829086000000	0.437270000000	1.049197000000
C	-3.788833000000	0.845955000000	1.878474000000
C	-2.535979000000	1.099576000000	1.343113000000
H	4.276437000000	0.133256000000	2.370499000000
H	4.301596000000	-0.396306000000	-1.884476000000
H	2.014095000000	-1.323894000000	-1.777183000000
H	2.012362000000	-0.782694000000	2.472354000000
H	-3.616990000000	-3.291476000000	-1.157663000000
H	-2.474079000000	-2.054037000000	-1.731646000000
H	-1.936165000000	-3.749879000000	-1.535353000000
H	-3.188438000000	0.393244000000	-1.906483000000
H	-5.416747000000	-0.038541000000	-0.961658000000
H	-5.808519000000	0.240990000000	1.466282000000
H	-3.957654000000	0.965748000000	2.940655000000
H	-1.732490000000	1.415591000000	1.994496000000
O	5.694217000000	0.431726000000	0.061005000000
C	6.401438000000	0.877549000000	1.213564000000
H	6.559095000000	0.059490000000	1.920470000000
H	7.361914000000	1.236638000000	0.854135000000
H	5.870390000000	1.692268000000	1.711524000000

TS1C''

C	4.315315000000	-1.017723000000	0.710730000000
C	5.027462000000	-0.686366000000	-0.442450000000
C	4.357914000000	-0.613137000000	-1.667701000000
C	3.001179000000	-0.855649000000	-1.739799000000
C	2.276337000000	-1.200127000000	-0.591786000000
C	2.953926000000	-1.276703000000	0.627434000000
C	0.862274000000	-1.466416000000	-0.621146000000
C	-0.079542000000	-2.212366000000	-0.339437000000
B	-0.352895000000	0.232460000000	-1.419522000000
S	-1.426491000000	-3.065411000000	0.046836000000
C	-1.781201000000	-3.963093000000	-1.503381000000
Cl	0.675296000000	1.560615000000	-0.746077000000
Cl	-0.094832000000	-0.091936000000	-3.186752000000
C	-1.800259000000	0.093138000000	-0.847102000000
C	-2.854380000000	-0.402021000000	-1.622772000000
C	-4.133317000000	-0.550144000000	-1.097623000000
C	-4.385360000000	-0.211993000000	0.225683000000
C	-3.351065000000	0.279492000000	1.017516000000

C	-2.079775000000	0.432973000000	0.484254000000
H	4.804553000000	-1.080328000000	1.670709000000
H	4.920891000000	-0.356537000000	-2.555021000000
H	2.493125000000	-0.781002000000	-2.689097000000
H	2.406282000000	-1.533710000000	1.523905000000
H	-2.663396000000	-4.565678000000	-1.296158000000
H	-1.993854000000	-3.247297000000	-2.291876000000
H	-0.941713000000	-4.600960000000	-1.763784000000
H	-2.672909000000	-0.672397000000	-2.654373000000
H	-4.931103000000	-0.931751000000	-1.722180000000
H	-5.379022000000	-0.329004000000	0.639032000000
H	-3.538911000000	0.545519000000	2.050046000000
H	-1.285578000000	0.817374000000	1.110846000000
O	6.357178000000	-0.421862000000	-0.472028000000
C	7.087079000000	-0.478370000000	0.750088000000
H	7.046321000000	-1.478715000000	1.187134000000
H	8.114742000000	-0.235138000000	0.494853000000
H	6.707164000000	0.250664000000	1.469455000000

INT2C''

C	3.598396000000	-1.692214000000	-0.114941000000
C	4.257429000000	-0.732749000000	-0.895502000000
C	3.564802000000	0.402388000000	-1.318397000000
C	2.234308000000	0.572826000000	-0.964663000000
C	1.564713000000	-0.371286000000	-0.184613000000
C	2.279394000000	-1.509949000000	0.231025000000
C	0.163915000000	-0.205321000000	0.207496000000
C	-0.491230000000	-1.007976000000	0.974448000000
B	-0.872640000000	1.049642000000	-0.279459000000
S	-1.316775000000	-1.875296000000	2.022648000000
C	-2.171931000000	-3.147654000000	1.023814000000
Cl	-0.072130000000	2.640495000000	0.348955000000
Cl	-0.853808000000	1.016688000000	-2.169021000000
C	-2.358295000000	0.865375000000	0.291529000000
C	-3.292269000000	0.058951000000	-0.372186000000
C	-4.541589000000	-0.213593000000	0.173677000000
C	-4.893620000000	0.318167000000	1.409264000000
C	-3.988648000000	1.132100000000	2.081813000000
C	-2.742825000000	1.400267000000	1.526167000000
H	4.143320000000	-2.569832000000	0.205357000000
H	4.047976000000	1.156268000000	-1.920603000000
H	1.717006000000	1.456273000000	-1.299209000000
H	1.785966000000	-2.262476000000	0.832859000000
H	-2.679794000000	-3.789400000000	1.740722000000
H	-2.888081000000	-2.645156000000	0.380385000000

H	-1.439324000000	-3.709326000000	0.452746000000
H	-3.034511000000	-0.365742000000	-1.333731000000
H	-5.240159000000	-0.841505000000	-0.365682000000
H	-5.864721000000	0.107579000000	1.839033000000
H	-4.253266000000	1.558622000000	3.041500000000
H	-2.049923000000	2.030375000000	2.067770000000
O	5.550398000000	-0.992070000000	-1.185912000000
C	6.275373000000	-0.049741000000	-1.975392000000
H	6.325826000000	0.920604000000	-1.477398000000
H	7.276066000000	-0.458882000000	-2.079961000000
H	5.821212000000	0.066074000000	-2.961435000000

TS2C''

C	4.013204000000	-1.838133000000	-0.147315000000
C	4.776519000000	-0.990093000000	-0.961032000000
C	4.187678000000	0.145742000000	-1.518556000000
C	2.852610000000	0.420361000000	-1.259121000000
C	2.076110000000	-0.414089000000	-0.449108000000
C	2.688428000000	-1.553718000000	0.101949000000
C	0.687422000000	-0.071744000000	-0.220062000000
C	-0.264062000000	-0.665357000000	0.491837000000
B	-0.266760000000	1.091603000000	-0.729353000000
S	-0.592722000000	-1.981269000000	1.510767000000
C	-1.149895000000	-3.248888000000	0.327631000000
Cl	0.267454000000	2.810945000000	-0.193068000000
Cl	-0.546249000000	1.053342000000	-2.587157000000
C	-1.604245000000	0.547305000000	0.123794000000
C	-2.623250000000	-0.214186000000	-0.516986000000
C	-3.808486000000	-0.504200000000	0.122311000000
C	-4.034174000000	-0.009900000000	1.408048000000
C	-3.097082000000	0.812538000000	2.038222000000
C	-1.913200000000	1.100640000000	1.399938000000
H	4.485131000000	-2.714765000000	0.275995000000
H	4.753937000000	0.814624000000	-2.148384000000
H	2.401040000000	1.301749000000	-1.691775000000
H	2.113956000000	-2.217150000000	0.733379000000
H	-1.363048000000	-4.139892000000	0.916096000000
H	-2.057308000000	-2.911142000000	-0.167991000000
H	-0.367749000000	-3.454551000000	-0.398190000000
H	-2.430819000000	-0.598065000000	-1.509192000000
H	-4.558756000000	-1.115216000000	-0.360854000000
H	-4.957150000000	-0.254493000000	1.918234000000
H	-3.301521000000	1.207797000000	3.023836000000
H	-1.168836000000	1.716341000000	1.885249000000
O	6.066555000000	-1.352687000000	-1.148230000000

C	6.893161000000	-0.529936000000	-1.968788000000
H	6.979680000000	0.476615000000	-1.554165000000
H	7.869853000000	-1.005414000000	-1.977706000000
H	6.503415000000	-0.474522000000	-2.987432000000

Z'''

C	2.914745000000	-1.594449000000	-1.841763000000
C	3.950738000000	-0.910665000000	-1.199447000000
C	3.650337000000	0.164085000000	-0.364891000000
C	2.322416000000	0.541707000000	-0.183639000000
C	1.282007000000	-0.130367000000	-0.814902000000
C	1.601925000000	-1.206239000000	-1.648130000000
C	-0.140772000000	0.289749000000	-0.612984000000
C	-0.851173000000	-0.332706000000	0.374467000000
B	-0.676363000000	1.391036000000	-1.522093000000
S	-0.021500000000	-1.526564000000	1.357994000000
C	-1.314173000000	-2.245870000000	2.407576000000
Cl	-2.183141000000	2.295672000000	-1.308747000000
Cl	0.290472000000	1.896372000000	-2.929605000000
C	-2.282155000000	-0.105165000000	0.671534000000
C	-3.259517000000	-0.712355000000	-0.114522000000
C	-4.605028000000	-0.511814000000	0.165915000000
C	-4.982417000000	0.294195000000	1.234035000000
C	-4.008334000000	0.891541000000	2.028130000000
C	-2.663444000000	0.685714000000	1.754488000000
H	3.160346000000	-2.425934000000	-2.489183000000
H	4.430155000000	0.711059000000	0.143035000000
H	2.097496000000	1.379301000000	0.464834000000
H	0.807157000000	-1.742877000000	-2.150656000000
H	-0.814654000000	-3.053588000000	2.940272000000
H	-1.700451000000	-1.524240000000	3.121502000000
H	-2.123895000000	-2.652997000000	1.807929000000
H	-2.961128000000	-1.330935000000	-0.950668000000
H	-5.358057000000	-0.981569000000	-0.453257000000
H	-6.030731000000	0.454855000000	1.448890000000
H	-4.296699000000	1.520200000000	2.860429000000
H	-1.903733000000	1.152204000000	2.368092000000
O	5.210045000000	-1.362175000000	-1.448718000000
C	6.299762000000	-0.697268000000	-0.820244000000
H	6.227051000000	-0.760977000000	0.268265000000
H	7.197080000000	-1.211453000000	-1.154079000000
H	6.348417000000	0.352410000000	-1.120084000000
