# Supplementary material for: Diversity-oriented synthesis of stereodefined tetrasubstituted alkenes via a modular alkyne gem-addition strategy
Source: Nat Commun. 2025 Jan 25;16:1025. doi: 10.1038/s41467-025-56184-3 (PMC11763084; doi:10.1038/s41467-025-56184-3)
Supplement: Supplementary file 6 — NCOMMS-24-50497-T-s06 [file 41467_2025_56184_MOESM6_ESM.pdf]

nature portfolio

Corresponding author(s): Jie Han, Jin Xie and Hongming Jin

Last updated by author(s): Dec 14, 2024

Reporting Summary

Nature Portfolio wishes to improve the reproducibility of the work that we publish. This form provides structure for consistency and transparency in reporting. For further information on Nature Portfolio policies, see our Editorial Policies and the Editorial Policy Checklist.

Statistics

For all statistical analyses, confirm that the following items are present in the figure legend, table legend, main text, or Methods section.

n/a	Confirmed	
☐	☒	The exact sample size (*n*) for each experimental group/condition, given as a discrete number and unit of measurement
☐	☒	A statement on whether measurements were taken from distinct samples or whether the same sample was measured repeatedly
☐	☒	The statistical test(s) used AND whether they are one- or two-sided<br>*Only common tests should be described solely by name; describe more complex techniques in the Methods section.*
☐	☒	A description of all covariates tested
☐	☒	A description of any assumptions or corrections, such as tests of normality and adjustment for multiple comparisons
☐	☒	A full description of the statistical parameters including central tendency (e.g. means) or other basic estimates (e.g. regression coefficient) AND variation (e.g. standard deviation) or associated estimates of uncertainty (e.g. confidence intervals)
☒	☐	For null hypothesis testing, the test statistic (e.g. *F*, *t*, *r*) with confidence intervals, effect sizes, degrees of freedom and *P* value noted<br>*Give P values as exact values whenever suitable.*
☒	☐	For Bayesian analysis, information on the choice of priors and Markov chain Monte Carlo settings
☒	☐	For hierarchical and complex designs, identification of the appropriate level for tests and full reporting of outcomes
☒	☐	Estimates of effect sizes (e.g. Cohen's *d*, Pearson's *r*), indicating how they were calculated

Our web collection on statistics for biologists contains articles on many of the points above.

Software and code

Policy information about availability of computer code

Data collection	N/A
Data analysis	All data were analyzed by GraphPad Prism version 7.0 (RRID: SCR_002798).

For manuscripts utilizing custom algorithms or software that are central to the research but not yet described in published literature, software must be made available to editors and reviewers. We strongly encourage code deposition in a community repository (e.g. GitHub). See the Nature Portfolio guidelines for submitting code & software for further information.

Data

Policy information about availability of data

All manuscripts must include a data availability statement. This statement should provide the following information, where applicable:

- Accession codes, unique identifiers, or web links for publicly available datasets
- A description of any restrictions on data availability
- For clinical datasets or third party data, please ensure that the statement adheres to our policy

CCDC 2370343 (17), 2369379 (104), 2369378 (105) contains the supplementary crystallographic data for this paper. The data can be obtained free of charge from the Cambridge Crystallographic Data Centre via www.ccdc.cam.ac.uk/data_request/cif. All other data that support the findings of this paper, including detailed experimental procedures, biological data, compound characterizations and DFT calculations, are available within the paper and its Supplementary Information.

Research involving human participants, their data, or biological material

Policy information about studies with human participants or human data. See also policy information about sex, gender (identity/presentation), and sexual orientation and race, ethnicity and racism.

Reporting on sex and gender	N/A
Reporting on race, ethnicity, or other socially relevant groupings	N/A
Population characteristics	N/A
Recruitment	N/A
Ethics oversight	N/A

Note that full information on the approval of the study protocol must also be provided in the manuscript.

Field-specific reporting

Please select the one below that is the best fit for your research. If you are not sure, read the appropriate sections before making your selection.

- [x] Life sciences
- [ ] Behavioural & social sciences
- [ ] Ecological, evolutionary & environmental sciences

For a reference copy of the document with all sections, see nature.com/documents/nr-reporting-summary-flat.pdf

Life sciences study design

All studies must disclose on these points even when the disclosure is negative.

Sample size	Each in vitro experiment were performed on cells containing over 6,000 cells per well and was repeated at least three times for statistical analysis. Seven animals were used in each group for in vivo experiments to exclude the influence of individual differences on the results.
Data exclusions	No data was excluded from analysis.
Replication	Each in vitro experiment was repeated at least three times for statistical analysis. Seven mice were used in each group for in vivo experiments to exclude the influence of individual differences on the results. In Figure S2, at least three different tissue sections were imaged, and each experiment was repeated at least three times.
Randomization	In vivo experiment, mice were randomly divided into five groups according to tumor volume to ensure that the average tumor volume of mice in each group was almost same, so as to abjectively evaluate the effecct of the compound on tumor growth.
Blinding	Investigators were blinded to group allocation during data collection and analysis.

Reporting for specific materials, systems and methods

We require information from authors about some types of materials, experimental systems and methods used in many studies. Here, indicate whether each material, system or method listed is relevant to your study. If you are not sure if a list item applies to your research, read the appropriate section before selecting a response.

Materials & experimental systems

n/a	Involved in the study	
x		Antibodies
	x	Eukaryotic cell lines
x		Palaeontology and archaeology
	x	Animals and other organisms
x		Clinical data
x		Dual use research of concern
x		Plants

Methods

n/a	Involved in the study	
x		ChIP-seq
x		Flow cytometry
x		MRI-based neuroimaging

Eukaryotic cell lines

Policy information about cell lines and Sex and Gender in Research

Cell line source(s)	Cells were obtained from ATCC.
Authentication	STR was used to identify the cell lines. The steps include:1.Extraction of DNA of cell line;2.DNA amplification;3.Genetic analyzer;4.Analyze and derive STR map;5. Data comparision.
Mycoplasma contamination	The cell lines were not tested for mycoplasma contamination.
Commonly misidentified lines (See ICLAC register)	*Name any commonly misidentified cell lines used in the study and provide a rationale for their use.*

Animals and other research organisms

Policy information about studies involving animals; ARRIVE guidelines recommended for reporting animal research, and Sex and Gender in Research

Laboratory animals	Female NU/NU nude mice (4 weeks old) were used in the study to evaluate the antitumor effect of compounds.
Wild animals	No wild animal was used in the study.
Reporting on sex	Tamoxifen is commonly used in the treatment of estrogen-dependent tumors, so the female mice were mainly used in the study to evaluate the anti-tumor effect of the compouds. In addition, estradiol was injected to improve the oncogenesis of MCF-7 transplanted tumors during the construction of xenograft model.
Field-collected samples	The study did not involve samples collected from the field.
Ethics oversight	All animal experiments were performed in accordance with the institutional ethical guidelines on animal care of Nanjing University of Chinese Medicine(Approval NO.202406A093).

Note that full information on the approval of the study protocol must also be provided in the manuscript.

Plants

Seed stocks	N/A
Novel plant genotypes	N/A
Authentication	N/A
