# Supplementary material for: Diversity-oriented synthesis of stereodefined tetrasubstituted alkenes via a modular alkyne gem-addition strategy
Source: Nat Commun. 2025 Jan 25;16:1025. doi: 10.1038/s41467-025-56184-3 (PMC11763084; doi:10.1038/s41467-025-56184-3)
Supplement: Supplementary file 7 — NCOMMS-24-50497-T-s07 [file 41467_2025_56184_MOESM7_ESM.pdf]

nature portfolio

Peer Review File

Diversity-Oriented Synthesis of Stereodefined Tetrasubstituted Alkenes via a Modular Alkyne gem-Addition Strategy

Corresponding Author: Professor Jin Xie

This file contains all reviewer reports in order by version, followed by all author rebuttals in order by version.

Version 0:

Reviewer comments:

Reviewer #1

(Remarks to the Author)
In this manuscript, the authors explored a type of meaningful alkynyl selenide substrates and demonstrated that they perform exclusive 1,1-addition selectivity when react with ArBCl2. The excellent stereoselectivity of this 1,1-carboboration is satisfying, enabling the stereocontrolled construction of tetrasubstituted alkenes. To elucidate the selenide and substituent influence on the regio- and stereoselectivity, a mass of computational studies was conducted, which further disclosed the specific impact of selenium atom on the reaction mechanism, especially compared with its sulfur analogue. A large scope of functional groups, including some Lewis-acid sensitive substituents, proved of the great compatibility of this methodology. A gram-scale experiment attested the practicability of this method. On basis of the gem-addition strategy, a modular access to all-carbon tetrasubstituted alkenes was described. The authors further developed Ni-catalyzed C-Se bond activation. The selenide serving as a removable directing group could convert to various carbon substituents. The broad applications in AIE materials and medicinal chemistry are much impressive. Up to six stereoisomers of alkene with four different aryl groups was successfully prepared by this protocol. A library of tetrasubstituted alkene-based antitumor lead compounds also proved the merit of diversity-oriented synthesis in drug discovery. Overall, this solid work presents a novel programmed synthetic approach to achieving the diversity-oriented synthesis of stereodefined all-carbon tetrasubstituted alkenes, which had been a challenging issue in organic chemistry. Thus, I would like to recommend its publication in Nature Communications after some revisions and answering following questions.

(1) In the introduction, the serial number of small letters in Fig. 1 brackets is inconsistent with the numbers in Fig. 1 graphic.
(2) In the final product of 1,1-caboboration, there might be a coordination of Se to boron. The authors should explain whether it is favorable to the E-selectivity in manuscript.
(3) In Table 1, the C-Se bond of compound 38 is too long. The authors need to redraw the structure.
(4) Except for forming aryl dichloroboranes from ArTMS, did authors try other approaches to access ArBCl2?
(5) In Table 2, 1,1-disubstituted 1,3-enyne is not included. How about this kind of substrate?
(6) In Ni-catalyzed C-Se bond transformations, is there any stereoisomer byproducts? Do authors try other transition metal catalysis?
(7) Some peaks in 13C-NMR spectra are too low, such as in page S125, S139, S172, S194, S210, S215, S221. These spectra should be re-edited.
(8) Some literatures about synthesis of stereodefined tetrasubstituted alkenes (such as Nat. Commun. 2021, 12, 7298, Angew. Chem. Int. Ed. 2024, 63, e202400805) should be involving.

Reviewer #2

(Remarks to the Author)
This manuscript describes a facile synthesis of stereodefined tetrasubstituted alkenes via a modular alkyne gem-addition strategy, in which alkynyl selenides, when treated with ArBCl2, are subject to an exclusive 1,1- carboboration, affording tetrasubstituted alkenes with excellent levels of E-selectivity. The DFT calculations show that alkynyl selenide tend to undergo 1,1-addition reaction to form E-alkenes. Then, a series of fluorescent molecules and selenium derivatives of tamoxifen were synthesized. Among them, derivative 109 showed slightly better anti-breast cancer activity than tamoxifen in vitro and in vivo. In general, the strategy used in this study to stereoselectively synthesize tetrasubstituted alkenes is

innovative, and the manuscript is well written and the result is robust. However, there are still some issues/problems in the manuscript that need to be addressed before ready for publication. Some of concerns are given below.
1. Based on experimental results and DFT calculations, the authors are suggested to propose a catalytic cycle with respect to the exclusive 1,1-addition of alkynyl selenide for better ubderstanding.
2. The cytotoxicity of Tamoxifen and derivatives (109-118) to normal cell should be assayed and provided.
3. Table S1. The yield of the 6-hour reaction time (73%) was almost the same as that of the 12-hour reaction time (76%). The effect of an extension of 6 hours on the yield did not seem to be significant. Why not shorten the reaction time?
4. Table S1. Please annotate the meaning of the abbreviation “NR”.
5. Table S1. The yield of addition reaction in this study is not high, why the authors did not screen the base to further optimize the reaction condition?
6. The purity of Tamoxifen and its derivatives for biological testing (109-118) in this study should be analyzed (e.g. HPLC) and supplemented.

Reviewer #3

(Remarks to the Author)
The manuscript titled "Diversity-Oriented Synthesis of Stereodefined Tetrasubstituted Alkenes via a Modular Alkyne gem-Addition Strategy" by Jin et al. reports a novel and effective method for the stereodefined synthesis of tetrasubstituted alkenes using a modular alkyne gem-addition strategy. The approach is innovative and important because it addresses a significant challenge in view of diversity-oriented synthesis. The authors have demonstrated a unique strategy where selenium in 1,1-carboboration offers selective formation of E-products. The performed DFT calculations have provided insight into the role of selenium in the 1,1-carboboration process. This mechanistic clarity enhances the understanding of the reaction dynamics. The scope of the study is broad, covering various substrates and demonstrating the versatility and robustness of the proposed method. The application of this method in the synthesis of drug molecules and the exploration of AIEgens and potential antitumor lead compounds further underscores the practical utility of this research.
The manuscript is well-written and the proposed strategy is both innovative and versatile, with broad implications in organic chemistry, materials science and medicinal chemistry. There are only a few issues that I would suggest the authors consider before the manuscript can be recommended for publication:
1.The authors have supported the reaction mechanism with DFT calculations. It would have been better if they had included the drawing of a simple reaction pathway so as to understand the reaction mechanism within a short time.
2.The discussion section could benefit from a more in-depth comparison with existing methodologies for the synthesis of tetrasubstituted alkenes. Highlighting the advantages and potential limitations of the current approach compared to previous strategies would provide a more balanced perspective and emphasize the novelty and impact of the work.
3.While the manuscript highlights the broad substrate scope, I would like to know if there were any limitations in this study. Are there specific substrates or functional groups not tolerable by the current reaction conditions?
The manuscript describes the involvement of selenium in the 1,1-carboboration process. Yet, it would be of great help if more experimental data can be added that compare the effect of selenium with other potential chalcogen analogs, for example, Tellurium, to confirm this special role and thus provide a further underpinning for the computational conclusions.

Version 1:

Reviewer comments:

Reviewer #1

(Remarks to the Author)
The authors have adequately revised their manuscript according to the comments and suggestions arised by the reviewers. The quality of the manuscript has been improved after the revision. The manuscript is now well publishable in Nature Communications.

Reviewer #2

(Remarks to the Author)
In my opinion the authors have adequately answered to all the points raised by the reviewer, the manuscript has been improved properly and can be accepted for publication in Nature Communications.

Open Access This Peer Review File is licensed under a Creative Commons Attribution 4.0 International License, which permits use, sharing, adaptation, distribution and reproduction in any medium or format, as long as you give appropriate credit to the original author(s) and the source, provide a link to the Creative Commons license, and indicate if changes were made.
In cases where reviewers are anonymous, credit should be given to 'Anonymous Referee' and the source.
The images or other third party material in this Peer Review File are included in the article's Creative Commons license, unless indicated otherwise in a credit line to the material. If material is not included in the article's Creative Commons license and your intended use is not permitted by statutory regulation or exceeds the permitted use, you will need to obtain permission directly from the copyright holder.
To view a copy of this license, visit https://creativecommons.org/licenses/by/4.0/

Response to the reviewers

Reviewer #1 (Remarks to the Author):

In this manuscript, the authors explored a type of meaningful alkynyl selenide substrates and demonstrated that they perform exclusive 1,1-addition selectivity when react with $ArBCl_2$. The excellent stereoselectivity of this 1,1-carboboration is satisfying, enabling the stereocontrolled construction of tetrasubstituted alkenes. To elucidate the selenide and substituent influence on the regio- and stereoselectivity, a mass of computational studies was conducted, which further disclosed the specific impact of selenium atom on the reaction mechanism, especially compared with its sulfur analogue. A large scope of functional groups, including some Lewis-acid sensitive substituents, proved of the great compatibility of this methodology. A gram-scale experiment attested the practicability of this method. On basis of the gem-addition strategy, a modular access to all-carbon tetrasubstituted alkenes was described. The authors further developed Ni-catalyzed C-Se bond activation. The selenide serving as a removable directing group could convert to various carbon substituents. The broad applications in AIE materials and medicinal chemistry are much impressive. Up to six stereoisomers of alkene with four different aryl groups was successfully prepared by this protocol. A library of tetrasubstituted alkene-based antitumor lead compounds also proved the merit of diversity-oriented synthesis in drug discovery. Overall, this solid work presents a novel programmed synthetic approach to achieving the diversity-oriented synthesis of stereodefined all-carbon tetrasubstituted alkenes, which had been a challenging issue in organic chemistry. Thus, I would like to recommend its publication in Nature Communications after some revisions and answering following questions.

(1) In the introduction, the serial number of small letters in Fig. 1 brackets is inconsistent with the numbers in Fig. 1 graphic.

Author's reply: Yes, thank you very much for your reminder. Now, we have corrected the serial number of small letters in brackets, highlighted by yellow color. Please check the introduction again.

(2) In the final product of 1,1-caboboration, there might be a coordination of Se to boron. The authors should explain whether it is favorable to the *E*-selectivity in manuscript.

Author's reply: Thank you for your suggestion. We totally agree that the coordination effect of Se to boron enables the formation of thermodynamically stable *E*-product. Since the reaction is thermodynamically controlled, we have conducted distortion-interaction analysis for transition states **TS3A** and **TS3B,** which lead to the formation of the final *E*- and *Z*-products, respectively. Our analysis reveals that the primary factor contributing to the difference in the electronic energy barriers between **TS3A** and **TS3B** is the variation in electronic interactions (**Table S5**). Additionally, the highest occupied molecular orbital (HOMO) of **TS3A** shows an extended electronic delocalization from the Se atom in fragment 1 to the C atom bonded to the B atom in fragment 2. In contrast, HOMO of **TS3B** displays a notable repulsion interaction between these atoms (**Figure S6.**). Based on these

results, it is concluded that the Se atom plays an important role in favoring the *E*-selectivity of the 1,1-caboboration process. This viewpoint was also added to the 104-107th row in the manuscript, highlighted by yellow color. We hope these clarifications and additional data address your concerns.

Table S5. Distortion-interaction analysis of **TS3A** and **TS3B**. Energies are in kcal mol^{-1}.

Transition state		$\Delta E^{‡}$	$\Delta E^{‡}_{dis}$	$\Delta E^{‡}_{int}$
**TS3A**	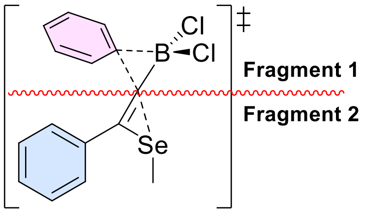	7.7	199.2	-191.5
**TS3B**	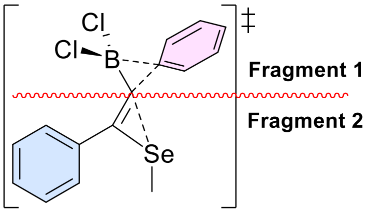	15.4	196.0	-180.6

Distortion-interaction analysis ($\Delta E^{‡} = \Delta E^{‡}_{dis} + \Delta E^{‡}_{int}$) of **TS3A** and **TS3B** shows comparable distortion energies ($\Delta E^{‡}_{dis}$) for both transition states (199.2 vs. 196.0 kcal mol^{-1}). However, the interaction energy term ($\Delta E^{‡}_{int}$) strongly favors the generation of *E*-product (-191.5 vs. -180.6 kcal mol^{-1}).

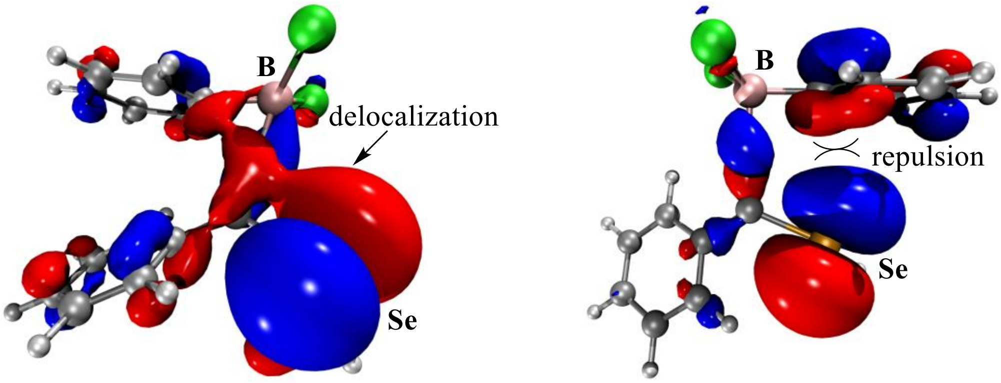

TS3A **TS3B**

Figure S6. HOMOs of **TS3A** and **TS3B** (isosurface = 0.03 a.u.).

(3) In Table 1, the C-Se bond of compound 38 is too long. The authors need to redraw the structure.

Author's reply: Thank you for your reminder. We have redrawn the structure of compound 38. Please check Table 1, compound 38.

(4) Except for forming aryl dichloroboranes from ArTMS, did authors try other approaches to access $ArBCl_2$?

Author's reply: Except for using the ArTMS to produce $ArBCl_2$, we also try to gain $ArBCl_2$ *in-situ* from the treatment of $ArBF_4K$ with $SiCl_4$. Although it is also an efficient route to access $ArBCl_2$, the final yield of 1,1-carboboration of alkynyl selenide is not satisfied (20%-30%). We suspect that the *in-situ* residues of KCl, $ArBF_2$ and $SiCl_4$ may be adverse to the following 1,1-carboboration.

(5) In Table 2, 1,1-disubstituted 1,3-enyne is not included. How about this kind of substrate?

Author's reply: Thank you for this suggestion. We prepared a 1,1-diphenyl 1,3-enyne, but it couldn't react under the standard condition. We suspect that the generated carbocation would be facilely stabilized by the di-benzene, hindering the formation of selenonium intermediate (as shown below). This substrate has been listed in Table 2 as an unsuccessful example.

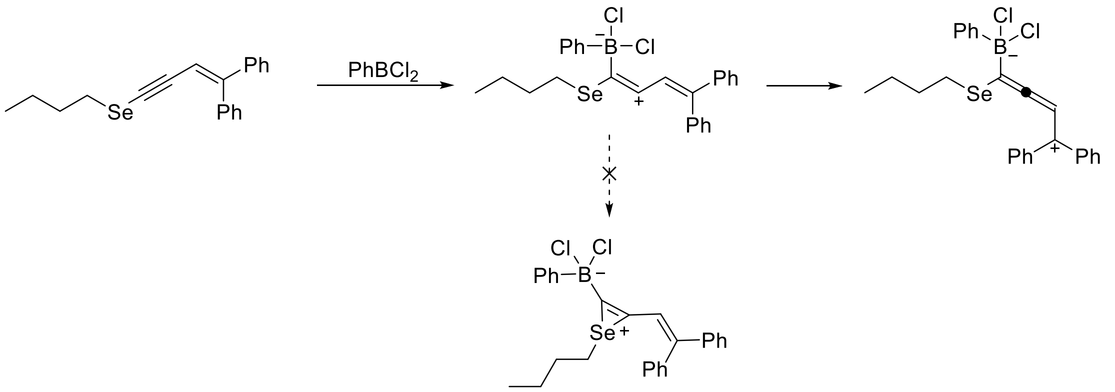

(6) In Ni-catalyzed C-Se bond transformations, is there any stereoisomer byproducts? Do authors try other transition metal catalysis?

Author's reply: We have carefully checked the NMR spectra and HPLC, there is no other stereoisomer found in Ni-catalyzed C-Se bond transformations. Before we selected Ni catalysis, we also attempted Pd-catalyzed C-Se bond activation through the formation of selenonium salts firstly (as shown below). However, the Pd-catalyzed protocol results in the generation of stereoisomers with low yield.

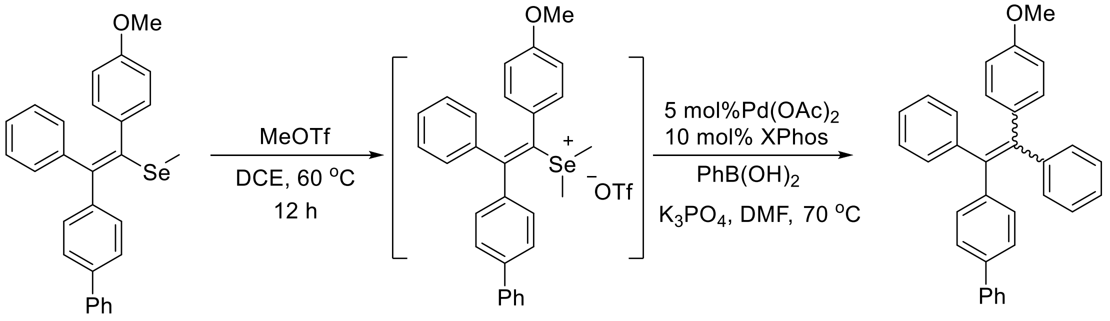

(7) Some peaks in 13C-NMR spectra are too low, such as in page S125, S139, S172, S194, S210, S215, S221. These spectra should be re-edited.

Author's reply: Thank you very much for your suggestion. These spectra have been

edited to fit the criterion. Please check the supplementary information.

(8) Some literatures about synthesis of stereodefined tetrasubstituted alkenes (such as Nat. Commun. 2021, 12, 7298, Angew. Chem. Int. Ed. 2024, 63, e202400805) should be involving.

Author's reply: Thank you for your reminder. We are pleased to cite the two references that presented modular strategies to access functionalized tetrasubstituted alkenes. Please see the reference 17 and 18, highlighted by yellow color.

Reviewer #2 (Remarks to the Author):

This manuscript describes a facile synthesis of stereodefined tetrasubstituted alkenes via a modular alkyne gem-addition strategy, in which alkynyl selenides, when treated with $ArBCl_2$, are subject to an exclusive 1,1- carboboration, affording tetrasubstituted alkenes with excellent levels of E-selectivity. The DFT calculations show that alkynyl selenide tend to undergo 1,1-addition reaction to form E-alkenes. Then, a series of fluorescent molecules and selenium derivatives of tamoxifen were synthesized. Among them, derivative 109 showed slightly better anti-breast cancer activity than tamoxifen in vitro and in vivo. In general, the strategy used in this study to stereoselectively synthesize tetrasubstituted alkenes is innovative, and the manuscript is well written and the result is robust. However, there are still some issues/problems in the manuscript that need to be addressed before ready for publication. Some of concerns are given below.

1. Based on experimental results and DFT calculations, the authors are suggested to propose a catalytic cycle with respect to the exclusive 1,1-addition of alkynyl selenide for better ubderstanding.

Author's reply: Thank you for your kind suggestion. We added a straightforward reaction mechanism for quickly understanding the 1,1-carboboration process. Please check the mechanism outline in Fig. 3 bottom.

2. The cytotoxicity of Tamoxifen and derivatives (109-118) to normal cell should be assayed and provided.

Author's reply: Thank you for your kind suggestion. We investigated the cytotoxicity of Tamoxifen and Se-containing derivatives (**109**-**118**) to the normal human mammary epithelial cell (MCF-10A). Notably, the compound **109** not only exhibits more potent antitumor activity, but also has lower cytotoxicity to the normal cell than Tamoxifen. In the 220-221st row, we clarified the cytotoxicity and the advantage of compound 109 over Tamoxifen, which was highlighted by yellow color. The IC_{50} value was added in Fig. 8. This diagram was put in supplementary information (Figure S4). We hope these clarifications and additional data address your concerns.

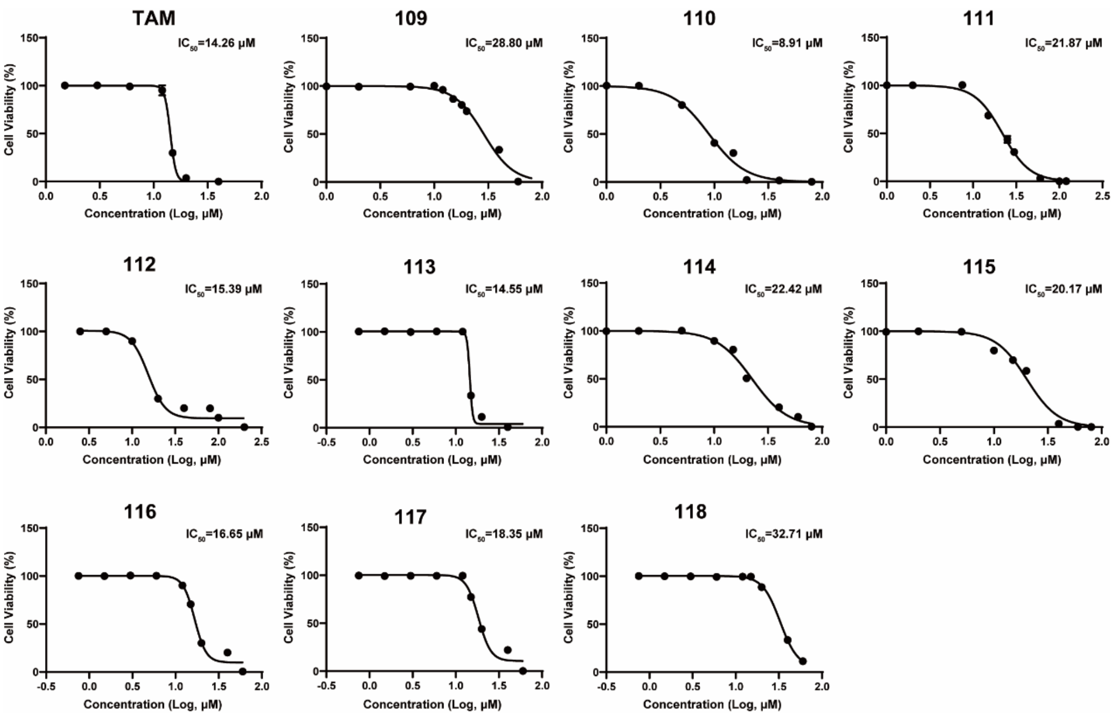

Figure S4. The cytotoxicity of compounds to normal cell (MCF-10A)

3. Table S1. The yield of the 6-hour reaction time (73%) was almost the same as that of the 12-hour reaction time (76%). The effect of an extension of 6 hours on the yield did not seem to be significant. Why not shorten the reaction time?

Author's reply: We noticed that a small amount of substrate still remains after 6 hours, thus the reaction time was extended to 12 hours for exhausting alkynyl selenide.

4. Table S1. Please annotate the meaning of the abbreviation "NR".

Author's reply: Thank you for your reminder. We added the annotation of "NR = no reaction" in Table S1.

5. Table S1. The yield of addition reaction in this study is not high, why the authors did not screen the base to further optimize the reaction condition?

Author's reply: Thank you for your kind suggestion. We checked different bases, including *i*-Pr_2NEt, pyridine, DBU and piperidine, to further optimize the yield, which were added in Table S1 (entry 12-15). However, Et_3N still proved to be optimal. The decomposition of substrate may restrain the further improvement of yield.

6. The purity of Tamoxifen and its derivatives for biological testing (109-118) in this study should be analyzed (e.g. HPLC) and supplemented.

Author's reply: Thank you for your kind suggestion. The purity of Tamoxifen and compounds (109-118) is examined by HPLC. As shown below, the purity of all compounds is higher than 96%. We also put the HPLC result of every compound in characterizations.

Compound 109

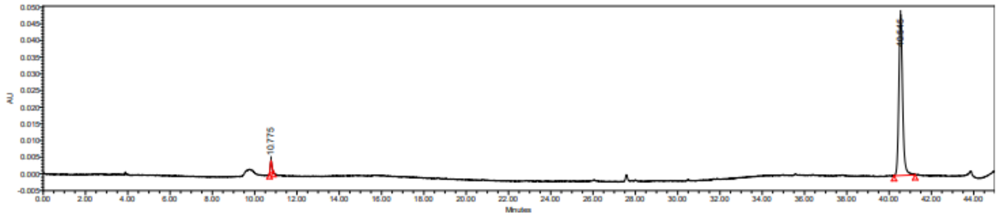

Name	Retention Time	Area	% Area	Height
1	10.775	19798	3.21	3688
2	40.545	596712	96.79	48311

Compound 110

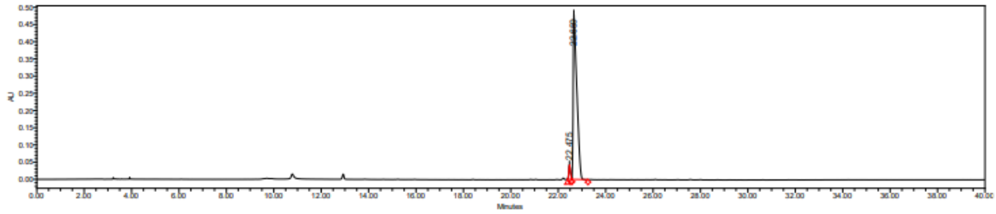

Name	Retention Time	Area	% Area	Height
1	22.475	196951	3.70	41690
2	22.659	5127062	96.30	480494

Compound 111

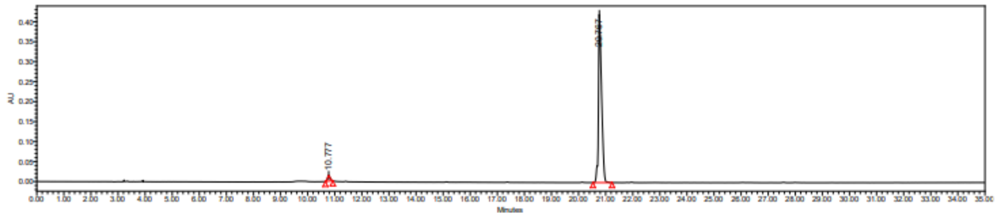

Name	Retention Time	Area	% Area	Height
1	10.777	98718	2.86	14626
2	20.767	3353948	97.14	420881

Compound 112

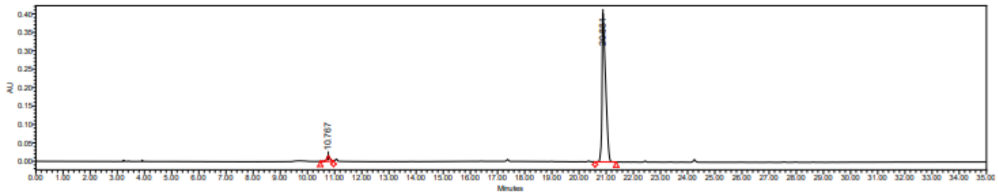

Name	Retention Time	Area	% Area	Height
1	10.767	132674	3.49	15332
2	20.881	3673003	96.51	403638

Compound 113

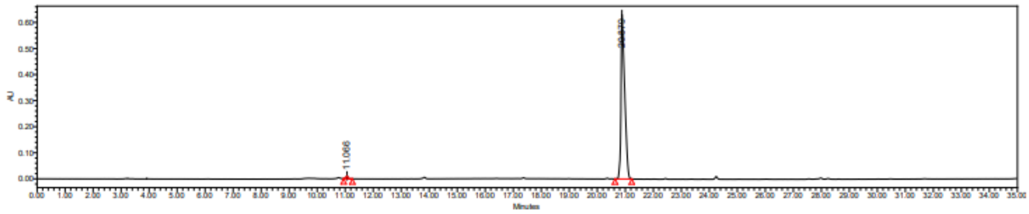

Name	Retention Time	Area	% Area	Height
1	11.066	52736	0.91	11402
2	20.879	5748056	99.09	631589

Compound 114

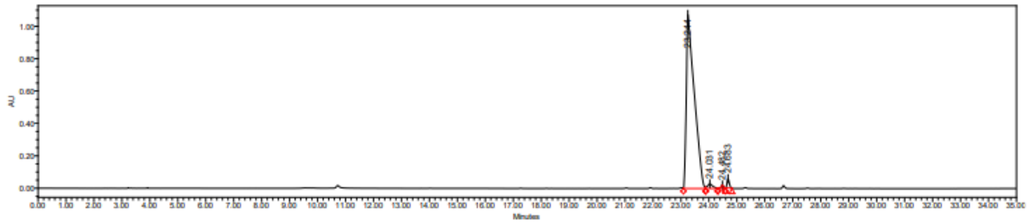

Name	Retention Time	Area	% Area	Height
1	23.244	21272601	96.24	1075014
2	24.031	372093	1.68	28897
3	24.482	141848	0.64	21464
4	24.683	317646	1.44	58045

Compound 115

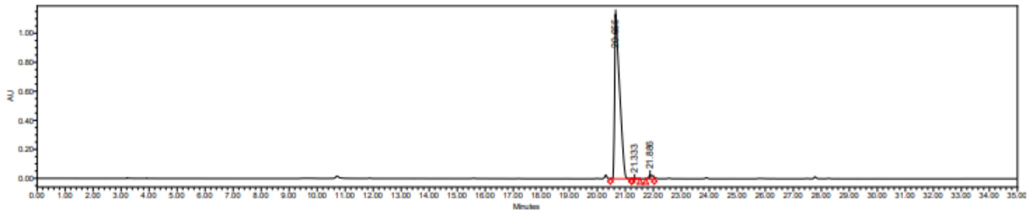

Name	Retention Time	Area	% Area	Height
2	21.333	7532	0.05	771
3	21.886	253081	1.80	26678
1	20.655	13799580	98.15	1134275

Compound 116

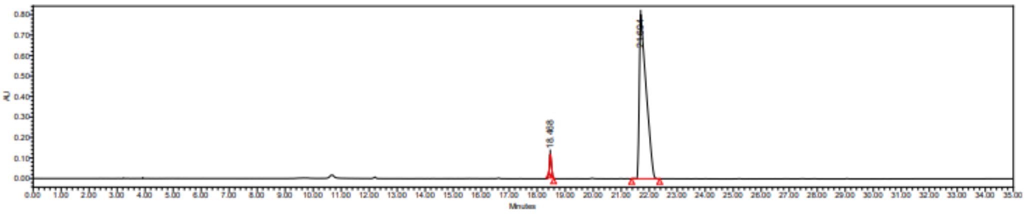

Name	Retention Time	Area	% Area	Height
1	18.468	432898	3.10	98158
2	21.694	13550296	96.90	802410

Compound 117

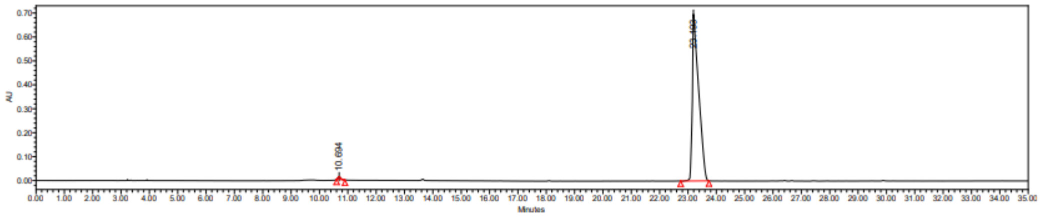

Name	Retention Time	Area	% Area	Height
1	10.694	81608	0.75	12000
2	23.193	10869002	99.25	697147

Compound 118

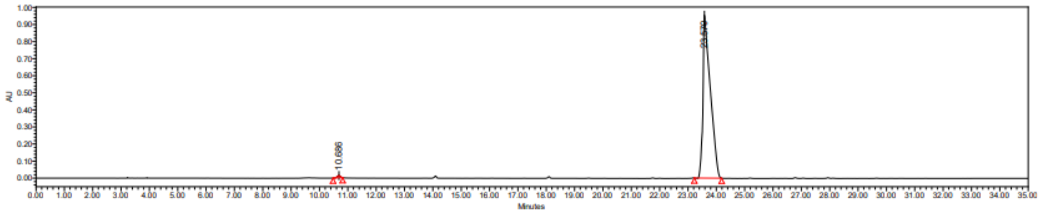

Name	Retention Time	Area	% Area	Height
1	10.686	104194	0.60	13374
2	23.579	17305979	99.40	954037

Tamoxifen

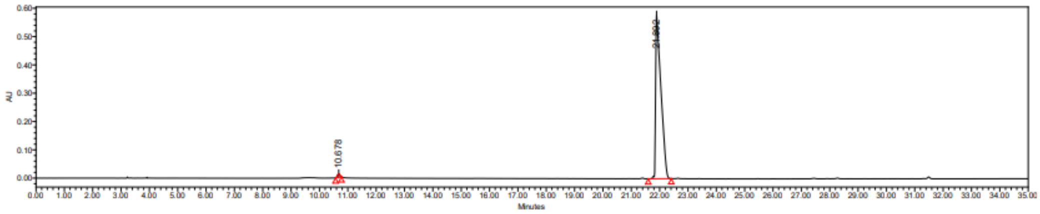

Name	Retention Time	Area	% Area	Height
1	10.678	73010	0.96	12066
2	21.892	7545462	99.04	577780

Reviewer #3 (Remarks to the Author):

The manuscript titled "Diversity-Oriented Synthesis of Stereodefined Tetrasubstituted Alkenes via a Modular Alkyne *gem*-Addition Strategy" by Jin et al. reports a novel and effective method for the stereodefined synthesis of tetrasubstituted alkenes using a modular alkyne gem-addition strategy. The approach is innovative and important because it addresses a significant challenge in view of diversity-oriented synthesis. The authors have demonstrated a unique strategy where selenium in 1,1-carboboration offers selective formation of E-products. The performed DFT calculations have provided insight into the role of selenium in the 1,1-carboboration process. This mechanistic clarity enhances the understanding of the reaction dynamics. The scope of the study is broad, covering various substrates and demonstrating the versatility and robustness of the proposed method. The application of this method in the synthesis of drug molecules and the exploration of AIEgens and potential antitumor lead compounds further underscores the practical utility of this research.

The manuscript is well-written and the proposed strategy is both innovative and versatile, with broad implications in organic chemistry, materials science and medicinal chemistry. There are only a few issues that I would suggest the authors consider before the manuscript can be recommended for publication:

1. The authors have supported the reaction mechanism with DFT calculations. It would have been better if they had included the drawing of a simple reaction pathway so as to understand the reaction mechanism within a short time.

Author's reply: Thank you for your kind suggestion. We added a straightforward reaction mechanism for quickly understanding the 1,1-carboboration process. Please see the mechanism outline in Fig. 3 bottom.

2. The discussion section could benefit from a more in-depth comparison with existing methodologies for the synthesis of tetrasubstituted alkenes. Highlighting the advantages and potential limitations of the current approach compared to previous strategies would provide a more balanced perspective and emphasize the novelty and impact of the work.

Author's reply: Thank you for your kind suggestion. We made a comprehensive comparison with existing access to tetrasubstituted alkenes with up to four different aryl groups in the 196-201st row, highlighted by yellow color. The 3 steps procedure is the shortest linear synthesis protocol for stereodefined olefins with four distinct aryl groups. Although previous methodologies also afforded efficient approaches to achieve tetraarylethylenes with excellent stereoselectivity, a small amount of regioselective isomers is still inevitable. Moreover, compared with the prior synthesis from moisture-sensitive alkynyl boron substrates, the alkynyl selenides are easily prepared, isolated and handled. Thus, the current approach may be more suitable for gram-scale synthesis. The potential limitation of this alkyne gem-addition strategy should be that the alkynyl selenides with electronic withdrawing groups usually lead to low yields. We hope these clarifications could address your concerns.

3.While the manuscript highlights the broad substrate scope, I would like to know if there were any limitations in this study. Are there specific substrates or functional groups not tolerable by the current reaction conditions?

The manuscript describes the involvement of selenium in the 1,1-carboboration process. Yet, it would be of great help if more experimental data can be added that compare the effect of selenium with other potential chalcogen analogs, for example, Tellurium, to confirm this special role and thus provide a further underpinning for the computational conclusions.

Author's reply: Thank you for your kind suggestion. Due to the Lewis-acid conditions, some Lewis-basic functional groups, such as amine, hydroxy and CN group, are not compatible. 1,1-disubstituted 1,3-enyne is also not suitable. One unsuccessful example has been listed in Table 2. For chalcogen Tellurium, we treated alkynyl telluride with $ArBCl_2$, but no reaction happened. It may owe to the larger orbital radius of Te atom that can not overlap well with the triple bond, reducing the electronic donating ability of Tellurium.

Reviewer #1 (Remarks to the Author):

The authors have adequately revised their manuscript according to the comments and suggestions arised by the reviewers. The quality of the manuscript has been improved after the revision. The manuscript is now well publishable in Nature Communications.
Thank you very much for comments.

Reviewer #2 (Remarks to the Author):

In my opinion the authors have adequately answered to all the points raised by the reviewer, the manuscript has been improved properly and can be accepted for publication in Nature Communications.
Thank you very much for comments.
